# DNA Sequencing Predicts 1st-Line Tuberculosis Drug Susceptibility Profiles

**DOI:** 10.1056/NEJMoa1800474

**Published:** 2018-09-26

**Authors:** CRyPTIC consortium, Caroline Allix-Béguec, Irena Arandjelovic, Lijun Bi, Patrick Beckert, Maryline Bonnet, Phelim Bradley, Andrea M Cabibbe, Irving Cancino-Muñoz, Mark J Caulfield, Angkana Chaiprasert, Daniela Cirillo, David Clifton, Iñaki Comas, Derrick W Crook, Maria Rosaria De Filippo, Han de Neeling, Roland Diel, Francis A Drobniewski, Kiatichai Faksri, Maha R Farhat, Joy Fleming, Philip Fowler, Tom A Fowler, Qian Gao, Jennifer Gardy, Deborah Gascoyne-Binzi, Ana Gibertoni Cruz, Ana Gil-Brusola, Tanya Golubchik, Ximena Gonzalo, Louis Grandjean, Guangxue He, Jennifer L Guthrie, Sarah Hoosdally, Martin Hunt, Zamin Iqbal, Nazir Ismail, James Johnston, Faisal Masood Khanzada, Chiea Chuen Khor, Thomas A Kohl, Clare Kong, Sam Lipworth, Qingyun Liu, Gugu Maphalala, Elena Martinez, Vanessa Mathys, Matthias Merker, Paolo Miotto, Nerges Mistry, David Moore, Megan Murray, Stefan Niemann, Rick Twee-Hee Ong, Tim E A Peto, James E Posey, Therdsak Prammananan, Alexander Pym, Camilla Rodrigues, Mabel Rodrigues, Timothy Rodwell, Gian Maria Rossolini, Elisabeth Sánchez Padilla, Marco Schito, Marco Schito, Xin Shen, Jay Shendure, Vitali Sintchenko, Alex Sloutsky, E Grace Smith, Matthew Snyder, Karine Soetaert, Angela M Starks, Philip Supply, Prapat Suriyapol, Sabira Tahseen, Patrick Tang, Yik-Ying Teo, Thuong Nguyen Thuy Thuong, Guy Thwaites, Enrico Tortoli, Shaheed Vally Omar, Dick van Soolingen, A Sarah Walker, Timothy M Walker, Mark Wilcox, Daniel J Wilson, David Wyllie, Yang Yang, Hongtai Zhang, Yanlin Zhao, Baoli Zhu

**Affiliations:** Genoscreen, Lille, France; Institute of Microbiology and Immunology, Faculty of Medicine, University of Belgrade, Belgrade, Serbia; Key Laboratory of RNA Biology, Institute of Biophysics, Chinese Academy of Sciences, Beijing, 100101, China; Forschungszentrum Borstel, Leibniz Lungenzentrum, Borstel, Germany; Epicentre, Paris, France; IRD UMI233/ INSERM U1175/ Université de Montpellier, France; European Bioinformatics Institute Hinxton, Cambridge, UK; Emerging Bacterial Pathogens Unit, WHO collaborating Centre and TB Supranational Reference laboratory, IRCCS San Raffaele Scientific institute, Milan, Italy; Instituto de Biomedicina de Valencia (IBV-CSIC). Calle Jaime Roig, Valencia, Spain; FISABIO Public Health, Valencia, Spain; Genomics England; NIHR Biomedical Research Centre at Barts. London, UK; William Harvey Research Institute, Queen Mary University of London, London, UK; Faculty of Medicine Siriraj Hospital, Mahidol University, Thailand; Emerging Bacterial Pathogens Unit, WHO collaborating Centre and TB Supranational Reference laboratory, IRCCS San Raffaele Scientific institute, Milan, Italy; Computational Health Informatics Laboratory, Department of Engineering, University of Oxford, Oxford, UK; Instituto de Biomedicina de Valencia (IBV-CSIC). Calle Jaime Roig, Valencia, Spain; FISABIO Public Health, Valencia, Spain; CIBER in Epidemiology and Public Health, Madrid, Spain; Nuffield Department of Medicine, John Radcliffe Hospital, University of Oxford, Oxford, UK, National Infection Service, Public Health England, UK; Emerging Bacterial Pathogens Unit, WHO collaborating Centre and TB Supranational Reference laboratory, IRCCS San Raffaele Scientific institute, Milan, Italy; National Institute for Public Health and the Environment (RIVM), Bilthoven, The Netherlands; Institute for Epidemiology, University Medical Hospital Schleswig-Holstein, Kiel, Airway Research Center North (ARCN), Kiel, Germany; Imperial College, London, UK; Department of Microbiology, Faculty of Medicine, Khon Kaen University, Thailand; Harvard Medical School, Boston, USA; Key Laboratory of RNA Biology, Institute of Biophysics, Chinese Academy of Sciences, Beijing, 100101, China; Nuffield Department of Medicine, John Radcliffe Hospital, University of Oxford, Oxford, UK; Genomics England; Shanghai Medical College, Fudan University, Shanghai, China; British Columbia Centre for Disease Control, Vancouver, Canada; University of British Columbia, Vancouver, Canada; Leeds Teaching Hospital NHS Trust, Leeds, UK; Nuffield Department of Medicine, John Radcliffe Hospital, University of Oxford, Oxford, UK; Hospital Universitario y Politécnico La Fe, Avinguda de Fernando Abril Martorell, Valencia, Spain; Big Data Institute, Old Road Campus, University of Oxford, Oxford, UK; Imperial College, London, UK; National Mycobacterial Reference Laboratory, Public Health England, London, UK; Dept of Infectious Disease Epidemiology, London School of Hygiene and Tropical Medicine, London, UK; Chinese Centre of Disease Control and Prevention, Beijing, China; British Columbia Centre for Disease Control, Vancouver, Canada; Nuffield Department of Medicine, John Radcliffe Hospital, University of Oxford, Oxford, UK; European Bioinformatics Institute Hinxton, Cambridge, UK; Nuffield Department of Medicine, John Radcliffe Hospital, University of Oxford, Oxford, UK; European Bioinformatics Institute Hinxton, Cambridge, UK; National Institute for Communicable Diseases (NICD), Johannesburg, South Africa; British Columbia Centre for Disease Control, Vancouver, Canada; National TB Reference Laboratory, National TB control Program , Islamabad , Pakistan; Genome Institute of Singapore, Agency for Science, Technology and Research; Forschungszentrum Borstel, Leibniz Lungenzentrum, Borstel, Germany; British Columbia Centre for Disease Control, Vancouver, Canada; Nuffield Department of Medicine, John Radcliffe Hospital, University of Oxford, Oxford, UK; Key Laboratory of Medical Molecular Virology, School of Basic Medicine, Fudan University; National Reference Laboratory (NRL), Ministry of Health, Mbabane, Swaziland; Centre for Infectious Diseases and Microbiology, Westmead Hospital, Wentworthville New South Wales, Australia; Bacterial Diseases Service, Operational Direction Communicable and Infectious Diseases, Scientific Institute of Public Health (WIV-ISP), Brussels, Belgium; Forschungszentrum Borstel, Leibniz Lungenzentrum, Borstel, Germany; Emerging Bacterial Pathogens Unit, WHO collaborating Centre and TB Supranational Reference laboratory, IRCCS San Raffaele Scientific institute, Milan, Italy; Foundation for Medical Research, Mumbai, India; Dept of Infectious Disease Epidemiology, London School of Hygiene and Tropical Medicine, London, UK; Harvard Medical School, Boston, USA; Forschungszentrum Borstel, Leibniz Lungenzentrum, Borstel, Germany; German Center for Infection Research, Borstel Site, Borstel, Germany; Saw Swee Hock School of Public Health, National University of Singapore, Singapore; Nuffield Department of Medicine, John Radcliffe Hospital, University of Oxford, Oxford, UK; Centres for Disease Control and Prevention, Atlanta, Georgia, USA; Biotec, National Science and Technology Development Agency, Thailand; Africa Health Research Institute (AHRI), Nelson Mandela School of Medicine, UKZN, Durban, South Africa; Department of Microbiology, Hinduja Hospital, Mumbai, India; British Columbia Centre for Disease Control, Vancouver, Canada; University of California, San Diego, La Jolla, USA; Foundation for Innovative and New Diagnostics (FIND), Geneva, Switzerland; Clinical Microbiology and Virology Unit, Florence - Careggi University Hospital; Epicentre, Paris, France; University of California, San Diego, La Jolla, USA; Foundation for Innovative and New Diagnostics (FIND), Geneva, Switzerland; Critical Path Institute, Tucson, Arizona, USA 85718; Shenzhen Center for Chronic Disease Control, Shenzhen, China; University of Washington, Seattle, USA; Centre for Infectious Diseases and Microbiology - Public Health, University of Sydney, Sydney, Australia; University of Massachusetts Medical School Worcester, MA, USA 01655; Massachusetts State Tuberculosis Laboratory Jamaica Plain, MA, USA 02130-3597; National Mycobacterial Reference Service, Public Health England Public Health Laboratory Birmingham, Birmingham, UK; University of Washington, Seattle, USA; Bacterial Diseases Service, Operational Direction Communicable and Infectious Diseases, Scientific Institute of Public Health (WIV-ISP), Brussels, Belgium; Centres for Disease Control and Prevention, Atlanta, Georgia, USA; Genoscreen, Lille, France; Univ. Lille, CNRS, Inserm, CHU Lille, Institut Pasteur de Lille, U1019 - UMR 8204 - CIIL - Centre d'Infection et d'Immunité de Lille, F-59000 Lille, France; Faculty of Medicine Siriraj Hospital, Mahidol University, Thailand; National TB Reference Laboratory, National TB control Program , Islamabad , Pakistan; British Columbia Centre for Disease Control, Vancouver, Canada; Department of Pathology, Sidra Medical and Research Center, Doha, Qatar; Saw Swee Hock School of Public Health, National University of Singapore, Singapore; Life Sciences Institute, National University of Singapore; Genome Institute of Singapore, Agency for Science, Technology and Research; Oxford University Clinical Research Unit, Hosptial for Tropical Disease, Ho Chi Minh City, Vietnam; Oxford University Clinical Research Unit, Hosptial for Tropical Disease, Ho Chi Minh City, Vietnam; Emerging Bacterial Pathogens Unit, WHO collaborating Centre and TB Supranational Reference laboratory, IRCCS San Raffaele Scientific institute, Milan, Italy; National Institute for Communicable Diseases (NICD), Johannesburg, South Africa; National Institute for Public Health and the Environment (RIVM), Bilthoven, The Netherlands; Nuffield Department of Medicine, John Radcliffe Hospital, University of Oxford, Oxford, UK; Nuffield Department of Medicine, John Radcliffe Hospital, University of Oxford, Oxford, UK; Leeds Teaching Hospital NHS Trust, Leeds, UK; University of Leeds, Leeds, UK; Nuffield Department of Medicine, John Radcliffe Hospital, University of Oxford, Oxford, UK; Nuffield Department of Medicine, John Radcliffe Hospital, University of Oxford, Oxford, UK; Computational Health Informatics Laboratory, Department of Engineering, University of Oxford, Oxford, UK; Oxford-Suzhou China Advance Research Center, Shanghai, China; Key Laboratory of RNA Biology, Institute of Biophysics, Chinese Academy of Sciences, Beijing, 100101, China; National Tuberculosis Reference Laboratory of Chinese Centre of Disease Control and Prevention, Beijing, China; Microbial Genome Research Center, Chinese Academy of Sciences, Beijing, China

## Abstract

**Background:**

The World Health Organization recommends universal drug susceptibility testing for *Mycobacterium tuberculosis* complex to guide treatment decisions and improve outcomes. We assessed whether DNA sequencing can accurately predict antibiotic susceptibility profiles for first-line anti-tuberculosis drugs.

**Methods:**

Whole-genome sequences and associated phenotypes to isoniazid, rifampicin, ethambutol and pyrazinamide were obtained for isolates from 16 countries across six continents. For each isolate, mutations associated with drug-resistance and drug-susceptibility were identified across nine genes, and individual phenotypes were predicted unless mutations of unknown association were also present. To identify how whole-genome sequencing might direct first-line drug therapy, complete susceptibility profiles were predicted. These were predicted to be pan-susceptible if predicted susceptible to isoniazid and to other drugs, or contained mutations of unknown association in genes affecting these other drugs. We simulated how negative predictive value changed with drug-resistance prevalence.

**Results:**

10,209 isolates were analysed. The greatest proportion of phenotypes were predicted for rifampicin (9,660/10,130; (95.4%)) and the lowest for ethambutol (8,794/9,794; (89.8%)). Isoniazid, rifampicin, ethambutol and pyrazinamide resistance was correctly predicted with 97.1%, 97.5% 94.6% and 91.3% sensitivity, and susceptibility with 99.0%, 98.8%, 93.6% and 96.8% specificity, respectively. 5,250 (89.5%) drug profiles were correctly predicted for 5,865/7,516 (78.0%) isolates with complete phenotypic profiles. Among these, 3,952/4,037 (97.9%) predictions of pan-susceptibility were correct. The negative predictive value for 97.5% of simulated drug profiles exceeded 95% where the prevalence of drug-resistance was below 47.0%.

**Conclusions:**

Phenotypic testing for first-line drugs can be phased down in favour of DNA sequencing to guide anti- tuberculosis drug therapy.

*Mycobacterium tuberculosis* killed more people than any other pathogen in 2016, when over 10 million active cases were estimated, and 1.7 million patients died.^1^ In 2014, the World Health Organization (WHO) set a target to ‘END TB’ by 2035, acknowledging that success depends on the development of better preventative, diagnostic and therapeutic interventions. The global emergence of antimicrobial resistance poses a major challenge. Despite a call for universal access to drug susceptibility testing to direct individualised therapies, high costs and skills shortages mean it is unavailable in many countries with greatest need. Consequently, only 22% of an estimated 600,000 patients requiring treatment for multidrug-resistant tuberculosis were diagnosed and treated in 2016,^1^ facilitating the onward transmission of multidrug-resistant strains.^2^

The Xpert MTB/RIF (Cepheid, Sunnyvale, California, USA) assay has partially eased the global diagnostic need. It uses polymerase chain reaction technology to identify both *M. tuberculosis* complex and mutations in the *rpoB* gene (predictive of multidrug resistance) directly from clinical samples.^3^ However, as it targets only a few potential resistance-conferring mutations, antimicrobial susceptibility cannot be reliably inferred from a negative result.^4^ To direct individualised therapies, a diagnostic assay is needed to determine which drugs to give, in addition to which to avoid. 

Advances in whole-genome sequencing mean it is now the most promising solution to the need for universal drug susceptibility testing. It is faster, more scalable, and likely to become cheaper than phenotypic testing.^5^ As the number of genomic sites whole-genome sequencing covers are virtually unrestricted, it should be possible to infer *M. tuberculosis* antimicrobial susceptibility from the absence of resistance-conferring mutations.^6^ Here we assess how well this performs for first-line anti- tuberculosis drugs, considering WHO target product profiles for new molecular assays,^7^ and whether whole-genome sequencing can be used to accurately direct anti-tuberculosis therapy.

## Methods

### Sample selection

Collections of *M. tuberculosis* complex isolates unenriched for resistance and largely sequenced prospectively for routine diagnostic reasons, or for disease surveillance, were included from Germany, Italy, the Netherlands and the UK. Collections enriched for antimicrobial resistance, were included from across six continents (Table1, Supplement S1). Analyses of both the unenriched and complete collection were planned.

**Table 1:** Number of isolates by country and drug resistance profile

Country of sample origin	Time period of isolation	Enriched for resistance	Susceptible to all 4 drugs	Susceptible to 3 drugs, with missing pyrazinamide result	Isoniazid resistant, rifampicin susceptible	Isoniazid susceptible, rifampicin resistant	Isoniazid resistant, rifampicin resistant	Other pattern	Total
Australia	2006-2016	Yes	0	0	4	0	38	0	42
Belgium	2007-2015	Yes	121	0	2	0	97	14	234
Canada	2003-2014	Yes	11	1,118	164	14	24	12	1343
China	2009-2012	Yes	0	44	0	0	236	0	280
Germany	1998-2015	No	248	0	9	1	13	2	273
Italy	2008-2016	Yes and No*	82	1	9	0	132	2	226
Netherlands	1993-2016	No	420	42	24	1	149	31	667
Pakistan	2014-2015	Yes	47	5	11	6	345	1	415
Peru	1997-2009	Yes	24	12	49	18	199	13	315
Russia	2008-2010	Yes	282	0	116	15	407	22	842
Serbia	2008-2014	Yes	0	0	0	0	105	0	105
South Africa	2012-2014	Yes	593	11	37	69	151	130	991
Spain	2013-2015	Yes	45	3	5	2	8	1	64
Swaziland	2009-2010	Yes	2	130	14	4	116	7	273
Thailand	1998-2013	Yes	0	53	7	4	188	0	252
UK	2009-2017	Yes and No*	3,036	82	167	6	442	154	3,887
Total			4911	1501	618	140	2650	389	10209

* More than one collection was derived from Italy and the UK, some enriched and some not enriched for resistance. See supplement for details.

### Sequencing

Isolates were sequenced on Illumina platforms and reads processed by the Public Health England bioinformatics pipeline at Genomics England,^8^ as described.^6^ Reads were mapped to the pan- susceptible *M. tuberculosis* reference genome (Genbank NC_000962.2) using Stampy (v.1.0.17)^9^, with repetitive regions masked. SAMtools mpileup^10^ (v.0.1.18) made variant-calls based on a minimum depth of 5X and at least one read on each strand. Mixed-calls were assigned where minority alleles composed >10% of read depth. Insertions and deletions were determined using Cortex (v.1.0.5.21).^11^

### Drug susceptibility testing and prediction

Phenotypic drug susceptibility testing was performed locally using MGIT 960 (Becton Dickinson, New Jersey, USA), 7H10 or Löwenstein-Jensen agar, or by microscopic-observation drug- susceptibility (MODS), with method-specific critical concentrations for isoniazid (MGIT 0.1-0.2μg/mL; Agar 0.2μg/mL; MODS 0.4μg/mL), rifampicin (MGIT 1.0μg/mL; 40μg/mL Agar), ethambutol (MGIT 5.0μg/mL; Agar 0.2μg/mL), and pyrazinamide (100μg/mL). Not all laboratories routinely tested all agents (S1). Genotypic predictions were based on mutations in, or upstream of, genes associated with resistance to isoniazid (*ahpC, inhA, fabG1, katG*), rifampicin (*rpoB*), ethambutol (*embA, embB, embC*), and pyrazinamide (*pncA*).^6^ A knowledgebase of mutations predicting antimicrobial resistance, or not, was informed by (i) the molecular targets of WHO-recommended line-probe assays (MTBDR*plus*, MTBDR*sl* v1.0, HAIN Lifesciences, Germany), (ii) a systematic literature review,^12^ (iii) the CDC, Atlanta, USA, panel and (iv) two recent studies, with no isolates in common with this study (S2),^6,13^ of which one became available after this study commenced.^13^

Isolates containing resistance-mutations were predicted phenotypically resistant, whereas isolates containing only wild-type sequence, phylogenetic mutations,^6^ or mutations considered consistent with susceptibility, were predicted susceptible. Predictions were withheld for isolates containing mutations affecting target genes but of unknown association, or where no nucleotide-call could be determined at a resistance-associated site. In these circumstances, the genotype was reported ‘unknown’ or ‘failed’, respectively. Using phenotypic results as a gold-standard, sensitivity, specificity, negative and positive predictive value were calculated for the correct assignment of susceptibility or resistance. Primary analyses excluded phenotypes without a prediction.

Laboratory error was assumed where three or more phenotypes were discordant with an isolate’s genotype, or where susceptible phenotypes were recorded despite the presence of high-level resistance *katG* S315T mutations for isoniazid, or *rpoB* S450L mutations for rifampicin.^14^ Such isolates were excluded from further analysis. 

Analysis was performed using STATA (Texas, USA, v13.1). No institutional review board approval was required except in Thailand, it was granted through Mahidol University (Si029/2557).

The study was first designed by TMW,TEAP,DWC, with subsequent contributions from others (supplement). Data were gathered at participating centres. Initial analysis was performed by TMW,TEAP,ASW,ZI,MH,SL,DW,PF,PM with later input from others (supplement). TMW wrote the first draft. TMW vouches for the analysis and had full access to the data; all authors agreed to publication.

## Results

10,290 isolates were available for the study. 81 (0.8%) were excluded due to likely laboratory error. 10,209 isolates remained, for which full first-line phenotypic profiles were available for 7,516 (73.6%), and partial profiles for the remainder. 4,911 (48.1%) isolates were phenotypically susceptible to all drugs (Table 1).

For each isolate, the complete sequence of nine genes and their promoter regions was interrogated to make genotypic predictions of each available phenotypic result. Predictions could be made for 8,405/8,976 (93.6%) resistant and 26,879/28,746 (93.5%) susceptible phenotypes. The remainder contained uncharacterised mutations, or missing key nucleotide calls. For isoniazid and rifampicin, ethambutol and pyrazinamide, sensitivity (proportion of resistant phenotypes predicted resistant) was 97.1%, 97.5%, 94.6% and 91.3%, and specificity (proportion of susceptible phenotypes predicted susceptible) was 99.0%, 98.8%, 93.6% and 96.8%, respectively. By comparison, an in-silico prediction of the results that would have been obtained from WHO-recommended molecular assays (Xpert MTB/RIF, MTBDR*plus*, MTBDR*sl* v1.0) had a significantly lower sensitivity than whole-genome sequencing for isoniazid, rifampicin and ethambutol (p<0.001), but greater specificity for isoniazid and ethambutol (p<0.001) (Table 2a,b). 

**Table 2:** Prediction of individual drug phenotypes

	Resistant phenotype, n (%)					Susceptible phenotype, n (%)												
	R	S	U	F	Total	R	S	U	F	Total	Sensitivity of predictions, %(95% CI)	Specificity of predictions, % (95% CI)	PPV, % (95% CI)	NPV, % (95% CI)	Sensitivity (all*), %	Specificity (all*), %	No genotypic prediction made, %	Resistance prevalence (all), %
(a) All isolates																		
Isoniazid	3067	90	93	44	3294	65	6313	215	117	6710	97.1 (96.5-97.7)	99.0 (98.7-99.2)	97.9 (97.4-98.4)	98.6 (98.3-98.9)	93.1	94.1	4.7	32.9
Rifampicin	2743	69	7	84	2903	85	6763	232	147	7227	97.5 (96.9-98.1)	98.8 (98.5-99.0)	97.0 (96.3-97.6)	99.0 (98.7-99.2)	94.5	93.6	4.6	28.7
Ethambutol	1410	81	94	55	1640	468	6835	781	70	8154	94.6 (93.3-95.7)	93.6 (93.0-94.1)	75.1 (73.0-77.0)	98.8 (98.5-99.1)	86.0	83.8	10.2	16.7
Pyrazinamide	863	82	117	77	1139	204	6146	197	108	6655	91.3 (89.3-93.0)	96.8 (96.3-97.2)	80.9 (78.4-83.2)	98.7 (98.4-99.0)	75.8 92.4	6.4	14.6
(b) In silico prediction of performance of MTB/RIF Xpert and HAIN MTBDRplus/MTBDRsl line-probe assays for all isolates																		
Isoniazid	2886	355	53	3294	27	6675	8	6710	89.0 (87.9-90.1)†	99.6 (99.4-99.7)†	99.1 (98.7-99.4)†	95.0 (94.4-95.5)†	0.6 32.9
Rifampicin	2669	143	91	2903	129	6826	272	7227	94.9 (94.0-95.7)†98.1	(97.8-98.4)‡	95.4 (94.5-96.1)‡	97.9 (97.6-98.3)†	3.6	28.7
Ethambutol	961	641	38	1640	241	7895	18	8154	60.0 (57.5-62.4)†	97.0 (96.6-97.4)†	80.0 (77.6-82.2)‡	92.5 (91.9-93.0)†	0.6	16.7
Pyrazinamide																		
(c) Collections from Germany, Italy, the Netherlands and the UK, unenriched for resistance																		
Isoniazid	314	8	9	4	335	15	3770	104	90	3979	97.5 (95.2-98.9)	99.6 (99.3-99.8)†	95.4 (92.6-97.4)‡	99.8 (99.6-99.9)†	93.7	94.7	4.8	7.8
Rifampicin	126	0	0	9	135	31	3958	103	116	4208	100.0 (97.1-100.0)	99.2	(98.9-99.5)§	80.3 (73.2-86.2)†	100.0 (99.9-100.0)†	93.3	94.1	5.2	3.1
Ethambutol	72	1	0	0	73	47	3711	458	36	4252	98.6 (92.6-100.0)	98.7 (98.3-99.1)†	60.5 (51.1-69.3)†	100.0 (99.8-100.0)†	98.6	87.3	11.4	1.7
Pyrazinamide	109	6	4	6	125	30	4003	14	58	4105	94.8 (89.0-98.1)	99.3 (98.9-99.5)†	78.4 (70.6-84.9)	99.9 (99.7-99.9)†	87.2	97.5	1.9	3.0
(d) In silico prediction of performance of MTB/RIF Xpert and HAIN MTBDRplus/MTBDRsl line-probe assays for collections unenriched for resistance																		
Isoniazid	295	36	4	335	10	3965	4	3979	89.1 (85.3-92.3)†	99.7 (99.5-99.9)	96.7 (94.1-98.4)	99.1 (98.8-99.4)†	0.2
Rifampicin	114	11	10	135	22	3957	229	4208	91.2 (84.8-95.6)†	99.4 (99.2-99.7)	83.8 (76.5-89.6)	99.7 (99.5-99.9)†	5.5
Ethambutol	57	16	0	73	29	4220	3	4252	78.1 (66.9-86.9)†	99.3 (99.0-99.5)§	66.3 (55.3-76.1)	99.6 (99.4-99.8)†	0.1
Pyrazinamide																		

PPV = Positive Predictive Value; NPV = Negative Predictive Value; R=resistant; S=susceptible; U=mutation of unknown association present; F=genotypic prediction failed due to missing data around a genomic resistance locus; All % results based on R/S genotypic predictions only, excluding U and F except where * for which denominator includes R, S, U and F. †p≤0.001 , ‡p≤0.01, and §p≤0.05 comparing sensitivity, specificity, NPV and PPV for each drug for (b) and (c) against (a), and comparing (d) against (c); p>0.05 for all results not marked †, ‡ or §. In silico predictions of resistance for Xpert and HAIN assays were based on the presence of non-wild type sequence within the genomic regions interrogated by these assays. 'F' was reported in the presence of minority alleles at relevant sites, just as for WGS predictions.

The negative predictive value (proportion of concordant susceptible predictions) was over 98.5% for all four drugs. Although dependent on prevalence, this also varied with isolates’ background phenotypic profiles. For example, at 20% prevalence of pyrazinamide resistance, the expected negative predictive value for pyrazinamide was 93.6% and 99.0% for isolates susceptible and resistant to the other three drugs, respectively (Table 3, S3).

**Table:** 

	Phenotypic profiles	R	S	U	F	Total	R	S	U	F	Total	Prevalence of resistance among each of the listed drug profiles, %	Sensitivity, %	Specificity, %	PPV, %	NPV,%	Expected NPV at given prevalence of resistance based on simulations, % (95% CI)*	Calculated NPV at 20% prevalence of resistance, % (see table S3)	Calculated NPV at 40% prevalence of resistance, % (see table S3)
Isoniazid	-SSS	391	30	18	12	451	21	4,653	133	104	4,911	8.4	93	100	95	99.4	99.3-100	98.2	95.4
	-RSS	459	21	20	6	506	7	85	5	1	98	83.8	96	92	98	80.2	83.5-100	98.8	96.9
	-RRS	424	3	13	4	444	2	2	2	0	6	98.7	99	50	100	40.0	73.7-85.6	99.6	99.1
	-SRS	24	4	1	0	29	0	10	1	0	11	72.5	86	100	100	71.4	90.5-95.6	96.6	91.3
	-SSR	24	1	2	1	28	0	95	6	3	104	21.2	96	100	100	99.0	98.5-99.7	99	97.4
	-RRR	662	3	11	4	680	0	0	0	0	0	100.0	100	.	100	0.0	73.7-85.6	n/a	n/a
	-RSR	217	3	5	5	230	0	3	0	0	3	98.7	99	100	100	50.0	73.7-85.6	99.7	99.1
	-SRR	13	0	0	2	15	0	0	0	0	0	100.0	100	.	100	.	73.7-85.6	n/a	n/a
																			
Rifampicin	S-SS	74	16	0	8	98	30	4,632	126	123	4,911	2.0	82	99	71	99.7	99.3-100	95.7	89.3
	S-RS	6	0	0	0	6	1	9	1	0	11	35.3	100	90	86	100.0	97.8-99.5	100	100
	S-SR	1	2	0	0	3	0	100	3	1	104	2.8	33	100	100	98.0	99.3-100	85.7	69.2
	S-RR	0	0	0	0	0	0	0	0	0	0	.	.	.	.	.	.	n/a	n/a
	R-SS	464	20	1	21	506	18	424	3	6	451	52.9	96	96	96	95.5	95.8-98.6	98.9	97.2
	R-RS	424	7	2	11	444	4	25	0	0	29	93.9	98	86	99	78.1	76.2-86.6	99.5	98.8
	R-SR	218	4	0	8	230	7	20	0	1	28	89.1	98	74	97	83.3	77.9-87.9	99.4	98.4
	R-RR	665	2	0	13	680	10	3	0	2	15	97.8	100	23	99	60.0	76.2-86.6	99.7	99.1
																			
Ethambutol	SS-S	1	9	1	0	11	4	4,399	472	36	4,911	0.2	10	100	20	99.8	98.8-99.9	81.6	62.5
	RS-S	21	5	3	0	29	31	376	40	4	451	6.0	81	92	40	98.7	98.8-99.9	95.1	87.8
	SR-S	4	2	0	0	6	1	93	3	1	98	5.8	67	99	80	97.9	98.8-99.9	92.2	81.7
	RR-S	375	20	30	19	444	203	241	48	14	506	46.7	95	54	65	92.3	93.4-96.7	97.7	94.1
	SS-R	0	0	0	0	0	1	81	22	0	104	0.0	.	99	0	100.0	98.8-99.9	n/a	n/a
	RS-R	12	2	1	0	15	7	20	1	0	28	34.9	86	74	63	90.9	95.7-98.1	95.4	88.6
	SR-R	0	0	0	0	0	0	3	0	0	3	0.0	.	100	.	100.0	98.8-99.9	n/a	n/a
	RR-R	625	9	26	20	680	150	50	25	5	230	74.7	99	25	81	84.7	82.0-88.2	98.6	96.4
																			
Pyrazinamide	SSS-	74	28	0	2	104	12	4,826	13	60	4,911	2.1	73	100	86	99.4	98.6-99.6	93.6	84.5
	RSS-	13	8	4	3	28	5	431	2	13	451	5.8	62	99	72	98.2	98.6-99.6	91.2	79.6
	RRS-	166	25	22	17	230	49	374	68	15	506	31.3	87	88	77	93.7	95.5-97.7	96.4	91
	SRS-	0	3	0	0	3	0	97	0	1	98	3.0	0	100	.	97.0	98.6-99.6	80	60
	RRR-	532	15	83	50	680	107	216	105	16	444	60.5	97	67	83	93.5	87.3-91.0	99	97.3
	SRR-	0	0	0	0	0	0	6	0	0	6	0.0	.	100	.	100.0	98.6-99.6	n/a	n/a
	RSR-	10	2	1	2	15	0	28	0	1	29	34.1	83	100	100	93.3	95.0-97.3	96	90
	SSR-	0	0	0	0	0	0	11	0	0	11	0.0	.	100	.	100.0	98.6-99.6	n/a	n/a
																			

Phenotypic profiles are listed in the following order: Isoniazid, Rifampicin, Ethambutol, Pyrazinamide. '-' under 'Phenotypic profiles' marks the drug phenotype being assessed. PPV = Positive Predictive Value; NPV = Negative Predictive Value; R=resistant; S=susceptible; U=mutation of unknown association present; F=genotypic prediction failed due to missing data around a genomic resistance locus; All % results based on R/S genotypic predictions only, excluding U and F. Expected NPV was calculated as follows: specificity x (1-prevlence) / (specificity x (1-prevlence)+(1-sensitivity) x prevalence). * indicates that for prevalence <10% or >90%, simulated values are given for 10% and 90% respectively as simulations were not performed below or above these values.

As some collections included clustered isolates, the analysis was repeated after randomly selecting one representative among genomically indistinguishable isolates, and again from isolates within five single nucleotide polymorphisms of another. No significant change in sensitivity or specificity was observed for any drugs (p>0.1, S4).

To reflect the emerging practice of routinely sequencing isolates for clinical care, the analysis was repeated for the subset of 4,397 isolates from German, Italian, Dutch and UK collections that were not enriched for resistance. Among these isolates, 335 (7.6%) were isoniazid resistant and 125 (2.8%) multidrug-resistant. For each drug, specificity and negative predictive values increased, whilst positive predictive values (the proportion of concordant resistant predictions) decreased relative to the overall results. There was no significant change in sensitivity (Table 2c).

### Predicting complete phenotypic profiles

For DNA sequencing to help individualise therapy, a minimum requirement is that all first-line antimicrobial phenotypes are predicted. Phenotypic profiles were thus predicted for 7,516 isolates with phenotypic data available for all first-line drugs (S1&6). ‘Unknown’ or ‘failed’ was reported for at least one drug for 1,651 (22.0%) profiles. 5,865 (78.0%) were predicted completely, of which 5,250 (89.5%) were predicted correctly (S5). Among the 5,865 profiles, 4,007 were phenotypically pan-susceptible, of which 3952 (98.6%) were predicted correctly (Table 4).

**Table 4:** Genotypic drug profile predictions of pan-susceptibility

Prediction	Genotypic drug profile				Number predicted to have drug profile	Number predicted to have drug profile that are phenotypic ally pansusceptible (%)	Sensitivity %	Specificity %	PPV %	NPV %	Predictions made %
	Inh	Rif	Emb	Pza							
(a) Predicted pan-susceptible	S	S	S	S	4,037	3952 (97.9)					

(b) Predicted pansusceptible after inferring that 'U' mutations are consistent with susceptibility in this context	S	S	S	U	11	11 (100)					
	S	S	U	S	410	399 (97.3)					
	S	S	U	U	2	2 (100)					
	S	U	S	S	93	88 (94.6)					
	S	U	U	S	29	29 (100)					
	Total				4,582	4481 (97.8)					
(c) Predicted to have some phenotypic resistance	R	S	R or S		397	18 (4.5)					
	S	At least one R, no U or F			158	36 (22.8)					
	R	R	R or S		1273	1 (0.1)					
	Total				1828	55 (3.0)					
							95.4	98.6	97.0	97.9	78.0
							94.6	98.8	97.0	97.8	85.1
No prediction made (drug profile prediction incomplete)	U	S or U			150	126 (84.0)					
	At least one F, no R				280	240 (85.7)					
	At least one R and U, no F				499	6 (1.2)					
	At least one R and F, no U				159	3 (1.9)					
	At least one R, U, and F				18	0 (0.0)					
	Total				1106	375 (33.9)					

As the proportion of incompletely predicted profiles was substantial (22.0%), we assessed whether pan-susceptibility could be accurately predicted for some of these isolates anyway. Because isoniazid susceptibility predicts susceptibility to other first-line drugs,^15^ we maximised confidence in isoniazid predictions by conditioning predictions on the absence of ‘unknown’ mutations in isoniazid- related genes. ‘Unknown’ mutations relevant to other drugs were permitted. Doing this, pan- susceptibility was correctly predicted for 4,481/4,582 (97.8%) isolates, including 545/1,651 (33.0%) previously incompletely predicted profiles (Table 4). Among the collections unenriched for resistance, 3439/3450 (99.7%) profiles were thereby correctly predicted pan-susceptible (S7).

**Figure 1:**
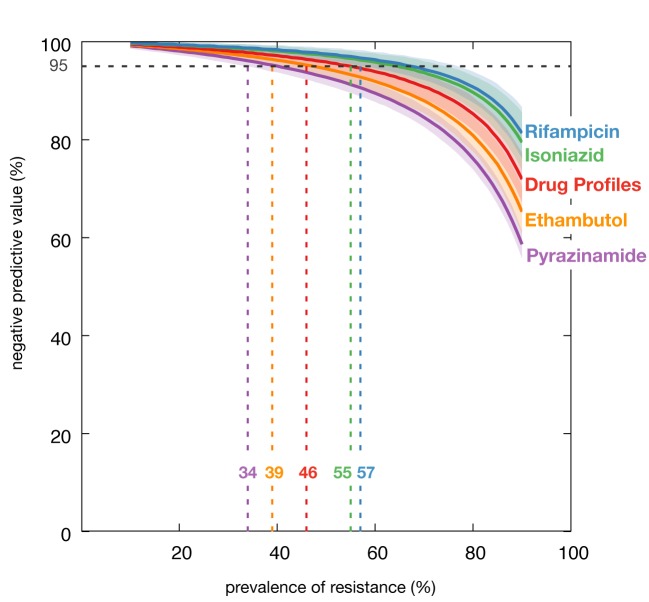
Simulated negative predictive values for individual drugs and complete drug profiles Negative predictive vales shown for individual drugs and complete drug profiles, according to simulated prevalence of resistance to each drug, or within each drug profile (‘any resistance’). For each percentage prevalence between 10% and 90%, 1,000 isolates were randomly selected, 1,000 times. Lines indicate the median with shaded areas showing the 95% confidence intervals.

To simulate how this approach would perform in settings with differing burdens of antimicrobial resistance, we assessed the decline in negative predictive value with increasing prevalence of resistance to individual drugs, and with prevalence of any resistance within drug profiles. We randomly sub-sampled 1,000 isolates to represent every 1% increment in antimicrobial-resistance prevalence between 10%-90%, repeating this 1,000 times for each drug and for complete drug profiles. Negative predictive value declined further for ethambutol and pyrazinamide than for complete drug profiles, but declined least for isoniazid and rifampicin. Below 47.0% prevalence of resistance to any drug, the simulated negative predictive value remained above 95% for 97.5% of drug profiles (Figure 1). 

### Discrepancy analyses

In Australia, eleven ethambutol susceptible isolates containing *embB* mutations were re- phenotyped. Three repeat assays failed, but seven of the remaining eight yielded, now consistent, resistant phenotypes. In Peru, 10 of 16 repeated assays remained phenotypically susceptible by MODS despite *fabG1* C-15T or G-17T mutations. In isolates from the Netherlands, six resistant phenotypes predicted susceptible were identified as clerical errors, and three susceptible phenotypes predicted resistant tested phenotypically resistant by alternative phenotypic assays (S8). Although additional re- phenotyping was not possible, we conducted a ‘per mutation’ analysis to further assess discrepancies. 

Of the 322 resistant phenotypes predicted susceptible, 290 (90.1%) had no mutations affecting targeted genes, and 32 (9.9%) had one or more of 15 mutations per isolate, each previously characterised as consistent with antimicrobial susceptibility. Supporting this, across all isolates in which these 15 mutations occurred as the sole mutation, they correctly predicted isoniazid susceptibility in 286/293 (97.6%) isolates and ethambutol susceptibility in 95/119 (79.8%) isolates. The one mutation relevant to pyrazinamide was seen in two isolates, both of which were phenotypically resistant. None of these mutations were relevant to rifampicin (S9). 

Among 822 susceptible phenotypes predicted resistant, 145 different resistance-conferring mutations were found. Of these, 142 (97.9%) featured as the only resistance-conferring mutation in at least one isolate in the dataset, allowing assessment of individual predictive performance. They correctly predicted resistance to isoniazid in 308/371 (83.0%) isolates, rifampicin in 548/627 (87.4%) isolates, ethambutol in 1280/1743 (73.4%) isolates, and pyrazinamide in 459/663 (69.2%) isolates (S9). 14 of 17 (82.3%) mutations leading to rifampicin resistance predictions in phenotypically susceptible isolates were in the genetic region targeted by Xpert MTB/RIF and MTBDR*plus*.

Laboratory sample mislabelling probably also contributed discrepant results. This was estimated for each collection from the proportion of isolates excluded because of *katG* S315T or *rpoB* S450L mutations and susceptible phenotypes, the collection’s discrepancy rate, and the prevalence of antimicrobial resistance (S10). Overall, about 43% of isoniazid, and 12% of rifampicin discrepancies were thereby attributable to mislabelling. 

## Discussion

This analysis of over 10,000 *M. tuberculosis* isolates collected from 16 countries across six continents, and representing all major lineages, demonstrates that whole-genome sequencing can now characterise susceptible first-line anti-tuberculosis drug profiles sufficiently accurately for clinical use. The importance of this is twofold: First, it demonstrates that the genomic approach can be used to tailor individual treatment regimens. Extended to all drugs, individualised therapies promise to improve cure rates over those achieved by semi-empiric regimens directed by more limited diagnostic tests.1 Second, it is now possible to reduce the phenotypic workload where routine whole-genome sequencing is performed.

The WHO’s target product profiles for new molecular assays for *M. tuberculosis* require over 90% and 95% sensitivity and specificity, respectively.^7^ Overall, both these targets were met for all drugs with the exception of specificity for ethambutol (93.6%). This is no surprise as phenotyping is an imperfect gold standard, in particular for isolates with *embB* mutations.^6,13,16^ For the collections unenriched for resistance, all drugs did however meet these targets, as did the predictions of pan- susceptibility in all collections. Only categorical agreement was assessed for complete drug profile predictions because of the number of permutations. These met the external quality assurance criteria (>80% concordance) for the European TB reference laboratory network.^17^

There are three reasons why pan-susceptibility predictions were particularly accurate. First, the knowledgebase included both resistance-associated genomic mutations, and mutations compatible with phenotypic susceptibility. Second, anti-tuberculosis drug susceptibility phenotypes are not independent of one another, allowing the use of isoniazid susceptibility to predict susceptibility to other drugs. Third, no predictions were attempted for isolates containing genomic variation of unknown association in genes affecting isoniazid. This maximised confidence in isoniazid predictions that were made. Consequently, the prediction of drug profiles performed better than the per-drug analysis for ethambutol and pyrazinamide, and although there was a slight corresponding decline in performance for isoniazid and rifampicin, simulations showed that the prevalence of resistance would have to exceed that seen in most of the worst affected countries in the world before these predictions no longer satisfied the WHO targets.^1^

Our findings showed substantial improvements over the *in-silico* predictions for the sensitivity of WHO-recommended PCR-based assays because whole-genome sequencing is able to identify many more mutations. These additional mutations were however simultaneously responsible for the losses in specificity, largely because of the number of mutations for which a minority of isolates did not manifest a resistant phenotype. A typical example of such is the *rpoB* I491F mutation which frequently gives a susceptible rifampicin result in liquid culture but has been linked to treatment failure.^4,18,19^

The broader discrepancy analysis highlighted the same phenomenon. Whilst the predictive performance of individual mutations, whether probed by WHO-recommended assays or not, was good, each mutation has an error rate, occasionally leading to an unexpected phenotype in a minority of isolates. This is most likely where a mutation elevates the minimum drug concentration required to inhibit bacterial growth to close to the concentration above which an isolate is considered resistant. Canonical ethambutol mutations are a classic example,^20^ but there are many others including the mutations missed by the MODS assay in Peru.^16,21,22^ Such phenomena are thus likely to explain the majority of isolates that were predicted resistant, yet were phenotypically susceptible. They are also the most likely reason why predicting pan-susceptible drug profiles was more accurate than predicting profiles apparently resistant to one or more drugs.

One study limitation is that the scale and cost of repeat sequencing and phenotyping of isolates meant that we could not definitively resolve most discrepancies. This was most concerning for phenotypically resistant isolates predicted susceptible. For these, possible explanations include phenotypic error, resistant minority bacterial populations undetected by sequencing, mechanisms of resistance linked to genes we did not interrogate, or laboratory labelling error.

More work remains to be done before predictions can be extended to second and third-line drugs, and to newer compounds. However, following external review, Public Health England has already decided to stop phenotyping isolates predicted pan-susceptible to first-line drugs (personal communication, Derrick Crook, Director, National Infection Service). Similar moves are expected in the Netherlands (Dick van Soolingen, Rijksinstituut voor Volksgezondheid en Milieu) and New York (Kimberlee Musser, Wadsworth Center, New York State Department of Health). For low and middle- income countries without easy access to phenotyping, there is now the prospect that emerging mobile sequencing platforms could be used to implement sequence-directed therapies, a potential solution to the call for universal susceptibility testing. Portable platform sequencing directly from spiked-samples has been achieved, although real-world systematic evaluation is still required.^23^

Should whole-genome sequencing perform as well for second and third-line drugs as for first- line, a clinical trial could be needed to assess the performance of individualised over standardized treatment regimens in countries with a high drug-resistant disease burden.^24^ Individualised therapies would be expected to reduce the amplification of resistance (to other drugs) in individual patients, side- effects, likelihood of onward transmission, and to exert a weaker selection pressure on strains at a population level, which is key where empiric regimens have been targeted on the basis of very narrow data on antimicrobial susceptibility.^4^ Welcome public health benefits could result from monitoring transmission using the very same sequences.^2^

The current investment in whole-genome sequencing in high-income countries is likely to help accelerate implementation in lower-income, higher-burden countries where the potential benefit is greatest.^25^ These data demonstrate how our understanding of the molecular determinants of resistance to first-line anti-tuberculosis drugs is now sufficiently good to start using DNA sequencing to guide therapy. Similar performance must now be replicated for the remaining drugs.

## Supplementary Appendix

Supplementary Material
