# Supplementary material for: DNA Sequencing Predicts 1st-Line Tuberculosis Drug Susceptibility Profiles
Source: N Engl J Med. Author manuscript; Available in PMC 2018 Sep 26. (PMC6121966; doi:10.1056/NEJMoa1800474)
Supplement: Supplementary Material [file NEJMoa1800474_Walker_SuppApp.pdf]

DNA Sequencing Predicts 1st-Line Tuberculosis Drug Susceptibility Profiles

Supplementary materials

Contents:

Further details on sampling frame (including Table S0), missing data, *in silico* predictions for commercial molecular assays, and mislabeling rates. p.2

S1 summary. p.4
S1 details. p.5
(All isolates, SRA accession numbers, phenotypic methods and results, and genotypic predictions)

S2: p.94
(Knowledgebase of mutations used to make genomic predictions)

S3: p.108
(Performance of predictions by drug, and against different associated drug phenotypes)

S4: p.117
(Performance of predictions for de-duplicated data set, i.e. using only one representative per genomic cluster)

S5: p.118
(Drug profile predictions)

S6: p.119
(Performance for individual drugs for isolates with complete phenotypic data available)

S7: p.120
(Performance of drug profile predictions for isolates from countries' collections that were not enriched for resistance)

S8: p.121
(Re-phenotyping results, and results of clerical cross-checking analysis)

S9: p.122
(Performance of individual mutations that led to discrepancies, across the whole data set)

S10: p.124
(Estimated proportion of isolates discrepant due to mislabelling error)

<u>Further details on sampling frames, missing data, *in* silico predictions for commercial molecular assays, and mislabelling rates</u>

1. Sampling frame:

Isolates were derived from different collections, as detailed in table S1. All were enriched for resistance, with the exception of the collections from Germany (Hamburg), UK (Birmingham, Oxford, Leeds), Netherlands, and Italy (MGIT study).

The unenriched UK isolates were drawn from a prospective population study from Birmingham (2009-13) and from a collection of routinely sequenced clinical isolates from all of Birmingham since 2013. The surrounding region (the ‘Midlands’) has been prospectively sequenced since 2014, and was retrospectively sequenced from 2012-14. All isolates from the Midlands region are referred to the one centralised reference laboratory and stored there. Additional UK isolates were prospectively sequenced in Leeds as part of a study to sequence all positive MGIT cultures coming through Leeds laboratory between Oct 2013 to January 2014. Samples from Oxford were all sequenced as part of a prospective local surveillance effort, as well as part of the same study as in Leeds.

The unenriched Italian isolates were sequenced as part of a different prospective study of all positive MGIT cultures in Florence and surrounding province between February and October 2016. In Florence, all TB samples are referred to this one, centralised laboratory.

Isolates from Germany were all sequenced as part of a population study in Hamburg.

Isolates from the Netherlands were sequenced as part of a prospective population-based study in 2016, with additional samples sequenced from previous years as part of outbreak surveillance work. These latter samples were not enriched for resistance.

Although samples from the Birmingham, Oxford and Hamburg population studies were included in a previous study from which part of the knowledgebase of resistance mutations was derived, as described in the methods section (<u>http://www.thelancet.com/journals/laninf/article/PIIS1473-3099(15)00062-6/abstract</u>), no samples that were included in that 2015 paper were re-used in this study (i.e. there is zero overlap across the studies).

Had any of the components of this subset been inadvertently enriched for resistance, we would expect bias towards worse outcome in terms of the negative predictive value, rather than better outcomes. To be secure that no bias occurred, the subset analysis was re-run using only the truly prospectively sampled isolates (samples from Birmingham, only prospective samples from the Midlands outside of Birmingham, samples from Leeds, from Florence, from Hamburg over the final 3 years of that study, and from the Netherlands from 2016 only). The results are shown in table S0 below and are not materially different from those presented in Table 2c, based on all isolates from ‘collections unenriched for resistance’.

Table S0:

	Resistant phenotype, n (%)					Susceptible phenotype, n (%)								
	R	S	U	F		R	S	U	F		Sensitivity	Specificity	PPV	NPV
INH	269	8	5	4	286	14	2,746	80	59	2,899	97.1	99.5	95.1	99.7
RIF	113	0	0	9	122	27	2,877	85	104	3,093	100.0	99.1	80.7	100.0
EMB	64	1	0	0	65	44	2,716	342	33	3,135	98.5	98.4	59.3	100.0
PZA	92	6	3	6	107	23	2,938	10	51	3,022	93.9	99.2	80.0	99.8

2. Missing phenotypic data

Missing phenotypic data was largely systematic, with different countries and laboratories having different policies on what was routinely tested. Details are shown in table S1. Some within centre variation was seen, which reflects of occasional assay failure or individual clinical decision to only test a subset of drugs – for example in the context of >1 isolate from the same patient having been referred to the laboratory for testing, or one culture becoming contaminated and another clinical isolate being sought. Alternatively, where PZA susceptibility was not routinely tested, clinical grounds occasionally determined that phenotypic PZA assays were performed.

3. *In silico* predictions for the MTB/RIF Xpert and the HAIN MTBDR*plus* and MTBDR*sl* v1.0

All genomic loci probed by these tests were explored in the genome sequence data. Where a nucleotide call other than 'wild type' was identified, a prediction was made that the molecular assay in question would report 'resistance'. This included both synonymous and non-synonymous mutations, and therefore covered both HAIN line-probe eventualities of 'MUT' or 'wild type loss'. No prediction for these assays was made where there was a mixed population in the genomic data as it is not clear how the molecular assays would perform in such circumstances.

4. Mislabelling rates

The estimated mislabelling rate for isoniazid was greater than for rifampicin. The reason for this is not certain, but a significantly higher error rate was seen in mono-resistant isolates than for MDR-TB. For example, for isoniazid susceptible isolates containing rpoB_S450L mutations, 8/347 (2.2%) were phenotypically rifampicin susceptible, whilst for isoniazid resistant isolates containing rpoB_S450L mutations only 11/1527 (0.7%) were phenotypically rifampicin susceptible (p=0.01). Similarly, for katG_S315T mutations, for riampicin susceptible isolates containing katG_S315T mutations, 20/1093 (1.8%) were phenotypically isoniazid susceptible, whereas for riampicin resistant isolates containing katG_S315T mutations, only 13/1501 (0.9%) were phenotypically isoniazid susceptible (p=0.03). It may therefore be that concern about MDR-TB resulted in more careful cross-checking in individual centres, and that this concern was more often present when rpoB mutations had been detected (e.g. by MTB/RIF Xpert pre-testing), leading to a lower error rate than for katG mutations.

Table S1 summary

Source of original samples	Source of data	Country	WGS location	Phenotypic method	Agents routinely tested in this centre (for exceptions, see phenotypic results in Table S1)	Critical concentrations
Birmingham and surrounding Midlands, UK	Public Health England, Birmingham, UK	UK	UK	MGIT 960	HREZ	H 0.1μg/mL; R 1μg/mL; E 5μg/mL; Z 100μg/mL
British Columbia , Canada	British Columbia Centre for Disease Control, Canada	Canada	Canada	MGIT 960	HRE	H 0.1μg/mL; R 1μg/mL; E 5μg/mL; Z - direct detection of pyrazinamidase activity
Samara, Russian Federation	Casali et. al. Nat Genet. 2014 (PMID: 24464101)	Russia	Russia	Resistance ratio method on LJ slopes. Modified Marks biphasic method for pyrazinamide, with confirmation in MGIT 960	HREZ	n/a
Belgium	Genoscreen	Belgium	France	MGIT 960	HREZ	H 0.1μg/mL; R 1.0μg/mL; E 5.0μg/mL; Z 100μg/mL
Hamburg	Forschungszentrum Borstel, Germany	Germany	Germany	MGIT 960	HREZ	H 0.1μg/mL; R 1μg/mL; E 5μg/mL; Z 100μg/mL
Netherlands	Harvard School of Public Health, USA	Netherlands	USA	MGIT 960	HREZ	H 0.1μg/mL; R 1.0μg/mL; E 5.0μg/mL; Z 100μg/mL
Peru	Harvard School of Public Health, USA	Peru	USA	MGIT 960	HREZ	H 0.2μg/mL; R 1.0μg/mL; E 5.0μg/mL; Z 100μg/mL
Italy	San Raffaele Hospital, Milan, Italy	Italy	Italy	MGIT 960	HREZ	H 0.1μg/mL; R 1μg/mL; E 5μg/mL; Z 100μg/mL
Italy, MGIT study in Florence	San Raffaele Hospital, Milan, Italy	Italy	Italy	MGIT 960	HREZ	H 0.1μg/mL; R 1μg/mL; E 5μg/mL; Z 100μg/mL
Leeds	Public Health England and Leeds NHS teaching hospital, UK	UK	UK	MGIT 960	HREZ	H 0.1μg/mL; R 1μg/mL; E 5μg/mL; Z 100μg/mL
London	Public Health England, London and Birmingham, UK	UK	UK	Resistance ratio method on LJ slopes. Modified Marks biphasic method for pyrazinamide, with confirmation in MGIT 960	HREZ	n/a
Swaziland	MSF Swaziland and Forschungszentrum Borstel, Germany	Swaziland	Germany	MGIT 960	HRE	H 0.1μg/mL; R 1μg/mL; E 5μg/mL; Z 100μg/mL
Netherlands	RIVM, Netherlands	Netherlands	Netherlands	MGIT 960	HREZ	H 0.1μg/mL; R 1μg/mL; E 5μg/mL; Z 100μg/mL
Oxford	Oxford University Hospitals NHS Foundation Trust	UK	UK	Resistance ratio method on LJ slopes. Modified Marks biphasic method for pyrazinamide, with confirmation in MGIT 960	HREZ	n/a
Pakistan	National TB Control Programme, Pakistan and San Raffaele Hospital,Milan	Pakistan	Italy	LJ proportion method	HREZ	H 0.2μg/mL; R 40μg/mL; E 2μg/mL; Z 100μg/mL
Lima, Peru	Lima, Peru and London School of Hygiene and Tropical Medicine, UK	Peru	UK	MODS	HR	H 0.4μg/mL; R 1.0μg/mL
Serbia	University of Belgrade, Serbia and Forschungszentrum Borstel, Germany	Serbia	Serbia	MGIT 960	HREZ	H 0.1μg/mL; R 1μg/mL; E 5μg/mL; Z 100μg/mL
South Africa	NICD, Johannesburg, South Africa	South Africa	South Africa	MGIT 960	HREZ	H 0.1μg/mL; R 1μg/mL; E 5μg/mL; Z 100μg/mL
Valencia, Spain	Valencia, Spain	Spain	Spain	MGIT 960	HREZ	H 0.1μg/mL; R 1μg/mL; E 5μg/mL; Z 100μg/mL
Thailand	National University of Singapore	Thailand	Singapore	Middlebrooks 7H10 Agar	HRE	H 0.2μg/mL; R 1.0μg/mL; E 5.0μg/mL
New South Wales, Australia	University of Sydney, Australia	Australia	Australia	MGIT 960	HREZ	H 0.1μg/mL; R 1μg/mL; E 5μg/mL; Z 100μg/mL
Shanghai, China	Yang et. al. Lancet Infect Diseases 2017 (PMID: 27919643)	China	China	MGIT 960	HRE	H 0.1μg/mL; R 1μg/mL; E 5μg/mL
China	Zhang et. al. Nat Genet. 2013 (PMID: 23995137)	China	China	Agar proportion	HRE	H 0.2μg/mL; R 40μg/mL; E 2μg/mL

Table S1, details

						Phenotype				Prediction			
labnumber	source	country	lineage	SRA	Bioproject	Isoniazid	Rifampicin	Ethambutol	Pyrazinamide	Isoniazid	Rifampicin	Ethambutol	Pyrazinamide
S15	University of Sydney	Australia	Beijing	SRR2333215	PRJNA270697	R	R	S	R	R	R	R	R
S43	University of Sydney	Australia	Beijing	SRR2328057	PRJNA270697	R	S	S	R	R	S	S	R
S6	University of Sydney	Australia	lineage4	SRR6369878	PRJNA393378	R	R	S	R	R	S	S	R
S27	University of Sydney	Australia	Beijing	SRR6339651	PRJNA393378	R	R	R	S	R	R	R	S
S3	University of Sydney	Australia	LAM	SRR6369876	PRJNA393378	R	R	S	R	R	R	R	R
S11	University of Sydney	Australia	Beijing	SRR6339662	PRJNA393378	R	R	S	S	R	R	R	R
S37	University of Sydney	Australia	Beijing	SRR6339664	PRJNA393378	R	R	S	S	R	R	S	S
S19	University of Sydney	Australia	LAM	SRR5817471	PRJNA393378	R	R	S	R	R	R	S	S
S18	University of Sydney	Australia	Delhi	SRR5817467	PRJNA393378	R	R	S	S	R	R	S	S
S4	University of Sydney	Australia	Beijing	SRR6369875	PRJNA393378	R	R	S	R	S	F	S	S
S21	University of Sydney	Australia	EAI	SRR6367396	PRJNA393378	R	R	S	S	S	S	S	S
S28	University of Sydney	Australia	lineage4	SRR6339645	PRJNA393378	R	R	S	S	S	R	U	S
S44	University of Sydney	Australia	Beijing	SRR6369877	PRJNA393378	R	S	R	S	R	S	R	S
S2	University of Sydney	Australia	Beijing	SRR5817478	PRJNA393378	R	R	S	R	R	R	R	R
S22	University of Sydney	Australia	Delhi	SRR6339641	PRJNA393378	R	R	S	S	R	R	S	S
S31	University of Sydney	Australia	EAI	SRR6339667	PRJNA393378	R	S	R	S	R	S	R	S
S30	University of Sydney	Australia	Delhi	SRR6339661	PRJNA393378	R	R	S	S	R	R	R	R
S33	University of Sydney	Australia	LAM	SRR5817480	PRJNA393378	R	R	S	S	R	R	R	S
S26	University of Sydney	Australia	Haarlem	SRR6339648	PRJNA393378	R	R	S	S	R	R	S	S
S29	University of Sydney	Australia	EAI	SRR6339653	PRJNA393378	R	R	R	R	R	R	R	R
S13	University of Sydney	Australia	Beijing	SRR6367401	PRJNA393378	R	R	R	R	R	R	R	R
S20	University of Sydney	Australia	Beijing	SRR6339640	PRJNA393378	R	R	S	R	R	R	R	R
S8	University of Sydney	Australia	Beijing	SRR6339666	PRJNA393378	R	R	S	R	R	R	R	R
S25	University of Sydney	Australia	LAM	SRR6339637	PRJNA393378	R	R	S	S	R	R	S	S
S17	University of Sydney	Australia	Beijing	SRR6339643	PRJNA393378	R	R	R	R	R	R	R	R
S39	University of Sydney	Australia	Beijing	SRR5817472	PRJNA393378	R	R	R	R	S	F	F	F
S10	University of Sydney	Australia	Beijing	SRR5817475	PRJNA393378	R	R	S	S	R	R	S	S
S16	University of Sydney	Australia	Beijing	SRR5817469	PRJNA393378	R	R	R	R	R	R	R	R
S14	University of Sydney	Australia	Beijing	SRR5817464	PRJNA393378	R	R	R	R	R	R	R	R
S34	University of Sydney	Australia	Haarlem	SRR5817470	PRJNA393378	R	R	R	R	R	R	R	R
S35	University of Sydney	Australia	Tur	SRR6339649	PRJNA393378	R	R	R	S	R	R	R	S
S23	University of Sydney	Australia	EAI	SRR5817479	PRJNA393378	R	R	S	R	R	R	R	R
S42	University of Sydney	Australia	Beijing	SRR6339665	PRJNA393378	R	S	S	R	R	S	S	R
S38	University of Sydney	Australia	Beijing	SRR5817473	PRJNA393378	R	R	S	R	R	R	U	R
S9	University of Sydney	Australia	EAI	SRR6339644	PRJNA393378	R	R	R	R	R	R	R	R
S1	University of Sydney	Australia	EAI	SRR6367399	PRJNA393378	R	R	R	R	R	R	R	R
S32	University of Sydney	Australia	EAI	SRR5817481	PRJNA393378	R	R	R	S	R	F	R	F
S41	University of Sydney	Australia	Beijing	SRR6367398	PRJNA393378	R	R	S	R	R	R	R	F
S36	University of Sydney	Australia	Beijing	SRR5817466	PRJNA393378	R	R	R	R	R	F	R	R
S12	University of Sydney	Australia	Delhi	SRR6339642	PRJNA393378	R	R	R	R	R	R	R	R
S7	University of Sydney	Australia	Delhi	SRR5817463	PRJNA393378	R	R	S	S	R	R	R	S
S5	University of Sydney	Australia	Beijing	SRR6367400	PRJNA393378	R	R	R	S	R	R	R	S
12MY1752	Genoscreen	Belgium	lineage4			R	R	R	R	R	R	R	R
10MY0917	Genoscreen	Belgium	lineage4			R	R	R	R	R	R	R	U
13MY0792	Genoscreen	Belgium	lineage4			S	S	S	S	S	S	S	S
13MY0573	Genoscreen	Belgium	lineage4			S	S	S	S	S	S	S	S
13MY0452	Genoscreen	Belgium	Haarlem			S	S	S	S	S	S	S	S
13MY0772	Genoscreen	Belgium	LAM			S	S	S	S	S	S	S	S
13MY1139	Genoscreen	Belgium	Delhi			S	S	S	S	S	S	S	S
13MY0669	Genoscreen	Belgium	Haarlem			S	S	S	S	S	S	S	S
13MY1440	Genoscreen	Belgium	S-type			S	S	S	S	S	S	S	S
12MY1792	Genoscreen	Belgium	LAM			R	R	S	R	R	R	R	R
07MY0662	Genoscreen	Belgium	lineage4			R	R	R	n/a	R	R	R	S
10MY1099	Genoscreen	Belgium	Beijing			R	R	R	R	R	R	R	R
13MY1270	Genoscreen	Belgium	lineage4			R	R	S	S	R	R	S	S
13MY1250	Genoscreen	Belgium	Haarlem			S	S	S	S	F	F	S	F
13MY2090	Genoscreen	Belgium	Delhi			S	S	S	S	S	S	S	S
11MY0618	Genoscreen	Belgium	LAM			R	R	R	R	R	R	R	R
08MY1539	Genoscreen	Belgium	Beijing			S	S	S	R	S	S	U	S
08MY0449	Genoscreen	Belgium	Beijing			R	R	R	R	R	R	R	R
13MY1366	Genoscreen	Belgium	S-type			S	S	S	S	S	S	S	S
13MY2169	Genoscreen	Belgium	LAM			S	S	S	S	S	S	S	S
09MY0190	Genoscreen	Belgium	Beijing			R	R	R	S	R	R	R	R
13MY2158	Genoscreen	Belgium	Haarlem			S	S	S	S	S	U	S	S
14MY0713	Genoscreen	Belgium	Beijing			R	R	R	R	R	R	R	S
13MY0539	Genoscreen	Belgium	BCG			S	S	S	R	S	S	U	R
08MY1353	Genoscreen	Belgium	Haarlem			R	R	S	n/a	U	R	S	S
13MY0773	Genoscreen	Belgium	BCG			S	S	S	R	S	S	S	R
15MY0145	Genoscreen	Belgium	Tur			R	R	S	S	R	R	S	R
13MY1143	Genoscreen	Belgium	lineage4			S	S	S	S	S	S	U	S
13MY1533	Genoscreen	Belgium	Beijing			S	S	S	S	S	S	S	S
13MY0570	Genoscreen	Belgium	Uganda			S	S	S	S	S	S	S	S
14MY0112	Genoscreen	Belgium	Beijing			R	R	R	R	R	R	R	R
13MY1206	Genoscreen	Belgium	lineage4			S	S	S	S	U	S	S	S
11MY0284	Genoscreen	Belgium	Beijing			R	R	R	S	R	R	R	S
14MY1450	Genoscreen	Belgium	Haarlem			R	R	R	R	S	R	R	R
13MY0526	Genoscreen	Belgium	S-type			S	S	S	S	S	S	S	S
13MY0757	Genoscreen	Belgium	LAM			S	S	S	S	S	S	U	S
13MY1824	Genoscreen	Belgium	Haarlem			S	S	S	S	S	S	S	S
13MY0319	Genoscreen	Belgium	Beijing			R	R	S	S	R	R	R	R
13MY1058	Genoscreen	Belgium	Haarlem			S	S	S	S	S	S	S	S
13MY1372	Genoscreen	Belgium	S-type			S	S	S	S	S	S	S	S
11MY1855	Genoscreen	Belgium	LAM			R	R	R	R	R	R	R	R
13MY1557	Genoscreen	Belgium	M. bovis			S	S	S	R	S	S	S	R
08MY0205	Genoscreen	Belgium	LAM			R	R	R	S	R	R	R	S
08MY1581	Genoscreen	Belgium	Beijing			R	R	R	R	R	R	R	U
13MY1309	Genoscreen	Belgium	Delhi			S	S	S	S	S	S	S	S
13MY1985	Genoscreen	Belgium	Haarlem			S	S	S	S	S	S	S	S
13MY1307	Genoscreen	Belgium	LAM			S	S	S	S	S	S	S	S
13MY0494	Genoscreen	Belgium	Beijing			R	R	R	R	R	R	R	R
14MY2137	Genoscreen	Belgium	Beijing			R	R	S	R	R	R	F	F
13MY0586	Genoscreen	Belgium	BCG			S	S	S	R	S	S	S	R
13MY1114	Genoscreen	Belgium	lineage4			S	S	S	S	U	S	S	S
08MY0349	Genoscreen	Belgium	Beijing			R	R	R	R	R	R	R	R
13MY1467	Genoscreen	Belgium	S-type			S	S	S	S	S	S	S	S
08MY0385	Genoscreen	Belgium	Beijing			R	R	S	S	U	R	S	S
14MY1571	Genoscreen	Belgium	lineage4			R	R	S	R	R	R	S	R
13MY2082	Genoscreen	Belgium	Haarlem			S	S	S	S	S	U	S	S
13MY2202	Genoscreen	Belgium	Haarlem			S	S	S	S	S	S	S	S
13MY1123	Genoscreen	Belgium	Haarlem			S	S	S	S	S	S	S	S
13MY1199	Genoscreen	Belgium	LAM			S	S	S	S	S	S	S	S
13MY1221	Genoscreen	Belgium	lineage4			S	S	S	S	S	S	S	S
09MY0467	Genoscreen	Belgium	Beijing			R	R	R	R	R	R	R	S
13MY1311	Genoscreen	Belgium	lineage4			S	S	S	S	S	S	S	S
13MY0822	Genoscreen	Belgium	lineage4			S	S	S	S	S	S	S	S
07MY1001	Genoscreen	Belgium	Beijing			R	R	R	n/a	R	R	R	R
13MY1442	Genoscreen	Belgium	Beijing			R	R	R	R	R	R	R	R
13MY2048	Genoscreen	Belgium	Haarlem			S	S	S	S	S	S	S	S
07MY0942	Genoscreen	Belgium	Uganda			R	R	R	n/a	R	R	R	R
13MY0518	Genoscreen	Belgium	Haarlem			S	S	S	S	S	S	S	S
13MY1981	Genoscreen	Belgium	Haarlem			S	S	S	S	S	S	S	S
13MY0965	Genoscreen	Belgium	Haarlem			S	S	S	S	S	S	S	S
13MY1265	Genoscreen	Belgium	Haarlem			S	S	S	S	S	S	S	S
07MY0789	Genoscreen	Belgium	Uganda			R	R	R	n/a	R	R	R	R

13MY1881	Genoscreen	Belgium	Haarlem	S	S	S	S	S	S	S	S
13MY0493	Genoscreen	Belgium	Beijing	R	R	R	R	R	R	R	R
13MY2178	Genoscreen	Belgium	Beijing	S	S	S	S	S	S	S	S
13MY1294	Genoscreen	Belgium	Haarlem	S	S	S	S	S	S	S	S
13MY1242	Genoscreen	Belgium	Cameroon	S	S	S	S	S	S	S	S
14MY0483	Genoscreen	Belgium	Beijing	R	R	S	R	R	R	R	R
13MY0848	Genoscreen	Belgium	LAM	S	S	S	S	S	S	S	S
13MY0161	Genoscreen	Belgium	Beijing	R	R	R	S	R	R	R	S
13MY1660	Genoscreen	Belgium	S-type	S	S	S	S	S	S	S	S
13MY2039	Genoscreen	Belgium	LAM	S	S	S	S	S	S	S	S
13MY1240	Genoscreen	Belgium	lineage4	S	S	S	S	S	S	S	S
07MY0787	Genoscreen	Belgium	Beijing	R	R	R	n/a	R	R	R	R
13MY1516	Genoscreen	Belgium	Haarlem	S	S	S	S	S	S	S	S
09MY0069	Genoscreen	Belgium	lineage4	R	R	R	n/a	R	R	R	U
10MY1172	Genoscreen	Belgium	BCG	R	R	S	R	R	R	S	R
13MY2092	Genoscreen	Belgium	Haarlem	S	S	S	S	S	S	U	S
11MY1029	Genoscreen	Belgium	Beijing	R	R	S	R	R	R	R	R
08MY0089	Genoscreen	Belgium	Ghana	R	R	R	R	R	R	U	U
08MY1089	Genoscreen	Belgium	Beijing	R	R	R	R	R	R	R	R
12MY1616	Genoscreen	Belgium	LAM	R	R	R	S	R	R	R	S
13MY1823	Genoscreen	Belgium	LAM	S	S	S	S	S	S	S	S
13MY0991	Genoscreen	Belgium	Haarlem	S	S	S	S	S	S	U	S
13MY1200	Genoscreen	Belgium	lineage4	S	S	S	S	S	S	S	S
12MY1287	Genoscreen	Belgium	Beijing	R	R	R	R	R	R	R	F
13MY1715	Genoscreen	Belgium	M. bovis	S	S	S	R	S	S	U	R
10MY0992	Genoscreen	Belgium	Beijing	R	R	S	R	R	R	U	R
13MY0655	Genoscreen	Belgium	Beijing	R	R	R	R	R	R	R	R
13MY2152	Genoscreen	Belgium	S-type	S	S	S	S	S	S	U	S
08MY0891	Genoscreen	Belgium	LAM	R	R	R	S	R	R	U	S
13MY1108	Genoscreen	Belgium	Haarlem	S	S	S	S	S	S	S	S
13MY1995	Genoscreen	Belgium	LAM	S	S	S	S	S	S	S	S
13MY1584	Genoscreen	Belgium	Cameroon	S	S	S	S	S	S	U	S
12MY0570	Genoscreen	Belgium	Beijing	R	R	R	R	R	R	R	R
08MY0057	Genoscreen	Belgium	Delhi	R	R	R	S	R	R	R	S
13MY1141	Genoscreen	Belgium	Haarlem	S	S	S	S	S	S	U	S
13MY0952	Genoscreen	Belgium	EAI	S	S	S	R	U	S	S	S
12MY0258	Genoscreen	Belgium	Tur	R	R	S	S	R	R	S	S
13MY1045	Genoscreen	Belgium	Haarlem	S	S	S	S	S	S	S	S
13MY0758	Genoscreen	Belgium	lineage4	S	S	S	S	S	S	S	S
13MY0512	Genoscreen	Belgium	Haarlem	S	S	S	S	S	S	S	S
13MY1023	Genoscreen	Belgium	LAM	S	S	S	S	S	S	S	S
13MY0618	Genoscreen	Belgium	Haarlem	S	S	S	S	S	S	S	S
09MY0471	Genoscreen	Belgium	EAI	R	R	R	n/a	R	R	R	S
13MY0048	Genoscreen	Belgium	lineage4	R	R	R	R	R	R	R	U
08MY1454	Genoscreen	Belgium	lineage4	R	R	R	n/a	R	R	R	U
13MY0462	Genoscreen	Belgium	LAM	S	S	S	S	S	S	S	U
13MY0715	Genoscreen	Belgium	LAM	S	S	S	S	S	S	U	S
09MY0391	Genoscreen	Belgium	Beijing	R	R	R	R	R	R	R	R
12MY1761	Genoscreen	Belgium	LAM	R	R	R	S	R	R	R	R
12MY0093	Genoscreen	Belgium	LAM	R	S	R	R	R	R	R	R
13MY2312	Genoscreen	Belgium	Beijing	R	R	R	R	R	R	R	R
11MY1719	Genoscreen	Belgium	Cameroon	R	R	S	R	R	R	R	U
13MY0681	Genoscreen	Belgium	Beijing	R	S	S	S	R	S	S	S
07MY0402	Genoscreen	Belgium	Beijing	R	R	R	R	R	R	R	R
13MY0531	Genoscreen	Belgium	LAM	S	S	S	S	S	S	S	S
13MY1437	Genoscreen	Belgium	Beijing	S	S	S	S	S	S	S	S
13MY1111	Genoscreen	Belgium	X-type	S	S	S	S	S	S	S	S
12MY0988	Genoscreen	Belgium	Beijing	R	R	S	S	R	R	R	S
13MY2069	Genoscreen	Belgium	S-type	S	S	S	S	S	S	S	S
08MY1150	Genoscreen	Belgium	Beijing	R	R	R	n/a	R	R	R	R
13MY1947	Genoscreen	Belgium	lineage4	S	S	S	S	S	S	S	S
13MY1132	Genoscreen	Belgium	LAM	S	S	S	S	S	S	U	S
13MY1561	Genoscreen	Belgium	Haarlem	S	S	S	S	S	S	S	S
11MY0673	Genoscreen	Belgium	LAM	R	R	S	S	R	R	S	S
11MY1724	Genoscreen	Belgium	LAM	R	R	R	R	R	R	R	R
11MY0210	Genoscreen	Belgium	Haarlem	R	R	S	S	R	R	S	S
13MY2044	Genoscreen	Belgium	LAM	S	S	S	S	S	S	S	S
13MY1412	Genoscreen	Belgium	Haarlem	S	S	S	S	U	S	S	S
13MY1427	Genoscreen	Belgium	Delhi	S	S	S	S	S	S	S	S
13MY1662	Genoscreen	Belgium	BCG	S	S	S	R	S	S	S	R
13MY0049	Genoscreen	Belgium	Beijing	R	R	R	R	R	R	R	R
13MY2019	Genoscreen	Belgium	LAM	S	S	S	S	S	S	S	S
13MY0694	Genoscreen	Belgium	West Africar	S	S	S	S	F	F	U	F
13MY2136	Genoscreen	Belgium	BCG	S	S	S	R	S	S	S	R
13MY1099	Genoscreen	Belgium	LAM	S	S	S	S	U	S	S	S
131686	Genoscreen	Belgium	LAM	R	R	S	R	R	R	R	R
13MY0739	Genoscreen	Belgium	lineage4	S	S	S	S	S	S	S	S
13MY0861	Genoscreen	Belgium	lineage4	S	S	S	S	S	S	S	S
13MY1574	Genoscreen	Belgium	West Africar	R	R	S	R	R	R	R	R
13MY2005	Genoscreen	Belgium	Haarlem	S	S	S	S	S	S	S	S
13MY1645	Genoscreen	Belgium	lineage4	S	S	S	S	S	U	S	S
13MY1369	Genoscreen	Belgium	S-type	S	S	S	S	S	S	S	S
07MY0775	Genoscreen	Belgium	Beijing	R	R	R	n/a	R	R	R	S
13MY0442	Genoscreen	Belgium	S-type	S	S	S	S	S	U	U	S
13MY1519	Genoscreen	Belgium	S-type	S	S	S	S	S	S	S	S
13MY1013	Genoscreen	Belgium	Haarlem	S	S	S	S	S	S	U	S
13MY1804	Genoscreen	Belgium	Delhi	S	S	S	S	S	S	S	S
07MY1196	Genoscreen	Belgium	Delhi	R	R	S	S	R	R	S	S
13MY0577	Genoscreen	Belgium	lineage4	S	S	S	S	S	S	S	S
07MY1166	Genoscreen	Belgium	Beijing	R	R	R	S	R	R	R	S
13MY1067	Genoscreen	Belgium	lineage4	S	S	S	S	S	S	S	S
08MY0559	Genoscreen	Belgium	LAM	R	R	R	R	R	R	R	R
07MY1253	Genoscreen	Belgium	Beijing	R	R	R	S	R	R	R	S
13MY2046	Genoscreen	Belgium	S-type	S	S	S	S	S	S	S	S
13MY1184	Genoscreen	Belgium	S-type	S	S	S	S	S	S	S	S
11MY1044	Genoscreen	Belgium	Uganda	R	R	R	R	R	R	S	R
13MY0448	Genoscreen	Belgium	lineage4	S	S	S	S	S	S	S	S
13MY1780	Genoscreen	Belgium	M. bovis	S	S	S	R	S	S	S	R
13MY0200	Genoscreen	Belgium	Beijing	R	R	R	R	R	R	R	R
13MY0515	Genoscreen	Belgium	lineage4	S	S	S	S	S	S	S	S
13MY2225	Genoscreen	Belgium	West Africar	R	R	S	R	R	R	R	R
13MY0816	Genoscreen	Belgium	LAM	S	S	S	R	S	S	S	S
13MY1808	Genoscreen	Belgium	lineage4	R	R	R	S	R	R	R	U
13MY1638	Genoscreen	Belgium	Haarlem	S	S	S	S	S	S	S	S
13MY0736	Genoscreen	Belgium	lineage4	S	S	S	S	S	S	S	S
121341	Genoscreen	Belgium	Beijing	R	R	R	R	R	R	R	R
07MY0277	Genoscreen	Belgium	Ghana	R	R	R	n/a	R	R	U	U
13MY0159	Genoscreen	Belgium	Beijing	R	R	S	R	R	R	R	R
13MY0653	Genoscreen	Belgium	S-type	S	S	S	S	S	S	S	S
12MY1475	Genoscreen	Belgium	Ural	R	R	S	R	R	R	S	R
13MY1213	Genoscreen	Belgium	lineage4	S	S	S	S	S	S	S	S
12MY1730	Genoscreen	Belgium	Beijing	R	R	R	R	R	R	R	F
13MY0829	Genoscreen	Belgium	Haarlem	S	S	S	S	S	S	S	S
13MY0292	Genoscreen	Belgium	LAM	R	R	S	S	R	R	S	S
11MY1339	Genoscreen	Belgium	Beijing	R	R	R	R	R	R	R	R
13MY1582	Genoscreen	Belgium	Haarlem	S	S	S	S	S	S	S	S

13MY1937	Genoscreen	Belgium	LAM		S	S	S	S	S	S	S	S
13MY0475	Genoscreen	Belgium	Haarlem		S	S	S	S	S	S	S	S
09MY1304	Genoscreen	Belgium	Beijing		R	R	S	S	R	R	U	S
12MY0223	Genoscreen	Belgium	lineage4		R	R	S	S	R	R	S	S
13MY1983	Genoscreen	Belgium	lineage4		S	S	S	S	S	S	S	S
07MY0788	Genoscreen	Belgium	Beijing		R	R	R	n/a	R	R	R	R
13MY1065	Genoscreen	Belgium	Beijing		S	S	S	S	U	S	S	S
11MY0630	Genoscreen	Belgium	Delhi		R	R	S	R	R	R	R	R
13MY1323	Genoscreen	Belgium	S-type		S	S	S	S	S	S	S	S
13MY0578	Genoscreen	Belgium	lineage4		S	S	S	S	S	S	S	S
12MY1157	Genoscreen	Belgium	Beijing		R	R	R	R	R	R	R	R
12MY1487	Genoscreen	Belgium	Beijing		R	R	S	R	U	R	S	R
13MY0866	Genoscreen	Belgium	LAM		S	S	S	S	S	S	S	S
09MY0426	Genoscreen	Belgium	Beijing		R	R	R	R	R	R	R	U
13MY2189	Genoscreen	Belgium	lineage4		S	S	S	S	S	U	S	S
13MY1127	Genoscreen	Belgium	Haarlem		S	S	S	S	S	S	S	S
13MY1163	Genoscreen	Belgium	lineage4		S	S	S	S	S	S	S	U
13MY1581	Genoscreen	Belgium	Cameroon		S	S	S	S	S	S	S	S
13MY1003	Genoscreen	Belgium	lineage4		S	S	S	S	S	S	S	S
11MY0214	Genoscreen	Belgium	Beijing		R	R	R	R	R	R	R	R
13MY1864	Genoscreen	Belgium	lineage4		S	S	S	S	S	S	S	S
11MY1117	Genoscreen	Belgium	LAM		R	R	R	S	R	R	U	S
13MY1095	Genoscreen	Belgium	BCG		S	S	S	R	S	S	S	R
10MY1264	Genoscreen	Belgium	Beijing		R	R	S	S	R	R	S	S
13MY1825	Genoscreen	Belgium	LAM		S	S	S	S	S	S	S	S
13MY0969	Genoscreen	Belgium	Ural		S	S	S	S	S	S	S	S
13MY1216	Genoscreen	Belgium	Ghana		S	S	S	S	S	S	S	S
13MY1867	Genoscreen	Belgium	LAM		S	S	S	S	S	S	U	S
11MY0596	Genoscreen	Belgium	Beijing		R	R	R	R	R	R	R	R
13MY1106	Genoscreen	Belgium	EAI		S	S	S	S	S	S	S	S
13MY1164	Genoscreen	Belgium	West Africar		S	S	S	S	S	U	U	S
13MY0803	Genoscreen	Belgium	Haarlem		S	S	S	S	S	S	U	S
13MY0702	Genoscreen	Belgium	Haarlem		S	S	S	S	S	S	S	S
07MY1281	Genoscreen	Belgium	Beijing		R	R	R	R	R	R	R	R
13MY1832	Genoscreen	Belgium	lineage4		S	S	S	S	S	S	S	S
15MY0479	Genoscreen	Belgium	Ural		R	R	S	S	R	R	U	S
07MY0066	Genoscreen	Belgium	Beijing		R	R	R	n/a	R	R	R	R
08MY1602	Genoscreen	Belgium	LAM		R	R	R	R	R	R	R	R
12MY1124	Genoscreen	Belgium	Delhi		R	R	S	S	R	R	S	S
13MY0537	Genoscreen	Belgium	LAM		S	S	S	S	S	S	S	S
13MY0802	Genoscreen	Belgium	Haarlem		S	S	S	R	S	S	S	S
13MY2167	Genoscreen	Belgium	S-type		S	S	S	S	S	S	S	S
14MY1492	Genoscreen	Belgium	Beijing		R	R	R	R	R	R	R	R
14MY1932	Genoscreen	Belgium	Haarlem		R	R	S	R	R	R	R	U
13MY0734	Genoscreen	Belgium	EAI		S	S	S	R	S	S	S	S
08MY1520	Genoscreen	Belgium	Beijing		R	R	R	R	R	R	R	R
14s464	British Columbia Centre for Disease Control	Canada	EAI	PRJNA413593	S	S	S	n/a	S	S	S	S
10s433	British Columbia Centre for Disease Control	Canada	EAI	PRJNA413593	S	S	S	n/a	S	S	S	S
A9s165	British Columbia Centre for Disease Control	Canada	Beijing	PRJNA413593	R	S	S	n/a	R	S	S	S
10s098	British Columbia Centre for Disease Control	Canada	EAI	PRJNA413593	S	S	S	n/a	S	S	S	S
13s341	British Columbia Centre for Disease Control	Canada	S-type	PRJNA413593	S	S	S	n/a	S	S	S	S
14s364	British Columbia Centre for Disease Control	Canada	Delhi	PRJNA413593	R	S	S	S	R	S	S	S
A9s053	British Columbia Centre for Disease Control	Canada	EAI	PRJNA413593	S	S	S	n/a	S	S	S	S
11s419	British Columbia Centre for Disease Control	Canada	Beijing	PRJNA413593	R	R	S	R	R	R	S	R
11s350	British Columbia Centre for Disease Control	Canada	Ural	PRJNA413593	S	S	S	n/a	S	S	S	S
A6s148	British Columbia Centre for Disease Control	Canada	Beijing	PRJNA413593	S	S	S	n/a	S	S	S	S
13s039	British Columbia Centre for Disease Control	Canada	lineage4	PRJNA413593	S	S	S	n/a	S	S	U	S
A4s024	British Columbia Centre for Disease Control	Canada	LAM	PRJNA413593	S	S	S	n/a	S	S	S	S
A8s260	British Columbia Centre for Disease Control	Canada	Beijing	PRJNA413593	R	S	S	n/a	R	S	S	S
A6s372	British Columbia Centre for Disease Control	Canada	S-type	PRJNA413593	S	S	S	n/a	S	U	S	S
13s350	British Columbia Centre for Disease Control	Canada	EAI	PRJNA413593	S	S	S	n/a	S	S	S	S
A7s071	British Columbia Centre for Disease Control	Canada	lineage4	PRJNA413593	S	S	S	n/a	S	S	S	S
10s195	British Columbia Centre for Disease Control	Canada	Beijing	PRJNA413593	S	S	S	n/a	S	S	S	S
A7s217	British Columbia Centre for Disease Control	Canada	Beijing	PRJNA413593	S	S	S	n/a	S	S	S	S
A8s145	British Columbia Centre for Disease Control	Canada	Delhi	PRJNA413593	S	S	S	n/a	S	S	S	S
A6s145	British Columbia Centre for Disease Control	Canada	lineage4	PRJNA413593	S	S	S	n/a	S	S	S	S
14s063	British Columbia Centre for Disease Control	Canada	EAI	PRJNA413593	S	S	S	n/a	S	S	S	S
10s354	British Columbia Centre for Disease Control	Canada	EAI	PRJNA413593	S	S	S	n/a	S	S	S	S
11s045	British Columbia Centre for Disease Control	Canada	S-type	PRJNA413593	S	S	S	n/a	S	S	S	S
A5s023	British Columbia Centre for Disease Control	Canada	lineage4	PRJNA413593	R	S	S	S	R	S	S	S
12s051	British Columbia Centre for Disease Control	Canada	LAM	PRJNA413593	S	S	S	n/a	S	S	S	S
A6s400	British Columbia Centre for Disease Control	Canada	EAI	PRJNA413593	S	S	S	n/a	S	S	S	S
A5s137	British Columbia Centre for Disease Control	Canada	S-type	PRJNA413593	S	S	S	n/a	S	S	S	S
13s450	British Columbia Centre for Disease Control	Canada	Delhi	PRJNA413593	S	S	S	n/a	S	S	S	S
A8s057	British Columbia Centre for Disease Control	Canada	Ural	PRJNA413593	S	S	S	n/a	S	S	S	S
11s234	British Columbia Centre for Disease Control	Canada	Beijing	PRJNA413593	R	S	S	S	R	S	S	S
A8s259	British Columbia Centre for Disease Control	Canada	Haarlem	PRJNA413593	S	S	S	n/a	S	S	S	S
14s316	British Columbia Centre for Disease Control	Canada	EAI	PRJNA413593	R	S	S	S	R	S	S	S
12s384	British Columbia Centre for Disease Control	Canada	lineage4	PRJNA413593	S	S	S	n/a	S	S	S	S
A7s235	British Columbia Centre for Disease Control	Canada	Beijing	PRJNA413593	R	R	R	R	R	R	R	R
A6s187	British Columbia Centre for Disease Control	Canada	Haarlem	PRJNA413593	S	S	S	n/a	S	S	S	S
13s446	British Columbia Centre for Disease Control	Canada	Delhi	PRJNA413593	S	S	S	n/a	U	S	S	S
11s386	British Columbia Centre for Disease Control	Canada	LAM	PRJNA413593	S	S	S	n/a	S	S	S	S
A6s025	British Columbia Centre for Disease Control	Canada	Beijing	PRJNA413593	S	S	S	n/a	S	U	S	S
A5s298	British Columbia Centre for Disease Control	Canada	EAI	PRJNA413593	S	S	S	n/a	S	S	U	S
A5s070	British Columbia Centre for Disease Control	Canada	S-type	PRJNA413593	S	S	S	n/a	S	U	S	S
A5s043	British Columbia Centre for Disease Control	Canada	Delhi	PRJNA413593	R	S	S	S	R	S	S	S
A2s309	British Columbia Centre for Disease Control	Canada	Delhi	PRJNA413593	S	S	S	n/a	S	S	S	S
14s308	British Columbia Centre for Disease Control	Canada	Beijing	PRJNA413593	R	S	S	S	U	S	U	S
10s171	British Columbia Centre for Disease Control	Canada	lineage4	PRJNA413593	S	S	S	n/a	S	S	S	S
A5s172	British Columbia Centre for Disease Control	Canada	Beijing	PRJNA413593	S	S	S	n/a	U	S	S	S
A9s079	British Columbia Centre for Disease Control	Canada	LAM	PRJNA413593	R	S	R	n/a	R	S	R	S
12s025	British Columbia Centre for Disease Control	Canada	lineage4	PRJNA413593	R	S	S	S	R	S	S	S
A8s323	British Columbia Centre for Disease Control	Canada	Delhi	PRJNA413593	R	R	R	R	R	R	R	R
11s357	British Columbia Centre for Disease Control	Canada	EAI	PRJNA413593	R	S	S	S	R	S	S	S
A7s265	British Columbia Centre for Disease Control	Canada	EAI	PRJNA413593	n/a	S	S	S	S	S	S	S
A8s083	British Columbia Centre for Disease Control	Canada	lineage4	PRJNA413593	S	S	S	n/a	S	S	S	S
12s343	British Columbia Centre for Disease Control	Canada	S-type	PRJNA413593	S	S	S	n/a	S	U	S	S
A5s131	British Columbia Centre for Disease Control	Canada	Beijing	PRJNA413593	S	S	S	n/a	S	S	S	S
12s182	British Columbia Centre for Disease Control	Canada	Tur	PRJNA413593	R	S	S	S	R	S	R	S
10s312	British Columbia Centre for Disease Control	Canada	Ural	PRJNA413593	S	S	S	n/a	S	S	S	S
10s348	British Columbia Centre for Disease Control	Canada	Beijing	PRJNA413593	S	S	S	n/a	S	S	S	S
11s159	British Columbia Centre for Disease Control	Canada	LAM	PRJNA413593	S	S	S	n/a	S	S	U	S
A9s029	British Columbia Centre for Disease Control	Canada	S-type	PRJNA413593	S	S	S	n/a	U	S	S	S
10s020	British Columbia Centre for Disease Control	Canada	Beijing	PRJNA413593	S	S	S	n/a	S	S	S	S
A7s267	British Columbia Centre for Disease Control	Canada	EAI	PRJNA413593	S	S	S	n/a	S	S	S	S
10s291	British Columbia Centre for Disease Control	Canada	Ural	PRJNA413593	S	S	S	n/a	S	S	S	S
13s095	British Columbia Centre for Disease Control	Canada	Haarlem	PRJNA413593	S	S	S	n/a	S	S	S	S
11s411	British Columbia Centre for Disease Control	Canada	Beijing	PRJNA413593	S	S	S	n/a	S	S	S	S
14s482	British Columbia Centre for Disease Control	Canada	Ural	PRJNA413593	S	S	S	n/a	S	S	S	S
13s320	British Columbia Centre for Disease Control	Canada	Beijing	PRJNA413593	S	S	S	n/a	S	S	S	S
A8s004	British Columbia Centre for Disease Control	Canada	EAI	PRJNA413593	S	S	S	n/a	S	S	S	S
A7s159	British Columbia Centre for Disease Control	Canada	Ural	PRJNA413593	S	S	S	n/a	S	S	S	S
A9s356	British Columbia Centre for Disease Control	Canada	Delhi	PRJNA413593	S	S	S	n/a	S	S	S	S
A8s220	British Columbia Centre for Disease Control	Canada	Haarlem	PRJNA413593	S	S	S	n/a	S	S	S	S
10s205	British Columbia Centre for Disease Control	Canada	Beijing	PRJNA413593	S	S	S	n/a	S	S	S	S

A7s068	British Columbia Centre for Disease Control	Canada	Ural	PRJNA413593	S	S	S	n/a	S	S	S	S
A7s066	British Columbia Centre for Disease Control	Canada	lineage4	PRJNA413593	S	S	S	n/a	S	S	S	S
A7s041	British Columbia Centre for Disease Control	Canada	Beijing	PRJNA413593	S	S	S	n/a	S	S	S	S
A5s203	British Columbia Centre for Disease Control	Canada	LAM	PRJNA413593	R	S	S	S	R	S	S	S
A8s290	British Columbia Centre for Disease Control	Canada	Ural	PRJNA413593	S	S	S	n/a	S	S	S	S
A8s136	British Columbia Centre for Disease Control	Canada	Haarlem	PRJNA413593	S	S	S	n/a	S	S	S	S
A8s191	British Columbia Centre for Disease Control	Canada	Delhi	PRJNA413593	S	S	S	n/a	U	S	S	S
A1s040	British Columbia Centre for Disease Control	Canada	EAI	PRJNA413593	S	S	S	n/a	S	S	S	S
A5s059	British Columbia Centre for Disease Control	Canada	EAI	PRJNA413593	S	S	S	n/a	S	S	S	S
13s289	British Columbia Centre for Disease Control	Canada	Beijing	PRJNA413593	S	S	S	n/a	S	S	U	S
12s376	British Columbia Centre for Disease Control	Canada	Haarlem	PRJNA413593	S	S	S	n/a	S	S	S	S
11s136	British Columbia Centre for Disease Control	Canada	Beijing	PRJNA413593	S	S	S	n/a	S	S	S	S
A6s081	British Columbia Centre for Disease Control	Canada	Beijing	PRJNA413593	R	R	R	R	R	R	R	U
11s128	British Columbia Centre for Disease Control	Canada	Ural	PRJNA413593	S	S	S	n/a	S	S	S	S
13s344	British Columbia Centre for Disease Control	Canada	Delhi	PRJNA413593	S	S	S	n/a	U	S	S	S
A9s395	British Columbia Centre for Disease Control	Canada	lineage4	PRJNA413593	R	S	S	S	R	S	S	S
10s050	British Columbia Centre for Disease Control	Canada	Beijing	PRJNA413593	S	S	S	n/a	S	S	S	S
A5s245	British Columbia Centre for Disease Control	Canada	Beijing	PRJNA413593	S	S	S	n/a	S	S	S	S
A9s001	British Columbia Centre for Disease Control	Canada	Delhi	PRJNA413593	S	S	S	n/a	S	S	S	S
A9s103	British Columbia Centre for Disease Control	Canada	S-type	PRJNA413593	S	S	S	n/a	S	U	S	S
13s194	British Columbia Centre for Disease Control	Canada	lineage4	PRJNA413593	S	S	S	n/a	S	S	S	S
A9s120	British Columbia Centre for Disease Control	Canada	S-type	PRJNA413593	S	S	S	n/a	S	U	S	S
A8s058	British Columbia Centre for Disease Control	Canada	Haarlem	PRJNA413593	S	S	S	n/a	S	S	S	S
A9s211	British Columbia Centre for Disease Control	Canada	Beijing	PRJNA413593	R	S	S	n/a	R	S	S	S
A5s199	British Columbia Centre for Disease Control	Canada	Delhi	PRJNA413593	S	S	S	n/a	S	S	U	S
11s108	British Columbia Centre for Disease Control	Canada	Beijing	PRJNA413593	S	S	S	n/a	S	S	S	S
A6s225	British Columbia Centre for Disease Control	Canada	Delhi	PRJNA413593	S	S	S	n/a	S	R	S	S
A9s081	British Columbia Centre for Disease Control	Canada	lineage4	PRJNA413593	S	S	S	n/a	S	U	U	S
A4s089	British Columbia Centre for Disease Control	Canada	Beijing	PRJNA413593	S	S	S	n/a	S	S	S	S
10s199	British Columbia Centre for Disease Control	Canada	lineage4	PRJNA413593	S	S	S	n/a	R	S	S	S
A8s268	British Columbia Centre for Disease Control	Canada	Delhi	PRJNA413593	S	S	S	n/a	S	S	S	S
A6s257	British Columbia Centre for Disease Control	Canada	Ural	PRJNA413593	S	S	S	n/a	S	S	S	S
12s033	British Columbia Centre for Disease Control	Canada	EAI	PRJNA413593	S	S	S	n/a	S	S	S	S
10s292	British Columbia Centre for Disease Control	Canada	S-type	PRJNA413593	S	S	S	n/a	S	U	S	S
14s263	British Columbia Centre for Disease Control	Canada	Beijing	PRJNA413593	R	R	S	S	R	R	S	S
11s232	British Columbia Centre for Disease Control	Canada	Delhi	PRJNA413593	R	S	S	S	R	R	S	S
13s316	British Columbia Centre for Disease Control	Canada	EAI	PRJNA413593	S	S	S	n/a	S	S	S	S
14s144	British Columbia Centre for Disease Control	Canada	Beijing	PRJNA413593	S	S	S	n/a	S	S	S	S
11s389	British Columbia Centre for Disease Control	Canada	Ural	PRJNA413593	S	S	S	n/a	S	S	S	S
A6s330	British Columbia Centre for Disease Control	Canada	EAI	PRJNA413593	S	S	S	n/a	S	S	S	S
12s027	British Columbia Centre for Disease Control	Canada	Delhi	PRJNA413593	S	S	S	n/a	S	S	S	S
12s060	British Columbia Centre for Disease Control	Canada	Beijing	PRJNA413593	S	S	S	n/a	S	S	S	S
A9s381	British Columbia Centre for Disease Control	Canada	Beijing	PRJNA413593	S	S	S	n/a	S	S	S	S
A9s242	British Columbia Centre for Disease Control	Canada	Haarlem	PRJNA413593	S	S	S	n/a	S	S	S	S
A5s027	British Columbia Centre for Disease Control	Canada	S-type	PRJNA413593	S	S	S	n/a	S	S	S	S
12s361	British Columbia Centre for Disease Control	Canada	Beijing	PRJNA413593	R	S	S	S	R	S	S	S
12s319	British Columbia Centre for Disease Control	Canada	Ural	PRJNA413593	S	S	S	n/a	S	S	S	S
A5s259	British Columbia Centre for Disease Control	Canada	lineage4	PRJNA413593	S	S	S	n/a	S	S	S	S
14s270	British Columbia Centre for Disease Control	Canada	lineage4	PRJNA413593	S	S	S	n/a	S	S	U	S
A5s128	British Columbia Centre for Disease Control	Canada	S-type	PRJNA413593	S	S	S	n/a	S	S	S	S
A5s287	British Columbia Centre for Disease Control	Canada	S-type	PRJNA413593	S	S	S	n/a	S	S	S	S
A8s090	British Columbia Centre for Disease Control	Canada	Haarlem	PRJNA413593	S	S	S	n/a	S	S	S	S
A5s286	British Columbia Centre for Disease Control	Canada	Ural	PRJNA413593	S	S	S	n/a	S	S	S	S
A8s357	British Columbia Centre for Disease Control	Canada	S-type	PRJNA413593	S	S	S	n/a	S	S	S	S
A9s033	British Columbia Centre for Disease Control	Canada	Beijing	PRJNA413593	S	S	S	n/a	S	S	U	S
A6s226	British Columbia Centre for Disease Control	Canada	Beijing	PRJNA413593	R	S	S	n/a	R	S	S	S
A5s218	British Columbia Centre for Disease Control	Canada	Haarlem	PRJNA413593	S	S	S	n/a	S	S	S	S
10s052	British Columbia Centre for Disease Control	Canada	Beijing	PRJNA413593	S	S	S	n/a	S	S	S	S
13s286	British Columbia Centre for Disease Control	Canada	Beijing	PRJNA413593	S	S	S	n/a	S	S	S	S
A8s195	British Columbia Centre for Disease Control	Canada	S-type	PRJNA413593	S	S	S	n/a	S	S	U	S
A9s250	British Columbia Centre for Disease Control	Canada	Haarlem	PRJNA413593	S	S	S	n/a	S	S	S	S
A8s007	British Columbia Centre for Disease Control	Canada	EAI	PRJNA413593	S	S	S	n/a	S	S	S	S
A0s004	British Columbia Centre for Disease Control	Canada	S-type	PRJNA413593	S	S	S	n/a	S	U	S	S
A9s122	British Columbia Centre for Disease Control	Canada	Ural	PRJNA413593	S	S	S	n/a	S	S	S	S
12s402	British Columbia Centre for Disease Control	Canada	Haarlem	PRJNA413593	S	S	S	n/a	S	S	S	S
10s304	British Columbia Centre for Disease Control	Canada	lineage4	PRJNA413593	S	S	S	n/a	S	S	S	S
12s199	British Columbia Centre for Disease Control	Canada	Ural	PRJNA413593	S	S	S	n/a	S	S	S	S
A9s124	British Columbia Centre for Disease Control	Canada	Delhi	PRJNA413593	S	S	S	n/a	S	S	S	S
A8s221	British Columbia Centre for Disease Control	Canada	S-type	PRJNA413593	S	S	S	n/a	S	U	U	S
A7s309	British Columbia Centre for Disease Control	Canada	Ural	PRJNA413593	S	S	S	n/a	S	S	S	S
12s108	British Columbia Centre for Disease Control	Canada	Delhi	PRJNA413593	S	S	S	n/a	U	S	S	S
12s207	British Columbia Centre for Disease Control	Canada	LAM	PRJNA413593	S	S	S	n/a	S	S	S	S
A8s315	British Columbia Centre for Disease Control	Canada	S-type	PRJNA413593	S	S	S	n/a	S	S	S	S
13s189	British Columbia Centre for Disease Control	Canada	Beijing	PRJNA413593	S	S	S	n/a	S	S	S	S
A6s443	British Columbia Centre for Disease Control	Canada	S-type	PRJNA413593	S	S	S	n/a	S	U	S	S
11s462	British Columbia Centre for Disease Control	Canada	Beijing	PRJNA413593	S	S	S	n/a	S	S	U	S
11s322	British Columbia Centre for Disease Control	Canada	Beijing	PRJNA413593	S	S	S	n/a	S	S	S	S
14s363	British Columbia Centre for Disease Control	Canada	Beijing	PRJNA413593	S	S	S	n/a	S	S	S	S
11s247	British Columbia Centre for Disease Control	Canada	lineage4	PRJNA413593	S	S	S	n/a	S	S	U	S
A7s029	British Columbia Centre for Disease Control	Canada	lineage4	PRJNA413593	S	S	S	n/a	R	S	S	S
14s240	British Columbia Centre for Disease Control	Canada	EAI	PRJNA413593	R	S	S	S	R	S	S	S
A8s301	British Columbia Centre for Disease Control	Canada	Ural	PRJNA413593	S	S	S	n/a	S	S	S	S
10s269	British Columbia Centre for Disease Control	Canada	EAI	PRJNA413593	S	S	S	n/a	S	S	S	S
12s462	British Columbia Centre for Disease Control	Canada	EAI	PRJNA413593	S	S	S	n/a	S	S	S	S
A7s028	British Columbia Centre for Disease Control	Canada	Beijing	PRJNA413593	R	R	R	R	R	R	R	U
A9s057	British Columbia Centre for Disease Control	Canada	Beijing	PRJNA413593	S	S	S	n/a	S	S	S	S
A7s218	British Columbia Centre for Disease Control	Canada	Haarlem	PRJNA413593	S	S	S	n/a	S	S	S	S
11s363	British Columbia Centre for Disease Control	Canada	EAI	PRJNA413593	S	S	S	n/a	S	S	S	S
13s339	British Columbia Centre for Disease Control	Canada	EAI	PRJNA413593	R	S	S	S	R	S	S	S
A9s382	British Columbia Centre for Disease Control	Canada	Beijing	PRJNA413593	S	S	S	n/a	S	S	S	S
A2s300	British Columbia Centre for Disease Control	Canada	Delhi	PRJNA413593	S	S	S	n/a	S	S	S	S
A8s036	British Columbia Centre for Disease Control	Canada	S-type	PRJNA413593	R	S	S	n/a	R	S	U	S
A5s300	British Columbia Centre for Disease Control	Canada	S-type	PRJNA413593	S	S	S	n/a	S	S	S	S
A6s103	British Columbia Centre for Disease Control	Canada	Beijing	PRJNA413593	S	S	S	n/a	S	S	S	S
10s327	British Columbia Centre for Disease Control	Canada	S-type	PRJNA413593	S	S	S	n/a	S	S	U	S
A8s135	British Columbia Centre for Disease Control	Canada	Beijing	PRJNA413593	S	S	S	n/a	S	S	S	S
14s303	British Columbia Centre for Disease Control	Canada	S-type	PRJNA413593	S	S	S	n/a	S	U	S	S
14s198	British Columbia Centre for Disease Control	Canada	Delhi	PRJNA413593	S	S	S	n/a	S	S	S	S
14s192	British Columbia Centre for Disease Control	Canada	EAI	PRJNA413593	R	S	S	S	F	S	S	U
13s467	British Columbia Centre for Disease Control	Canada		PRJNA413593	S	S	S	n/a	S	S	S	S
13s237	British Columbia Centre for Disease Control	Canada	Beijing	PRJNA413593	S	S	S	n/a	S	S	S	S
A6s056	British Columbia Centre for Disease Control	Canada	LAM	PRJNA413593	S	S	R	n/a	S	S	S	S
A7s020	British Columbia Centre for Disease Control	Canada	S-type	PRJNA413593	S	S	S	n/a	S	S	S	S
11s404	British Columbia Centre for Disease Control	Canada	LAM	PRJNA413593	S	S	S	n/a	S	S	S	S
13s077	British Columbia Centre for Disease Control	Canada	Delhi	PRJNA413593	S	S	S	n/a	S	S	S	S
10s236	British Columbia Centre for Disease Control	Canada	Haarlem	PRJNA413593	S	S	S	n/a	S	S	S	S
11s229	British Columbia Centre for Disease Control	Canada	lineage4	PRJNA413593	S	S	S	n/a	S	S	S	S
A9s219	British Columbia Centre for Disease Control	Canada	Beijing	PRJNA413593	S	S	S	n/a	S	S	S	S
A8s210	British Columbia Centre for Disease Control	Canada	S-type	PRJNA413593	S	S	S	n/a	S	U	S	S
A9s295	British Columbia Centre for Disease Control	Canada	lineage4	PRJNA413593	S	S	S	n/a	S	S	S	S
A7s138	British Columbia Centre for Disease Control	Canada	EAI	PRJNA413593	S	S	S	n/a	S	S	S	S
A7s180	British Columbia Centre for Disease Control	Canada	S-type	PRJNA413593	S	S	S	n/a	S	U	S	S
10s095	British Columbia Centre for Disease Control	Canada	EAI	PRJNA413593	S	S	S	n/a	S	S	S	S
11s070	British Columbia Centre for Disease Control	Canada	Beijing	PRJNA413593	R	S	S	S	U	S	S	S
11s129	British Columbia Centre for Disease Control	Canada	EAI	PRJNA413593	S	S	S	n/a	U	S	S	R
12s398	British Columbia Centre for Disease Control	Canada	LAM	PRJNA413593	S	S	S	n/a	S	S	S	S

A8s061	British Columbia Centre for Disease Control	Canada	Beijing	PRJNA413593	S	S	S	n/a	U	S	S	S
A8s163	British Columbia Centre for Disease Control	Canada	Beijing	PRJNA413593	R	S	S	n/a	R	S	S	S
14s190	British Columbia Centre for Disease Control	Canada	Beijing	PRJNA413593	S	S	S	n/a	S	S	S	S
A5s062	British Columbia Centre for Disease Control	Canada	lineage4	PRJNA413593	S	S	S	n/a	S	S	S	S
10s005	British Columbia Centre for Disease Control	Canada	S-type	PRJNA413593	S	S	S	n/a	S	S	S	S
A8s029	British Columbia Centre for Disease Control	Canada	S-type	PRJNA413593	S	R	S	n/a	S	R	S	S
A8s062	British Columbia Centre for Disease Control	Canada	EAI	PRJNA413593	R	S	S	S	R	S	S	S
A8s265	British Columbia Centre for Disease Control	Canada	Delhi	PRJNA413593	S	S	S	n/a	S	S	S	S
A6s253	British Columbia Centre for Disease Control	Canada	Beijing	PRJNA413593	S	S	S	n/a	S	S	S	S
A8s197	British Columbia Centre for Disease Control	Canada	Beijing	PRJNA413593	S	S	S	n/a	S	S	S	S
10s152	British Columbia Centre for Disease Control	Canada	EAI	PRJNA413593	R	S	S	S	F	S	S	S
A7s072	British Columbia Centre for Disease Control	Canada	EAI	PRJNA413593	S	S	S	n/a	S	S	S	S
11s046	British Columbia Centre for Disease Control	Canada	EAI	PRJNA413593	R	S	S	S	R	S	S	S
A8s316	British Columbia Centre for Disease Control	Canada	EAI	PRJNA413593	R	S	S	S	R	S	S	S
11s073	British Columbia Centre for Disease Control	Canada	Beijing	PRJNA413593	S	S	S	n/a	S	S	S	S
A4s002	British Columbia Centre for Disease Control	Canada	EAI	PRJNA413593	S	S	S	n/a	S	S	S	S
A9s014	British Columbia Centre for Disease Control	Canada	Haarlem	PRJNA413593	S	S	S	n/a	S	S	S	S
14s416	British Columbia Centre for Disease Control	Canada	EAI	PRJNA413593	R	S	S	S	R	S	S	S
A7s105	British Columbia Centre for Disease Control	Canada	S-type	PRJNA413593	S	S	S	n/a	U	S	S	S
10s055	British Columbia Centre for Disease Control	Canada	EAI	PRJNA413593	S	S	S	n/a	S	S	S	S
A6s444	British Columbia Centre for Disease Control	Canada	EAI	PRJNA413593	S	S	S	n/a	S	S	S	S
14s200	British Columbia Centre for Disease Control	Canada	EAI	PRJNA413593	S	S	S	n/a	S	S	S	S
11s351	British Columbia Centre for Disease Control	Canada	Beijing	PRJNA413593	S	S	S	n/a	S	S	S	S
A6s239	British Columbia Centre for Disease Control	Canada	Beijing	PRJNA413593	S	S	S	n/a	S	S	S	S
A6s130	British Columbia Centre for Disease Control	Canada	Beijing	PRJNA413593	S	S	S	n/a	S	S	S	S
10s417	British Columbia Centre for Disease Control	Canada	Delhi	PRJNA413593	S	S	S	n/a	S	S	S	S
12s117	British Columbia Centre for Disease Control	Canada	EAI	PRJNA413593	S	S	S	n/a	S	S	S	S
A1s388	British Columbia Centre for Disease Control	Canada	Beijing	PRJNA413593	S	S	S	n/a	R	S	S	S
A5s169	British Columbia Centre for Disease Control	Canada	Haarlem	PRJNA413593	S	S	S	n/a	S	S	S	S
A9s212	British Columbia Centre for Disease Control	Canada	Beijing	PRJNA413593	S	S	S	n/a	S	S	S	S
12s066	British Columbia Centre for Disease Control	Canada	EAI	PRJNA413593	S	S	S	n/a	S	S	S	S
14s353	British Columbia Centre for Disease Control	Canada	Beijing	PRJNA413593	S	S	S	n/a	S	S	S	S
11s026	British Columbia Centre for Disease Control	Canada	S-type	PRJNA413593	S	S	S	n/a	S	U	S	S
13s131	British Columbia Centre for Disease Control	Canada	lineage4	PRJNA413593	S	S	S	n/a	S	S	S	S
A6s078	British Columbia Centre for Disease Control	Canada	S-type	PRJNA413593	S	S	S	n/a	S	S	S	S
10s280	British Columbia Centre for Disease Control	Canada	LAM	PRJNA413593	S	S	S	n/a	S	S	S	S
A5s021	British Columbia Centre for Disease Control	Canada	S-type	PRJNA413593	S	S	S	S	S	U	S	S
11s144	British Columbia Centre for Disease Control	Canada	Delhi	PRJNA413593	S	S	S	n/a	S	S	S	S
A6s274	British Columbia Centre for Disease Control	Canada	Delhi	PRJNA413593	S	S	S	n/a	S	S	S	S
A7s286	British Columbia Centre for Disease Control	Canada	Haarlem	PRJNA413593	S	S	S	n/a	S	S	S	S
A5s289	British Columbia Centre for Disease Control	Canada	Beijing	PRJNA413593	S	S	S	n/a	S	S	S	S
14s475	British Columbia Centre for Disease Control	Canada	Beijing	PRJNA413593	R	S	S	S	R	S	S	S
A5s132	British Columbia Centre for Disease Control	Canada	Beijing	PRJNA413593	S	S	S	n/a	S	S	S	S
A6s030	British Columbia Centre for Disease Control	Canada	EAI	PRJNA413593	S	S	S	n/a	S	S	S	S
13s288	British Columbia Centre for Disease Control	Canada	EAI	PRJNA413593	S	S	S	n/a	S	S	S	S
A4s042	British Columbia Centre for Disease Control	Canada	Beijing	PRJNA413593	n/a	S	S	S	U	S	S	S
A6s152	British Columbia Centre for Disease Control	Canada	S-type	PRJNA413593	S	S	S	n/a	U	S	S	S
A5s161	British Columbia Centre for Disease Control	Canada	Ural	PRJNA413593	S	S	S	n/a	S	S	S	S
11s066	British Columbia Centre for Disease Control	Canada	EAI	PRJNA413593	S	S	S	n/a	S	S	S	S
13s128	British Columbia Centre for Disease Control	Canada	lineage4	PRJNA413593	S	S	S	n/a	S	S	S	S
A6s006	British Columbia Centre for Disease Control	Canada	Beijing	PRJNA413593	S	S	S	n/a	S	S	S	S
A7s253	British Columbia Centre for Disease Control	Canada	Beijing	PRJNA413593	S	S	S	n/a	S	S	S	S
A9s213	British Columbia Centre for Disease Control	Canada	S-type	PRJNA413593	S	S	S	n/a	S	S	U	S
11s437	British Columbia Centre for Disease Control	Canada	Delhi	PRJNA413593	S	S	S	n/a	S	S	S	S
A7s168	British Columbia Centre for Disease Control	Canada	Haarlem	PRJNA413593	S	S	S	n/a	S	S	S	S
13s403	British Columbia Centre for Disease Control	Canada	Ural	PRJNA413593	S	S	S	n/a	S	S	S	S
11s242	British Columbia Centre for Disease Control	Canada	EAI	PRJNA413593	S	S	S	n/a	S	S	S	U
14s133	British Columbia Centre for Disease Control	Canada	lineage4	PRJNA413593	S	S	S	n/a	S	S	S	S
A7s191	British Columbia Centre for Disease Control	Canada	S-type	PRJNA413593	S	S	S	n/a	S	S	S	S
14s296	British Columbia Centre for Disease Control	Canada	Beijing	PRJNA413593	S	S	S	n/a	S	S	S	S
A6s371	British Columbia Centre for Disease Control	Canada	EAI	PRJNA413593	S	S	S	n/a	S	S	S	S
A4s066	British Columbia Centre for Disease Control	Canada	Ural	PRJNA413593	S	S	S	n/a	S	S	S	S
14s269	British Columbia Centre for Disease Control	Canada	Haarlem	PRJNA413593	S	S	S	n/a	S	S	S	S
A5s193	British Columbia Centre for Disease Control	Canada	Delhi	PRJNA413593	S	S	S	n/a	S	S	S	S
A9s281	British Columbia Centre for Disease Control	Canada	EAI	PRJNA413593	S	S	S	n/a	S	S	S	S
A5s115	British Columbia Centre for Disease Control	Canada	Delhi	PRJNA413593	S	S	S	n/a	S	S	S	S
A7s257	British Columbia Centre for Disease Control	Canada	Delhi	PRJNA413593	S	S	S	n/a	S	S	S	S
A6s153	British Columbia Centre for Disease Control	Canada	S-type	PRJNA413593	S	R	S	n/a	S	R	S	S
10s259	British Columbia Centre for Disease Control	Canada	Ural	PRJNA413593	S	S	S	n/a	S	S	S	S
A5s120	British Columbia Centre for Disease Control	Canada	Ural	PRJNA413593	S	S	S	n/a	S	S	S	S
A9s115	British Columbia Centre for Disease Control	Canada	EAI	PRJNA413593	S	S	S	n/a	U	S	S	S
10s049	British Columbia Centre for Disease Control	Canada	Beijing	PRJNA413593	S	S	S	n/a	S	S	S	S
A4s006	British Columbia Centre for Disease Control	Canada	Beijing	PRJNA413593	S	S	S	n/a	S	S	S	S
13s029	British Columbia Centre for Disease Control	Canada	Delhi	PRJNA413593	S	S	S	n/a	S	S	S	S
A9s388	British Columbia Centre for Disease Control	Canada	Beijing	PRJNA413593	S	S	S	n/a	U	S	S	S
A9s259	British Columbia Centre for Disease Control	Canada	Beijing	PRJNA413593	S	S	S	n/a	S	S	S	S
14s352	British Columbia Centre for Disease Control	Canada	Haarlem	PRJNA413593	S	S	S	n/a	S	S	S	S
A7s178	British Columbia Centre for Disease Control	Canada	EAI	PRJNA413593	S	S	S	n/a	S	S	S	S
10s265	British Columbia Centre for Disease Control	Canada	LAM	PRJNA413593	R	S	S	S	U	S	S	S
12s063	British Columbia Centre for Disease Control	Canada	S-type	PRJNA413593	S	S	S	n/a	F	U	S	S
A6s316	British Columbia Centre for Disease Control	Canada	Delhi	PRJNA413593	R	S	S	n/a	R	S	S	S
11s461	British Columbia Centre for Disease Control	Canada	Ural	PRJNA413593	S	S	S	n/a	S	S	S	S
A8s112	British Columbia Centre for Disease Control	Canada	S-type	PRJNA413593	S	S	S	n/a	U	S	S	S
A7s010	British Columbia Centre for Disease Control	Canada	Uganda	PRJNA413593	S	S	S	n/a	U	S	U	S
12s037	British Columbia Centre for Disease Control	Canada	lineage4	PRJNA413593	S	S	S	n/a	U	S	S	S
A8s159	British Columbia Centre for Disease Control	Canada	Beijing	PRJNA413593	S	S	S	n/a	S	S	S	S
11s231	British Columbia Centre for Disease Control	Canada	lineage4	PRJNA413593	S	S	S	n/a	S	S	S	S
A7s317	British Columbia Centre for Disease Control	Canada	Ural	PRJNA413593	S	S	S	n/a	S	S	S	S
11s463	British Columbia Centre for Disease Control	Canada	lineage4	PRJNA413593	S	S	S	n/a	S	S	U	S
A8s140	British Columbia Centre for Disease Control	Canada	Haarlem	PRJNA413593	S	S	S	n/a	S	S	S	S
10s389	British Columbia Centre for Disease Control	Canada	lineage4	PRJNA413593	R	S	S	S	R	S	S	S
13s372	British Columbia Centre for Disease Control	Canada	Ural	PRJNA413593	S	S	S	n/a	S	S	S	S
11s008	British Columbia Centre for Disease Control	Canada	lineage4	PRJNA413593	S	S	S	n/a	S	S	S	S
11s123	British Columbia Centre for Disease Control	Canada	lineage4	PRJNA413593	R	S	S	S	R	S	S	S
11s441	British Columbia Centre for Disease Control	Canada	S-type	PRJNA413593	S	S	S	n/a	S	U	S	S
13s365	British Columbia Centre for Disease Control	Canada	Beijing	PRJNA413593	R	S	S	S	R	S	U	S
A4s008	British Columbia Centre for Disease Control	Canada	S-type	PRJNA413593	S	S	S	n/a	S	U	S	S
12s410	British Columbia Centre for Disease Control	Canada	S-type	PRJNA413593	S	S	S	n/a	S	U	S	S
13s072	British Columbia Centre for Disease Control	Canada	EAI	PRJNA413593	R	S	S	S	R	S	S	S
11s375	British Columbia Centre for Disease Control	Canada	Beijing	PRJNA413593	S	S	S	n/a	S	S	S	S
10s160	British Columbia Centre for Disease Control	Canada	Delhi	PRJNA413593	S	S	S	n/a	S	S	U	S
13s491	British Columbia Centre for Disease Control	Canada	Haarlem	PRJNA413593	S	S	S	n/a	S	S	S	S
12s186	British Columbia Centre for Disease Control	Canada	Beijing	PRJNA413593	S	S	S	n/a	S	S	S	S
A7s043	British Columbia Centre for Disease Control	Canada	lineage4	PRJNA413593	S	S	S	n/a	S	S	S	S
A8s293	British Columbia Centre for Disease Control	Canada	S-type	PRJNA413593	S	S	S	n/a	S	S	S	S
12s234	British Columbia Centre for Disease Control	Canada	Beijing	PRJNA413593	R	R	R	R	R	R	R	R
11s122	British Columbia Centre for Disease Control	Canada	Beijing	PRJNA413593	S	S	S	n/a	S	S	S	S
13s121	British Columbia Centre for Disease Control	Canada	EAI	PRJNA413593	S	S	S	n/a	S	S	U	S
11s104	British Columbia Centre for Disease Control	Canada	lineage4	PRJNA413593	R	S	S	S	R	S	S	S
A7s273	British Columbia Centre for Disease Control	Canada	S-type	PRJNA413593	S	S	S	n/a	S	U	S	S
A8s109	British Columbia Centre for Disease Control	Canada	LAM	PRJNA413593	S	S	S	n/a	S	S	S	S
A6s014	British Columbia Centre for Disease Control	Canada	S-type	PRJNA413593	S	S	S	n/a	S	S	S	S
A8s299	British Columbia Centre for Disease Control	Canada	Beijing	PRJNA413593	S	S	S	n/a	S	S	S	S
A8s123	British Columbia Centre for Disease Control	Canada	S-type	PRJNA413593	S	S	S	n/a	S	S	U	S
14s076	British Columbia Centre for Disease Control	Canada	lineage4	PRJNA413593	S	S	S	n/a	S	S	U	S
11s279	British Columbia Centre for Disease Control	Canada	EAI	PRJNA413593	S	S	S	n/a	S	S	S	S

A9s390	British Columbia Centre for Disease Control	Canada	EAI	PRJNA413593	S	S	S	n/a	S	S	S	S
10s353	British Columbia Centre for Disease Control	Canada	LAM	PRJNA413593	S	S	S	n/a	S	S	U	S
12s193	British Columbia Centre for Disease Control	Canada	Delhi	PRJNA413593	S	S	S	n/a	S	S	S	S
A5s112	British Columbia Centre for Disease Control	Canada	EAI	PRJNA413593	S	S	S	n/a	S	S	S	S
11s329	British Columbia Centre for Disease Control	Canada	EAI	PRJNA413593	R	S	S	S	R	S	S	S
13s310	British Columbia Centre for Disease Control	Canada	lineage4	PRJNA413593	S	S	S	n/a	R	S	S	S
11s361	British Columbia Centre for Disease Control	Canada	Delhi	PRJNA413593	S	S	S	n/a	U	S	S	S
13s313	British Columbia Centre for Disease Control	Canada	lineage4	PRJNA413593	S	S	S	n/a	S	S	S	S
A9s007	British Columbia Centre for Disease Control	Canada	EAI	PRJNA413593	R	S	S	n/a	R	U	S	S
A9s377	British Columbia Centre for Disease Control	Canada	EAI	PRJNA413593	S	S	S	n/a	U	S	S	S
13s373	British Columbia Centre for Disease Control	Canada	lineage4	PRJNA413593	S	S	S	n/a	S	S	U	S
A9s264	British Columbia Centre for Disease Control	Canada	Beijing	PRJNA413593	S	S	S	n/a	S	S	S	S
11s173	British Columbia Centre for Disease Control	Canada	Ural	PRJNA413593	S	S	S	n/a	S	S	S	S
12s034	British Columbia Centre for Disease Control	Canada	S-type	PRJNA413593	S	S	S	n/a	S	U	S	S
A8s332	British Columbia Centre for Disease Control	Canada	Beijing	PRJNA413593	R	S	S	n/a	R	S	S	S
12s069	British Columbia Centre for Disease Control	Canada	Beijing	PRJNA413593	S	S	S	n/a	S	S	S	S
A8s054	British Columbia Centre for Disease Control	Canada	Beijing	PRJNA413593	S	S	S	n/a	S	S	S	S
10s092	British Columbia Centre for Disease Control	Canada	Beijing	PRJNA413593	S	S	S	n/a	S	S	S	S
10s022	British Columbia Centre for Disease Control	Canada	Delhi	PRJNA413593	S	S	S	n/a	S	S	S	S
A7s112	British Columbia Centre for Disease Control	Canada	Beijing	PRJNA413593	S	S	S	n/a	S	S	S	S
11s246	British Columbia Centre for Disease Control	Canada	LAM	PRJNA413593	S	S	S	n/a	S	S	S	S
11s323	British Columbia Centre for Disease Control	Canada	S-type	PRJNA413593	S	S	S	n/a	S	U	S	S
A9s096	British Columbia Centre for Disease Control	Canada	lineage4	PRJNA413593	S	S	S	n/a	S	S	U	S
A6s120	British Columbia Centre for Disease Control	Canada	S-type	PRJNA413593	S	S	S	n/a	S	U	S	S
A8s234	British Columbia Centre for Disease Control	Canada	Beijing	PRJNA413593	S	S	S	n/a	S	S	S	S
A9s323	British Columbia Centre for Disease Control	Canada	S-type	PRJNA413593	S	R	S	n/a	S	R	S	S
A8s043	British Columbia Centre for Disease Control	Canada	Ural	PRJNA413593	S	S	S	n/a	S	S	U	S
10s053	British Columbia Centre for Disease Control	Canada	Beijing	PRJNA413593	S	S	S	n/a	S	S	S	S
A9s235	British Columbia Centre for Disease Control	Canada	Haarlem	PRJNA413593	S	S	S	n/a	S	S	S	S
A5s014	British Columbia Centre for Disease Control	Canada	Ural	PRJNA413593	S	S	S	n/a	S	S	S	S
10s234	British Columbia Centre for Disease Control	Canada	Beijing	PRJNA413593	S	S	S	n/a	S	S	S	S
A9s405	British Columbia Centre for Disease Control	Canada	S-type	PRJNA413593	S	S	S	n/a	S	U	S	S
A9s346	British Columbia Centre for Disease Control	Canada	Delhi	PRJNA413593	S	S	S	n/a	S	S	U	S
A6s431	British Columbia Centre for Disease Control	Canada	EAI	PRJNA413593	S	S	S	n/a	S	S	S	S
A6s368	British Columbia Centre for Disease Control	Canada	EAI	PRJNA413593	S	S	S	n/a	S	S	S	S
A4s086	British Columbia Centre for Disease Control	Canada	LAM	PRJNA413593	S	S	S	n/a	S	S	S	S
13s292	British Columbia Centre for Disease Control	Canada	EAI	PRJNA413593	S	S	S	n/a	S	S	S	S
A5s254	British Columbia Centre for Disease Control	Canada	Haarlem	PRJNA413593	S	S	S	n/a	S	S	S	S
A7s007	British Columbia Centre for Disease Control	Canada	Beijing	PRJNA413593	S	S	S	n/a	S	S	S	S
13s456	British Columbia Centre for Disease Control	Canada	Beijing	PRJNA413593	S	S	S	n/a	S	S	S	S
A3s053	British Columbia Centre for Disease Control	Canada	Beijing	PRJNA413593	S	S	S	n/a	S	S	S	S
14s395	British Columbia Centre for Disease Control	Canada	Delhi	PRJNA413593	S	S	S	n/a	S	S	S	S
A5s232	British Columbia Centre for Disease Control	Canada	Ural	PRJNA413593	S	S	S	n/a	S	S	S	S
10s256	British Columbia Centre for Disease Control	Canada	S-type	PRJNA413593	S	S	S	n/a	S	U	S	S
A8s240	British Columbia Centre for Disease Control	Canada	Beijing	PRJNA413593	R	S	S	n/a	U	S	S	S
A5s093	British Columbia Centre for Disease Control	Canada	EAI	PRJNA413593	S	S	S	n/a	S	S	S	S
13s502	British Columbia Centre for Disease Control	Canada	lineage4	PRJNA413593	S	S	S	n/a	S	S	U	S
A8s356	British Columbia Centre for Disease Control	Canada	Delhi	PRJNA413593	S	S	S	n/a	S	S	S	S
14s145	British Columbia Centre for Disease Control	Canada	EAI	PRJNA413593	S	S	S	n/a	S	S	S	S
12s087	British Columbia Centre for Disease Control	Canada	Beijing	PRJNA413593	S	S	S	n/a	S	S	S	S
12s172	British Columbia Centre for Disease Control	Canada	Delhi	PRJNA413593	S	S	S	n/a	S	S	S	S
12s094	British Columbia Centre for Disease Control	Canada	LAM	PRJNA413593	S	S	S	n/a	S	S	S	S
A8s174	British Columbia Centre for Disease Control	Canada	S-type	PRJNA413593	S	S	S	n/a	S	S	S	S
12s304	British Columbia Centre for Disease Control	Canada	Beijing	PRJNA413593	S	S	S	n/a	S	S	S	S
A9s398	British Columbia Centre for Disease Control	Canada	EAI	PRJNA413593	S	S	S	n/a	S	S	S	S
13s023	British Columbia Centre for Disease Control	Canada	Delhi	PRJNA413593	S	S	S	n/a	S	S	S	S
A6s131	British Columbia Centre for Disease Control	Canada	Beijing	PRJNA413593	R	S	S	S	R	S	S	S
A4s018	British Columbia Centre for Disease Control	Canada	Beijing	PRJNA413593	R	R	R	R	R	R	R	U
11s284	British Columbia Centre for Disease Control	Canada	EAI	PRJNA413593	S	S	S	n/a	U	S	S	S
12s082	British Columbia Centre for Disease Control	Canada	EAI	PRJNA413593	S	S	S	n/a	S	S	S	S
A8s219	British Columbia Centre for Disease Control	Canada	LAM	PRJNA413593	S	S	S	n/a	S	S	S	S
A5s178	British Columbia Centre for Disease Control	Canada	Ural	PRJNA413593	S	S	S	n/a	S	S	S	S
A7s294	British Columbia Centre for Disease Control	Canada	S-type	PRJNA413593	S	S	S	n/a	S	S	S	S
11s348	British Columbia Centre for Disease Control	Canada	LAM	PRJNA413593	S	S	S	n/a	S	S	S	S
A9s251	British Columbia Centre for Disease Control	Canada	Delhi	PRJNA413593	S	S	S	n/a	S	U	S	S
10s444	British Columbia Centre for Disease Control	Canada	Beijing	PRJNA413593	R	S	S	S	R	S	S	S
12s212	British Columbia Centre for Disease Control	Canada	EAI	PRJNA413593	S	S	S	n/a	S	S	S	S
12s326	British Columbia Centre for Disease Control	Canada	lineage4	PRJNA413593	S	S	S	S	R	S	S	S
A6s074	British Columbia Centre for Disease Control	Canada	Delhi	PRJNA413593	R	S	S	S	U	S	S	S
A5s051	British Columbia Centre for Disease Control	Canada	Ural	PRJNA413593	S	S	S	n/a	S	S	S	S
A9s248	British Columbia Centre for Disease Control	Canada	Haarlem	PRJNA413593	S	S	S	n/a	U	S	S	S
A7s167	British Columbia Centre for Disease Control	Canada	Beijing	PRJNA413593	S	S	S	n/a	S	S	S	S
12s155	British Columbia Centre for Disease Control	Canada	S-type	PRJNA413593	S	S	S	n/a	U	S	S	S
A8s095	British Columbia Centre for Disease Control	Canada	lineage4	PRJNA413593	R	S	S	n/a	R	S	S	S
A7s333	British Columbia Centre for Disease Control	Canada	Beijing	PRJNA413593	R	R	R	R	R	R	R	R
12s204	British Columbia Centre for Disease Control	Canada	EAI	PRJNA413593	S	S	S	n/a	S	S	S	S
14s166	British Columbia Centre for Disease Control	Canada	EAI	PRJNA413593	S	S	S	S	S	S	S	S
A8s143	British Columbia Centre for Disease Control	Canada	Beijing	PRJNA413593	S	S	S	n/a	S	S	S	S
13s079	British Columbia Centre for Disease Control	Canada	EAI	PRJNA413593	S	S	S	n/a	S	S	S	S
A5s140	British Columbia Centre for Disease Control	Canada	Beijing	PRJNA413593	S	S	S	n/a	S	S	S	S
A5s279	British Columbia Centre for Disease Control	Canada	S-type	PRJNA413593	S	R	S	S	S	R	S	S
13s006	British Columbia Centre for Disease Control	Canada	lineage4	PRJNA413593	S	S	S	n/a	S	S	U	S
11s021	British Columbia Centre for Disease Control	Canada	lineage4	PRJNA413593	S	S	S	S	S	S	S	S
11s252	British Columbia Centre for Disease Control	Canada	EAI	PRJNA413593	S	S	S	n/a	S	U	S	S
A7s315	British Columbia Centre for Disease Control	Canada	Delhi	PRJNA413593	S	S	S	n/a	S	S	S	S
14s129	British Columbia Centre for Disease Control	Canada	Beijing	PRJNA413593	S	S	S	n/a	S	S	S	S
A9s064	British Columbia Centre for Disease Control	Canada	Beijing	PRJNA413593	S	S	S	n/a	S	S	S	S
10s121	British Columbia Centre for Disease Control	Canada	LAM	PRJNA413593	S	S	S	n/a	S	S	S	S
A5s038	British Columbia Centre for Disease Control	Canada	S-type	PRJNA413593	S	S	S	n/a	S	U	S	S
13s174	British Columbia Centre for Disease Control	Canada	EAI	PRJNA413593	S	S	S	n/a	S	S	S	S
A9s044	British Columbia Centre for Disease Control	Canada	EAI	PRJNA413593	S	S	S	n/a	S	S	S	S
A6s043	British Columbia Centre for Disease Control	Canada	Beijing	PRJNA413593	S	S	S	n/a	S	S	S	S
A8s063	British Columbia Centre for Disease Control	Canada	S-type	PRJNA413593	S	S	S	n/a	U	S	S	S
A5s163	British Columbia Centre for Disease Control	Canada	S-type	PRJNA413593	S	S	S	n/a	S	S	S	S
A9s328	British Columbia Centre for Disease Control	Canada	Delhi	PRJNA413593	R	S	S	S	R	S	R	S
12s419	British Columbia Centre for Disease Control	Canada	lineage4	PRJNA413593	S	S	S	n/a	S	S	U	S
10s173	British Columbia Centre for Disease Control	Canada	Beijing	PRJNA413593	S	S	S	n/a	S	S	S	S
A7s172	British Columbia Centre for Disease Control	Canada	lineage4	PRJNA413593	S	S	S	n/a	S	S	S	S
13s466	British Columbia Centre for Disease Control	Canada	Ural	PRJNA413593	S	S	S	n/a	S	S	S	S
A6s315	British Columbia Centre for Disease Control	Canada	EAI	PRJNA413593	S	S	S	n/a	S	S	S	S
A8s358	British Columbia Centre for Disease Control	Canada	S-type	PRJNA413593	S	S	S	n/a	S	U	S	S
12s436	British Columbia Centre for Disease Control	Canada	Ural	PRJNA413593	S	S	S	n/a	S	S	S	S
A9s104	British Columbia Centre for Disease Control	Canada	lineage4	PRJNA413593	R	S	S	n/a	R	S	S	S
12s338	British Columbia Centre for Disease Control	Canada	Beijing	PRJNA413593	R	R	S	R	R	R	S	R
A9s112	British Columbia Centre for Disease Control	Canada	Ural	PRJNA413593	S	S	S	n/a	S	S	S	S
A9s062	British Columbia Centre for Disease Control	Canada	Haarlem	PRJNA413593	S	S	S	n/a	S	S	S	S
A7s123	British Columbia Centre for Disease Control	Canada	Ural	PRJNA413593	S	S	S	n/a	S	S	S	S
A7s045	British Columbia Centre for Disease Control	Canada	Beijing	PRJNA413593	R	S	S	n/a	R	S	S	S
A8s363	British Columbia Centre for Disease Control	Canada	EAI	PRJNA413593	S	S	S	n/a	S	S	S	S
A8s104	British Columbia Centre for Disease Control	Canada	Beijing	PRJNA413593	S	S	S	n/a	S	S	S	S
A6s128	British Columbia Centre for Disease Control	Canada	Haarlem	PRJNA413593	S	S	S	n/a	S	S	S	S
10s071	British Columbia Centre for Disease Control	Canada	Beijing	PRJNA413593	S	S	S	n/a	S	S	S	S
A7s321	British Columbia Centre for Disease Control	Canada	EAI	PRJNA413593	S	S	S	n/a	S	S	S	S
A3s233	British Columbia Centre for Disease Control	Canada	Beijing	PRJNA413593	S	S	S	n/a	S	S	S	S
A6s186	British Columbia Centre for Disease Control	Canada	Beijing	PRJNA413593	R	S	S	n/a	R	S	U	S
10s203	British Columbia Centre for Disease Control	Canada	Haarlem	PRJNA413593	S	S	S	S	S	S	S	S

12s074	British Columbia Centre for Disease Control	Canada	Delhi	PRJNA413593	S	S	S	n/a	S	S	S	S
A5s181	British Columbia Centre for Disease Control	Canada	EAI	PRJNA413593	R	S	S	S	R	S	S	S
11s395	British Columbia Centre for Disease Control	Canada	Ural	PRJNA413593	S	S	S	n/a	S	S	S	S
A8s212	British Columbia Centre for Disease Control	Canada	lineage4	PRJNA413593	S	S	S	n/a	S	S	S	S
12s280	British Columbia Centre for Disease Control	Canada	Beijing	PRJNA413593	S	S	S	n/a	S	S	S	S
11s038	British Columbia Centre for Disease Control	Canada	S-type	PRJNA413593	S	S	S	n/a	S	S	S	S
12s312	British Columbia Centre for Disease Control	Canada	Haarlem	PRJNA413593	S	S	S	n/a	S	S	S	S
10s432	British Columbia Centre for Disease Control	Canada	lineage4	PRJNA413593	R	S	S	S	R	S	S	S
13s301	British Columbia Centre for Disease Control	Canada	Ural	PRJNA413593	S	S	S	n/a	S	S	S	S
A6s094	British Columbia Centre for Disease Control	Canada	Beijing	PRJNA413593	S	S	S	n/a	S	S	S	S
A9s087	British Columbia Centre for Disease Control	Canada	EAI	PRJNA413593	S	S	S	n/a	S	S	S	S
A5s240	British Columbia Centre for Disease Control	Canada	EAI	PRJNA413593	S	S	S	n/a	S	S	S	S
A6s217	British Columbia Centre for Disease Control	Canada	Beijing	PRJNA413593	S	S	S	n/a	S	S	S	S
A6s366	British Columbia Centre for Disease Control	Canada	Beijing	PRJNA413593	S	S	S	n/a	S	S	S	S
11s438	British Columbia Centre for Disease Control	Canada	EAI	PRJNA413593	S	S	S	n/a	S	S	S	S
A8s139	British Columbia Centre for Disease Control	Canada	S-type	PRJNA413593	S	S	S	n/a	U	S	S	S
A6s144	British Columbia Centre for Disease Control	Canada	EAI	PRJNA413593	S	S	S	n/a	S	S	S	S
14s041	British Columbia Centre for Disease Control	Canada	lineage4	PRJNA413593	S	S	S	n/a	U	S	S	S
A7s246	British Columbia Centre for Disease Control	Canada	Beijing	PRJNA413593	S	S	S	n/a	S	S	S	S
11s157	British Columbia Centre for Disease Control	Canada	EAI	PRJNA413593	S	S	S	n/a	S	S	S	S
A6s077	British Columbia Centre for Disease Control	Canada	EAI	PRJNA413593	S	S	S	n/a	S	S	S	S
14s457	British Columbia Centre for Disease Control	Canada	EAI	PRJNA413593	R	R	S	R	R	R	U	R
A4s109	British Columbia Centre for Disease Control	Canada	Tur	PRJNA413593	S	S	S	n/a	S	S	S	S
A7s268	British Columbia Centre for Disease Control	Canada	Beijing	PRJNA413593	S	S	S	n/a	S	S	S	S
14s181	British Columbia Centre for Disease Control	Canada	Beijing	PRJNA413593	R	R	S	S	R	R	S	S
13s013	British Columbia Centre for Disease Control	Canada	lineage4	PRJNA413593	S	S	S	n/a	S	S	U	S
A8s282	British Columbia Centre for Disease Control	Canada	S-type	PRJNA413593	S	R	S	n/a	S	R	S	S
11s105	British Columbia Centre for Disease Control	Canada	lineage4	PRJNA413593	R	S	S	S	R	S	S	S
A7s064	British Columbia Centre for Disease Control	Canada	Ural	PRJNA413593	S	S	S	n/a	S	S	S	S
A6s218	British Columbia Centre for Disease Control	Canada	Ural	PRJNA413593	S	S	S	n/a	S	S	S	S
13s058	British Columbia Centre for Disease Control	Canada	EAI	PRJNA413593	R	S	S	S	R	S	S	S
13s080	British Columbia Centre for Disease Control	Canada	Delhi	PRJNA413593	S	S	S	n/a	S	S	S	S
13s377	British Columbia Centre for Disease Control	Canada	EAI	PRJNA413593	S	S	S	n/a	S	S	S	S
A7S230	British Columbia Centre for Disease Control	Canada	Ural	PRJNA413593	S	S	S	n/a	S	S	S	S
11s161	British Columbia Centre for Disease Control	Canada	Ural	PRJNA413593	S	S	S	n/a	S	S	U	S
A7s005	British Columbia Centre for Disease Control	Canada	Haarlem	PRJNA413593	S	S	S	n/a	S	S	S	S
A9s263	British Columbia Centre for Disease Control	Canada	EAI	PRJNA413593	S	S	S	n/a	S	S	S	S
A6s213	British Columbia Centre for Disease Control	Canada	Beijing	PRJNA413593	R	S	S	n/a	U	S	S	S
A8s168	British Columbia Centre for Disease Control	Canada	Haarlem	PRJNA413593	S	S	S	n/a	S	S	S	S
13s026	British Columbia Centre for Disease Control	Canada	EAI	PRJNA413593	S	S	S	n/a	S	S	S	S
13s329	British Columbia Centre for Disease Control	Canada	Haarlem	PRJNA413593	S	S	S	n/a	S	S	U	S
12s428	British Columbia Centre for Disease Control	Canada	Beijing	PRJNA413593	R	S	S	S	R	S	S	S
10s016	British Columbia Centre for Disease Control	Canada	EAI	PRJNA413593	S	S	R	n/a	S	S	R	S
14s026	British Columbia Centre for Disease Control	Canada	EAI	PRJNA413593	R	S	S	S	R	S	R	S
13s142	British Columbia Centre for Disease Control	Canada	Beijing	PRJNA413593	S	S	S	n/a	S	S	S	S
A6s069	British Columbia Centre for Disease Control	Canada	Delhi	PRJNA413593	S	R	S	S	S	R	S	S
A9s321	British Columbia Centre for Disease Control	Canada	Haarlem	PRJNA413593	S	S	S	n/a	S	S	S	S
A5s285	British Columbia Centre for Disease Control	Canada	Beijing	PRJNA413593	S	S	S	n/a	S	S	S	S
10s223	British Columbia Centre for Disease Control	Canada	Beijing	PRJNA413593	S	S	S	n/a	S	S	U	S
14s012	British Columbia Centre for Disease Control	Canada	EAI	PRJNA413593	S	S	S	n/a	S	S	S	S
A4s019	British Columbia Centre for Disease Control	Canada	S-type	PRJNA413593	S	S	S	n/a	S	U	S	S
A6s319	British Columbia Centre for Disease Control	Canada	lineage4	PRJNA413593	S	S	S	n/a	S	S	S	S
13s190	British Columbia Centre for Disease Control	Canada	Haarlem	PRJNA413593	S	S	S	n/a	S	S	S	S
A8s285	British Columbia Centre for Disease Control	Canada	Haarlem	PRJNA413593	S	S	S	n/a	S	S	S	S
A7s009	British Columbia Centre for Disease Control	Canada	Beijing	PRJNA413593	S	S	S	n/a	S	S	S	S
A9s369	British Columbia Centre for Disease Control	Canada	Beijing	PRJNA413593	S	S	S	n/a	S	S	S	S
11s177	British Columbia Centre for Disease Control	Canada	S-type	PRJNA413593	S	S	S	n/a	S	S	S	S
A5s267	British Columbia Centre for Disease Control	Canada	EAI	PRJNA413593	S	S	S	n/a	S	S	S	S
A9s193	British Columbia Centre for Disease Control	Canada	lineage4	PRJNA413593	S	S	S	n/a	S	S	S	S
A8s016	British Columbia Centre for Disease Control	Canada	Beijing	PRJNA413593	S	S	S	n/a	S	S	S	S
10s399	British Columbia Centre for Disease Control	Canada	Ural	PRJNA413593	S	S	S	n/a	S	S	S	S
A9s241	British Columbia Centre for Disease Control	Canada	EAI	PRJNA413593	S	S	S	n/a	S	S	S	S
12s300	British Columbia Centre for Disease Control	Canada	Beijing	PRJNA413593	S	S	S	n/a	S	S	S	S
A6s331	British Columbia Centre for Disease Control	Canada	Ural	PRJNA413593	S	S	S	n/a	S	S	S	S
A9s314	British Columbia Centre for Disease Control	Canada	lineage4	PRJNA413593	R	S	S	S	R	S	S	S
12s129	British Columbia Centre for Disease Control	Canada	lineage4	PRJNA413593	R	S	S	S	R	S	S	S
14s397	British Columbia Centre for Disease Control	Canada	Delhi	PRJNA413593	R	S	S	S	R	S	S	S
11s116	British Columbia Centre for Disease Control	Canada	Ural	PRJNA413593	S	S	S	n/a	S	S	S	S
A6s013	British Columbia Centre for Disease Control	Canada	Delhi	PRJNA413593	S	R	S	S	S	R	S	S
A9s243	British Columbia Centre for Disease Control	Canada	Delhi	PRJNA413593	S	S	S	n/a	S	S	S	S
A5s252	British Columbia Centre for Disease Control	Canada	Delhi	PRJNA413593	S	S	S	n/a	S	S	S	S
10s336	British Columbia Centre for Disease Control	Canada	Ural	PRJNA413593	S	S	S	n/a	S	S	S	S
14s017	British Columbia Centre for Disease Control	Canada	EAI	PRJNA413593	S	S	S	n/a	S	S	S	S
A9s139	British Columbia Centre for Disease Control	Canada	S-type	PRJNA413593	S	S	S	n/a	S	U	S	S
12s161	British Columbia Centre for Disease Control	Canada	EAI	PRJNA413593	S	S	S	n/a	S	S	S	S
A9s249	British Columbia Centre for Disease Control	Canada	Haarlem	PRJNA413593	S	S	S	n/a	S	S	S	S
13s129	British Columbia Centre for Disease Control	Canada	EAI	PRJNA413593	S	S	S	n/a	S	S	U	S
A7s008	British Columbia Centre for Disease Control	Canada	Beijing	PRJNA413593	S	S	S	n/a	S	S	S	S
11s043	British Columbia Centre for Disease Control	Canada	Haarlem	PRJNA413593	S	S	S	n/a	S	S	S	S
A9s071	British Columbia Centre for Disease Control	Canada	Beijing	PRJNA413593	S	S	S	n/a	S	S	S	S
A9s025	British Columbia Centre for Disease Control	Canada	EAI	PRJNA413593	S	S	S	n/a	U	S	S	S
A7s293	British Columbia Centre for Disease Control	Canada	EAI	PRJNA413593	R	S	S	n/a	R	S	S	S
14s419	British Columbia Centre for Disease Control	Canada	S-type	PRJNA413593	S	S	S	n/a	U	S	S	S
11s087	British Columbia Centre for Disease Control	Canada	Beijing	PRJNA413593	S	S	S	n/a	S	S	S	S
14s313	British Columbia Centre for Disease Control	Canada	EAI	PRJNA413593	S	S	S	n/a	U	S	S	S
A9s379	British Columbia Centre for Disease Control	Canada	S-type	PRJNA413593	S	S	S	n/a	S	U	S	S
A6s327	British Columbia Centre for Disease Control	Canada	S-type	PRJNA413593	S	S	S	n/a	S	S	S	S
A4s099	British Columbia Centre for Disease Control	Canada	Ural	PRJNA413593	S	S	S	n/a	S	S	S	S
A8s194	British Columbia Centre for Disease Control	Canada	Delhi	PRJNA413593	S	S	S	n/a	S	S	U	S
A8s035	British Columbia Centre for Disease Control	Canada	Delhi	PRJNA413593	R	S	S	S	R	S	S	S
11s092	British Columbia Centre for Disease Control	Canada	S-type	PRJNA413593	S	R	S	S	S	R	S	S
13s388	British Columbia Centre for Disease Control	Canada	EAI	PRJNA413593	S	S	S	n/a	S	S	S	S
A9s234	British Columbia Centre for Disease Control	Canada	lineage4	PRJNA413593	S	S	S	n/a	S	S	S	S
12s306	British Columbia Centre for Disease Control	Canada	Beijing	PRJNA413593	S	S	S	n/a	S	S	S	S
A8s263	British Columbia Centre for Disease Control	Canada	S-type	PRJNA413593	S	S	S	n/a	S	U	S	S
12s394	British Columbia Centre for Disease Control	Canada	Beijing	PRJNA413593	S	S	S	n/a	S	S	S	S
14s271	British Columbia Centre for Disease Control	Canada	EAI	PRJNA413593	R	S	S	S	R	S	S	S
A5s185	British Columbia Centre for Disease Control	Canada	EAI	PRJNA413593	R	S	S	S	R	S	U	S
10s266	British Columbia Centre for Disease Control	Canada	Beijing	PRJNA413593	R	S	S	S	R	S	S	S
A8s346	British Columbia Centre for Disease Control	Canada	EAI	PRJNA413593	S	S	S	n/a	S	S	S	S
11s418	British Columbia Centre for Disease Control	Canada	Haarlem	PRJNA413593	S	S	S	n/a	S	S	S	S
A7s220	British Columbia Centre for Disease Control	Canada	Beijing	PRJNA413593	S	S	S	n/a	S	S	S	S
A9s140	British Columbia Centre for Disease Control	Canada	Delhi	PRJNA413593	S	S	S	n/a	S	S	S	S
A9s344	British Columbia Centre for Disease Control	Canada	Beijing	PRJNA413593	R	S	S	S	S	S	S	S
A7s107	British Columbia Centre for Disease Control	Canada	S-type	PRJNA413593	S	S	S	n/a	S	U	S	S
A8s076	British Columbia Centre for Disease Control	Canada	Beijing	PRJNA413593	S	S	S	n/a	S	S	S	S
13s053	British Columbia Centre for Disease Control	Canada	lineage4	PRJNA413593	S	S	S	n/a	R	S	S	S
12s050	British Columbia Centre for Disease Control	Canada	lineage4	PRJNA413593	R	S	S	S	R	S	S	S
14s163	British Columbia Centre for Disease Control	Canada	Beijing	PRJNA413593	S	S	S	n/a	S	S	S	S
A8s073	British Columbia Centre for Disease Control	Canada	lineage4	PRJNA413593	S	S	S	n/a	R	S	S	S
11s230	British Columbia Centre for Disease Control	Canada	Ural	PRJNA413593	S	S	S	n/a	S	S	S	S
12s107	British Columbia Centre for Disease Control	Canada	S-type	PRJNA413593	S	S	S	n/a	S	S	U	S
A6s076	British Columbia Centre for Disease Control	Canada	EAI	PRJNA413593	S	S	S	n/a	S	S	U	S
A6s351	British Columbia Centre for Disease Control	Canada	lineage4	PRJNA413593	S	S	S	n/a	S	S	S	S
A5s107	British Columbia Centre for Disease Control	Canada	lineage4	PRJNA413593	S	S	S	n/a	S	S	U	S
13s498	British Columbia Centre for Disease Control	Canada	Beijing	PRJNA413593	S	S	S	n/a	S	S	S	S

A6s099	British Columbia Centre for Disease Control	Canada	Beijing	PRJNA413593	S	S	S	n/a	S	S	S	S
A6s283	British Columbia Centre for Disease Control	Canada	Beijing	PRJNA413593	S	S	S	n/a	S	S	S	S
10s431	British Columbia Centre for Disease Control	Canada	EAI	PRJNA413593	S	S	S	n/a	S	S	S	S
A7s224	British Columbia Centre for Disease Control	Canada	S-type	PRJNA413593	S	S	S	n/a	S	U	S	S
A6s442	British Columbia Centre for Disease Control	Canada	Ural	PRJNA413593	S	S	S	n/a	S	S	S	S
A8s206	British Columbia Centre for Disease Control	Canada	Beijing	PRJNA413593	S	S	S	n/a	S	S	S	S
14s037	British Columbia Centre for Disease Control	Canada	EAI	PRJNA413593	S	S	S	n/a	S	S	S	S
A4s386	British Columbia Centre for Disease Control	Canada	Ural	PRJNA413593	S	S	S	n/a	S	S	S	S
A4s058	British Columbia Centre for Disease Control	Canada	Ural	PRJNA413593	S	S	S	n/a	S	S	S	S
14s132	British Columbia Centre for Disease Control	Canada	Beijing	PRJNA413593	R	R	R	R	R	R	R	R
A7S346	British Columbia Centre for Disease Control	Canada	Ural	PRJNA413593	S	S	S	n/a	S	S	S	S
A6s237	British Columbia Centre for Disease Control	Canada	LAM	PRJNA413593	S	S	S	n/a	S	S	U	S
10s407	British Columbia Centre for Disease Control	Canada	Delhi	PRJNA413593	S	S	S	n/a	S	S	S	S
11s089	British Columbia Centre for Disease Control	Canada	S-type	PRJNA413593	S	S	S	n/a	S	U	S	S
10s136	British Columbia Centre for Disease Control	Canada	Delhi	PRJNA413593	S	S	S	n/a	S	S	S	S
A6s258	British Columbia Centre for Disease Control	Canada	Beijing	PRJNA413593	S	S	S	n/a	S	S	S	S
A6s399	British Columbia Centre for Disease Control	Canada	Tur	PRJNA413593	S	S	S	n/a	S	S	R	S
A4s027	British Columbia Centre for Disease Control	Canada	Haarlem	PRJNA413593	S	S	n/a	n/a	S	S	S	S
A5s039	British Columbia Centre for Disease Control	Canada	LAM	PRJNA413593	S	S	S	n/a	S	S	S	S
11s114	British Columbia Centre for Disease Control	Canada	lineage4	PRJNA413593	S	S	S	n/a	R	S	S	S
12s315	British Columbia Centre for Disease Control	Canada	Delhi	PRJNA413593	S	S	S	n/a	S	S	S	S
A5s134	British Columbia Centre for Disease Control	Canada	Ural	PRJNA413593	S	S	S	n/a	S	S	S	S
12s431	British Columbia Centre for Disease Control	Canada	lineage4	PRJNA413593	S	S	S	n/a	S	S	U	S
10s255	British Columbia Centre for Disease Control	Canada	Delhi	PRJNA413593	S	S	S	n/a	S	S	S	S
A6s134	British Columbia Centre for Disease Control	Canada	S-type	PRJNA413593	S	S	S	n/a	S	S	S	S
A9s284	British Columbia Centre for Disease Control	Canada	Beijing	PRJNA413593	S	S	S	n/a	S	S	S	S
A9s371	British Columbia Centre for Disease Control	Canada	Delhi	PRJNA413593	R	S	S	n/a	R	S	R	S
A8s002	British Columbia Centre for Disease Control	Canada	Beijing	PRJNA413593	S	S	S	n/a	S	S	S	S
14s116	British Columbia Centre for Disease Control	Canada	lineage4	PRJNA413593	S	S	S	n/a	S	S	S	S
A9s065	British Columbia Centre for Disease Control	Canada	LAM	PRJNA413593	S	S	S	n/a	S	S	S	S
12s026	British Columbia Centre for Disease Control	Canada	Ural	PRJNA413593	S	S	S	n/a	S	S	S	S
14s454	British Columbia Centre for Disease Control	Canada	S-type	PRJNA413593	S	S	S	n/a	U	S	S	S
A7s292	British Columbia Centre for Disease Control	Canada	Delhi	PRJNA413593	S	S	S	n/a	S	S	U	S
A0s338	British Columbia Centre for Disease Control	Canada	Beijing	PRJNA413593	R	R	S	n/a	R	R	S	S
13s103	British Columbia Centre for Disease Control	Canada	Delhi	PRJNA413593	R	S	S	S	R	S	S	S
10s357	British Columbia Centre for Disease Control	Canada	Ural	PRJNA413593	S	S	S	n/a	S	S	S	S
A6s060	British Columbia Centre for Disease Control	Canada	LAM	PRJNA413593	S	S	n/a	n/a	S	S	S	S
A2s244	British Columbia Centre for Disease Control	Canada	lineage4	PRJNA413593	S	S	S	n/a	S	S	U	S
A8s261	British Columbia Centre for Disease Control	Canada	S-type	PRJNA413593	S	S	S	n/a	S	U	S	S
12s341	British Columbia Centre for Disease Control	Canada	EAI	PRJNA413593	R	S	S	S	R	S	S	S
A5s187	British Columbia Centre for Disease Control	Canada	LAM	PRJNA413593	S	S	R	n/a	S	S	S	S
A9s286	British Columbia Centre for Disease Control	Canada	lineage4	PRJNA413593	S	S	S	n/a	S	S	U	S
A7s313	British Columbia Centre for Disease Control	Canada	Delhi	PRJNA413593	S	S	S	n/a	S	S	S	S
13s311	British Columbia Centre for Disease Control	Canada	lineage4	PRJNA413593	S	S	S	n/a	R	S	S	S
11s007	British Columbia Centre for Disease Control	Canada	Beijing	PRJNA413593	R	S	S	R	R	S	S	R
A2s241	British Columbia Centre for Disease Control	Canada	Ural	PRJNA413593	S	S	S	n/a	S	S	S	S
13s367	British Columbia Centre for Disease Control	Canada	EAI	PRJNA413593	S	S	S	n/a	S	S	S	S
11s343	British Columbia Centre for Disease Control	Canada	EAI	PRJNA413593	S	S	S	n/a	S	S	S	S
12s008	British Columbia Centre for Disease Control	Canada	EAI	PRJNA413593	S	S	S	n/a	S	S	S	S
A9s119	British Columbia Centre for Disease Control	Canada	EAI	PRJNA413593	S	S	S	n/a	S	S	S	S
A7s188	British Columbia Centre for Disease Control	Canada	Beijing	PRJNA413593	S	S	S	n/a	S	S	S	S
A7s146	British Columbia Centre for Disease Control	Canada	Delhi	PRJNA413593	S	S	S	n/a	S	S	S	S
11s290	British Columbia Centre for Disease Control	Canada	Beijing	PRJNA413593	S	S	S	n/a	S	S	S	S
12s292	British Columbia Centre for Disease Control	Canada	EAI	PRJNA413593	S	S	S	n/a	S	S	S	S
12s127	British Columbia Centre for Disease Control	Canada	Ural	PRJNA413593	S	S	S	n/a	S	S	S	S
A7s264	British Columbia Centre for Disease Control	Canada	Beijing	PRJNA413593	S	S	S	n/a	S	S	S	S
10s105	British Columbia Centre for Disease Control	Canada	EAI	PRJNA413593	R	S	S	S	R	S	S	S
13s488	British Columbia Centre for Disease Control	Canada	Delhi	PRJNA413593	S	S	S	n/a	S	S	S	S
10s207	British Columbia Centre for Disease Control	Canada	Delhi	PRJNA413593	S	S	S	n/a	S	S	S	S
13s356	British Columbia Centre for Disease Control	Canada	Delhi	PRJNA413593	S	S	S	n/a	U	S	S	S
13s314	British Columbia Centre for Disease Control	Canada	lineage4	PRJNA413593	S	S	S	n/a	R	S	S	S
13s212	British Columbia Centre for Disease Control	Canada	EAI	PRJNA413593	R	S	S	S	R	S	S	S
A5s153	British Columbia Centre for Disease Control	Canada	S-type	PRJNA413593	S	S	S	n/a	S	U	S	S
A5s054	British Columbia Centre for Disease Control	Canada	EAI	PRJNA413593	R	S	S	S	R	S	S	S
A7s343	British Columbia Centre for Disease Control	Canada	EAI	PRJNA413593	S	S	S	n/a	U	S	S	S
13s499	British Columbia Centre for Disease Control	Canada	Ural	PRJNA413593	S	S	S	n/a	S	S	S	S
12s348	British Columbia Centre for Disease Control	Canada	Delhi	PRJNA413593	S	S	S	n/a	S	S	S	S
11s072	British Columbia Centre for Disease Control	Canada	EAI	PRJNA413593	S	S	S	n/a	S	S	S	S
11s283	British Columbia Centre for Disease Control	Canada	Delhi	PRJNA413593	S	S	S	n/a	S	S	S	S
A9s203	British Columbia Centre for Disease Control	Canada	EAI	PRJNA413593	S	S	S	n/a	S	S	S	S
A6s165	British Columbia Centre for Disease Control	Canada	Delhi	PRJNA413593	S	S	S	n/a	S	S	S	S
14s384	British Columbia Centre for Disease Control	Canada	lineage4	PRJNA413593	S	S	S	n/a	R	S	S	S
A6s193	British Columbia Centre for Disease Control	Canada	Beijing	PRJNA413593	S	S	S	n/a	S	S	S	S
14s004	British Columbia Centre for Disease Control	Canada	Haarlem	PRJNA413593	S	S	S	n/a	S	S	S	S
A7s124	British Columbia Centre for Disease Control	Canada	Beijing	PRJNA413593	S	S	S	n/a	S	S	S	S
14s213	British Columbia Centre for Disease Control	Canada	Ural	PRJNA413593	S	S	S	n/a	S	S	S	S
A7s351	British Columbia Centre for Disease Control	Canada	Delhi	PRJNA413593	S	S	S	n/a	S	S	S	S
A9s214	British Columbia Centre for Disease Control	Canada	Ural	PRJNA413593	S	S	S	n/a	S	S	S	S
A9s223	British Columbia Centre for Disease Control	Canada	lineage4	PRJNA413593	S	S	S	n/a	S	S	S	S
12s415	British Columbia Centre for Disease Control	Canada	Beijing	PRJNA413593	R	S	S	S	R	S	S	S
A6s053	British Columbia Centre for Disease Control	Canada	Delhi	PRJNA413593	S	S	S	n/a	S	S	S	S
10s401	British Columbia Centre for Disease Control	Canada	EAI	PRJNA413593	S	S	S	n/a	S	S	S	S
A9s137	British Columbia Centre for Disease Control	Canada	Beijing	PRJNA413593	S	S	S	n/a	S	S	S	S
A6s126	British Columbia Centre for Disease Control	Canada	Beijing	PRJNA413593	S	S	S	n/a	S	S	S	S
A5s083	British Columbia Centre for Disease Control	Canada	EAI	PRJNA413593	S	S	S	n/a	S	S	S	R
A9s164	British Columbia Centre for Disease Control	Canada	EAI	PRJNA413593	S	S	S	n/a	S	S	S	S
11s171	British Columbia Centre for Disease Control	Canada	Haarlem	PRJNA413593	S	S	S	n/a	S	S	S	S
14s060	British Columbia Centre for Disease Control	Canada	Beijing	PRJNA413593	S	S	S	n/a	S	S	S	S
14s279	British Columbia Centre for Disease Control	Canada	Beijing	PRJNA413593	S	S	S	n/a	S	S	S	S
14s131	British Columbia Centre for Disease Control	Canada	Ural	PRJNA413593	S	S	S	n/a	S	S	S	S
A9s155	British Columbia Centre for Disease Control	Canada	Beijing	PRJNA413593	S	S	S	n/a	S	S	S	S
13s199	British Columbia Centre for Disease Control	Canada	Ural	PRJNA413593	S	S	S	n/a	S	S	S	S
A4s103	British Columbia Centre for Disease Control	Canada	lineage4	PRJNA413593	S	S	S	n/a	S	S	S	S
11s358	British Columbia Centre for Disease Control	Canada	LAM	PRJNA413593	R	S	S	S	R	S	S	S
A9s162	British Columbia Centre for Disease Control	Canada	Beijing	PRJNA413593	S	S	S	n/a	S	S	S	S
10s007	British Columbia Centre for Disease Control	Canada	Ural	PRJNA413593	S	S	S	n/a	S	S	U	S
13s303	British Columbia Centre for Disease Control	Canada	Beijing	PRJNA413593	S	S	S	n/a	U	S	S	S
14s203	British Columbia Centre for Disease Control	Canada	Haarlem	PRJNA413593	S	S	S	n/a	S	S	S	S
A5s098	British Columbia Centre for Disease Control	Canada	Beijing	PRJNA413593	R	S	S	S	R	S	S	S
A5s302	British Columbia Centre for Disease Control	Canada	Ural	PRJNA413593	S	S	S	n/a	S	S	S	S
A9s343	British Columbia Centre for Disease Control	Canada	lineage4	PRJNA413593	S	S	S	n/a	S	S	S	S
A6s133	British Columbia Centre for Disease Control	Canada	Beijing	PRJNA413593	S	S	S	n/a	S	S	S	S
A9s038	British Columbia Centre for Disease Control	Canada	Beijing	PRJNA413593	S	S	S	n/a	S	S	S	S
A7s299	British Columbia Centre for Disease Control	Canada	lineage4	PRJNA413593	S	S	S	n/a	S	S	U	S
A6s178	British Columbia Centre for Disease Control	Canada	Beijing	PRJNA413593	S	S	S	n/a	S	S	S	S
A5s209	British Columbia Centre for Disease Control	Canada	Beijing	PRJNA413593	R	S	S	S	U	S	U	S
10s038	British Columbia Centre for Disease Control	Canada	S-type	PRJNA413593	S	S	S	n/a	S	U	S	S
12s274	British Columbia Centre for Disease Control	Canada	Beijing	PRJNA413593	S	S	S	n/a	S	S	S	S
14s007	British Columbia Centre for Disease Control	Canada	Delhi	PRJNA413593	S	S	S	n/a	S	S	S	S
13s387	British Columbia Centre for Disease Control	Canada	EAI	PRJNA413593	S	S	S	n/a	U	S	S	S
12s271	British Columbia Centre for Disease Control	Canada	Beijing	PRJNA413593	R	S	S	S	S	S	S	S
A4s007	British Columbia Centre for Disease Control	Canada	lineage4	PRJNA413593	S	S	S	n/a	S	S	S	S
11s384	British Columbia Centre for Disease Control	Canada	Ural	PRJNA413593	S	S	S	n/a	S	S	S	S
11s410	British Columbia Centre for Disease Control	Canada	S-type	PRJNA413593	S	S	S	n/a	U	S	S	S
A9s196	British Columbia Centre for Disease Control	Canada	S-type	PRJNA413593	S	S	S	n/a	S	S	S	S
A8s164	British Columbia Centre for Disease Control	Canada	Ural	PRJNA413593	S	S	S	n/a	S	S	S	S

A9s385	British Columbia Centre for Disease Control	Canada	Haarlem	PRJNA413593	S	S	S	n/a	S	S	S	S
14s467	British Columbia Centre for Disease Control	Canada	lineage4	PRJNA413593	R	S	S	S	R	S	S	S
11s442	British Columbia Centre for Disease Control	Canada	LAM	PRJNA413593	S	S	S	n/a	S	S	S	S
14s071	British Columbia Centre for Disease Control	Canada	Haarlem	PRJNA413593	S	S	S	n/a	S	S	S	S
A6s168	British Columbia Centre for Disease Control	Canada	Delhi	PRJNA413593	S	R	S	n/a	S	R	S	S
A9s045	British Columbia Centre for Disease Control	Canada	Haarlem	PRJNA413593	S	S	S	n/a	S	S	S	S
A6s163	British Columbia Centre for Disease Control	Canada	S-type	PRJNA413593	S	S	S	n/a	S	S	S	S
13s154	British Columbia Centre for Disease Control	Canada	S-type	PRJNA413593	S	S	S	n/a	S	U	S	S
14s315	British Columbia Centre for Disease Control	Canada	EAI	PRJNA413593	S	S	S	n/a	S	S	S	S
A8s021	British Columbia Centre for Disease Control	Canada	lineage4	PRJNA413593	S	S	R	n/a	S	S	S	S
12s218	British Columbia Centre for Disease Control	Canada	X-type	PRJNA413593	S	S	S	n/a	S	S	S	S
13s266	British Columbia Centre for Disease Control	Canada	Haarlem	PRJNA413593	S	S	S	n/a	S	S	S	S
A6s320	British Columbia Centre for Disease Control	Canada	Ural	PRJNA413593	S	S	S	n/a	S	S	S	S
A7s067	British Columbia Centre for Disease Control	Canada	Beijing	PRJNA413593	S	S	S	n/a	S	S	S	S
A5s164	British Columbia Centre for Disease Control	Canada	S-type	PRJNA413593	S	S	S	n/a	S	U	S	S
A8s289	British Columbia Centre for Disease Control	Canada	EAI	PRJNA413593	S	S	S	n/a	S	S	S	S
A7s347	British Columbia Centre for Disease Control	Canada	Beijing	PRJNA413593	S	S	S	n/a	S	S	S	S
12s346	British Columbia Centre for Disease Control	Canada	S-type	PRJNA413593	S	S	S	n/a	S	U	S	S
12s058	British Columbia Centre for Disease Control	Canada	Delhi	PRJNA413593	S	S	S	n/a	S	S	S	S
14s355	British Columbia Centre for Disease Control	Canada	EAI	PRJNA413593	S	S	S	n/a	S	S	S	S
14s256	British Columbia Centre for Disease Control	Canada	Haarlem	PRJNA413593	S	S	S	n/a	S	S	S	S
A6s270	British Columbia Centre for Disease Control	Canada	Haarlem	PRJNA413593	S	S	S	n/a	S	S	S	S
13s158	British Columbia Centre for Disease Control	Canada	Haarlem	PRJNA413593	S	S	S	n/a	S	S	S	S
14s162	British Columbia Centre for Disease Control	Canada	lineage4	PRJNA413593	S	S	S	n/a	S	S	S	S
14s302	British Columbia Centre for Disease Control	Canada	EAI	PRJNA413593	R	S	S	S	R	S	S	S
A8s303	British Columbia Centre for Disease Control	Canada	S-type	PRJNA413593	S	S	S	n/a	S	U	S	S
11s354	British Columbia Centre for Disease Control	Canada	S-type	PRJNA413593	S	S	S	n/a	S	U	S	S
14s242	British Columbia Centre for Disease Control	Canada	EAI	PRJNA413593	R	S	S	S	R	S	S	S
A7s291	British Columbia Centre for Disease Control	Canada	Beijing	PRJNA413593	S	S	S	n/a	S	S	S	S
A4s048	British Columbia Centre for Disease Control	Canada	X-type	PRJNA413593	S	S	S	n/a	S	S	S	S
A5s035	British Columbia Centre for Disease Control	Canada	Beijing	PRJNA413593	R	R	R	R	R	R	R	U
A8s302	British Columbia Centre for Disease Control	Canada	S-type	PRJNA413593	S	S	S	n/a	S	U	S	S
A7s302	British Columbia Centre for Disease Control	Canada	lineage4	PRJNA413593	R	S	S	n/a	R	S	S	S
13s132	British Columbia Centre for Disease Control	Canada	EAI	PRJNA413593	R	S	S	S	R	S	S	S
13s057	British Columbia Centre for Disease Control	Canada	EAI	PRJNA413593	S	S	S	n/a	S	S	S	S
11s394	British Columbia Centre for Disease Control	Canada	Delhi	PRJNA413593	S	S	S	n/a	S	S	S	S
A9s005	British Columbia Centre for Disease Control	Canada	S-type	PRJNA413593	S	S	S	n/a	S	U	S	S
A5s130	British Columbia Centre for Disease Control	Canada	Beijing	PRJNA413593	S	S	S	n/a	S	S	S	S
12s438r	British Columbia Centre for Disease Control	Canada	EAI	PRJNA413593	S	S	S	n/a	S	S	S	S
A7s352	British Columbia Centre for Disease Control	Canada	Beijing	PRJNA413593	S	S	S	n/a	S	S	S	S
A7s044	British Columbia Centre for Disease Control	Canada	Delhi	PRJNA413593	S	S	S	n/a	S	S	S	S
A8s276	British Columbia Centre for Disease Control	Canada	Beijing	PRJNA413593	R	S	S	n/a	R	S	S	S
A7s131	British Columbia Centre for Disease Control	Canada	S-type	PRJNA413593	S	S	S	n/a	S	U	S	S
A9s194	British Columbia Centre for Disease Control	Canada	Haarlem	PRJNA413593	S	S	S	n/a	S	S	S	S
14s487	British Columbia Centre for Disease Control	Canada	EAI	PRJNA413593	S	S	S	n/a	S	S	S	S
A6s417	British Columbia Centre for Disease Control	Canada	S-type	PRJNA413593	S	S	S	n/a	S	S	R	S
12s437r	British Columbia Centre for Disease Control	Canada	LAM	PRJNA413593	S	S	S	n/a	S	S	S	S
12s185	British Columbia Centre for Disease Control	Canada	lineage4	PRJNA413593	S	S	S	n/a	S	S	S	S
A6s286	British Columbia Centre for Disease Control	Canada	Beijing	PRJNA413593	S	S	S	n/a	S	S	S	S
A8s072	British Columbia Centre for Disease Control	Canada	S-type	PRJNA413593	S	S	S	n/a	S	S	U	S
A5s008	British Columbia Centre for Disease Control	Canada	EAI	PRJNA413593	S	S	S	n/a	S	S	S	S
A8s327	British Columbia Centre for Disease Control	Canada	Haarlem	PRJNA413593	S	S	S	n/a	S	S	S	S
11s172	British Columbia Centre for Disease Control	Canada	Beijing	PRJNA413593	S	S	S	n/a	S	S	S	S
A7s214	British Columbia Centre for Disease Control	Canada	Beijing	PRJNA413593	R	S	S	n/a	R	S	S	S
A8s304	British Columbia Centre for Disease Control	Canada	EAI	PRJNA413593	S	S	S	n/a	S	S	S	S
A8s049	British Columbia Centre for Disease Control	Canada	EAI	PRJNA413593	S	S	S	n/a	S	S	S	S
A9s100	British Columbia Centre for Disease Control	Canada	Beijing	PRJNA413593	S	S	S	n/a	S	S	S	S
11s151	British Columbia Centre for Disease Control	Canada	EAI	PRJNA413593	S	S	S	n/a	S	S	S	S
A9s391	British Columbia Centre for Disease Control	Canada	Haarlem	PRJNA413593	S	S	S	n/a	S	S	S	S
A6s031	British Columbia Centre for Disease Control	Canada	EAI	PRJNA413593	S	S	S	n/a	S	S	S	S
A8s359	British Columbia Centre for Disease Control	Canada	EAI	PRJNA413593	S	S	S	n/a	S	S	S	S
11s182	British Columbia Centre for Disease Control	Canada	Beijing	PRJNA413593	S	S	S	n/a	S	S	S	S
A6s157	British Columbia Centre for Disease Control	Canada	lineage4	PRJNA413593	S	S	S	n/a	S	S	S	S
A3s176	British Columbia Centre for Disease Control	Canada	EAI	PRJNA413593	R	S	S	n/a	R	S	S	S
A5s011	British Columbia Centre for Disease Control	Canada	Beijing	PRJNA413593	S	S	S	n/a	S	S	S	S
A6s015	British Columbia Centre for Disease Control	Canada	Ural	PRJNA413593	S	S	S	n/a	S	S	S	S
A5s282	British Columbia Centre for Disease Control	Canada	S-type	PRJNA413593	R	S	S	S	U	S	S	S
A8s231	British Columbia Centre for Disease Control	Canada	Beijing	PRJNA413593	S	S	S	n/a	S	S	S	S
11s453	British Columbia Centre for Disease Control	Canada	Beijing	PRJNA413593	R	S	S	S	R	S	S	S
A4s023	British Columbia Centre for Disease Control	Canada	Beijing	PRJNA413593	S	S	S	n/a	S	S	U	S
A6s008	British Columbia Centre for Disease Control	Canada	Ural	PRJNA413593	S	S	S	n/a	S	S	S	S
14s286	British Columbia Centre for Disease Control	Canada	EAI	PRJNA413593	R	S	S	S	R	S	U	S
A6s169	British Columbia Centre for Disease Control	Canada	Haarlem	PRJNA413593	S	S	S	n/a	S	S	S	S
A7s125	British Columbia Centre for Disease Control	Canada	Beijing	PRJNA413593	S	S	S	n/a	S	S	S	S
A8s226	British Columbia Centre for Disease Control	Canada	LAM	PRJNA413593	S	S	S	n/a	S	S	S	S
12s263	British Columbia Centre for Disease Control	Canada	Ural	PRJNA413593	S	S	S	n/a	S	S	S	S
A7s290	British Columbia Centre for Disease Control	Canada	EAI	PRJNA413593	S	S	S	n/a	S	S	S	S
A4s083	British Columbia Centre for Disease Control	Canada	Beijing	PRJNA413593	S	S	S	n/a	S	S	S	S
12s310	British Columbia Centre for Disease Control	Canada	Beijing	PRJNA413593	S	S	S	n/a	S	S	S	S
14s128	British Columbia Centre for Disease Control	Canada	Beijing	PRJNA413593	S	S	S	S	S	S	S	S
10s438	British Columbia Centre for Disease Control	Canada	lineage4	PRJNA413593	S	S	S	n/a	S	S	S	S
A8s317	British Columbia Centre for Disease Control	Canada	Beijing	PRJNA413593	S	S	S	n/a	S	S	S	S
A6s346	British Columbia Centre for Disease Control	Canada	Beijing	PRJNA413593	S	S	S	n/a	S	S	S	S
A7s024	British Columbia Centre for Disease Control	Canada	Haarlem	PRJNA413593	S	S	S	n/a	S	S	S	S
A8s208	British Columbia Centre for Disease Control	Canada	Beijing	PRJNA413593	S	S	S	n/a	S	S	S	S
A8s130	British Columbia Centre for Disease Control	Canada	EAI	PRJNA413593	S	S	S	n/a	S	S	S	S
A6s256	British Columbia Centre for Disease Control	Canada	LAM	PRJNA413593	S	S	S	n/a	S	S	S	S
A9s396	British Columbia Centre for Disease Control	Canada	lineage4	PRJNA413593	S	S	S	n/a	S	S	S	S
11s015	British Columbia Centre for Disease Control	Canada	Beijing	PRJNA413593	S	S	S	n/a	S	S	S	S
A7s202	British Columbia Centre for Disease Control	Canada	EAI	PRJNA413593	S	S	S	n/a	S	S	S	S
A9s224	British Columbia Centre for Disease Control	Canada	Beijing	PRJNA413593	S	S	S	n/a	S	S	S	S
A7s300	British Columbia Centre for Disease Control	Canada	Delhi	PRJNA413593	S	S	S	n/a	S	S	S	S
A7s097	British Columbia Centre for Disease Control	Canada	Beijing	PRJNA413593	R	S	R	n/a	R	F	S	S
12s091	British Columbia Centre for Disease Control	Canada	EAI	PRJNA413593	S	S	S	n/a	S	S	S	S
12s092	British Columbia Centre for Disease Control	Canada	Delhi	PRJNA413593	S	S	S	n/a	S	S	S	S
14s365	British Columbia Centre for Disease Control	Canada	Beijing	PRJNA413593	S	S	S	n/a	S	S	S	S
11s324	British Columbia Centre for Disease Control	Canada	EAI	PRJNA413593	S	S	S	n/a	S	S	S	S
A7s033	British Columbia Centre for Disease Control	Canada	EAI	PRJNA413593	R	S	S	n/a	R	S	S	S
A9s111	British Columbia Centre for Disease Control	Canada	Beijing	PRJNA413593	S	S	S	n/a	S	S	S	S
13s436	British Columbia Centre for Disease Control	Canada	Beijing	PRJNA413593	S	S	S	n/a	S	U	S	S
A8s328	British Columbia Centre for Disease Control	Canada	Ural	PRJNA413593	S	S	S	n/a	S	S	S	S
14s517	British Columbia Centre for Disease Control	Canada	Beijing	PRJNA413593	R	S	S	S	R	S	S	S
10s227	British Columbia Centre for Disease Control	Canada	Haarlem	PRJNA413593	S	S	S	n/a	S	S	S	S
A9s116	British Columbia Centre for Disease Control	Canada	lineage4	PRJNA413593	S	S	S	n/a	S	S	S	S
A5s271	British Columbia Centre for Disease Control	Canada	Haarlem	PRJNA413593	S	S	S	n/a	S	S	S	S
A9s049	British Columbia Centre for Disease Control	Canada	lineage4	PRJNA413593	S	S	S	n/a	S	S	S	S
12s277	British Columbia Centre for Disease Control	Canada	Haarlem	PRJNA413593	S	S	S	n/a	S	S	U	S
14s253	British Columbia Centre for Disease Control	Canada	Haarlem	PRJNA413593	S	S	S	n/a	S	S	S	S
A7s357	British Columbia Centre for Disease Control	Canada	Beijing	PRJNA413593	S	S	S	n/a	S	S	S	S
A6s018	British Columbia Centre for Disease Control	Canada	Ural	PRJNA413593	S	S	S	n/a	S	S	S	S
A9s047	British Columbia Centre for Disease Control	Canada	S-type	PRJNA413593	S	S	S	n/a	S	S	S	S
10s011	British Columbia Centre for Disease Control	Canada	lineage4	PRJNA413593	S	S	S	n/a	S	S	S	S
10s425	British Columbia Centre for Disease Control	Canada	Haarlem	PRJNA413593	S	S	S	n/a	S	S	S	S
A7s240	British Columbia Centre for Disease Control	Canada	Delhi	PRJNA413593	S	S	S	n/a	S	S	S	S
13s302	British Columbia Centre for Disease Control	Canada	EAI	PRJNA413593	S	S	S	n/a	S	S	S	S
13s306	British Columbia Centre for Disease Control	Canada	Beijing	PRJNA413593	S	S	S	n/a	S	S	S	S

A7s238	British Columbia Centre for Disease Control	Canada	Haarlem	PRJNA413593	S	S	S	n/a	S	S	S	S
A9s043	British Columbia Centre for Disease Control	Canada	lineage4	PRJNA413593	S	S	S	n/a	R	S	S	S
A5s179	British Columbia Centre for Disease Control	Canada	LAM	PRJNA413593	S	S	S	n/a	S	S	S	S
10s201	British Columbia Centre for Disease Control	Canada	Delhi	PRJNA413593	S	S	S	n/a	S	S	S	S
A7s306	British Columbia Centre for Disease Control	Canada	EAI	PRJNA413593	S	S	S	n/a	S	S	S	S
14s237	British Columbia Centre for Disease Control	Canada	EAI	PRJNA413593	S	S	S	n/a	S	S	S	S
13s503	British Columbia Centre for Disease Control	Canada	S-type	PRJNA413593	S	S	S	n/a	S	S	S	S
12s089	British Columbia Centre for Disease Control	Canada	S-type	PRJNA413593	S	S	S	n/a	S	U	S	S
13s407	British Columbia Centre for Disease Control	Canada	Delhi	PRJNA413593	S	S	S	n/a	S	S	S	S
14s262	British Columbia Centre for Disease Control	Canada	Beijing	PRJNA413593	S	S	S	n/a	S	S	S	S
13s010	British Columbia Centre for Disease Control	Canada	S-type	PRJNA413593	S	S	S	n/a	S	U	S	S
A8s306	British Columbia Centre for Disease Control	Canada	LAM	PRJNA413593	S	S	S	n/a	S	S	S	S
12s121	British Columbia Centre for Disease Control	Canada	Haarlem	PRJNA413593	S	S	S	n/a	S	S	S	S
A8s170	British Columbia Centre for Disease Control	Canada	Haarlem	PRJNA413593	S	S	S	n/a	S	S	S	S
14s134	British Columbia Centre for Disease Control	Canada	Delhi	PRJNA413593	S	R	S	S	S	R	S	S
A7s305	British Columbia Centre for Disease Control	Canada	S-type	PRJNA413593	S	S	S	n/a	S	S	S	S
A5s154	British Columbia Centre for Disease Control	Canada	lineage4	PRJNA413593	S	S	S	n/a	S	S	S	S
A5s018	British Columbia Centre for Disease Control	Canada	Beijing	PRJNA413593	S	S	S	n/a	S	S	S	S
14s147	British Columbia Centre for Disease Control	Canada	S-type	PRJNA413593	S	S	S	n/a	S	S	S	S
A4s082	British Columbia Centre for Disease Control	Canada	Beijing	PRJNA413593	S	S	S	n/a	S	S	S	S
14s036	British Columbia Centre for Disease Control	Canada	lineage4	PRJNA413593	R	S	S	S	R	S	S	S
14s390	British Columbia Centre for Disease Control	Canada	EAI	PRJNA413593	S	S	S	n/a	S	S	S	S
A8s142	British Columbia Centre for Disease Control	Canada	S-type	PRJNA413593	S	S	S	n/a	S	S	U	S
A8s137	British Columbia Centre for Disease Control	Canada	Haarlem	PRJNA413593	S	S	S	n/a	S	S	S	S
13s217	British Columbia Centre for Disease Control	Canada	EAI	PRJNA413593	R	S	S	S	R	S	S	S
10s169	British Columbia Centre for Disease Control	Canada	X-type	PRJNA413593	S	S	S	n/a	S	S	S	S
A6s394	British Columbia Centre for Disease Control	Canada	S-type	PRJNA413593	S	S	S	n/a	S	S	S	S
A0s090	British Columbia Centre for Disease Control	Canada	S-type	PRJNA413593	S	S	S	n/a	S	U	S	S
12s365	British Columbia Centre for Disease Control	Canada	Beijing	PRJNA413593	S	S	S	n/a	S	S	S	S
A9s303	British Columbia Centre for Disease Control	Canada	lineage4	PRJNA413593	S	S	S	n/a	S	S	U	S
13s501	British Columbia Centre for Disease Control	Canada	Ural	PRJNA413593	S	S	S	n/a	S	S	S	S
14s091	British Columbia Centre for Disease Control	Canada	Haarlem	PRJNA413593	S	S	S	n/a	S	S	S	S
10s421	British Columbia Centre for Disease Control	Canada	Beijing	PRJNA413593	S	S	S	n/a	S	S	S	S
A6s426	British Columbia Centre for Disease Control	Canada	Beijing	PRJNA413593	S	S	S	n/a	S	S	S	S
A5s283	British Columbia Centre for Disease Control	Canada	lineage4	PRJNA413593	S	S	S	n/a	S	S	S	S
14s164	British Columbia Centre for Disease Control	Canada	EAI	PRJNA413593	R	S	S	S	R	S	S	S
A7s348	British Columbia Centre for Disease Control	Canada	Ural	PRJNA413593	S	S	S	n/a	S	S	S	S
14s426	British Columbia Centre for Disease Control	Canada	Delhi	PRJNA413593	S	S	S	n/a	S	S	S	S
A6s437	British Columbia Centre for Disease Control	Canada	lineage4	PRJNA413593	S	S	S	n/a	S	S	S	S
14s488	British Columbia Centre for Disease Control	Canada	EAI	PRJNA413593	S	S	S	n/a	S	S	S	S
12s353	British Columbia Centre for Disease Control	Canada	EAI	PRJNA413593	S	S	S	S	S	S	S	S
A6s058	British Columbia Centre for Disease Control	Canada	lineage4	PRJNA413593	R	S	S	S	R	S	S	S
10s137	British Columbia Centre for Disease Control	Canada	Beijing	PRJNA413593	S	S	S	n/a	S	S	S	S
10s044	British Columbia Centre for Disease Control	Canada	Beijing	PRJNA413593	S	S	S	n/a	S	S	S	S
13s001	British Columbia Centre for Disease Control	Canada	LAM	PRJNA413593	S	S	S	n/a	S	S	S	S
10s279	British Columbia Centre for Disease Control	Canada	Ural	PRJNA413593	S	S	S	n/a	S	S	S	S
10s303	British Columbia Centre for Disease Control	Canada	lineage4	PRJNA413593	R	S	S	S	R	S	S	S
A9s088	British Columbia Centre for Disease Control	Canada	EAI	PRJNA413593	S	S	S	n/a	S	S	S	S
A8s012	British Columbia Centre for Disease Control	Canada	S-type	PRJNA413593	S	S	S	n/a	S	U	S	S
13s191	British Columbia Centre for Disease Control	Canada	lineage4	PRJNA413593	R	S	S	S	R	S	S	S
A9s273	British Columbia Centre for Disease Control	Canada	LAM	PRJNA413593	S	S	S	n/a	S	S	S	S
A9s386	British Columbia Centre for Disease Control	Canada	lineage4	PRJNA413593	R	S	S	S	R	S	S	S
12s276	British Columbia Centre for Disease Control	Canada	S-type	PRJNA413593	S	S	S	n/a	S	S	S	S
A8s018	British Columbia Centre for Disease Control	Canada	Beijing	PRJNA413593	S	S	S	n/a	S	S	S	S
10s194	British Columbia Centre for Disease Control	Canada	EAI	PRJNA413593	S	S	S	n/a	S	S	S	S
13s051	British Columbia Centre for Disease Control	Canada	EAI	PRJNA413593	S	S	S	S	S	S	S	S
A8s091	British Columbia Centre for Disease Control	Canada	Beijing	PRJNA413593	R	S	S	n/a	R	S	S	S
A3s232	British Columbia Centre for Disease Control	Canada	S-type	PRJNA413593	S	S	S	n/a	S	S	S	S
10s069	British Columbia Centre for Disease Control	Canada	Beijing	PRJNA413593	S	S	S	n/a	S	S	S	S
A9s177	British Columbia Centre for Disease Control	Canada	Ural	PRJNA413593	S	S	S	n/a	S	S	S	S
10s045	British Columbia Centre for Disease Control	Canada	Delhi	PRJNA413593	S	S	S	n/a	S	S	S	S
10s337	British Columbia Centre for Disease Control	Canada	Beijing	PRJNA413593	S	S	S	n/a	S	S	U	S
10s381	British Columbia Centre for Disease Control	Canada	Beijing	PRJNA413593	S	S	S	n/a	S	S	S	S
A6s297	British Columbia Centre for Disease Control	Canada	S-type	PRJNA413593	S	S	S	n/a	S	S	S	S
A9s058	British Columbia Centre for Disease Control	Canada	S-type	PRJNA413593	S	S	S	n/a	S	U	S	S
A6s231	British Columbia Centre for Disease Control	Canada	EAI	PRJNA413593	S	S	S	n/a	S	S	S	S
A7s026	British Columbia Centre for Disease Control	Canada	EAI	PRJNA413593	S	S	S	n/a	S	S	S	S
11s413	British Columbia Centre for Disease Control	Canada	Haarlem	PRJNA413593	S	S	S	n/a	S	S	U	S
A9s027	British Columbia Centre for Disease Control	Canada	lineage4	PRJNA413593	S	S	S	n/a	S	S	S	S
A6s360	British Columbia Centre for Disease Control	Canada	lineage4	PRJNA413593	S	S	S	n/a	S	S	S	S
12s068	British Columbia Centre for Disease Control	Canada	EAI	PRJNA413593	S	S	S	n/a	S	S	S	S
12s327	British Columbia Centre for Disease Control	Canada	Haarlem	PRJNA413593	S	S	S	n/a	S	S	S	S
10s314	British Columbia Centre for Disease Control	Canada	Delhi	PRJNA413593	S	S	S	n/a	U	S	S	S
14s058	British Columbia Centre for Disease Control	Canada	EAI	PRJNA413593	S	S	S	n/a	S	S	S	S
A8s294	British Columbia Centre for Disease Control	Canada	lineage4	PRJNA413593	S	S	S	n/a	R	S	S	S
10s177	British Columbia Centre for Disease Control	Canada	lineage4	PRJNA413593	R	S	S	S	R	S	S	S
A9s105	British Columbia Centre for Disease Control	Canada	Haarlem	PRJNA413593	S	S	S	n/a	S	S	S	S
13s110	British Columbia Centre for Disease Control	Canada	Delhi	PRJNA413593	S	S	S	n/a	S	S	U	S
A8s118	British Columbia Centre for Disease Control	Canada	Delhi	PRJNA413593	S	S	S	n/a	S	S	U	S
A5s044	British Columbia Centre for Disease Control	Canada	Beijing	PRJNA413593	R	S	S	S	R	S	U	S
13s197	British Columbia Centre for Disease Control	Canada	Beijing	PRJNA413593	S	S	S	n/a	S	S	S	S
13s278	British Columbia Centre for Disease Control	Canada	EAI	PRJNA413593	R	S	S	S	R	S	S	S
A9s167	British Columbia Centre for Disease Control	Canada	Beijing	PRJNA413593	S	S	S	n/a	S	S	S	S
13s003	British Columbia Centre for Disease Control	Canada	Delhi	PRJNA413593	S	S	S	n/a	S	S	S	S
12s152	British Columbia Centre for Disease Control	Canada	EAI	PRJNA413593	S	S	S	n/a	U	S	S	S
A9s247	British Columbia Centre for Disease Control	Canada	lineage4	PRJNA413593	S	S	S	n/a	S	S	S	S
A4s354	British Columbia Centre for Disease Control	Canada	Ural	PRJNA413593	S	S	S	n/a	S	S	S	S
A5s188	British Columbia Centre for Disease Control	Canada	EAI	PRJNA413593	R	S	R	S	R	S	R	S
A2s338	British Columbia Centre for Disease Control	Canada	Delhi	PRJNA413593	R	S	S	n/a	R	S	S	S
A8s313	British Columbia Centre for Disease Control	Canada	Ural	PRJNA413593	S	S	S	n/a	S	F	S	S
A8s094	British Columbia Centre for Disease Control	Canada	EAI	PRJNA413593	S	S	S	n/a	S	S	S	S
13s408	British Columbia Centre for Disease Control	Canada	S-type	PRJNA413593	S	S	S	n/a	S	S	S	S
A5s037	British Columbia Centre for Disease Control	Canada	LAM	PRJNA413593	S	S	R	n/a	S	S	S	S
12s351	British Columbia Centre for Disease Control	Canada	Beijing	PRJNA413593	S	S	S	n/a	S	S	S	S
11s121	British Columbia Centre for Disease Control	Canada	Haarlem	PRJNA413593	S	S	S	n/a	S	S	S	S
12s377	British Columbia Centre for Disease Control	Canada	lineage4	PRJNA413593	S	S	S	n/a	R	S	S	S
A6s091	British Columbia Centre for Disease Control	Canada	S-type	PRJNA413593	S	S	S	n/a	S	S	S	S
13s290	British Columbia Centre for Disease Control	Canada	EAI	PRJNA413593	R	S	S	S	R	S	S	S
12s379	British Columbia Centre for Disease Control	Canada	EAI	PRJNA413593	R	S	S	S	R	S	S	S
A6s447	British Columbia Centre for Disease Control	Canada	Ural	PRJNA413593	S	S	S	n/a	S	S	S	S
14s366	British Columbia Centre for Disease Control	Canada	EAI	PRJNA413593	S	S	S	n/a	S	S	S	S
A7s130	British Columbia Centre for Disease Control	Canada	Beijing	PRJNA413593	S	S	S	n/a	S	S	S	S
A6s345	British Columbia Centre for Disease Control	Canada	Beijing	PRJNA413593	S	S	S	n/a	S	S	S	S
A8s179	British Columbia Centre for Disease Control	Canada	Beijing	PRJNA413593	S	S	S	n/a	S	S	S	S
10s146	British Columbia Centre for Disease Control	Canada	S-type	PRJNA413593	S	S	S	n/a	S	U	S	S
12s285	British Columbia Centre for Disease Control	Canada	Beijing	PRJNA413593	S	S	S	n/a	S	S	S	S
11s206	British Columbia Centre for Disease Control	Canada	Beijing	PRJNA413593	R	S	S	S	R	S	U	S
A8s008	British Columbia Centre for Disease Control	Canada	EAI	PRJNA413593	S	S	S	n/a	S	S	S	S
A8s310	British Columbia Centre for Disease Control	Canada	lineage4	PRJNA413593	S	S	S	n/a	S	S	S	S
14s450	British Columbia Centre for Disease Control	Canada	Haarlem	PRJNA413593	S	S	S	n/a	S	S	S	S
10s124	British Columbia Centre for Disease Control	Canada	lineage4	PRJNA413593	R	S	S	S	R	S	S	S
A4s075	British Columbia Centre for Disease Control	Canada	Ural	PRJNA413593	S	S	S	n/a	S	S	S	S
11s316	British Columbia Centre for Disease Control	Canada	S-type	PRJNA413593	S	S	S	n/a	S	U	S	S
14s003	British Columbia Centre for Disease Control	Canada	Delhi	PRJNA413593	S	S	S	n/a	S	S	S	S
14s097	British Columbia Centre for Disease Control	Canada	Beijing	PRJNA413593	R	S	S	R	F	S	S	S
A8s172	British Columbia Centre for Disease Control	Canada	Beijing	PRJNA413593	R	S	S	n/a	U	S	S	S

A5s198	British Columbia Centre for Disease Control	Canada	Haarlem	PRJNA413593	S	S	S	n/a	S	S	S	S
11s287	British Columbia Centre for Disease Control	Canada	Delhi	PRJNA413593	S	S	S	n/a	S	S	S	S
12s208	British Columbia Centre for Disease Control	Canada	Haarlem	PRJNA413593	S	S	S	n/a	S	S	U	S
A9s107	British Columbia Centre for Disease Control	Canada		PRJNA413593	S	S	S	n/a	S	S	S	S
12s073	British Columbia Centre for Disease Control	Canada	LAM	PRJNA413593	S	S	S	n/a	S	S	S	S
10s410	British Columbia Centre for Disease Control	Canada	lineage4	PRJNA413593	R	S	S	S	R	S	S	S
10s125	British Columbia Centre for Disease Control	Canada	Delhi	PRJNA413593	S	S	S	n/a	S	S	S	S
10s254	British Columbia Centre for Disease Control	Canada	LAM	PRJNA413593	R	S	S	S	R	S	S	S
A6s441	British Columbia Centre for Disease Control	Canada	Delhi	PRJNA413593	S	S	S	n/a	S	S	S	S
A5s236	British Columbia Centre for Disease Control	Canada	lineage4	PRJNA413593	S	S	S	n/a	S	S	S	S
A5s277	British Columbia Centre for Disease Control	Canada	Beijing	PRJNA413593	S	S	S	n/a	S	S	S	S
14s428	British Columbia Centre for Disease Control	Canada	lineage4	PRJNA413593	R	S	S	S	R	S	S	S
A9s307	British Columbia Centre for Disease Control	Canada	Beijing	PRJNA413593	S	S	S	n/a	S	S	S	S
11s289	British Columbia Centre for Disease Control	Canada	Delhi	PRJNA413593	S	S	S	n/a	S	S	S	S
13s485	British Columbia Centre for Disease Control	Canada	EAI	PRJNA413593	R	S	S	S	R	S	S	S
A8s183	British Columbia Centre for Disease Control	Canada	Delhi	PRJNA413593	S	S	S	n/a	S	S	S	S
A2s269r	British Columbia Centre for Disease Control	Canada	S-type	PRJNA413593	S	S	S	n/a	S	U	S	S
A6s412	British Columbia Centre for Disease Control	Canada	Ural	PRJNA413593	S	S	S	n/a	S	S	S	S
12s337	British Columbia Centre for Disease Control	Canada	EAI	PRJNA413593	S	S	S	n/a	S	S	S	S
A9s278	British Columbia Centre for Disease Control	Canada	Haarlem	PRJNA413593	S	S	S	n/a	S	S	S	S
13s021	British Columbia Centre for Disease Control	Canada	lineage4	PRJNA413593	S	S	S	n/a	S	S	U	S
A6s408	British Columbia Centre for Disease Control	Canada	Ural	PRJNA413593	S	S	S	n/a	S	S	S	S
11s458	British Columbia Centre for Disease Control	Canada	Beijing	PRJNA413593	S	S	S	n/a	S	S	S	S
12s021	British Columbia Centre for Disease Control	Canada	Beijing	PRJNA413593	S	S	S	n/a	S	S	S	S
12s397	British Columbia Centre for Disease Control	Canada	EAI	PRJNA413593	S	S	S	n/a	S	S	S	S
10s271	British Columbia Centre for Disease Control	Canada	Beijing	PRJNA413593	S	S	S	n/a	S	S	S	S
A1s399	British Columbia Centre for Disease Control	Canada	Ural	PRJNA413593	S	S	S	n/a	S	S	S	S
14s214	British Columbia Centre for Disease Control	Canada	Beijing	PRJNA413593	R	R	S	S	R	R	S	S
A8s077	British Columbia Centre for Disease Control	Canada	Delhi	PRJNA413593	R	S	S	S	R	S	S	S
A7S019	British Columbia Centre for Disease Control	Canada	Beijing	PRJNA413593	R	S	R	S	R	S	R	S
A7s048	British Columbia Centre for Disease Control	Canada	Beijing	PRJNA413593	S	S	S	n/a	S	S	U	S
A5s136	British Columbia Centre for Disease Control	Canada	lineage4	PRJNA413593	S	S	S	n/a	S	S	S	S
A5s182	British Columbia Centre for Disease Control	Canada	Ural	PRJNA413593	S	S	S	n/a	S	S	S	S
A6s223	British Columbia Centre for Disease Control	Canada	Ural	PRJNA413593	S	S	S	n/a	S	S	S	S
A9s216	British Columbia Centre for Disease Control	Canada	Haarlem	PRJNA413593	S	S	S	n/a	S	S	S	S
A5s149	British Columbia Centre for Disease Control	Canada	lineage4	PRJNA413593	S	S	S	n/a	S	S	S	S
13s483	British Columbia Centre for Disease Control	Canada	lineage4	PRJNA413593	S	S	S	n/a	S	S	S	S
14s280	British Columbia Centre for Disease Control	Canada	Haarlem	PRJNA413593	S	S	S	n/a	S	S	S	S
11s362	British Columbia Centre for Disease Control	Canada	Beijing	PRJNA413593	S	S	S	n/a	S	S	S	S
A8s204	British Columbia Centre for Disease Control	Canada	Ural	PRJNA413593	S	S	S	n/a	S	S	S	S
10s165	British Columbia Centre for Disease Control	Canada	lineage4	PRJNA413593	R	S	S	S	R	S	S	S
A5s084	British Columbia Centre for Disease Control	Canada	EAI	PRJNA413593	S	S	S	n/a	S	S	S	S
A9s272	British Columbia Centre for Disease Control	Canada	lineage4	PRJNA413593	R	S	S	S	R	S	S	S
11s152	British Columbia Centre for Disease Control	Canada	Beijing	PRJNA413593	S	S	S	n/a	S	S	S	S
14s016	British Columbia Centre for Disease Control	Canada	Delhi	PRJNA413593	S	S	S	n/a	S	S	S	S
12s143	British Columbia Centre for Disease Control	Canada	lineage4	PRJNA413593	S	S	S	n/a	S	S	S	S
A7s282	British Columbia Centre for Disease Control	Canada	EAI	PRJNA413593	S	S	S	n/a	S	S	S	S
A5s269	British Columbia Centre for Disease Control	Canada	Beijing	PRJNA413593	S	S	S	n/a	S	S	S	S
A7s219	British Columbia Centre for Disease Control	Canada	Beijing	PRJNA413593	S	S	S	n/a	S	S	S	S
11s069	British Columbia Centre for Disease Control	Canada	S-type	PRJNA413593	S	S	S	n/a	S	S	S	S
13s354	British Columbia Centre for Disease Control	Canada	Beijing	PRJNA413593	S	S	S	n/a	S	S	S	S
A9s126	British Columbia Centre for Disease Control	Canada	S-type	PRJNA413593	S	S	S	n/a	S	S	S	S
A5s146	British Columbia Centre for Disease Control	Canada	Ural	PRJNA413593	S	S	S	n/a	S	S	S	S
13s012	British Columbia Centre for Disease Control	Canada	Haarlem	PRJNA413593	S	S	S	n/a	S	S	S	S
14s410	British Columbia Centre for Disease Control	Canada	EAI	PRJNA413593	S	S	S	n/a	S	S	S	S
14s362	British Columbia Centre for Disease Control	Canada	Beijing	PRJNA413593	R	S	S	R	R	S	S	R
A4s098	British Columbia Centre for Disease Control	Canada	S-type	PRJNA413593	S	S	S	n/a	U	S	S	S
A9s182	British Columbia Centre for Disease Control	Canada	Beijing	PRJNA413593	S	S	S	n/a	S	S	S	S
13s293	British Columbia Centre for Disease Control	Canada	Beijing	PRJNA413593	S	S	S	n/a	S	S	S	S
A6s002	British Columbia Centre for Disease Control	Canada	Beijing	PRJNA413593	R	R	R	R	R	R	R	R
A6s116	British Columbia Centre for Disease Control	Canada	Beijing	PRJNA413593	S	S	S	n/a	S	S	S	S
12s183	British Columbia Centre for Disease Control	Canada	EAI	PRJNA413593	S	S	S	n/a	S	S	S	S
12s301	British Columbia Centre for Disease Control	Canada	Haarlem	PRJNA413593	S	S	S	n/a	S	S	S	S
A9s198	British Columbia Centre for Disease Control	Canada	EAI	PRJNA413593	S	S	S	n/a	U	S	S	S
A5s210	British Columbia Centre for Disease Control	Canada	Beijing	PRJNA413593	R	R	R	S	R	R	R	S
A9s319	British Columbia Centre for Disease Control	Canada	Ural	PRJNA413593	S	S	S	n/a	S	S	S	S
14s117	British Columbia Centre for Disease Control	Canada	EAI	PRJNA413593	R	R	S	R	R	R	S	S
11s022	British Columbia Centre for Disease Control	Canada	Beijing	PRJNA413593	S	S	S	n/a	S	S	S	S
A6s124	British Columbia Centre for Disease Control	Canada	Beijing	PRJNA413593	S	S	S	n/a	U	S	S	S
12s006	British Columbia Centre for Disease Control	Canada	Ural	PRJNA413593	S	S	S	n/a	S	S	S	S
14s202	British Columbia Centre for Disease Control	Canada	Beijing	PRJNA413593	S	S	S	n/a	S	S	S	S
A9s051	British Columbia Centre for Disease Control	Canada	Beijing	PRJNA413593	R	S	S	n/a	R	S	S	S
11s062	British Columbia Centre for Disease Control	Canada	EAI	PRJNA413593	R	S	S	S	R	S	S	S
A8s045	British Columbia Centre for Disease Control	Canada	Beijing	PRJNA413593	S	S	S	n/a	S	S	S	S
12s380	British Columbia Centre for Disease Control	Canada	EAI	PRJNA413593	S	S	S	n/a	S	S	S	S
A9s389	British Columbia Centre for Disease Control	Canada	EAI	PRJNA413593	S	S	S	n/a	S	U	S	S
A5s121	British Columbia Centre for Disease Control	Canada	Delhi	PRJNA413593	S	S	S	n/a	S	S	S	S
A7s012	British Columbia Centre for Disease Control	Canada	Ural	PRJNA413593	S	S	S	n/a	S	S	S	S
10s047	British Columbia Centre for Disease Control	Canada	S-type	PRJNA413593	S	S	S	S	U	S	S	S
A7s279	British Columbia Centre for Disease Control	Canada	lineage4	PRJNA413593	S	S	S	n/a	S	S	U	S
A7s242	British Columbia Centre for Disease Control	Canada	Delhi	PRJNA413593	S	S	S	n/a	S	S	S	S
A6s132	British Columbia Centre for Disease Control	Canada	EAI	PRJNA413593	S	S	S	n/a	S	S	S	S
14s101	British Columbia Centre for Disease Control	Canada	Beijing	PRJNA413593	S	S	S	n/a	S	S	S	S
A6s162	British Columbia Centre for Disease Control	Canada	Beijing	PRJNA413593	S	S	S	n/a	S	S	S	S
A9s231	British Columbia Centre for Disease Control	Canada	Haarlem	PRJNA413593	S	S	S	n/a	S	S	S	S
A6s123	British Columbia Centre for Disease Control	Canada	LAM	PRJNA413593	S	S	R	n/a	S	S	S	S
13s159	British Columbia Centre for Disease Control	Canada	Delhi	PRJNA413593	S	S	S	n/a	S	S	U	S
A6s118	British Columbia Centre for Disease Control	Canada	Haarlem	PRJNA413593	S	S	S	n/a	S	S	S	S
14s427	British Columbia Centre for Disease Control	Canada	Delhi	PRJNA413593	S	S	S	n/a	S	S	S	S
A8s146	British Columbia Centre for Disease Control	Canada	Ural	PRJNA413593	S	S	S	n/a	S	S	S	S
11s165	British Columbia Centre for Disease Control	Canada	Haarlem	PRJNA413593	S	S	S	n/a	S	S	S	S
A8s089	British Columbia Centre for Disease Control	Canada	EAI	PRJNA413593	S	S	S	n/a	S	S	S	S
A7s099	British Columbia Centre for Disease Control	Canada	S-type	PRJNA413593	S	S	S	n/a	S	U	S	S
A9s320	British Columbia Centre for Disease Control	Canada	Haarlem	PRJNA413593	S	S	S	n/a	S	S	S	S
A9s192	British Columbia Centre for Disease Control	Canada	Ural	PRJNA413593	S	S	S	n/a	S	S	S	S
A7s186	British Columbia Centre for Disease Control	Canada	S-type	PRJNA413593	S	S	S	n/a	S	S	S	S
A5s207	British Columbia Centre for Disease Control	Canada	Ural	PRJNA413593	S	S	S	n/a	S	S	S	S
A6s242	British Columbia Centre for Disease Control	Canada	Ural	PRJNA413593	S	S	S	n/a	S	S	S	S
10s385	British Columbia Centre for Disease Control	Canada	Beijing	PRJNA413593	S	S	S	n/a	S	S	S	S
A6s037	British Columbia Centre for Disease Control	Canada	Ural	PRJNA413593	S	S	S	n/a	S	S	S	S
A8s233	British Columbia Centre for Disease Control	Canada	EAI	PRJNA413593	S	S	S	n/a	S	S	S	S
12s044	British Columbia Centre for Disease Control	Canada	Beijing	PRJNA413593	S	S	S	n/a	S	S	S	S
14s218	British Columbia Centre for Disease Control	Canada	Haarlem	PRJNA413593	S	S	S	n/a	S	S	S	S
10s042	British Columbia Centre for Disease Control	Canada	lineage4	PRJNA413593	S	S	S	n/a	S	S	S	S
14s113	British Columbia Centre for Disease Control	Canada	EAI	PRJNA413593	R	S	S	S	R	S	S	S
A9s266	British Columbia Centre for Disease Control	Canada	Haarlem	PRJNA413593	S	S	S	n/a	S	S	S	S
A6s321	British Columbia Centre for Disease Control	Canada	EAI	PRJNA413593	R	R	S	S	R	R	S	S
A5s071	British Columbia Centre for Disease Control	Canada	Haarlem	PRJNA413593	S	S	S	n/a	S	S	S	S
A3s085	British Columbia Centre for Disease Control	Canada	LAM	PRJNA413593	R	S	S	n/a	U	S	S	S
11s148	British Columbia Centre for Disease Control	Canada	Beijing	PRJNA413593	R	S	S	S	R	S	S	S
12s206	British Columbia Centre for Disease Control	Canada	EAI	PRJNA413593	S	S	S	n/a	S	S	S	S
A9s114	British Columbia Centre for Disease Control	Canada	Beijing	PRJNA413593	S	S	S	n/a	S	S	S	S
12s109	British Columbia Centre for Disease Control	Canada	lineage4	PRJNA413593	S	S	S	n/a	S	S	S	S
12s052	British Columbia Centre for Disease Control	Canada	lineage4	PRJNA413593	R	S	S	S	R	S	S	S
12s439r	British Columbia Centre for Disease Control	Canada	lineage4	PRJNA413593	S	S	S	n/a	R	S	S	S
14s068	British Columbia Centre for Disease Control	Canada	Delhi	PRJNA413593	R	S	S	S	R	S	S	S

A5s129	British Columbia Centre for Disease Control	Canada	Beijing	PRJNA413593	S	S	S	n/a	S	S	S	S
10s138	British Columbia Centre for Disease Control	Canada	Beijing	PRJNA413593	S	S	S	n/a	S	S	S	S
A5s007	British Columbia Centre for Disease Control	Canada	Beijing	PRJNA413593	S	S	S	n/a	S	S	S	S
A9s280	British Columbia Centre for Disease Control	Canada	EAI	PRJNA413593	S	S	S	n/a	S	S	S	S
A6s272	British Columbia Centre for Disease Control	Canada	lineage4	PRJNA413593	S	S	S	n/a	S	S	S	S
A7s086	British Columbia Centre for Disease Control	Canada	EAI	PRJNA413593	S	S	R	n/a	S	S	R	S
A8s284	British Columbia Centre for Disease Control	Canada	lineage4	PRJNA413593	S	S	S	n/a	S	S	U	S
10s233	British Columbia Centre for Disease Control	Canada	lineage4	PRJNA413593	S	S	S	n/a	S	S	U	S
10s341	British Columbia Centre for Disease Control	Canada	S-type	PRJNA413593	S	S	S	n/a	S	U	S	S
11s337	British Columbia Centre for Disease Control	Canada	Delhi	PRJNA413593	S	S	S	n/a	S	S	S	S
10s212	British Columbia Centre for Disease Control	Canada	EAI	PRJNA413593	S	S	S	n/a	S	S	S	S
10s237	British Columbia Centre for Disease Control	Canada	Beijing	PRJNA413593	S	S	S	n/a	S	S	S	S
A4s076	British Columbia Centre for Disease Control	Canada	S-type	PRJNA413593	S	S	S	n/a	S	U	S	S
13s205	British Columbia Centre for Disease Control	Canada	Beijing	PRJNA413593	S	S	S	n/a	S	S	S	S
A5s258	British Columbia Centre for Disease Control	Canada	lineage4	PRJNA413593	S	S	S	n/a	S	S	S	S
A7s185	British Columbia Centre for Disease Control	Canada	Ural	PRJNA413593	S	S	S	n/a	S	S	U	S
A9s158	British Columbia Centre for Disease Control	Canada	Beijing	PRJNA413593	R	S	S	n/a	R	S	S	S
10s032	British Columbia Centre for Disease Control	Canada	Delhi	PRJNA413593	S	S	S	n/a	S	S	S	R
14s252	British Columbia Centre for Disease Control	Canada	EAI	PRJNA413593	S	S	S	n/a	S	S	S	S
A5s189	British Columbia Centre for Disease Control	Canada	Haarlem	PRJNA413593	S	S	S	n/a	S	S	S	S
A7s155	British Columbia Centre for Disease Control	Canada	Haarlem	PRJNA413593	S	S	S	n/a	S	S	S	S
10s130	British Columbia Centre for Disease Control	Canada	EAI	PRJNA413593	S	S	S	n/a	S	S	S	S
A5s026	British Columbia Centre for Disease Control	Canada	Ural	PRJNA413593	S	S	S	n/a	S	S	S	S
A8s003	British Columbia Centre for Disease Control	Canada	EAI	PRJNA413593	S	S	S	n/a	S	S	S	S
11s199	British Columbia Centre for Disease Control	Canada	Haarlem	PRJNA413593	S	S	S	n/a	S	S	S	S
11s018	British Columbia Centre for Disease Control	Canada	Delhi	PRJNA413593	S	S	S	n/a	S	S	S	S
10s228	British Columbia Centre for Disease Control	Canada	Delhi	PRJNA413593	S	S	S	n/a	S	S	U	S
11s235	British Columbia Centre for Disease Control	Canada	EAI	PRJNA413593	R	S	S	S	R	S	S	S
13s204	British Columbia Centre for Disease Control	Canada	EAI	PRJNA413593	S	S	S	n/a	S	S	S	S
A7s340	British Columbia Centre for Disease Control	Canada	Ural	PRJNA413593	S	S	S	n/a	S	S	S	S
13s207	British Columbia Centre for Disease Control	Canada	EAI	PRJNA413593	S	S	S	n/a	S	S	S	S
A4s096	British Columbia Centre for Disease Control	Canada	Ural	PRJNA413593	S	S	S	n/a	S	S	S	S
11s263	British Columbia Centre for Disease Control	Canada	Ural	PRJNA413593	S	S	S	n/a	S	S	S	S
A8s020	British Columbia Centre for Disease Control	Canada	S-type	PRJNA413593	S	S	S	n/a	S	S	U	S
A9s233	British Columbia Centre for Disease Control	Canada	lineage4	PRJNA413593	R	S	S	n/a	R	S	S	S
14s207	British Columbia Centre for Disease Control	Canada	Tur	PRJNA413593	R	S	S	S	R	S	R	S
A8s005	British Columbia Centre for Disease Control	Canada	Beijing	PRJNA413593	R	R	R	R	R	R	R	R
A8s278	British Columbia Centre for Disease Control	Canada	EAI	PRJNA413593	S	S	S	n/a	S	S	S	S
A8s300	British Columbia Centre for Disease Control	Canada	LAM	PRJNA413593	S	S	S	n/a	S	S	S	S
14s298	British Columbia Centre for Disease Control	Canada	EAI	PRJNA413593	S	S	S	n/a	S	S	U	S
11s179	British Columbia Centre for Disease Control	Canada	Ural	PRJNA413593	S	S	S	n/a	S	S	U	S
A8s115	British Columbia Centre for Disease Control	Canada	S-type	PRJNA413593	S	S	S	n/a	U	F	S	S
A5s241	British Columbia Centre for Disease Control	Canada	Beijing	PRJNA413593	S	S	S	n/a	S	S	S	S
A9s135	British Columbia Centre for Disease Control	Canada	Beijing	PRJNA413593	S	S	S	n/a	S	S	S	S
A5s147	British Columbia Centre for Disease Control	Canada	Beijing	PRJNA413593	R	R	S	R	R	R	S	R
14s351	British Columbia Centre for Disease Control	Canada	S-type	PRJNA413593	S	S	S	n/a	S	U	S	S
11s374	British Columbia Centre for Disease Control	Canada	lineage4	PRJNA413593	R	S	S	S	R	S	S	S
13s243	British Columbia Centre for Disease Control	Canada	EAI	PRJNA413593	S	S	S	n/a	S	S	S	S
A9s026	British Columbia Centre for Disease Control	Canada	Beijing	PRJNA413593	S	S	S	n/a	S	S	S	S
12s245	British Columbia Centre for Disease Control	Canada	EAI	PRJNA413593	S	S	S	n/a	S	S	S	S
A7s053	British Columbia Centre for Disease Control	Canada	Beijing	PRJNA413593	S	S	S	n/a	S	S	S	S
12s456	British Columbia Centre for Disease Control	Canada	Beijing	PRJNA413593	S	S	S	n/a	S	S	S	S
14s205	British Columbia Centre for Disease Control	Canada	EAI	PRJNA413593	S	S	S	n/a	S	S	S	S
11s341	British Columbia Centre for Disease Control	Canada	Haarlem	PRJNA413593	S	S	S	n/a	S	S	S	S
14s239	British Columbia Centre for Disease Control	Canada	EAI	PRJNA413593	S	S	S	n/a	S	S	S	S
A9s152	British Columbia Centre for Disease Control	Canada	EAI	PRJNA413593	S	S	S	n/a	S	S	S	S
A9s054	British Columbia Centre for Disease Control	Canada	Beijing	PRJNA413593	S	S	S	n/a	S	S	S	S
13s155	British Columbia Centre for Disease Control	Canada	Delhi	PRJNA413593	S	S	S	n/a	S	S	S	S
12s213	British Columbia Centre for Disease Control	Canada	S-type	PRJNA413593	S	S	S	n/a	S	U	S	S
12s429	British Columbia Centre for Disease Control	Canada	Beijing	PRJNA413593	S	S	S	n/a	S	S	S	S
13s360	British Columbia Centre for Disease Control	Canada	Delhi	PRJNA413593	S	S	S	n/a	S	S	S	S
12s083	British Columbia Centre for Disease Control	Canada	Beijing	PRJNA413593	R	S	S	S	R	S	U	S
14s241	British Columbia Centre for Disease Control	Canada	lineage4	PRJNA413593	S	S	S	n/a	S	S	U	S
14s066r	British Columbia Centre for Disease Control	Canada	Haarlem	PRJNA413593	S	S	S	n/a	S	S	S	S
A8s312	British Columbia Centre for Disease Control	Canada	Beijing	PRJNA413593	S	S	S	n/a	U	S	S	S
11s198	British Columbia Centre for Disease Control	Canada	lineage4	PRJNA413593	S	S	S	n/a	S	S	S	S
A7s329	British Columbia Centre for Disease Control	Canada	lineage4	PRJNA413593	R	S	S	n/a	R	S	S	S
14s305	British Columbia Centre for Disease Control	Canada	EAI	PRJNA413593	S	S	S	n/a	S	S	S	S
14s515	British Columbia Centre for Disease Control	Canada	EAI	PRJNA413593	S	S	S	n/a	S	S	S	S
A8s177	British Columbia Centre for Disease Control	Canada	Beijing	PRJNA413593	S	S	S	n/a	S	S	S	S
A9s372	British Columbia Centre for Disease Control	Canada	EAI	PRJNA413593	S	S	S	n/a	S	S	S	S
A6s150	British Columbia Centre for Disease Control	Canada	EAI	PRJNA413593	S	S	S	n/a	S	S	S	S
A3s009	British Columbia Centre for Disease Control	Canada	S-type	PRJNA413593	S	S	S	n/a	S	S	S	S
14s273	British Columbia Centre for Disease Control	Canada	lineage4	PRJNA413593	S	S	S	n/a	S	S	U	S
A6s288	British Columbia Centre for Disease Control	Canada	Beijing	PRJNA413593	S	S	S	n/a	S	S	S	S
A9s336	British Columbia Centre for Disease Control	Canada	lineage4	PRJNA413593	R	S	S	S	R	S	S	S
A7s189	British Columbia Centre for Disease Control	Canada	Beijing	PRJNA413593	S	S	S	n/a	S	S	S	S
A7s160	British Columbia Centre for Disease Control	Canada	Haarlem	PRJNA413593	S	S	S	n/a	S	S	S	S
10s283	British Columbia Centre for Disease Control	Canada	EAI	PRJNA413593	S	S	S	n/a	U	S	S	S
A9s143	British Columbia Centre for Disease Control	Canada	EAI	PRJNA413593	R	S	S	n/a	R	S	U	S
A6s029	British Columbia Centre for Disease Control	Canada	Delhi	PRJNA413593	S	S	S	n/a	S	S	S	S
13s007	British Columbia Centre for Disease Control	Canada	lineage4	PRJNA413593	S	S	S	n/a	R	S	S	S
12s290	British Columbia Centre for Disease Control	Canada	lineage4	PRJNA413593	S	S	S	n/a	S	S	S	S
A9s169	British Columbia Centre for Disease Control	Canada	EAI	PRJNA413593	S	S	S	n/a	S	S	U	S
10s222	British Columbia Centre for Disease Control	Canada	lineage4	PRJNA413593	S	S	S	n/a	S	U	S	S
11s135	British Columbia Centre for Disease Control	Canada	Haarlem	PRJNA413593	S	S	S	n/a	S	S	S	S
10s308	British Columbia Centre for Disease Control	Canada	lineage4	PRJNA413593	R	S	S	S	R	S	S	S
14s064	British Columbia Centre for Disease Control	Canada	S-type	PRJNA413593	S	S	S	n/a	S	U	S	S
A9s299	British Columbia Centre for Disease Control	Canada	LAM	PRJNA413593	S	S	S	n/a	S	S	S	S
A7s327	British Columbia Centre for Disease Control	Canada	Delhi	PRJNA413593	S	S	S	n/a	S	S	S	S
A8s071	British Columbia Centre for Disease Control	Canada	S-type	PRJNA413593	S	S	S	n/a	S	S	S	S
12s321	British Columbia Centre for Disease Control	Canada	S-type	PRJNA413593	S	S	S	n/a	S	U	S	S
13s500	British Columbia Centre for Disease Control	Canada	Delhi	PRJNA413593	R	S	S	S	R	S	S	S
12s417	British Columbia Centre for Disease Control	Canada	S-type	PRJNA413593	S	S	S	n/a	S	U	S	S
A5s230	British Columbia Centre for Disease Control	Canada	Beijing	PRJNA413593	S	S	S	n/a	U	S	S	S
A7s085	British Columbia Centre for Disease Control	Canada	Ural	PRJNA413593	S	S	S	n/a	S	S	S	S
10s411	British Columbia Centre for Disease Control	Canada	lineage4	PRJNA413593	R	S	S	S	R	S	S	S
A9s349	British Columbia Centre for Disease Control	Canada	Haarlem	PRJNA413593	S	S	S	n/a	S	S	S	S
12s176	British Columbia Centre for Disease Control	Canada	LAM	PRJNA413593	S	S	S	n/a	S	S	S	S
A7s181	British Columbia Centre for Disease Control	Canada	Ural	PRJNA413593	S	S	S	n/a	S	S	S	S
14s066	British Columbia Centre for Disease Control	Canada	Haarlem	PRJNA413593	S	S	S	n/a	S	S	S	S
11s238	British Columbia Centre for Disease Control	Canada	Beijing	PRJNA413593	R	S	S	S	R	S	S	S
11s187	British Columbia Centre for Disease Control	Canada	EAI	PRJNA413593	S	S	S	n/a	U	S	S	S
11s255	British Columbia Centre for Disease Control	Canada	lineage4	PRJNA413593	S	S	S	n/a	S	S	S	S
A5s299	British Columbia Centre for Disease Control	Canada	Beijing	PRJNA413593	S	S	S	n/a	S	S	S	S
A9s269	British Columbia Centre for Disease Control	Canada	Haarlem	PRJNA413593	S	S	S	n/a	U	S	S	S
14s204	British Columbia Centre for Disease Control	Canada	EAI	PRJNA413593	S	S	S	n/a	S	S	S	S
A4s039	British Columbia Centre for Disease Control	Canada	LAM	PRJNA413593	S	S	S	n/a	S	S	S	S
10s313	British Columbia Centre for Disease Control	Canada	lineage4	PRJNA413593	S	S	S	n/a	S	S	S	S
A6s177	British Columbia Centre for Disease Control	Canada	S-type	PRJNA413593	S	S	S	n/a	S	U	S	S
A5s072	British Columbia Centre for Disease Control	Canada	EAI	PRJNA413593	S	S	S	n/a	S	S	S	S
A8s082	British Columbia Centre for Disease Control	Canada	S-type	PRJNA413593	S	S	S	n/a	S	S	U	S
A5s141	British Columbia Centre for Disease Control	Canada	Beijing	PRJNA413593	S	S	S	n/a	S	U	S	S
11s282	British Columbia Centre for Disease Control	Canada	Delhi	PRJNA413593	S	S	S	n/a	S	S	S	S
A8s187	British Columbia Centre for Disease Control	Canada	lineage4	PRJNA413593	S	S	S	n/a	S	S	S	S
A7s108	British Columbia Centre for Disease Control	Canada	EAI	PRJNA413593	S	S	S	n/a	S	S	S	S

A9s082	British Columbia Centre for Disease Control	Canada	lineage4	PRJNA413593	R	n/a	R	n/a	R	S	S	S
13s327	British Columbia Centre for Disease Control	Canada	Haarlem	PRJNA413593	S	S	S	n/a	S	S	S	S
A5s219	British Columbia Centre for Disease Control	Canada	EAI	PRJNA413593	S	S	S	n/a	S	S	S	R
13s052	British Columbia Centre for Disease Control	Canada	EAI	PRJNA413593	R	S	S	S	R	S	S	S
12s284	British Columbia Centre for Disease Control	Canada	Beijing	PRJNA413593	S	S	S	n/a	S	S	S	S
A6s395	British Columbia Centre for Disease Control	Canada	Beijing	PRJNA413593	S	S	S	n/a	S	S	S	S
12s191	British Columbia Centre for Disease Control	Canada	Haarlem	PRJNA413593	S	S	S	n/a	S	S	S	S
10s305	British Columbia Centre for Disease Control	Canada	Delhi	PRJNA413593	S	S	S	n/a	S	S	S	S
A5s243	British Columbia Centre for Disease Control	Canada	Beijing	PRJNA413593	S	S	S	n/a	S	S	S	S
A8s059	British Columbia Centre for Disease Control	Canada	S-type	PRJNA413593	S	S	S	n/a	S	S	U	S
A6s248	British Columbia Centre for Disease Control	Canada	Beijing	PRJNA413593	S	S	S	n/a	S	S	U	S
12s442r	British Columbia Centre for Disease Control	Canada	EAI	PRJNA413593	S	S	S	n/a	S	S	S	S
11s560-ONT	British Columbia Centre for Disease Control	Canada	lineage4	PRJNA413593	R	S	S	S	R	S	S	S
A6s020	British Columbia Centre for Disease Control	Canada	Beijing	PRJNA413593	S	S	S	n/a	S	S	S	S
14s156	British Columbia Centre for Disease Control	Canada	Beijing	PRJNA413593	S	S	S	n/a	S	S	S	S
A6s289	British Columbia Centre for Disease Control	Canada	Ural	PRJNA413593	S	S	S	n/a	S	S	S	S
A5s227	British Columbia Centre for Disease Control	Canada	Beijing	PRJNA413593	S	S	S	n/a	S	S	S	S
A6s086	British Columbia Centre for Disease Control	Canada	EAI	PRJNA413593	S	S	S	n/a	S	S	S	S
11s378	British Columbia Centre for Disease Control	Canada	Haarlem	PRJNA413593	S	S	S	n/a	S	S	S	S
A9s153	British Columbia Centre for Disease Control	Canada	S-type	PRJNA413593	S	S	S	n/a	S	U	S	S
13s405	British Columbia Centre for Disease Control	Canada	EAI	PRJNA413593	S	S	S	n/a	S	S	S	S
10s263	British Columbia Centre for Disease Control	Canada	S-type	PRJNA413593	S	S	S	n/a	S	S	S	S
14s201	British Columbia Centre for Disease Control	Canada	lineage4	PRJNA413593	R	S	S	S	R	S	S	S
12s049	British Columbia Centre for Disease Control	Canada	lineage4	PRJNA413593	R	S	S	S	R	S	S	S
13s065	British Columbia Centre for Disease Control	Canada	Beijing	PRJNA413593	R	S	S	S	R	S	S	S
10s342	British Columbia Centre for Disease Control	Canada	Delhi	PRJNA413593	S	S	S	n/a	S	S	S	S
13s094	British Columbia Centre for Disease Control	Canada	lineage4	PRJNA413593	R	S	S	S	R	S	S	S
A9s136	British Columbia Centre for Disease Control	Canada	Haarlem	PRJNA413593	S	S	S	n/a	S	S	S	S
12s369	British Columbia Centre for Disease Control	Canada	Beijing	PRJNA413593	S	S	S	n/a	S	S	S	S
A5s117	British Columbia Centre for Disease Control	Canada	EAI	PRJNA413593	S	S	S	n/a	S	S	S	S
10s174	British Columbia Centre for Disease Control	Canada	EAI	PRJNA413593	R	R	S	R	R	R	R	R
A6s350	British Columbia Centre for Disease Control	Canada	Haarlem	PRJNA413593	S	S	S	n/a	S	S	S	S
A9s080	British Columbia Centre for Disease Control	Canada	lineage4	PRJNA413593	S	S	S	n/a	S	S	U	S
10s052r	British Columbia Centre for Disease Control	Canada	Beijing	PRJNA413593	S	S	S	n/a	S	S	S	S
10s144	British Columbia Centre for Disease Control	Canada	Beijing	PRJNA413593	S	S	S	n/a	S	S	S	S
A7s161	British Columbia Centre for Disease Control	Canada	Ural	PRJNA413593	S	S	S	n/a	S	S	S	S
14s046	British Columbia Centre for Disease Control	Canada	Beijing	PRJNA413593	S	S	S	n/a	S	S	S	S
13s046	British Columbia Centre for Disease Control	Canada	Haarlem	PRJNA413593	S	S	S	n/a	S	S	S	S
A9s394	British Columbia Centre for Disease Control	Canada	Delhi	PRJNA413593	R	S	S	S	R	S	S	S
A6s149	British Columbia Centre for Disease Control	Canada	Ural	PRJNA413593	S	S	S	n/a	S	S	S	S
11s342	British Columbia Centre for Disease Control	Canada	Beijing	PRJNA413593	S	S	S	n/a	S	S	S	S
13s170	British Columbia Centre for Disease Control	Canada	S-type	PRJNA413593	S	S	S	n/a	S	S	U	S
A4s329	British Columbia Centre for Disease Control	Canada	Ural	PRJNA413593	S	S	S	n/a	S	S	S	S
A9s305	British Columbia Centre for Disease Control	Canada	Haarlem	PRJNA413593	S	S	S	n/a	S	S	S	S
A8s333	British Columbia Centre for Disease Control	Canada	S-type	PRJNA413593	S	S	S	n/a	S	S	S	S
A9s188	British Columbia Centre for Disease Control	Canada	S-type	PRJNA413593	S	S	S	n/a	S	U	S	S
A9s089	British Columbia Centre for Disease Control	Canada	S-type	PRJNA413593	S	S	S	n/a	S	U	S	S
10s081	British Columbia Centre for Disease Control	Canada	lineage4	PRJNA413593	S	S	S	n/a	S	S	U	S
A9s067	British Columbia Centre for Disease Control	Canada	S-type	PRJNA413593	S	S	S	n/a	S	S	S	S
11s448	British Columbia Centre for Disease Control	Canada	EAI	PRJNA413593	S	S	S	n/a	S	S	S	S
A8s022	British Columbia Centre for Disease Control	Canada	EAI	PRJNA413593	R	S	S	n/a	F	S	S	S
14s306	British Columbia Centre for Disease Control	Canada	S-type	PRJNA413593	S	R	S	S	S	R	S	S
10s004	British Columbia Centre for Disease Control	Canada	EAI	PRJNA413593	S	S	S	n/a	S	S	S	S
10s120	British Columbia Centre for Disease Control	Canada	S-type	PRJNA413593	S	S	S	n/a	S	S	S	S
A9s052	British Columbia Centre for Disease Control	Canada	LAM	PRJNA413593	S	S	S	n/a	S	S	S	S
12s167	British Columbia Centre for Disease Control	Canada	Delhi	PRJNA413593	S	S	S	n/a	S	S	S	S
A8s126	British Columbia Centre for Disease Control	Canada	Beijing	PRJNA413593	S	S	S	n/a	S	S	U	S
11s142	British Columbia Centre for Disease Control	Canada	S-type	PRJNA413593	S	S	S	n/a	S	U	S	S
A6s154	British Columbia Centre for Disease Control	Canada	EAI	PRJNA413593	S	S	S	n/a	S	S	S	S
A6s119	British Columbia Centre for Disease Control	Canada	Beijing	PRJNA413593	S	S	S	n/a	S	S	S	S
14s174	British Columbia Centre for Disease Control	Canada	lineage4	PRJNA413593	S	S	S	n/a	S	S	S	S
10s301	British Columbia Centre for Disease Control	Canada	EAI	PRJNA413593	S	S	S	n/a	S	S	U	S
A6s042	British Columbia Centre for Disease Control	Canada	Beijing	PRJNA413593	S	S	S	n/a	U	S	S	S
A5s180	British Columbia Centre for Disease Control	Canada	EAI	PRJNA413593	S	S	S	n/a	S	S	S	S
14s008	British Columbia Centre for Disease Control	Canada	Haarlem	PRJNA413593	S	S	S	n/a	S	S	S	S
11s103	British Columbia Centre for Disease Control	Canada	lineage4	PRJNA413593	S	S	S	n/a	R	S	S	S
A6s386	British Columbia Centre for Disease Control	Canada	Delhi	PRJNA413593	S	S	S	n/a	S	S	S	S
11s093	British Columbia Centre for Disease Control	Canada	Beijing	PRJNA413593	R	S	S	S	R	S	S	S
11s042	British Columbia Centre for Disease Control	Canada	Delhi	PRJNA413593	S	S	S	n/a	S	U	S	S
A8s133	British Columbia Centre for Disease Control	Canada	Haarlem	PRJNA413593	S	S	S	n/a	S	S	S	S
14s178	British Columbia Centre for Disease Control	Canada	lineage4	PRJNA413593	S	S	S	n/a	R	S	S	S
A5s212	British Columbia Centre for Disease Control	Canada	S-type	PRJNA413593	S	S	S	n/a	S	U	S	S
A8s277	British Columbia Centre for Disease Control	Canada	Beijing	PRJNA413593	S	S	S	n/a	S	S	U	S
A6s294	British Columbia Centre for Disease Control	Canada	EAI	PRJNA413593	S	S	S	n/a	S	S	S	S
14s081	British Columbia Centre for Disease Control	Canada	EAI	PRJNA413593	S	S	S	n/a	S	S	S	S
10s405	British Columbia Centre for Disease Control	Canada	Delhi	PRJNA413593	S	S	S	n/a	S	S	S	S
A5s095	British Columbia Centre for Disease Control	Canada	S-type	PRJNA413593	S	S	S	n/a	S	S	S	S
A9s230	British Columbia Centre for Disease Control	Canada	lineage4	PRJNA413593	S	S	S	n/a	S	S	S	S
14s038	British Columbia Centre for Disease Control	Canada	Beijing	PRJNA413593	S	S	S	n/a	S	S	S	S
A8s257	British Columbia Centre for Disease Control	Canada	Beijing	PRJNA413593	S	S	S	n/a	S	S	S	S
A6s072	British Columbia Centre for Disease Control	Canada	S-type	PRJNA413593	S	R	S	S	S	R	S	S
A6s192	British Columbia Centre for Disease Control	Canada	Delhi	PRJNA413593	S	S	S	n/a	S	S	S	S
11s064	British Columbia Centre for Disease Control	Canada	Beijing	PRJNA413593	R	S	S	S	R	S	S	S
13s451	British Columbia Centre for Disease Control	Canada	Beijing	PRJNA413593	R	S	S	S	U	S	S	S
13s294	British Columbia Centre for Disease Control	Canada	EAI	PRJNA413593	S	S	S	n/a	U	R	S	S
A7s221	British Columbia Centre for Disease Control	Canada	Beijing	PRJNA413593	S	S	S	n/a	S	S	S	S
A7s308	British Columbia Centre for Disease Control	Canada	Beijing	PRJNA413593	S	S	S	n/a	S	S	S	S
14s367	British Columbia Centre for Disease Control	Canada	Delhi	PRJNA413593	S	S	S	n/a	S	S	S	R
A8s100	British Columbia Centre for Disease Control	Canada	lineage4	PRJNA413593	S	S	S	n/a	S	S	S	S
11s032	British Columbia Centre for Disease Control	Canada	Delhi	PRJNA413593	S	S	S	n/a	S	S	S	S
A1s092	British Columbia Centre for Disease Control	Canada	Haarlem	PRJNA413593	S	S	S	n/a	S	S	S	S
11s218	British Columbia Centre for Disease Control	Canada	Beijing	PRJNA413593	S	S	S	n/a	S	S	S	S
13s098	British Columbia Centre for Disease Control	Canada	Beijing	PRJNA413593	S	S	S	n/a	S	S	S	S
12s275	British Columbia Centre for Disease Control	Canada	S-type	PRJNA413593	S	S	S	n/a	S	U	S	S
14s069	British Columbia Centre for Disease Control	Canada	Ural	PRJNA413593	S	S	S	n/a	S	S	S	S
10s282	British Columbia Centre for Disease Control	Canada	lineage4	PRJNA413593	S	S	S	n/a	S	S	S	S
A7s312	British Columbia Centre for Disease Control	Canada	Haarlem	PRJNA413593	S	S	S	n/a	S	S	S	S
12s453	British Columbia Centre for Disease Control	Canada	Beijing	PRJNA413593	S	S	S	n/a	S	S	S	S
A9s287	British Columbia Centre for Disease Control	Canada	Haarlem	PRJNA413593	S	S	S	n/a	S	S	S	S
12s022	British Columbia Centre for Disease Control	Canada	S-type	PRJNA413593	S	S	S	n/a	S	U	S	S
10s150	British Columbia Centre for Disease Control	Canada	Beijing	PRJNA413593	S	S	S	n/a	S	S	S	S
11s050	British Columbia Centre for Disease Control	Canada	Ural	PRJNA413593	S	S	S	n/a	S	S	S	S
A8s078	British Columbia Centre for Disease Control	Canada	Beijing	PRJNA413593	R	R	S	S	R	R	S	S
12s258	British Columbia Centre for Disease Control	Canada	Beijing	PRJNA413593	S	S	S	n/a	S	S	S	S
A9s068	British Columbia Centre for Disease Control	Canada	Beijing	PRJNA413593	S	S	S	n/a	S	S	S	S
A7s210	British Columbia Centre for Disease Control	Canada	EAI	PRJNA413593	S	S	S	n/a	S	S	S	S
A7S301	British Columbia Centre for Disease Control	Canada	X-type	PRJNA413593	S	S	S	n/a	S	S	S	S
14s372	British Columbia Centre for Disease Control	Canada	Delhi	PRJNA413593	S	S	S	n/a	S	S	S	S
A9s256	British Columbia Centre for Disease Control	Canada	Beijing	PRJNA413593	S	S	S	n/a	S	S	S	S
12s059	British Columbia Centre for Disease Control	Canada	lineage4	PRJNA413593	R	S	S	S	R	S	S	S
A9s092	British Columbia Centre for Disease Control	Canada	EAI	PRJNA413593	S	S	S	n/a	S	S	S	S
13s370	British Columbia Centre for Disease Control	Canada	Haarlem	PRJNA413593	S	S	S	n/a	S	S	S	S
14s478	British Columbia Centre for Disease Control	Canada	Delhi	PRJNA413593	S	S	S	n/a	S	S	S	S
A9s093	British Columbia Centre for Disease Control	Canada	lineage4	PRJNA413593	S	S	S	n/a	S	S	S	S
A6s024	British Columbia Centre for Disease Control	Canada	S-type	PRJNA413593	S	R	S	S	S	R	S	S
A7s283	British Columbia Centre for Disease Control	Canada	EAI	PRJNA413593	S	S	S	n/a	S	S	S	S

12s154	British Columbia Centre for Disease Control	Canada	Haarlem		PRJNA413593	S	S	S	n/a	S	S	S	S
13s380	British Columbia Centre for Disease Control	Canada	EAI		PRJNA413593	S	S	S	n/a	S	S	S	S
10s066	British Columbia Centre for Disease Control	Canada	Beijing		PRJNA413593	S	S	S	n/a	S	S	S	S
13s282	British Columbia Centre for Disease Control	Canada	lineage4		PRJNA413593	S	S	S	n/a	S	S	S	S
11s212	British Columbia Centre for Disease Control	Canada	EAI		PRJNA413593	S	S	S	n/a	S	S	S	S
11s150	British Columbia Centre for Disease Control	Canada	EAI		PRJNA413593	R	S	S	S	R	S	U	S
12s151	British Columbia Centre for Disease Control	Canada	Beijing		PRJNA413593	S	S	S	n/a	S	S	S	S
A9s261	British Columbia Centre for Disease Control	Canada	Beijing		PRJNA413593	R	S	S	n/a	R	S	S	S
A5s108	British Columbia Centre for Disease Control	Canada	Delhi		PRJNA413593	S	S	S	n/a	S	S	S	S
10s400	British Columbia Centre for Disease Control	Canada	Beijing		PRJNA413593	S	S	S	n/a	S	S	S	S
A9s009	British Columbia Centre for Disease Control	Canada	Delhi		PRJNA413593	S	S	S	n/a	S	S	S	S
11s346	British Columbia Centre for Disease Control	Canada	Beijing		PRJNA413593	S	S	S	n/a	S	S	U	S
A8s024	British Columbia Centre for Disease Control	Canada	S-type		PRJNA413593	S	S	S	n/a	S	F	S	S
A6s348	British Columbia Centre for Disease Control	Canada	Beijing		PRJNA413593	S	S	S	n/a	S	S	S	S
A6s401	British Columbia Centre for Disease Control	Canada	Haarlem		PRJNA413593	S	S	S	n/a	S	S	S	S
A4s041	British Columbia Centre for Disease Control	Canada	Ural		PRJNA413593	S	S	S	n/a	S	S	U	S
A8s366	British Columbia Centre for Disease Control	Canada	S-type		PRJNA413593	S	S	S	n/a	U	S	S	S
A6s122	British Columbia Centre for Disease Control	Canada	EAI		PRJNA413593	S	S	S	n/a	S	S	S	S
11s047	British Columbia Centre for Disease Control	Canada	lineage4		PRJNA413593	R	S	S	S	R	S	S	S
11s120	British Columbia Centre for Disease Control	Canada	S-type		PRJNA413593	S	S	S	n/a	S	U	S	S
12s309	British Columbia Centre for Disease Control	Canada	Tur		PRJNA413593	S	S	S	n/a	S	S	S	S
A5s228	British Columbia Centre for Disease Control	Canada	lineage4		PRJNA413593	S	S	S	n/a	S	S	U	S
A6s240	British Columbia Centre for Disease Control	Canada	Haarlem		PRJNA413593	S	S	S	n/a	S	S	S	S
13s345	British Columbia Centre for Disease Control	Canada	lineage4		PRJNA413593	R	S	S	S	R	S	S	S
11s149	British Columbia Centre for Disease Control	Canada	Haarlem		PRJNA413593	S	S	S	n/a	S	S	S	S
A8s027	British Columbia Centre for Disease Control	Canada	EAI		PRJNA413593	S	S	S	n/a	S	S	S	S
A9s246	British Columbia Centre for Disease Control	Canada	Beijing		PRJNA413593	S	S	S	n/a	S	S	S	S
12s146	British Columbia Centre for Disease Control	Canada	Beijing		PRJNA413593	S	S	S	n/a	S	S	S	S
13s371	British Columbia Centre for Disease Control	Canada	lineage4		PRJNA413593	S	S	S	n/a	S	S	S	S
11s168	British Columbia Centre for Disease Control	Canada	EAI		PRJNA413593	S	S	S	n/a	S	S	S	S
A7s013	British Columbia Centre for Disease Control	Canada	Ural		PRJNA413593	S	S	S	n/a	S	S	S	S
A8s182	British Columbia Centre for Disease Control	Canada	Delhi		PRJNA413593	S	S	S	n/a	S	S	S	S
12s203	British Columbia Centre for Disease Control	Canada	S-type		PRJNA413593	S	S	S	n/a	S	U	S	S
A8s198	British Columbia Centre for Disease Control	Canada	Ural		PRJNA413593	S	S	S	n/a	S	S	S	S
13s305	British Columbia Centre for Disease Control	Canada	Beijing		PRJNA413593	S	S	S	n/a	S	S	S	S
A5s001	British Columbia Centre for Disease Control	Canada	lineage4		PRJNA413593	S	S	S	n/a	S	S	S	S
A6s254	British Columbia Centre for Disease Control	Canada	EAI		PRJNA413593	S	S	S	n/a	S	S	S	S
A9s151	British Columbia Centre for Disease Control	Canada	Ural		PRJNA413593	S	S	S	n/a	S	S	U	S
A6s273	British Columbia Centre for Disease Control	Canada	lineage4		PRJNA413593	S	S	S	n/a	R	S	S	S
A8s111	British Columbia Centre for Disease Control	Canada	Haarlem		PRJNA413593	S	S	S	n/a	S	S	S	S
A9s149	British Columbia Centre for Disease Control	Canada	Beijing		PRJNA413593	S	S	S	n/a	U	S	S	S
14s165	British Columbia Centre for Disease Control	Canada	Haarlem		PRJNA413593	S	S	S	n/a	S	S	S	S
A5s213	British Columbia Centre for Disease Control	Canada	lineage4		PRJNA413593	S	S	S	n/a	S	S	S	S
11s006	British Columbia Centre for Disease Control	Canada	Haarlem		PRJNA413593	S	S	S	n/a	S	S	S	S
14s235	British Columbia Centre for Disease Control	Canada	Delhi		PRJNA413593	R	S	S	S	F	S	S	S
13s386	British Columbia Centre for Disease Control	Canada	EAI		PRJNA413593	S	S	S	n/a	S	S	S	S
A4s061	British Columbia Centre for Disease Control	Canada	S-type		PRJNA413593	S	S	S	n/a	S	U	S	S
A7s073	British Columbia Centre for Disease Control	Canada	EAI		PRJNA413593	S	S	S	n/a	S	S	S	S
A8s255	British Columbia Centre for Disease Control	Canada	EAI		PRJNA413593	S	S	S	n/a	S	S	S	S
A7s036	British Columbia Centre for Disease Control	Canada	Beijing		PRJNA413593	S	S	S	n/a	S	S	S	S
A7s056	British Columbia Centre for Disease Control	Canada	lineage4		PRJNA413593	S	S	S	n/a	S	S	S	S
A5s089	British Columbia Centre for Disease Control	Canada	Beijing		PRJNA413593	S	S	S	n/a	S	S	S	S
A4s022	British Columbia Centre for Disease Control	Canada	Beijing		PRJNA413593	S	S	S	n/a	S	S	S	S
10s267	British Columbia Centre for Disease Control	Canada	Haarlem		PRJNA413593	S	S	S	S	S	S	S	S
A6s329	British Columbia Centre for Disease Control	Canada	Haarlem		PRJNA413593	S	S	S	n/a	S	S	S	S
12s142	British Columbia Centre for Disease Control	Canada	EAI		PRJNA413593	S	S	S	n/a	S	S	S	S
12s214	British Columbia Centre for Disease Control	Canada	Beijing		PRJNA413593	R	S	S	S	R	S	S	S
A9s131	British Columbia Centre for Disease Control	Canada	lineage4		PRJNA413593	S	S	S	n/a	S	S	S	S
A8s097	British Columbia Centre for Disease Control	Canada	Beijing		PRJNA413593	S	S	S	n/a	S	S	S	S
A9s148	British Columbia Centre for Disease Control	Canada	S-type		PRJNA413593	S	S	S	n/a	S	U	S	S
13s218	British Columbia Centre for Disease Control	Canada	Beijing		PRJNA413593	S	S	S	n/a	S	S	S	S
11s356	British Columbia Centre for Disease Control	Canada	EAI		PRJNA413593	R	S	S	S	R	S	S	S
A7s208	British Columbia Centre for Disease Control	Canada	Beijing		PRJNA413593	S	S	S	n/a	S	S	S	S
10s012	British Columbia Centre for Disease Control	Canada	lineage4		PRJNA413593	S	S	S	n/a	S	S	S	S
12s268	British Columbia Centre for Disease Control	Canada	Beijing		PRJNA413593	R	S	S	S	R	S	S	S
A5s151	British Columbia Centre for Disease Control	Canada	EAI		PRJNA413593	R	S	S	S	R	R	S	S
A0s018	British Columbia Centre for Disease Control	Canada	Beijing		PRJNA413593	S	S	S	n/a	U	S	S	S
12s305	British Columbia Centre for Disease Control	Canada	Haarlem		PRJNA413593	S	S	S	n/a	S	S	S	S
A8s241	British Columbia Centre for Disease Control	Canada	Beijing		PRJNA413593	R	S	S	n/a	R	S	S	S
A6s082	British Columbia Centre for Disease Control	Canada	EAI		PRJNA413593	S	S	S	n/a	S	S	S	S
10s103	British Columbia Centre for Disease Control	Canada	EAI		PRJNA413593	R	S	S	S	R	S	S	S
10s002	British Columbia Centre for Disease Control	Canada	Beijing		PRJNA413593	S	S	S	n/a	S	S	S	S
A8s314	British Columbia Centre for Disease Control	Canada	Haarlem		PRJNA413593	S	S	S	n/a	S	S	S	S
12s441	British Columbia Centre for Disease Control	Canada	Beijing		PRJNA413593	S	S	S	n/a	S	S	S	S
11s400	British Columbia Centre for Disease Control	Canada	S-type		PRJNA413593	S	S	S	n/a	S	U	S	S
A6s211	British Columbia Centre for Disease Control	Canada	EAI		PRJNA413593	S	S	S	n/a	S	S	S	S
10s364	British Columbia Centre for Disease Control	Canada	EAI		PRJNA413593	R	S	S	S	R	S	S	S
10s388	British Columbia Centre for Disease Control	Canada	EAI		PRJNA413593	S	S	S	n/a	S	S	S	S
11s388	British Columbia Centre for Disease Control	Canada	EAI		PRJNA413593	S	S	S	n/a	S	S	S	S
A6s425	British Columbia Centre for Disease Control	Canada	Beijing		PRJNA413593	S	S	S	n/a	S	S	S	S
14s051	British Columbia Centre for Disease Control	Canada	lineage4		PRJNA413593	S	S	S	n/a	S	S	S	S
12s388	British Columbia Centre for Disease Control	Canada	Beijing		PRJNA413593	S	S	S	n/a	S	S	S	S
10s443	British Columbia Centre for Disease Control	Canada	lineage4		PRJNA413593	S	S	S	n/a	S	S	S	S
12s169	British Columbia Centre for Disease Control	Canada	S-type		PRJNA413593	S	S	S	n/a	S	U	S	S
A6s303	British Columbia Centre for Disease Control	Canada	S-type		PRJNA413593	S	S	S	n/a	U	S	S	S
A6s159	British Columbia Centre for Disease Control	Canada	Haarlem		PRJNA413593	S	S	S	n/a	S	S	S	S
A3s144	British Columbia Centre for Disease Control	Canada	Haarlem		PRJNA413593	S	S	S	n/a	S	S	S	S
A8s134	British Columbia Centre for Disease Control	Canada	Haarlem		PRJNA413593	S	S	S	n/a	S	S	S	S
A8s110	British Columbia Centre for Disease Control	Canada	Haarlem		PRJNA413593	S	S	S	n/a	S	S	S	S
A6s259	British Columbia Centre for Disease Control	Canada	S-type		PRJNA413593	S	R	S	n/a	S	R	S	S
A9s129	British Columbia Centre for Disease Control	Canada	Delhi		PRJNA413593	S	S	S	n/a	S	S	S	S
14s169	British Columbia Centre for Disease Control	Canada	Delhi		PRJNA413593	S	S	S	n/a	S	U	S	S
12s283	British Columbia Centre for Disease Control	Canada	Beijing		PRJNA413593	S	S	S	n/a	U	S	S	S
14s277	British Columbia Centre for Disease Control	Canada	Beijing		PRJNA413593	S	S	S	n/a	S	S	S	S
10s322	British Columbia Centre for Disease Control	Canada	Beijing		PRJNA413593	S	S	S	n/a	S	S	U	S
A0s075	British Columbia Centre for Disease Control	Canada	EAI		PRJNA413593	S	S	S	n/a	S	S	S	R
A7s303	British Columbia Centre for Disease Control	Canada	S-type		PRJNA413593	S	S	S	n/a	S	U	S	S
A7s017	British Columbia Centre for Disease Control	Canada	Haarlem		PRJNA413593	S	S	S	n/a	S	S	S	S
A7s251	British Columbia Centre for Disease Control	Canada	EAI		PRJNA413593	S	S	S	n/a	S	S	S	S
A6s158	British Columbia Centre for Disease Control	Canada	Ural		PRJNA413593	S	S	S	n/a	S	S	S	S
14s409	British Columbia Centre for Disease Control	Canada	Haarlem		PRJNA413593	S	S	S	n/a	S	S	S	S
14s086r	British Columbia Centre for Disease Control	Canada	Beijing		PRJNA413593	S	S	S	n/a	S	S	S	S
A7s092	British Columbia Centre for Disease Control	Canada	Uganda		PRJNA413593	S	S	S	n/a	U	S	U	S
12s003	British Columbia Centre for Disease Control	Canada	Beijing		PRJNA413593	S	S	S	n/a	S	S	U	S
12s396	British Columbia Centre for Disease Control	Canada	Haarlem		PRJNA413593	S	S	S	n/a	S	S	S	S
12s434	British Columbia Centre for Disease Control	Canada	S-type		PRJNA413593	S	S	S	n/a	S	U	S	S
A7S361	British Columbia Centre for Disease Control	Canada	S-type		PRJNA413593	S	S	S	S	S	S	U	S
A9s050	British Columbia Centre for Disease Control	Canada	Haarlem		PRJNA413593	S	S	S	n/a	S	S	U	S
13s183	British Columbia Centre for Disease Control	Canada	Delhi		PRJNA413593	S	S	S	n/a	S	S	S	S
A7s054	British Columbia Centre for Disease Control	Canada	Beijing		PRJNA413593	S	S	S	n/a	S	S	S	S
10s379	British Columbia Centre for Disease Control	Canada	S-type		PRJNA413593	S	S	S	n/a	S	U	S	S
14s065	British Columbia Centre for Disease Control	Canada	EAI		PRJNA413593	S	S	S	n/a	S	S	S	S
A2s262	British Columbia Centre for Disease Control	Canada	EAI		PRJNA413593	S	S	S	n/a	S	S	S	S
09_1058	Yang et. al. Lancet Infect Dis. 2017 Mar;17(3):275-284	China	Beijing	SRS935950	SRP058221	R	R	R	n/a	R	R	R	S
12_1828	Yang et. al. Lancet Infect Dis. 2017 Mar;17(3):275-284	China	Beijing	SRS935942	SRP058221	R	R	R	n/a	R	R	U	R
10_0390	Yang et. al. Lancet Infect Dis. 2017 Mar;17(3):275-284	China	Beijing	SRS935989	SRP058221	R	R	R	n/a	R	R	R	U

10_1872	Yang et. al. Lancet Infect Dis. 2017 Mar;17(3):275-284	China	Beijing	SRS936003	SRP058221	R	R	R	n/a	R	R	R	S
10_1081	Yang et. al. Lancet Infect Dis. 2017 Mar;17(3):275-284	China	Beijing	SRS936009	SRP058221	R	R	R	n/a	R	R	R	R
09_0784	Yang et. al. Lancet Infect Dis. 2017 Mar;17(3):275-284	China	Beijing	SRS935956	SRP058221	R	R	S	n/a	R	R	S	S
12_1050	Yang et. al. Lancet Infect Dis. 2017 Mar;17(3):275-284	China	Beijing	SRS935922	SRP058221	R	R	R	n/a	R	R	R	R
09_0643	Yang et. al. Lancet Infect Dis. 2017 Mar;17(3):275-284	China	Beijing	SRS936002	SRP058221	R	R	S	n/a	R	R	S	R
10_1730	Yang et. al. Lancet Infect Dis. 2017 Mar;17(3):275-284	China	Beijing	SRS936006	SRP058221	R	R	R	n/a	R	R	R	S
11_0344	Yang et. al. Lancet Infect Dis. 2017 Mar;17(3):275-284	China	Beijing	SRS936022	SRP058221	R	R	R	n/a	R	R	R	R
11_1549	Yang et. al. Lancet Infect Dis. 2017 Mar;17(3):275-284	China	Beijing	SRS936036	SRP058221	R	R	R	n/a	R	R	R	U
10_0122	Yang et. al. Lancet Infect Dis. 2017 Mar;17(3):275-284	China	Beijing	SRS935969	SRP058221	R	R	S	n/a	R	R	U	S
09_0620	Yang et. al. Lancet Infect Dis. 2017 Mar;17(3):275-284	China	Beijing	SRS935999	SRP058221	R	R	R	n/a	R	R	R	R
12_1055	Yang et. al. Lancet Infect Dis. 2017 Mar;17(3):275-284	China	Beijing	SRS935925	SRP058221	R	R	S	n/a	R	R	S	S
09_1994	Yang et. al. Lancet Infect Dis. 2017 Mar;17(3):275-284	China	Beijing	SRS935972	SRP058221	R	R	R	n/a	R	R	R	S
10_0806	Yang et. al. Lancet Infect Dis. 2017 Mar;17(3):275-284	China	Beijing	SRS935982	SRP058221	R	R	R	n/a	R	R	R	F
12_1813	Yang et. al. Lancet Infect Dis. 2017 Mar;17(3):275-284	China	S-type	SRS935943	SRP058221	R	R	R	n/a	R	R	R	R
09_0817	Yang et. al. Lancet Infect Dis. 2017 Mar;17(3):275-284	China	Beijing	SRS935954	SRP058221	R	R	R	n/a	R	R	R	S
12_0219	Yang et. al. Lancet Infect Dis. 2017 Mar;17(3):275-284	China	Beijing	SRS936035	SRP058221	R	R	R	n/a	R	R	R	S
10_0621	Yang et. al. Lancet Infect Dis. 2017 Mar;17(3):275-284	China	Beijing	SRS935984	SRP058221	R	R	R	n/a	R	R	U	S
09_0994	Yang et. al. Lancet Infect Dis. 2017 Mar;17(3):275-284	China	Beijing	SRS935952	SRP058221	R	R	R	n/a	R	R	R	R
10_0190	Yang et. al. Lancet Infect Dis. 2017 Mar;17(3):275-284	China	Beijing	SRS935994	SRP058221	R	R	S	n/a	R	R	S	S
12_1484	Yang et. al. Lancet Infect Dis. 2017 Mar;17(3):275-284	China	Beijing	SRS935923	SRP058221	R	R	R	n/a	R	R	F	F
09_1161	Yang et. al. Lancet Infect Dis. 2017 Mar;17(3):275-284	China	Beijing	SRS935957	SRP058221	R	R	R	n/a	R	R	R	U
12_0567	Yang et. al. Lancet Infect Dis. 2017 Mar;17(3):275-284	China	Beijing	SRS935932	SRP058221	R	R	S	n/a	R	R	S	S
09_0295	Yang et. al. Lancet Infect Dis. 2017 Mar;17(3):275-284	China	Beijing	SRS935967	SRP058221	R	R	S	n/a	R	R	S	S
12_0358	Yang et. al. Lancet Infect Dis. 2017 Mar;17(3):275-284	China	Beijing	SRS935935	SRP058221	R	R	R	n/a	R	R	R	R
11_1912	Yang et. al. Lancet Infect Dis. 2017 Mar;17(3):275-284	China	Beijing	SRS936033	SRP058221	R	R	S	n/a	R	R	S	S
12_0603	Yang et. al. Lancet Infect Dis. 2017 Mar;17(3):275-284	China	Beijing	SRS935921	SRP058221	R	R	R	n/a	R	R	R	R
09_1303	Yang et. al. Lancet Infect Dis. 2017 Mar;17(3):275-284	China	Beijing	SRS935964	SRP058221	R	R	S	n/a	R	R	R	F
09_1333	Yang et. al. Lancet Infect Dis. 2017 Mar;17(3):275-284	China	Beijing	SRS935965	SRP058221	R	R	S	n/a	R	R	U	S
09_1862	Yang et. al. Lancet Infect Dis. 2017 Mar;17(3):275-284	China	Beijing	SRS935975	SRP058221	R	R	R	n/a	R	R	U	F
11_0144	Yang et. al. Lancet Infect Dis. 2017 Mar;17(3):275-284	China	Beijing	SRS936012	SRP058221	R	R	R	n/a	R	R	R	R
12_0043	Yang et. al. Lancet Infect Dis. 2017 Mar;17(3):275-284	China	Beijing	SRS936030	SRP058221	R	R	S	n/a	R	R	S	R
10_0381	Yang et. al. Lancet Infect Dis. 2017 Mar;17(3):275-284	China	Beijing	SRS935990	SRP058221	R	R	S	n/a	R	R	U	S
11_2094	Yang et. al. Lancet Infect Dis. 2017 Mar;17(3):275-284	China	Beijing	SRS936032	SRP058221	R	R	S	n/a	R	R	R	S
10_0757	Yang et. al. Lancet Infect Dis. 2017 Mar;17(3):275-284	China	S-type	SRS935979	SRP058221	R	R	R	n/a	R	R	R	R
10_2132	Yang et. al. Lancet Infect Dis. 2017 Mar;17(3):275-284	China	Beijing	SRS936027	SRP058221	R	R	S	n/a	R	R	R	R
10_0539	Yang et. al. Lancet Infect Dis. 2017 Mar;17(3):275-284	China	Beijing	SRS935988	SRP058221	R	R	R	n/a	R	R	S	R
10_1563	Yang et. al. Lancet Infect Dis. 2017 Mar;17(3):275-284	China	Beijing	SRS935997	SRP058221	R	R	R	n/a	R	R	R	S
12_0659	Yang et. al. Lancet Infect Dis. 2017 Mar;17(3):275-284	China	Beijing	SRS935931	SRP058221	R	R	R	n/a	R	R	R	R
10_2065	Yang et. al. Lancet Infect Dis. 2017 Mar;17(3):275-284	China	Beijing	SRS936029	SRP058221	R	R	R	n/a	F	F	F	S
12_1614	Yang et. al. Lancet Infect Dis. 2017 Mar;17(3):275-284	China	Beijing	SRS935946	SRP058221	R	R	S	n/a	R	R	S	S
10_0768	Yang et. al. Lancet Infect Dis. 2017 Mar;17(3):275-284	China	Beijing	SRS935983	SRP058221	R	R	S	n/a	R	R	S	S
10_2206	Yang et. al. Lancet Infect Dis. 2017 Mar;17(3):275-284	China	Beijing	SRS936016	SRP058221	R	R	S	n/a	R	R	S	S
10_0020	Yang et. al. Lancet Infect Dis. 2017 Mar;17(3):275-284	China	Beijing	SRS935971	SRP058221	R	R	R	n/a	R	R	U	R
10_2303	Yang et. al. Lancet Infect Dis. 2017 Mar;17(3):275-284	China	Beijing	SRS936013	SRP058221	R	R	S	n/a	R	R	S	S
09_0511	Yang et. al. Lancet Infect Dis. 2017 Mar;17(3):275-284	China	Beijing	SRS935986	SRP058221	R	R	R	n/a	R	R	R	S
10_0054	Yang et. al. Lancet Infect Dis. 2017 Mar;17(3):275-284	China	Beijing	SRS935970	SRP058221	R	R	S	n/a	R	R	U	S
09_1413	Yang et. al. Lancet Infect Dis. 2017 Mar;17(3):275-284	China	Beijing	SRS935962	SRP058221	R	R	R	n/a	R	R	S	S
10_1180	Yang et. al. Lancet Infect Dis. 2017 Mar;17(3):275-284	China	Beijing	SRS936008	SRP058221	R	R	R	n/a	R	R	S	S
10_2094	Yang et. al. Lancet Infect Dis. 2017 Mar;17(3):275-284	China	Beijing	SRS936028	SRP058221	R	R	S	n/a	U	R	U	R
11_1025	Yang et. al. Lancet Infect Dis. 2017 Mar;17(3):275-284	China	Beijing	SRS936038	SRP058221	R	R	R	n/a	R	R	R	R
09_1780	Yang et. al. Lancet Infect Dis. 2017 Mar;17(3):275-284	China	Beijing	SRS935976	SRP058221	R	R	S	n/a	R	R	R	F
10_2246	Yang et. al. Lancet Infect Dis. 2017 Mar;17(3):275-284	China	Beijing	SRS936014	SRP058221	R	R	S	n/a	R	R	S	S
10_1753	Yang et. al. Lancet Infect Dis. 2017 Mar;17(3):275-284	China	Beijing	SRS936000	SRP058221	R	R	R	n/a	R	R	R	R
11_0370	Yang et. al. Lancet Infect Dis. 2017 Mar;17(3):275-284	China	Beijing	SRS936021	SRP058221	R	R	R	n/a	R	R	R	S
10_0560	Yang et. al. Lancet Infect Dis. 2017 Mar;17(3):275-284	China	Beijing	SRS935987	SRP058221	R	R	S	n/a	R	R	R	S
12_0696	Yang et. al. Lancet Infect Dis. 2017 Mar;17(3):275-284	China	Beijing	SRS935930	SRP058221	R	R	R	n/a	R	R	U	R
11_0209	Yang et. al. Lancet Infect Dis. 2017 Mar;17(3):275-284	China	Beijing	SRS936011	SRP058221	R	R	S	n/a	R	R	S	S
10_0614	Yang et. al. Lancet Infect Dis. 2017 Mar;17(3):275-284	China	Beijing	SRS935985	SRP058221	R	R	R	n/a	R	R	S	R
09_1687	Yang et. al. Lancet Infect Dis. 2017 Mar;17(3):275-284	China	Beijing	SRS935960	SRP058221	R	R	S	n/a	R	R	S	U
11_0227	Yang et. al. Lancet Infect Dis. 2017 Mar;17(3):275-284	China	Beijing	SRS936025	SRP058221	R	R	S	n/a	R	R	S	U
11_0295	Yang et. al. Lancet Infect Dis. 2017 Mar;17(3):275-284	China	Beijing	SRS936023	SRP058221	R	R	R	n/a	R	R	R	R
12_0569	Yang et. al. Lancet Infect Dis. 2017 Mar;17(3):275-284	China	Beijing	SRS935933	SRP058221	R	R	S	n/a	R	R	U	S
12_1655	Yang et. al. Lancet Infect Dis. 2017 Mar;17(3):275-284	China	Beijing	SRS935944	SRP058221	R	R	R	n/a	R	R	U	U
10_0876	Yang et. al. Lancet Infect Dis. 2017 Mar;17(3):275-284	China	Beijing	SRS935981	SRP058221	R	R	R	n/a	R	R	R	R
11_2277	Yang et. al. Lancet Infect Dis. 2017 Mar;17(3):275-284	China	Beijing	SRS936031	SRP058221	R	R	R	n/a	R	R	R	S
09_1585	Yang et. al. Lancet Infect Dis. 2017 Mar;17(3):275-284	China	Beijing	SRS935958	SRP058221	R	R	R	n/a	R	R	R	S
11_0644	Yang et. al. Lancet Infect Dis. 2017 Mar;17(3):275-284	China	Beijing	SRS936018	SRP058221	R	R	S	n/a	R	R	S	S
11_0870	Yang et. al. Lancet Infect Dis. 2017 Mar;17(3):275-284	China	Beijing	SRS936039	SRP058221	R	R	R	n/a	R	R	R	R
09_0294	Yang et. al. Lancet Infect Dis. 2017 Mar;17(3):275-284	China	Beijing	SRS935959	SRP058221	R	R	S	n/a	R	R	S	R
12_1198	Yang et. al. Lancet Infect Dis. 2017 Mar;17(3):275-284	China	S-type	SRS935924	SRP058221	R	R	R	n/a	R	R	R	R
11_1430	Yang et. al. Lancet Infect Dis. 2017 Mar;17(3):275-284	China	Beijing	SRS936037	SRP058221	R	R	R	n/a	R	R	R	S
09_0007	Yang et. al. Lancet Infect Dis. 2017 Mar;17(3):275-284	China	Beijing	SRS935937	SRP058221	R	R	R	n/a	R	R	R	U
09_1011	Yang et. al. Lancet Infect Dis. 2017 Mar;17(3):275-284	China	Beijing	SRS935951	SRP058221	R	R	R	n/a	R	R	R	R
10_1628	Yang et. al. Lancet Infect Dis. 2017 Mar;17(3):275-284	China	Beijing	SRS935995	SRP058221	R	R	R	n/a	R	R	R	R
11_0431	Yang et. al. Lancet Infect Dis. 2017 Mar;17(3):275-284	China	Beijing	SRS936019	SRP058221	R	R	S	n/a	R	R	S	U
10_0592	Yang et. al. Lancet Infect Dis. 2017 Mar;17(3):275-284	China	Beijing	SRS935978	SRP058221	R	R	S	n/a	R	R	R	R
12_0701	Yang et. al. Lancet Infect Dis. 2017 Mar;17(3):275-284	China	Beijing	SRS935929	SRP058221	R	R	R	n/a	R	R	R	U
12_1810	Yang et. al. Lancet Infect Dis. 2017 Mar;17(3):275-284	China	Beijing	SRS935945	SRP058221	R	R	R	n/a	R	R	S	S
10_0360	Yang et. al. Lancet Infect Dis. 2017 Mar;17(3):275-284	China	Beijing	SRS935991	SRP058221	R	R	R	n/a	R	R	R	S
09_0645	Yang et. al. Lancet Infect Dis. 2017 Mar;17(3):275-284	China	Beijing	SRS936026	SRP058221	R	R	R	n/a	R	R	S	R
10_1232	Yang et. al. Lancet Infect Dis. 2017 Mar;17(3):275-284	China	Beijing	SRS935998	SRP058221	R	R	R	n/a	R	R	U	S
09_0682	Yang et. al. Lancet Infect Dis. 2017 Mar;17(3):275-284	China	Beijing	SRS935936	SRP058221	R	R	R	n/a	R	R	R	R
10_1603	Yang et. al. Lancet Infect Dis. 2017 Mar;17(3):275-284	China	Beijing	SRS935996	SRP058221	R	R	S	n/a	R	R	S	S
12_2164	Yang et. al. Lancet Infect Dis. 2017 Mar;17(3):275-284	China	Beijing	SRS935939	SRP058221	R	R	R	n/a	R	R	R	R
10_1781	Yang et. al. Lancet Infect Dis. 2017 Mar;17(3):275-284	China	Beijing	SRS936004	SRP058221	R	R	R	n/a	R	R	R	R
12_0429	Yang et. al. Lancet Infect Dis. 2017 Mar;17(3):275-284	China	Beijing	SRS935934	SRP058221	R	R	S	n/a	R	R	R	S
11_0697	Yang et. al. Lancet Infect Dis. 2017 Mar;17(3):275-284	China	Beijing	SRS936017	SRP058221	R	R	R	n/a	R	R	F	F
09_1121	Yang et. al. Lancet Infect Dis. 2017 Mar;17(3):275-284	China	Beijing	SRS935966	SRP058221	R	R	R	n/a	R	R	R	S
10_0600	Yang et. al. Lancet Infect Dis. 2017 Mar;17(3):275-284	China	Beijing	SRS935977	SRP058221	R	R	S	n/a	R	R	S	S
10_2232	Yang et. al. Lancet Infect Dis. 2017 Mar;17(3):275-284	China	Beijing	SRS936015	SRP058221	R	R	S	n/a	R	R	U	R
09_1924	Yang et. al. Lancet Infect Dis. 2017 Mar;17(3):275-284	China	Beijing	SRS935974	SRP058221	R	R	S	n/a	R	R	S	S
10_0297	Yang et. al. Lancet Infect Dis. 2017 Mar;17(3):275-284	China	Beijing	SRS935992	SRP058221	R	R	R	n/a	R	R	R	R
09_1928	Yang et. al. Lancet Infect Dis. 2017 Mar;17(3):275-284	China	Beijing	SRS935973	SRP058221	R	R	S	n/a	R	R	S	S
09_0647	Yang et. al. Lancet Infect Dis. 2017 Mar;17(3):275-284	China	Beijing	SRS936040	SRP058221	R	R	S	n/a	R	R	R	S
11_0293	Yang et. al. Lancet Infect Dis. 2017 Mar;17(3):275-284	China	Beijing	SRS936024	SRP058221	R	R	R	n/a	R	R	R	S
09_0116	Yang et. al. Lancet Infect Dis. 2017 Mar;17(3):275-284	China	Beijing	SRS935948	SRP058221	R	R	S	n/a	R	R	S	S
10_1770	Yang et. al. Lancet Infect Dis. 2017 Mar;17(3):275-284	China	Beijing	SRS936005	SRP058221	R	R	R	n/a	R	R	R	R
11_0867	Yang et. al. Lancet Infect Dis. 2017 Mar;17(3):275-284	China	Beijing	SRS936041	SRP058221	R	R	S	n/a	R	R	S	S
10_1647	Yang et. al. Lancet Infect Dis. 2017 Mar;17(3):275-284	China	Beijing	SRS936007	SRP058221	R	R	R	n/a	R	R	R	S
10_0183	Yang et. al. Lancet Infect Dis. 2017 Mar;17(3):275-284	China	Beijing	SRS935968	SRP058221	R	R	R	n/a	R	R	R	R
09_0004	Yang et. al. Lancet Infect Dis. 2017 Mar;17(3):275-284	China	Beijing	SRS935938	SRP058221	R	R	S	n/a	R	R	S	R
10_0208	Yang et. al. Lancet Infect Dis. 2017 Mar;17(3):275-284	China	Tur	SRS935993	SRP058221	R	R	S	n/a	R	R	S	R
10_1007	Yang et. al. Lancet Infect Dis. 2017 Mar;17(3):275-284	China	Beijing	SRS936010	SRP058221	R	R	R	n/a	R	R	R	R
12_0779	Yang et. al. Lancet Infect Dis. 2017 Mar;17(3):275-284	China	Beijing	SRS935927	SRP058221	R	R	R	n/a	R	R	R	R
09_1098	Yang et. al. Lancet Infect Dis. 2017 Mar;17(3):275-284	China	Beijing	SRS935947	SRP058221	R	R	R	n/a	R	R	R	U
09_0783	Yang et. al. Lancet Infect Dis. 2017 Mar;17(3):275-284	China	S-type	SRS935941	SRP058221	R	R	R	n/a	R	R	R	R
09_1060	Yang et. al. Lancet Infect Dis. 2017 Mar;17(3):275-284	China	Tur	SRS935949	SRP058221	R	R	R	n/a	R	R	R	R
12_0242	Yang et. al. Lancet Infect Dis. 2017 Mar;17(3):275-284	China	Beijing	SRS936034	SRP058221	R	R	R	n/a	R	R	R	S
09_0956	Yang et. al. Lancet Infect Dis. 2017 Mar;17(3):275-284	China	Beijing	SRS935953	SRP058221	R	R	S	n/a	R	R	S	S
09_1543	Yang et. al. Lancet Infect Dis. 2017 Mar;17(3):275-284	China	Beijing	SRS935961	SRP058221	R	R	R	n/a	R	R	S	R
12_2121	Yang et. al. Lancet Infect Dis. 2017 Mar;17(3):275-284	China	Beijing	SRS935940	SRP058221	R	R	R	n/a	R	R	R	S
09_1578	Yang et. al. Lancet Infect Dis. 2017 Mar;17(3):275-284	China	Beijing	SRS935963	SRP058221	R	R	R	n/a	R	R	R	U
12_0715	Yang et. al. Lancet Infect Dis. 2017 Mar;17(3):275-284	China	Beijing	SRS935928	SRP058221	R	R	S	n/a	R	R	S	R
11_0426	Yang et. al. Lancet Infect Dis. 2017 Mar;17(3):275-284	China	Beijing	SRS936020	SRP058221	R	R	S	n/a	R	R	S	S
10_2010	Yang et. al. Lancet Infect Dis. 2017 Mar;17(3):275-284	China		SRS936001	SRP058221	R	R	R	n/a	R	R	F	F
09_0799	Yang et. al. Lancet Infect Dis. 2017 Mar;17(3):275-284	China	Beijing	SRS935955	SRP058221	R	R	R	n/a	R	R	R	S

09_0716	Yang et. al. Lancet Infect Dis. 2017 Mar;17(3):275-284	China	Beijing	SRS935926	SRP058221	R	R	S	n/a	R	R	S	S
12_0583	Yang et. al. Lancet Infect Dis. 2017 Mar;17(3):275-284	China	Beijing	SRS935920	SRP058221	R	R	R	n/a	R	R	R	S
BGI_252	Zhang et. al. Nat Genet. 2013 Oct;45(10):1255-60	China	Beijing	SRR671867		R	R	S	n/a	R	R	U	R
BGI_HEN06035	Zhang et. al. Nat Genet. 2013 Oct;45(10):1255-60	China	Beijing	SRR671856		S	S	S	n/a	S	S	S	S
BGI_B-160	Zhang et. al. Nat Genet. 2013 Oct;45(10):1255-60	China	Beijing	SRR671723		S	S	S	n/a	S	R	S	S
BGI_LN228	Zhang et. al. Nat Genet. 2013 Oct;45(10):1255-60	China	Beijing	SRR671796		R	R	R	n/a	R	R	R	U
BGI_J-xz09105	Zhang et. al. Nat Genet. 2013 Oct;45(10):1255-60	China	Beijing	SRR671735		R	R	S	n/a	R	R	U	S
BGI_SH171	Zhang et. al. Nat Genet. 2013 Oct;45(10):1255-60	China	Beijing	SRR671808		S	S	S	n/a	S	S	S	S
BGI_GuangZ8	Zhang et. al. Nat Genet. 2013 Oct;45(10):1255-60	China	Beijing	SRR671726		R	R	R	n/a	R	R	R	R
BGI_GZ066	Zhang et. al. Nat Genet. 2013 Oct;45(10):1255-60	China	Beijing	SRR671843		R	R	S	n/a	R	R	U	S
BGI_I-xz09061	Zhang et. al. Nat Genet. 2013 Oct;45(10):1255-60	China	Beijing	SRR671734		R	R	S	n/a	R	R	S	S
BGI_140	Zhang et. al. Nat Genet. 2013 Oct;45(10):1255-60	China	Beijing	SRR671722		R	R	S	n/a	R	R	U	R
BGI_FJ05189	Zhang et. al. Nat Genet. 2013 Oct;45(10):1255-60	China	lineage4	SRR671757		R	R	S	n/a	U	R	R	R
BGI_GuangZ0016	Zhang et. al. Nat Genet. 2013 Oct;45(10):1255-60	China	S-type	SRR671861		R	R	S	n/a	R	R	R	R
BGI_GuangZ0026	Zhang et. al. Nat Genet. 2013 Oct;45(10):1255-60	China	S-type	SRR671862		R	R	R	n/a	R	R	U	R
BGI_XJ06007	Zhang et. al. Nat Genet. 2013 Oct;45(10):1255-60	China	Beijing	SRR671871		S	S	S	n/a	S	S	S	S
BGI_GZ119	Zhang et. al. Nat Genet. 2013 Oct;45(10):1255-60	China	Beijing	SRR671837		R	R	R	n/a	R	R	R	U
BGI_LN373	Zhang et. al. Nat Genet. 2013 Oct;45(10):1255-60	China	Beijing	SRR671854		R	R	R	n/a	R	R	F	U
BGI_144	Zhang et. al. Nat Genet. 2013 Oct;45(10):1255-60	China	Beijing	SRR671869		R	R	S	n/a	R	R	U	U
BGI_LN236	Zhang et. al. Nat Genet. 2013 Oct;45(10):1255-60	China	Beijing	SRR671799		R	R	R	n/a	R	R	U	R
BGI_XZ06030	Zhang et. al. Nat Genet. 2013 Oct;45(10):1255-60	China	lineage4	SRR671838		R	R	S	n/a	R	R	F	S
BGI_SH166	Zhang et. al. Nat Genet. 2013 Oct;45(10):1255-60	China	Beijing	SRR671806		R	R	R	n/a	R	R	R	F
BGI_FJ07070	Zhang et. al. Nat Genet. 2013 Oct;45(10):1255-60	China	Beijing	SRR671842		R	R	R	n/a	R	R	R	R
BGI_SH311	Zhang et. al. Nat Genet. 2013 Oct;45(10):1255-60	China	Beijing	SRR671815		R	R	R	n/a	R	R	R	R
BGI_NM11008	Zhang et. al. Nat Genet. 2013 Oct;45(10):1255-60	China	Beijing	SRR671870		S	S	S	n/a	S	S	S	S
BGI_SH327	Zhang et. al. Nat Genet. 2013 Oct;45(10):1255-60	China	Beijing	SRR671816		R	R	S	n/a	R	R	F	R
BGI_NM11014	Zhang et. al. Nat Genet. 2013 Oct;45(10):1255-60	China	Beijing	SRR671876		S	S	S	n/a	S	S	S	S
BGI_U-xz09006	Zhang et. al. Nat Genet. 2013 Oct;45(10):1255-60	China	Beijing	SRR671745		R	R	S	n/a	R	R	S	S
BGI_L-xz09102	Zhang et. al. Nat Genet. 2013 Oct;45(10):1255-60	China	Beijing	SRR671736		R	R	S	n/a	R	R	U	S
BGI_SHX05178	Zhang et. al. Nat Genet. 2013 Oct;45(10):1255-60	China	lineage4	SRR671853		R	R	R	n/a	U	R	R	S
BGI_FJ05136	Zhang et. al. Nat Genet. 2013 Oct;45(10):1255-60	China	Beijing	SRR671752		R	R	S	n/a	R	R	R	F
BGI_XJ06090	Zhang et. al. Nat Genet. 2013 Oct;45(10):1255-60	China	lineage4	SRR671874		S	S	S	n/a	S	S	S	S
BGI_LN270	Zhang et. al. Nat Genet. 2013 Oct;45(10):1255-60	China	Beijing	SRR671801		R	R	S	n/a	R	R	R	S
BGI_BJ05026	Zhang et. al. Nat Genet. 2013 Oct;45(10):1255-60	China	lineage4	SRR671859		S	S	S	n/a	S	S	S	S
BGI_GZ10125	Zhang et. al. Nat Genet. 2013 Oct;45(10):1255-60	China	lineage4	SRR671768		R	R	S	n/a	R	R	S	S
BGI_V-xz09011	Zhang et. al. Nat Genet. 2013 Oct;45(10):1255-60	China	Beijing	SRR671746		R	R	S	n/a	R	R	S	S
BGI_XZ06017	Zhang et. al. Nat Genet. 2013 Oct;45(10):1255-60	China	Beijing	SRR671848		R	R	S	n/a	R	R	U	S
BGI_XZ06217	Zhang et. al. Nat Genet. 2013 Oct;45(10):1255-60	China	Beijing	SRR671840		R	R	R	n/a	R	R	U	S
BGI_Q-xz09122	Zhang et. al. Nat Genet. 2013 Oct;45(10):1255-60	China	Beijing	SRR671742		R	R	S	n/a	R	R	R	F
BGI_Y-xz09033	Zhang et. al. Nat Genet. 2013 Oct;45(10):1255-60	China	Beijing	SRR671747		S	S	S	n/a	S	S	S	S
BGI_336	Zhang et. al. Nat Genet. 2013 Oct;45(10):1255-60	China	Beijing	SRR671866		R	R	R	n/a	R	R	R	R
BGI_SH361	Zhang et. al. Nat Genet. 2013 Oct;45(10):1255-60	China	Beijing	SRR671820		R	R	S	n/a	R	R	R	S
BGI_NM11011	Zhang et. al. Nat Genet. 2013 Oct;45(10):1255-60	China	Beijing	SRR671877		S	S	S	n/a	S	S	S	S
BGI_ShX127	Zhang et. al. Nat Genet. 2013 Oct;45(10):1255-60	China	Beijing	SRR671795		R	R	S	n/a	R	R	R	S
BGI_SH292	Zhang et. al. Nat Genet. 2013 Oct;45(10):1255-60	China	Beijing	SRR671813		R	R	R	n/a	U	R	R	U
BGI_A-xz09095	Zhang et. al. Nat Genet. 2013 Oct;45(10):1255-60	China	Beijing	SRR671727		R	R	S	n/a	U	R	F	F
BGI_40195	Zhang et. al. Nat Genet. 2013 Oct;45(10):1255-60	China	Beijing	SRR671780		R	R	S	n/a	F	R	F	S
BGI_GuangZ0017	Zhang et. al. Nat Genet. 2013 Oct;45(10):1255-60	China	Beijing	SRR671864		R	R	R	n/a	R	R	R	R
BGI_X-xz09017	Zhang et. al. Nat Genet. 2013 Oct;45(10):1255-60	China	Beijing	SRR671738		R	R	S	n/a	R	R	R	S
BGI_GZ104	Zhang et. al. Nat Genet. 2013 Oct;45(10):1255-60	China	Beijing	SRR671845		R	R	R	n/a	U	R	R	S
BGI_ShX519	Zhang et. al. Nat Genet. 2013 Oct;45(10):1255-60	China	Beijing	SRR671798		R	R	S	n/a	R	R	U	S
BGI_P-xz09119	Zhang et. al. Nat Genet. 2013 Oct;45(10):1255-60	China	Delhi	SRR671741		S	S	S	n/a	S	S	S	S
BGI_SH390	Zhang et. al. Nat Genet. 2013 Oct;45(10):1255-60	China	Beijing	SRR671822		R	R	R	n/a	R	R	R	R
BGI_LN151	Zhang et. al. Nat Genet. 2013 Oct;45(10):1255-60	China	Beijing	SRR671792		S	S	S	n/a	S	S	S	S
BGI_GZ10115	Zhang et. al. Nat Genet. 2013 Oct;45(10):1255-60	China	Beijing	SRR671771		R	R	R	n/a	R	R	R	U
BGI_315	Zhang et. al. Nat Genet. 2013 Oct;45(10):1255-60	China	Beijing	SRR671724		R	R	R	n/a	R	R	R	R
BGI_SH287	Zhang et. al. Nat Genet. 2013 Oct;45(10):1255-60	China	Beijing	SRR671812		R	R	S	n/a	R	R	R	U
BGI_FJ05121	Zhang et. al. Nat Genet. 2013 Oct;45(10):1255-60	China	lineage4	SRR671750		R	R	S	n/a	R	R	R	F
BGI_FJ05132	Zhang et. al. Nat Genet. 2013 Oct;45(10):1255-60	China	lineage4	SRR671751		R	R	R	n/a	R	R	R	R
BGI_FJ05141	Zhang et. al. Nat Genet. 2013 Oct;45(10):1255-60	China	Beijing	SRR671841		R	R	R	n/a	R	R	R	F
BGI_R-xz09105	Zhang et. al. Nat Genet. 2013 Oct;45(10):1255-60	China	Beijing	SRR671743		R	R	S	n/a	R	R	U	S
BGI_FJ05112	Zhang et. al. Nat Genet. 2013 Oct;45(10):1255-60	China	Beijing	SRR671748		R	R	R	n/a	R	R	F	R
BGI_10	Zhang et. al. Nat Genet. 2013 Oct;45(10):1255-60	China	LAM	SRR671775		S	S	S	n/a	S	S	S	S
BGI_LN175	Zhang et. al. Nat Genet. 2013 Oct;45(10):1255-60	China	Beijing	SRR671797		R	R	R	n/a	R	R	R	R
BGI_FJ05474	Zhang et. al. Nat Genet. 2013 Oct;45(10):1255-60	China	S-type	SRR671764		S	S	S	n/a	S	S	S	S
BGI_LN007	Zhang et. al. Nat Genet. 2013 Oct;45(10):1255-60	China	Beijing	SRR671788		S	S	S	n/a	U	S	U	S
BGI_GuangZ29	Zhang et. al. Nat Genet. 2013 Oct;45(10):1255-60	China	S-type	SRR671790		R	R	R	n/a	S	U	S	S
BGI_XZ06194	Zhang et. al. Nat Genet. 2013 Oct;45(10):1255-60	China	Beijing	SRR671851		R	R	R	n/a	R	R	R	R
BGI_322	Zhang et. al. Nat Genet. 2013 Oct;45(10):1255-60	China	Beijing	SRR671720		R	R	R	n/a	R	R	R	S
BGI_GZ077	Zhang et. al. Nat Genet. 2013 Oct;45(10):1255-60	China	Beijing	SRR671834		R	R	R	n/a	R	R	S	S
BGI_SH396	Zhang et. al. Nat Genet. 2013 Oct;45(10):1255-60	China	Beijing	SRR671823		S	S	S	n/a	S	S	S	S
BGI_LN018	Zhang et. al. Nat Genet. 2013 Oct;45(10):1255-60	China	Beijing	SRR671855		R	R	R	n/a	R	R	U	R
BGI_LN230	Zhang et. al. Nat Genet. 2013 Oct;45(10):1255-60	China	Beijing	SRR671829		R	R	S	n/a	R	R	S	S
BGI_GuangZ0008	Zhang et. al. Nat Genet. 2013 Oct;45(10):1255-60	China	Beijing	SRR671863		R	R	R	n/a	R	R	R	R
BGI_XZ06055	Zhang et. al. Nat Genet. 2013 Oct;45(10):1255-60	China	lineage4	SRR671850		R	R	R	n/a	R	R	R	U
BGI_GuangZ6	Zhang et. al. Nat Genet. 2013 Oct;45(10):1255-60	China	S-type	SRR671725		R	R	R	n/a	R	R	R	R
BGI_HEN05034	Zhang et. al. Nat Genet. 2013 Oct;45(10):1255-60	China	Beijing	SRR671857		R	R	R	n/a	R	R	S	S
BGI_LN077	Zhang et. al. Nat Genet. 2013 Oct;45(10):1255-60	China	Beijing	SRR671787		R	R	R	n/a	R	R	R	R
BGI_FJ05060	Zhang et. al. Nat Genet. 2013 Oct;45(10):1255-60	China	S-type	SRR671762		S	S	S	n/a	S	S	U	S
BGI_G-xz09148	Zhang et. al. Nat Genet. 2013 Oct;45(10):1255-60	China	Beijing	SRR671732		R	R	R	n/a	R	R	S	S
BGI_XJ06017	Zhang et. al. Nat Genet. 2013 Oct;45(10):1255-60	China	S-type	SRR671878		S	S	S	n/a	S	S	S	S
BGI_GZ10108	Zhang et. al. Nat Genet. 2013 Oct;45(10):1255-60	China	Beijing	SRR671765		R	R	R	n/a	R	R	R	S
BGI_GZ10145	Zhang et. al. Nat Genet. 2013 Oct;45(10):1255-60	China	lineage4	SRR671773		S	S	S	n/a	S	S	S	S
BGI_SH068	Zhang et. al. Nat Genet. 2013 Oct;45(10):1255-60	China	Beijing	SRR671811		R	R	R	n/a	R	R	R	R
BGI_SH404	Zhang et. al. Nat Genet. 2013 Oct;45(10):1255-60	China	Beijing	SRR671825		R	R	R	n/a	R	R	S	R
BGI_SH002	Zhang et. al. Nat Genet. 2013 Oct;45(10):1255-60	China	Beijing	SRR671804		R	R	R	n/a	R	R	R	F
BGI_SH060	Zhang et. al. Nat Genet. 2013 Oct;45(10):1255-60	China	Beijing	SRR671810		R	R	R	n/a	R	R	R	R
BGI_GZ126	Zhang et. al. Nat Genet. 2013 Oct;45(10):1255-60	China	lineage4	SRR671846		S	S	S	n/a	S	S	S	S
BGI_BJ05010	Zhang et. al. Nat Genet. 2013 Oct;45(10):1255-60	China	Beijing	SRR671858		S	S	S	n/a	S	S	S	S
BGI_ShX477	Zhang et. al. Nat Genet. 2013 Oct;45(10):1255-60	China	Beijing	SRR671793		S	S	S	n/a	S	S	S	S
BGI_FJ09132	Zhang et. al. Nat Genet. 2013 Oct;45(10):1255-60	China	Beijing	SRR671754		R	R	R	n/a	R	R	R	F
BGI_GuangZ24	Zhang et. al. Nat Genet. 2013 Oct;45(10):1255-60	China	Beijing	SRR671789		R	R	S	n/a	R	R	R	R
BGI_FJ09114	Zhang et. al. Nat Genet. 2013 Oct;45(10):1255-60	China	Beijing	SRR671756		R	R	R	n/a	R	R	R	R
BGI_GZ10059	Zhang et. al. Nat Genet. 2013 Oct;45(10):1255-60	China	lineage4	SRR671769		R	R	R	n/a	R	R	R	S
BGI_E-xz09109	Zhang et. al. Nat Genet. 2013 Oct;45(10):1255-60	China	Beijing	SRR671730		S	S	S	n/a	S	S	S	S
BGI_SH298	Zhang et. al. Nat Genet. 2013 Oct;45(10):1255-60	China	Beijing	SRR671814		S	S	S	n/a	S	S	S	S
BGI_143	Zhang et. al. Nat Genet. 2013 Oct;45(10):1255-60	China	lineage4	SRR671868		R	R	R	n/a	R	R	U	F
BGI_SH147	Zhang et. al. Nat Genet. 2013 Oct;45(10):1255-60	China	Beijing	SRR671805		S	S	S	n/a	S	S	S	S
BGI_GZ113	Zhang et. al. Nat Genet. 2013 Oct;45(10):1255-60	China	Beijing	SRR671836		R	R	S	n/a	U	S	S	S
BGI_SH400	Zhang et. al. Nat Genet. 2013 Oct;45(10):1255-60	China	Beijing	SRR671824		R	R	S	n/a	R	R	R	R
BGI_BJ05043	Zhang et. al. Nat Genet. 2013 Oct;45(10):1255-60	China	S-type	SRR671860		S	S	S	n/a	S	S	U	S
BGI_SH415	Zhang et. al. Nat Genet. 2013 Oct;45(10):1255-60	China	Beijing	SRR671826		R	R	R	n/a	R	R	R	R
BGI_SH340	Zhang et. al. Nat Genet. 2013 Oct;45(10):1255-60	China	Beijing	SRR671819		R	R	S	n/a	R	R	R	S
BGI_SH186	Zhang et. al. Nat Genet. 2013 Oct;45(10):1255-60	China	Beijing	SRR671809		R	R	R	n/a	R	R	R	S
BGI_H-xz09043	Zhang et. al. Nat Genet. 2013 Oct;45(10):1255-60	China	Beijing	SRR671733		R	R	S	n/a	R	R	R	S
BGI_D-xz06169	Zhang et. al. Nat Genet. 2013 Oct;45(10):1255-60	China	Beijing	SRR671729		S	S	S	n/a	S	S	S	S
BGI_LN005	Zhang et. al. Nat Genet. 2013 Oct;45(10):1255-60	China	Beijing	SRR671784		R	R	S	n/a	R	R	S	S
BGI_SHX669	Zhang et. al. Nat Genet. 2013 Oct;45(10):1255-60	China	Beijing	SRR671852		R	R	S	n/a	R	R	R	R
BGI_M-xz06013	Zhang et. al. Nat Genet. 2013 Oct;45(10):1255-60	China	Beijing	SRR671737		R	R	R	n/a	R	R	R	R
BGI_ShX321	Zhang et. al. Nat Genet. 2013 Oct;45(10):1255-60	China	S-type	SRR671831		R	R	S	n/a	R	R	R	R
BGI_42063	Zhang et. al. Nat Genet. 2013 Oct;45(10):1255-60	China	Beijing	SRR671777		S	S	S	n/a	F	S	S	S
BGI_SH170	Zhang et. al. Nat Genet. 2013 Oct;45(10):1255-60	China	Beijing	SRR671807		R	R	R	n/a	R	R	R	R
BGI_XZ06044	Zhang et. al. Nat Genet. 2013 Oct;45(10):1255-60	China	Beijing	SRR671849		R	R	R	n/a	R	F	F	F
BGI_LN161	Zhang et. al. Nat Genet. 2013 Oct;45(10):1255-60	China	Beijing	SRR671800		S	S	S	n/a	S	S	S	S
BGI_LN081	Zhang et. al. Nat Genet. 2013 Oct;45(10):1255-60	China	Beijing	SRR671785		R	R	R	n/a	R	R	R	R
BGI_N-xz06042	Zhang et. al. Nat Genet. 2013 Oct;45(10):1255-60	China	Beijing	SRR671739		R	R	R	n/a	R	R	R	R

BGI_FJ05120	Zhang et. al. Nat Genet. 2013 Oct;45(10):1255-60	China	S-type	SRR671749	R	R	S	n/a	R	R	U	S
BGI_42065	Zhang et. al. Nat Genet. 2013 Oct;45(10):1255-60	China	S-type	SRR671778	S	S	S	n/a	F	S	S	F
BGI_SH51	Zhang et. al. Nat Genet. 2013 Oct;45(10):1255-60	China	Beijing	SRR671828	R	R	S	n/a	R	R	R	R
BGI_C-xz06202	Zhang et. al. Nat Genet. 2013 Oct;45(10):1255-60	China	Beijing	SRR671728	R	R	R	n/a	U	R	R	F
BGI_GZ146	Zhang et. al. Nat Genet. 2013 Oct;45(10):1255-60	China	Beijing	SRR671847	R	R	S	n/a	S	F	U	S
BGI_GZ10109	Zhang et. al. Nat Genet. 2013 Oct;45(10):1255-60	China	Beijing	SRR671844	R	R	R	n/a	R	R	R	R
BGI_LN037	Zhang et. al. Nat Genet. 2013 Oct;45(10):1255-60	China	Beijing	SRR671786	S	S	S	n/a	S	S	S	S
BGI_GZ061	Zhang et. al. Nat Genet. 2013 Oct;45(10):1255-60	China	lineage4	SRR671833	S	S	S	n/a	S	S	U	S
BGI_ShX347	Zhang et. al. Nat Genet. 2013 Oct;45(10):1255-60	China	Beijing	SRR671802	S	S	S	n/a	S	S	U	S
BGI_FJ07036	Zhang et. al. Nat Genet. 2013 Oct;45(10):1255-60	China	Beijing	SRR671755	R	R	S	n/a	R	R	R	U
BGI_FJ05195	Zhang et. al. Nat Genet. 2013 Oct;45(10):1255-60	China	Beijing	SRR671759	R	R	R	n/a	R	R	R	U
BGI_GZ10057	Zhang et. al. Nat Genet. 2013 Oct;45(10):1255-60	China	S-type	SRR671835	S	S	S	n/a	S	S	S	S
BGI_SH337	Zhang et. al. Nat Genet. 2013 Oct;45(10):1255-60	China	Beijing	SRR671818	R	R	R	n/a	R	R	R	R
BGI_GZ10120	Zhang et. al. Nat Genet. 2013 Oct;45(10):1255-60	China	lineage4	SRR671766	R	R	R	n/a	R	R	R	S
BGI_FJ05148	Zhang et. al. Nat Genet. 2013 Oct;45(10):1255-60	China	Beijing	SRR671753	R	R	S	n/a	R	R	F	S
BGI_293	Zhang et. al. Nat Genet. 2013 Oct;45(10):1255-60	China	Beijing	SRR671865	R	R	R	n/a	R	R	R	R
BGI_XZ06050	Zhang et. al. Nat Genet. 2013 Oct;45(10):1255-60	China	Beijing	SRR671839	R	R	R	n/a	R	R	R	R
BGI_42061	Zhang et. al. Nat Genet. 2013 Oct;45(10):1255-60	China	Beijing	SRR671776	S	S	S	n/a	F	S	S	F
BGI_LN023	Zhang et. al. Nat Genet. 2013 Oct;45(10):1255-60	China	Beijing	SRR671781	R	R	R	n/a	R	R	U	R
BGI_LN130	Zhang et. al. Nat Genet. 2013 Oct;45(10):1255-60	China	Beijing	SRR671794	R	R	R	n/a	R	R	U	R
BGI_NM11010	Zhang et. al. Nat Genet. 2013 Oct;45(10):1255-60	China	Beijing	SRR671875	S	S	S	n/a	S	S	S	S
BGI_FJ05179	Zhang et. al. Nat Genet. 2013 Oct;45(10):1255-60	China	Beijing	SRR671758	R	R	S	n/a	F	R	R	S
BGI_SH045	Zhang et. al. Nat Genet. 2013 Oct;45(10):1255-60	China	Beijing	SRR671827	S	S	S	n/a	S	S	S	S
BGI_41310	Zhang et. al. Nat Genet. 2013 Oct;45(10):1255-60	China	Beijing	SRR671779	R	R	S	n/a	R	R	R	R
BGI_FJ09143	Zhang et. al. Nat Genet. 2013 Oct;45(10):1255-60	China	Beijing	SRR671761	R	R	R	n/a	R	R	R	S
BGI_GZ10116	Zhang et. al. Nat Genet. 2013 Oct;45(10):1255-60	China	lineage4	SRR671774	R	R	S	n/a	R	R	R	S
BGI_GZ10103	Zhang et. al. Nat Genet. 2013 Oct;45(10):1255-60	China	lineage4	SRR671767	R	R	R	n/a	R	R	R	U
BGI_GuangZ28	Zhang et. al. Nat Genet. 2013 Oct;45(10):1255-60	China	Beijing	SRR671783	R	R	R	n/a	R	S	U	S
BGI_ShX141	Zhang et. al. Nat Genet. 2013 Oct;45(10):1255-60	China	Beijing	SRR671830	S	S	S	n/a	S	S	S	S
BGI_GZ10102	Zhang et. al. Nat Genet. 2013 Oct;45(10):1255-60	China	Beijing	SRR671770	R	R	R	n/a	R	R	R	R
BGI_FJ07028	Zhang et. al. Nat Genet. 2013 Oct;45(10):1255-60	China	S-type	SRR671760	R	R	S	n/a	U	R	R	R
BGI_XJ06055	Zhang et. al. Nat Genet. 2013 Oct;45(10):1255-60	China	lineage4	SRR671872	S	S	S	n/a	S	S	S	S
BGI_LN314	Zhang et. al. Nat Genet. 2013 Oct;45(10):1255-60	China	Beijing	SRR671791	R	R	S	n/a	R	R	S	R
BGI_S-xz09021	Zhang et. al. Nat Genet. 2013 Oct;45(10):1255-60	China	Beijing	SRR671744	R	R	S	n/a	R	R	R	S
BGI_60	Zhang et. al. Nat Genet. 2013 Oct;45(10):1255-60	China	Beijing	SRR671719	R	R	R	n/a	R	R	S	S
BGI_SH331	Zhang et. al. Nat Genet. 2013 Oct;45(10):1255-60	China	Beijing	SRR671817	R	R	S	n/a	R	R	S	R
BGI_SH384	Zhang et. al. Nat Genet. 2013 Oct;45(10):1255-60	China	Beijing	SRR671821	R	R	R	n/a	R	R	U	R
BGI_F-xz09121	Zhang et. al. Nat Genet. 2013 Oct;45(10):1255-60	China	Beijing	SRR671731	R	R	S	n/a	R	R	R	S
BGI_FJ05349	Zhang et. al. Nat Genet. 2013 Oct;45(10):1255-60	China	lineage4	SRR671763	S	S	S	n/a	S	S	U	S
BGI_FJ05395	Zhang et. al. Nat Genet. 2013 Oct;45(10):1255-60	China	Tur	SRR671832	S	S	S	n/a	S	S	S	S
BGI_XJ06014	Zhang et. al. Nat Genet. 2013 Oct;45(10):1255-60	China	Delhi	SRR671873	S	S	S	n/a	S	S	S	S
BGI_O-xz06008	Zhang et. al. Nat Genet. 2013 Oct;45(10):1255-60	China	S-type	SRR671740	R	R	S	n/a	U	R	R	R
BGI_NM11006	Zhang et. al. Nat Genet. 2013 Oct;45(10):1255-60	China	Beijing	SRR671879	S	S	S	n/a	S	S	S	S
BGI_142	Zhang et. al. Nat Genet. 2013 Oct;45(10):1255-60	China	Beijing	SRR671721	R	R	R	n/a	R	R	R	R
BGI_ShX640	Zhang et. al. Nat Genet. 2013 Oct;45(10):1255-60	China	Beijing	SRR671803	S	S	S	n/a	S	S	S	S
12848-13	Hamburg	Germany	Delhi	ERR2199821	R	S	S	S	S	S	S	S
108-14	Hamburg	Germany	Haarlem	ERR2199781	S	S	S	S	S	S	S	S
10078-13	Hamburg	Germany	Haarlem	ERR2199746	S	S	S	S	S	S	S	S
3936-02	Hamburg	Germany	Beijing	ERR551572	S	S	S	S	S	R	S	S
9652-13	Hamburg	Germany	Delhi	ERR2200140	S	S	S	S	S	S	S	S
4502-14	Hamburg	Germany	Cameroon	ERR2199963	S	S	S	S	S	S	S	S
10459-03	Hamburg	Germany	lineage4	ERR551584 ERR	S	S	S	S	S	S	S	S
10264-03	Hamburg	Germany	Tur	ERR552332 ERR	S	S	S	S	S	S	S	S
10393-14	Hamburg	Germany	LAM	ERR2199762 ER	R	R	S	S	R	R	S	S
5192-14	Hamburg	Germany	Delhi	ERR2200012	S	S	S	S	S	S	S	S
4092-13	Hamburg	Germany	lineage4	ERR2199915	S	S	S	S	S	S	U	S
4880-01	Hamburg	Germany	Haarlem	ERR551674 ERR	S	S	S	S	S	S	S	S
1908-15	Hamburg	Germany	Haarlem	ERR2199849	S	S	S	S	S	S	S	S
9139-14	Hamburg	Germany	Tur	ERR2200128 ER	S	S	S	S	S	S	S	S
2533-15	Hamburg	Germany	Haarlem	ERR2199862 ER	S	S	S	S	S	S	S	S
11734-02	Hamburg	Germany	Haarlem	ERR551288	S	S	S	S	S	S	S	S
3883-14	Hamburg	Germany	Haarlem	ERR2199912	S	S	S	S	S	S	S	S
1084-13	Hamburg	Germany	S-type	ERR2199783	S	S	S	S	S	S	S	S
12849-13	Hamburg	Germany	S-type	ERR2199822	S	S	S	S	S	S	S	S
947-01	Hamburg	Germany	EAI	ERR551865 ERR	S	S	S	S	S	S	S	S
8753-00	Hamburg	Germany	M. microti	ERR2200122 ER	S	S	S	S	U	S	U	S
11435-14	Hamburg	Germany	M. bovis	ERR2199797	S	S	S	S	S	U	S	R
10038-14	Hamburg	Germany	Haarlem	ERR2199743	S	S	S	S	U	S	S	S
10759-13	Hamburg	Germany	Beijing	ERR2199779	R	R	R	R	R	R	R	R
7742-14	Hamburg	Germany	lineage4	ERR2200106	S	S	S	S	S	S	S	S
12508-13	Hamburg	Germany	LAM	ERR2199817	S	S	S	S	S	S	S	S
9746-13	Hamburg	Germany	lineage4	ERR2200141	S	S	S	S	S	S	S	S
6339-13	Hamburg	Germany	Delhi	ERR2200081	S	S	S	S	S	S	S	S
2866-13	Hamburg	Germany	Delhi	ERR2199880 ER	S	S	S	S	S	S	S	S
6713-13	Hamburg	Germany	lineage4	ERR2200089	S	S	S	S	S	S	S	S
4855-13	Hamburg	Germany	Ghana	ERR2199984 ER	S	S	S	S	S	S	S	S
3118-13	Hamburg	Germany	lineage4	ERR2199893	R	S	S	S	R	S	U	S
3674-14	Hamburg	Germany	Delhi	ERR2199904	S	S	S	S	S	S	S	S
1856-13	Hamburg	Germany	lineage4	ERR2199846	S	S	S	S	S	S	S	S
2988-14	Hamburg	Germany	Haarlem	ERR2199887	S	S	S	S	S	S	S	S
4498-13	Hamburg	Germany	LAM	ERR2199962	S	S	S	S	S	S	U	S
1144-15	Hamburg	Germany	Delhi	ERR2199798	S	S	S	S	S	S	U	S
998-15	Hamburg	Germany	EAI	ERR2200150 ER	S	S	S	S	S	S	S	S
5551-14	Hamburg	Germany	Haarlem	ERR2200036	S	S	S	S	S	S	S	S
8798-14	Hamburg	Germany	lineage4	ERR2200124	S	S	S	S	S	S	S	S
9563-14	Hamburg	Germany	Beijing	ERR2200137 ER	R	R	R	R	R	R	R	R
2095-13	Hamburg	Germany	lineage4	ERR553293	S	S	S	S	S	S	S	S
934-13	Hamburg	Germany	S-type	ERR2200132	S	S	S	S	S	S	S	S
1858-13	Hamburg	Germany	lineage4	ERR2199847	S	R	S	S	S	R	S	S
4508-14	Hamburg	Germany	lineage4	ERR2199964	S	S	S	S	S	S	S	S
132-15	Hamburg	Germany	Beijing	ERR1768629	R	R	R	R	R	R	R	R
10582-13	Hamburg	Germany	Delhi	ERR2199773	S	S	S	S	S	S	U	S
9002-01	Hamburg	Germany	Haarlem	ERR551315 ERR	S	S	S	S	S	S	S	S
2387-04	Hamburg	Germany	Haarlem	ERR498357 ERR	S	S	S	S	S	S	S	S
378-14	Hamburg	Germany	Beijing	ERR2199905	S	S	S	S	S	S	S	S
2319-03	Hamburg	Germany	LAM	ERR552251 ERR	S	S	S	S	S	S	S	S
8753-14	Hamburg	Germany	Haarlem	ERR2200123	S	S	S	S	S	S	U	S
8454-14	Hamburg	Germany	Haarlem	ERR2200116	S	S	S	S	S	S	S	S
4201-13	Hamburg	Germany	lineage4	ERR2199917	S	S	S	S	S	S	S	S
8032-14	Hamburg	Germany	Cameroon	ERR2200109	S	S	S	S	S	S	S	S
4155-03	Hamburg	Germany	Haarlem	ERR498287 ERR	S	S	S	S	S	S	S	S
8506-04	Hamburg	Germany	Haarlem	ERR498367 ERR	S	S	S	S	S	S	S	S
2587-14	Hamburg	Germany	LAM	ERR2199871	S	S	S	S	S	S	U	S
9055-13	Hamburg	Germany	lineage4	ERR2200127	S	S	S	S	S	S	S	S
2551-14	Hamburg	Germany	Ural	ERR2199865	S	S	S	S	S	S	S	S
6631-04	Hamburg	Germany	Haarlem	ERR498363 ERR	S	S	S	S	S	S	S	S
5481-14	Hamburg	Germany	Haarlem	ERR2200029	S	S	S	S	S	S	S	S
2183-13	Hamburg	Germany	lineage4	ERR2199859	R	S	S	S	R	S	S	S
3524-15	Hamburg	Germany	Delhi	ERR2199899 ER	S	S	S	S	S	S	S	S
12448-03	Hamburg	Germany	Beijing	ERR552667	R	R	S	S	R	R	S	S
9773-13	Hamburg	Germany	Haarlem	ERR2200144	S	S	S	S	S	S	S	S
4320-14	Hamburg	Germany	X-type	ERR2199948	S	S	S	S	S	S	S	S
6029-14	Hamburg	Germany	Beijing	ERR2200062	S	S	S	S	S	S	S	S
2302-03	Hamburg	Germany	LAM	ERR553384 ERR	S	S	S	S	S	S	S	S
7968-03	Hamburg	Germany	LAM	ERR550898 ERR	S	S	S	S	S	S	U	S
11782-13	Hamburg	Germany	Haarlem	ERR2199801	S	S	S	S	S	S	S	S

7679-03	Hamburg	Germany	Haarlem	ERR498353 ERR	S	S	S	S	S	S	S	S
6348-14	Hamburg	Germany	LAM	ERR2200082	S	S	S	S	S	S	U	S
7199-99	Hamburg	Germany	Haarlem	ERR2200104	S	S	S	S	S	S	S	S
7114-14	Hamburg	Germany	Haarlem	ERR2200102	S	S	S	S	S	S	S	S
11639-13	Hamburg	Germany	LAM	ERR2199800	S	S	S	S	S	S	S	S
1760-13	Hamburg	Germany	S-type	ERR2199841	R	S	S	S	R	S	S	S
258-15	Hamburg	Germany	Beijing	ERR2199869 ER	S	S	S	S	S	S	U	S
8195-13	Hamburg	Germany	Haarlem	ERR2200111	S	S	S	S	S	S	S	S
5755-13	Hamburg	Germany	Haarlem	ERR2200050	S	S	S	S	S	S	S	S
3881-13	Hamburg	Germany	lineage4	ERR2199910 ER	S	S	S	S	S	S	S	S
3589-14	Hamburg	Germany	lineage4	ERR2199903	S	S	S	S	S	R	S	S
5721-14	Hamburg	Germany	LAM	ERR2200042	S	S	S	S	S	S	S	S
9947-13	Hamburg	Germany	LAM	ERR2200149	S	S	S	S	S	S	S	S
984-13	Hamburg	Germany	Haarlem	ERR2200145	S	S	S	S	S	S	S	S
9751-14	Hamburg	Germany	EAI	ERR2200142 ER	S	S	S	S	S	S	S	S
10820-13	Hamburg	Germany	Delhi	ERR2199782	R	S	S	S	R	S	U	S
1657-03	Hamburg	Germany	Ural	ERR551317 ERR	S	S	S	S	S	S	S	S
8196-13	Hamburg	Germany	Cameroon	ERR2200112	S	S	S	S	S	S	S	S
6207-14	Hamburg	Germany	EAI	ERR2200073	S	S	S	S	S	S	S	S
12148-13	Hamburg	Germany	Haarlem	ERR2199812	S	S	S	S	S	S	S	S
2870-14	Hamburg	Germany	Delhi	ERR2199882 ER	S	S	S	S	S	S	S	S
4222-13	Hamburg	Germany	Beijing	ERR2199918	S	S	S	S	S	S	S	S
12541-13	Hamburg	Germany	Haarlem	ERR2199819	S	S	S	S	S	S	S	S
2611-14	Hamburg	Germany	Haarlem	ERR2199874	S	S	S	S	S	S	S	S
9560-14	Hamburg	Germany	Beijing	ERR2200135 ER	S	S	S	S	S	S	S	S
2548-14	Hamburg	Germany	Haarlem	ERR2199864	S	S	S	S	S	S	S	S
1757-13	Hamburg	Germany	lineage4	ERR2199838	S	S	S	S	S	S	U	S
3802-15	Hamburg	Germany	lineage4	ERR2199906	S	S	S	S	S	S	S	S
4708-14	Hamburg	Germany	Haarlem	ERR2199974	S	S	S	S	S	S	U	S
5010-13	Hamburg	Germany	S-type	ERR2199987	S	S	S	S	S	S	S	S
10121-01	Hamburg	Germany	Beijing	ERR553124 ERR	R	R	R	S	R	R	R	S
1691-01	Hamburg	Germany	Beijing	ERR552755	S	S	S	S	S	S	S	S
3104-15	Hamburg	Germany	Haarlem	ERR2199891	S	S	S	S	S	S	U	S
106-14	Hamburg	Germany	Cameroon	ERR2199776	S	S	S	S	S	S	S	S
3876-04	Hamburg	Germany	Haarlem	ERR552313 ERR	S	S	S	S	S	S	S	S
2611-13	Hamburg	Germany	Haarlem	ERR2199872 ER	S	S	S	S	S	S	U	S
10424-13	Hamburg	Germany	lineage4	ERR2199764 ER	S	S	S	S	S	U	S	S
531-15	Hamburg	Germany	Ural	ERR2200026 ER	S	S	S	S	S	S	S	S
7087-13	Hamburg	Germany	lineage4	ERR2200098 ER	S	S	S	S	S	S	S	S
9532-03	Hamburg	Germany	Haarlem	ERR550639 ERR	S	S	S	S	S	S	S	S
10053-13	Hamburg	Germany	lineage4	ERR2199745	S	S	S	S	S	S	S	S
8885-03	Hamburg	Germany	LAM	ERR551264 ERR	S	S	S	S	S	S	S	S
2569-15	Hamburg	Germany	Haarlem	ERR2199868	S	S	S	S	S	S	S	S
4726-13	Hamburg	Germany	EAI	ERR2199978	S	S	S	S	S	S	U	S
5790-04	Hamburg	Germany	Beijing	ERR550739	S	S	S	S	S	S	S	S
4407-14	Hamburg	Germany	Tur	ERR2199959 ER	S	S	S	S	S	S	S	S
1797-03	Hamburg	Germany	EAI	ERR551470 ERR	S	S	S	S	S	S	S	S
10486-13	Hamburg	Germany	EAI	ERR2199769	S	S	S	S	S	S	U	S
1322-15	Hamburg	Germany	Delhi	ERR2199826 ER	S	S	S	S	S	S	S	S
6044-14	Hamburg	Germany	Cameroon	ERR2200063	S	S	S	S	S	S	S	S
304-14	Hamburg	Germany	Haarlem	ERR2199888	S	S	S	S	S	S	S	S
330-01	Hamburg	Germany	lineage4	ERR550998	S	S	S	S	S	S	S	S
1850-03	Hamburg	Germany	LAM	ERR553278	S	S	S	S	S	S	S	S
4558-13	Hamburg	Germany	Ural	ERR2199965	S	S	S	S	S	S	S	S
4613-14	Hamburg	Germany	EAI	ERR2199967	S	S	S	S	U	S	S	S
1759-13	Hamburg	Germany	lineage4	ERR2199840	S	S	S	S	S	S	S	S
3508-01	Hamburg	Germany	LAM	ERR552783 ERR	S	S	S	S	S	S	S	S
5789-13	Hamburg	Germany	lineage4	ERR2200054	S	S	S	S	S	S	S	S
5374-14	Hamburg	Germany	lineage4	ERR2200028	S	S	S	S	R	S	S	S
5720-14	Hamburg	Germany	Ghana	ERR2200041	S	S	S	S	S	S	S	S
3113-14	Hamburg	Germany	LAM	ERR2199892	S	S	S	S	S	S	U	S
2405-15	Hamburg	Germany	Ural	ERR2199861	S	S	S	S	S	S	S	S
2987-14	Hamburg	Germany	Haarlem	ERR2199885 ER	S	S	S	S	S	S	S	S
11880-13	Hamburg	Germany	lineage4	ERR2199804	R	S	S	S	R	S	S	S
10789-14	Hamburg	Germany	Haarlem	ERR2199780	S	S	S	S	S	S	S	S
10221-14	Hamburg	Germany	Delhi	ERR2199755 ER	S	S	S	S	S	S	S	S
2151-03	Hamburg	Germany	S-type	ERR551852 ERR	S	S	S	S	S	S	S	S
2952-14	Hamburg	Germany	lineage4	ERR2199884	S	S	S	S	S	S	S	S
1314-04	Hamburg	Germany	Beijing	ERR553291	S	S	S	S	S	S	S	S
1032-15	Hamburg	Germany	EAI	ERR2199760 ER	S	S	S	S	S	S	S	S
10529-03	Hamburg	Germany	Tur	ERR552468 ERR	S	S	S	S	S	S	S	S
5510-00	Hamburg	Germany	Beijing	ERR550718	R	R	S	S	R	R	R	S
7953-13	Hamburg	Germany	LAM	ERR2200108	S	S	S	S	S	S	S	S
7684-04	Hamburg	Germany	Haarlem	ERR498366	S	S	S	S	S	S	S	S
11399-14	Hamburg	Germany	Haarlem	ERR2199796	S	S	S	S	S	S	S	S
3286-13	Hamburg	Germany	EAI	ERR2199896	S	S	S	S	S	S	S	S
1000-15	Hamburg	Germany	M. orygis	ERR2199740 ER	S	S	S	S	S	S	U	S
9956-03	Hamburg	Germany	Haarlem	ERR498356	S	S	S	S	S	S	S	S
3401-15	Hamburg	Germany	Haarlem	ERR2199898	S	S	S	S	S	S	S	S
10944-13	Hamburg	Germany	M. bovis	ERR2199785	S	S	S	R	U	S	S	R
8713-14	Hamburg	Germany	lineage4	ERR2200120	S	S	S	S	R	S	S	S
10553-00	Hamburg	Germany	lineage4	ERR550701	S	S	S	S	S	S	S	S
7961-03	Hamburg	Germany	LAM	ERR550624	S	S	S	S	S	S	S	S
1024-01	Hamburg	Germany	Haarlem	ERR498285 ERR	S	S	S	S	S	S	S	S
128-15	Hamburg	Germany	Haarlem	ERR2199820	S	S	S	S	S	S	S	S
11019-13	Hamburg	Germany	Delhi	ERR2199788	S	S	S	S	S	S	S	S
1541-14	Hamburg	Germany	Beijing	ERR2199831	S	S	S	S	S	S	S	S
2638-03	Hamburg	Germany	LAM	ERR551881 ERR	S	S	S	S	S	S	S	S
7091-14	Hamburg	Germany	LAM	ERR2200100	S	S	S	S	S	S	S	S
8101-14	Hamburg	Germany	EAI	ERR2200110	S	S	S	S	S	S	S	S
1556-14	Hamburg	Germany	Haarlem	ERR2199832	S	S	S	S	S	S	S	S
1426-14	Hamburg	Germany	M. orygis	ERR2199829	S	S	S	S	S	S	S	S
4203-01	Hamburg	Germany	Beijing	ERR553277	S	S	S	S	S	S	S	S
11054-14	Hamburg	Germany	Haarlem	ERR2199790 ER	S	S	S	S	S	S	S	S
164-04	Hamburg	Germany	Haarlem	ERR498350 ERR	S	S	S	S	S	S	S	S
11930-14	Hamburg	Germany	Haarlem	ERR2199806	S	S	S	S	S	S	S	S
3103-15	Hamburg	Germany	lineage4	ERR2199890	S	S	S	S	S	S	S	S
5994-14	Hamburg	Germany	Tur	ERR2200059	S	S	S	S	S	S	S	S
1954-13	Hamburg	Germany	lineage4	ERR2199850	S	S	S	S	S	S	S	S
1758-13	Hamburg	Germany	Delhi	ERR2199839	S	S	S	S	S	U	S	S
2049-13	Hamburg	Germany	lineage4	ERR2199851	S	S	S	S	S	S	S	S
3279-14	Hamburg	Germany	Haarlem	ERR2199895	S	S	S	S	S	S	S	S
1500-03	Hamburg	Germany	Beijing	ERR553081 ERR	S	S	S	S	S	S	S	S
1608-04	Hamburg	Germany	Haarlem	ERR498360 ERR	S	S	S	S	S	S	S	S
8308-13	Hamburg	Germany	lineage4	ERR2200113	S	S	S	S	S	S	S	S
2828-13	Hamburg	Germany	S-type	ERR2199879	S	S	S	S	S	S	S	S
675-13	Hamburg	Germany	Haarlem	ERR2200090	S	S	S	S	S	S	S	S
4397-13	Hamburg	Germany	lineage4	ERR2199958	S	S	S	S	S	S	S	S
6533-13	Hamburg	Germany	lineage4	ERR2200085	S	S	S	S	S	S	S	S
8431-03	Hamburg	Germany	Ural	ERR552958 ERR	S	S	S	S	S	S	S	S
2091-14	Hamburg	Germany	lineage4	ERR2199857	S	S	S	S	S	S	U	S
575-03	Hamburg	Germany	Beijing	ERR551086	S	S	S	S	S	S	S	S
4584-14	Hamburg	Germany	lineage4	ERR2199966	S	S	S	S	S	S	U	S
1724-13	Hamburg	Germany	lineage4	ERR2199833	S	S	S	S	R	S	U	S
15292-14	Hamburg	Germany	EAI	ERR2199830	S	S	S	S	S	S	S	S
2208-14	Hamburg	Germany	lineage4	ERR2199860	S	S	S	S	S	S	S	S

7106-13	Hamburg	Germany	Haarlem	ERR2200101	S	S	S	S	S	S	S	S
11252-13	Hamburg	Germany	lineage4	ERR2199794	S	S	S	S	S	S	U	S
10978-14	Hamburg	Germany	Beijing	ERR2199786	R	R	R	R	R	R	R	R
1840-13	Hamburg	Germany	Beijing	ERR2199843 ER	S	S	S	S	S	S	U	S
8100-14	Hamburg	Germany	Beijing	ERR1768638	R	R	R	R	R	R	R	R
6576-12	Hamburg	Germany	Beijing	ERR2200086	S	S	S	S	S	R	U	S
2728-13	Hamburg	Germany	lineage4	ERR2199876 ER	S	S	S	S	S	S	S	S
4898-14	Hamburg	Germany	lineage4	ERR2199986	S	S	S	S	S	S	S	S
4157-13	Hamburg	Germany	S-type	ERR2199916	S	S	S	S	S	S	S	S
2639-14	Hamburg	Germany	Haarlem	ERR2199875	S	S	S	S	U	S	S	S
1511-02	Hamburg	Germany	Beijing	ERR551693 ERR	S	S	S	S	R	S	S	S
10737-02	Hamburg	Germany	Beijing	ERR551927 ERR	S	S	S	S	S	S	S	S
3525-15	Hamburg	Germany	Haarlem	ERR2199901 ER	S	S	S	S	S	S	S	S
3922-13	Hamburg	Germany	Tur	ERR2199913	S	S	S	S	S	S	S	S
178-03	Hamburg	Germany	Beijing	ERR552689 ERR	S	S	S	S	S	S	S	S
1746-14	Hamburg	Germany	Beijing	ERR2199835	S	S	S	S	S	S	S	S
1934-03	Hamburg	Germany	Beijing	ERR551977 ERR	S	S	S	S	S	S	S	S
10487-14	Hamburg	Germany	Delhi	ERR2199770	S	S	S	S	S	S	S	S
9140-14	Hamburg	Germany	Tur	ERR2200130	S	S	S	S	S	S	S	S
8729-13	Hamburg	Germany	Delhi	ERR2200121	S	S	S	S	S	S	S	S
3855-13	Hamburg	Germany	lineage4	ERR2199909	S	S	S	S	S	S	S	S
2057-14	Hamburg	Germany	Haarlem	ERR2199852	S	S	S	S	S	S	S	S
10942-13	Hamburg	Germany	Haarlem	ERR2199784	S	S	S	S	S	S	S	S
11272-14	Hamburg	Germany	Delhi	ERR2199795	R	S	S	S	R	S	S	S
5820-13	Hamburg	Germany	lineage4	ERR2200055	S	S	S	S	S	S	S	S
4373-14	Hamburg	Germany	Delhi	ERR2199957	S	S	S	S	S	S	S	S
12892-13	Hamburg	Germany	LAM	ERR2199823	S	S	S	S	S	S	S	S
5254-14	Hamburg	Germany	lineage4	ERR2200016	S	S	S	S	S	S	S	S
9209-14	Hamburg	Germany	Haarlem	ERR2200131	S	S	S	S	S	S	U	S
11817-14	Hamburg	Germany	lineage4	ERR2199802 ER	S	S	S	S	S	S	S	S
6206-14	Hamburg	Germany	M. bovis	ERR2200072	S	S	S	R	U	S	S	R
11114-03	Hamburg	Germany	Haarlem	ERR498296 ERR	S	S	S	S	S	S	S	S
8859-98	Hamburg	Germany	LAM	ERR553214 ERR	S	S	S	S	S	S	S	S
1020-13	Hamburg	Germany	lineage4	ERR2199754	S	S	S	S	S	S	U	S
4804-13	Hamburg	Germany	Haarlem	ERR2199983	S	S	S	S	S	S	S	S
273-13	Hamburg	Germany	lineage4	ERR2199878	S	S	S	S	S	S	S	S
6372-13	Hamburg	Germany	lineage4	ERR2200083 ER	S	S	S	S	S	S	S	S
51-13	Hamburg	Germany	lineage4	ERR2200003	S	S	S	S	S	S	S	S
9853-13	Hamburg	Germany	lineage4	ERR2200146 ER	S	S	S	S	S	S	S	S
12273-13	Hamburg	Germany	Haarlem	ERR2199816	S	S	S	S	S	S	S	S
4484-13	Hamburg	Germany	Delhi	ERR2199961	S	S	S	S	S	S	S	S
10547-13	Hamburg	Germany	Haarlem	ERR2199772	S	S	S	S	S	S	S	S
8370-04	Hamburg	Germany	Haarlem	ERR498383 ERR	S	S	S	S	S	S	S	S
2111-14	Hamburg	Germany	Haarlem	ERR2199858	S	S	S	S	S	S	S	U
6863-14	Hamburg	Germany	Haarlem	ERR2200091	S	S	S	S	S	S	U	S
12079-13	Hamburg	Germany	LAM	ERR2199809	S	S	S	S	S	S	S	S
2065-13	Hamburg	Germany	Beijing	ERR2199853 ER	R	R	S	S	R	R	R	S
11156-14	Hamburg	Germany	Haarlem	ERR2199792	S	S	S	S	S	S	U	S
10169-14	Hamburg	Germany	lineage4	ERR2199752 ER	S	S	S	S	S	S	S	S
2568-15	Hamburg	Germany	Beijing	ERR2199866 ER	S	S	S	S	S	S	S	S
11615-14	Hamburg	Germany	Beijing	ERR2199799	R	R	R	S	R	R	R	S
1900-14	Hamburg	Germany	EAI	ERR2199848	S	S	S	S	S	S	S	S
9346-14	Hamburg	Germany	LAM	ERR2200133	S	S	S	S	S	S	S	S
2336-02	Hamburg	Germany	Haarlem	ERR551665 ERR	S	S	S	S	S	S	S	S
794-15	Hamburg	Germany	Cameroon	ERR2200107	S	S	S	S	S	S	S	S
11054-13	Hamburg	Germany	lineage4	ERR2199789	S	S	S	S	S	S	S	S
5778-13	Hamburg	Germany	lineage4	ERR2200053	S	S	S	S	S	S	S	S
8073-03	Hamburg	Germany	Haarlem	ERR498293 ERR	S	S	S	S	S	S	S	S
6967-13	Hamburg	Germany	Haarlem	ERR2200097	S	S	S	S	S	S	S	S
8309-13	Hamburg	Germany	M. orygis	ERR2200114	S	S	S	S	S	S	U	S
3093-14	Hamburg	Germany	S-type	ERR2199889	S	S	S	S	S	S	S	S
10047-14	Hamburg	Germany	lineage4	ERR2199744	S	S	S	S	S	S	S	S
1389-13	Hamburg	Germany	Beijing	ERR2199828	S	S	S	S	S	S	S	S
10006-13	Hamburg	Germany	lineage4	ERR2199742	S	S	S	S	S	S	S	S
5914-14	Hamburg	Germany	lineage4	ERR2200057	S	S	S	S	S	S	S	S
1754-15	Hamburg	Germany	Beijing	ERR2199836 ER	R	R	R	R	R	R	R	R
946-03	Hamburg	Germany	LAM	ERR551122 ERR	S	S	S	S	S	S	S	S
12591-02	Hamburg	Germany	lineage4	ERR552283 ERR	S	S	S	S	S	S	S	S
8873-13	Hamburg	Germany	Haarlem	ERR2200125	S	S	S	S	S	S	S	S
12078-13	Hamburg	Germany	LAM	ERR2199808	S	S	S	S	S	S	U	S
9049-13	Hamburg	Germany	EAI	ERR2200126	S	S	S	S	S	S	U	S
4223-13	Hamburg	Germany	lineage4	ERR2199919	S	S	S	S	S	S	S	S
13018-13	Hamburg	Germany	Haarlem	ERR2199824 ER	S	S	S	S	S	S	S	S
12510-13	Hamburg	Germany	Beijing	ERR2199818	R	R	R	R	R	R	R	R
9535-13	Hamburg	Germany	lineage4	ERR2200134	S	S	S	S	S	S	S	S
4950-04	Hamburg	Germany	Beijing	ERR550927	S	S	S	S	S	S	S	S
3399-13	Hamburg	Germany	Delhi	ERR2199897	R	S	S	S	R	S	S	S
3198-13	Hamburg	Germany	lineage4	ERR2199894	S	S	S	S	S	S	S	S
11313-03	Hamburg	Germany	Tur	ERR551400 ERR	S	S	S	S	S	S	S	S
6200-13	Hamburg	Germany	Ghana	ERR2200071	R	S	S	S	R	S	S	S
4727-13	Hamburg	Germany	Haarlem	ERR2199980	S	S	S	S	S	S	S	S
2090-14	Hamburg	Germany	S-type	ERR2199855 ER	S	S	S	S	S	S	S	S
12111-13	Hamburg	Germany	Haarlem	ERR2199810	S	S	S	S	S	U	U	S
3854-13	Hamburg	Germany	lineage4	ERR2199907 ER	S	S	S	S	S	S	S	S
10689-14	Hamburg	Germany	lineage4	ERR2199777	S	S	S	S	S	S	S	S
5907-14	Hamburg	Germany	EAI	ERR2200056	S	S	S	S	S	S	S	S
IT_mdr_IT108	Italy	Italy	Ural		R	R	S	R	R	R	U	R
IT_mdr_485-10	Italy	Italy	Haarlem		R	R	S	S	R	R	R	R
IT_mdr_IT-116	Italy	Italy	Beijing		R	R	R	R	R	R	R	R
IT_mdr_IT235	Italy	Italy	lineage4		R	R	R	S	R	R	R	U
IT_mdr_IT170	Italy	Italy	Beijing		R	R	R	R	R	R	R	R
IT_mdr_865	Italy	Italy	Beijing		R	R	R	n/a	R	R	R	S
IT_mdr_IT231	Italy	Italy	Beijing		R	R	R	R	R	R	U	R
IT_mdr_IT152	Italy	Italy	lineage4		R	R	n/a	R	R	R	R	R
IT_mdr_IT196	Italy	Italy	Beijing		R	R	R	R	R	R	R	U
IT_mdr_IT177	Italy	Italy	lineage4		R	R	R	R	R	F	R	R
IT_mdr_21-LB-IT191	Italy	Italy	Beijing		R	R	R	R	R	R	R	R
IT_mdr_CCM1022	Italy	Italy	LAM		R	R	n/a	n/a	R	R	R	R
IT_mdr_IT247	Italy	Italy	lineage4		R	R	R	R	R	R	R	S
IT_mdr_IT-175	Italy	Italy	Beijing		R	R	R	R	R	R	R	U
IT_mdr_20-LB-IT190	Italy	Italy	lineage4		R	R	S	S	R	R	R	S
IT_mdr_260-15	Italy	Italy	Beijing		R	R	R	S	R	R	R	S
IT_mdr_IT229	Italy	Italy	Haarlem		R	R	R	S	R	R	U	S
IT_mdr_IT43	Italy	Italy	Beijing		R	R	n/a	R	R	R	U	R
IT_mdr_IT-92	Italy	Italy	Cameroon		R	R	S	S	R	R	S	S
IT_mdr_272-15	Italy	Italy	Ural		R	R	S	S	R	R	R	S
IT_mdr_IT171	Italy	Italy	Delhi		R	R	S	S	R	R	S	S
IT_mdr_23-LB-IT198	Italy	Italy	Beijing		R	R	R	R	R	R	R	R
IT_mdr_262-15	Italy	Italy	Beijing		R	R	R	R	R	R	U	R
IT_mdr_IT239	Italy	Italy	lineage4		R	R	R	R	R	R	F	R
IT_mdr_55-10	Italy	Italy	Haarlem		R	R	R	R	R	R	R	U
IT_mdr_IT106	Italy	Italy	Beijing		R	R	R	R	R	R	R	R
IT_mdr_IT167	Italy	Italy	Beijing		R	R	S	S	R	R	S	S
IT_mdr_495-15	Italy	Italy	Beijing		R	R	R	R	R	R	R	R
IT_mdr_17-LB-IT186	Italy	Italy			R	R	R	R	F	F	F	F
IT_mdr_IT131	Italy	Italy	Beijing		R	R	R	R	R	R	R	R

IT_mdr_IT168	Italy	Italy	Delhi	R	R	R	R	R	R	R	R
IT_mdr_IT165	Italy	Italy	West Africar	R	R	S	R	R	F	U	S
IT_mdr_IT179	Italy	Italy	Beijing	R	R	R	R	R	R	R	R
IT_mdr_IT75	Italy	Italy	Haarlem	R	R	R	R	R	R	R	R
IT_mdr_70-10	Italy	Italy	lineage4	R	R	S	S	R	S	S	S
IT_mdr_7ES-ITA204-15	Italy	Italy	Haarlem	R	R	R	R	R	R	R	R
IT_mdr_IT242	Italy	Italy	lineage4	R	R	R	R	R	R	R	R
IT_mdr_LB-IT183	Italy	Italy	Ural	R	R	S	S	R	R	U	S
IT_mdr_IT240	Italy	Italy	Cameroon	R	R	S	S	R	R	S	S
IT_mdr_273-15	Italy	Italy	Beijing	R	R	R	R	R	R	R	R
IT_mdr_8ES-ITA215-15	Italy	Italy	LAM	R	R	R	R	R	R	S	U
IT_mdr_10ES-ITA218-15	Italy	Italy	Beijing	R	R	R	n/a	R	R	R	F
IT_mdr_IT69	Italy	Italy	Beijing	R	R	R	R	R	R	R	R
IT_mdr_IT172	Italy	Italy	Beijing	R	R	R	R	R	R	R	R
IT_mdr_IT201	Italy	Italy	Beijing	R	R	S	S	R	R	R	R
IT_mdr_217-15	Italy	Italy	Beijing	R	R	R	S	R	R	R	S
IT_mdr_258-15	Italy	Italy	Beijing	R	R	n/a	R	R	R	R	U
IT_mdr_IT245	Italy	Italy	Tur	R	R	S	S	R	R	S	S
IT_mdr_18-LB-IT187	Italy	Italy	lineage4	R	R	S	S	R	R	R	S
IT_mdr_IT60	Italy	Italy	lineage4	R	R	S	S	R	R	S	S
IT_mdr_IT-89	Italy	Italy	lineage4	R	R	R	R	R	R	R	R
IT_mdr_IT216	Italy	Italy	lineage4	R	R	n/a	n/a	R	R	R	U
IT_mdr_IT213	Italy	Italy	S-type	R	S	S	S	R	S	S	F
IT_mdr_IT194	Italy	Italy	Ural	R	R	R	R	R	R	U	R
IT_mdr_IT174	Italy	Italy	LAM	R	R	S	S	R	R	S	S
IT_mdr_266-15	Italy	Italy	Tur	R	R	R	S	R	R	R	S
IT_mdr_40-10	Italy	Italy	lineage4	R	R	S	R	F	S	U	S
IT_mdr_IT199	Italy	Italy	Beijing	R	R	R	R	R	R	R	R
IT_mdr_33ES-ITA533-15	Italy	Italy	Ural	R	R	S	S	R	R	U	S
IT_mdr_IT163	Italy	Italy	lineage4	R	R	S	R	R	R	R	R
IT_mdr_IT151	Italy	Italy	Beijing	R	R	n/a	n/a	R	R	S	S
IT_mdr_271-15	Italy	Italy	Beijing	R	R	R	R	R	R	R	R
IT_mdr_IT195	Italy	Italy	Beijing	R	R	S	R	R	R	R	R
IT_mdr_54-15	Italy	Italy	lineage4	R	R	R	R	R	R	R	U
IT_mdr_CCM1021	Italy	Italy	lineage4	R	R	n/a	n/a	R	R	R	U
IT_mdr_IT185	Italy	Italy	Beijing	R	R	R	R	R	R	R	R
IT_mdr_IT197	Italy	Italy	Beijing	R	R	R	R	R	R	R	F
IT_mdr_267-15	Italy	Italy	Haarlem	R	R	S	R	R	R	R	U
IT_mdr_28ES-ITA308-15	Italy	Italy	Beijing	R	R	R	R	R	R	R	F
IT_mdr_IT160	Italy	Italy	Beijing	R	R	R	S	R	R	R	S
IT_mdr_IT238	Italy	Italy	Beijing	R	R	R	R	R	R	R	R
IT_mdr_IT173	Italy	Italy	Ural	R	R	S	S	R	R	U	S
IT_mdr_263-15	Italy	Italy	Beijing	R	R	R	S	R	R	R	U
IT_mdr_IT161	Italy	Italy	lineage4	R	R	R	R	R	R	R	R
IT_mdr_493-15	Italy	Italy	Beijing	R	R	R	R	R	R	R	R
IT_mdr_IT241	Italy	Italy	Ural	R	R	S	S	R	R	R	S
IT_mdr_IT77	Italy	Italy	Beijing	R	R	R	R	R	R	R	R
IT_mdr_11ES-ITA219-15	Italy	Italy	lineage4	R	R	R	R	R	R	R	R
IT_mdr_IT193	Italy	Italy	Ural	R	R	R	R	R	R	R	R
IT_mdr_CCM1013	Italy	Italy	LAM	R	R	R	R	R	R	R	R
IT_mdr_293-15	Italy	Italy	lineage4	R	R	R	R	R	R	R	R
IT_mdr_2ES-ITA43-13	Italy	Italy	Beijing	R	R	R	R	R	R	R	R
IT_mdr_IT-109	Italy	Italy	LAM	R	R	n/a	n/a	R	R	S	S
IT_mdr_IT-176	Italy	Italy	Beijing	n/a	n/a	n/a	R	R	F	R	R
IT_mdr_19-LB-IT189	Italy	Italy	Haarlem	R	R	R	R	R	R	R	R
IT_mdr_IT93	Italy	Italy	lineage4	R	R	S	S	R	R	R	U
IT_mdr_IT192	Italy	Italy	LAM	R	R	S	S	R	R	S	S
IT_mdr_IT248	Italy	Italy	Beijing	R	R	R	S	R	R	U	U
IT_mdr_IT166	Italy	Italy	LAM	R	R	S	R	R	F	S	R
IT_mdr_IT162	Italy	Italy	lineage4	R	R	R	S	R	R	R	R
IT_mdr_270-15	Italy	Italy	Beijing	R	R	R	R	R	R	R	R
IT_mdr_4ES-ITA384-13	Italy	Italy	Beijing	R	R	R	R	R	R	R	R
IT_mdr_IT182	Italy	Italy	Haarlem	R	R	R	R	R	R	R	U
IT_mdr_261-15	Italy	Italy	Beijing	R	R	R	R	R	R	R	F
IT_mdr_IT-128	Italy	Italy	Beijing	R	R	R	R	R	R	R	R
IT_mdr_IT208	Italy	Italy	Haarlem	R	S	R	S	S	S	S	S
IT_mdr_42-IT188	Italy	Italy	Ural	R	R	R	S	R	R	U	S
IT_mdr_207-15	Italy	Italy	LAM	R	R	R	R	R	R	R	R
IT_mdr_257-15	Italy	Italy	Beijing	R	R	R	S	R	R	S	S
IT_mdr_274-15	Italy	Italy	Beijing	R	R	R	S	R	R	U	U
IT_mdr_9ES-ITA216-15	Italy	Italy	Ural	R	R	R	S	R	R	U	S
IT_mdr_IT-90	Italy	Italy	Haarlem	R	R	S	S	R	R	R	U
IT_mdr_IT203	Italy	Italy	Beijing	R	R	S	R	R	R	U	R
IT_mdr_264-15	Italy	Italy	Beijing	R	R	R	R	R	R	R	R
IT_mdr_IT212	Italy	Italy	Beijing	R	R	S	R	R	R	R	R
IT_mdr_53-10	Italy	Italy	Beijing	R	R	R	R	R	R	R	R
IT_mdr_IT-96	Italy	Italy	LAM	R	R	R	R	R	R	R	R
IT_mdr_IT232	Italy	Italy	Beijing	R	R	R	R	R	R	U	R
IT_mdr_IT234	Italy	Italy	lineage4	R	R	R	R	R	R	R	R
IT_mdr_853	Italy	Italy	Beijing	R	R	S	S	R	R	S	S
IT_mdr_IT-104	Italy	Italy	LAM	R	R	n/a	n/a	R	R	R	R
IT_mdr_203-15	Italy	Italy	Ural	R	R	R	S	R	R	U	S
IT_mdr_IT233	Italy	Italy	Beijing	R	R	R	S	R	R	R	R
IT_mdr_IT202	Italy	Italy	Beijing	R	R	R	R	R	R	R	R
IT_mdr_276-15	Italy	Italy	Beijing	R	R	S	R	R	R	R	R
IT_mdr_IT-117	Italy	Italy	Delhi	R	R	n/a	n/a	R	R	R	R
IT_mdr_899	Italy	Italy	Beijing	R	R	S	R	R	R	R	R
IT_mdr_IT-105	Italy	Italy	Beijing	R	R	S	R	R	R	R	S
IT_mdr_IT243	Italy	Italy	Beijing	R	R	R	R	R	R	R	R
IT_mdr_259-15	Italy	Italy	Beijing	R	R	S	R	R	R	S	S
IT_mdr_69-10	Italy	Italy	Haarlem	R	R	S	R	R	R	R	U
IT_mdr_IT181	Italy	Italy	lineage4	R	R	R	R	R	R	R	U
IT_mdr_IT164	Italy	Italy	Beijing	R	R	R	R	R	F	R	R
IT_mdr_IT200	Italy	Italy	Beijing	R	R	R	R	R	R	R	R
IT_mdr_IT97	Italy	Italy	Haarlem	R	R	S	R	R	R	R	U
IT_mdr_IT-178	Italy	Italy	Beijing	R	R	R	R	R	R	R	R
IT_mdr_IT211	Italy	Italy	lineage4	R	R	n/a	S	R	R	S	S
IT_mdr_44-10	Italy	Italy	Haarlem	R	R	R	S	R	R	R	R
IT_mdr_268-15	Italy	Italy	Haarlem	R	R	R	R	R	R	R	U
IT_mdr_IT249	Italy	Italy	Beijing	R	R	R	S	R	R	U	U
IT_mdr_IT169	Italy	Italy	Delhi	R	R	S	S	U	R	S	S
Italy_MGIT68	Italy_MGITstudy	Italy	Beijing	S	S	S	S	S	S	S	S
Italy_MGIT115	Italy_MGITstudy	Italy	LAM	S	S	S	S	S	S	S	S
Italy_MGIT71	Italy_MGITstudy	Italy	lineage4	S	S	S	S	S	S	S	S
Italy_MGITCOS	Italy_MGITstudy	Italy	Haarlem	S	S	S	S	S	S	U	S
Italy_MGIT58	Italy_MGITstudy	Italy	lineage4	S	S	S	S	S	S	S	S
Italy_MGITMAZS	Italy_MGITstudy	Italy	Cameroon	S	S	S	S	S	S	S	S
Italy_MGITPALG	Italy_MGITstudy	Italy	LAM	S	S	S	S	S	S	S	S
Italy_MGIT89	Italy_MGITstudy	Italy	Beijing	R	R	R	R	R	R	R	R
Italy_MGITCIS	Italy_MGITstudy	Italy	Haarlem	S	S	S	S	U	S	S	S
Italy_MGITBRO	Italy_MGITstudy	Italy	Haarlem	S	S	S	S	S	S	S	S
Italy_MGIT70	Italy_MGITstudy	Italy	Delhi	S	S	S	S	S	S	S	S
Italy_MGIT127	Italy_MGITstudy	Italy	LAM	S	S	S	S	S	S	S	S
Italy_MGIT45	Italy_MGITstudy	Italy	lineage4	S	S	S	S	S	U	S	S
Italy_MGITEKU	Italy_MGITstudy	Italy	Delhi	R	S	S	S	R	S	S	S
Italy_MGIT112	Italy_MGITstudy	Italy	lineage4	S	S	S	S	S	S	S	S

Italy_MGITTOUNAW	Italy_MGITstudy	Italy	Tur	S	S	S	S	S	S	S	S
Italy_MGITVOFLO	Italy_MGITstudy	Italy	lineage4	S	S	S	S	S	S	S	S
Italy_MGITPALG2	Italy_MGITstudy	Italy	LAM	S	S	S	S	S	F	S	S
Italy_MGITAMBF	Italy_MGITstudy	Italy	Haarlem	S	S	S	S	S	S	S	S
Italy_MGIT72	Italy_MGITstudy	Italy	Ural	S	S	S	S	S	S	S	S
Italy_MGIT42	Italy_MGITstudy	Italy	Beijing	S	S	S	S	S	S	S	S
Italy_MGIT46	Italy_MGITstudy	Italy	Haarlem	S	S	S	S	S	S	S	S
Italy_MGIT121	Italy_MGITstudy	Italy	lineage4	S	S	S	S	S	S	S	S
Italy_MGITMELAL	Italy_MGITstudy	Italy	S-type	S	S	S	S	S	S	S	S
Italy_MGITPIMO	Italy_MGITstudy	Italy		R	S	S	S	U	S	S	S
Italy_MGITMARK	Italy_MGITstudy	Italy	lineage4	S	S	S	S	U	F	S	S
Italy_MGIT86	Italy_MGITstudy	Italy	LAM	S	S	S	S	S	S	S	S
Italy_MGIT74	Italy_MGITstudy	Italy	Delhi	S	S	S	S	S	S	S	S
Italy_MGIT_BENP	Italy_MGITstudy	Italy	Haarlem	S	S	S	S	F	S	S	S
Italy_MGIT88	Italy_MGITstudy	Italy	Haarlem	S	S	S	S	S	S	S	S
Italy_MGITBEQU	Italy_MGITstudy	Italy	EAI	S	S	S	S	S	S	S	S
Italy_MGIT76	Italy_MGITstudy	Italy	West Africar	S	S	S	S	S	U	U	S
Italy_MGIT54	Italy_MGITstudy	Italy	Haarlem	S	S	S	S	U	S	S	S
Italy_MGIT117	Italy_MGITstudy	Italy	S-type	S	S	S	S	S	S	S	S
Italy_MGIT64	Italy_MGITstudy	Italy	lineage4	S	S	S	S	S	S	S	S
Italy_MGIT44	Italy_MGITstudy	Italy	EAI	S	S	S	S	S	S	S	S
Italy_MGIT98	Italy_MGITstudy	Italy	Beijing	S	S	S	S	S	F	S	S
Italy_MGIT96	Italy_MGITstudy	Italy	Haarlem	S	S	S	S	F	S	S	F
Italy_MGIT108	Italy_MGITstudy	Italy	Haarlem	S	S	S	S	S	S	U	S
Italy_MGIT65	Italy_MGITstudy	Italy	Haarlem	S	S	S	S	S	S	S	S
Italy_MGIT62	Italy_MGITstudy	Italy	Haarlem	S	S	S	S	S	S	S	S
Italy_MGIT77	Italy_MGITstudy	Italy	EAI	S	S	S	S	S	S	S	S
Italy_MGITDJYA	Italy_MGITstudy	Italy		R	S	S	S	R	S	S	S
Italy_MGIT50	Italy_MGITstudy	Italy	Beijing	S	S	S	n/a	S	S	S	S
Italy_MGIT52	Italy_MGITstudy	Italy	lineage4	R	R	R	R	R	R	R	R
Italy_MGIT80	Italy_MGITstudy	Italy	Haarlem	S	S	S	S	S	S	S	S
Italy_MGIT69	Italy_MGITstudy	Italy	Tur	S	S	S	S	S	S	S	S
Italy_MGIT83	Italy_MGITstudy	Italy	lineage4	S	S	S	S	S	F	U	S
Italy_MGIT102	Italy_MGITstudy	Italy	Haarlem	S	S	S	R	S	S	S	R
Italy_MGIT90	Italy_MGITstudy	Italy	Haarlem	S	S	S	S	S	S	U	S
Italy_MGIT53	Italy_MGITstudy	Italy	lineage4	R	R	R	R	R	F	R	R
Italy_MGIT59	Italy_MGITstudy	Italy	Haarlem	S	S	S	S	S	S	S	S
Italy_MGITKEN	Italy_MGITstudy	Italy	Beijing	S	S	S	S	S	S	S	S
Italy_MGITCROCO	Italy_MGITstudy	Italy	lineage4	S	S	S	S	F	S	U	S
Italy_MGIT60	Italy_MGITstudy	Italy	Uganda	S	S	S	S	S	S	S	S
Italy_MGIT_PAGGL	Italy_MGITstudy	Italy	lineage4	S	S	S	S	F	S	S	S
Italy_MGIT79	Italy_MGITstudy	Italy	EAI	S	S	S	S	S	S	S	S
Italy_MGITSOW	Italy_MGITstudy	Italy	Haarlem	S	S	S	S	F	S	S	S
Italy_MGIT51	Italy_MGITstudy	Italy	LAM	R	S	S	S	R	S	S	S
Italy_MGIT73	Italy_MGITstudy	Italy	Haarlem	S	S	S	S	S	S	S	S
Italy_MGIT100	Italy_MGITstudy	Italy	Haarlem	S	S	S	S	S	S	S	S
Italy_MGITDIMOU	Italy_MGITstudy	Italy	EAI	S	S	S	S	F	S	U	S
Italy_MGIT55	Italy_MGITstudy	Italy	Haarlem	S	S	S	S	S	S	S	S
Italy_MGIT99	Italy_MGITstudy	Italy	lineage4	S	S	S	S	S	S	U	S
Italy_MGIT92	Italy_MGITstudy	Italy	Haarlem	S	S	S	S	S	S	S	S
Italy_MGIT101	Italy_MGITstudy	Italy	S-type	S	S	S	S	S	S	S	S
Italy_MGIT43	Italy_MGITstudy	Italy		R	S	S	S	R	S	S	S
Italy_MGITAHS	Italy_MGITstudy	Italy	Uganda	S	S	S	S	S	S	S	S
Italy_MGIT67	Italy_MGITstudy	Italy	West Africar	S	S	S	S	S	U	U	S
Italy_MGIT94	Italy_MGITstudy	Italy	lineage4	S	S	S	S	S	S	S	S
Italy_MGIT_SABD	Italy_MGITstudy	Italy	lineage4	S	S	S	S	S	S	S	S
Italy_MGIT63	Italy_MGITstudy	Italy	EAI	S	S	S	S	S	S	S	S
Italy_MGITDPB	Italy_MGITstudy	Italy	West Africar	S	S	S	S	S	U	U	S
Italy_MGIT_MANA	Italy_MGITstudy	Italy	S-type	S	S	S	S	S	S	S	S
Italy_MGIT113	Italy_MGITstudy	Italy	lineage4	S	S	S	S	S	S	S	S
Italy_MGIT97	Italy_MGITstudy	Italy	LAM	S	S	S	S	S	S	S	S
Italy_MGIT82	Italy_MGITstudy	Italy	West Africar	S	S	S	S	S	U	U	S
Italy_MGIT107	Italy_MGITstudy	Italy	Beijing	S	S	S	S	S	S	S	S
Italy_MGIT114	Italy_MGITstudy	Italy	Haarlem	S	S	S	S	S	S	S	S
Italy_MGITTU	Italy_MGITstudy	Italy	lineage4	S	S	S	S	S	S	S	S
Italy_MGIT66	Italy_MGITstudy	Italy	Beijing	R	S	S	S	R	S	S	S
Italy_MGIT87	Italy_MGITstudy	Italy	LAM	R	S	S	S	R	F	S	S
Italy_MGIT47	Italy_MGITstudy	Italy	Ural	S	S	S	S	S	S	S	S
Italy_MGIT61	Italy_MGITstudy	Italy	lineage4	S	S	S	S	S	S	S	S
Italy_MGIT109	Italy_MGITstudy	Italy	Beijing	S	S	S	S	S	S	U	S
Italy_MGIT85	Italy_MGITstudy	Italy	LAM	S	S	S	S	S	F	R	S
Italy_MGIT116	Italy_MGITstudy	Italy	S-type	S	S	S	S	S	S	S	S
Italy_MGIT84	Italy_MGITstudy	Italy	LAM	R	R	R	S	R	F	R	R
Italy_MGITFRAU	Italy_MGITstudy	Italy	Haarlem	S	S	S	S	S	S	S	S
Italy_MGIT118	Italy_MGITstudy	Italy	lineage4	S	S	S	S	S	S	S	S
Italy_MGIT75	Italy_MGITstudy	Italy	LAM	S	S	S	S	S	S	S	S
Italy_MGITSOW2	Italy_MGITstudy	Italy	Haarlem	S	S	S	S	F	S	S	S
Italy_MGIT105	Italy_MGITstudy	Italy	Haarlem	S	S	S	S	S	S	S	S
Italy_MGIT81	Italy_MGITstudy	Italy	Cameroon	S	S	S	S	S	F	S	S
Italy_MGITABOK	Italy_MGITstudy	Italy	Ghana	S	S	S	S	S	S	S	S
NLA009401354	Harvard	Netherlands	lineage4	R	R	S	n/a	U	R	U	R
NLA009601030	Harvard	Netherlands	Delhi	R	R	S	n/a	R	R	S	S
NLA000301128	Harvard	Netherlands	Haarlem	R	R	R	S	R	R	R	S
NLA009401480	Harvard	Netherlands	Tur	R	R	S	n/a	R	R	S	S
NLA009900478	Harvard	Netherlands	LAM	R	R	S	R	R	R	R	R
NLA009901354	Harvard	Netherlands	Ural	R	R	S	S	R	R	R	R
NLA000201001	Harvard	Netherlands	Tur	R	R	R	S	R	R	R	S
NLA009900895	Harvard	Netherlands	Beijing	R	R	R	S	R	R	R	S
NLA009402429	Harvard	Netherlands	Uganda	R	R	S	n/a	U	R	S	S
NLA000200042	Harvard	Netherlands	Beijing	R	R	R	n/a	R	R	F	R
NLA000700569	Harvard	Netherlands	LAM	R	R	R	R	R	R	R	R
NLA000001768	Harvard	Netherlands	LAM	R	R	S	S	R	R	U	R
NLA000400122	Harvard	Netherlands	LAM	R	R	S	S	R	R	S	S
NLA000401745	Harvard	Netherlands	Beijing	R	R	S	S	R	R	S	U
NLA000301149	Harvard	Netherlands	Haarlem	R	R	S	S	R	R	S	S
NLA009601360	Harvard	Netherlands	LAM	R	R	R	n/a	R	R	R	R
NLA000017744	Harvard	Netherlands	LAM	R	R	R	n/a	R	R	R	S
NLA000801695	Harvard	Netherlands	Beijing	R	R	n/a	n/a	R	R	U	R
NLA009901304	Harvard	Netherlands	lineage4	R	R	R	n/a	U	R	R	U
NLA000300848	Harvard	Netherlands	Haarlem	R	R	R	S	R	F	F	S
NLA009501837	Harvard	Netherlands	Haarlem	R	R	S	n/a	R	R	S	R
NLA009702191	Harvard	Netherlands	Tur	R	R	S	S	R	R	S	S
NLA009802070	Harvard	Netherlands	EAI	R	R	R	R	R	R	R	R
NLA009702403	Harvard	Netherlands	Cameroon	R	R	S	S	R	R	S	R
NLA000017891	Harvard	Netherlands	LAM	R	R	R	S	R	R	R	S
NLA009501599	Harvard	Netherlands	M. bovis	R	R	R	n/a	R	R	R	R
NLA000016604	Harvard	Netherlands	Haarlem	R	R	S	n/a	R	R	S	U
NLA000701259	Harvard	Netherlands	Beijing	R	R	R	R	R	R	R	R
NLA009700174	Harvard	Netherlands	Haarlem	R	R	S	S	R	R	U	S
NLA009400010	Harvard	Netherlands	Delhi	R	R	S	n/a	U	R	S	F
NLA000102124	Harvard	Netherlands	LAM	R	R	R	S	R	R	R	R
NLA009600800	Harvard	Netherlands	EAI	R	R	S	n/a	R	R	S	S
NLA009701938	Harvard	Netherlands	EAI	R	R	S	n/a	R	R	S	S
NLA000400074	Harvard	Netherlands	Ural	R	R	R	R	R	R	R	R
NLA000201392	Harvard	Netherlands	Beijing	R	R	R	S	R	R	R	R
NLA000101954	Harvard	Netherlands	Ghana	R	R	S	S	R	R	S	S

NLA009702465	Harvard	Netherlands	Haarlem	R	R	S	R	S	F	S	S
NLA000100072	Harvard	Netherlands	Cameroon	R	R	S	S	R	R	S	S
NLA009400322	Harvard	Netherlands	Ghana	R	R	S	S	R	R	S	S
NLA009800926	Harvard	Netherlands	Beijing	R	R	S	R	U	R	U	F
NLA000100352	Harvard	Netherlands	Delhi	R	R	R	R	R	R	R	F
NLA000100906	Harvard	Netherlands	LAM	R	R	S	S	R	R	S	R
NLA009902322	Harvard	Netherlands	S-type	R	R	S	S	R	R	R	S
NLA009601659	Harvard	Netherlands	Beijing	R	R	R	S	R	R	U	R
NLA000400459	Harvard	Netherlands	LAM	R	R	S	n/a	R	R	S	S
NLA000401210	Harvard	Netherlands	Beijing	R	R	S	n/a	R	R	R	U
NLA009701714	Harvard	Netherlands	Beijing	R	R	S	S	U	R	S	R
NLA009400630	Harvard	Netherlands	lineage4	R	R	R	n/a	R	R	F	F
NLA000201572	Harvard	Netherlands	EAI	R	R	R	R	R	R	R	R
NLA000500952	Harvard	Netherlands	Uganda	R	R	R	R	R	R	S	R
NLA000301546	Harvard	Netherlands	Ural	R	R	S	S	R	R	R	S
NLA000201244	Harvard	Netherlands	Beijing	R	R	R	S	R	R	R	R
NLA000000560	Harvard	Netherlands	Beijing	R	R	R	S	R	R	R	U
NLA000401230	Harvard	Netherlands	Beijing	R	R	R	S	R	R	R	S
NLA000100450	Harvard	Netherlands	Ghana	R	R	S	S	R	R	S	S
NLA000301077	Harvard	Netherlands	M. bovis	R	R	R	R	R	R	R	R
NLA000601176	Harvard	Netherlands	Delhi	R	R	R	S	R	R	R	R
NLA000100143	Harvard	Netherlands	LAM	R	R	S	n/a	R	R	R	S
NLA009902230	Harvard	Netherlands	LAM	R	R	S	S	R	R	S	S
NLA009501710	Harvard	Netherlands	LAM	R	R	S	n/a	R	R	S	S
NLA009501246	Harvard	Netherlands	lineage4	R	R	R	n/a	R	R	R	R
NLA009600875	Harvard	Netherlands	LAM	R	R	S	S	R	R	S	S
NLA000101151	Harvard	Netherlands	lineage4	R	R	S	S	R	F	R	S
NLA009800720	Harvard	Netherlands	EAI	R	R	R	S	R	R	R	S
NLA000801697	Harvard	Netherlands	Haarlem	R	R	n/a	n/a	S	S	S	S
NLA009600108	Harvard	Netherlands	Beijing	R	R	S	S	R	R	S	S
NLA009700296	Harvard	Netherlands	Delhi	R	R	S	S	R	R	S	S
NLA009501310	Harvard	Netherlands	LAM	R	R	S	n/a	R	R	R	R
NLA009500730	Harvard	Netherlands	lineage4	R	R	R	n/a	R	R	U	S
NLA000101717	Harvard	Netherlands	LAM	R	R	S	S	R	R	S	S
NLA000016985	Harvard	Netherlands	Tur	R	R	R	n/a	R	R	R	S
NLA000301652	Harvard	Netherlands	Beijing	R	R	S	S	R	R	R	R
NLA009500011	Harvard	Netherlands	lineage4	R	R	R	n/a	R	R	R	R
NLA000101876	Harvard	Netherlands	Cameroon	R	R	S	n/a	R	R	S	S
NLA000202008	Harvard	Netherlands	lineage4	R	R	R	n/a	R	R	R	R
NLA000201734	Harvard	Netherlands	lineage4	R	R	R	R	R	R	R	R
NLA000200442	Harvard	Netherlands	Beijing	R	R	R	S	R	R	R	S
NLA000601492	Harvard	Netherlands	LAM	R	R	S	S	R	R	S	S
NLA009801095	Harvard	Netherlands	Delhi	R	R	R	n/a	R	R	U	S
NLA009900182	Harvard	Netherlands	Haarlem	R	R	R	R	R	R	R	R
NLA000000709	Harvard	Netherlands	Delhi	R	R	S	S	R	R	S	S
NLA000401316	Harvard	Netherlands	Delhi	R	R	R	R	R	R	R	R
NLA000201358	Harvard	Netherlands	Delhi	R	R	S	S	R	R	R	F
NLA009701340	Harvard	Netherlands	lineage4	R	R	S	S	R	R	S	S
NLA000601773	Harvard	Netherlands	Delhi	R	R	S	S	R	R	R	S
NLA000401303	Harvard	Netherlands	Ghana	R	R	S	n/a	R	R	S	S
NLA009800667	Harvard	Netherlands	Haarlem	R	R	R	R	R	R	R	R
NLA000001061	Harvard	Netherlands	Delhi	R	R	S	S	R	R	S	S
NLA009401519	Harvard	Netherlands	Beijing	R	R	S	n/a	R	R	S	S
NLA000017603	Harvard	Netherlands	Delhi	R	R	S	n/a	R	R	S	F
NLA000700190	Harvard	Netherlands	X-type	R	R	R	n/a	R	R	R	U
NLA000001969	Harvard	Netherlands	LAM	R	R	S	S	R	R	S	S
NLA000016764	Harvard	Netherlands	LAM	R	R	R	n/a	U	F	S	S
NLA000401820	Harvard	Netherlands	Delhi	R	R	R	S	R	R	R	R
NLA000301359	Harvard	Netherlands	S-type	R	R	S	S	R	F	S	S
NLA009400815	Harvard	Netherlands	S-type	R	R	R	n/a	R	R	R	U
NLA000301067	Harvard	Netherlands	Delhi	R	R	S	S	R	R	R	R
NLA009702211	Harvard	Netherlands	LAM	R	R	S	R	R	R	R	R
NLA009502009	Harvard	Netherlands	LAM	R	R	S	n/a	R	R	R	U
NLA009801661	Harvard	Netherlands	S-type	R	R	S	R	R	R	S	R
NLA000300607	Harvard	Netherlands	Beijing	R	R	S	S	R	R	S	S
NLA000401297	Harvard	Netherlands	Delhi	R	R	S	S	R	R	S	U
NLA000200433	Harvard	Netherlands	West Africar	R	R	S	n/a	R	R	U	S
NLA000801696	Harvard	Netherlands	Beijing	R	R	n/a	n/a	R	R	R	R
NLA009901267	Harvard	Netherlands	lineage4	R	R	R	S	U	R	R	U
NLA000701201	Harvard	Netherlands	Delhi	R	R	S	S	R	R	S	S
NLA009900939	Harvard	Netherlands	Ural	R	R	S	R	R	R	R	F
NLA000301074	Harvard	Netherlands	Ghana	R	R	R	S	R	R	R	R
NLA000801810	Harvard	Netherlands	Haarlem	R	R	n/a	n/a	R	R	S	S
NLA009801164	Harvard	Netherlands	Ural	R	R	R	R	R	R	R	F
NLA000400812	Harvard	Netherlands	Delhi	R	R	S	S	R	R	R	S
NLA000000636	Harvard	Netherlands	LAM	R	R	S	S	R	R	U	S
NLA009601810	Harvard	Netherlands	LAM	R	R	S	R	R	R	R	R
NLA009801154	Harvard	Netherlands	lineage4	R	R	S	S	R	R	S	F
NLA000018190	Harvard	Netherlands	Haarlem	R	R	S	S	U	R	S	S
NLA000500538	Harvard	Netherlands	Beijing	R	R	R	S	R	R	R	S
NLA009502172	Harvard	Netherlands	LAM	R	R	S	n/a	R	R	R	R
NLA000201682	Harvard	Netherlands	Haarlem	R	R	S	S	R	R	S	S
NLA000017848	Harvard	Netherlands	LAM	R	R	R	n/a	R	R	R	S
NLA009500746	Harvard	Netherlands	lineage4	R	R	R	S	R	R	U	S
NLA009801500	Harvard	Netherlands	Beijing	R	R	S	S	R	R	S	S
NLA009700256	Harvard	Netherlands	Ural	R	R	R	S	R	R	R	S
NLA009701444	Harvard	Netherlands	lineage4	R	R	S	S	R	R	S	S
NLA000301029	Harvard	Netherlands	Haarlem	R	R	S	S	R	R	R	S
NLA000500665	Harvard	Netherlands	lineage4	R	R	R	S	U	R	R	R
NLA000301715	Harvard	Netherlands	Beijing	R	R	S	S	R	R	R	S
NLA009700983	Harvard	Netherlands	Uganda	R	R	R	n/a	R	R	R	F
NLA000300865	Harvard	Netherlands	Ghana	R	R	R	S	R	R	R	S
NLA000300339	Harvard	Netherlands	Tur	R	R	R	S	R	R	R	R
NLA000401154	Harvard	Netherlands	Beijing	R	R	S	S	R	R	R	R
NLA000501371	Harvard	Netherlands	Beijing	R	R	R	n/a	R	R	R	U
NLA000200052	Harvard	Netherlands	Uganda	R	R	R	n/a	R	R	R	R
NLA009902034	Harvard	Netherlands	lineage4	R	R	R	S	U	R	R	U
NLA001000120	Harvard	Netherlands	Haarlem	S	S	n/a	n/a	U	R	S	R
NLA000016726	Harvard	Netherlands	LAM	R	R	S	n/a	R	R	S	S
NLA009800243	Harvard	Netherlands	Tur	R	R	R	S	R	R	R	S
NLA009900180	Harvard	Netherlands	Beijing	R	R	R	R	R	R	R	F
NLA000100510	Harvard	Netherlands	S-type	R	R	S	S	R	R	S	S
NLA000801694	Harvard	Netherlands	Delhi	R	R	n/a	n/a	S	S	S	S
NL532	Netherlands	Netherlands	lineage4	S	S	S	S	S	S	S	S
NL015	Netherlands	Netherlands	Haarlem	S	S	S	S	S	S	S	S
NL039	Netherlands	Netherlands	lineage4	S	S	S	S	S	S	S	S
NL153	Netherlands	Netherlands	Haarlem	S	R	S	S	S	R	S	S
NL041	Netherlands	Netherlands	Ural	S	S	S	S	S	S	S	S
NL147	Netherlands	Netherlands	Cameroon	n/a	S	S	S	R	S	S	S
NL060	Netherlands	Netherlands	Tur	S	S	S	n/a	S	S	S	S
NL515	Netherlands	Netherlands	Tur	S	S	S	S	U	S	S	S
NL243	Netherlands	Netherlands	LAM	S	S	S	S	S	S	S	S
NL034	Netherlands	Netherlands	Uganda	S	S	S	S	F	S	S	S
NL328	Netherlands	Netherlands	M. bovis	S	S	S	R	U	S	S	R
NL135	Netherlands	Netherlands	Haarlem	n/a	S	S	S	R	S	S	S
NL198	Netherlands	Netherlands	EAI	S	S	S	S	S	S	S	S

NL047	Netherlands	Netherlands	lineage4	S	S	S	S	S	S	S	S
NL355	Netherlands	Netherlands	lineage4	S	S	S	S	S	S	S	S
NL554	Netherlands	Netherlands	Delhi	S	S	S	S	S	S	S	U
NL317	Netherlands	Netherlands	Delhi	S	S	S	S	S	S	U	S
NL001	Netherlands	Netherlands	lineage4	S	S	S	S	S	S	S	S
NL430	Netherlands	Netherlands	Haarlem	S	S	S	S	S	S	S	S
NL125	Netherlands	Netherlands	Delhi	R	S	S	S	R	S	S	S
NL588	Netherlands	Netherlands	lineage4	R	n/a	S	S	R	R	S	S
NL421	Netherlands	Netherlands	Haarlem	S	S	S	S	S	S	S	S
NL530	Netherlands	Netherlands	Beijing	S	S	S	S	S	S	S	S
NL331	Netherlands	Netherlands	Delhi	S	S	S	S	S	S	S	S
NL220	Netherlands	Netherlands	lineage4	S	S	S	n/a	S	S	U	F
NL491	Netherlands	Netherlands	lineage4	n/a	R	R	S	R	R	S	S
NL164	Netherlands	Netherlands	Delhi	S	S	S	S	S	S	S	S
NL079	Netherlands	Netherlands	lineage4	S	S	S	S	S	S	U	S
NL553	Netherlands	Netherlands	lineage4	S	S	S	S	S	S	S	S
NL529	Netherlands	Netherlands	Haarlem	S	S	S	S	S	S	S	S
NL241	Netherlands	Netherlands	Beijing	R	R	S	R	R	R	R	R
NL201	Netherlands	Netherlands	lineage4	S	S	S	S	F	F	F	F
NL287	Netherlands	Netherlands	EAI	S	S	S	S	S	S	S	S
NL145	Netherlands	Netherlands	lineage4	S	S	S	S	S	S	S	S
NL270	Netherlands	Netherlands	LAM	S	S	S	S	S	S	S	S
NL542	Netherlands	Netherlands	lineage4	S	S	S	S	S	U	S	S
NL508	Netherlands	Netherlands	lineage4	S	S	S	S	S	S	S	S
NL555	Netherlands	Netherlands	LAM	S	S	S	S	S	S	S	S
NL066	Netherlands	Netherlands	EAI	S	S	S	S	S	S	S	S
NL136	Netherlands	Netherlands	Beijing	S	S	S	S	S	S	S	S
NL004	Netherlands	Netherlands	Haarlem	S	S	S	n/a	S	F	U	S
NL391	Netherlands	Netherlands	S-type	S	S	S	S	S	S	S	S
NL032	Netherlands	Netherlands	LAM	S	S	S	S	U	S	S	S
NL087	Netherlands	Netherlands	Delhi	S	S	S	S	S	S	S	S
NL402	Netherlands	Netherlands	lineage4	S	S	S	S	S	S	S	S
NL045	Netherlands	Netherlands	lineage4	S	S	S	S	S	S	S	S
NL175	Netherlands	Netherlands	Delhi	S	S	S	S	S	S	S	S
NL265	Netherlands	Netherlands	Tur	S	S	S	S	S	S	S	S
NL370	Netherlands	Netherlands	EAI	R	S	S	S	R	R	S	S
NL077	Netherlands	Netherlands	Beijing	R	R	n/a	S	R	R	U	S
NL035	Netherlands	Netherlands	lineage4	S	S	S	S	S	S	U	S
NL245	Netherlands	Netherlands	Tur	S	S	S	S	S	S	U	S
NL580	Netherlands	Netherlands	EAI	n/a	S	S	S	R	S	S	S
NL385	Netherlands	Netherlands	Delhi	S	S	S	S	S	S	S	S
NL100	Netherlands	Netherlands	Delhi	S	S	S	S	S	S	S	S
NL545	Netherlands	Netherlands	lineage4	S	S	S	S	S	S	S	S
NL543	Netherlands	Netherlands	Beijing	S	S	S	S	S	S	U	S
NL253	Netherlands	Netherlands	Haarlem	S	S	S	S	F	S	U	S
NL062	Netherlands	Netherlands	BCG	S	S	S	R	S	S	S	R
NL434	Netherlands	Netherlands	Delhi	S	S	S	S	S	S	S	S
NL102	Netherlands	Netherlands	Beijing	S	S	S	S	S	S	S	S
NL061	Netherlands	Netherlands	Haarlem	S	S	S	S	S	S	S	S
NL454	Netherlands	Netherlands	lineage4	S	S	S	n/a	S	S	U	S
NL428	Netherlands	Netherlands	Haarlem	S	S	S	S	S	S	S	S
NL130	Netherlands	Netherlands	EAI	S	S	S	R	S	S	S	S
NL172	Netherlands	Netherlands	EAI	S	S	S	R	S	S	S	S
NL185	Netherlands	Netherlands	EAI	S	S	S	S	S	S	S	S
NL224	Netherlands	Netherlands	Tur	S	S	S	S	S	S	S	S
NL056	Netherlands	Netherlands	Beijing	S	S	S	S	S	S	S	S
NL492	Netherlands	Netherlands	Beijing	R	R	n/a	R	R	R	U	R
NL496	Netherlands	Netherlands	Haarlem	S	S	S	S	S	S	S	S
NL363	Netherlands	Netherlands	Delhi	R	S	S	S	R	S	U	S
NL213	Netherlands	Netherlands	Haarlem	S	S	S	n/a	S	S	S	S
NL068	Netherlands	Netherlands	Delhi	S	S	S	S	S	S	S	S
NL065	Netherlands	Netherlands	Delhi	S	S	S	S	S	S	U	S
NL023	Netherlands	Netherlands	Delhi	S	S	S	S	S	S	S	S
NL250	Netherlands	Netherlands	LAM	S	S	S	S	S	S	S	S
NL111	Netherlands	Netherlands	EAI	S	S	S	S	S	S	S	S
NL285	Netherlands	Netherlands	LAM	S	S	S	S	S	S	S	S
NL503	Netherlands	Netherlands	LAM	S	S	S	S	S	S	S	S
NL396	Netherlands	Netherlands	lineage4	S	S	S	S	U	S	S	S
NL299	Netherlands	Netherlands	LAM	S	S	S	S	S	S	S	S
NL601	Netherlands	Netherlands	S-type	S	S	S	S	S	S	S	S
NL433	Netherlands	Netherlands	lineage4	R	S	S	S	R	R	S	S
NL151	Netherlands	Netherlands	Beijing	S	S	S	S	S	S	U	S
NL084	Netherlands	Netherlands	Haarlem	S	S	S	S	S	S	S	S
NL105	Netherlands	Netherlands	Delhi	R	S	S	S	R	S	S	S
NL419	Netherlands	Netherlands	S-type	S	S	S	S	S	S	S	S
NL365	Netherlands	Netherlands	S-type	S	S	S	n/a	S	S	U	S
NL332	Netherlands	Netherlands	Delhi	S	S	S	S	S	S	S	S
NL106	Netherlands	Netherlands	Delhi	S	S	S	S	S	S	S	S
NL244	Netherlands	Netherlands	Haarlem	R	S	S	S	R	S	S	S
NL086	Netherlands	Netherlands	Haarlem	S	S	S	S	S	S	S	S
NL076	Netherlands	Netherlands	Haarlem	S	S	S	S	S	S	S	S
NL559	Netherlands	Netherlands	EAI	S	S	S	S	S	S	S	S
NL548	Netherlands	Netherlands	West Africar	S	S	S	S	S	U	U	S
NL573	Netherlands	Netherlands	Tur	S	S	S	S	S	S	S	S
NL330	Netherlands	Netherlands	EAI	R	S	S	S	R	S	S	S
NL570	Netherlands	Netherlands	Haarlem	S	S	S	S	S	S	S	S
NL090	Netherlands	Netherlands	Haarlem	S	S	S	S	S	S	S	S
NL044	Netherlands	Netherlands	lineage4	S	S	S	S	S	S	S	S
NL346	Netherlands	Netherlands	lineage4	S	S	S	S	S	S	S	S
NL264	Netherlands	Netherlands	lineage4	S	S	S	S	S	S	S	S
NL455	Netherlands	Netherlands	Haarlem	S	S	S	S	S	S	S	S
NL410	Netherlands	Netherlands	Haarlem	S	S	S	S	S	S	S	S
NL158	Netherlands	Netherlands	Haarlem	S	S	S	n/a	S	S	S	S
NL322	Netherlands	Netherlands	Delhi	S	S	S	S	S	S	S	S
NL193	Netherlands	Netherlands	EAI	S	S	S	S	U	S	S	S
NL324	Netherlands	Netherlands	EAI	S	S	S	S	S	S	S	S
NL431	Netherlands	Netherlands	BCG	S	S	S	R	S	S	S	R
NL148	Netherlands	Netherlands	Delhi	S	S	S	S	S	S	S	S
NL273	Netherlands	Netherlands	Haarlem	S	S	S	S	S	S	S	S
NL073	Netherlands	Netherlands	Delhi	S	S	S	S	S	S	U	S
NL449	Netherlands	Netherlands	Delhi	S	S	S	S	S	S	S	S
NL202	Netherlands	Netherlands	Beijing	R	S	S	S	R	S	S	S
NL437	Netherlands	Netherlands	Delhi	S	S	S	n/a	S	S	S	S
NL613	Netherlands	Netherlands	EAI	S	S	S	S	S	S	S	S
NL263	Netherlands	Netherlands	Delhi	S	S	S	S	S	S	U	S
NL565	Netherlands	Netherlands	Haarlem	S	S	S	S	S	S	S	S
NL096	Netherlands	Netherlands	EAI	S	S	S	S	S	S	S	S
NL550	Netherlands	Netherlands	Haarlem	S	S	S	n/a	S	S	S	S
NL247	Netherlands	Netherlands	Delhi	S	S	S	S	S	S	S	S
NL392	Netherlands	Netherlands	Uganda	S	S	S	S	S	S	U	S
NL566	Netherlands	Netherlands	Tur	S	S	S	S	S	S	S	S
NL351	Netherlands	Netherlands	M. bovis	S	S	S	R	S	S	U	R
NL203	Netherlands	Netherlands	EAI	S	S	S	S	S	S	S	S
NL196	Netherlands	Netherlands	EAI	S	S	S	S	S	S	S	S
NL451	Netherlands	Netherlands	Haarlem	S	S	S	S	S	S	S	S
NL149	Netherlands	Netherlands	Beijing	S	S	S	S	S	S	S	S

NL304	Netherlands	Netherlands	lineage4	S	S	S	S	S	S	U	S
NL534	Netherlands	Netherlands	lineage4	S	S	S	S	S	S	S	S
NL485	Netherlands	Netherlands	EAI	S	S	S	S	S	S	S	S
NL309	Netherlands	Netherlands	Ghana	S	S	S	n/a	S	S	S	S
NL315	Netherlands	Netherlands	lineage4	S	S	S	S	S	S	S	S
NL108	Netherlands	Netherlands	Delhi	S	S	S	S	S	S	S	S
NL156	Netherlands	Netherlands	Delhi	S	S	S	S	S	U	S	S
NL414	Netherlands	Netherlands	LAM	S	S	S	S	S	S	S	S
NL382	Netherlands	Netherlands	lineage4	S	S	S	S	S	S	S	S
NL275	Netherlands	Netherlands	LAM	R	S	S	S	R	S	R	S
NL563	Netherlands	Netherlands	lineage4	S	S	S	n/a	S	S	S	S
NL549	Netherlands	Netherlands	LAM	S	S	S	S	S	S	S	S
NL003	Netherlands	Netherlands	Haarlem	S	S	S	S	S	S	S	S
NL082	Netherlands	Netherlands	EAI	S	S	S	S	S	S	S	S
NL439	Netherlands	Netherlands	LAM	S	S	S	S	S	S	U	S
NL072	Netherlands	Netherlands	Delhi	R	R	n/a	S	R	R	R	S
NL178	Netherlands	Netherlands	EAI	R	S	S	S	R	S	S	S
NL354	Netherlands	Netherlands	Tur	S	S	S	n/a	S	S	S	S
NL471	Netherlands	Netherlands	lineage4	S	S	S	S	S	S	S	S
NL445	Netherlands	Netherlands	Cameroon	S	S	S	S	S	S	S	S
NL409	Netherlands	Netherlands	EAI	S	S	S	S	S	S	S	S
NL133	Netherlands	Netherlands	Haarlem	S	S	S	S	S	S	S	S
NL126	Netherlands	Netherlands	lineage4	S	S	S	S	S	S	S	S
NL018	Netherlands	Netherlands	lineage4	S	S	S	S	S	S	S	S
NL583	Netherlands	Netherlands	lineage4	S	S	S	n/a	S	U	S	S
NL207	Netherlands	Netherlands	EAI	S	S	S	S	S	S	S	S
NL480	Netherlands	Netherlands	EAI	S	S	S	S	S	S	S	S
NL167	Netherlands	Netherlands	LAM	S	S	S	S	S	S	S	S
NL380	Netherlands	Netherlands	Haarlem	S	S	S	S	S	S	S	S
NL232	Netherlands	Netherlands	LAM	S	S	S	S	S	S	S	S
NL204	Netherlands	Netherlands	Haarlem	S	S	S	S	S	S	S	S
NL361	Netherlands	Netherlands	Haarlem	S	S	S	S	S	S	U	S
NL456	Netherlands	Netherlands	Haarlem	S	S	S	S	S	S	S	S
NL452	Netherlands	Netherlands	Delhi	S	S	S	S	U	S	S	S
NL401	Netherlands	Netherlands	EAI	S	S	S	S	S	S	S	S
NL152	Netherlands	Netherlands	Cameroon	R	R	S	S	R	R	S	S
NL435	Netherlands	Netherlands	LAM	S	S	S	S	S	S	S	S
NL569	Netherlands	Netherlands	LAM	S	S	S	S	S	S	S	S
NL012	Netherlands	Netherlands	Beijing	R	S	S	S	R	S	S	S
NL479	Netherlands	Netherlands	S-type	S	S	S	S	S	S	S	S
NL343	Netherlands	Netherlands	Delhi	S	S	S	S	S	S	S	S
NL176	Netherlands	Netherlands	LAM	S	S	S	S	S	S	S	S
NL104	Netherlands	Netherlands	S-type	S	S	S	n/a	S	S	U	S
NL599	Netherlands	Netherlands	EAI	S	S	S	S	S	S	S	S
NL266	Netherlands	Netherlands	Delhi	S	S	S	S	S	S	S	S
NL500	Netherlands	Netherlands	EAI	S	S	S	S	S	S	S	S
NL295	Netherlands	Netherlands	Delhi	S	S	S	S	S	S	S	S
NL069	Netherlands	Netherlands	EAI	S	S	S	S	S	S	U	S
NL609	Netherlands	Netherlands	LAM	R	S	S	S	R	S	S	S
NL134	Netherlands	Netherlands	S-type	S	S	S	n/a	S	S	U	S
NL043	Netherlands	Netherlands	EAI	S	S	S	S	S	S	S	S
NL574	Netherlands	Netherlands	EAI	S	S	S	S	S	S	S	S
NL611	Netherlands	Netherlands	LAM	S	S	S	S	S	S	S	S
NL181	Netherlands	Netherlands	LAM	S	S	S	n/a	S	S	S	S
NL400	Netherlands	Netherlands	lineage4	S	S	S	S	S	S	S	S
NL058	Netherlands	Netherlands	S-type	S	S	S	S	S	S	S	S
NL572	Netherlands	Netherlands	EAI	S	S	S	R	S	S	U	S
NL422	Netherlands	Netherlands	Delhi	S	S	S	S	S	S	S	S
NL493	Netherlands	Netherlands	LAM	S	S	S	S	S	S	S	S
NL586	Netherlands	Netherlands	LAM	S	S	S	S	S	S	U	S
NL249	Netherlands	Netherlands	LAM	S	S	S	S	S	S	S	S
NL283	Netherlands	Netherlands	Delhi	R	R	n/a	R	R	R	R	R
NL081	Netherlands	Netherlands	EAI	S	S	S	S	S	S	S	S
NL404	Netherlands	Netherlands	Haarlem	S	S	S	S	S	S	S	S
NL225	Netherlands	Netherlands	S-type	n/a	S	S	S	R	S	S	S
NL313	Netherlands	Netherlands	EAI	S	S	S	S	R	S	S	S
NL592	Netherlands	Netherlands	Delhi	S	S	S	S	S	S	S	S
NL042	Netherlands	Netherlands	Beijing	S	S	S	S	S	S	S	S
NL415	Netherlands	Netherlands	Delhi	S	S	S	S	S	S	S	S
NL490	Netherlands	Netherlands	EAI	S	S	S	S	S	S	S	S
NL132	Netherlands	Netherlands	S-type	S	S	S	S	S	S	S	S
NL088	Netherlands	Netherlands	S-type	S	S	S	n/a	S	S	U	S
NL005	Netherlands	Netherlands	lineage4	S	S	S	S	S	S	S	S
NL379	Netherlands	Netherlands	Haarlem	S	S	S	S	S	S	S	S
NL155	Netherlands	Netherlands	Haarlem	n/a	S	S	S	R	S	S	S
NL107	Netherlands	Netherlands	Tur	S	S	S	S	S	S	S	S
NL154	Netherlands	Netherlands	Tur	S	S	S	S	F	S	S	S
NL022	Netherlands	Netherlands	Tur	S	S	S	n/a	S	S	S	S
NL412	Netherlands	Netherlands	lineage4	S	S	S	S	S	S	U	S
NL371	Netherlands	Netherlands	Delhi	S	S	S	S	S	S	S	S
NL478	Netherlands	Netherlands	Cameroon	S	S	S	S	S	S	S	S
NL297	Netherlands	Netherlands	Tur	S	S	S	S	S	S	S	S
NL221	Netherlands	Netherlands	LAM	S	S	S	S	S	S	S	S
NL466	Netherlands	Netherlands	Beijing	S	S	S	S	S	S	S	S
NL442	Netherlands	Netherlands	Haarlem	S	S	S	S	S	S	S	S
NL571	Netherlands	Netherlands	M. bovis	S	S	S	R	S	S	S	R
NL171	Netherlands	Netherlands	Beijing	n/a	S	S	S	R	S	S	S
NL537	Netherlands	Netherlands	Tur	S	S	S	S	S	S	S	S
NL296	Netherlands	Netherlands	EAI	S	S	S	S	S	S	U	S
NL587	Netherlands	Netherlands	Haarlem	S	S	S	S	S	S	U	S
NL095	Netherlands	Netherlands	Delhi	S	S	S	S	S	S	U	S
NL458	Netherlands	Netherlands	M. bovis	S	S	S	R	S	S	S	R
NL405	Netherlands	Netherlands	Cameroon	S	S	S	S	S	S	U	S
NL120	Netherlands	Netherlands	EAI	S	S	S	R	S	S	U	S
NL498	Netherlands	Netherlands	lineage4	S	S	S	S	S	S	S	S
NL501	Netherlands	Netherlands	LAM	S	S	S	S	S	S	S	S
NL163	Netherlands	Netherlands	Haarlem	S	S	S	n/a	S	S	S	S
NL157	Netherlands	Netherlands	Haarlem	S	S	S	S	S	S	S	S
NL420	Netherlands	Netherlands	Delhi	R	S	S	S	R	S	U	S
NL438	Netherlands	Netherlands	LAM	S	S	S	S	S	U	S	S
NL166	Netherlands	Netherlands	EAI	S	S	S	S	S	S	S	S
NL137	Netherlands	Netherlands	Haarlem	S	S	S	S	S	U	S	S
NL170	Netherlands	Netherlands	Tur	R	S	S	S	R	S	U	S
NL215	Netherlands	Netherlands	lineage4	S	S	S	S	S	S	U	S
NL378	Netherlands	Netherlands	Delhi	S	S	S	S	S	S	S	S
NL462	Netherlands	Netherlands	Haarlem	S	S	S	S	S	S	S	S
NL535	Netherlands	Netherlands	LAM	S	S	S	S	S	S	S	S
NL372	Netherlands	Netherlands	Delhi	S	S	S	S	S	S	S	S
NL230	Netherlands	Netherlands	Delhi	S	S	S	S	S	S	S	S
NL598	Netherlands	Netherlands	Delhi	S	S	S	S	S	S	S	S
NL486	Netherlands	Netherlands	Haarlem	S	S	S	S	S	S	S	S
NL308	Netherlands	Netherlands	lineage4	S	S	S	n/a	S	S	S	S
NL284	Netherlands	Netherlands	Beijing	S	S	S	S	S	S	S	S
NL540	Netherlands	Netherlands	Delhi	S	S	S	S	S	S	S	S
NL387	Netherlands	Netherlands	Delhi	S	S	S	S	F	U	U	S
NL209	Netherlands	Netherlands	Haarlem	S	S	S	n/a	S	S	S	S

NL246	Netherlands	Netherlands	Haarlem	S	S	S	S	S	S	S	S
NL381	Netherlands	Netherlands	lineage4	S	S	S	S	S	S	S	S
NL292	Netherlands	Netherlands	lineage4	S	S	S	S	S	S	S	S
NL180	Netherlands	Netherlands	Beijing	S	S	S	S	S	S	S	S
NL538	Netherlands	Netherlands	Delhi	S	S	S	S	S	S	S	S
NL048	Netherlands	Netherlands	lineage4	S	S	S	S	S	S	S	S
NL289	Netherlands	Netherlands	LAM	S	S	S	S	S	S	S	S
NL321	Netherlands	Netherlands	LAM	S	S	S	S	S	S	S	S
NL511	Netherlands	Netherlands	EAI	S	S	S	S	S	S	S	S
NL168	Netherlands	Netherlands	Haarlem	S	S	S	S	S	S	S	S
NL408	Netherlands	Netherlands	lineage4	S	S	S	S	S	S	S	S
NL255	Netherlands	Netherlands	M. caprae	S	S	S	S	S	S	S	S
NL169	Netherlands	Netherlands	Haarlem	S	S	S	S	S	S	S	S
NL546	Netherlands	Netherlands	lineage4	S	S	S	S	S	S	S	S
NL350	Netherlands	Netherlands	lineage4	S	S	S	S	S	S	S	S
NL578	Netherlands	Netherlands	Delhi	S	S	S	S	S	S	S	S
NL144	Netherlands	Netherlands	Delhi	S	S	S	S	S	S	S	S
NL397	Netherlands	Netherlands	Delhi	S	S	S	n/a	S	S	S	S
NL239	Netherlands	Netherlands	Delhi	S	S	S	S	S	S	S	S
NL227	Netherlands	Netherlands	lineage4	S	S	S	S	S	S	S	S
NL199	Netherlands	Netherlands	LAM	S	S	S	n/a	S	S	S	S
NL375	Netherlands	Netherlands	Delhi	S	S	S	S	S	S	S	S
NL013	Netherlands	Netherlands	Delhi	S	S	S	S	F	S	S	S
NL377	Netherlands	Netherlands	lineage4	S	S	S	S	S	S	S	S
NL139	Netherlands	Netherlands	Delhi	S	S	S	S	S	S	S	S
NL063	Netherlands	Netherlands	Haarlem	S	S	S	S	S	S	S	S
NL318	Netherlands	Netherlands	lineage4	S	S	S	S	S	S	S	S
NL179	Netherlands	Netherlands	Haarlem	S	S	S	S	S	S	S	S
NL510	Netherlands	Netherlands	Haarlem	S	S	S	S	S	S	S	S
NL234	Netherlands	Netherlands	EAI	S	S	S	S	S	S	S	S
NL505	Netherlands	Netherlands	Haarlem	S	S	S	S	S	S	S	S
NL294	Netherlands	Netherlands	Tur	R	R	S	S	R	R	R	R
NL083	Netherlands	Netherlands	Haarlem	S	S	S	S	S	S	S	S
NL183	Netherlands	Netherlands	LAM	S	S	S	S	S	S	S	S
NL278	Netherlands	Netherlands	Ural	S	S	S	S	S	S	S	S
NL521	Netherlands	Netherlands	Haarlem	S	S	S	S	S	S	S	S
NL307	Netherlands	Netherlands	LAM	S	S	S	S	S	S	S	S
NL595	Netherlands	Netherlands	Haarlem	S	S	S	S	S	S	S	S
NL019	Netherlands	Netherlands	LAM	S	S	S	S	S	S	S	S
NL527	Netherlands	Netherlands	Delhi	S	S	S	S	S	S	S	S
NL484	Netherlands	Netherlands	Beijing	S	S	S	S	S	S	S	S
NL582	Netherlands	Netherlands	Delhi	S	S	S	S	S	S	S	S
NL413	Netherlands	Netherlands	Haarlem	S	S	S	S	S	S	S	S
NL477	Netherlands	Netherlands	LAM	S	S	S	S	S	S	S	S
NL268	Netherlands	Netherlands	BCG	S	S	S	R	S	S	S	R
NL585	Netherlands	Netherlands	LAM	S	S	S	S	S	S	S	S
NL252	Netherlands	Netherlands	Uganda	S	S	S	S	S	S	S	S
NL281	Netherlands	Netherlands	Beijing	S	S	S	S	S	S	U	S
NL436	Netherlands	Netherlands	West Africar	S	S	S	S	S	U	U	S
NL233	Netherlands	Netherlands	Haarlem	S	S	S	S	S	S	S	S
NL551	Netherlands	Netherlands	Delhi	S	S	S	S	S	S	S	S
NL305	Netherlands	Netherlands	Haarlem	S	S	S	S	S	S	S	S
NL333	Netherlands	Netherlands	Beijing	S	S	S	S	S	S	S	S
NL256	Netherlands	Netherlands	Haarlem	S	S	S	n/a	S	S	S	S
NL562	Netherlands	Netherlands	Haarlem	S	S	S	S	S	S	S	S
NL038	Netherlands	Netherlands	Delhi	S	S	S	S	S	S	S	S
NL312	Netherlands	Netherlands	Delhi	S	S	S	S	S	S	S	S
NL025	Netherlands	Netherlands	Beijing	S	S	S	S	S	S	S	S
NL314	Netherlands	Netherlands	Beijing	S	S	S	S	S	S	S	S
NL533	Netherlands	Netherlands	Delhi	S	S	S	S	S	S	S	S
NL614	Netherlands	Netherlands	Beijing	S	S	S	S	U	S	S	S
NL050	Netherlands	Netherlands	Beijing	S	S	S	S	U	S	U	S
NL216	Netherlands	Netherlands	lineage4	S	S	S	S	S	S	S	S
NL389	Netherlands	Netherlands	LAM	S	S	S	S	S	S	S	S
NL186	Netherlands	Netherlands	EAI	S	S	S	S	S	U	U	S
NL002	Netherlands	Netherlands	EAI	S	S	S	S	S	S	S	S
NL055	Netherlands	Netherlands	Uganda	S	S	S	S	S	S	S	S
NL504	Netherlands	Netherlands	LAM	S	S	S	n/a	S	S	S	S
NL368	Netherlands	Netherlands	Delhi	S	S	S	S	S	S	S	S
NL522	Netherlands	Netherlands	Delhi	S	S	S	S	S	S	S	S
NL016	Netherlands	Netherlands	Haarlem	S	S	S	S	S	S	S	S
NL223	Netherlands	Netherlands	LAM	R	S	S	S	R	S	R	S
NL411	Netherlands	Netherlands	LAM	S	S	S	S	S	S	S	S
NL074	Netherlands	Netherlands	LAM	S	S	S	S	S	S	S	S
NL257	Netherlands	Netherlands	LAM	S	S	S	S	S	S	S	S
NL218	Netherlands	Netherlands	EAI	S	S	S	S	F	S	F	S
NL064	Netherlands	Netherlands	Delhi	S	S	S	S	S	S	S	S
NL518	Netherlands	Netherlands	Haarlem	S	S	S	S	S	S	S	S
NL460	Netherlands	Netherlands	Haarlem	S	S	S	S	S	S	S	S
NL467	Netherlands	Netherlands	lineage4	S	S	S	S	S	S	S	S
NL516	Netherlands	Netherlands	Tur	R	R	n/a	R	R	R	R	R
NL150	Netherlands	Netherlands	Delhi	S	S	S	S	S	S	S	S
NL142	Netherlands	Netherlands	Delhi	S	S	S	S	S	S	S	S
NL373	Netherlands	Netherlands	M. bovis	S	S	S	R	S	S	S	R
NL506	Netherlands	Netherlands	Haarlem	S	S	S	S	S	S	S	S
NL116	Netherlands	Netherlands	lineage4	S	S	S	n/a	S	S	S	S
NL384	Netherlands	Netherlands	EAI	S	S	S	S	S	S	S	S
NL211	Netherlands	Netherlands	LAM	R	S	S	S	R	S	S	S
NL519	Netherlands	Netherlands	Haarlem	S	S	S	S	S	S	S	S
NL600	Netherlands	Netherlands	EAI	S	S	S	S	S	U	S	S
NL612	Netherlands	Netherlands	lineage4	S	S	S	S	S	S	R	S
NL031	Netherlands	Netherlands	Delhi	S	S	S	S	S	S	S	S
NL581	Netherlands	Netherlands	Delhi	S	S	S	S	S	S	U	S
NL357	Netherlands	Netherlands	Beijing	S	S	S	S	S	S	U	S
NL602	Netherlands	Netherlands	Haarlem	S	S	S	S	F	S	S	F
NL280	Netherlands	Netherlands	BCG	S	S	S	R	S	S	S	R
NL206	Netherlands	Netherlands	LAM	R	S	S	S	R	S	S	S
NL335	Netherlands	Netherlands	Beijing	S	S	S	S	S	S	S	S
NL040	Netherlands	Netherlands	Haarlem	S	S	S	S	S	S	S	S
NL593	Netherlands	Netherlands	lineage4	S	S	S	S	S	S	S	S
NL604	Netherlands	Netherlands	Haarlem	S	S	S	S	S	S	S	S
NL450	Netherlands	Netherlands	S-type	S	S	S	S	S	S	S	S
NL226	Netherlands	Netherlands	lineage4	S	S	S	S	S	S	S	S
NL191	Netherlands	Netherlands	lineage4	S	S	S	S	S	S	S	U
NL558	Netherlands	Netherlands	LAM	S	S	S	S	S	S	S	S
NL561	Netherlands	Netherlands	Haarlem	S	S	S	S	U	S	S	S
NL259	Netherlands	Netherlands	M. bovis	S	S	S	R	S	S	S	R
NL276	Netherlands	Netherlands	lineage4	S	S	S	S	S	S	S	S
NL267	Netherlands	Netherlands	Tur	S	S	S	n/a	S	S	S	S
NL345	Netherlands	Netherlands	Delhi	S	S	S	S	S	S	S	S
NL306	Netherlands	Netherlands	Haarlem	S	S	S	S	S	S	S	S
NL298	Netherlands	Netherlands	Tur	S	S	S	n/a	S	S	S	F
NL141	Netherlands	Netherlands	EAI	R	S	S	S	R	S	S	S
NL376	Netherlands	Netherlands	Delhi	S	S	S	S	S	S	U	S
NL448	Netherlands	Netherlands	LAM	S	S	S	S	S	S	S	S
NL547	Netherlands	Netherlands	M. orygis	S	S	S	n/a	S	S	U	S

NL029	Netherlands	Netherlands	BCG	S	S	S	R	S	S	S	R
NL188	Netherlands	Netherlands	Delhi	R	S	S	S	R	S	S	S
NL189	Netherlands	Netherlands	Delhi	S	S	S	S	S	S	S	S
NL432	Netherlands	Netherlands	LAM	S	S	S	S	S	S	S	S
NL092	Netherlands	Netherlands	Beijing	S	S	S	S	S	S	S	S
NL200	Netherlands	Netherlands	S-type	S	S	S	S	S	S	S	S
NL594	Netherlands	Netherlands	Haarlem	S	S	S	S	S	S	S	S
NL085	Netherlands	Netherlands	LAM	S	S	S	S	S	S	S	S
NL177	Netherlands	Netherlands	S-type	S	S	S	S	S	S	S	S
NL160	Netherlands	Netherlands	Haarlem	S	S	S	n/a	S	S	S	S
NL059	Netherlands	Netherlands	Haarlem	S	S	S	S	S	S	S	S
NL575	Netherlands	Netherlands	Delhi	S	S	S	S	S	U	S	S
NL406	Netherlands	Netherlands	Haarlem	S	S	S	S	S	S	S	S
NL374	Netherlands	Netherlands	lineage4	S	S	S	n/a	F	F	S	F
NL398	Netherlands	Netherlands	Delhi	S	S	S	S	S	S	S	S
NL399	Netherlands	Netherlands	Haarlem	S	S	S	S	S	S	U	S
NL568	Netherlands	Netherlands	EAI	S	S	S	S	S	S	S	S
NL336	Netherlands	Netherlands	EAI	S	S	S	S	S	S	S	S
NL319	Netherlands	Netherlands	Beijing	S	S	S	S	S	S	S	S
NL128	Netherlands	Netherlands	EAI	S	S	S	S	S	S	S	S
NL109	Netherlands	Netherlands	Uganda	S	S	S	S	S	S	S	S
NL184	Netherlands	Netherlands	Haarlem	S	S	S	S	S	S	S	S
NL140	Netherlands	Netherlands	Delhi	S	S	S	S	S	S	S	S
NL338	Netherlands	Netherlands	Tur	S	S	S	S	S	S	S	S
NL383	Netherlands	Netherlands	Cameroon	S	S	S	S	S	S	S	S
NL097	Netherlands	Netherlands	lineage4	S	S	S	S	S	S	S	S
NL021	Netherlands	Netherlands	Beijing	S	S	S	S	S	S	S	S
NL425	Netherlands	Netherlands	LAM	S	S	S	S	S	S	U	S
NL473	Netherlands	Netherlands	Haarlem	S	S	S	S	S	S	S	S
NL138	Netherlands	Netherlands	lineage4	S	S	S	S	S	S	S	S
NL564	Netherlands	Netherlands	Tur	S	S	S	S	S	S	S	S
NL271	Netherlands	Netherlands	Beijing	S	S	S	S	S	S	U	S
NL556	Netherlands	Netherlands	Delhi	S	S	S	S	S	S	S	S
NL514	Netherlands	Netherlands	LAM	n/a	S	S	n/a	U	S	S	S
NL205	Netherlands	Netherlands		S	S	S	R	S	S	S	F
NL131	Netherlands	Netherlands	Delhi	S	S	S	S	S	S	S	S
NL080	Netherlands	Netherlands	EAI	S	S	S	R	S	S	S	S
NL567	Netherlands	Netherlands	Beijing	S	S	S	S	S	S	S	S
NL342	Netherlands	Netherlands	lineage4	S	S	S	S	S	U	S	S
NL099	Netherlands	Netherlands	Haarlem	S	S	S	S	S	S	U	S
NL089	Netherlands	Netherlands	S-type	S	S	S	n/a	S	S	U	S
NL403	Netherlands	Netherlands	lineage4	S	S	S	S	S	S	U	S
NL279	Netherlands	Netherlands	LAM	S	S	S	S	S	S	S	S
NL118	Netherlands	Netherlands	lineage4	S	S	S	S	S	S	S	S
NL597	Netherlands	Netherlands	Tur	S	S	S	S	S	S	S	S
NL094	Netherlands	Netherlands	LAM	S	S	S	S	S	S	S	S
NL407	Netherlands	Netherlands	lineage4	S	S	S	S	F	U	F	S
NL468	Netherlands	Netherlands	Delhi	S	S	S	S	F	F	U	S
NL316	Netherlands	Netherlands	Tur	S	S	S	n/a	S	S	S	S
NL101	Netherlands	Netherlands	lineage4	S	S	S	S	S	S	S	S
NL075	Netherlands	Netherlands	X-type	S	S	S	n/a	S	S	S	S
NL231	Netherlands	Netherlands	S-type	S	S	S	S	S	S	S	S
NL577	Netherlands	Netherlands	Delhi	S	S	S	S	S	S	S	S
NL584	Netherlands	Netherlands	Beijing	S	S	S	S	S	S	S	S
NL208	Netherlands	Netherlands	Beijing	R	R	R	R	R	R	R	R
NL010	Netherlands	Netherlands	S-type	S	S	S	S	S	S	S	S
NL258	Netherlands	Netherlands	Delhi	S	S	S	S	S	S	S	S
NL344	Netherlands	Netherlands	Delhi	S	S	S	S	S	S	S	S
NL117	Netherlands	Netherlands	LAM	S	S	S	S	S	S	S	S
NL470	Netherlands	Netherlands	LAM	S	S	S	S	S	S	S	S
NL369	Netherlands	Netherlands	Delhi	S	S	S	S	S	S	U	S
NL444	Netherlands	Netherlands	Delhi	S	S	S	S	S	S	S	S
NL006	Netherlands	Netherlands	Delhi	S	S	S	S	S	S	S	S
NL367	Netherlands	Netherlands	EAI	S	S	S	S	S	U	S	S
NL362	Netherlands	Netherlands	LAM	S	S	S	S	S	S	S	S
NL277	Netherlands	Netherlands	lineage4	S	S	S	S	S	S	S	S
NL197	Netherlands	Netherlands	X-type	S	S	S	S	S	S	S	S
NL093	Netherlands	Netherlands	lineage4	S	S	S	S	U	S	S	S
NL579	Netherlands	Netherlands	Delhi	R	S	S	S	R	S	S	S
NL054	Netherlands	Netherlands	Haarlem	S	S	S	S	S	S	S	S
NL353	Netherlands	Netherlands	Haarlem	R	S	S	S	R	S	S	S
NL424	Netherlands	Netherlands	Haarlem	S	S	S	S	U	F	U	S
NL541	Netherlands	Netherlands	X-type	S	S	S	S	S	S	S	S
NL429	Netherlands	Netherlands	Delhi	S	S	S	S	S	S	S	S
NL037	Netherlands	Netherlands	Delhi	R	S	S	S	R	S	S	S
NL261	Netherlands	Netherlands	Delhi	S	S	S	S	S	S	S	S
NL091	Netherlands	Netherlands	Delhi	S	S	S	S	S	S	S	S
NL161	Netherlands	Netherlands	Delhi	S	S	S	S	S	S	S	S
NL386	Netherlands	Netherlands	Delhi	S	S	S	S	S	S	S	S
NL098	Netherlands	Netherlands	Haarlem	S	S	S	S	S	S	S	S
NL536	Netherlands	Netherlands	lineage4	S	S	S	S	S	S	S	S
NL395	Netherlands	Netherlands	Haarlem	S	S	S	S	S	S	S	S
NL481	Netherlands	Netherlands	Delhi	S	S	S	S	S	S	S	S
NL366	Netherlands	Netherlands	Beijing	R	R	n/a	R	R	R	R	R
NL494	Netherlands	Netherlands	Haarlem	S	S	S	S	U	S	S	S
NL219	Netherlands	Netherlands	LAM	S	S	S	S	S	S	S	S
NL606	Netherlands	Netherlands	lineage4	S	S	S	S	S	S	S	S
NL248	Netherlands	Netherlands	LAM	S	S	S	S	S	S	S	S
NL463	Netherlands	Netherlands	M. bovis	S	S	S	R	S	S	S	R
NL228	Netherlands	Netherlands	lineage4	S	S	S	n/a	F	U	S	F
NL222	Netherlands	Netherlands	lineage4	S	S	S	S	S	S	S	S
NL192	Netherlands	Netherlands	Delhi	S	S	S	S	S	S	U	S
NL194	Netherlands	Netherlands	Ural	S	S	S	S	S	S	S	S
NL052	Netherlands	Netherlands	Haarlem	S	S	S	S	S	S	S	S
NL129	Netherlands	Netherlands	lineage4	S	S	S	S	S	S	S	S
NL260	Netherlands	Netherlands	Haarlem	S	S	S	S	S	S	S	S
NL531	Netherlands	Netherlands	LAM	S	S	S	S	S	S	S	S
NL014	Netherlands	Netherlands	Haarlem	S	S	S	S	S	S	S	S
NL036	Netherlands	Netherlands	LAM	S	S	S	S	S	S	S	S
NL359	Netherlands	Netherlands	Haarlem	S	S	S	S	S	S	S	S
NL327	Netherlands	Netherlands	Delhi	S	S	S	S	S	S	S	S
NL459	Netherlands	Netherlands	lineage4	S	S	S	S	S	S	S	S
NL339	Netherlands	Netherlands	Delhi	S	S	S	S	S	S	U	S
NL251	Netherlands	Netherlands	Tur	R	R	S	S	R	R	S	S
NL502	Netherlands	Netherlands	lineage4	S	S	S	S	S	S	S	S
NL560	Netherlands	Netherlands	Delhi	S	S	S	S	S	F	U	S
NL610	Netherlands	Netherlands	Delhi	S	S	S	n/a	S	S	S	S
NL282	Netherlands	Netherlands	Haarlem	S	S	S	S	S	S	S	S
NL472	Netherlands	Netherlands	Haarlem	S	S	S	S	S	S	S	S
NL520	Netherlands	Netherlands	LAM	S	S	S	n/a	S	S	S	S
NL288	Netherlands	Netherlands	Delhi	S	S	S	S	S	S	S	S
NL475	Netherlands	Netherlands	LAM	S	S	S	S	S	S	S	S
NL071	Netherlands	Netherlands	lineage4	S	S	S	S	S	S	S	S
NL310	Netherlands	Netherlands	lineage4	S	S	S	S	S	S	S	S
NL009	Netherlands	Netherlands	Delhi	R	S	S	S	R	S	S	S
NL274	Netherlands	Netherlands	Delhi	S	S	S	S	S	S	S	S

NL446	Netherlands	Netherlands	EAI	S	S	S	S	S	S	S	S
NL103	Netherlands	Netherlands	LAM	S	S	S	S	S	S	S	S
NL499	Netherlands	Netherlands	Haarlem	S	S	S	S	S	S	S	S
NL608	Netherlands	Netherlands	LAM	S	S	S	S	S	S	U	S
NL028	Netherlands	Netherlands	lineage4	S	S	S	S	S	S	S	S
NL469	Netherlands	Netherlands	LAM	S	S	S	S	S	S	S	S
NL495	Netherlands	Netherlands	Haarlem	S	S	S	S	S	S	S	S
NL291	Netherlands	Netherlands	West Africar	S	S	S	S	S	S	U	S
NL418	Netherlands	Netherlands	Beijing	n/a	R	S	S	R	R	U	S
NL110	Netherlands	Netherlands	Delhi	S	S	S	S	S	S	S	S
NL489	Netherlands	Netherlands	lineage4	S	S	S	S	S	S	S	S
NL119	Netherlands	Netherlands	LAM	R	S	S	S	R	S	S	S
NL348	Netherlands	Netherlands	lineage4	S	S	S	S	S	S	S	S
NL388	Netherlands	Netherlands	Haarlem	S	S	S	n/a	S	S	S	S
NL426	Netherlands	Netherlands	Delhi	S	S	S	S	S	S	S	S
NL394	Netherlands	Netherlands	Haarlem	S	S	S	S	S	S	S	S
NL523	Netherlands	Netherlands	lineage4	S	S	S	S	S	S	S	S
NL497	Netherlands	Netherlands	lineage4	S	S	S	S	S	S	S	S
NL465	Netherlands	Netherlands	Delhi	S	S	S	S	S	S	U	S
NL046	Netherlands	Netherlands	BCG	S	S	S	R	S	S	S	R
NL453	Netherlands	Netherlands	lineage4	S	S	S	S	F	S	U	S
NL020	Netherlands	Netherlands	Beijing	S	S	S	S	S	S	S	S
NL464	Netherlands	Netherlands	lineage4	S	S	S	S	S	S	S	S
NL240	Netherlands	Netherlands	Haarlem	S	S	S	S	S	S	S	S
NL017	Netherlands	Netherlands	Delhi	S	S	S	S	S	S	S	S
NL174	Netherlands	Netherlands	lineage4	S	S	S	S	S	S	S	S
NL482	Netherlands	Netherlands	lineage4	S	S	S	S	S	S	S	S
NL053	Netherlands	Netherlands	Delhi	S	S	S	S	S	S	S	S
NL070	Netherlands	Netherlands	Delhi	S	S	S	S	S	S	S	S
NL115	Netherlands	Netherlands	Haarlem	S	S	S	S	S	S	U	S
NL329	Netherlands	Netherlands	Haarlem	S	S	S	S	S	S	S	S
NL440	Netherlands	Netherlands	LAM	S	S	S	S	S	S	S	S
NL007	Netherlands	Netherlands	Haarlem	S	S	S	S	S	S	S	S
NL000	Netherlands	Netherlands	Delhi	S	S	S	S	S	S	U	S
NL124	Netherlands	Netherlands	LAM	S	S	S	S	S	S	S	S
NL236	Netherlands	Netherlands	EAI	S	S	S	n/a	S	S	S	S
NL235	Netherlands	Netherlands	Cameroon	S	S	S	S	S	S	U	S
NL596	Netherlands	Netherlands	Cameroon	S	S	S	S	S	S	S	S
NL112	Netherlands	Netherlands	M. bovis	S	S	S	R	U	S	U	R
NL507	Netherlands	Netherlands	Haarlem	S	S	S	n/a	S	S	U	S
NL557	Netherlands	Netherlands	Delhi	S	S	S	n/a	S	S	S	S
NL301	Netherlands	Netherlands	Tur	S	S	S	n/a	S	S	S	S
NL488	Netherlands	Netherlands	LAM	S	S	S	S	S	S	S	S
NL011	Netherlands	Netherlands	lineage4	S	S	S	S	S	S	S	S
NL311	Netherlands	Netherlands	Beijing	S	S	S	S	S	U	S	S
NL320	Netherlands	Netherlands	S-type	S	S	S	S	S	S	S	S
NL326	Netherlands	Netherlands	lineage4	S	S	S	S	S	S	S	S
NL589	Netherlands	Netherlands	Beijing	S	S	S	S	S	S	U	S
NL461	Netherlands	Netherlands	LAM	S	S	S	S	S	S	S	S
NL364	Netherlands	Netherlands	Beijing	S	S	S	S	S	S	S	S
NL272	Netherlands	Netherlands	Delhi	S	S	S	S	S	S	S	S
CRYPAK227-17	Pakistan	Pakistan	Delhi	R	S	S	S	R	S	S	S
CRYPAK239-17	Pakistan	Pakistan	Delhi	R	R	S	R	R	R	R	R
CRYPAK319-17	Pakistan	Pakistan	Delhi	R	R	S	R	R	R	R	U
CRYPAK422-16	Pakistan	Pakistan	Delhi	R	R	R	R	R	R	R	U
CRYPAK47-17	Pakistan	Pakistan	X-type	R	R	R	R	R	R	R	R
CRYPAK479-16	Pakistan	Pakistan	Delhi	R	R	R	S	R	R	R	R
CRYPAK434-16	Pakistan	Pakistan	EAI	R	R	R	S	R	R	R	S
CRYPAK212-17	Pakistan	Pakistan	Delhi	R	R	R	R	R	R	R	R
CRYPAK175-17	Pakistan	Pakistan	Delhi	R	R	S	S	R	R	S	S
CRYPAK204-17	Pakistan	Pakistan	Delhi	S	S	S	S	S	S	S	S
CRYPAK86-17	Pakistan	Pakistan	Cameroon	R	R	S	S	R	R	S	R
CRYPAK560-16	Pakistan	Pakistan	Beijing	R	R	S	S	R	F	F	S
CRYPAK273-17	Pakistan	Pakistan	Delhi	R	R	S	R	R	R	R	F
CRYPAK439-16	Pakistan	Pakistan	Delhi	R	R	S	S	S	S	S	S
CRYPAK470-16	Pakistan	Pakistan	Delhi	R	R	R	R	R	R	R	R
332-16	Pakistan	Pakistan	Delhi	R	R	R	S	R	R	S	S
CRYPAK561-16	Pakistan	Pakistan	Delhi	R	R	S	S	R	R	F	S
CRYPAK468-16	Pakistan	Pakistan	Delhi	R	R	R	S	S	R	S	S
CRYPAK419-16	Pakistan	Pakistan	Delhi	R	R	R	S	R	R	R	U
CRYPAK87-17	Pakistan	Pakistan	Delhi	R	R	R	S	R	R	R	U
CRYPAK396-16	Pakistan	Pakistan	EAI	R	R	R	R	R	R	S	R
CRYPAK128-17	Pakistan	Pakistan	Delhi	S	R	S	S	S	R	S	S
CRYPAK152-17	Pakistan	Pakistan	lineage4	R	R	R	R	R	R	R	R
CRYPAK420-16	Pakistan	Pakistan	Delhi	R	R	R	R	R	R	R	R
CRYPAK282-17	Pakistan	Pakistan	Delhi	R	S	S	S	R	S	S	S
CRYPAK295-17	Pakistan	Pakistan	Delhi	R	R	S	S	R	R	U	S
CRYPAK512-16	Pakistan	Pakistan	Ural	R	R	R	R	R	R	R	R
CRYPAK240-17	Pakistan	Pakistan	Delhi	R	R	S	S	R	R	R	S
CRYPAK49-17	Pakistan	Pakistan	Delhi	R	R	R	S	R	R	R	S
CRYPAK262-17	Pakistan	Pakistan	Delhi	S	S	n/a	S	S	S	S	S
CRYPAK398-16	Pakistan	Pakistan	Delhi	R	R	S	S	R	R	S	S
CRYPAK169-17	Pakistan	Pakistan	Delhi	R	R	S	S	R	F	S	S
CRYPAK78-17	Pakistan	Pakistan	Delhi	R	R	R	R	R	R	R	R
CRYPAK267-17	Pakistan	Pakistan	Delhi	S	S	S	S	S	S	S	S
CRYPAK137-17	Pakistan	Pakistan	Delhi	R	R	S	S	R	R	R	S
CRYPAK417-16	Pakistan	Pakistan	Beijing	R	R	S	S	R	R	U	S
CRYPAK418-16	Pakistan	Pakistan	Tur	R	R	R	R	R	R	R	U
CRYPAK548-16	Pakistan	Pakistan	Delhi	R	R	R	S	R	R	F	F
CRYPAK198-17	Pakistan	Pakistan	Delhi	R	R	R	S	F	R	U	S
CRYPAK558-16	Pakistan	Pakistan	lineage4	R	R	R	R	R	R	R	R
CRYPAK112-17	Pakistan	Pakistan	Delhi	R	R	R	R	R	R	R	R
CRYPAK209-17	Pakistan	Pakistan	Delhi	R	R	S	S	U	R	U	S
CRYPAK80-17	Pakistan	Pakistan	Beijing	R	R	R	R	R	R	R	R
CRYPAK121-17	Pakistan	Pakistan	EAI	R	R	R	R	R	R	F	R
CRYPAK254-17	Pakistan	Pakistan	Delhi	S	S	S	S	S	S	S	S
CRYPAK221-17	Pakistan	Pakistan	Delhi	R	R	R	R	R	R	R	U
321-16	Pakistan	Pakistan	Delhi	R	R	S	S	R	R	R	U
CRYPAK45-17	Pakistan	Pakistan	Delhi	R	R	R	S	R	R	R	S
CRYPAK416-16	Pakistan	Pakistan	Tur	R	R	R	S	R	R	R	S
CRYPAK322-17	Pakistan	Pakistan	Delhi	R	R	R	R	R	R	R	R
CRYPAK480-16	Pakistan	Pakistan	lineage4	R	R	R	S	R	R	R	S
CRYPAK433-16	Pakistan	Pakistan	Beijing	R	R	R	S	R	R	R	U
CRYPAK299-17	Pakistan	Pakistan	Delhi	S	S	S	S	S	S	S	S
CRYPAK555-16	Pakistan	Pakistan	Delhi	R	R	S	S	R	R	R	S
CRYPAK117-17	Pakistan	Pakistan	Beijing	R	R	R	S	R	R	R	S
CRYPAK378-16	Pakistan	Pakistan	Delhi	R	R	R	S	R	R	R	R
CRYPAK44-17	Pakistan	Pakistan	Delhi	R	R	S	S	R	R	R	S
CRYPAK465-16	Pakistan	Pakistan	lineage4	R	R	R	S	R	R	R	R
CRYPAK278-17	Pakistan	Pakistan	Delhi	S	S	S	S	S	S	S	S
CRYPAK494-16	Pakistan	Pakistan	Delhi	R	R	R	S	R	R	R	S
CRYPAK244-17	Pakistan	Pakistan	lineage4	R	R	R	R	R	R	R	R
CRYPAK189-17	Pakistan	Pakistan	Delhi	S	S	S	n/a	S	S	S	S
CRYPAK216-17	Pakistan	Pakistan	Delhi	R	R	S	R	R	R	U	R
CRYPAK95-17	Pakistan	Pakistan	Delhi	R	R	R	S	R	R	R	S
CRYPAK200-17	Pakistan	Pakistan	Delhi	R	R	R	n/a	R	R	R	R

CRYPAK466-16	Pakistan	Pakistan	Delhi	R	R	S	R	R	R	R	R
CRYPAK73-17	Pakistan	Pakistan	Delhi	R	R	R	S	R	R	U	S
CRYPAK359-16	Pakistan	Pakistan	Delhi	R	R	R	R	R	R	R	R
CRYPAK219-17	Pakistan	Pakistan	Delhi	R	R	S	S	R	R	S	S
CRYPAK493-16	Pakistan	Pakistan	Delhi	R	R	S	S	R	R	S	S
CRYPAK471-16	Pakistan	Pakistan	Delhi	R	R	R	R	R	R	R	R
CRYPAK550-16	Pakistan	Pakistan	Delhi	R	R	R	S	R	R	R	R
CRYPAK361-16	Pakistan	Pakistan	Delhi	R	R	R	S	R	R	R	S
CRYPAK113-17	Pakistan	Pakistan	lineage4	R	R	R	S	R	R	F	S
CRYPAK71-17	Pakistan	Pakistan	Delhi	R	R	S	S	R	R	R	S
CRYPAK506-16	Pakistan	Pakistan	Beijing	R	R	R	R	U	R	R	F
315-16	Pakistan	Pakistan	Delhi	R	R	R	S	R	R	R	S
CRYPAK491-16	Pakistan	Pakistan	Delhi	R	R	R	R	U	R	R	F
CRYPAK141-17	Pakistan	Pakistan	Delhi	R	R	R	R	R	R	R	R
CRYPAK328-17	Pakistan	Pakistan	Delhi	S	S	S	n/a	S	S	S	S
CRYPAK191-17	Pakistan	Pakistan	Delhi	S	S	S	S	S	S	S	S
CRYPAK423-16	Pakistan	Pakistan	Delhi	R	R	S	S	R	R	R	S
CRYPAK237-17	Pakistan	Pakistan	Delhi	R	R	R	S	R	R	S	R
CRYPAK109-17	Pakistan	Pakistan	Delhi	R	R	R	S	R	R	R	S
CRYPAK395-16	Pakistan	Pakistan	Delhi	R	R	R	S	R	R	S	S
333-16	Pakistan	Pakistan	Delhi	R	R	R	R	R	R	R	R
CRYPAK76-17	Pakistan	Pakistan	Delhi	R	R	R	S	R	R	R	R
CRYPAK500-16	Pakistan	Pakistan	Delhi	R	R	R	S	R	R	R	R
CRYPAK293-17	Pakistan	Pakistan	Delhi	S	S	S	S	S	S	S	S
CRYPAK153-17	Pakistan	Pakistan	lineage4	S	S	S	S	U	S	S	S
CRYPAK171-17	Pakistan	Pakistan	Delhi	R	R	R	S	R	R	R	S
CRYPAK104-17	Pakistan	Pakistan	Delhi	R	R	R	R	R	R	R	R
CRYPAK393-16	Pakistan	Pakistan	lineage4	R	R	R	R	R	R	R	R
CRYPAK211-17	Pakistan	Pakistan	Delhi	R	R	R	S	F	R	F	S
CRYPAK339-16	Pakistan	Pakistan	Delhi	R	R	R	R	R	F	F	F
CRYPAK270-17	Pakistan	Pakistan	Delhi	S	S	S	S	S	S	S	S
CRYPAK187-17	Pakistan	Pakistan	Delhi	R	R	S	S	R	R	R	S
CRYPAK190-17	Pakistan	Pakistan	Delhi	S	S	S	S	S	S	S	S
CRYPAK384-16	Pakistan	Pakistan	Delhi	R	R	R	R	R	R	R	R
CRYPAK315-17	Pakistan	Pakistan	Delhi	S	S	S	S	S	U	S	S
CRYPAK389-16	Pakistan	Pakistan	lineage4	R	R	S	R	R	R	R	R
CRYPAK210-17	Pakistan	Pakistan	Delhi	R	R	R	n/a	R	R	R	S
CRYPAK400-16	Pakistan	Pakistan	Delhi	R	R	R	S	R	R	R	U
CRYPAK444-16	Pakistan	Pakistan	Delhi	R	R	R	S	R	R	F	S
CRYPAK562-16	Pakistan	Pakistan	Delhi	R	R	R	S	R	R	R	S
CRYPAK385-16	Pakistan	Pakistan	Tur	R	R	R	R	R	R	R	S
CRYPAK324-17	Pakistan	Pakistan	Delhi	R	R	S	S	R	R	S	S
CRYPAK106-17	Pakistan	Pakistan	EAI	R	R	R	R	R	R	R	U
CRYPAK263-17	Pakistan	Pakistan	Delhi	R	R	R	R	R	R	R	R
CRYPAK82-17	Pakistan	Pakistan	Delhi	R	R	S	S	R	R	U	S
CRYPAK443-16	Pakistan	Pakistan	Delhi	R	R	R	S	R	R	R	S
CRYPAK111-17	Pakistan	Pakistan	Delhi	R	R	S	S	R	R	R	S
CRYPAK343-16	Pakistan	Pakistan	Delhi	R	R	S	S	S	R	S	S
CRYPAK120-17	Pakistan	Pakistan	Delhi	R	R	R	R	R	R	R	R
CRYPAK436-16	Pakistan	Pakistan	Delhi	R	R	S	R	S	R	F	R
CRYPAK556-16	Pakistan	Pakistan	Delhi	R	R	R	R	R	R	R	R
CRYPAK255-17	Pakistan	Pakistan	lineage4	S	S	S	S	S	S	S	S
CRYPAK214-17	Pakistan	Pakistan	Delhi	R	R	R	S	R	R	U	S
CRYPAK247-17	Pakistan	Pakistan	Delhi	R	S	S	S	S	S	S	S
CRYPAK346-16	Pakistan	Pakistan	lineage4	R	R	R	R	R	R	R	R
CRYPAK264-17	Pakistan	Pakistan	Delhi	R	R	R	S	U	R	R	S
CRYPAK261-17	Pakistan	Pakistan	Delhi	R	R	R	S	R	R	F	S
CRYPAK207-17	Pakistan	Pakistan	Delhi	S	S	S	S	S	S	S	S
CRYPAK552-16	Pakistan	Pakistan	EAI	R	R	R	R	R	R	R	R
CRYPAK430-16	Pakistan	Pakistan	EAI	R	R	S	S	F	R	S	S
CRYPAK447-16	Pakistan	Pakistan	Delhi	R	R	R	S	R	R	R	S
CRYPAK450-16	Pakistan	Pakistan	Delhi	R	R	R	S	R	R	R	S
CRYPAK499-16	Pakistan	Pakistan	Delhi	R	R	R	S	R	R	R	R
CRYPAK498-16	Pakistan	Pakistan	Delhi	R	R	S	S	R	R	S	U
CRYPAK511-16	Pakistan	Pakistan	Delhi	R	R	S	S	R	R	R	S
CRYPAK52-17	Pakistan	Pakistan	Delhi	R	R	S	R	R	R	U	R
CRYPAK510-16	Pakistan	Pakistan	Delhi	R	R	R	S	R	R	R	S
CRYPAK488-16	Pakistan	Pakistan	Tur	R	R	R	R	R	R	R	F
CRYPAK291-17	Pakistan	Pakistan	Delhi	R	R	R	R	R	R	R	U
CRY382-16	Pakistan	Pakistan	Delhi	R	R	R	S	R	R	R	U
CRYPAK288-17	Pakistan	Pakistan	Delhi	R	R	R	R	R	R	R	R
CRYPAK388-16	Pakistan	Pakistan	Delhi	R	R	S	S	R	R	U	S
CRY380-16	Pakistan	Pakistan	Delhi	R	R	R	R	R	R	R	F
CRYPAK63-17	Pakistan	Pakistan	Ural	R	R	R	R	R	R	R	U
CRYPAK387-16	Pakistan	Pakistan	Delhi	R	R	R	R	R	R	R	R
CRYPAK358-16	Pakistan	Pakistan	lineage4	R	R	R	R	R	R	R	R
CRYPAK242-17	Pakistan	Pakistan	lineage4	S	R	S	S	S	R	S	S
CRYPAK284-17	Pakistan	Pakistan	Delhi	S	S	S	S	S	S	S	S
CRYPAK343-17	Pakistan	Pakistan	Delhi	S	S	S	n/a	S	S	S	S
CRYPAK505-16	Pakistan	Pakistan	Beijing	R	R	R	S	R	R	R	R
CRYPAK454-16	Pakistan	Pakistan	Delhi	R	R	R	S	R	R	R	U
CRYPAK481-16	Pakistan	Pakistan	lineage4	R	R	S	S	R	R	R	R
316-16	Pakistan	Pakistan	lineage4	R	R	R	R	R	R	R	R
CRYPAK50-17	Pakistan	Pakistan	Delhi	R	R	S	S	R	R	S	S
CRYPAK305-17	Pakistan	Pakistan	Delhi	S	S	S	S	S	S	S	S
CRYPAK233-17	Pakistan	Pakistan	Delhi	R	R	S	R	F	R	U	R
CRYPAK301-17	Pakistan	Pakistan	EAI	S	S	S	S	S	S	U	S
CRYPAK541-16	Pakistan	Pakistan	Delhi	R	R	R	S	R	R	R	S
CRYPAK483-16	Pakistan	Pakistan	lineage4	R	R	S	S	R	R	S	S
325-16	Pakistan	Pakistan	Delhi	R	R	R	S	R	R	R	S
CRYPAK126-17	Pakistan	Pakistan	lineage4	R	R	R	R	R	R	R	R
CRYPAK336-17	Pakistan	Pakistan	lineage4	R	R	R	R	R	R	R	R
CRYPAK357-16	Pakistan	Pakistan	Delhi	R	R	S	S	R	R	S	S
CRYPAK201-17	Pakistan	Pakistan	lineage4	R	R	R	R	R	R	R	R
CRYPAK350-16	Pakistan	Pakistan	Delhi	R	R	R	S	R	R	R	R
CRYPAK245-17	Pakistan	Pakistan	Delhi	R	R	R	R	R	R	R	F
CRYPAK164-17	Pakistan	Pakistan	lineage4	R	R	R	R	R	R	U	R
CRYPAK230-17	Pakistan	Pakistan	Delhi	S	S	S	S	S	S	S	S
CRYPAK296-17	Pakistan	Pakistan	lineage4	R	R	R	S	R	R	R	S
331-16	Pakistan	Pakistan	lineage4	R	R	R	R	R	R	R	R
320-16	Pakistan	Pakistan	Delhi	R	R	R	R	R	R	R	R
330-16	Pakistan	Pakistan	lineage4	R	R	S	R	R	R	R	R
CRYPAK478-16	Pakistan	Pakistan	Delhi	R	R	R	S	R	F	F	F
319-16	Pakistan	Pakistan		R	R	S	R	F	F	F	F
CRYPAK314-17	Pakistan	Pakistan	Cameroon	R	R	S	S	R	R	R	S
327-16	Pakistan	Pakistan	Delhi	R	R	S	S	R	R	R	S
CRYPAK127-17	Pakistan	Pakistan	Delhi	R	R	S	S	R	R	R	S
CRYPAK60-17	Pakistan	Pakistan	Delhi	R	R	S	S	R	R	S	S
CRYPAK99-17	Pakistan	Pakistan	Beijing	R	R	R	R	R	R	R	R
CRYPAK331-17	Pakistan	Pakistan	lineage4	R	R	R	S	R	R	R	S
CRYPAK330-17	Pakistan	Pakistan	Beijing	R	R	R	S	R	R	U	S
CRYPAK265-17	Pakistan	Pakistan	Delhi	R	R	R	S	R	R	R	R
CRYPAK206-17	Pakistan	Pakistan	Delhi	S	S	S	S	S	S	S	S
CRYPAK236-17	Pakistan	Pakistan	EAI	R	R	R	S	R	R	R	S
CRYPAK102-17	Pakistan	Pakistan	Delhi	R	R	S	S	R	R	S	S
CRYPAK563-16	Pakistan	Pakistan	Delhi	R	R	R	S	R	R	R	S

CRYPAK51-17	Pakistan	Pakistan	Delhi	S	R	S	S	U	R	S	S
CRYPAK448-16	Pakistan	Pakistan	EAI	R	R	S	R	R	R	R	F
CRYPAK421-16	Pakistan	Pakistan	Delhi	R	R	R	R	R	R	R	R
CRYPAK186-17	Pakistan	Pakistan	Delhi	S	S	S	S	S	S	S	S
CRYPAK48-17	Pakistan	Pakistan	Delhi	S	S	S	S	S	R	U	S
CRYPAK356-16	Pakistan	Pakistan	Delhi	R	R	R	n/a	R	R	R	U
CRYPAK37-17	Pakistan	Pakistan	Delhi	R	R	S	S	R	R	U	S
CRYPAK360-16	Pakistan	Pakistan	Delhi	R	R	R	R	R	R	R	F
CRYPAK317-17	Pakistan	Pakistan	EAI	S	R	S	S	R	R	U	S
CRYPAK250-17	Pakistan	Pakistan	Tur	R	R	R	S	R	R	R	F
CRYPAK85-17	Pakistan	Pakistan	lineage4	R	R	R	R	R	R	R	R
335-16	Pakistan	Pakistan	lineage4	R	R	R	R	R	R	R	R
CRYPAK147-17	Pakistan	Pakistan	Delhi	R	R	R	S	R	R	R	S
CRY342-16	Pakistan	Pakistan	Tur	R	R	R	S	R	R	R	S
CRYPAK394-16	Pakistan	Pakistan	lineage4	R	R	R	R	R	R	R	R
CRYPAK208-17	Pakistan	Pakistan	Delhi	R	R	S	S	R	R	S	S
CRYPAK503-16	Pakistan	Pakistan	Delhi	R	R	R	R	F	R	R	R
CRYPAK327-17	Pakistan	Pakistan	Delhi	R	R	R	R	R	R	U	R
CRYPAK313-17	Pakistan	Pakistan	Delhi	S	S	S	S	S	S	S	S
CRYPAK308-17	Pakistan	Pakistan	Delhi	S	S	S	S	S	S	S	S
CRYPAK184-17	Pakistan	Pakistan	Delhi	R	R	R	S	U	R	R	F
CRYPAK551-16	Pakistan	Pakistan	Delhi	S	S	S	S	S	R	S	S
CRYPAK281-17	Pakistan	Pakistan	Delhi	S	R	R	S	U	R	R	S
CRYPAK246-17	Pakistan	Pakistan	EAI	R	R	R	R	R	R	U	R
CRYPAK241-17	Pakistan	Pakistan	lineage4	S	S	S	S	S	S	S	S
CRYPAK349-16	Pakistan	Pakistan	EAI	R	R	R	R	R	R	R	U
322-16	Pakistan	Pakistan	Delhi	R	R	R	R	R	R	R	R
CRYPAK266-17	Pakistan	Pakistan	Delhi	R	S	n/a	S	S	S	S	S
CRYPAK437-16	Pakistan	Pakistan	Delhi	S	S	S	S	S	R	S	S
CRYPAK192-17	Pakistan	Pakistan	Delhi	S	S	S	S	S	S	S	S
CRYPAK474-16	Pakistan	Pakistan	Delhi	R	R	R	R	R	R	R	R
CRYPAK490-16	Pakistan	Pakistan	Delhi	R	R	R	S	R	R	R	R
CRYPAK249-17	Pakistan	Pakistan	Delhi	R	R	S	R	R	R	S	R
CRYPAK77-17	Pakistan	Pakistan	Delhi	R	R	R	R	R	R	R	R
CRYPAK243-17	Pakistan	Pakistan	Delhi	R	R	R	R	R	R	R	R
CRYPAK124-17	Pakistan	Pakistan	Delhi	R	R	S	R	R	R	S	R
CRYPAK485-16	Pakistan	Pakistan	Beijing	R	R	R	R	R	R	R	R
CRYPAK504-16	Pakistan	Pakistan	lineage4	R	R	R	R	R	R	R	R
CRYPAK139-17	Pakistan	Pakistan	Delhi	R	R	S	S	F	R	R	S
336-16	Pakistan	Pakistan	EAI	R	R	R	R	R	R	R	S
318-16	Pakistan	Pakistan	Beijing	R	R	R	R	R	R	R	R
CRYPAK64-17	Pakistan	Pakistan	lineage4	R	R	R	R	R	R	R	R
CRYPAK61-17	Pakistan	Pakistan	Tur	R	R	R	S	R	R	R	U
CRYPAK440-16	Pakistan	Pakistan	Delhi	R	R	S	S	R	R	R	S
CRYPAK310-17	Pakistan	Pakistan	Delhi	R	S	n/a	S	R	R	U	S
CRYPAK93-17	Pakistan	Pakistan	Delhi	R	S	R	S	R	R	R	S
CRYPAK431-16	Pakistan	Pakistan	lineage4	R	R	R	R	R	R	R	R
CRYPAK325-17	Pakistan	Pakistan	Delhi	R	R	R	S	F	R	F	S
CRYPAK509-16	Pakistan	Pakistan	Delhi	R	R	S	R	R	R	R	S
CRYPAK425-16	Pakistan	Pakistan	lineage4	R	R	R	R	R	R	R	R
CRYPAK302-17	Pakistan	Pakistan	Beijing	R	R	R	R	R	R	R	R
CRYPAK43-17	Pakistan	Pakistan	Delhi	R	R	R	R	R	R	R	R
CRYPAK105-17	Pakistan	Pakistan	EAI	R	R	R	R	R	R	R	R
CRYPAK544-16	Pakistan	Pakistan	Delhi	R	R	S	S	F	R	R	F
CRYPAK492-16	Pakistan	Pakistan	Delhi	R	R	S	S	R	R	S	S
CRYPAK543-16	Pakistan	Pakistan	Delhi	R	R	R	S	R	R	R	R
CRYPAK383-16	Pakistan	Pakistan	Delhi	R	R	S	S	R	R	R	S
CRYPAK457-16	Pakistan	Pakistan	Delhi	R	R	R	R	R	R	R	R
CRYPAK445-16	Pakistan	Pakistan	Delhi	R	R	S	R	R	R	U	R
CRYPAK148-17	Pakistan	Pakistan	Delhi	R	R	R	R	R	R	U	R
CRYPAK342-17	Pakistan	Pakistan	Delhi	S	S	S	n/a	S	S	S	S
CRYPAK280-17	Pakistan	Pakistan	Delhi	S	S	S	S	S	S	S	S
CRYPAK318-17	Pakistan	Pakistan	Delhi	R	S	S	S	R	R	S	S
CRYPAK143-17	Pakistan	Pakistan	Delhi	R	R	S	S	R	R	S	S
CRYPAK57-17	Pakistan	Pakistan	EAI	R	R	n/a	S	R	R	R	S
CRYPAK311-17	Pakistan	Pakistan	Delhi	S	S	S	S	S	S	S	S
CRYPAK193-17	Pakistan	Pakistan	Delhi	S	S	S	S	S	S	S	S
CRYPAK228-17	Pakistan	Pakistan	Cameroon	S	S	S	S	S	S	S	S
CRYPAK332-17	Pakistan	Pakistan	Delhi	R	R	R	S	R	R	S	S
CRYPAK458-16	Pakistan	Pakistan	lineage4	R	R	R	R	R	R	U	R
CRYPAK502-16	Pakistan	Pakistan	Delhi	R	R	R	R	U	R	R	F
CRYPAK286-17	Pakistan	Pakistan	Tur	R	R	S	R	R	R	R	F
CRYPAK484-16	Pakistan	Pakistan	Delhi	R	R	R	S	R	R	R	S
CRYPAK54-17	Pakistan	Pakistan	Delhi	R	R	S	R	R	R	R	R
CRYPAK414-16	Pakistan	Pakistan	Delhi	R	R	S	R	R	R	U	R
CRYPAK456-16	Pakistan	Pakistan	Tur	R	R	R	R	R	R	R	R
CRYPAK162-17	Pakistan	Pakistan	Delhi	R	R	S	R	R	R	R	R
CRY345-16	Pakistan	Pakistan	Beijing	R	R	R	R	R	R	R	R
CRYPAK202-17	Pakistan	Pakistan	Delhi	S	S	S	S	S	S	S	S
CRYPAK229-17	Pakistan	Pakistan	Delhi	S	S	S	S	S	S	S	S
CRYPAK145-17	Pakistan	Pakistan	Delhi	R	R	S	S	R	R	U	S
CRYPAK218-17	Pakistan	Pakistan	Delhi	R	R	R	S	R	R	R	U
CRYPAK486-16	Pakistan	Pakistan	Delhi	R	R	S	S	R	R	S	S
CRYPAK226-17	Pakistan	Pakistan	Delhi	S	S	S	S	S	S	S	S
CRYPAK290-17	Pakistan	Pakistan	Delhi	R	R	S	R	R	F	R	F
CRYPAK334-17	Pakistan	Pakistan	Delhi	R	R	S	R	R	R	R	S
CRYPAK482-16	Pakistan	Pakistan	Delhi	R	R	R	S	R	R	R	S
CRYPAK557-16	Pakistan	Pakistan	Delhi	R	S	S	S	R	R	U	S
CRYPAK298-17	Pakistan	Pakistan	Delhi	S	S	S	S	S	S	S	S
CRYPAK426-16	Pakistan	Pakistan	Delhi	R	R	R	R	R	R	U	R
CRYPAK144-17	Pakistan	Pakistan	Delhi	R	R	R	S	R	R	R	S
CRYPAK108-17	Pakistan	Pakistan	EAI	R	R	S	S	R	R	R	R
CRYPAK303-17	Pakistan	Pakistan	Beijing	R	R	S	S	R	R	F	S
CRYPAK151-17	Pakistan	Pakistan	Delhi	R	R	R	R	R	R	R	R
323-16	Pakistan	Pakistan	Delhi	R	R	R	S	R	R	U	S
CRYPAK442-16	Pakistan	Pakistan	Delhi	R	R	R	S	R	R	R	S
CRYPAK149-17	Pakistan	Pakistan	Delhi	R	R	S	S	R	R	F	S
CRYPAK497-16	Pakistan	Pakistan	Delhi	R	R	R	S	R	R	R	U
CRYPAK329-17	Pakistan	Pakistan	Delhi	R	R	S	S	R	R	R	S
CRYPAK392-16	Pakistan	Pakistan	Delhi	R	R	R	R	R	R	S	U
CRYPAK469-16	Pakistan	Pakistan	Delhi	R	R	R	R	U	R	R	R
CRYPAK355-16	Pakistan	Pakistan	Delhi	S	S	S	S	S	S	S	S
CRYPAK272-17	Pakistan	Pakistan	Delhi	S	S	S	S	S	S	U	S
CRYPAK455-16	Pakistan	Pakistan	Delhi	R	R	R	S	R	R	R	F
CRYPAK129-17	Pakistan	Pakistan	Beijing	R	R	R	R	R	R	R	R
CRYPAK472-16	Pakistan	Pakistan	Delhi	R	R	S	n/a	R	R	R	U
CRYPAK59-17	Pakistan	Pakistan	Delhi	R	R	R	S	R	R	R	R
CRYPAK397-16	Pakistan	Pakistan	Delhi	R	R	S	S	R	F	S	S
CRYPAK75-17	Pakistan	Pakistan	Delhi	R	R	R	S	R	R	R	S
CRYPAK559-16	Pakistan	Pakistan	EAI	R	R	S	R	R	R	R	R
CRYPAK321-17	Pakistan	Pakistan	Delhi	R	R	S	n/a	R	R	R	R
CRYPAK351-16	Pakistan	Pakistan	Delhi	R	R	R	S	F	F	F	F
CRYPAK347-16	Pakistan	Pakistan	Delhi	R	R	R	S	R	R	S	S
CRYPAK501-16	Pakistan	Pakistan	Delhi	R	R	S	S	S	R	U	S
CRYPAK297-17	Pakistan	Pakistan	Delhi	R	R	S	S	R	R	U	S
CRYPAK58-17	Pakistan	Pakistan	Delhi	R	R	R	R	R	R	R	R

CRYPAK435-16	Pakistan	Pakistan	Delhi	R	R	S	S	R	R	R	R
CRYPAK253-17	Pakistan	Pakistan	Delhi	R	R	R	R	R	R	R	R
329-16	Pakistan	Pakistan	Delhi	R	R	R	R	R	R	R	U
CRYPAK473-16	Pakistan	Pakistan	Beijing	R	R	R	R	R	R	R	R
CRYPAK258-17	Pakistan	Pakistan	Delhi	R	R	n/a	S	R	R	S	S
CRYPAK123-17	Pakistan	Pakistan	Delhi	R	R	R	S	R	R	R	F
CRYPAK540-16	Pakistan	Pakistan	lineage4	S	R	S	S	S	R	S	S
CRY391-16	Pakistan	Pakistan	Delhi	R	R	R	S	R	R	R	R
CRYPAK174-17	Pakistan	Pakistan	Delhi	R	R	R	S	R	R	R	S
CRYPAK220-17	Pakistan	Pakistan	Delhi	R	R	R	S	R	R	R	U
CRYPAK354-16	Pakistan	Pakistan	Delhi	R	R	R	S	R	R	U	S
CRYPAK477-16	Pakistan	Pakistan	Delhi	R	R	R	S	R	R	R	R
CRYPAK177-17	Pakistan	Pakistan	lineage4	R	R	R	S	R	R	R	S
CRYPAK268-17	Pakistan	Pakistan	Delhi	S	S	S	S	S	S	S	S
CRYPAK215-17	Pakistan	Pakistan	Delhi	S	S	S	n/a	S	S	S	S
CRYPAK36-17	Pakistan	Pakistan	Delhi	R	R	R	S	R	R	R	R
CRYPAK413-16	Pakistan	Pakistan	Delhi	R	R	R	S	R	S	R	S
CRYPAK487-16	Pakistan	Pakistan	Delhi	R	R	R	S	R	R	F	S
CRYPAK115-17	Pakistan	Pakistan	Delhi	R	R	R	S	R	R	R	F
CRYPAK116-17	Pakistan	Pakistan	Delhi	R	R	S	R	R	R	R	R
CRYPAK338-16	Pakistan	Pakistan	EAI	R	R	R	R	R	R	R	R
CRYPAK88-17	Pakistan	Pakistan	Delhi	R	R	R	S	U	R	R	S
CRYPAK179-17	Pakistan	Pakistan	lineage4	R	R	R	R	R	R	R	R
CRYPAK257-17	Pakistan	Pakistan	EAI	R	R	R	R	R	R	R	R
CRYPAK379-16	Pakistan	Pakistan	lineage4	R	R	R	R	R	R	R	R
CRYPAK415-16	Pakistan	Pakistan	Delhi	R	R	R	S	R	R	R	S
CRYPAK72-17	Pakistan	Pakistan	Delhi	R	R	R	S	R	R	R	R
CRYPAK180-17	Pakistan	Pakistan	Delhi	R	S	n/a	S	R	S	S	S
CRYPAK256-17	Pakistan	Pakistan	lineage4	S	S	S	S	S	S	S	S
CRYPAK460-16	Pakistan	Pakistan	Delhi	R	R	R	R	R	F	F	F
CRYPAK428-16	Pakistan	Pakistan	Delhi	R	R	R	S	U	R	R	R
CRYPAK279-17	Pakistan	Pakistan	Delhi	R	S	S	S	U	S	S	S
CRYPAK546-16	Pakistan	Pakistan	Delhi	R	R	R	R	R	R	U	R
CRYPAK553-16	Pakistan	Pakistan	Delhi	R	R	R	R	R	R	R	F
CRYPAK252-17	Pakistan	Pakistan	Delhi	R	R	R	S	R	R	R	S
CRYPAK224-17	Pakistan	Pakistan	lineage4	R	R	R	S	R	R	R	R
CRYPAK307-17	Pakistan	Pakistan	Delhi	R	R	R	S	R	R	R	S
CRYPAK547-16	Pakistan	Pakistan	Delhi	R	R	S	S	U	R	F	S
CRYPAK475-16	Pakistan	Pakistan	lineage4	R	R	R	S	R	R	R	R
CRYPAK287-17	Pakistan	Pakistan	Delhi	S	S	S	S	S	S	S	S
CRYPAK304-17	Pakistan	Pakistan	Cameroon	S	S	S	S	S	S	U	S
CRYPAK217-17	Pakistan	Pakistan	Delhi	R	R	R	S	R	R	R	R
CRYPAK74-17	Pakistan	Pakistan	Delhi	R	R	S	R	R	R	R	R
CRYPAK98-17	Pakistan	Pakistan	Delhi	R	R	R	S	R	R	S	S
CRYPAK235-17	Pakistan	Pakistan	Beijing	R	R	R	R	R	R	R	R
CRYPAK125-17	Pakistan	Pakistan	Cameroon	R	R	R	R	S	R	S	F
CRYPAK276-17	Pakistan	Pakistan	Beijing	R	R	R	S	R	R	R	S
CRYPAK118-17	Pakistan	Pakistan	Tur	R	R	R	R	R	R	R	U
CRYPAK545-16	Pakistan	Pakistan	Delhi	R	R	S	S	S	R	R	S
CRYPAK62-17	Pakistan	Pakistan	Delhi	R	R	S	R	R	R	R	F
CRYPAK424-16	Pakistan	Pakistan	Delhi	R	R	R	S	R	R	R	R
CRYPAK496-16	Pakistan	Pakistan	Delhi	R	R	R	R	U	R	R	R
CRYPAK213-17	Pakistan	Pakistan	Delhi	R	R	R	R	R	R	U	R
334-16	Pakistan	Pakistan	lineage4	R	R	R	R	R	R	R	R
CRYPAK300-17	Pakistan	Pakistan	Delhi	R	R	S	S	R	R	S	S
CRYPAK136-17	Pakistan	Pakistan	Delhi	R	R	R	S	R	R	R	S
CRYPAK294-17	Pakistan	Pakistan	lineage4	R	R	S	S	R	R	R	S
CRYPAK508-16	Pakistan	Pakistan	Delhi	R	R	R	R	R	R	R	R
CRYPAK381-16	Pakistan	Pakistan	Beijing	R	R	R	S	R	R	R	S
CRYPAK122-17	Pakistan	Pakistan	Delhi	R	R	R	S	R	R	R	S
CRYPAK274-17	Pakistan	Pakistan	Delhi	R	R	S	S	R	R	R	S
CRYPAK292-17	Pakistan	Pakistan	Delhi	R	R	S	S	R	R	S	S
CRYPAK446-16	Pakistan	Pakistan	Delhi	R	R	R	S	R	R	R	S
CRYPAK289-17	Pakistan	Pakistan	Delhi	S	S	S	S	S	S	S	S
CRYPAK316-17	Pakistan	Pakistan	Delhi	S	S	S	S	S	S	S	S
CRYPAK188-17	Pakistan	Pakistan	Delhi	S	S	S	S	S	S	S	S
CRYPAK167-17	Pakistan	Pakistan	Delhi	R	R	R	S	R	R	R	S
CRYPAK306-17	Pakistan	Pakistan	Delhi	R	R	S	S	S	R	S	S
CRYPAK97-17	Pakistan	Pakistan	Delhi	R	R	S	S	R	R	R	S
CRYPAK432-16	Pakistan	Pakistan	Delhi	R	R	S	R	R	R	U	R
337-16	Pakistan	Pakistan	Delhi	R	R	S	S	R	R	S	S
CRYPAK386-16	Pakistan	Pakistan	Delhi	R	R	S	S	R	R	R	S
CRYPAK467-16	Pakistan	Pakistan	Haarlem	R	R	n/a	S	R	R	R	S
CRYPAK453-16	Pakistan	Pakistan	Delhi	R	R	S	S	R	R	S	S
CRYPAK103-17	Pakistan	Pakistan	Delhi	R	R	R	S	R	R	R	S
CRYPAK341-16	Pakistan	Pakistan	Delhi	R	R	S	R	R	R	S	R
CRYPAK441-16	Pakistan	Pakistan	Delhi	R	R	R	S	R	R	U	S
CRYPAK53-17	Pakistan	Pakistan	Delhi	R	R	n/a	R	R	R	R	U
CRYPAK251-17	Pakistan	Pakistan	Delhi	S	S	S	S	S	S	S	S
CRYPAK335-17	Pakistan	Pakistan	Delhi	S	S	S	S	R	S	S	S
CRYPAK248-17	Pakistan	Pakistan	Delhi	R	R	R	S	R	R	R	S
CRYPAK507-16	Pakistan	Pakistan	Delhi	R	R	R	S	R	R	F	S
CRYPAK140-17	Pakistan	Pakistan	Delhi	R	R	R	S	R	R	R	S
CRYPAK275-17	Pakistan	Pakistan	Delhi	S	S	S	S	S	S	S	S
CRYPAK259-17	Pakistan	Pakistan	lineage4	R	R	R	S	R	R	S	S
CRYPAK340-16	Pakistan	Pakistan	Delhi	R	R	S	S	R	R	U	S
328-16	Pakistan	Pakistan	Delhi	R	R	R	S	R	R	R	R
CRYPAK176-17	Pakistan	Pakistan	lineage4	R	R	R	R	R	R	R	R
CRYPAK269-17	Pakistan	Pakistan	Delhi	R	R	S	S	R	R	U	S
CRYPAK427-16	Pakistan	Pakistan	Delhi	R	R	S	R	R	R	R	R
CRYPAK260-17	Pakistan	Pakistan	Delhi	R	R	R	S	R	R	R	U
CRYPAK333-17	Pakistan	Pakistan	Delhi	R	R	R	S	R	R	R	S
CRYPAK223-17	Pakistan	Pakistan	Delhi	R	R	S	S	R	F	U	S
CRYPAK114-17	Pakistan	Pakistan	Delhi	R	R	R	R	R	R	R	R
CRYPAK100-17	Pakistan	Pakistan	Delhi	R	R	R	R	R	R	R	R
314-16	Pakistan	Pakistan	Delhi	R	R	R	R	U	R	F	R
CRYPAK90-17	Pakistan	Pakistan	Cameroon	R	R	S	S	R	R	R	S
324-16	Pakistan	Pakistan	Delhi	R	R	R	S	R	S	R	S
CRYPAK495-16	Pakistan	Pakistan	Delhi	R	R	R	S	R	R	R	S
CRYPAK459-16	Pakistan	Pakistan	Delhi	R	R	S	R	R	R	R	R
326-16	Pakistan	Pakistan	Beijing	R	R	R	R	R	R	R	R
CRYPAK205-17	Pakistan	Pakistan	Delhi	R	S	S	S	R	S	S	S
CRYPAK283-17	Pakistan	Pakistan	Delhi	S	S	S	S	S	S	S	S
CRYPAK312-17	Pakistan	Pakistan	Delhi	R	R	R	R	R	R	R	R
CRYPAK68-17	Pakistan	Pakistan	Delhi	R	R	R	S	R	R	R	F
CRYPAK84-17	Pakistan	Pakistan	Delhi	R	R	S	S	U	R	R	S
CRYPAK401-16	Pakistan	Pakistan	Haarlem	R	R	R	R	R	R	R	R
CRYPAK344-16	Pakistan	Pakistan	Beijing	R	R	R	S	R	R	R	S
CRYPAK119-17	Pakistan	Pakistan	Delhi	R	R	R	R	R	R	R	U
CRYPAK285-17	Pakistan	Pakistan	Delhi	R	R	R	R	R	R	U	F
CRYPAK476-16	Pakistan	Pakistan	Beijing	R	R	R	R	R	R	R	R
CRYPAK449-16	Pakistan	Pakistan	Delhi	R	R	n/a	R	R	R	F	F
CRYPAK101-17	Pakistan	Pakistan	Delhi	R	R	R	S	R	R	R	U
CRYPAK271-17	Pakistan	Pakistan	Delhi	R	R	S	S	R	R	S	S
CRYPAK92-17	Pakistan	Pakistan	Delhi	R	R	R	R	R	R	R	R
CRYPAK238-17	Pakistan	Pakistan	Delhi	R	R	S	n/a	R	R	F	S

CRYPAK163-17	Pakistan	Pakistan	Delhi	R	R	R	S	R	R	R	S
CRYPAK341-17	Pakistan	Pakistan	Haarlem	R	R	R	n/a	R	R	R	S
03-R1504	Harvard	Peru	Beijing	R	R	R	R	R	R	R	R
01-R1387	Harvard	Peru	LAM	R	R	R	R	R	R	R	R
01-R0185	Harvard	Peru	Haarlem	R	R	R	R	R	R	U	U
02-R1457	Harvard	Peru	LAM	R	R	R	R	R	R	U	R
02-R1681	Harvard	Peru	LAM	R	R	R	R	R	R	R	R
01-R1466	Harvard	Peru	LAM	R	R	R	S	R	R	R	S
01-R0451	Harvard	Peru	lineage4	R	R	R	R	R	R	R	R
01-R0774	Harvard	Peru	Beijing	R	R	R	R	R	R	R	F
02-R1101	Harvard	Peru	Haarlem	S	S	S	S	S	F	S	F
01-R0909	Harvard	Peru	lineage4	R	R	R	R	R	R	R	R
01-R0420	Harvard	Peru	LAM	R	R	R	R	R	R	F	S
01-R1321	Harvard	Peru	Beijing	R	R	R	R	R	R	R	F
01-R1505	Harvard	Peru	Beijing	R	R	R	R	R	R	R	R
02-R1275	Harvard	Peru	lineage4	S	S	S	S	S	S	S	S
02-R0325	Harvard	Peru	LAM	R	R	R	S	R	R	R	R
01-R1339	Harvard	Peru	lineage4	R	R	S	R	R	R	R	R
03-R0797	Harvard	Peru	Haarlem	R	R	R	R	R	R	R	R
02-R1076	Harvard	Peru	Haarlem	S	S	S	S	S	S	S	S
01-R0878	Harvard	Peru	lineage4	R	R	R	R	R	R	R	R
02-R1140	Harvard	Peru	lineage4	S	S	S	S	S	S	S	S
02-R1645	Harvard	Peru	Haarlem	R	R	S	S	S	R	S	S
03-R0951	Harvard	Peru	Haarlem	R	R	R	R	U	R	R	R
02-R1683	Harvard	Peru	LAM	R	R	R	S	R	R	R	S
00-R1562	Harvard	Peru	Beijing	R	R	R	S	R	R	S	U
03-10017	Harvard	Peru	Beijing	S	S	S	S	S	S	S	S
03-R0795	Harvard	Peru	LAM	S	S	S	S	S	S	S	S
02-R1726	Harvard	Peru	Haarlem	R	R	R	S	R	R	U	U
02-R1915	Harvard	Peru	LAM	R	S	S	S	S	S	S	S
03-R0061	Harvard	Peru	LAM	R	R	R	R	R	R	R	R
02-09688	Harvard	Peru	EAI	S	S	S	n/a	S	S	U	S
03-R1082	Harvard	Peru	LAM	R	R	R	R	R	R	F	R
00-R1547	Harvard	Peru	lineage4	R	R	R	R	R	R	R	R
99-R862	Harvard	Peru	LAM	R	R	R	S	R	R	R	R
02-R1485	Harvard	Peru	LAM	R	R	R	R	R	R	R	R
00-R0308	Harvard	Peru	LAM	R	R	R	R	R	R	R	R
01-R0265	Harvard	Peru	Haarlem	R	R	S	R	R	R	U	R
03-R0986	Harvard	Peru	LAM	R	R	S	R	R	R	S	R
01-R1386	Harvard	Peru	Haarlem	R	R	S	S	S	R	S	S
02-R1262	Harvard	Peru	LAM	S	S	S	n/a	S	S	S	S
01-R0272	Harvard	Peru	lineage4	R	S	S	R	R	R	R	R
03-R0293	Harvard	Peru	Beijing	R	R	R	S	R	R	R	S
98-R660	Harvard	Peru	LAM	R	R	R	R	R	R	R	R
03-R0655	Harvard	Peru	Haarlem	R	R	R	S	R	R	U	S
03-R0419	Harvard	Peru	LAM	R	R	R	R	R	R	R	S
03-R0929	Harvard	Peru	LAM	S	S	S	S	S	S	S	S
98-R790	Harvard	Peru	Beijing	R	R	R	R	R	R	S	R
99-R1043	Harvard	Peru	lineage4	R	n/a	R	n/a	R	S	R	S
02-R0951	Harvard	Peru	lineage4	R	R	R	R	R	F	F	F
02-R0236	Harvard	Peru	Haarlem	R	R	S	R	R	R	R	U
03-R0749	Harvard	Peru	Haarlem	S	S	S	S	S	S	S	S
01-R0238	Harvard	Peru	LAM	R	R	S	S	R	R	S	S
01-R1468	Harvard	Peru	LAM	R	R	R	R	R	R	R	R
03-R0194	Harvard	Peru	LAM	R	R	R	R	R	R	R	R
03-R0070	Harvard	Peru	LAM	R	R	R	R	R	R	R	R
03-R0177	Harvard	Peru	LAM	R	R	R	R	R	R	R	R
02-R1945	Harvard	Peru	LAM	R	R	R	R	R	R	R	R
02-R0984	Harvard	Peru	LAM	R	R	S	R	R	R	R	R
99-R545	Harvard	Peru	lineage4	R	R	R	R	R	R	R	R
01-R0880	Harvard	Peru	LAM	R	R	R	R	R	F	R	S
03-R0327	Harvard	Peru	Beijing	R	R	R	R	R	R	S	R
02-R0237	Harvard	Peru	lineage4	R	R	R	n/a	R	R	R	F
01-R1305	Harvard	Peru	lineage4	R	R	R	R	R	R	R	R
04-R0438	Harvard	Peru	LAM	R	R	R	R	R	R	U	R
02-R1191	Harvard	Peru	Haarlem	S	S	S	S	S	S	S	S
03-R0319	Harvard	Peru	LAM	R	R	R	R	R	R	R	R
01-R0899	Harvard	Peru	lineage4	R	R	R	R	R	R	R	R
02-R1288	Harvard	Peru	Beijing	R	R	R	R	R	R	R	F
02-09397	Harvard	Peru	Haarlem	S	S	S	n/a	S	S	S	S
03-R1176	Harvard	Peru	Beijing	R	R	R	R	R	R	R	R
02-R0759	Harvard	Peru	Haarlem	R	R	R	R	R	R	R	R
03-R0768	Harvard	Peru	X-type	S	S	S	S	U	S	S	S
02-R1589	Harvard	Peru	lineage4	R	R	S	R	R	R	S	R
02-R1203	Harvard	Peru	lineage4	S	S	S	S	S	S	S	S
03-R0780	Harvard	Peru	LAM	S	S	S	S	S	S	S	S
02-R1630	Harvard	Peru	lineage4	R	R	R	R	R	R	R	R
01-R0153	Harvard	Peru	X-type	R	R	R	R	R	R	R	R
03-R1451	Harvard	Peru	Haarlem	R	R	R	S	R	R	R	U
99-10364	Harvard	Peru	LAM	R	R	R	R	R	R	R	R
02-R0911	Harvard	Peru	LAM	R	R	R	R	R	R	U	R
03-R0411	Harvard	Peru	LAM	R	S	R	S	U	S	U	S
02-09856	Harvard	Peru	lineage4	S	S	S	n/a	S	S	S	S
03-R1465	Harvard	Peru	Haarlem	R	R	R	R	R	R	R	R
02-R1940	Harvard	Peru	lineage4	R	R	R	R	R	R	R	R
03-R0878	Harvard	Peru	Beijing	R	R	R	R	R	R	R	F
01-R0685	Harvard	Peru	lineage4	R	R	R	R	R	R	R	R
02-R1544	Harvard	Peru	LAM	R	S	S	R	R	R	S	R
02-R0754	Harvard	Peru	LAM	R	R	S	R	R	R	S	R
02-R0948	Harvard	Peru	lineage4	R	R	R	R	R	R	R	R
99-R576	Harvard	Peru	X-type	R	R	R	R	R	R	R	R
03-R0871	Harvard	Peru	LAM	R	R	R	R	R	R	R	R
03-R0268	Harvard	Peru	Beijing	R	R	R	R	R	R	R	R
01-R0908	Harvard	Peru	lineage4	R	R	R	R	R	R	R	U
02-R0099	Harvard	Peru	lineage4	R	R	R	S	R	R	R	S
02-R0119	Harvard	Peru	LAM	R	R	S	S	R	R	R	R
01-R1599	Harvard	Peru	LAM	R	R	S	R	R	R	R	R
01-R1309	Harvard	Peru	lineage4	R	R	R	R	R	R	R	F
02-R1685	Harvard	Peru	LAM	R	R	R	R	R	R	R	R
98-R454	Harvard	Peru	Haarlem	R	R	R	n/a	R	R	R	R
01-R0166	Harvard	Peru	LAM	R	R	R	R	R	R	R	R
02-R0848	Harvard	Peru	S-type	R	R	S	R	R	R	U	R
03-R0783	Harvard	Peru	lineage4	S	S	S	S	S	S	S	S
03-R0920	Harvard	Peru	Haarlem	R	S	S	S	R	S	S	S
02-R1687	Harvard	Peru	LAM	R	R	R	R	R	R	R	R
01-R1018	Harvard	Peru	LAM	R	R	R	R	R	R	R	F
00-R1566	Harvard	Peru	X-type	R	R	R	R	R	R	R	R
00-R1399	Harvard	Peru	lineage4	R	R	R	n/a	R	R	R	R
04-R0292	Harvard	Peru	Beijing	R	R	R	R	R	R	R	R
02-R1114	Harvard	Peru	LAM	S	S	S	S	U	S	S	S
02-10075	Harvard	Peru	Haarlem	S	S	S	n/a	S	S	S	S
02-R1244	Harvard	Peru	X-type	S	S	S	S	S	S	S	S
03-R0068	Harvard	Peru	LAM	R	R	S	S	R	R	U	S
99-R719	Harvard	Peru	LAM	R	R	R	S	R	R	S	S
01-R1499	Harvard	Peru	lineage4	R	R	R	n/a	R	R	R	R
00-R0223	Harvard	Peru	lineage4	S	S	S	n/a	S	S	S	S

02-R0272	Harvard	Peru	LAM	R	R	R	S	R	R	R	R
03-R0110	Harvard	Peru	Haarlem	R	R	R	R	R	R	U	U
02-R0971	Harvard	Peru	lineage4	R	R	R	S	U	R	R	R
00-R1405	Harvard	Peru	lineage4	R	R	R	R	R	R	R	U
02-09928	Harvard	Peru	LAM	S	S	S	n/a	S	S	U	S
02-R0286	Harvard	Peru	LAM	R	R	S	S	R	R	R	S
02-R0890	Harvard	Peru	X-type	R	S	S	S	R	R	R	R
01-R0239	Harvard	Peru	LAM	R	R	R	S	R	R	R	R
01-R0904	Harvard	Peru	LAM	R	R	R	R	R	R	R	R
02-R1142	Harvard	Peru	LAM	S	S	S	S	S	S	S	S
02-R0407	Harvard	Peru	Haarlem	S	S	S	S	S	S	S	S
99-R1083	Harvard	Peru	LAM	R	R	R	S	R	R	R	R
03-R0979	Harvard	Peru	LAM	R	R	R	S	R	R	R	U
02-R0360	Harvard	Peru	Haarlem	R	R	R	S	R	R	R	S
01-R0244	Harvard	Peru	Haarlem	R	R	S	R	R	R	R	U
03-R0821	Harvard	Peru	LAM	R	R	R	R	R	R	R	R
03-R0908	Harvard	Peru	LAM	S	S	S	S	S	S	S	S
02-R1871	Harvard	Peru	Haarlem	R	R	R	S	R	R	R	S
02-R1793	Harvard	Peru	Beijing	R	R	R	R	R	R	R	U
02-R1479	Harvard	Peru	X-type	R	R	R	R	R	R	R	R
03-R0221	Harvard	Peru	LAM	R	R	R	R	R	R	R	R
02-R1709	Harvard	Peru	LAM	R	R	R	R	R	R	R	U
01-R0897	Harvard	Peru	lineage4	R	R	R	S	R	R	R	S
00-R0435	Harvard	Peru	lineage4	R	R	R	R	R	R	R	U
02-10028	Harvard	Peru	EAI	S	S	S	n/a	S	S	S	F
03-R0324	Harvard	Peru	LAM	R	R	R	R	R	R	R	R
02-R1106	Harvard	Peru	LAM	S	S	S	S	S	S	S	S
03-R0736	Harvard	Peru	Haarlem	S	S	S	S	S	S	S	S
03-R0058	Harvard	Peru	Beijing	R	S	R	R	R	R	R	R
01-R0697	Harvard	Peru	lineage4	R	R	R	R	R	R	R	R
01-R0903	Harvard	Peru	LAM	R	R	R	R	R	R	R	R
01-R0737	Harvard	Peru	LAM	R	R	R	R	R	R	S	R
02-R1789	Harvard	Peru	Beijing	R	R	R	R	R	R	R	F
00-R0312	Harvard	Peru	LAM	R	R	R	n/a	R	R	R	R
02-R0417	Harvard	Peru	lineage4	R	R	R	R	R	R	R	R
00-R0025	Harvard	Peru	Beijing	R	R	R	n/a	R	R	R	U
02-R1723	Harvard	Peru	Haarlem	R	R	R	R	R	R	R	R
02-R0241	Harvard	Peru	LAM	R	R	R	S	R	R	R	U
99-R890	Harvard	Peru	X-type	R	R	R	n/a	R	R	R	R
00-R1156	Harvard	Peru	X-type	R	R	R	R	R	R	R	R
02-R1742	Harvard	Peru	Beijing	R	R	R	R	R	R	R	S
02-R1952	Harvard	Peru	Beijing	R	R	R	R	R	R	R	F
03-R1338	Harvard	Peru	lineage4	R	R	R	R	R	R	R	R
03-R1337	Harvard	Peru	LAM	R	R	R	R	R	R	R	R
01-R1559	Harvard	Peru	Beijing	R	R	R	S	R	R	R	S
02-R0191	Harvard	Peru	Haarlem	R	R	R	R	R	R	R	U
00-R0086	Harvard	Peru	Beijing	R	R	R	R	R	R	R	R
02-10098	Harvard	Peru	X-type	S	S	S	n/a	S	S	S	S
02-R1942	Harvard	Peru	lineage4	R	R	R	R	R	R	R	R
02-R1854	Harvard	Peru	LAM	S	S	S	S	S	S	U	S
02-R1728	Harvard	Peru	LAM	R	R	R	R	R	R	R	R
02-R1137	Harvard	Peru	LAM	S	S	S	S	S	S	S	S
02-R1641	Harvard	Peru	Beijing	R	R	R	S	R	R	R	S
04-R0273	Harvard	Peru	lineage4	R	R	R	R	R	R	R	F
01-R0276	Harvard	Peru	LAM	R	R	S	R	R	R	R	F
02-R1260	Harvard	Peru	Delhi	S	S	S	n/a	S	S	S	S
00-R0453	Harvard	Peru	LAM	R	R	R	n/a	R	R	R	R
02-R1210	Harvard	Peru	Haarlem	S	S	S	S	S	S	S	S
02-R0793	Harvard	Peru	lineage4	R	R	R	R	R	R	R	R
99-R887	Harvard	Peru	LAM	R	R	R	R	R	R	R	R
01-R0647	Harvard	Peru	Beijing	R	R	S	R	R	R	R	R
01-R1241	Harvard	Peru	Haarlem	R	R	R	S	R	R	U	U
02-R0861	Harvard	Peru	LAM	R	R	R	R	R	R	R	R
02-R1267	Harvard	Peru	EAI	S	S	S	n/a	S	S	S	S
02-R1753	Harvard	Peru	Beijing	R	S	R	S	R	R	U	S
02-R0812	Harvard	Peru	LAM	R	R	R	R	R	R	R	R
00-R0178	Harvard	Peru	LAM	R	R	R	R	R	R	R	R
00-R1549	Harvard	Peru	lineage4	R	R	R	S	R	R	R	U
03-10150	Harvard	Peru	LAM	S	S	S	n/a	S	S	S	S
02-R1543	Harvard	Peru	S-type	R	R	R	S	R	R	R	S
01-R1341	Harvard	Peru	X-type	R	R	R	R	R	R	R	R
02-R0016	Harvard	Peru	LAM	R	R	R	R	R	R	R	F
99-R141	Harvard	Peru	lineage4	R	R	R	n/a	R	R	R	R
02-R1825	Harvard	Peru	Beijing	R	R	R	R	R	R	R	R
01-R1540	Harvard	Peru	Beijing	R	R	S	R	R	R	S	R
02-R0328	Harvard	Peru	Beijing	R	R	R	R	R	R	R	S
03-R1404	Harvard	Peru	Beijing	R	R	R	R	R	R	U	R
02-R1527	Harvard	Peru	lineage4	R	R	R	R	R	R	R	R
99-R855	Harvard	Peru	Beijing	R	R	R	R	R	R	R	F
03-R1373	Harvard	Peru	X-type	R	R	R	R	R	R	R	R
02-R1444	Harvard	Peru	LAM	R	R	S	R	R	R	R	R
01-R0902	Harvard	Peru	LAM	R	R	R	R	R	R	R	R
03-R1084	Harvard	Peru	Beijing	R	S	R	R	R	R	R	R
01-R0290	Harvard	Peru	LAM	R	R	R	R	R	R	R	U
03-10019	Harvard	Peru	Beijing	S	S	S	S	S	S	S	S
02-R1941	Harvard	Peru	Haarlem	R	R	R	R	R	R	R	R
14893_2_49	Peru via LSHTM, UK	Peru	Haarlem	S	R	n/a	n/a	S	R	S	S
14722_6_94	Peru via LSHTM, UK	Peru	Haarlem	R	R	n/a	n/a	R	R	R	R
14892_2_31	Peru via LSHTM, UK	Peru	LAM	R	S	n/a	n/a	R	S	S	S
14893_2_1	Peru via LSHTM, UK	Peru	LAM	R	S	n/a	n/a	R	S	S	S
14892_2_34	Peru via LSHTM, UK	Peru	LAM	R	R	n/a	n/a	R	R	S	S
14892_2_41	Peru via LSHTM, UK	Peru	Haarlem	R	R	n/a	n/a	R	R	U	R
14892_2_57	Peru via LSHTM, UK	Peru	Haarlem	R	S	n/a	n/a	R	S	S	S
14893_2_43	Peru via LSHTM, UK	Peru	Haarlem	R	R	n/a	n/a	R	R	R	R
14893_2_34	Peru via LSHTM, UK	Peru	LAM	R	R	n/a	n/a	R	R	R	S
15277_3_50	Peru via LSHTM, UK	Peru	lineage4	S	R	n/a	n/a	S	R	R	S
14722_6_33	Peru via LSHTM, UK	Peru	LAM	R	R	n/a	n/a	R	R	R	S
14892_2_33	Peru via LSHTM, UK	Peru	LAM	R	S	n/a	n/a	R	S	S	S
14893_2_61	Peru via LSHTM, UK	Peru	LAM	R	R	n/a	n/a	R	R	S	S
14893_2_45	Peru via LSHTM, UK	Peru	LAM	S	R	n/a	n/a	R	R	S	R
14892_2_10	Peru via LSHTM, UK	Peru	Haarlem	R	S	n/a	n/a	R	S	S	S
14722_6_26	Peru via LSHTM, UK	Peru	LAM	S	S	n/a	n/a	S	S	U	S
14893_2_20	Peru via LSHTM, UK	Peru	X-type	R	S	n/a	n/a	R	S	S	S
14893_2_47	Peru via LSHTM, UK	Peru	Haarlem	S	R	n/a	n/a	S	R	S	S
14892_2_5	Peru via LSHTM, UK	Peru	Beijing	R	R	n/a	n/a	R	R	S	S
14892_2_22	Peru via LSHTM, UK	Peru	X-type	R	S	n/a	n/a	R	S	S	S
14893_2_9	Peru via LSHTM, UK	Peru	lineage4	R	R	n/a	n/a	R	R	R	S
14892_2_30	Peru via LSHTM, UK	Peru	Haarlem	R	S	n/a	n/a	R	S	S	S
14893_2_18	Peru via LSHTM, UK	Peru	LAM	S	R	n/a	n/a	F	F	S	F
14893_2_5	Peru via LSHTM, UK	Peru	EAI	S	S	n/a	n/a	S	S	S	S
14893_2_65	Peru via LSHTM, UK	Peru	LAM	R	S	n/a	n/a	R	S	S	S
14893_2_36	Peru via LSHTM, UK	Peru	LAM	R	R	n/a	n/a	R	R	R	R
14892_2_9	Peru via LSHTM, UK	Peru	LAM	R	R	n/a	n/a	R	R	S	S
14892_2_18	Peru via LSHTM, UK	Peru	Haarlem	R	S	n/a	n/a	R	S	S	S
14892_2_36	Peru via LSHTM, UK	Peru	Haarlem	R	S	n/a	n/a	R	U	S	S
14722_6_69	Peru via LSHTM, UK	Peru	LAM	S	S	n/a	n/a	S	S	S	S

14892_2_44	Peru via LSHTM, UK	Peru	X-type		S	S	n/a	n/a	S	S	S	S
14893_2_55	Peru via LSHTM, UK	Peru	Beijing		R	R	n/a	n/a	R	R	S	S
14893_2_63	Peru via LSHTM, UK	Peru	Haarlem		R	R	n/a	n/a	R	R	U	S
14893_2_42	Peru via LSHTM, UK	Peru	Haarlem		R	S	n/a	n/a	R	S	S	S
14722_7_80	Peru via LSHTM, UK	Peru	LAM		R	S	n/a	n/a	R	S	S	S
14722_7_29	Peru via LSHTM, UK	Peru	Haarlem		R	S	n/a	n/a	R	S	R	S
14893_2_14	Peru via LSHTM, UK	Peru	Haarlem		R	R	n/a	n/a	S	S	S	S
14893_2_8	Peru via LSHTM, UK	Peru	LAM		R	R	n/a	n/a	R	R	S	R
14722_6_84	Peru via LSHTM, UK	Peru	lineage4		R	S	n/a	n/a	R	S	S	S
14893_2_10	Peru via LSHTM, UK	Peru	LAM		R	R	n/a	n/a	R	R	U	R
14893_2_21	Peru via LSHTM, UK	Peru	lineage4		R	R	n/a	n/a	R	R	R	S
14722_6_13	Peru via LSHTM, UK	Peru	LAM		S	R	n/a	n/a	R	R	R	R
14893_2_50	Peru via LSHTM, UK	Peru	LAM		R	R	n/a	n/a	R	R	R	R
15277_3_52	Peru via LSHTM, UK	Peru	lineage4		R	R	n/a	n/a	R	R	R	R
14893_2_23	Peru via LSHTM, UK	Peru	LAM		S	R	n/a	n/a	R	R	S	R
14722_6_21	Peru via LSHTM, UK	Peru	Haarlem		R	S	n/a	n/a	S	U	S	S
14892_2_1	Peru via LSHTM, UK	Peru	LAM		R	R	n/a	n/a	R	R	R	R
14722_7_77	Peru via LSHTM, UK	Peru	lineage4		R	R	n/a	n/a	R	R	R	R
14893_2_57	Peru via LSHTM, UK	Peru	Haarlem		S	S	n/a	n/a	S	S	S	S
14722_6_35	Peru via LSHTM, UK	Peru	LAM		R	S	n/a	n/a	R	S	S	S
14893_2_60	Peru via LSHTM, UK	Peru	Haarlem		S	S	n/a	n/a	S	S	S	S
14722_7_70	Peru via LSHTM, UK	Peru	lineage4		R	S	n/a	n/a	R	S	S	S
14892_2_60	Peru via LSHTM, UK	Peru	LAM		R	S	n/a	n/a	R	S	S	S
14722_7_90	Peru via LSHTM, UK	Peru	lineage4		R	R	n/a	n/a	R	R	R	S
14722_6_59	Peru via LSHTM, UK	Peru	lineage4		S	R	n/a	n/a	R	R	R	S
14722_6_48	Peru via LSHTM, UK	Peru	M. caprae		S	S	n/a	n/a	S	U	S	S
14892_2_59	Peru via LSHTM, UK	Peru	lineage4		S	R	n/a	n/a	R	R	R	S
14722_7_62	Peru via LSHTM, UK	Peru	Beijing		R	R	n/a	n/a	R	R	U	S
14722_6_49	Peru via LSHTM, UK	Peru	S-type		S	S	n/a	n/a	S	S	S	S
14893_2_62	Peru via LSHTM, UK	Peru	LAM		R	R	n/a	n/a	R	R	S	S
14892_2_50	Peru via LSHTM, UK	Peru	LAM		R	R	n/a	n/a	R	R	R	S
14722_6_10	Peru via LSHTM, UK	Peru	LAM		R	R	n/a	n/a	R	S	U	S
14893_2_54	Peru via LSHTM, UK	Peru	Beijing		R	R	n/a	n/a	R	R	S	R
14722_7_74	Peru via LSHTM, UK	Peru	Haarlem		R	R	n/a	n/a	R	R	S	S
14892_2_42	Peru via LSHTM, UK	Peru	LAM		R	S	n/a	n/a	R	S	S	S
14892_2_23	Peru via LSHTM, UK	Peru	LAM		R	S	n/a	n/a	R	S	S	S
14892_2_58	Peru via LSHTM, UK	Peru	LAM		R	R	n/a	n/a	R	R	R	U
14893_2_48	Peru via LSHTM, UK	Peru	LAM		S	R	n/a	n/a	R	R	R	S
14893_2_59	Peru via LSHTM, UK	Peru	LAM		R	R	n/a	n/a	R	R	R	R
15277_3_57	Peru via LSHTM, UK	Peru	LAM		S	R	n/a	n/a	R	R	S	R
14892_2_7	Peru via LSHTM, UK	Peru	Haarlem		R	S	n/a	n/a	R	S	S	S
14892_2_12	Peru via LSHTM, UK	Peru	Beijing		R	R	n/a	n/a	R	R	R	R
14722_6_19	Peru via LSHTM, UK	Peru	LAM		R	S	n/a	n/a	R	S	S	S
14893_2_4	Peru via LSHTM, UK	Peru	LAM		S	R	n/a	n/a	S	R	U	S
15277_3_53	Peru via LSHTM, UK	Peru	LAM		R	R	n/a	n/a	R	R	R	R
14722_6_64	Peru via LSHTM, UK	Peru	lineage4		R	S	n/a	n/a	R	R	R	S
14722_6_18	Peru via LSHTM, UK	Peru	LAM		R	S	n/a	n/a	R	S	S	S
14892_2_61	Peru via LSHTM, UK	Peru	lineage4		R	S	n/a	n/a	F	S	R	S
14722_6_22	Peru via LSHTM, UK	Peru	M. caprae		S	S	n/a	n/a	U	U	S	S
14893_2_53	Peru via LSHTM, UK	Peru	LAM		R	S	n/a	n/a	R	S	S	S
14893_2_33	Peru via LSHTM, UK	Peru	Haarlem		R	S	n/a	n/a	R	S	S	S
14722_7_88	Peru via LSHTM, UK	Peru	Haarlem		R	S	n/a	n/a	R	S	S	S
14722_7_64	Peru via LSHTM, UK	Peru	LAM		R	S	n/a	n/a	R	S	S	S
14722_6_40	Peru via LSHTM, UK	Peru	LAM		R	R	n/a	n/a	R	R	R	U
14722_6_86	Peru via LSHTM, UK	Peru	LAM		S	R	n/a	n/a	R	R	S	R
14722_7_61	Peru via LSHTM, UK	Peru	X-type		R	S	n/a	n/a	R	S	S	S
14893_2_12	Peru via LSHTM, UK	Peru	Haarlem		S	R	n/a	n/a	S	R	S	S
14892_2_43	Peru via LSHTM, UK	Peru	LAM		R	R	n/a	n/a	R	R	R	R
14722_6_41	Peru via LSHTM, UK	Peru	Beijing		R	S	n/a	n/a	R	R	U	S
14893_2_58	Peru via LSHTM, UK	Peru	LAM		R	R	n/a	n/a	R	R	R	R
14892_2_39	Peru via LSHTM, UK	Peru	LAM		R	R	n/a	n/a	R	R	R	R
14893_2_6	Peru via LSHTM, UK	Peru	Haarlem		R	R	n/a	n/a	R	R	R	U
14722_6_27	Peru via LSHTM, UK	Peru	Beijing		S	S	n/a	n/a	S	S	S	S
14893_2_64	Peru via LSHTM, UK	Peru	Haarlem		R	R	n/a	n/a	S	U	S	S
14893_2_51	Peru via LSHTM, UK	Peru	X-type		R	S	n/a	n/a	R	S	S	S
14892_2_37	Peru via LSHTM, UK	Peru	LAM		R	S	n/a	n/a	R	R	R	R
14892_2_51	Peru via LSHTM, UK	Peru	LAM		R	R	n/a	n/a	R	R	R	R
14893_2_56	Peru via LSHTM, UK	Peru	LAM		R	R	n/a	n/a	R	R	R	U
14722_7_53	Peru via LSHTM, UK	Peru	lineage4		S	R	n/a	n/a	S	R	R	S
14892_2_2	Peru via LSHTM, UK	Peru	Beijing		R	S	n/a	n/a	R	S	S	S
14722_7_81	Peru via LSHTM, UK	Peru	Haarlem		S	R	n/a	n/a	S	R	S	S
14892_2_20	Peru via LSHTM, UK	Peru	lineage4		S	S	n/a	n/a	S	S	S	S
14722_6_66	Peru via LSHTM, UK	Peru	LAM		R	S	n/a	n/a	R	S	S	S
14893_2_52	Peru via LSHTM, UK	Peru	lineage4		S	R	n/a	n/a	R	R	S	S
14893_2_66	Peru via LSHTM, UK	Peru	LAM		R	R	n/a	n/a	R	R	S	R
14722_6_38	Peru via LSHTM, UK	Peru	LAM		S	R	n/a	n/a	R	R	S	R
14893_2_29	Peru via LSHTM, UK	Peru	lineage4		R	R	n/a	n/a	R	R	S	S
14892_2_29	Peru via LSHTM, UK	Peru	LAM		R	R	n/a	n/a	R	R	R	S
14893_2_41	Peru via LSHTM, UK	Peru	Haarlem		R	S	n/a	n/a	R	S	S	S
14722_6_36	Peru via LSHTM, UK	Peru	Beijing		R	S	n/a	n/a	R	R	S	S
14893_2_46	Peru via LSHTM, UK	Peru	LAM		R	R	n/a	n/a	R	R	R	U
14722_6_71	Peru via LSHTM, UK	Peru	lineage4		S	S	n/a	n/a	S	S	S	S
14722_6_70	Peru via LSHTM, UK	Peru	Beijing		R	S	n/a	n/a	R	S	S	S
14893_2_24	Peru via LSHTM, UK	Peru	LAM		R	S	n/a	n/a	R	S	S	S
14722_6_54	Peru via LSHTM, UK	Peru	LAM		R	R	n/a	n/a	R	R	S	R
0528A	Casali et. al. Nat Genet. 2014 Mar;46(3):279-86	Russia	Haarlem	ERR133877	S	S	R	S	S	S	S	S
2414A	Casali et. al. Nat Genet. 2014 Mar;46(3):279-86	Russia	Beijing	ERR229935	R	S	S	S	R	S	S	S
0443H	Casali et. al. Nat Genet. 2014 Mar;46(3):279-86	Russia	Beijing	ERR067627	R	R	S	R	R	R	R	R
0293V	Casali et. al. Nat Genet. 2014 Mar;46(3):279-86	Russia	Beijing	ERR133839	R	R	R	S	R	R	R	U
1292F	Casali et. al. Nat Genet. 2014 Mar;46(3):279-86	Russia	Ural	ERR108472	S	S	S	S	S	S	S	S
0096F	Casali et. al. Nat Genet. 2014 Mar;46(3):279-86	Russia	Beijing	ERR067608	R	R	S	S	R	S	S	S
1825K	Casali et. al. Nat Genet. 2014 Mar;46(3):279-86	Russia	Beijing	ERR144562	R	R	S	S	R	R	R	U
0405R	Casali et. al. Nat Genet. 2014 Mar;46(3):279-86	Russia	Beijing	ERR133860	R	R	S	S	R	R	R	U
0259H	Casali et. al. Nat Genet. 2014 Mar;46(3):279-86	Russia	Beijing	ERR133827	R	R	R	R	R	R	R	U
2626F	Casali et. al. Nat Genet. 2014 Mar;46(3):279-86	Russia	lineage4	ERR234694	S	S	S	S	S	S	S	S
0197Q	Casali et. al. Nat Genet. 2014 Mar;46(3):279-86	Russia	Haarlem	ERR133818	S	S	S	S	S	S	S	S
0820S	Casali et. al. Nat Genet. 2014 Mar;46(3):279-86	Russia	Beijing	ERR133947	S	R	S	S	S	S	S	S
0401M	Casali et. al. Nat Genet. 2014 Mar;46(3):279-86	Russia	Beijing	ERR133859	S	S	S	S	S	S	S	S
0556F	Casali et. al. Nat Genet. 2014 Mar;46(3):279-86	Russia	Beijing	ERR133885	R	R	R	S	R	R	R	U
2180W	Casali et. al. Nat Genet. 2014 Mar;46(3):279-86	Russia	Beijing	ERR228039	R	R	R	R	R	R	R	F
2373F	Casali et. al. Nat Genet. 2014 Mar;46(3):279-86	Russia	Beijing	ERR229965	S	R	R	S	R	R	R	S
2565P	Casali et. al. Nat Genet. 2014 Mar;46(3):279-86	Russia	Ural	ERR234669	R	R	S	R	R	R	R	R
0754W	Casali et. al. Nat Genet. 2014 Mar;46(3):279-86	Russia	Beijing	ERR133924	R	S	S	S	R	U	R	S
0511G	Casali et. al. Nat Genet. 2014 Mar;46(3):279-86	Russia	Beijing	ERR067761	R	R	S	S	R	R	R	R
0589R	Casali et. al. Nat Genet. 2014 Mar;46(3):279-86	Russia	Beijing	ERR133888	R	R	R	S	R	S	S	R
0710Y	Casali et. al. Nat Genet. 2014 Mar;46(3):279-86	Russia	Beijing	ERR117454	R	R	S	R	R	R	R	R
0943B	Casali et. al. Nat Genet. 2014 Mar;46(3):279-86	Russia	Beijing	ERR067643	R	R	R	S	R	R	R	R
2482Z	Casali et. al. Nat Genet. 2014 Mar;46(3):279-86	Russia	LAM	ERR229944	S	S	S	S	U	S	S	S
1366L	Casali et. al. Nat Genet. 2014 Mar;46(3):279-86	Russia	Beijing	ERR137220	R	R	R	S	R	R	R	U
1456J	Casali et. al. Nat Genet. 2014 Mar;46(3):279-86	Russia	Ural	ERR067700	R	R	R	n/a	U	S	S	S
0063V	Casali et. al. Nat Genet. 2014 Mar;46(3):279-86	Russia	Haarlem	ERR133800	R	R	S	S	R	R	R	R
2326E	Casali et. al. Nat Genet. 2014 Mar;46(3):279-86	Russia	Haarlem	ERR228021	S	S	S	R	S	S	S	R
2042W	Casali et. al. Nat Genet. 2014 Mar;46(3):279-86	Russia	Ural	ERR144624	R	R	R	S	R	R	R	S
1900R	Casali et. al. Nat Genet. 2014 Mar;46(3):279-86	Russia	lineage4	ERR144592	S	S	S	S	S	S	S	S
1626T	Casali et. al. Nat Genet. 2014 Mar;46(3):279-86	Russia	Beijing	ERR067687	R	R	R	R	R	R	R	R
1466V	Casali et. al. Nat Genet. 2014 Mar;46(3):279-86	Russia	Haarlem	ERR067703	S	S	S	S	S	S	S	S

2597Z	Casali et. al. Nat Genet. 2014 Mar;46(3):279-86	Russia	Ural	ERR234691	S	S	S	S	S	S	S	S
2344Z	Casali et. al. Nat Genet. 2014 Mar;46(3):279-86	Russia	Beijing	ERR229971	R	R	R	R	R	R	R	R
0427Q	Casali et. al. Nat Genet. 2014 Mar;46(3):279-86	Russia	Beijing	ERR234575	R	S	S	R	R	S	S	F
1084E	Casali et. al. Nat Genet. 2014 Mar;46(3):279-86	Russia	lineage4	ERR133989	S	S	S	S	S	U	S	S
0024C	Casali et. al. Nat Genet. 2014 Mar;46(3):279-86	Russia	lineage4	ERR108463	S	S	S	S	S	S	S	S
0693E	Casali et. al. Nat Genet. 2014 Mar;46(3):279-86	Russia	Beijing	ERR234642	S	S	S	S	S	S	U	S
0539M	Casali et. al. Nat Genet. 2014 Mar;46(3):279-86	Russia	Beijing	ERR234600	R	R	R	R	R	R	R	R
1513W	Casali et. al. Nat Genet. 2014 Mar;46(3):279-86	Russia	Beijing	ERR137240	R	S	R	S	R	S	R	S
0309M	Casali et. al. Nat Genet. 2014 Mar;46(3):279-86	Russia	Haarlem	ERR067603	R	R	R	R	R	R	R	R
0773R	Casali et. al. Nat Genet. 2014 Mar;46(3):279-86	Russia	Beijing	ERR133930	R	R	S	S	R	R	R	U
2467H	Casali et. al. Nat Genet. 2014 Mar;46(3):279-86	Russia	Beijing	ERR229999	R	S	R	S	R	S	R	S
1176E	Casali et. al. Nat Genet. 2014 Mar;46(3):279-86	Russia	Beijing	ERR137195	S	S	S	S	S	S	U	S
0432W	Casali et. al. Nat Genet. 2014 Mar;46(3):279-86	Russia	Beijing	ERR133862	R	S	S	S	R	S	R	S
0416D	Casali et. al. Nat Genet. 2014 Mar;46(3):279-86	Russia	Beijing	ERR067745	R	S	S	S	R	R	R	S
2507B	Casali et. al. Nat Genet. 2014 Mar;46(3):279-86	Russia	Haarlem	ERR229938	S	S	S	R	S	S	S	S
0132V	Casali et. al. Nat Genet. 2014 Mar;46(3):279-86	Russia	Ural	ERR133809	R	S	S	S	R	S	S	S
0995H	Casali et. al. Nat Genet. 2014 Mar;46(3):279-86	Russia	Beijing	ERR133979	S	S	S	S	S	S	S	S
0395F	Casali et. al. Nat Genet. 2014 Mar;46(3):279-86	Russia	Beijing	ERR133855	R	R	S	S	R	R	R	S
1673V	Casali et. al. Nat Genet. 2014 Mar;46(3):279-86	Russia	Ural	ERR108509	S	S	S	S	S	S	S	S
1605W	Casali et. al. Nat Genet. 2014 Mar;46(3):279-86	Russia	Beijing	ERR067717	R	R	R	S	R	R	R	F
1805N	Casali et. al. Nat Genet. 2014 Mar;46(3):279-86	Russia	Beijing	ERR144552	S	S	S	S	S	S	U	S
0640X	Casali et. al. Nat Genet. 2014 Mar;46(3):279-86	Russia	LAM	ERR067599	R	R	R	S	R	R	R	S
1921P	Casali et. al. Nat Genet. 2014 Mar;46(3):279-86	Russia	Beijing	ERR144600	R	R	R	S	R	R	R	U
0040V	Casali et. al. Nat Genet. 2014 Mar;46(3):279-86	Russia	Beijing	ERR234596	R	R	R	S	R	R	R	U
1054X	Casali et. al. Nat Genet. 2014 Mar;46(3):279-86	Russia	LAM	ERR133985	R	S	S	S	R	S	S	S
1559W	Casali et. al. Nat Genet. 2014 Mar;46(3):279-86	Russia	Beijing	ERR067715	R	R	R	S	R	U	F	F
1645P	Casali et. al. Nat Genet. 2014 Mar;46(3):279-86	Russia	Beijing	ERR108505	S	S	S	S	S	S	S	S
0660T	Casali et. al. Nat Genet. 2014 Mar;46(3):279-86	Russia	Ural	ERR234586	S	S	S	S	U	S	S	S
2294V	Casali et. al. Nat Genet. 2014 Mar;46(3):279-86	Russia	Beijing	ERR228043	R	R	S	R	R	R	R	R
0991D	Casali et. al. Nat Genet. 2014 Mar;46(3):279-86	Russia	Haarlem	ERR108482	R	R	S	S	R	R	R	R
1418S	Casali et. al. Nat Genet. 2014 Mar;46(3):279-86	Russia	Haarlem	ERR137228	R	S	S	S	R	S	S	S
0752T	Casali et. al. Nat Genet. 2014 Mar;46(3):279-86	Russia	LAM	ERR133922	R	R	S	S	R	R	R	S
1849L	Casali et. al. Nat Genet. 2014 Mar;46(3):279-86	Russia	Ural	ERR144569	S	S	S	S	S	S	S	S
2396F	Casali et. al. Nat Genet. 2014 Mar;46(3):279-86	Russia	lineage4	ERR229990	S	R	R	S	S	R	S	S
0070C	Casali et. al. Nat Genet. 2014 Mar;46(3):279-86	Russia	Beijing	ERR108501	S	S	S	S	S	S	S	S
1949V	Casali et. al. Nat Genet. 2014 Mar;46(3):279-86	Russia	Beijing	ERR108453	R	R	R	R	R	R	R	R
1769Z	Casali et. al. Nat Genet. 2014 Mar;46(3):279-86	Russia	Beijing	ERR144542	R	R	S	R	R	R	S	R
2585L	Casali et. al. Nat Genet. 2014 Mar;46(3):279-86	Russia	Haarlem	ERR234684	S	S	S	S	S	S	S	S
1831R	Casali et. al. Nat Genet. 2014 Mar;46(3):279-86	Russia	Beijing	ERR144564	R	S	S	S	R	S	S	S
0940Y	Casali et. al. Nat Genet. 2014 Mar;46(3):279-86	Russia	Beijing	ERR067641	R	R	S	R	R	R	R	R
2559H	Casali et. al. Nat Genet. 2014 Mar;46(3):279-86	Russia	lineage4	ERR234667	S	S	S	S	S	U	U	S
1024P	Casali et. al. Nat Genet. 2014 Mar;46(3):279-86	Russia	LAM	ERR108487	S	S	S	S	U	S	S	S
0228Z	Casali et. al. Nat Genet. 2014 Mar;46(3):279-86	Russia	Beijing	ERR133820	S	S	S	S	S	S	U	S
0831E	Casali et. al. Nat Genet. 2014 Mar;46(3):279-86	Russia	Beijing	ERR067653	R	R	R	S	R	R	R	R
1067L	Casali et. al. Nat Genet. 2014 Mar;46(3):279-86	Russia	lineage4	ERR108427	S	S	S	S	S	S	S	S
2227X	Casali et. al. Nat Genet. 2014 Mar;46(3):279-86	Russia	LAM	ERR228009	R	R	S	S	R	R	R	S
0545T	Casali et. al. Nat Genet. 2014 Mar;46(3):279-86	Russia	Ural	ERR234616	R	R	S	S	R	S	U	S
2339T	Casali et. al. Nat Genet. 2014 Mar;46(3):279-86	Russia	Beijing	ERR229931	R	R	R	R	R	R	R	R
1815Z	Casali et. al. Nat Genet. 2014 Mar;46(3):279-86	Russia	Beijing	ERR144556	R	R	S	S	F	F	F	F
1473C	Casali et. al. Nat Genet. 2014 Mar;46(3):279-86	Russia	Beijing	ERR067709	R	R	R	R	R	R	R	R
0630L	Casali et. al. Nat Genet. 2014 Mar;46(3):279-86	Russia	Beijing	ERR067766	S	S	S	S	S	S	U	S
2071C	Casali et. al. Nat Genet. 2014 Mar;46(3):279-86	Russia	Beijing	ERR144631	R	R	R	S	R	R	R	S
0903H	Casali et. al. Nat Genet. 2014 Mar;46(3):279-86	Russia	Beijing	ERR133968	R	S	S	S	R	S	S	S
0839N	Casali et. al. Nat Genet. 2014 Mar;46(3):279-86	Russia	Beijing	ERR067659	R	R	S	S	R	R	R	S
0013Q	Casali et. al. Nat Genet. 2014 Mar;46(3):279-86	Russia	Haarlem	ERR108459	S	S	S	S	S	S	S	S
1545F	Casali et. al. Nat Genet. 2014 Mar;46(3):279-86	Russia	Beijing	ERR117465	R	R	S	S	S	S	U	S
0398J	Casali et. al. Nat Genet. 2014 Mar;46(3):279-86	Russia	LAM	ERR067624	R	R	S	S	S	S	S	S
1086G	Casali et. al. Nat Genet. 2014 Mar;46(3):279-86	Russia	lineage4	ERR108448	S	S	S	S	S	S	S	S
0133W	Casali et. al. Nat Genet. 2014 Mar;46(3):279-86	Russia	Beijing	ERR133810	S	S	S	S	S	S	S	S
0915W	Casali et. al. Nat Genet. 2014 Mar;46(3):279-86	Russia	Beijing	ERR067673	R	R	S	S	R	R	R	U
0269T	Casali et. al. Nat Genet. 2014 Mar;46(3):279-86	Russia	lineage4	ERR133830	S	S	S	S	S	S	S	S
0344A	Casali et. al. Nat Genet. 2014 Mar;46(3):279-86	Russia	LAM	ERR067612	R	S	S	R	U	R	R	R
0548X	Casali et. al. Nat Genet. 2014 Mar;46(3):279-86	Russia	Ural	ERR067731	S	S	S	S	U	S	S	S
1524H	Casali et. al. Nat Genet. 2014 Mar;46(3):279-86	Russia	Haarlem	ERR108449	S	R	R	n/a	S	S	S	S
0457Y	Casali et. al. Nat Genet. 2014 Mar;46(3):279-86	Russia	Haarlem	ERR067751	S	S	S	R	S	S	S	S
1617J	Casali et. al. Nat Genet. 2014 Mar;46(3):279-86	Russia	lineage4	ERR067685	S	R	S	S	S	R	S	S
2367Z	Casali et. al. Nat Genet. 2014 Mar;46(3):279-86	Russia	Haarlem	ERR229925	R	S	S	S	R	S	S	S
0788H	Casali et. al. Nat Genet. 2014 Mar;46(3):279-86	Russia	lineage4	ERR133939	S	S	S	S	S	S	S	S
0292T	Casali et. al. Nat Genet. 2014 Mar;46(3):279-86	Russia	Beijing	ERR234590	R	R	R	S	R	R	R	U
1592G	Casali et. al. Nat Genet. 2014 Mar;46(3):279-86	Russia	lineage4	ERR108432	S	S	S	S	S	S	S	S
2520Q	Casali et. al. Nat Genet. 2014 Mar;46(3):279-86	Russia	Beijing	ERR234652	S	S	S	S	S	S	U	S
1353X	Casali et. al. Nat Genet. 2014 Mar;46(3):279-86	Russia	Beijing	ERR137216	R	S	S	R	R	S	R	R
2290Q	Casali et. al. Nat Genet. 2014 Mar;46(3):279-86	Russia	Beijing	ERR228027	S	S	S	S	S	S	S	S
1392P	Casali et. al. Nat Genet. 2014 Mar;46(3):279-86	Russia	Beijing	ERR137223	R	R	R	S	R	R	R	U
2133V	Casali et. al. Nat Genet. 2014 Mar;46(3):279-86	Russia	Beijing	ERR158588	R	R	R	R	R	R	R	F
0889S	Casali et. al. Nat Genet. 2014 Mar;46(3):279-86	Russia	Haarlem	ERR133965	S	S	S	S	S	S	S	S
0177T	Casali et. al. Nat Genet. 2014 Mar;46(3):279-86	Russia	Beijing	ERR067580	S	S	S	S	S	S	S	S
0629K	Casali et. al. Nat Genet. 2014 Mar;46(3):279-86	Russia	Beijing	ERR117453	R	R	S	S	R	R	R	F
2438B	Casali et. al. Nat Genet. 2014 Mar;46(3):279-86	Russia	Haarlem	ERR229959	S	S	S	R	S	U	S	S
2084R	Casali et. al. Nat Genet. 2014 Mar;46(3):279-86	Russia	Beijing	ERR158572	S	S	R	S	S	R	S	S
1616H	Casali et. al. Nat Genet. 2014 Mar;46(3):279-86	Russia	Ural	ERR067684	S	S	S	S	U	S	S	S
1595K	Casali et. al. Nat Genet. 2014 Mar;46(3):279-86	Russia	Beijing	ERR108435	R	R	S	R	R	R	R	R
2033L	Casali et. al. Nat Genet. 2014 Mar;46(3):279-86	Russia	Beijing	ERR144620	R	R	R	R	R	R	R	R
0659S	Casali et. al. Nat Genet. 2014 Mar;46(3):279-86	Russia	lineage4	ERR234578	S	S	S	S	S	S	S	S
0051G	Casali et. al. Nat Genet. 2014 Mar;46(3):279-86	Russia	Beijing	ERR234620	R	S	R	R	R	S	S	S
1193Y	Casali et. al. Nat Genet. 2014 Mar;46(3):279-86	Russia	LAM	ERR067679	R	R	S	S	R	R	R	S
2473P	Casali et. al. Nat Genet. 2014 Mar;46(3):279-86	Russia	Beijing	ERR229920	S	S	S	R	S	S	U	R
0899D	Casali et. al. Nat Genet. 2014 Mar;46(3):279-86	Russia	Beijing	ERR133966	R	R	S	S	R	R	R	S
2385T	Casali et. al. Nat Genet. 2014 Mar;46(3):279-86	Russia	Beijing	ERR229918	R	R	R	S	R	F	F	F
0296Y	Casali et. al. Nat Genet. 2014 Mar;46(3):279-86	Russia	Beijing	ERR133840	S	S	S	S	S	S	S	S
0649G	Casali et. al. Nat Genet. 2014 Mar;46(3):279-86	Russia	Beijing	ERR234641	R	R	S	S	R	R	R	U
1561Y	Casali et. al. Nat Genet. 2014 Mar;46(3):279-86	Russia	Beijing	ERR117467	S	S	S	S	S	S	S	S
1250K	Casali et. al. Nat Genet. 2014 Mar;46(3):279-86	Russia	Beijing	ERR137206	R	R	S	S	R	F	F	F
0847X	Casali et. al. Nat Genet. 2014 Mar;46(3):279-86	Russia	lineage4	ERR067666	S	S	S	S	S	R	S	S
1890F	Casali et. al. Nat Genet. 2014 Mar;46(3):279-86	Russia	Beijing	ERR144588	R	S	S	S	R	S	S	S
0951K	Casali et. al. Nat Genet. 2014 Mar;46(3):279-86	Russia	Beijing	ERR067674	R	R	R	S	R	R	R	U
2405Q	Casali et. al. Nat Genet. 2014 Mar;46(3):279-86	Russia	Beijing	ERR230006	R	R	R	S	R	F	S	S
2478V	Casali et. al. Nat Genet. 2014 Mar;46(3):279-86	Russia	LAM	ERR229928	S	S	S	R	S	S	S	S
0438C	Casali et. al. Nat Genet. 2014 Mar;46(3):279-86	Russia	Beijing	ERR067625	R	R	R	S	R	R	U	S
0757Z	Casali et. al. Nat Genet. 2014 Mar;46(3):279-86	Russia	Beijing	ERR133926	S	S	S	S	S	S	S	S
2484B	Casali et. al. Nat Genet. 2014 Mar;46(3):279-86	Russia	Beijing	ERR229960	R	R	S	S	R	R	R	U
0159Z	Casali et. al. Nat Genet. 2014 Mar;46(3):279-86	Russia	lineage4	ERR067719	R	S	R	S	S	S	U	S
0868V	Casali et. al. Nat Genet. 2014 Mar;46(3):279-86	Russia	Beijing	ERR133958	S	S	S	S	S	S	S	S
0720J	Casali et. al. Nat Genet. 2014 Mar;46(3):279-86	Russia	Beijing	ERR133913	R	S	S	S	R	S	U	S
0469L	Casali et. al. Nat Genet. 2014 Mar;46(3):279-86	Russia	Beijing	ERR234560	R	R	R	S	R	R	R	U
0547W	Casali et. al. Nat Genet. 2014 Mar;46(3):279-86	Russia	Haarlem	ERR067730	S	S	S	S	U	S	S	S
1917K	Casali et. al. Nat Genet. 2014 Mar;46(3):279-86	Russia	Beijing	ERR144597	R	R	S	S	R	R	R	U
1492Y	Casali et. al. Nat Genet. 2014 Mar;46(3):279-86	Russia	Beijing	ERR227982	R	R	R	S	R	R	R	S
0188F	Casali et. al. Nat Genet. 2014 Mar;46(3):279-86	Russia	Beijing	ERR067721	R	R	R	S	R	R	R	U
0663X	Casali et. al. Nat Genet. 2014 Mar;46(3):279-86	Russia	lineage4	ERR234594	R	R	S	S	R	R	S	S
1862A	Casali et. al. Nat Genet. 2014 Mar;46(3):279-86	Russia	Haarlem	ERR144576	R	S	S	S	R	S	S	S
2303E	Casali et. al. Nat Genet. 2014 Mar;46(3):279-86	Russia	LAM	ERR227988	S	S	S	S	S	S	S	S
2337R	Casali et. al. Nat Genet. 2014 Mar;46(3):279-86	Russia	Beijing	ERR229915	R	R	R	S	R	R	R	S
2069A	Casali et. al. Nat Genet. 2014 Mar;46(3):279-86	Russia	lineage4	ERR144629	S	S	S	S	S	S	S	S
2341W	Casali et. al. Nat Genet. 2014 Mar;46(3):279-86	Russia	Beijing	ERR229947	R	R	S	S	R	R	R	S
2007H	Casali et. al. Nat Genet. 2014 Mar;46(3):279-86	Russia	Beijing	ERR144608	R	S	R	R	R	R	R	R

2500T	Casali et. al. Nat Genet. 2014 Mar;46(3):279-86	Russia	Ural	ERR229985	S	S	S	S	U	S	S	S
2154S	Casali et. al. Nat Genet. 2014 Mar;46(3):279-86	Russia	Beijing	ERR158598	R	R	S	S	R	R	R	S
0166G	Casali et. al. Nat Genet. 2014 Mar;46(3):279-86	Russia	Ural	ERR067577	R	R	S	S	R	R	S	S
0379N	Casali et. al. Nat Genet. 2014 Mar;46(3):279-86	Russia	LAM	ERR067737	S	S	S	S	S	S	S	S
0275A	Casali et. al. Nat Genet. 2014 Mar;46(3):279-86	Russia	Haarlem	ERR133832	S	S	S	R	S	U	S	S
0128Q	Casali et. al. Nat Genet. 2014 Mar;46(3):279-86	Russia	lineage4	ERR133807	S	S	S	S	S	S	U	S
2308K	Casali et. al. Nat Genet. 2014 Mar;46(3):279-86	Russia	Beijing	ERR227996	R	R	R	S	R	R	R	U
2411X	Casali et. al. Nat Genet. 2014 Mar;46(3):279-86	Russia	LAM	ERR229927	R	R	S	R	F	R	S	R
2328G	Casali et. al. Nat Genet. 2014 Mar;46(3):279-86	Russia	Beijing	ERR228037	R	R	S	S	R	R	U	S
0061S	Casali et. al. Nat Genet. 2014 Mar;46(3):279-86	Russia	Beijing	ERR067622	R	R	R	S	R	R	R	S
0928K	Casali et. al. Nat Genet. 2014 Mar;46(3):279-86	Russia	Beijing	ERR117460	R	R	R	S	R	R	R	U
0534G	Casali et. al. Nat Genet. 2014 Mar;46(3):279-86	Russia	Beijing	ERR133882	S	R	S	S	S	R	S	S
2535G	Casali et. al. Nat Genet. 2014 Mar;46(3):279-86	Russia	Beijing	ERR234660	R	R	R	S	R	R	R	U
0219P	Casali et. al. Nat Genet. 2014 Mar;46(3):279-86	Russia	LAM	ERR067585	S	S	S	S	S	S	S	S
1323P	Casali et. al. Nat Genet. 2014 Mar;46(3):279-86	Russia	Beijing	ERR108503	R	R	R	S	R	R	R	U
0772Q	Casali et. al. Nat Genet. 2014 Mar;46(3):279-86	Russia	Beijing	ERR133929	S	S	S	S	S	S	S	S
2390Z	Casali et. al. Nat Genet. 2014 Mar;46(3):279-86	Russia	lineage4	ERR229942	S	S	S	S	S	S	S	S
0728S	Casali et. al. Nat Genet. 2014 Mar;46(3):279-86	Russia	lineage4	ERR108457	R	S	R	R	R	S	U	U
2207A	Casali et. al. Nat Genet. 2014 Mar;46(3):279-86	Russia	Beijing	ERR228024	R	R	R	R	R	R	R	R
0028G	Casali et. al. Nat Genet. 2014 Mar;46(3):279-86	Russia	lineage4	ERR067614	S	S	S	S	S	S	S	S
0522T	Casali et. al. Nat Genet. 2014 Mar;46(3):279-86	Russia	Beijing	ERR234584	R	R	R	R	R	R	R	R
0014R	Casali et. al. Nat Genet. 2014 Mar;46(3):279-86	Russia	Ural	ERR234564	R	S	S	S	R	F	F	S
1049R	Casali et. al. Nat Genet. 2014 Mar;46(3):279-86	Russia	Beijing	ERR108420	R	R	R	S	R	R	R	S
2536H	Casali et. al. Nat Genet. 2014 Mar;46(3):279-86	Russia	Beijing	ERR234661	R	R	R	R	R	R	R	R
0591T	Casali et. al. Nat Genet. 2014 Mar;46(3):279-86	Russia	Beijing	ERR133890	R	R	S	S	R	R	R	R
1065J	Casali et. al. Nat Genet. 2014 Mar;46(3):279-86	Russia	Ural	ERR108425	R	R	S	S	R	R	R	S
1076W	Casali et. al. Nat Genet. 2014 Mar;46(3):279-86	Russia	Haarlem	ERR108445	S	S	S	R	S	S	S	S
1607Y	Casali et. al. Nat Genet. 2014 Mar;46(3):279-86	Russia	lineage4	ERR067680	S	S	S	S	S	S	S	S
2486D	Casali et. al. Nat Genet. 2014 Mar;46(3):279-86	Russia	Beijing	ERR229976	R	R	R	S	R	R	R	S
2080M	Casali et. al. Nat Genet. 2014 Mar;46(3):279-86	Russia	Beijing	ERR144636	R	R	R	R	R	R	R	U
0967C	Casali et. al. Nat Genet. 2014 Mar;46(3):279-86	Russia	Beijing	ERR108473	R	R	S	S	R	R	R	U
1659E	Casali et. al. Nat Genet. 2014 Mar;46(3):279-86	Russia	Beijing	ERR108507	R	R	R	S	R	R	R	S
0509E	Casali et. al. Nat Genet. 2014 Mar;46(3):279-86	Russia	Beijing	ERR067759	R	S	S	S	R	S	S	S
0912S	Casali et. al. Nat Genet. 2014 Mar;46(3):279-86	Russia	Haarlem	ERR133971	S	S	S	S	S	S	S	S
0669D	Casali et. al. Nat Genet. 2014 Mar;46(3):279-86	Russia	Beijing	ERR133902	R	R	S	S	R	R	R	U
2462C	Casali et. al. Nat Genet. 2014 Mar;46(3):279-86	Russia	LAM	ERR229983	R	S	S	S	R	S	S	S
2271V	Casali et. al. Nat Genet. 2014 Mar;46(3):279-86	Russia	LAM	ERR228034	S	S	S	S	S	S	S	S
2175Q	Casali et. al. Nat Genet. 2014 Mar;46(3):279-86	Russia	Beijing	ERR228015	S	S	S	S	S	S	U	S
0068A	Casali et. al. Nat Genet. 2014 Mar;46(3):279-86	Russia	Beijing	ERR108499	R	R	S	S	R	R	S	S
0829C	Casali et. al. Nat Genet. 2014 Mar;46(3):279-86	Russia	Beijing	ERR133954	R	R	R	R	R	R	R	U
2498R	Casali et. al. Nat Genet. 2014 Mar;46(3):279-86	Russia	Beijing	ERR229969	R	R	S	R	R	R	R	R
0328H	Casali et. al. Nat Genet. 2014 Mar;46(3):279-86	Russia	Beijing	ERR133847	S	S	S	S	S	S	S	S
0255D	Casali et. al. Nat Genet. 2014 Mar;46(3):279-86	Russia	Beijing	ERR133826	R	R	R	S	R	R	R	U
2092A	Casali et. al. Nat Genet. 2014 Mar;46(3):279-86	Russia	LAM	ERR158579	R	R	S	R	R	R	R	U
1080A	Casali et. al. Nat Genet. 2014 Mar;46(3):279-86	Russia	Beijing	ERR133988	S	S	S	S	S	S	S	S
1638G	Casali et. al. Nat Genet. 2014 Mar;46(3):279-86	Russia	lineage4	ERR137251	S	S	S	S	S	S	S	S
2490H	Casali et. al. Nat Genet. 2014 Mar;46(3):279-86	Russia	Beijing	ERR230008	R	R	S	S	R	R	R	R
2237H	Casali et. al. Nat Genet. 2014 Mar;46(3):279-86	Russia	LAM	ERR228033	S	S	S	S	S	S	S	S
1648S	Casali et. al. Nat Genet. 2014 Mar;46(3):279-86	Russia	Beijing	ERR137252	S	S	S	S	S	S	S	S
2395E	Casali et. al. Nat Genet. 2014 Mar;46(3):279-86	Russia	Beijing	ERR229982	R	R	R	R	R	R	R	R
1555R	Casali et. al. Nat Genet. 2014 Mar;46(3):279-86	Russia	LAM	ERR117466	R	S	S	S	R	R	R	S
0635R	Casali et. al. Nat Genet. 2014 Mar;46(3):279-86	Russia	Ural	ERR067596	R	S	S	S	R	S	S	S
0439D	Casali et. al. Nat Genet. 2014 Mar;46(3):279-86	Russia	lineage4	ERR234599	S	S	S	S	S	S	S	S
1136L	Casali et. al. Nat Genet. 2014 Mar;46(3):279-86	Russia	Beijing	ERR108496	S	S	S	S	S	U	S	S
2512G	Casali et. al. Nat Genet. 2014 Mar;46(3):279-86	Russia	Beijing	ERR229962	R	R	R	S	R	R	R	U
1334B	Casali et. al. Nat Genet. 2014 Mar;46(3):279-86	Russia	lineage4	ERR137213	R	S	S	S	S	S	S	S
2492K	Casali et. al. Nat Genet. 2014 Mar;46(3):279-86	Russia	Beijing	ERR229929	R	R	R	R	R	R	F	F
0145J	Casali et. al. Nat Genet. 2014 Mar;46(3):279-86	Russia	Beijing	ERR158613	R	R	R	S	R	R	R	U
0532E	Casali et. al. Nat Genet. 2014 Mar;46(3):279-86	Russia	Beijing	ERR133880	R	R	S	R	R	R	R	R
0925G	Casali et. al. Nat Genet. 2014 Mar;46(3):279-86	Russia	S-type	ERR133974	S	S	S	S	S	S	S	S
0429S	Casali et. al. Nat Genet. 2014 Mar;46(3):279-86	Russia	Ural	ERR067725	S	S	S	S	S	S	S	S
2185B	Casali et. al. Nat Genet. 2014 Mar;46(3):279-86	Russia	lineage4	ERR228055	S	R	S	S	S	F	S	S
1755J	Casali et. al. Nat Genet. 2014 Mar;46(3):279-86	Russia	Beijing	ERR137281	R	R	S	S	R	R	R	U
0525X	Casali et. al. Nat Genet. 2014 Mar;46(3):279-86	Russia	Beijing	ERR133875	S	S	R	S	S	S	S	S
1797E	Casali et. al. Nat Genet. 2014 Mar;46(3):279-86	Russia	Beijing	ERR144549	R	R	S	S	R	R	R	U
1470Z	Casali et. al. Nat Genet. 2014 Mar;46(3):279-86	Russia	LAM	ERR067706	S	S	S	S	S	S	S	S
0651J	Casali et. al. Nat Genet. 2014 Mar;46(3):279-86	Russia	Beijing	ERR234562	R	R	R	S	R	R	S	S
1030W	Casali et. al. Nat Genet. 2014 Mar;46(3):279-86	Russia	Beijing	ERR108489	R	R	S	S	R	R	R	U
0284K	Casali et. al. Nat Genet. 2014 Mar;46(3):279-86	Russia	Beijing	ERR234582	S	S	S	S	S	S	S	S
0981S	Casali et. al. Nat Genet. 2014 Mar;46(3):279-86	Russia	lineage4	ERR108478	S	S	S	S	S	S	S	S
2201T	Casali et. al. Nat Genet. 2014 Mar;46(3):279-86	Russia	lineage4	ERR228000	S	S	S	S	S	S	S	S
2314R	Casali et. al. Nat Genet. 2014 Mar;46(3):279-86	Russia	LAM	ERR228036	R	R	R	R	R	R	R	R
0050F	Casali et. al. Nat Genet. 2014 Mar;46(3):279-86	Russia	Beijing	ERR067619	R	R	R	S	R	R	R	R
2356M	Casali et. al. Nat Genet. 2014 Mar;46(3):279-86	Russia	Beijing	ERR229940	R	R	S	S	R	R	S	S
2502W	Casali et. al. Nat Genet. 2014 Mar;46(3):279-86	Russia	lineage4	ERR230001	S	S	S	S	S	S	S	S
2352H	Casali et. al. Nat Genet. 2014 Mar;46(3):279-86	Russia	Beijing	ERR229924	R	R	R	S	R	R	R	U
2403N	Casali et. al. Nat Genet. 2014 Mar;46(3):279-86	Russia	Beijing	ERR229998	R	R	R	S	R	R	R	R
1818C	Casali et. al. Nat Genet. 2014 Mar;46(3):279-86	Russia	Beijing	ERR144558	R	S	S	S	R	R	R	S
0353K	Casali et. al. Nat Genet. 2014 Mar;46(3):279-86	Russia	Ural	ERR117451	S	S	S	S	S	S	U	S
1695T	Casali et. al. Nat Genet. 2014 Mar;46(3):279-86	Russia	lineage4	ERR137261	S	S	S	S	S	S	S	S
0502X	Casali et. al. Nat Genet. 2014 Mar;46(3):279-86	Russia	Ural	ERR067757	S	S	S	S	S	S	S	S
2636R	Casali et. al. Nat Genet. 2014 Mar;46(3):279-86	Russia	Ural	ERR234697	S	S	S	S	S	S	U	S
1504L	Casali et. al. Nat Genet. 2014 Mar;46(3):279-86	Russia	Ural	ERR137239	S	S	S	S	S	S	S	S
0282H	Casali et. al. Nat Genet. 2014 Mar;46(3):279-86	Russia	Beijing	ERR133837	R	R	S	S	R	F	S	S
1511T	Casali et. al. Nat Genet. 2014 Mar;46(3):279-86	Russia	Haarlem	ERR227998	S	S	S	S	S	S	S	S
0154T	Casali et. al. Nat Genet. 2014 Mar;46(3):279-86	Russia	LAM	ERR067767	S	S	S	S	U	S	S	S
2073E	Casali et. al. Nat Genet. 2014 Mar;46(3):279-86	Russia	Beijing	ERR144633	R	R	R	S	R	R	R	U
1860Y	Casali et. al. Nat Genet. 2014 Mar;46(3):279-86	Russia	Beijing	ERR144575	R	R	S	S	R	R	R	U
1309Z	Casali et. al. Nat Genet. 2014 Mar;46(3):279-86	Russia	Beijing	ERR137212	R	S	S	S	R	S	S	S
2214H	Casali et. al. Nat Genet. 2014 Mar;46(3):279-86	Russia	Beijing	ERR228056	R	R	R	R	R	R	R	R
0011N	Casali et. al. Nat Genet. 2014 Mar;46(3):279-86	Russia	Beijing	ERR234556	R	R	R	S	R	R	R	R
2513H	Casali et. al. Nat Genet. 2014 Mar;46(3):279-86	Russia	Beijing	ERR229970	S	S	S	S	S	S	S	S
2353J	Casali et. al. Nat Genet. 2014 Mar;46(3):279-86	Russia	Haarlem	ERR229932	R	R	R	S	R	R	R	S
1588C	Casali et. al. Nat Genet. 2014 Mar;46(3):279-86	Russia	Beijing	ERR137249	S	S	S	S	S	S	S	S
1535V	Casali et. al. Nat Genet. 2014 Mar;46(3):279-86	Russia	Beijing	ERR228014	R	S	S	S	R	S	S	S
1053W	Casali et. al. Nat Genet. 2014 Mar;46(3):279-86	Russia	LAM	ERR108422	R	R	S	R	R	F	F	F
0638V	Casali et. al. Nat Genet. 2014 Mar;46(3):279-86	Russia	Beijing	ERR234609	R	R	R	S	R	R	R	U
2089X	Casali et. al. Nat Genet. 2014 Mar;46(3):279-86	Russia	Beijing	ERR158576	R	R	R	S	R	R	R	U
1432H	Casali et. al. Nat Genet. 2014 Mar;46(3):279-86	Russia	Beijing	ERR137230	R	R	R	S	R	R	R	U
0043Y	Casali et. al. Nat Genet. 2014 Mar;46(3):279-86	Russia	Haarlem	ERR067617	S	S	S	S	S	S	S	S
1743W	Casali et. al. Nat Genet. 2014 Mar;46(3):279-86	Russia	Beijing	ERR137279	S	S	S	S	S	S	S	S
1634C	Casali et. al. Nat Genet. 2014 Mar;46(3):279-86	Russia	LAM	ERR067690	S	S	S	R	S	S	S	S
0365Y	Casali et. al. Nat Genet. 2014 Mar;46(3):279-86	Russia	Beijing	ERR234630	R	R	R	R	R	R	R	U
0730V	Casali et. al. Nat Genet. 2014 Mar;46(3):279-86	Russia	LAM	ERR133917	R	R	S	S	R	S	S	S
0860L	Casali et. al. Nat Genet. 2014 Mar;46(3):279-86	Russia	Beijing	ERR117457	S	S	S	S	S	S	S	S
0869W	Casali et. al. Nat Genet. 2014 Mar;46(3):279-86	Russia	Beijing	ERR133959	R	R	S	S	R	R	R	U
2525W	Casali et. al. Nat Genet. 2014 Mar;46(3):279-86	Russia	Beijing	ERR234653	R	R	S	S	R	R	R	U
1190V	Casali et. al. Nat Genet. 2014 Mar;46(3):279-86	Russia	Ural	ERR067677	R	R	S	R	R	R	S	R
0790K	Casali et. al. Nat Genet. 2014 Mar;46(3):279-86	Russia	Beijing	ERR133941	S	S	S	S	S	S	S	S
0826Z	Casali et. al. Nat Genet. 2014 Mar;46(3):279-86	Russia	Beijing	ERR133953	R	R	R	S	R	R	R	U
2481Y	Casali et. al. Nat Genet. 2014 Mar;46(3):279-86	Russia	Beijing	ERR229936	R	R	R	R	R	R	R	R
0905K	Casali et. al. Nat Genet. 2014 Mar;46(3):279-86	Russia	Beijing	ERR067668	R	R	R	R	R	R	F	F
2570V	Casali et. al. Nat Genet. 2014 Mar;46(3):279-86	Russia	Ural	ERR234674	R	S	S	S	R	S	S	S
1623Q	Casali et. al. Nat Genet. 2014 Mar;46(3):279-86	Russia	Beijing	ERR067686	R	S	S	S	S	S	S	S
0990C	Casali et. al. Nat Genet. 2014 Mar;46(3):279-86	Russia	S-type	ERR108481	S	S	S	S	S	S	S	S
0848Y	Casali et. al. Nat Genet. 2014 Mar;46(3):279-86	Russia	Beijing	ERR067667	R	R	R	S	R	R	R	U

0840P	Casali et. al. Nat Genet. 2014 Mar;46(3):279-86	Russia	Beijing	ERR067660	R	R	S	R	R	R	R	S
0018W	Casali et. al. Nat Genet. 2014 Mar;46(3):279-86	Russia	Haarlem	ERR108461	S	S	S	S	S	S	U	S
0833G	Casali et. al. Nat Genet. 2014 Mar;46(3):279-86	Russia	Beijing	ERR067654	R	R	R	n/a	R	R	R	U
1920N	Casali et. al. Nat Genet. 2014 Mar;46(3):279-86	Russia	Beijing	ERR144599	R	S	S	S	R	S	S	S
1472B	Casali et. al. Nat Genet. 2014 Mar;46(3):279-86	Russia	lineage4	ERR067708	S	S	S	S	S	S	S	S
1017G	Casali et. al. Nat Genet. 2014 Mar;46(3):279-86	Russia	Beijing	ERR108486	S	S	S	S	S	S	U	S
0286M	Casali et. al. Nat Genet. 2014 Mar;46(3):279-86	Russia	LAM	ERR117450	S	S	S	S	S	S	S	S
2286L	Casali et. al. Nat Genet. 2014 Mar;46(3):279-86	Russia	LAM	ERR228011	S	S	S	S	S	S	S	S
2111W	Casali et. al. Nat Genet. 2014 Mar;46(3):279-86	Russia	Ural	ERR158582	S	S	S	S	S	S	S	S
1717S	Casali et. al. Nat Genet. 2014 Mar;46(3):279-86	Russia	Beijing	ERR137268	S	S	S	S	S	S	S	S
1570H	Casali et. al. Nat Genet. 2014 Mar;46(3):279-86	Russia	Beijing	ERR137247	R	R	R	R	R	R	R	R
0200T	Casali et. al. Nat Genet. 2014 Mar;46(3):279-86	Russia	Ural	ERR133819	S	S	S	S	U	S	S	S
0665Z	Casali et. al. Nat Genet. 2014 Mar;46(3):279-86	Russia	Beijing	ERR234610	R	R	S	S	R	R	R	S
0277C	Casali et. al. Nat Genet. 2014 Mar;46(3):279-86	Russia	lineage4	ERR133833	S	S	S	S	S	S	S	S
0996J	Casali et. al. Nat Genet. 2014 Mar;46(3):279-86	Russia	lineage4	ERR133980	R	S	S	S	S	S	U	S
2594W	Casali et. al. Nat Genet. 2014 Mar;46(3):279-86	Russia	Beijing	ERR234690	S	S	S	S	S	S	S	S
0838M	Casali et. al. Nat Genet. 2014 Mar;46(3):279-86	Russia	LAM	ERR067658	S	S	S	S	S	S	S	S
1945Q	Casali et. al. Nat Genet. 2014 Mar;46(3):279-86	Russia	Ural	ERR144603	S	S	S	S	S	S	S	S
2291R	Casali et. al. Nat Genet. 2014 Mar;46(3):279-86	Russia	lineage4	ERR228035	S	S	S	S	S	S	S	S
1060D	Casali et. al. Nat Genet. 2014 Mar;46(3):279-86	Russia	Beijing	ERR108423	R	S	S	R	R	S	S	U
0799V	Casali et. al. Nat Genet. 2014 Mar;46(3):279-86	Russia	Beijing	ERR133944	R	R	S	S	R	R	R	U
1187R	Casali et. al. Nat Genet. 2014 Mar;46(3):279-86	Russia	lineage4	ERR067676	S	S	S	S	S	S	S	S
0944C	Casali et. al. Nat Genet. 2014 Mar;46(3):279-86	Russia	Beijing	ERR067644	R	R	R	R	R	R	R	R
1773D	Casali et. al. Nat Genet. 2014 Mar;46(3):279-86	Russia	Beijing	ERR144545	R	S	S	R	R	S	S	R
0384T	Casali et. al. Nat Genet. 2014 Mar;46(3):279-86	Russia	Beijing	ERR067740	S	S	S	S	S	S	S	S
0896A	Casali et. al. Nat Genet. 2014 Mar;46(3):279-86	Russia	Beijing	ERR234563	R	R	S	R	R	R	R	S
2006G	Casali et. al. Nat Genet. 2014 Mar;46(3):279-86	Russia	Beijing	ERR144607	R	R	R	R	R	R	R	R
1901S	Casali et. al. Nat Genet. 2014 Mar;46(3):279-86	Russia	Beijing	ERR144593	R	R	S	S	R	R	R	U
2589Q	Casali et. al. Nat Genet. 2014 Mar;46(3):279-86	Russia	Ural	ERR234686	S	S	S	S	S	S	S	S
2199R	Casali et. al. Nat Genet. 2014 Mar;46(3):279-86	Russia	Ural	ERR227992	S	S	S	S	S	S	S	S
2501V	Casali et. al. Nat Genet. 2014 Mar;46(3):279-86	Russia	Beijing	ERR229993	R	R	R	R	R	R	R	U
0978P	Casali et. al. Nat Genet. 2014 Mar;46(3):279-86	Russia	Beijing	ERR108475	R	R	R	S	R	R	R	U
1042J	Casali et. al. Nat Genet. 2014 Mar;46(3):279-86	Russia	Beijing	ERR133983	S	S	S	S	S	S	S	S
1596L	Casali et. al. Nat Genet. 2014 Mar;46(3):279-86	Russia	Beijing	ERR108436	R	R	R	S	R	R	R	U
0887Q	Casali et. al. Nat Genet. 2014 Mar;46(3):279-86	Russia	Beijing	ERR133963	R	S	S	S	R	S	S	S
0942A	Casali et. al. Nat Genet. 2014 Mar;46(3):279-86	Russia	Beijing	ERR133975	R	S	S	S	R	S	S	S
0786F	Casali et. al. Nat Genet. 2014 Mar;46(3):279-86	Russia	lineage4	ERR133937	R	R	S	S	R	R	U	S
0639W	Casali et. al. Nat Genet. 2014 Mar;46(3):279-86	Russia	Beijing	ERR133898	S	S	S	S	S	S	S	S
0601E	Casali et. al. Nat Genet. 2014 Mar;46(3):279-86	Russia	Ural	ERR234561	S	S	S	R	S	S	S	S
0448N	Casali et. al. Nat Genet. 2014 Mar;46(3):279-86	Russia	Ural	ERR067748	R	R	S	S	R	R	R	S
2323B	Casali et. al. Nat Genet. 2014 Mar;46(3):279-86	Russia	Ural	ERR227997	R	S	S	S	R	S	S	S
0311P	Casali et. al. Nat Genet. 2014 Mar;46(3):279-86	Russia	Beijing	ERR133844	R	R	R	S	R	R	R	R
2072D	Casali et. al. Nat Genet. 2014 Mar;46(3):279-86	Russia	Beijing	ERR144632	R	R	R	S	R	R	R	U
1770A	Casali et. al. Nat Genet. 2014 Mar;46(3):279-86	Russia	Beijing	ERR144543	R	R	S	S	R	R	R	U
2211E	Casali et. al. Nat Genet. 2014 Mar;46(3):279-86	Russia	Beijing	ERR228048	R	S	S	S	R	S	S	S
0459A	Casali et. al. Nat Genet. 2014 Mar;46(3):279-86	Russia	LAM	ERR067591	R	R	R	S	S	U	S	S
1633B	Casali et. al. Nat Genet. 2014 Mar;46(3):279-86	Russia	Beijing	ERR067689	S	S	S	S	S	S	S	S
0352J	Casali et. al. Nat Genet. 2014 Mar;46(3):279-86	Russia	Beijing	ERR067694	R	R	S	S	R	R	R	S
0468K	Casali et. al. Nat Genet. 2014 Mar;46(3):279-86	Russia	Beijing	ERR234647	S	S	S	S	S	S	S	S
0498S	Casali et. al. Nat Genet. 2014 Mar;46(3):279-86	Russia	Haarlem	ERR133870	S	S	S	S	S	S	S	S
0617X	Casali et. al. Nat Genet. 2014 Mar;46(3):279-86	Russia	lineage4	ERR133897	S	S	S	S	S	S	S	S
0367A	Casali et. al. Nat Genet. 2014 Mar;46(3):279-86	Russia	Beijing	ERR067695	R	R	S	S	R	R	R	U
0916X	Casali et. al. Nat Genet. 2014 Mar;46(3):279-86	Russia	Beijing	ERR117458	S	S	S	S	S	S	S	S
0748P	Casali et. al. Nat Genet. 2014 Mar;46(3):279-86	Russia	Beijing	ERR133919	R	R	R	S	R	R	R	S
2381P	Casali et. al. Nat Genet. 2014 Mar;46(3):279-86	Russia	Beijing	ERR229997	R	R	S	S	R	R	R	S
0520R	Casali et. al. Nat Genet. 2014 Mar;46(3):279-86	Russia	lineage4	ERR133874	S	S	S	S	S	S	S	S
0346C	Casali et. al. Nat Genet. 2014 Mar;46(3):279-86	Russia	Ural	ERR133849	R	R	S	R	R	R	R	S
1636E	Casali et. al. Nat Genet. 2014 Mar;46(3):279-86	Russia	Ural	ERR067691	R	R	S	S	R	R	S	S
1506N	Casali et. al. Nat Genet. 2014 Mar;46(3):279-86	Russia	Beijing	ERR117462	R	R	S	S	R	R	R	S
0023B	Casali et. al. Nat Genet. 2014 Mar;46(3):279-86	Russia	Haarlem	ERR234580	S	S	S	S	S	S	S	S
0435Z	Casali et. al. Nat Genet. 2014 Mar;46(3):279-86	Russia	Beijing	ERR133865	R	S	S	S	R	S	S	S
2273X	Casali et. al. Nat Genet. 2014 Mar;46(3):279-86	Russia	Beijing	ERR228042	R	R	S	R	R	R	R	F
1766W	Casali et. al. Nat Genet. 2014 Mar;46(3):279-86	Russia	LAM	ERR137284	R	R	R	R	R	R	U	R
1446Y	Casali et. al. Nat Genet. 2014 Mar;46(3):279-86	Russia	Beijing	ERR137231	R	R	R	R	R	R	R	R
0878F	Casali et. al. Nat Genet. 2014 Mar;46(3):279-86	Russia	Beijing	ERR108441	S	S	S	S	S	S	S	S
0319Y	Casali et. al. Nat Genet. 2014 Mar;46(3):279-86	Russia	LAM	ERR133846	S	S	S	S	S	S	S	S
0613S	Casali et. al. Nat Genet. 2014 Mar;46(3):279-86	Russia	Beijing	ERR234585	R	S	S	S	R	S	S	S
2095D	Casali et. al. Nat Genet. 2014 Mar;46(3):279-86	Russia	Beijing	ERR158580	R	R	S	R	R	R	R	S
1864C	Casali et. al. Nat Genet. 2014 Mar;46(3):279-86	Russia	Beijing	ERR144577	S	S	S	S	S	S	S	S
2010L	Casali et. al. Nat Genet. 2014 Mar;46(3):279-86	Russia	Beijing	ERR144610	S	S	S	S	S	S	S	S
1070P	Casali et. al. Nat Genet. 2014 Mar;46(3):279-86	Russia	Beijing	ERR133987	S	S	S	S	S	S	S	S
1660F	Casali et. al. Nat Genet. 2014 Mar;46(3):279-86	Russia	lineage4	ERR108508	S	S	S	S	S	S	S	S
0044Z	Casali et. al. Nat Genet. 2014 Mar;46(3):279-86	Russia	Beijing	ERR067618	R	R	S	R	R	R	R	R
2035N	Casali et. al. Nat Genet. 2014 Mar;46(3):279-86	Russia	Beijing	ERR144622	R	R	R	R	R	R	R	R
0593W	Casali et. al. Nat Genet. 2014 Mar;46(3):279-86	Russia	Beijing	ERR133892	R	R	R	n/a	R	R	R	U
2280E	Casali et. al. Nat Genet. 2014 Mar;46(3):279-86	Russia	Beijing	ERR227987	R	R	S	S	R	R	R	S
0281G	Casali et. al. Nat Genet. 2014 Mar;46(3):279-86	Russia	lineage4	ERR133836	R	R	S	S	S	S	S	S
2318W	Casali et. al. Nat Genet. 2014 Mar;46(3):279-86	Russia	Beijing	ERR228068	S	S	S	S	S	S	S	S
2366Y	Casali et. al. Nat Genet. 2014 Mar;46(3):279-86	Russia	LAM	ERR229917	S	S	S	S	S	S	S	S
0415C	Casali et. al. Nat Genet. 2014 Mar;46(3):279-86	Russia	Beijing	ERR067744	R	R	S	S	R	R	R	S
2382Q	Casali et. al. Nat Genet. 2014 Mar;46(3):279-86	Russia	Beijing	ERR230005	R	R	R	R	R	R	R	U
0910Q	Casali et. al. Nat Genet. 2014 Mar;46(3):279-86	Russia	Haarlem	ERR067670	R	S	S	S	R	S	R	S
0544S	Casali et. al. Nat Genet. 2014 Mar;46(3):279-86	Russia	LAM	ERR067729	S	S	S	S	U	S	S	S
2216K	Casali et. al. Nat Genet. 2014 Mar;46(3):279-86	Russia	Beijing	ERR227977	R	R	R	S	R	R	R	S
2127N	Casali et. al. Nat Genet. 2014 Mar;46(3):279-86	Russia	Beijing	ERR158585	R	R	R	S	R	R	R	U
1519C	Casali et. al. Nat Genet. 2014 Mar;46(3):279-86	Russia	Beijing	ERR137242	R	S	S	S	R	S	R	S
0897B	Casali et. al. Nat Genet. 2014 Mar;46(3):279-86	Russia	Beijing	ERR108458	R	R	S	S	R	R	R	U
2505Z	Casali et. al. Nat Genet. 2014 Mar;46(3):279-86	Russia	Beijing	ERR229922	R	R	R	S	R	R	R	S
2139B	Casali et. al. Nat Genet. 2014 Mar;46(3):279-86	Russia	Beijing	ERR158591	S	S	S	R	S	S	S	S
2433W	Casali et. al. Nat Genet. 2014 Mar;46(3):279-86	Russia	LAM	ERR229943	S	S	S	S	S	S	S	S
1942M	Casali et. al. Nat Genet. 2014 Mar;46(3):279-86	Russia	lineage4	ERR144602	S	S	S	S	S	F	S	S
0734Z	Casali et. al. Nat Genet. 2014 Mar;46(3):279-86	Russia	Beijing	ERR117455	S	S	S	S	S	S	S	S
0195N	Casali et. al. Nat Genet. 2014 Mar;46(3):279-86	Russia	Haarlem	ERR234558	S	S	S	R	S	U	S	S
0575B	Casali et. al. Nat Genet. 2014 Mar;46(3):279-86	Russia	Ural	ERR234648	R	R	S	S	R	R	R	S
0364X	Casali et. al. Nat Genet. 2014 Mar;46(3):279-86	Russia	LAM	ERR133851	S	S	S	S	U	S	S	S
0322B	Casali et. al. Nat Genet. 2014 Mar;46(3):279-86	Russia	Beijing	ERR234598	R	R	R	S	R	R	R	U
2234E	Casali et. al. Nat Genet. 2014 Mar;46(3):279-86	Russia	Beijing	ERR228017	R	R	S	S	R	R	R	U
0604H	Casali et. al. Nat Genet. 2014 Mar;46(3):279-86	Russia	Ural	ERR234577	R	S	S	S	R	S	S	S
0233E	Casali et. al. Nat Genet. 2014 Mar;46(3):279-86	Russia	Beijing	ERR067586	R	S	R	R	R	S	R	R
2526X	Casali et. al. Nat Genet. 2014 Mar;46(3):279-86	Russia	Ural	ERR234654	R	R	R	S	R	R	R	S
0125M	Casali et. al. Nat Genet. 2014 Mar;46(3):279-86	Russia	Beijing	ERR133805	R	R	S	R	R	R	R	R
2097F	Casali et. al. Nat Genet. 2014 Mar;46(3):279-86	Russia	Beijing	ERR158581	R	R	R	S	R	R	R	S
0793N	Casali et. al. Nat Genet. 2014 Mar;46(3):279-86	Russia	Haarlem	ERR133942	R	R	R	R	R	R	R	R
1627V	Casali et. al. Nat Genet. 2014 Mar;46(3):279-86	Russia	Beijing	ERR067688	S	S	S	S	S	S	S	S
0217M	Casali et. al. Nat Genet. 2014 Mar;46(3):279-86	Russia	Beijing	ERR067583	R	R	R	S	R	R	R	U
2514J	Casali et. al. Nat Genet. 2014 Mar;46(3):279-86	Russia	Beijing	ERR229978	R	R	R	S	R	R	R	S
0888R	Casali et. al. Nat Genet. 2014 Mar;46(3):279-86	Russia	lineage4	ERR133964	S	S	S	S	S	S	S	S
0658R	Casali et. al. Nat Genet. 2014 Mar;46(3):279-86	Russia	Beijing	ERR234570	R	R	S	S	R	R	R	U
1082C	Casali et. al. Nat Genet. 2014 Mar;46(3):279-86	Russia	Beijing	ERR108447	R	S	S	S	R	S	R	S
0761D	Casali et. al. Nat Genet. 2014 Mar;46(3):279-86	Russia	Beijing	ERR108467	R	R	R	S	R	R	R	U
1744X	Casali et. al. Nat Genet. 2014 Mar;46(3):279-86	Russia	LAM	ERR137280	S	S	S	S	S	S	S	S
0600D	Casali et. al. Nat Genet. 2014 Mar;46(3):279-86	Russia	LAM	ERR117452	R	R	S	S	R	R	R	R
0609N	Casali et. al. Nat Genet. 2014 Mar;46(3):279-86	Russia	Beijing	ERR133895	R	R	S	S	R	R	R	U
0514K	Casali et. al. Nat Genet. 2014 Mar;46(3):279-86	Russia	Beijing	ERR067592	R	R	S	R	R	R	R	U
2198Q	Casali et. al. Nat Genet. 2014 Mar;46(3):279-86	Russia	Beijing	ERR227984	S	S	S	S	S	S	S	S
2169J	Casali et. al. Nat Genet. 2014 Mar;46(3):279-86	Russia	Beijing	ERR227983	S	R	R	S	R	R	R	S

2243P	Casali et. al. Nat Genet. 2014 Mar;46(3):279-86	Russia	Beijing	ERR228049	R	R	R	R	R	R	R	F
1461P	Casali et. al. Nat Genet. 2014 Mar;46(3):279-86	Russia	Beijing	ERR234651	R	S	R	R	R	F	R	R
2388X	Casali et. al. Nat Genet. 2014 Mar;46(3):279-86	Russia	LAM	ERR229934	S	S	S	S	S	S	S	S
2639V	Casali et. al. Nat Genet. 2014 Mar;46(3):279-86	Russia	Beijing	ERR234698	R	R	S	S	R	R	R	U
0066Y	Casali et. al. Nat Genet. 2014 Mar;46(3):279-86	Russia	Beijing	ERR108498	R	R	R	R	R	R	R	R
1919M	Casali et. al. Nat Genet. 2014 Mar;46(3):279-86	Russia	Ural	ERR144598	R	S	S	S	R	S	S	R
0355M	Casali et. al. Nat Genet. 2014 Mar;46(3):279-86	Russia	Haarlem	ERR133850	S	S	S	S	S	S	S	S
0450Q	Casali et. al. Nat Genet. 2014 Mar;46(3):279-86	Russia	Beijing	ERR067750	R	R	R	R	R	R	R	R
0218N	Casali et. al. Nat Genet. 2014 Mar;46(3):279-86	Russia	Beijing	ERR067584	R	R	R	S	R	R	R	U
2078K	Casali et. al. Nat Genet. 2014 Mar;46(3):279-86	Russia	Beijing	ERR144634	R	R	R	S	R	R	R	S
2324C	Casali et. al. Nat Genet. 2014 Mar;46(3):279-86	Russia	LAM	ERR228005	S	S	S	S	S	S	S	S
1191W	Casali et. al. Nat Genet. 2014 Mar;46(3):279-86	Russia	LAM	ERR137201	R	S	S	S	R	S	S	S
0873A	Casali et. al. Nat Genet. 2014 Mar;46(3):279-86	Russia	Beijing	ERR108440	S	S	S	S	S	S	S	S
2335P	Casali et. al. Nat Genet. 2014 Mar;46(3):279-86	Russia	Beijing	ERR228061	R	R	R	S	R	R	R	U
1858W	Casali et. al. Nat Genet. 2014 Mar;46(3):279-86	Russia	Beijing	ERR144574	R	R	R	S	R	R	R	U
0399K	Casali et. al. Nat Genet. 2014 Mar;46(3):279-86	Russia	lineage4	ERR133857	S	S	S	S	S	S	S	S
1705E	Casali et. al. Nat Genet. 2014 Mar;46(3):279-86	Russia	Beijing	ERR137262	R	R	R	S	R	R	R	U
1397V	Casali et. al. Nat Genet. 2014 Mar;46(3):279-86	Russia	lineage4	ERR137224	S	S	S	S	S	S	S	S
2625E	Casali et. al. Nat Genet. 2014 Mar;46(3):279-86	Russia	Ural	ERR234693	R	S	S	S	R	S	S	S
0668C	Casali et. al. Nat Genet. 2014 Mar;46(3):279-86	Russia	Beijing	ERR133901	R	R	R	S	R	R	R	R
2592T	Casali et. al. Nat Genet. 2014 Mar;46(3):279-86	Russia	Beijing	ERR234688	R	S	R	R	R	R	R	R
2049D	Casali et. al. Nat Genet. 2014 Mar;46(3):279-86	Russia	Beijing	ERR144625	R	R	S	S	S	F	S	S
2386V	Casali et. al. Nat Genet. 2014 Mar;46(3):279-86	Russia	Beijing	ERR229926	R	R	R	S	R	R	R	S
0678N	Casali et. al. Nat Genet. 2014 Mar;46(3):279-86	Russia	Haarlem	ERR234634	S	S	R	S	S	U	S	S
0818Q	Casali et. al. Nat Genet. 2014 Mar;46(3):279-86	Russia	Beijing	ERR067651	R	R	R	S	R	R	R	U
1820E	Casali et. al. Nat Genet. 2014 Mar;46(3):279-86	Russia	Haarlem	ERR144559	R	S	S	S	R	S	S	S
1256R	Casali et. al. Nat Genet. 2014 Mar;46(3):279-86	Russia	Beijing	ERR137207	S	S	R	S	S	S	U	S
1164R	Casali et. al. Nat Genet. 2014 Mar;46(3):279-86	Russia	Delhi	ERR137193	S	S	S	S	S	S	S	S
1268E	Casali et. al. Nat Genet. 2014 Mar;46(3):279-86	Russia	Beijing	ERR137208	R	R	R	R	R	R	R	R
1767X	Casali et. al. Nat Genet. 2014 Mar;46(3):279-86	Russia	Beijing	ERR137285	S	S	S	S	S	S	S	S
0972H	Casali et. al. Nat Genet. 2014 Mar;46(3):279-86	Russia	Ural	ERR133978	S	S	S	S	S	S	S	S
2371D	Casali et. al. Nat Genet. 2014 Mar;46(3):279-86	Russia	Beijing	ERR229949	R	R	R	R	R	R	R	R
0671F	Casali et. al. Nat Genet. 2014 Mar;46(3):279-86	Russia	LAM	ERR133903	R	R	R	R	R	R	U	R
2487E	Casali et. al. Nat Genet. 2014 Mar;46(3):279-86	Russia	Beijing	ERR229984	R	R	R	S	R	R	R	R
2312P	Casali et. al. Nat Genet. 2014 Mar;46(3):279-86	Russia	Beijing	ERR228028	R	R	R	R	R	R	R	R
2302D	Casali et. al. Nat Genet. 2014 Mar;46(3):279-86	Russia	Beijing	ERR227980	R	S	R	S	R	S	R	S
0770N	Casali et. al. Nat Genet. 2014 Mar;46(3):279-86	Russia	Beijing	ERR108469	R	R	R	S	R	R	R	U
2090Y	Casali et. al. Nat Genet. 2014 Mar;46(3):279-86	Russia	Beijing	ERR158577	S	S	S	S	S	S	S	S
0402N	Casali et. al. Nat Genet. 2014 Mar;46(3):279-86	Russia	Beijing	ERR067588	R	R	S	S	R	R	R	S
0904J	Casali et. al. Nat Genet. 2014 Mar;46(3):279-86	Russia	Ural	ERR133969	R	S	S	S	R	S	S	S
0816N	Casali et. al. Nat Genet. 2014 Mar;46(3):279-86	Russia	Beijing	ERR067649	R	R	S	S	R	R	R	U
0835J	Casali et. al. Nat Genet. 2014 Mar;46(3):279-86	Russia	Beijing	ERR067656	R	R	S	S	R	R	R	U
2236G	Casali et. al. Nat Genet. 2014 Mar;46(3):279-86	Russia	S-type	ERR228025	R	R	S	S	R	F	S	S
2219N	Casali et. al. Nat Genet. 2014 Mar;46(3):279-86	Russia	Ural	ERR227985	R	R	R	R	R	R	R	R
2052G	Casali et. al. Nat Genet. 2014 Mar;46(3):279-86	Russia	Beijing	ERR144628	R	R	R	S	R	R	R	U
2177S	Casali et. al. Nat Genet. 2014 Mar;46(3):279-86	Russia	lineage4	ERR228023	S	S	S	S	S	S	S	S
1342K	Casali et. al. Nat Genet. 2014 Mar;46(3):279-86	Russia	Beijing	ERR137215	R	R	S	S	S	S	S	S
1361F	Casali et. al. Nat Genet. 2014 Mar;46(3):279-86	Russia	Beijing	ERR234603	S	S	S	S	S	S	S	S
1772C	Casali et. al. Nat Genet. 2014 Mar;46(3):279-86	Russia	LAM	ERR144544	S	S	S	S	S	S	U	S
0696H	Casali et. al. Nat Genet. 2014 Mar;46(3):279-86	Russia	Beijing	ERR133906	R	R	R	R	R	R	R	U
0183A	Casali et. al. Nat Genet. 2014 Mar;46(3):279-86	Russia	Beijing	ERR234645	R	R	R	S	R	S	R	S
1455H	Casali et. al. Nat Genet. 2014 Mar;46(3):279-86	Russia	Ural	ERR137233	R	S	R	S	R	S	R	S
0075H	Casali et. al. Nat Genet. 2014 Mar;46(3):279-86	Russia	Beijing	ERR117469	R	S	S	S	R	S	S	S
0458Z	Casali et. al. Nat Genet. 2014 Mar;46(3):279-86	Russia	Beijing	ERR133866	R	R	S	S	R	R	R	U
0507C	Casali et. al. Nat Genet. 2014 Mar;46(3):279-86	Russia	Ural	ERR067728	R	R	S	S	R	R	R	F
0725P	Casali et. al. Nat Genet. 2014 Mar;46(3):279-86	Russia	Beijing	ERR133915	R	R	S	R	R	R	R	U
0590S	Casali et. al. Nat Genet. 2014 Mar;46(3):279-86	Russia	Beijing	ERR133889	R	R	R	S	R	R	R	U
1507P	Casali et. al. Nat Genet. 2014 Mar;46(3):279-86	Russia	LAM	ERR227990	R	S	S	S	R	S	U	R
2440D	Casali et. al. Nat Genet. 2014 Mar;46(3):279-86	Russia	lineage4	ERR229967	R	S	S	S	R	S	S	S
1409H	Casali et. al. Nat Genet. 2014 Mar;46(3):279-86	Russia	Beijing	ERR137225	R	R	S	R	R	R	R	F
1709J	Casali et. al. Nat Genet. 2014 Mar;46(3):279-86	Russia	Beijing	ERR137264	R	R	S	S	R	R	R	R
0650H	Casali et. al. Nat Genet. 2014 Mar;46(3):279-86	Russia	Beijing	ERR234649	R	R	S	R	R	R	R	R
2182Y	Casali et. al. Nat Genet. 2014 Mar;46(3):279-86	Russia	lineage4	ERR228047	S	S	S	S	S	S	S	S
0945D	Casali et. al. Nat Genet. 2014 Mar;46(3):279-86	Russia	Ural	ERR067645	R	R	S	S	R	R	U	S
0465G	Casali et. al. Nat Genet. 2014 Mar;46(3):279-86	Russia	Beijing	ERR067631	R	R	S	S	R	R	R	S
0530C	Casali et. al. Nat Genet. 2014 Mar;46(3):279-86	Russia	Beijing	ERR133879	R	R	R	R	R	R	R	R
2008J	Casali et. al. Nat Genet. 2014 Mar;46(3):279-86	Russia	Beijing	ERR144609	R	S	S	S	U	S	S	S
1518B	Casali et. al. Nat Genet. 2014 Mar;46(3):279-86	Russia	Beijing	ERR137241	S	S	S	S	S	S	S	S
0351H	Casali et. al. Nat Genet. 2014 Mar;46(3):279-86	Russia	LAM	ERR067693	S	S	S	S	S	S	S	S
1186Q	Casali et. al. Nat Genet. 2014 Mar;46(3):279-86	Russia	Beijing	ERR137198	R	S	S	S	R	S	S	S
2171L	Casali et. al. Nat Genet. 2014 Mar;46(3):279-86	Russia	Beijing	ERR227999	S	S	S	S	S	S	S	S
0026E	Casali et. al. Nat Genet. 2014 Mar;46(3):279-86	Russia	Beijing	ERR108514	R	R	R	S	R	R	U	U
2342X	Casali et. al. Nat Genet. 2014 Mar;46(3):279-86	Russia	Beijing	ERR229955	R	R	R	S	R	R	R	S
0392C	Casali et. al. Nat Genet. 2014 Mar;46(3):279-86	Russia	Beijing	ERR234559	S	S	S	S	S	S	S	S
1359D	Casali et. al. Nat Genet. 2014 Mar;46(3):279-86	Russia	Beijing	ERR137218	R	R	R	S	R	R	R	S
2533E	Casali et. al. Nat Genet. 2014 Mar;46(3):279-86	Russia	Beijing	ERR234658	R	R	R	R	R	R	R	S
2315S	Casali et. al. Nat Genet. 2014 Mar;46(3):279-86	Russia	Haarlem	ERR228044	R	S	S	S	R	S	S	S
1551M	Casali et. al. Nat Genet. 2014 Mar;46(3):279-86	Russia	Ural	ERR137245	S	S	S	S	S	S	S	S
1012B	Casali et. al. Nat Genet. 2014 Mar;46(3):279-86	Russia	Ural	ERR108485	R	R	S	S	R	R	S	S
2494M	Casali et. al. Nat Genet. 2014 Mar;46(3):279-86	Russia	Beijing	ERR229945	S	S	S	S	S	S	S	S
0390A	Casali et. al. Nat Genet. 2014 Mar;46(3):279-86	Russia	Beijing	ERR067724	R	R	S	R	R	R	R	S
0834H	Casali et. al. Nat Genet. 2014 Mar;46(3):279-86	Russia	Beijing	ERR067655	R	R	S	S	S	S	S	S
2568S	Casali et. al. Nat Genet. 2014 Mar;46(3):279-86	Russia	Beijing	ERR234672	R	R	R	R	R	R	R	U
2358P	Casali et. al. Nat Genet. 2014 Mar;46(3):279-86	Russia	lineage4	ERR229956	S	S	S	S	S	S	S	S
1465T	Casali et. al. Nat Genet. 2014 Mar;46(3):279-86	Russia	Beijing	ERR137235	S	S	S	S	S	S	S	S
1832S	Casali et. al. Nat Genet. 2014 Mar;46(3):279-86	Russia	Ural	ERR144565	R	S	R	S	R	S	S	S
1780L	Casali et. al. Nat Genet. 2014 Mar;46(3):279-86	Russia	LAM	ERR144547	S	S	S	S	U	S	S	S
0992E	Casali et. al. Nat Genet. 2014 Mar;46(3):279-86	Russia	LAM	ERR108483	S	S	S	S	U	S	S	S
2209C	Casali et. al. Nat Genet. 2014 Mar;46(3):279-86	Russia	Beijing	ERR228040	R	S	S	S	R	S	S	S
1137M	Casali et. al. Nat Genet. 2014 Mar;46(3):279-86	Russia	Ural	ERR108497	S	S	S	S	S	S	S	S
1782N	Casali et. al. Nat Genet. 2014 Mar;46(3):279-86	Russia	Beijing	ERR144548	R	R	S	S	R	R	R	U
1102Z	Casali et. al. Nat Genet. 2014 Mar;46(3):279-86	Russia	lineage4	ERR137191	S	S	S	S	S	S	S	S
0020Y	Casali et. al. Nat Genet. 2014 Mar;46(3):279-86	Russia	Beijing	ERR108462	R	R	R	R	R	R	R	R
0782B	Casali et. al. Nat Genet. 2014 Mar;46(3):279-86	Russia	lineage4	ERR133934	S	S	S	S	S	S	S	S
0527Z	Casali et. al. Nat Genet. 2014 Mar;46(3):279-86	Russia	LAM	ERR133876	S	S	S	S	S	S	S	S
0817P	Casali et. al. Nat Genet. 2014 Mar;46(3):279-86	Russia	Beijing	ERR067650	R	R	S	S	R	R	R	U
0268S	Casali et. al. Nat Genet. 2014 Mar;46(3):279-86	Russia	lineage4	ERR133829	S	S	S	S	S	S	S	S
2070B	Casali et. al. Nat Genet. 2014 Mar;46(3):279-86	Russia	Beijing	ERR144630	S	S	S	S	S	S	S	S
1391N	Casali et. al. Nat Genet. 2014 Mar;46(3):279-86	Russia	Beijing	ERR137222	R	R	S	S	R	R	R	U
2150N	Casali et. al. Nat Genet. 2014 Mar;46(3):279-86	Russia	Beijing	ERR158596	R	R	S	S	R	R	R	R
1739R	Casali et. al. Nat Genet. 2014 Mar;46(3):279-86	Russia	Beijing	ERR137275	R	R	R	R	R	R	R	R
1657C	Casali et. al. Nat Genet. 2014 Mar;46(3):279-86	Russia	Beijing	ERR137255	R	R	S	R	R	R	R	R
2283H	Casali et. al. Nat Genet. 2014 Mar;46(3):279-86	Russia	Beijing	ERR228003	R	R	S	S	R	R	R	S
2170K	Casali et. al. Nat Genet. 2014 Mar;46(3):279-86	Russia	Ural	ERR227991	S	S	S	S	S	S	S	S
0124L	Casali et. al. Nat Genet. 2014 Mar;46(3):279-86	Russia	Ural	ERR133804	R	S	S	S	R	S	S	S
2317V	Casali et. al. Nat Genet. 2014 Mar;46(3):279-86	Russia	Beijing	ERR228060	S	S	S	S	S	S	U	S
1451D	Casali et. al. Nat Genet. 2014 Mar;46(3):279-86	Russia	Beijing	ERR137232	S	S	S	S	S	S	S	S
0366Z	Casali et. al. Nat Genet. 2014 Mar;46(3):279-86	Russia	Beijing	ERR234638	S	S	S	S	S	S	S	S
0555E	Casali et. al. Nat Genet. 2014 Mar;46(3):279-86	Russia	lineage4	ERR067732	R	S	R	R	F	F	S	F
2391A	Casali et. al. Nat Genet. 2014 Mar;46(3):279-86	Russia	Haarlem	ERR229950	S	S	S	S	S	S	S	S
0508D	Casali et. al. Nat Genet. 2014 Mar;46(3):279-86	Russia	Beijing	ERR067758	R	R	R	S	R	R	R	U
0628J	Casali et. al. Nat Genet. 2014 Mar;46(3):279-86	Russia	Beijing	ERR067613	R	R	S	S	R	R	R	U
0822V	Casali et. al. Nat Genet. 2014 Mar;46(3):279-86	Russia	LAM	ERR133949	S	S	S	S	S	S	S	S
1908A	Casali et. al. Nat Genet. 2014 Mar;46(3):279-86	Russia	Beijing	ERR144596	R	S	R	R	R	R	R	R
1757L	Casali et. al. Nat Genet. 2014 Mar;46(3):279-86	Russia	Beijing	ERR137282	R	R	S	S	R	R	U	S
0953M	Casali et. al. Nat Genet. 2014 Mar;46(3):279-86	Russia	Haarlem	ERR067675	S	S	S	S	S	S	S	S

0345B	Casali et. al. Nat Genet. 2014 Mar;46(3):279-86	Russia	Beijing	ERR067587	R	R	S	S	R	R	R	U
2491J	Casali et. al. Nat Genet. 2014 Mar;46(3):279-86	Russia	Beijing	ERR229921	R	R	R	S	R	R	R	U
0016T	Casali et. al. Nat Genet. 2014 Mar;46(3):279-86	Russia	lineage4	ERR108460	S	S	S	S	S	S	S	S
1542C	Casali et. al. Nat Genet. 2014 Mar;46(3):279-86	Russia	Beijing	ERR108452	S	S	S	S	S	F	S	S
1852P	Casali et. al. Nat Genet. 2014 Mar;46(3):279-86	Russia	Beijing	ERR144571	R	R	R	R	R	R	R	R
0667B	Casali et. al. Nat Genet. 2014 Mar;46(3):279-86	Russia	Beijing	ERR133900	R	R	R	R	R	R	S	R
0404Q	Casali et. al. Nat Genet. 2014 Mar;46(3):279-86	Russia	Beijing	ERR067590	R	R	R	R	R	R	R	R
2408T	Casali et. al. Nat Genet. 2014 Mar;46(3):279-86	Russia	Ural	ERR229919	S	S	S	S	S	S	S	S
0552B	Casali et. al. Nat Genet. 2014 Mar;46(3):279-86	Russia	Beijing	ERR234624	R	S	S	S	R	S	S	S
0290R	Casali et. al. Nat Genet. 2014 Mar;46(3):279-86	Russia	Ural	ERR133838	R	S	S	S	R	S	U	S
0385V	Casali et. al. Nat Genet. 2014 Mar;46(3):279-86	Russia	Beijing	ERR234646	R	R	S	S	R	R	R	S
0433X	Casali et. al. Nat Genet. 2014 Mar;46(3):279-86	Russia	Beijing	ERR133863	S	S	S	S	S	S	S	S
1011A	Casali et. al. Nat Genet. 2014 Mar;46(3):279-86	Russia	Ural	ERR133981	R	R	R	S	U	R	R	S
2340V	Casali et. al. Nat Genet. 2014 Mar;46(3):279-86	Russia	Beijing	ERR229939	S	S	S	S	S	S	S	S
0512H	Casali et. al. Nat Genet. 2014 Mar;46(3):279-86	Russia	Beijing	ERR067762	R	R	S	S	R	R	R	U
0906L	Casali et. al. Nat Genet. 2014 Mar;46(3):279-86	Russia	Beijing	ERR133970	R	S	S	R	R	R	R	U
1680C	Casali et. al. Nat Genet. 2014 Mar;46(3):279-86	Russia	Haarlem	ERR137256	S	S	S	S	S	S	S	S
0042X	Casali et. al. Nat Genet. 2014 Mar;46(3):279-86	Russia	Beijing	ERR117449	S	S	S	S	S	S	U	S
0700M	Casali et. al. Nat Genet. 2014 Mar;46(3):279-86	Russia	Beijing	ERR133907	R	R	R	S	R	R	R	S
2248V	Casali et. al. Nat Genet. 2014 Mar;46(3):279-86	Russia	lineage4	ERR228065	S	S	S	S	S	S	S	S
2591S	Casali et. al. Nat Genet. 2014 Mar;46(3):279-86	Russia	LAM	ERR234687	S	S	S	S	S	S	S	S
1871K	Casali et. al. Nat Genet. 2014 Mar;46(3):279-86	Russia	Beijing	ERR144582	S	S	S	S	S	S	U	S
1741T	Casali et. al. Nat Genet. 2014 Mar;46(3):279-86	Russia	Beijing	ERR137277	R	R	R	S	R	R	R	U
2499S	Casali et. al. Nat Genet. 2014 Mar;46(3):279-86	Russia	Beijing	ERR229977	S	S	S	S	S	S	S	S
0273Y	Casali et. al. Nat Genet. 2014 Mar;46(3):279-86	Russia	Beijing	ERR133831	R	R	R	S	R	R	R	U
1870J	Casali et. al. Nat Genet. 2014 Mar;46(3):279-86	Russia	Beijing	ERR144581	R	R	S	R	R	R	U	S
0111X	Casali et. al. Nat Genet. 2014 Mar;46(3):279-86	Russia	Beijing	ERR234637	R	R	R	R	R	R	R	U
2247T	Casali et. al. Nat Genet. 2014 Mar;46(3):279-86	Russia	lineage4	ERR228057	S	S	S	S	S	S	S	S
1650V	Casali et. al. Nat Genet. 2014 Mar;46(3):279-86	Russia	Beijing	ERR108506	S	S	S	R	S	S	U	R
0373G	Casali et. al. Nat Genet. 2014 Mar;46(3):279-86	Russia	Haarlem	ERR067697	R	R	S	R	R	R	R	R
0430T	Casali et. al. Nat Genet. 2014 Mar;46(3):279-86	Russia	lineage4	ERR067726	S	S	S	S	S	S	S	S
1126A	Casali et. al. Nat Genet. 2014 Mar;46(3):279-86	Russia	Beijing	ERR108493	R	R	R	S	R	R	R	U
0653L	Casali et. al. Nat Genet. 2014 Mar;46(3):279-86	Russia	Beijing	ERR133899	R	R	R	S	R	R	R	R
0283J	Casali et. al. Nat Genet. 2014 Mar;46(3):279-86	Russia	Beijing	ERR234574	R	R	S	S	R	R	R	U
0784D	Casali et. al. Nat Genet. 2014 Mar;46(3):279-86	Russia	Beijing	ERR133935	R	R	R	S	R	R	R	S
0960V	Casali et. al. Nat Genet. 2014 Mar;46(3):279-86	Russia	Beijing	ERR067635	R	R	S	S	R	R	R	U
1691P	Casali et. al. Nat Genet. 2014 Mar;46(3):279-86	Russia	LAM	ERR137258	S	S	S	S	S	S	S	S
1373T	Casali et. al. Nat Genet. 2014 Mar;46(3):279-86	Russia	lineage4	ERR234627	R	R	S	S	R	R	S	S
1029V	Casali et. al. Nat Genet. 2014 Mar;46(3):279-86	Russia	Beijing	ERR108488	R	R	S	R	R	R	R	S
2511F	Casali et. al. Nat Genet. 2014 Mar;46(3):279-86	Russia	Haarlem	ERR229954	S	S	S	S	S	S	S	S
1346P	Casali et. al. Nat Genet. 2014 Mar;46(3):279-86	Russia	Beijing	ERR117470	R	R	R	S	R	R	R	U
1879T	Casali et. al. Nat Genet. 2014 Mar;46(3):279-86	Russia	lineage4	ERR144585	S	S	S	S	S	S	S	S
2081N	Casali et. al. Nat Genet. 2014 Mar;46(3):279-86	Russia	Beijing	ERR158569	S	R	S	S	S	R	S	S
0137A	Casali et. al. Nat Genet. 2014 Mar;46(3):279-86	Russia	LAM	ERR133811	S	S	S	S	S	S	S	S
0403P	Casali et. al. Nat Genet. 2014 Mar;46(3):279-86	Russia	Beijing	ERR067589	S	S	S	S	S	R	S	S
0060R	Casali et. al. Nat Genet. 2014 Mar;46(3):279-86	Russia	Beijing	ERR234557	R	R	R	S	S	S	U	S
2357N	Casali et. al. Nat Genet. 2014 Mar;46(3):279-86	Russia	lineage4	ERR229948	S	S	S	S	S	S	S	S
0541P	Casali et. al. Nat Genet. 2014 Mar;46(3):279-86	Russia	Beijing	ERR234608	R	R	S	S	R	R	R	R
0779Y	Casali et. al. Nat Genet. 2014 Mar;46(3):279-86	Russia	Beijing	ERR108470	S	S	S	S	S	S	S	S
0397H	Casali et. al. Nat Genet. 2014 Mar;46(3):279-86	Russia	Beijing	ERR133856	R	S	S	S	R	R	S	S
1370Q	Casali et. al. Nat Genet. 2014 Mar;46(3):279-86	Russia	Beijing	ERR234619	S	S	S	S	S	S	S	S
1522F	Casali et. al. Nat Genet. 2014 Mar;46(3):279-86	Russia	Beijing	ERR137243	R	R	R	R	R	R	R	R
2493L	Casali et. al. Nat Genet. 2014 Mar;46(3):279-86	Russia	lineage4	ERR229937	S	S	S	S	S	S	U	S
1475E	Casali et. al. Nat Genet. 2014 Mar;46(3):279-86	Russia	Beijing	ERR067711	R	R	R	S	R	R	R	U
0466H	Casali et. al. Nat Genet. 2014 Mar;46(3):279-86	Russia	Beijing	ERR234639	R	R	R	R	R	R	R	F
1723Z	Casali et. al. Nat Genet. 2014 Mar;46(3):279-86	Russia	Beijing	ERR117468	R	R	S	S	R	R	R	U
2376J	Casali et. al. Nat Genet. 2014 Mar;46(3):279-86	Russia	LAM	ERR229973	S	S	S	S	S	S	S	S
0069B	Casali et. al. Nat Genet. 2014 Mar;46(3):279-86	Russia	Beijing	ERR108500	R	R	S	S	R	R	R	R
1072R	Casali et. al. Nat Genet. 2014 Mar;46(3):279-86	Russia	Ural	ERR108429	R	S	S	S	R	S	S	S
1127B	Casali et. al. Nat Genet. 2014 Mar;46(3):279-86	Russia	Beijing	ERR108494	R	R	R	R	R	F	F	F
2379M	Casali et. al. Nat Genet. 2014 Mar;46(3):279-86	Russia	LAM	ERR229989	R	S	S	S	R	S	U	S
0890T	Casali et. al. Nat Genet. 2014 Mar;46(3):279-86	Russia	lineage4	ERR108442	S	S	S	S	S	S	S	S
0157X	Casali et. al. Nat Genet. 2014 Mar;46(3):279-86	Russia	Beijing	ERR067718	R	R	R	S	R	R	R	U
2162B	Casali et. al. Nat Genet. 2014 Mar;46(3):279-86	Russia	Beijing	ERR228062	R	R	R	R	R	R	R	R
1552N	Casali et. al. Nat Genet. 2014 Mar;46(3):279-86	Russia	Beijing	ERR137246	R	R	R	R	R	R	R	R
1735M	Casali et. al. Nat Genet. 2014 Mar;46(3):279-86	Russia		ERR137271	R	R	R	R	R	R	F	F
1586A	Casali et. al. Nat Genet. 2014 Mar;46(3):279-86	Russia	Beijing	ERR137248	R	R	S	R	R	R	R	R
0536J	Casali et. al. Nat Genet. 2014 Mar;46(3):279-86	Russia	Haarlem	ERR133883	S	S	S	S	S	S	S	S
1712M	Casali et. al. Nat Genet. 2014 Mar;46(3):279-86	Russia	Beijing	ERR137267	R	S	S	S	R	S	R	S
2360R	Casali et. al. Nat Genet. 2014 Mar;46(3):279-86	Russia	Beijing	ERR229964	R	S	S	S	R	R	S	S
1613E	Casali et. al. Nat Genet. 2014 Mar;46(3):279-86	Russia	lineage4	ERR067682	S	S	S	S	S	S	S	S
1594J	Casali et. al. Nat Genet. 2014 Mar;46(3):279-86	Russia	Beijing	ERR108434	S	S	S	S	S	S	S	S
0030J	Casali et. al. Nat Genet. 2014 Mar;46(3):279-86	Russia	Haarlem	ERR067616	S	S	S	S	S	S	S	S
0702P	Casali et. al. Nat Genet. 2014 Mar;46(3):279-86	Russia	lineage4	ERR133909	R	R	S	R	R	S	S	S
0236H	Casali et. al. Nat Genet. 2014 Mar;46(3):279-86	Russia	Ural	ERR133823	R	R	R	S	R	S	S	S
0473Q	Casali et. al. Nat Genet. 2014 Mar;46(3):279-86	Russia	Ural	ERR067753	S	S	S	S	S	S	S	S
1854R	Casali et. al. Nat Genet. 2014 Mar;46(3):279-86	Russia	Beijing	ERR144573	R	R	R	S	R	R	R	R
0080N	Casali et. al. Nat Genet. 2014 Mar;46(3):279-86	Russia	Beijing	ERR234573	R	R	S	S	R	R	R	S
1903V	Casali et. al. Nat Genet. 2014 Mar;46(3):279-86	Russia	Beijing	ERR144594	R	S	S	S	R	S	S	S
0285L	Casali et. al. Nat Genet. 2014 Mar;46(3):279-86	Russia	Beijing	ERR067723	R	R	S	S	R	R	R	S
0126N	Casali et. al. Nat Genet. 2014 Mar;46(3):279-86	Russia	Beijing	ERR133806	R	R	S	S	R	R	R	U
0616W	Casali et. al. Nat Genet. 2014 Mar;46(3):279-86	Russia	Beijing	ERR133896	R	R	S	S	R	R	R	R
2187D	Casali et. al. Nat Genet. 2014 Mar;46(3):279-86	Russia	lineage4	ERR228063	S	S	S	S	S	S	S	S
0278D	Casali et. al. Nat Genet. 2014 Mar;46(3):279-86	Russia	Ural	ERR133834	R	R	S	S	R	R	S	S
0378M	Casali et. al. Nat Genet. 2014 Mar;46(3):279-86	Russia	Beijing	ERR067736	S	S	S	S	S	S	S	S
1269F	Casali et. al. Nat Genet. 2014 Mar;46(3):279-86	Russia	LAM	ERR137209	R	R	S	R	R	R	U	R
0902G	Casali et. al. Nat Genet. 2014 Mar;46(3):279-86	Russia	Ural	ERR133967	S	S	S	S	U	S	S	S
0358Q	Casali et. al. Nat Genet. 2014 Mar;46(3):279-86	Russia	Beijing	ERR158616	R	R	R	R	R	R	R	R
1731H	Casali et. al. Nat Genet. 2014 Mar;46(3):279-86	Russia	Beijing	ERR137269	R	R	S	S	R	R	R	U
1079Z	Casali et. al. Nat Genet. 2014 Mar;46(3):279-86	Russia	LAM	ERR108446	S	S	S	S	U	S	S	S
0802Y	Casali et. al. Nat Genet. 2014 Mar;46(3):279-86	Russia	Beijing	ERR133945	R	S	S	R	R	R	R	F
1073S	Casali et. al. Nat Genet. 2014 Mar;46(3):279-86	Russia	Beijing	ERR108443	R	R	R	R	R	R	R	S
2215J	Casali et. al. Nat Genet. 2014 Mar;46(3):279-86	Russia	Beijing	ERR228064	R	R	S	S	R	R	R	U
1833T	Casali et. al. Nat Genet. 2014 Mar;46(3):279-86	Russia	Beijing	ERR144566	R	R	R	S	R	R	S	R
2392B	Casali et. al. Nat Genet. 2014 Mar;46(3):279-86	Russia	Haarlem	ERR229958	S	S	S	S	S	S	S	S
2441E	Casali et. al. Nat Genet. 2014 Mar;46(3):279-86	Russia	Beijing	ERR229975	S	S	S	S	S	S	S	S
1457K	Casali et. al. Nat Genet. 2014 Mar;46(3):279-86	Russia	Beijing	ERR137234	R	R	S	R	R	R	R	R
0939X	Casali et. al. Nat Genet. 2014 Mar;46(3):279-86	Russia	lineage4	ERR067640	S	S	S	S	U	S	S	S
2195M	Casali et. al. Nat Genet. 2014 Mar;46(3):279-86	Russia	Beijing	ERR227976	R	R	S	S	R	R	U	S
2050E	Casali et. al. Nat Genet. 2014 Mar;46(3):279-86	Russia	Beijing	ERR144626	S	S	S	S	S	S	S	S
2276A	Casali et. al. Nat Genet. 2014 Mar;46(3):279-86	Russia	Ural	ERR228058	R	S	S	S	R	S	U	S
1233R	Casali et. al. Nat Genet. 2014 Mar;46(3):279-86	Russia	Beijing	ERR137204	R	R	R	R	R	R	R	U
2347C	Casali et. al. Nat Genet. 2014 Mar;46(3):279-86	Russia	Haarlem	ERR229987	S	S	S	S	S	S	S	S
1742V	Casali et. al. Nat Genet. 2014 Mar;46(3):279-86	Russia	Beijing	ERR137278	S	S	S	S	S	R	S	S
2257E	Casali et. al. Nat Genet. 2014 Mar;46(3):279-86	Russia	Beijing	ERR228010	S	S	S	S	S	S	U	S
0406S	Casali et. al. Nat Genet. 2014 Mar;46(3):279-86	Russia	Beijing	ERR067742	R	R	S	S	R	R	R	U
0348E	Casali et. al. Nat Genet. 2014 Mar;46(3):279-86	Russia	Beijing	ERR067692	R	R	R	S	R	R	R	S
0175R	Casali et. al. Nat Genet. 2014 Mar;46(3):279-86	Russia	Beijing	ERR067578	R	R	S	R	R	R	R	U
0570W	Casali et. al. Nat Genet. 2014 Mar;46(3):279-86	Russia	Beijing	ERR133886	R	R	S	S	R	R	R	U
0672G	Casali et. al. Nat Genet. 2014 Mar;46(3):279-86	Russia	Beijing	ERR133904	S	S	S	S	S	S	S	S
2497Q	Casali et. al. Nat Genet. 2014 Mar;46(3):279-86	Russia	Beijing	ERR229961	R	S	R	S	R	R	R	S
0400L	Casali et. al. Nat Genet. 2014 Mar;46(3):279-86	Russia	Ural	ERR133858	R	R	R	S	R	R	S	S
2515K	Casali et. al. Nat Genet. 2014 Mar;46(3):279-86	Russia	Ural	ERR229986	S	S	S	S	S	S	S	S
1694S	Casali et. al. Nat Genet. 2014 Mar;46(3):279-86	Russia	Beijing	ERR137260	R	R	S	S	R	R	R	S
1487S	Casali et. al. Nat Genet. 2014 Mar;46(3):279-86	Russia	Beijing	ERR067713	S	S	S	S	S	S	S	S
0153S	Casali et. al. Nat Genet. 2014 Mar;46(3):279-86	Russia	Beijing	ERR133815	R	R	S	R	R	S	S	R

2363V	Casali et. al. Nat Genet. 2014 Mar;46(3):279-86	Russia	Ural	ERR229988	R	R	S	S	R	R	F	S
0923E	Casali et. al. Nat Genet. 2014 Mar;46(3):279-86	Russia	Beijing	ERR133973	S	S	S	S	S	R	S	S
1450C	Casali et. al. Nat Genet. 2014 Mar;46(3):279-86	Russia	Beijing	ERR108513	S	S	S	S	S	S	S	S
2287M	Casali et. al. Nat Genet. 2014 Mar;46(3):279-86	Russia	Beijing	ERR228019	R	R	S	S	R	R	R	U
0280F	Casali et. al. Nat Genet. 2014 Mar;46(3):279-86	Russia	lineage4	ERR133835	R	S	S	R	R	S	S	S
1837Y	Casali et. al. Nat Genet. 2014 Mar;46(3):279-86	Russia	Beijing	ERR144567	R	R	R	R	R	R	R	R
1850M	Casali et. al. Nat Genet. 2014 Mar;46(3):279-86	Russia	Beijing	ERR144570	R	R	S	S	R	R	U	S
2495N	Casali et. al. Nat Genet. 2014 Mar;46(3):279-86	Russia	Ural	ERR229953	R	R	S	S	R	R	R	S
0573Z	Casali et. al. Nat Genet. 2014 Mar;46(3):279-86	Russia	Ural	ERR067733	R	R	S	S	R	R	R	R
0717F	Casali et. al. Nat Genet. 2014 Mar;46(3):279-86	Russia	LAM	ERR133912	S	S	S	S	S	S	S	S
0705S	Casali et. al. Nat Genet. 2014 Mar;46(3):279-86	Russia	Ural	ERR133911	R	S	S	S	R	S	S	S
2134W	Casali et. al. Nat Genet. 2014 Mar;46(3):279-86	Russia	Beijing	ERR158589	R	S	R	S	R	S	S	S
2485C	Casali et. al. Nat Genet. 2014 Mar;46(3):279-86	Russia	Beijing	ERR229968	R	R	R	S	R	R	R	R
0805B	Casali et. al. Nat Genet. 2014 Mar;46(3):279-86	Russia	Beijing	ERR133946	S	R	S	S	S	S	S	S
0911R	Casali et. al. Nat Genet. 2014 Mar;46(3):279-86	Russia	Beijing	ERR067671	R	R	R	S	R	R	R	U
2298Z	Casali et. al. Nat Genet. 2014 Mar;46(3):279-86	Russia	S-type	ERR228067	S	S	S	S	S	S	S	S
1074T	Casali et. al. Nat Genet. 2014 Mar;46(3):279-86	Russia	Haarlem	ERR108444	S	S	S	S	S	S	S	S
1192X	Casali et. al. Nat Genet. 2014 Mar;46(3):279-86	Russia	lineage4	ERR067678	R	R	S	S	S	S	S	S
0812J	Casali et. al. Nat Genet. 2014 Mar;46(3):279-86	Russia	Beijing	ERR067647	R	R	S	S	R	R	R	S
2393C	Casali et. al. Nat Genet. 2014 Mar;46(3):279-86	Russia	Beijing	ERR229966	R	R	R	S	R	R	R	U
2013P	Casali et. al. Nat Genet. 2014 Mar;46(3):279-86	Russia	lineage4	ERR144613	S	S	S	S	S	F	S	S
1732J	Casali et. al. Nat Genet. 2014 Mar;46(3):279-86	Russia	Ural	ERR137270	S	S	S	S	S	S	S	S
2642Y	Casali et. al. Nat Genet. 2014 Mar;46(3):279-86	Russia	lineage4	ERR234699	R	S	S	R	R	S	S	S
1814Y	Casali et. al. Nat Genet. 2014 Mar;46(3):279-86	Russia	Ural	ERR144555	S	S	S	S	S	S	S	S
2319X	Casali et. al. Nat Genet. 2014 Mar;46(3):279-86	Russia	Beijing	ERR227981	R	R	S	R	R	R	R	R
1678A	Casali et. al. Nat Genet. 2014 Mar;46(3):279-86	Russia	Beijing	ERR108510	R	R	S	R	R	R	R	R
0529B	Casali et. al. Nat Genet. 2014 Mar;46(3):279-86	Russia	Haarlem	ERR133878	R	R	R	R	R	R	R	R
2378L	Casali et. al. Nat Genet. 2014 Mar;46(3):279-86	Russia	Ural	ERR229981	S	S	S	S	S	S	S	S
2146J	Casali et. al. Nat Genet. 2014 Mar;46(3):279-86	Russia	LAM	ERR158595	R	R	S	S	R	R	S	S
2564N	Casali et. al. Nat Genet. 2014 Mar;46(3):279-86	Russia	Beijing	ERR234668	R	R	R	R	R	R	U	R
2325D	Casali et. al. Nat Genet. 2014 Mar;46(3):279-86	Russia	Beijing	ERR228013	R	R	R	S	R	R	R	U
1047P	Casali et. al. Nat Genet. 2014 Mar;46(3):279-86	Russia	Beijing	ERR108492	S	S	S	S	S	S	S	S
1540A	Casali et. al. Nat Genet. 2014 Mar;46(3):279-86	Russia	LAM	ERR108451	S	S	S	S	U	S	S	S
0844T	Casali et. al. Nat Genet. 2014 Mar;46(3):279-86	Russia	Beijing	ERR067663	R	R	R	S	R	R	R	U
0691C	Casali et. al. Nat Genet. 2014 Mar;46(3):279-86	Russia	lineage4	ERR133905	S	R	S	S	S	S	S	S
0824X	Casali et. al. Nat Genet. 2014 Mar;46(3):279-86	Russia	Beijing	ERR133951	R	R	R	R	R	R	R	F
2140C	Casali et. al. Nat Genet. 2014 Mar;46(3):279-86	Russia	Beijing	ERR158592	R	R	S	S	R	R	R	U
2082P	Casali et. al. Nat Genet. 2014 Mar;46(3):279-86	Russia	Beijing	ERR158570	R	R	S	S	R	R	S	S
2129Q	Casali et. al. Nat Genet. 2014 Mar;46(3):279-86	Russia	Beijing	ERR158587	R	R	R	R	R	R	R	R
0851B	Casali et. al. Nat Genet. 2014 Mar;46(3):279-86	Russia	Ural	ERR067636	R	S	S	R	U	S	S	S
2028F	Casali et. al. Nat Genet. 2014 Mar;46(3):279-86	Russia	Beijing	ERR144619	R	R	R	S	R	R	R	R
2202V	Casali et. al. Nat Genet. 2014 Mar;46(3):279-86	Russia	Haarlem	ERR228008	S	S	S	S	S	S	S	S
2011M	Casali et. al. Nat Genet. 2014 Mar;46(3):279-86	Russia	Beijing	ERR144611	R	R	S	S	R	R	R	U
0082Q	Casali et. al. Nat Genet. 2014 Mar;46(3):279-86	Russia	Beijing	ERR234581	S	S	S	S	S	S	S	S
0235G	Casali et. al. Nat Genet. 2014 Mar;46(3):279-86	Russia	Haarlem	ERR133822	S	R	S	R	S	S	S	S
1830Q	Casali et. al. Nat Genet. 2014 Mar;46(3):279-86	Russia	Beijing	ERR144563	R	R	R	S	R	R	R	S
0336R	Casali et. al. Nat Genet. 2014 Mar;46(3):279-86	Russia	Ural	ERR067606	S	S	S	S	S	S	S	S
2333M	Casali et. al. Nat Genet. 2014 Mar;46(3):279-86	Russia	lineage4	ERR228045	S	S	S	S	R	S	S	S
0032L	Casali et. al. Nat Genet. 2014 Mar;46(3):279-86	Russia	Beijing	ERR067600	R	S	S	S	R	S	S	S
0913T	Casali et. al. Nat Genet. 2014 Mar;46(3):279-86	Russia	Beijing	ERR067672	R	R	S	S	R	R	R	S
2338S	Casali et. al. Nat Genet. 2014 Mar;46(3):279-86	Russia	Beijing	ERR229923	R	R	R	S	R	R	R	U
2208B	Casali et. al. Nat Genet. 2014 Mar;46(3):279-86	Russia	Beijing	ERR228032	R	R	S	S	R	R	R	S
0724N	Casali et. al. Nat Genet. 2014 Mar;46(3):279-86	Russia	Ural	ERR108456	S	S	S	S	S	S	S	S
2516L	Casali et. al. Nat Genet. 2014 Mar;46(3):279-86	Russia	Beijing	ERR229994	R	R	S	S	R	R	R	U
2517M	Casali et. al. Nat Genet. 2014 Mar;46(3):279-86	Russia	Ural	ERR230002	S	S	S	S	S	S	S	S
0031K	Casali et. al. Nat Genet. 2014 Mar;46(3):279-86	Russia	Beijing	ERR158611	R	R	R	S	R	R	R	U
0988A	Casali et. al. Nat Genet. 2014 Mar;46(3):279-86	Russia	Haarlem	ERR108480	R	R	R	R	R	R	R	R
1615G	Casali et. al. Nat Genet. 2014 Mar;46(3):279-86	Russia	LAM	ERR067683	R	S	S	S	R	S	R	S
1128C	Casali et. al. Nat Genet. 2014 Mar;46(3):279-86	Russia	Beijing	ERR108495	R	R	R	S	R	R	R	S
2531C	Casali et. al. Nat Genet. 2014 Mar;46(3):279-86	Russia	Beijing	ERR234657	R	R	R	R	R	R	R	U
0085T	Casali et. al. Nat Genet. 2014 Mar;46(3):279-86	Russia	Beijing	ERR234589	R	R	R	S	R	R	R	U
2178T	Casali et. al. Nat Genet. 2014 Mar;46(3):279-86	Russia	LAM	ERR228031	S	S	S	S	U	S	S	S
2334N	Casali et. al. Nat Genet. 2014 Mar;46(3):279-86	Russia	Haarlem	ERR228053	S	S	S	S	S	U	S	S
0414B	Casali et. al. Nat Genet. 2014 Mar;46(3):279-86	Russia	Beijing	ERR067743	S	S	S	S	S	S	S	S
0584L	Casali et. al. Nat Genet. 2014 Mar;46(3):279-86	Russia	Beijing	ERR067604	S	S	S	R	S	S	U	S
0516M	Casali et. al. Nat Genet. 2014 Mar;46(3):279-86	Russia	LAM	ERR133872	S	S	S	S	R	S	S	S
1904W	Casali et. al. Nat Genet. 2014 Mar;46(3):279-86	Russia	Beijing	ERR144595	R	R	R	R	R	R	R	R
1593H	Casali et. al. Nat Genet. 2014 Mar;46(3):279-86	Russia	Beijing	ERR108433	S	S	S	S	S	S	S	S
0574A	Casali et. al. Nat Genet. 2014 Mar;46(3):279-86	Russia	Beijing	ERR234640	S	S	S	S	S	S	S	S
0314S	Casali et. al. Nat Genet. 2014 Mar;46(3):279-86	Russia	Ural	ERR067609	R	R	S	S	R	R	F	S
0721K	Casali et. al. Nat Genet. 2014 Mar;46(3):279-86	Russia	Beijing	ERR133914	R	R	R	R	R	S	R	S
0716E	Casali et. al. Nat Genet. 2014 Mar;46(3):279-86	Russia	Beijing	ERR108455	R	R	S	R	R	R	R	R
0076J	Casali et. al. Nat Genet. 2014 Mar;46(3):279-86	Russia	Beijing	ERR234565	R	R	S	S	R	R	R	U
0437B	Casali et. al. Nat Genet. 2014 Mar;46(3):279-86	Russia	LAM	ERR067727	S	S	S	S	S	S	S	S
0845V	Casali et. al. Nat Genet. 2014 Mar;46(3):279-86	Russia	LAM	ERR067664	S	S	S	S	F	S	S	S
1052V	Casali et. al. Nat Genet. 2014 Mar;46(3):279-86	Russia	Beijing	ERR108421	R	R	R	R	R	R	R	R
0180X	Casali et. al. Nat Genet. 2014 Mar;46(3):279-86	Russia	Beijing	ERR067581	R	R	R	R	R	R	R	R
0592V	Casali et. al. Nat Genet. 2014 Mar;46(3):279-86	Russia	Beijing	ERR133891	S	S	S	S	S	S	S	S
0123K	Casali et. al. Nat Genet. 2014 Mar;46(3):279-86	Russia	lineage4	ERR133803	R	R	S	S	S	S	S	S
2001B	Casali et. al. Nat Genet. 2014 Mar;46(3):279-86	Russia	Ural	ERR144605	R	S	S	S	R	S	U	S
2279D	Casali et. al. Nat Genet. 2014 Mar;46(3):279-86	Russia	Beijing	ERR227979	S	S	S	S	S	S	S	S
2225V	Casali et. al. Nat Genet. 2014 Mar;46(3):279-86	Russia	Ural	ERR228001	R	R	S	R	R	R	R	R
2053H	Casali et. al. Nat Genet. 2014 Mar;46(3):279-86	Russia	Beijing	ERR228046	R	R	S	S	R	R	R	U
0825Y	Casali et. al. Nat Genet. 2014 Mar;46(3):279-86	Russia	Ural	ERR133952	S	S	S	S	S	S	S	S
1039F	Casali et. al. Nat Genet. 2014 Mar;46(3):279-86	Russia	Beijing	ERR108490	S	S	S	S	S	S	U	S
1654Z	Casali et. al. Nat Genet. 2014 Mar;46(3):279-86	Russia	Beijing	ERR137254	R	R	R	S	R	R	R	S
0533F	Casali et. al. Nat Genet. 2014 Mar;46(3):279-86	Russia	lineage4	ERR133881	S	R	S	S	S	S	S	S
1523G	Casali et. al. Nat Genet. 2014 Mar;46(3):279-86	Russia	Beijing	ERR117464	R	R	R	R	R	R	R	R
0963Y	Casali et. al. Nat Genet. 2014 Mar;46(3):279-86	Russia	LAM	ERR133977	R	S	S	S	R	S	S	S
0071D	Casali et. al. Nat Genet. 2014 Mar;46(3):279-86	Russia	Beijing	ERR108502	R	R	S	S	R	R	R	U
1371R	Casali et. al. Nat Genet. 2014 Mar;46(3):279-86	Russia	Beijing	ERR137221	R	S	S	S	R	S	S	S
2508C	Casali et. al. Nat Genet. 2014 Mar;46(3):279-86	Russia	Beijing	ERR229946	R	R	R	S	R	R	R	U
1711L	Casali et. al. Nat Genet. 2014 Mar;46(3):279-86	Russia	Haarlem	ERR137266	S	S	S	S	S	S	S	S
2051F	Casali et. al. Nat Genet. 2014 Mar;46(3):279-86	Russia	LAM	ERR144627	R	S	R	S	R	S	R	S
2135X	Casali et. al. Nat Genet. 2014 Mar;46(3):279-86	Russia	Beijing	ERR158590	R	R	R	S	R	F	F	F
0645C	Casali et. al. Nat Genet. 2014 Mar;46(3):279-86	Russia	Beijing	ERR234617	S	S	S	S	S	S	S	S
0464F	Casali et. al. Nat Genet. 2014 Mar;46(3):279-86	Russia	Haarlem	ERR234631	R	R	S	S	R	R	R	S
2309L	Casali et. al. Nat Genet. 2014 Mar;46(3):279-86	Russia	Beijing	ERR228004	S	S	S	S	S	S	U	S
0317W	Casali et. al. Nat Genet. 2014 Mar;46(3):279-86	Russia	Beijing	ERR067610	R	S	S	S	R	S	S	S
1467W	Casali et. al. Nat Genet. 2014 Mar;46(3):279-86	Russia	Beijing	ERR067704	R	R	R	S	R	R	R	U
0149N	Casali et. al. Nat Genet. 2014 Mar;46(3):279-86	Russia	Beijing	ERR133814	R	S	S	S	R	S	S	S
0163D	Casali et. al. Nat Genet. 2014 Mar;46(3):279-86	Russia	Beijing	ERR067576	R	R	R	S	R	R	R	U
2310M	Casali et. al. Nat Genet. 2014 Mar;46(3):279-86	Russia	Beijing	ERR228012	R	R	R	S	R	R	R	R
0603G	Casali et. al. Nat Genet. 2014 Mar;46(3):279-86	Russia	Beijing	ERR234569	R	S	S	S	R	S	S	S
1016F	Casali et. al. Nat Genet. 2014 Mar;46(3):279-86	Russia	Beijing	ERR133982	R	R	S	S	R	R	U	S
0462D	Casali et. al. Nat Genet. 2014 Mar;46(3):279-86	Russia	Beijing	ERR234623	R	R	S	S	R	R	R	U
0622C	Casali et. al. Nat Genet. 2014 Mar;46(3):279-86	Russia	Beijing	ERR234593	R	R	S	S	R	R	R	U
2252Z	Casali et. al. Nat Genet. 2014 Mar;46(3):279-86	Russia	Beijing	ERR228002	S	S	S	S	S	S	S	S
2538K	Casali et. al. Nat Genet. 2014 Mar;46(3):279-86	Russia	Beijing	ERR234663	S	S	S	S	S	S	S	S
2518N	Casali et. al. Nat Genet. 2014 Mar;46(3):279-86	Russia	Beijing	ERR230010	S	S	S	S	S	S	S	S
0776V	Casali et. al. Nat Genet. 2014 Mar;46(3):279-86	Russia	Beijing	ERR133932	S	S	S	S	S	S	S	S
0856G	Casali et. al. Nat Genet. 2014 Mar;46(3):279-86	Russia	Beijing	ERR067637	S	S	S	S	S	S	S	S
2593V	Casali et. al. Nat Genet. 2014 Mar;46(3):279-86	Russia	LAM	ERR234689	S	S	S	S	S	U	S	S
0837L	Casali et. al. Nat Genet. 2014 Mar;46(3):279-86	Russia	Beijing	ERR133955	R	S	S	S	R	S	U	S
0372F	Casali et. al. Nat Genet. 2014 Mar;46(3):279-86	Russia	Beijing	ERR067696	R	R	S	S	R	R	U	S
2079L	Casali et. al. Nat Genet. 2014 Mar;46(3):279-86	Russia	Beijing	ERR144635	R	R	R	R	R	R	R	R

0567S	Casali et. al. Nat Genet. 2014 Mar;46(3):279-86	Russia	lineage4	ERR067763	S	S	S	S	S	S	S	S
0338T	Casali et. al. Nat Genet. 2014 Mar;46(3):279-86	Russia	Beijing	ERR234622	R	R	R	R	R	R	R	U
1737P	Casali et. al. Nat Genet. 2014 Mar;46(3):279-86	Russia	Beijing	ERR137273	R	R	S	S	R	R	R	S
2370C	Casali et. al. Nat Genet. 2014 Mar;46(3):279-86	Russia	Haarlem	ERR229941	S	S	S	S	S	S	S	S
0611Q	Casali et. al. Nat Genet. 2014 Mar;46(3):279-86	Russia	Beijing	ERR067595	S	S	S	S	S	S	S	S
1821F	Casali et. al. Nat Genet. 2014 Mar;46(3):279-86	Russia	Beijing	ERR144560	R	R	R	R	R	R	R	R
0138B	Casali et. al. Nat Genet. 2014 Mar;46(3):279-86	Russia	Ural	ERR133812	R	S	S	S	R	R	U	S
1182L	Casali et. al. Nat Genet. 2014 Mar;46(3):279-86	Russia	lineage4	ERR137197	R	S	R	S	S	S	S	S
0846W	Casali et. al. Nat Genet. 2014 Mar;46(3):279-86	Russia	Beijing	ERR067665	R	R	R	R	R	R	R	U
0101L	Casali et. al. Nat Genet. 2014 Mar;46(3):279-86	Russia	LAM	ERR067601	S	S	S	S	S	S	S	S
2027E	Casali et. al. Nat Genet. 2014 Mar;46(3):279-86	Russia	Haarlem	ERR144618	R	S	S	S	F	S	S	S
2634P	Casali et. al. Nat Genet. 2014 Mar;46(3):279-86	Russia	lineage4	ERR234696	R	S	S	R	R	S	S	S
1066K	Casali et. al. Nat Genet. 2014 Mar;46(3):279-86	Russia	Beijing	ERR108426	R	R	R	S	R	R	R	S
1955B	Casali et. al. Nat Genet. 2014 Mar;46(3):279-86	Russia	Ural	ERR144604	R	S	S	S	R	S	S	S
0842R	Casali et. al. Nat Genet. 2014 Mar;46(3):279-86	Russia	Ural	ERR067661	R	S	S	S	R	S	R	S
1271H	Casali et. al. Nat Genet. 2014 Mar;46(3):279-86	Russia	Beijing	ERR137210	R	R	S	S	R	R	R	U
1532R	Casali et. al. Nat Genet. 2014 Mar;46(3):279-86	Russia	Beijing	ERR228006	R	R	S	R	R	R	R	R
2015R	Casali et. al. Nat Genet. 2014 Mar;46(3):279-86	Russia	Beijing	ERR144614	S	S	S	S	S	S	S	S
1189T	Casali et. al. Nat Genet. 2014 Mar;46(3):279-86	Russia	LAM	ERR137200	S	S	S	S	S	S	S	S
1324Q	Casali et. al. Nat Genet. 2014 Mar;46(3):279-86	Russia	lineage4	ERR108504	S	S	R	S	S	S	S	S
0647E	Casali et. al. Nat Genet. 2014 Mar;46(3):279-86	Russia	Beijing	ERR234633	R	R	R	S	R	R	R	U
1048Q	Casali et. al. Nat Genet. 2014 Mar;46(3):279-86	Russia	Beijing	ERR133984	R	R	R	R	R	R	R	R
1469Y	Casali et. al. Nat Genet. 2014 Mar;46(3):279-86	Russia	Beijing	ERR067705	R	R	S	R	R	R	S	F
0987Z	Casali et. al. Nat Genet. 2014 Mar;46(3):279-86	Russia	Beijing	ERR108479	S	S	S	S	S	S	S	S
2163C	Casali et. al. Nat Genet. 2014 Mar;46(3):279-86	Russia	Beijing	ERR227975	R	R	S	S	R	R	S	S
2345A	Casali et. al. Nat Genet. 2014 Mar;46(3):279-86	Russia	Ural	ERR229979	S	S	S	S	S	S	S	S
2631L	Casali et. al. Nat Genet. 2014 Mar;46(3):279-86	Russia	Delhi	ERR234695	S	S	S	S	S	S	S	S
1738Q	Casali et. al. Nat Genet. 2014 Mar;46(3):279-86	Russia	Ural	ERR137274	R	R	S	S	R	R	S	S
0596Z	Casali et. al. Nat Genet. 2014 Mar;46(3):279-86	Russia	lineage4	ERR133893	S	S	S	S	S	S	S	S
0131T	Casali et. al. Nat Genet. 2014 Mar;46(3):279-86	Russia	lineage4	ERR133808	S	S	S	S	S	S	S	S
2267Q	Casali et. al. Nat Genet. 2014 Mar;46(3):279-86	Russia	lineage4	ERR228026	R	S	S	S	R	S	S	S
2023A	Casali et. al. Nat Genet. 2014 Mar;46(3):279-86	Russia	Beijing	ERR144616	S	S	S	S	S	S	S	S
0666A	Casali et. al. Nat Genet. 2014 Mar;46(3):279-86	Russia	Beijing	ERR234618	R	R	R	S	R	R	R	U
0506B	Casali et. al. Nat Genet. 2014 Mar;46(3):279-86	Russia	Beijing	ERR133871	S	S	S	S	S	S	U	S
1188S	Casali et. al. Nat Genet. 2014 Mar;46(3):279-86	Russia	lineage4	ERR137199	R	S	S	S	F	S	F	S
1509R	Casali et. al. Nat Genet. 2014 Mar;46(3):279-86	Russia	Beijing	ERR117463	R	R	S	S	R	R	U	S
2527Y	Casali et. al. Nat Genet. 2014 Mar;46(3):279-86	Russia	Beijing	ERR234655	R	S	S	S	R	S	S	S
2361S	Casali et. al. Nat Genet. 2014 Mar;46(3):279-86	Russia	Ural	ERR229972	S	S	S	S	S	S	S	S
2088W	Casali et. al. Nat Genet. 2014 Mar;46(3):279-86	Russia	Beijing	ERR158575	S	S	S	S	S	S	S	S
1653Y	Casali et. al. Nat Genet. 2014 Mar;46(3):279-86	Russia	Beijing	ERR137253	S	S	S	S	S	S	S	S
2039S	Casali et. al. Nat Genet. 2014 Mar;46(3):279-86	Russia	Beijing	ERR144623	R	R	S	S	R	R	R	R
1071Q	Casali et. al. Nat Genet. 2014 Mar;46(3):279-86	Russia	Beijing	ERR108428	R	R	R	S	R	R	R	R
0518P	Casali et. al. Nat Genet. 2014 Mar;46(3):279-86	Russia	Beijing	ERR133873	R	R	S	R	R	R	R	R
1503K	Casali et. al. Nat Genet. 2014 Mar;46(3):279-86	Russia	lineage4	ERR137238	S	S	S	S	S	S	S	S
0434Y	Casali et. al. Nat Genet. 2014 Mar;46(3):279-86	Russia	lineage4	ERR133864	S	S	S	S	S	S	S	S
1765V	Casali et. al. Nat Genet. 2014 Mar;46(3):279-86	Russia	Beijing	ERR137283	R	R	S	S	R	R	R	U
0053J	Casali et. al. Nat Genet. 2014 Mar;46(3):279-86	Russia	Beijing	ERR067620	R	R	R	S	R	R	R	U
1892H	Casali et. al. Nat Genet. 2014 Mar;46(3):279-86	Russia	Beijing	ERR144590	R	R	S	S	R	R	R	U
2472N	Casali et. al. Nat Genet. 2014 Mar;46(3):279-86	Russia	Beijing	ERR230007	R	R	R	S	R	S	R	S
0389Z	Casali et. al. Nat Genet. 2014 Mar;46(3):279-86	Russia	Beijing	ERR133854	R	R	R	S	R	R	R	U
0977N	Casali et. al. Nat Genet. 2014 Mar;46(3):279-86	Russia	Haarlem	ERR108474	R	R	S	R	R	R	R	R
1414N	Casali et. al. Nat Genet. 2014 Mar;46(3):279-86	Russia	Beijing	ERR137227	R	S	S	S	R	S	S	S
2581G	Casali et. al. Nat Genet. 2014 Mar;46(3):279-86	Russia	Beijing	ERR234683	R	S	S	S	R	S	S	S
1604V	Casali et. al. Nat Genet. 2014 Mar;46(3):279-86	Russia	LAM	ERR067716	R	S	S	S	S	S	S	S
2336Q	Casali et. al. Nat Genet. 2014 Mar;46(3):279-86	Russia	LAM	ERR228069	S	S	S	S	S	S	S	S
2174P	Casali et. al. Nat Genet. 2014 Mar;46(3):279-86	Russia	Beijing	ERR228007	S	S	S	S	S	S	S	S
0664Y	Casali et. al. Nat Genet. 2014 Mar;46(3):279-86	Russia	Beijing	ERR234602	n/a	R	S	S	R	R	R	U
1336D	Casali et. al. Nat Genet. 2014 Mar;46(3):279-86	Russia	Ural	ERR137214	S	S	S	S	S	S	U	S
0495P	Casali et. al. Nat Genet. 2014 Mar;46(3):279-86	Russia	Beijing	ERR234576	R	R	R	S	R	R	R	U
2348D	Casali et. al. Nat Genet. 2014 Mar;46(3):279-86	Russia	Beijing	ERR229995	R	R	S	S	R	R	U	S
1802K	Casali et. al. Nat Genet. 2014 Mar;46(3):279-86	Russia	Beijing	ERR144551	R	R	R	S	R	R	R	U
0190H	Casali et. al. Nat Genet. 2014 Mar;46(3):279-86	Russia	Beijing	ERR067722	R	R	R	S	R	R	R	R
2327F	Casali et. al. Nat Genet. 2014 Mar;46(3):279-86	Russia	Beijing	ERR228029	R	R	S	S	R	R	R	U
2365X	Casali et. al. Nat Genet. 2014 Mar;46(3):279-86	Russia	Beijing	ERR230004	S	S	S	S	S	U	S	S
0475S	Casali et. al. Nat Genet. 2014 Mar;46(3):279-86	Russia	Beijing	ERR067754	R	R	S	S	R	R	R	U
0756Y	Casali et. al. Nat Genet. 2014 Mar;46(3):279-86	Russia	Beijing	ERR133925	S	S	S	S	S	S	S	S
0048D	Casali et. al. Nat Genet. 2014 Mar;46(3):279-86	Russia	Beijing	ERR133799	R	S	S	S	R	S	R	S
1558V	Casali et. al. Nat Genet. 2014 Mar;46(3):279-86	Russia	Beijing	ERR067714	R	R	R	R	R	R	R	R
1530P	Casali et. al. Nat Genet. 2014 Mar;46(3):279-86	Russia	Beijing	ERR108450	R	R	R	R	R	R	R	R
1237W	Casali et. al. Nat Genet. 2014 Mar;46(3):279-86	Russia	Beijing	ERR137205	R	S	R	S	S	S	S	S
0305H	Casali et. al. Nat Genet. 2014 Mar;46(3):279-86	Russia	lineage4	ERR133841	R	S	S	S	F	S	S	S
2266P	Casali et. al. Nat Genet. 2014 Mar;46(3):279-86	Russia	Beijing	ERR228018	S	S	S	S	S	S	U	S
0029H	Casali et. al. Nat Genet. 2014 Mar;46(3):279-86	Russia	Ural	ERR067615	R	R	S	R	R	R	R	S
1422X	Casali et. al. Nat Genet. 2014 Mar;46(3):279-86	Russia	Beijing	ERR137229	R	R	R	R	R	R	R	R
2034M	Casali et. al. Nat Genet. 2014 Mar;46(3):279-86	Russia	LAM	ERR144621	S	S	S	R	S	S	S	S
2586M	Casali et. al. Nat Genet. 2014 Mar;46(3):279-86	Russia	LAM	ERR234685	S	S	S	S	S	S	S	S
1867F	Casali et. al. Nat Genet. 2014 Mar;46(3):279-86	Russia	Haarlem	ERR144578	S	S	S	S	S	S	S	S
2549X	Casali et. al. Nat Genet. 2014 Mar;46(3):279-86	Russia	Beijing	ERR234666	R	R	S	S	R	F	R	F
0460B	Casali et. al. Nat Genet. 2014 Mar;46(3):279-86	Russia	Beijing	ERR067630	R	R	R	S	R	R	F	R
0441F	Casali et. al. Nat Genet. 2014 Mar;46(3):279-86	Russia	Beijing	ERR234607	R	R	S	R	R	R	U	S
0182Z	Casali et. al. Nat Genet. 2014 Mar;46(3):279-86	Russia	Beijing	ERR067582	R	S	R	S	R	S	R	S
1690N	Casali et. al. Nat Genet. 2014 Mar;46(3):279-86	Russia	Beijing	ERR137257	R	R	S	S	R	R	R	S
0107S	Casali et. al. Nat Genet. 2014 Mar;46(3):279-86	Russia	Beijing	ERR133802	S	S	S	S	S	S	S	S
0701N	Casali et. al. Nat Genet. 2014 Mar;46(3):279-86	Russia	Beijing	ERR133908	R	R	R	R	R	R	R	R
0875C	Casali et. al. Nat Genet. 2014 Mar;46(3):279-86	Russia	Haarlem	ERR133961	R	S	S	S	R	S	S	S
0765H	Casali et. al. Nat Genet. 2014 Mar;46(3):279-86	Russia	lineage4	ERR108468	S	S	S	S	S	S	S	S
2466G	Casali et. al. Nat Genet. 2014 Mar;46(3):279-86	Russia	lineage4	ERR229991	S	S	S	S	S	S	S	S
1824J	Casali et. al. Nat Genet. 2014 Mar;46(3):279-86	Russia	Beijing	ERR144561	R	R	S	S	R	R	R	U
0087W	Casali et. al. Nat Genet. 2014 Mar;46(3):279-86	Russia	Beijing	ERR234597	R	R	S	S	R	R	R	S
1679B	Casali et. al. Nat Genet. 2014 Mar;46(3):279-86	Russia	Beijing	ERR108511	R	R	S	S	R	R	R	S
2083Q	Casali et. al. Nat Genet. 2014 Mar;46(3):279-86	Russia	lineage4	ERR158571	S	S	S	S	S	S	S	S
2601D	Casali et. al. Nat Genet. 2014 Mar;46(3):279-86	Russia	Beijing	ERR234692	R	R	S	S	R	R	R	S
0484C	Casali et. al. Nat Genet. 2014 Mar;46(3):279-86	Russia	Beijing	ERR133868	R	R	S	S	R	R	R	U
1601R	Casali et. al. Nat Genet. 2014 Mar;46(3):279-86	Russia	Ural	ERR108437	S	S	S	S	S	S	S	S
0113Z	Casali et. al. Nat Genet. 2014 Mar;46(3):279-86	Russia	lineage4	ERR067602	R	S	S	R	S	S	S	S
0620A	Casali et. al. Nat Genet. 2014 Mar;46(3):279-86	Russia	Haarlem	ERR067734	S	S	S	S	S	S	S	S
1816A	Casali et. al. Nat Genet. 2014 Mar;46(3):279-86	Russia	lineage4	ERR144557	S	S	S	S	S	S	S	S
2349E	Casali et. al. Nat Genet. 2014 Mar;46(3):279-86	Russia	Haarlem	ERR230003	R	R	S	S	R	R	R	S
1868G	Casali et. al. Nat Genet. 2014 Mar;46(3):279-86	Russia	Beijing	ERR144579	R	R	R	S	R	R	R	S
2372E	Casali et. al. Nat Genet. 2014 Mar;46(3):279-86	Russia	Beijing	ERR229957	R	S	S	S	R	S	S	S
0749Q	Casali et. al. Nat Genet. 2014 Mar;46(3):279-86	Russia	LAM	ERR133920	S	S	S	S	S	S	S	S
1162P	Casali et. al. Nat Genet. 2014 Mar;46(3):279-86	Russia	Beijing	ERR137192	R	R	R	R	R	R	R	R
1774E	Casali et. al. Nat Genet. 2014 Mar;46(3):279-86	Russia	lineage4	ERR144546	S	R	S	S	S	S	S	S
0836K	Casali et. al. Nat Genet. 2014 Mar;46(3):279-86	Russia	Ural	ERR067657	R	R	S	S	R	R	S	S
1471A	Casali et. al. Nat Genet. 2014 Mar;46(3):279-86	Russia	Beijing	ERR067707	R	R	R	S	R	R	F	S
2567R	Casali et. al. Nat Genet. 2014 Mar;46(3):279-86	Russia	Beijing	ERR234671	R	R	S	R	R	R	R	R
0823W	Casali et. al. Nat Genet. 2014 Mar;46(3):279-86	Russia	Beijing	ERR133950	S	S	S	S	S	S	S	S
2537J	Casali et. al. Nat Genet. 2014 Mar;46(3):279-86	Russia	Beijing	ERR234662	R	S	R	S	R	S	R	S
0571X	Casali et. al. Nat Genet. 2014 Mar;46(3):279-86	Russia	Beijing	ERR067765	R	R	S	S	R	R	F	F
0864Q	Casali et. al. Nat Genet. 2014 Mar;46(3):279-86	Russia	Beijing	ERR108439	R	R	R	S	R	R	R	F
1869H	Casali et. al. Nat Genet. 2014 Mar;46(3):279-86	Russia	Beijing	ERR144580	R	S	S	S	R	S	R	S
0041W	Casali et. al. Nat Genet. 2014 Mar;46(3):279-86	Russia	Beijing	ERR108515	R	R	S	S	R	R	R	R
0370D	Casali et. al. Nat Genet. 2014 Mar;46(3):279-86	Russia	Beijing	ERR133852	S	R	S	S	R	R	R	S
2297Y	Casali et. al. Nat Genet. 2014 Mar;46(3):279-86	Russia	Beijing	ERR228059	R	R	R	S	R	R	R	S
0821T	Casali et. al. Nat Genet. 2014 Mar;46(3):279-86	Russia	Beijing	ERR133948	S	S	S	S	S	S	S	S
7403-11	Serbia	Serbia	Tur	ERR2041785	R	R	R	R	R	R	R	R

7388-11	Serbia	Serbia	Haarlem	ERR2041771	R	R	S	S	U	R	S	S
9942-13	Serbia	Serbia	Beijing	ERR2200148	R	R	S	R	R	R	S	R
113-14	Serbia	Serbia	Tur	ERR2041688	R	R	R	R	R	R	R	U
7391-11	Serbia	Serbia	Haarlem	ERR2041773	R	R	S	S	R	R	S	S
7398-11	Serbia	Serbia	Tur	ERR2041780	R	R	R	R	R	R	R	R
6878-08	Serbia	Serbia	Haarlem	ERR2041740 ER	R	R	S	S	R	R	R	U
3908-12	Serbia	Serbia	lineage4	ERR2041696	R	R	S	S	U	R	S	S
6141-09	Serbia	Serbia	Tur	ERR2041734	R	R	R	R	R	R	R	R
112-13	Serbia	Serbia	Tur	ERR2041686 ER	R	R	n/a	R	R	R	R	S
3918-12	Serbia	Serbia	Tur	ERR2041709	R	R	R	R	R	R	R	U
7404-11	Serbia	Serbia	Tur	ERR2041786	R	R	R	R	R	R	R	R
6884-08	Serbia	Serbia	Tur	ERR2041746	R	R	R	R	R	R	R	F
6134-09	Serbia	Serbia	S-type	ERR2041727	R	R	R	R	R	R	U	R
7406-11	Serbia	Serbia	Haarlem	ERR2041788	R	R	S	R	U	R	U	R
6893-08	Serbia	Serbia	Tur	ERR2041753	R	R	R	S	R	F	R	S
7401-11	Serbia	Serbia	Beijing	ERR2041783	R	R	R	R	R	R	U	R
7400-11	Serbia	Serbia	lineage4	ERR2041782	R	R	R	R	R	R	R	R
6899-08	Serbia	Serbia	lineage4	ERR2041758	R	R	S	S	R	S	S	S
6881-08	Serbia	Serbia	Beijing	ERR2041744	R	R	S	R	R	R	U	R
6879-08	Serbia	Serbia	Beijing	ERR2041742	R	R	S	R	R	R	U	R
3916-12	Serbia	Serbia	Beijing	ERR2041705 ER	R	R	S	R	R	R	S	R
9936-13	Serbia	Serbia	lineage4	ERR2041794	R	R	S	S	R	R	U	S
9935-13	Serbia	Serbia	lineage4	ERR2041792 ER	R	R	S	S	R	R	S	S
109-13	Serbia	Serbia	Tur	ERR2041683	R	R	R	S	R	R	R	S
6127-09	Serbia	Serbia	lineage4	ERR2041721	R	R	R	R	R	R	R	R
6902-08	Serbia	Serbia	Beijing	ERR2041760	R	R	R	R	R	R	R	F
6910-08	Serbia	Serbia	Haarlem	ERR2041767	R	R	S	S	U	R	S	S
114-14	Serbia	Serbia	Tur	ERR2041689	R	R	R	R	R	R	R	R
6913-08	Serbia	Serbia	lineage4	ERR2041770	R	R	S	S	R	R	S	S
6897-08	Serbia	Serbia	Tur	ERR2041756	R	R	R	S	R	R	R	S
9944-13	Serbia	Serbia	Haarlem	ERR2041804	R	R	S	S	R	R	R	S
6906-08	Serbia	Serbia	Tur	ERR2041764	R	R	R	R	R	R	R	U
6892-08	Serbia	Serbia	lineage4	ERR2041752	R	R	R	S	R	R	R	S
6129-09	Serbia	Serbia	Tur	ERR2041723	R	R	R	S	R	R	R	S
9945-13	Serbia	Serbia	Ural	ERR2041805	R	R	S	S	R	R	S	S
6903-08	Serbia	Serbia	Haarlem	ERR2041761	R	R	R	S	R	R	R	S
9943-13	Serbia	Serbia	Haarlem	ERR2041803	R	R	R	S	R	R	R	S
6896-08	Serbia	Serbia	S-type	ERR2200092	R	R	R	S	U	R	F	S
110-13	Serbia	Serbia	lineage4	ERR2041684	R	R	R	S	R	R	R	S
3922-12	Serbia	Serbia	lineage4	ERR2041713 ER	R	R	R	R	R	R	R	R
6139-09	Serbia	Serbia	lineage4	ERR2041732	R	R	S	R	R	R	S	R
6140-09	Serbia	Serbia	Haarlem	ERR2041733	R	R	R	S	R	R	F	S
117-14	Serbia	Serbia	Beijing	ERR2041694 ER	R	R	n/a	R	R	R	S	R
6909-08	Serbia	Serbia	Tur	ERR2041766	R	R	R	S	R	R	R	U
7396-11	Serbia	Serbia	Tur	ERR2041777	R	R	R	R	R	R	R	U
7393-11	Serbia	Serbia	S-type	ERR2041775	R	R	R	R	U	R	R	R
3917-12	Serbia	Serbia	lineage4	ERR2041707 ER	R	R	S	R	R	R	R	R
6122-09	Serbia	Serbia	Tur	ERR2041718	R	R	R	S	R	R	R	U
6143-09	Serbia	Serbia	Tur	ERR2041736	R	R	R	S	R	R	R	S
6904-08	Serbia	Serbia	Tur	ERR2041762	R	R	R	R	R	R	R	R
111-13	Serbia	Serbia	Beijing	ERR2041685	R	R	R	S	R	R	S	R
7394-11	Serbia	Serbia	Haarlem	ERR2041776	R	R	S	S	R	R	S	S
6898-08	Serbia	Serbia	Haarlem	ERR2041757	R	R	R	R	R	R	R	R
9937-13	Serbia	Serbia	Tur	ERR2041795	R	R	R	S	R	R	R	U
6137-09	Serbia	Serbia	lineage4	ERR2041730	R	R	R	S	U	R	R	R
6885-08	Serbia	Serbia	S-type	ERR2041747	R	R	R	S	U	R	R	S
6124-09	Serbia	Serbia	Haarlem	ERR2041719	R	R	S	S	R	R	S	S
7392-11	Serbia	Serbia	S-type	ERR2041774	R	R	R	R	U	R	R	R
6145-09	Serbia	Serbia	Tur	ERR2041739	R	R	R	S	R	R	R	S
9933-13	Serbia	Serbia	Tur	ERR2041789	R	R	R	R	R	R	R	U
6136-09	Serbia	Serbia	Tur	ERR2041729	R	R	S	S	R	R	U	R
3924-12	Serbia	Serbia	Beijing	ERR2041715 ER	R	R	S	R	R	R	S	R
6888-08	Serbia	Serbia	Tur	ERR2041749	R	R	R	R	R	R	R	U
3914-12	Serbia	Serbia	lineage4	ERR2041701 ER	R	R	R	R	R	R	R	R
3911-12	Serbia	Serbia	Tur	ERR2041699 ER	R	R	R	R	R	R	R	R
6144-09	Serbia	Serbia	Tur	ERR2041737 ER	R	R	R	R	R	R	R	R
6907-08	Serbia	Serbia	Haarlem	ERR2041765	R	R	S	R	U	R	R	R
6883-08	Serbia	Serbia	S-type	ERR2041745	R	R	S	S	U	R	F	S
6142-09	Serbia	Serbia	lineage4	ERR2041735	R	R	R	S	R	R	R	S
3925-12	Serbia	Serbia	Tur	ERR2199914	R	R	R	R	R	R	R	U
116-14	Serbia	Serbia	Haarlem	ERR2041692 ER	R	R	R	S	R	R	R	S
6912-08	Serbia	Serbia	lineage4	ERR2041769	R	R	R	S	U	R	R	S
9940-13	Serbia	Serbia	Beijing	ERR2041799 ER	R	R	S	R	R	R	U	R
3919-12	Serbia	Serbia	lineage4	ERR2041710 ER	R	R	S	R	U	R	S	R
6138-09	Serbia	Serbia	Tur	ERR2041731	R	R	R	S	R	R	R	R
9934-13	Serbia	Serbia	Tur	ERR2041790 ER	R	R	R	R	R	R	R	R
6911-08	Serbia	Serbia	Beijing	ERR2041768	R	R	R	S	R	R	R	S
6890-08	Serbia	Serbia	Haarlem	ERR2041750	R	R	R	R	R	R	R	R
7389-11	Serbia	Serbia	Beijing	ERR2041772	R	R	S	R	R	R	S	S
115-14	Serbia	Serbia	Beijing	ERR2041690 ER	R	R	n/a	R	R	R	S	R
6900-08	Serbia	Serbia	Tur	ERR2041759	R	R	R	R	R	R	R	U
9939-13	Serbia	Serbia	Tur	ERR2041797 ER	R	R	R	S	R	R	R	U
6891-08	Serbia	Serbia	Haarlem	ERR2041751	R	R	S	S	R	R	S	S
7405-11	Serbia	Serbia	Beijing	ERR2041787	R	R	R	R	R	R	R	U
9941-13	Serbia	Serbia	lineage4	ERR2041801 ER	R	R	R	R	R	R	R	R
3920-12	Serbia	Serbia	Tur	ERR2041712	R	R	R	R	R	R	R	R
3915-12	Serbia	Serbia	Beijing	ERR2041703 ER	R	R	S	R	R	R	S	R
3926-12	Serbia	Serbia	Tur	ERR2041717	R	R	R	R	R	R	R	R
6146-09	Serbia	Serbia	S-type	ERR2200070	R	R	R	R	U	R	R	U
6126-09	Serbia	Serbia	Beijing	ERR2041720	R	R	R	R	R	R	R	F
7399-11	Serbia	Serbia	Beijing	ERR2041781	R	R	R	S	R	R	U	S
9938-13	Serbia	Serbia	Tur	ERR2041796	R	R	R	R	R	R	R	R
108-13	Serbia	Serbia	Ural	ERR2041682	R	R	S	S	R	R	S	S
6901-08	Serbia	Serbia	Tur	ERR2200093	R	R	R	S	R	R	R	U
6128-09	Serbia	Serbia	lineage4	ERR2041722	R	R	S	S	R	R	U	S
3910-12	Serbia	Serbia	Tur	ERR2041698	R	R	R	R	R	R	R	U
6894-08	Serbia	Serbia	Tur	ERR2041754	R	R	R	R	R	R	R	U
7402-11	Serbia	Serbia	S-type	ERR2041784	R	R	R	R	U	R	R	S
6905-08	Serbia	Serbia	Tur	ERR2041763	R	R	R	R	R	R	R	R
6880-08	Serbia	Serbia	Beijing	ERR2041743	R	R	R	S	R	R	R	S
6886-08	Serbia	Serbia	Tur	ERR2041748	R	R	R	R	R	R	R	U
6131-09	Serbia	Serbia	Haarlem	ERR2041724	R	R	S	S	R	R	S	S
6135-09	Serbia	Serbia	Haarlem	ERR2041728	R	R	S	R	R	R	R	R
6895-08	Serbia	Serbia	lineage4	ERR2041755	R	R	R	S	R	R	R	S
DG00508444_S17	South Africa	South Africa	S-type		S	S	S	S	S	S	S	S
BN00034291_S4	South Africa	South Africa	Beijing		S	S	S	S	S	S	S	S
TRL0113094_S1	South Africa	South Africa	LAM		S	R	S	S	S	R	S	S
BF01462185_S4	South Africa	South Africa	Delhi		S	S	S	S	S	U	S	S
TRL0113639_S16	South Africa	South Africa	Beijing		R	R	R	S	R	R	R	S
BN00045523_S24	South Africa	South Africa	LAM		S	S	S	S	S	S	S	S
CD00745523_S11	South Africa	South Africa	LAM		S	S	S	S	S	R	S	S
TRL0084661_S16	South Africa	South Africa	Beijing		R	R	R	R	R	R	R	R
BF01451694_S6	South Africa	South Africa	Beijing		S	S	S	S	S	S	S	S
CE01509524_S21	South Africa	South Africa	S-type		R	S	S	S	R	S	S	S
TRL0116714_S5	South Africa	South Africa	EAI		R	R	R	n/a	S	R	U	S
CE01680254_S21	South Africa	South Africa	X-type		S	S	S	S	S	S	S	S

TRL0112292_S17	South Africa	South Africa	Beijing	S	S	S	S	S	S	S	S
CE01514835_S29	South Africa	South Africa	Beijing	S	S	S	S	S	S	S	S
PK00709697_S19	South Africa	South Africa	Beijing	S	R	S	S	S	S	S	S
DH00744330_S26	South Africa	South Africa	lineage4	S	S	S	S	S	S	S	S
TRL0065060_S29	South Africa	South Africa	Beijing	R	R	S	S	R	R	R	S
BQ00030439_S1	South Africa	South Africa	lineage4	S	S	S	S	S	S	S	S
TRL0073861_S19	South Africa	South Africa	lineage4	R	R	S	S	R	R	S	S
CE01632288_S29	South Africa	South Africa	Beijing	S	S	S	S	S	S	S	S
DG00622723_S13	South Africa	South Africa	lineage4	S	S	S	S	S	S	S	S
TRL0112884_S21	South Africa	South Africa	Beijing	S	R	S	S	S	R	S	S
ED00890639_S25	South Africa	South Africa	LAM	S	S	S	S	S	S	S	S
CG01628784_S23	South Africa	South Africa	Beijing	S	S	S	S	S	S	S	S
CE01835353_S27	South Africa	South Africa	Beijing	S	S	S	S	S	S	S	S
JG00244947_S31	South Africa	South Africa	LAM	R	R	S	R	R	R	S	R
PK00561581_S9	South Africa	South Africa	EAI	R	R	R	R	R	R	R	R
BT00031974_S18	South Africa	South Africa	S-type	S	S	S	S	S	S	S	S
TRL0116598_S7	South Africa	South Africa	X-type	n/a	R	R	n/a	R	R	R	R
CG01461742_S26	South Africa	South Africa	LAM	S	S	S	S	S	S	S	S
TRL0115026_S13	South Africa	South Africa	Haarlem	n/a	R	R	n/a	R	R	R	R
BK00081902_S20	South Africa	South Africa	LAM	S	S	S	S	S	S	S	S
DH00730366_S3	South Africa	South Africa	Haarlem	S	S	S	S	S	S	S	S
CD00831328_S31	South Africa	South Africa	Beijing	S	S	S	S	S	S	S	S
BN00045759_S27	South Africa	South Africa	S-type	S	S	S	S	S	S	S	S
BQ00026670_S17	South Africa	South Africa	Delhi	S	S	S	S	S	S	S	S
DH00680834_S14	South Africa	South Africa	lineage4	S	S	S	S	S	F	S	S
BF01503182_S11	South Africa	South Africa	LAM	S	S	n/a	S	S	S	S	S
TRL0084676_S26	South Africa	South Africa	lineage4	S	R	S	S	R	R	S	S
TRL0116928_S14	South Africa	South Africa	Beijing	n/a	R	R	n/a	R	R	R	R
BF01298610_S12	South Africa	South Africa	Haarlem	S	S	S	S	S	S	S	S
BE01109226_S13	South Africa	South Africa	Beijing	S	S	S	S	S	U	S	S
TRL0113054_S22	South Africa	South Africa	EAI	R	R	S	R	R	R	R	R
DG00528302_S16	South Africa	South Africa	LAM	S	S	S	S	S	F	S	S
BN00045790_S27	South Africa	South Africa	Beijing	S	S	S	S	S	S	S	S
BF01344980_S16	South Africa	South Africa	Beijing	S	S	S	S	S	S	S	S
BN00045778_S6	South Africa	South Africa	Beijing	S	S	S	S	S	S	S	S
TRL0115821_S8	South Africa	South Africa	lineage4	n/a	R	R	n/a	R	R	R	R
TRL0117023_S17	South Africa	South Africa	LAM	n/a	R	R	n/a	R	R	R	R
DH00540274_S14	South Africa	South Africa	X-type	S	S	S	S	S	S	F	S
PF00076589_S28	South Africa	South Africa	S-type	R	R	S	S	R	R	S	R
CE01896645_S29	South Africa	South Africa	Beijing	S	S	S	S	U	R	S	S
BT00030681_S18	South Africa	South Africa	LAM	S	S	S	S	S	S	S	S
BK00091914_S28	South Africa	South Africa	LAM	S	S	S	S	S	S	S	S
TRL0115398_S12	South Africa	South Africa	Beijing	n/a	R	R	n/a	S	R	R	S
DG00497215_S8	South Africa	South Africa	Delhi	S	S	S	R	S	S	S	R
DG00550733_S19	South Africa	South Africa	lineage4	S	S	S	S	S	S	S	S
FD00249184_S14	South Africa	South Africa	Delhi	S	S	S	S	S	S	S	S
TRL0113494_S3	South Africa	South Africa	EAI	R	R	R	R	R	R	R	R
FD00210395_S25	South Africa	South Africa	lineage4	S	S	S	S	S	S	S	S
TRL0105195_S13	South Africa	South Africa	Haarlem	R	R	R	n/a	R	R	R	R
TRL0040091_S29	South Africa	South Africa	lineage4	R	R	S	S	R	R	R	S
BQ00025088_S25	South Africa	South Africa	S-type	S	S	S	S	S	S	S	S
DH00713162_S9	South Africa	South Africa	S-type	S	S	S	S	S	S	S	S
BK00078535_S15	South Africa	South Africa	S-type	R	S	S	S	R	S	R	S
BE01194801_S10	South Africa	South Africa	LAM	S	S	S	S	S	S	S	S
FB00504841_S7	South Africa	South Africa	Beijing	S	S	S	S	S	S	S	S
CD00879967_S14	South Africa	South Africa	LAM	S	S	S	S	S	S	U	S
TRL0114585_S23	South Africa	South Africa	Beijing	n/a	R	R	n/a	R	R	R	R
TRL0083443_S9	South Africa	South Africa	Beijing	S	S	S	S	S	S	S	F
TRL0115921_S9	South Africa	South Africa	LAM	n/a	R	R	n/a	R	R	R	R
DH00743574_S5	South Africa	South Africa	S-type	S	S	S	S	S	S	S	S
BE01452991_S29	South Africa	South Africa	lineage4	S	S	S	S	S	S	U	S
TRL0113325_S4	South Africa	South Africa	Beijing	S	R	S	S	S	R	S	S
BF01304848_S20	South Africa	South Africa	Beijing	S	S	S	S	S	S	S	S
BK00085627_S24	South Africa	South Africa	Haarlem	S	S	S	S	S	S	S	S
DH00593014_S29	South Africa	South Africa	lineage4	S	S	S	S	S	S	S	S
TRL0114239_S21	South Africa	South Africa	Haarlem	R	R	R	R	R	R	R	R
EB00175368_S6	South Africa	South Africa	S-type	S	S	S	S	S	S	S	S
DG00532210_S10	South Africa	South Africa	LAM	S	S	S	S	S	S	S	S
ED00880062_S20	South Africa	South Africa	S-type	R	S	S	R	R	R	R	R
TRL0083502_S35	South Africa	South Africa	Beijing	S	S	S	S	S	S	S	S
DH00637914_S7	South Africa	South Africa	S-type	S	S	S	S	S	S	S	S
DG00589680_S7	South Africa	South Africa	Delhi	S	S	S	S	S	S	S	S
TRL0112979_S20	South Africa	South Africa	LAM	R	R	S	S	R	R	S	S
CE01521393_S1	South Africa	South Africa	Beijing	S	S	S	S	S	S	S	S
CE01890755_S13	South Africa	South Africa	Beijing	S	S	S	n/a	S	F	S	S
CE01428572_S2	South Africa	South Africa	lineage4	S	S	S	S	S	S	S	S
FB00459103_S16	South Africa	South Africa	LAM	S	S	S	S	S	S	S	S
DG00660292_S5	South Africa	South Africa	LAM	S	S	S	S	S	S	S	S
BE01391332_S20	South Africa	South Africa	LAM	S	R	S	S	S	R	U	S
EI00656767_S8	South Africa	South Africa	X-type	S	S	S	S	S	S	U	S
BT00031431_S7	South Africa	South Africa	LAM	S	S	S	S	U	S	S	S
DG00579942_S22	South Africa	South Africa	S-type	S	S	S	S	S	S	S	S
TRL0115159_S15	South Africa	South Africa	Haarlem	n/a	R	R	n/a	R	R	R	R
BR00089488_S21	South Africa	South Africa	Delhi	S	S	S	S	S	S	S	S
DG00538620_S17	South Africa	South Africa	Beijing	S	S	S	S	S	S	S	S
JA00176841_S1	South Africa	South Africa	lineage4	R	R	S	R	R	R	R	R
BF01381823_S11	South Africa	South Africa	X-type	S	S	S	S	S	S	S	S
FD00248236_S10	South Africa	South Africa	S-type	S	S	S	S	S	S	S	S
KF00065920_S19	South Africa	South Africa	LAM	S	R	S	R	S	S	S	S
BT00030319_S5	South Africa	South Africa	LAM	S	S	S	S	S	S	S	S
EB00165987_S16	South Africa	South Africa	Haarlem	S	S	S	S	S	S	S	S
BH00962940_S21	South Africa	South Africa	X-type	S	S	S	S	S	S	U	S
TRL0018609_S19	South Africa	South Africa	Beijing	R	R	S	S	R	R	S	S
TRL0114134_S8	South Africa	South Africa	Haarlem	R	R	S	R	R	R	S	R
PN00175023_S13	South Africa	South Africa	lineage4	S	S	S	S	S	S	S	S
BK00096329_S23	South Africa	South Africa	X-type	S	S	S	S	S	S	S	S
TRL0114469_S24	South Africa	South Africa	Beijing	n/a	R	R	n/a	R	R	R	R
BN00047410_S18	South Africa	South Africa	Beijing	S	S	S	S	S	S	S	S
EB00168504_S17	South Africa	South Africa	S-type	S	S	S	S	S	S	S	S
BF01076000_S23	South Africa	South Africa	lineage4	S	S	S	S	S	S	S	S
TRL0095895_S18	South Africa	South Africa	lineage4	S	S	S	S	S	S	S	S
TRL0113019_S9	South Africa	South Africa	lineage4	S	R	S	S	S	R	S	S
BD00550189_S2	South Africa	South Africa	S-type	S	S	S	S	S	S	S	S
DH00634094_S1	South Africa	South Africa	LAM	S	S	S	S	S	S	S	S
TRL0113198_S2	South Africa	South Africa	Haarlem	S	R	S	S	S	R	S	S
BR00078837_S17	South Africa	South Africa	Haarlem	S	S	S	S	S	S	S	S
PS00307966_S11	South Africa	South Africa	Beijing	S	R	S	n/a	S	R	S	S
PS00313772_S12	South Africa	South Africa	Beijing	R	R	S	S	R	R	R	R
PS00326134_S9	South Africa	South Africa	lineage4	R	R	S	S	R	R	S	S
CE01894895_S3	South Africa	South Africa	Beijing	S	S	S	S	S	S	S	S
TRL0113616_S14	South Africa	South Africa	Beijing	R	R	S	R	R	R	R	R
TRL0081287_S36	South Africa	South Africa	Beijing	R	R	R	R	F	S	F	F
BD00615651_S4	South Africa	South Africa	Beijing	S	S	S	S	S	S	S	S
BF01457868_S14	South Africa	South Africa	lineage4	S	S	S	S	F	F	S	S
QH00201945_S15	South Africa	South Africa	S-type	S	R	S	S	S	R	S	S
BN00041952_S22	South Africa	South Africa	LAM	S	S	S	S	S	S	S	S

DG00537228_S9	South Africa	South Africa	lineage4	S	S	S	S	S	S	S	S
BF01332263_S25	South Africa	South Africa	LAM	S	S	S	S	S	S	S	S
BN00041405_S21	South Africa	South Africa	LAM	S	S	S	S	S	S	S	S
BT00031500_S10	South Africa	South Africa	LAM	S	S	S	S	S	S	S	S
CE01938363_S31	South Africa	South Africa	LAM	S	S	S	S	S	S	S	S
TRL0116168_S18	South Africa	South Africa	Beijing	n/a	R	R	n/a	R	R	R	R
BF01338219_S2	South Africa	South Africa	LAM	S	S	S	S	U	S	S	S
BR00093395_S17	South Africa	South Africa	Beijing	S	S	S	S	S	S	S	S
EI00666603_S8	South Africa	South Africa	Beijing	S	S	S	S	S	S	S	S
BF01092541_S25	South Africa	South Africa	S-type	R	R	R	S	R	R	R	U
CD00934516_S20	South Africa	South Africa	Beijing	S	S	S	S	S	U	S	S
EI00603951_S24	South Africa	South Africa	LAM	S	S	S	S	S	S	S	S
DG00676352_S17	South Africa	South Africa	lineage4	S	R	R	S	S	R	S	S
TRL0044786_S19	South Africa	South Africa	Haarlem	R	R	S	R	R	R	S	R
JG00304736_S2	South Africa	South Africa	S-type	R	R	R	R	R	R	R	R
TRL0115004_S6	South Africa	South Africa	X-type	n/a	R	S	n/a	S	R	S	F
TRL0113493_S10	South Africa	South Africa	EAI	R	R	R	R	R	R	R	R
TRL0116865_S11	South Africa	South Africa	Beijing	n/a	R	R	n/a	R	R	R	R
EI00661011_S12	South Africa	South Africa	Beijing	S	S	S	S	S	S	S	S
TRL0091844_S5	South Africa	South Africa	EAI	R	R	R	R	R	R	R	R
BT00031455_S8	South Africa	South Africa	Delhi	S	S	S	S	S	S	S	S
DG00497195_S6	South Africa	South Africa	Delhi	S	S	S	S	F	S	S	S
DH00686932_S3	South Africa	South Africa	Beijing	R	R	S	S	F	F	F	F
DG00664475_S13	South Africa	South Africa	Beijing	S	S	S	S	S	S	S	S
CG01293502_S1	South Africa	South Africa	Beijing	S	S	S	S	S	S	S	S
RD00095112_S10	South Africa	South Africa	LAM	R	R	S	S	U	S	S	S
EI00608897_S32	South Africa	South Africa	Haarlem	S	S	S	S	S	S	S	S
BQ00025411_S6	South Africa	South Africa	S-type	S	S	S	S	S	S	S	S
DG00664485_S7	South Africa	South Africa	Delhi	R	S	S	R	R	S	S	U
FD00222433_S28	South Africa	South Africa	X-type	R	R	R	S	R	R	R	U
TRL0116219_S20	South Africa	South Africa	Beijing	n/a	R	R	n/a	R	R	R	S
DH00695092_S11	South Africa	South Africa	Haarlem	R	R	R	R	R	R	R	R
BR00088314_S16	South Africa	South Africa	S-type	R	R	R	R	R	R	R	R
PI00148593_S32	South Africa	South Africa	LAM	S	R	S	S	S	R	S	S
TRL0041018_S17	South Africa	South Africa	LAM	S	R	S	S	S	S	S	S
CD00809239_S32	South Africa	South Africa	Beijing	S	S	S	S	S	S	S	S
BF01127258_S30	South Africa	South Africa	S-type	S	S	S	S	S	S	S	S
CE01929749_S11	South Africa	South Africa	Beijing	S	S	S	S	S	S	S	S
BR00096737_S28	South Africa	South Africa	Beijing	S	S	S	n/a	S	S	S	S
BD00574429_S3	South Africa	South Africa	Beijing	S	S	S	S	S	S	S	S
BF01457882_S22	South Africa	South Africa	Beijing	S	S	S	S	S	S	S	S
BF01491838_S3	South Africa	South Africa	Haarlem	S	S	S	S	S	S	S	S
BN00040337_S32	South Africa	South Africa	Haarlem	S	S	S	S	S	S	S	S
DH00668828_S20	South Africa	South Africa	Beijing	S	S	S	S	S	S	S	S
QF00450839_S14	South Africa	South Africa	X-type	S	R	S	S	U	R	S	S
CG01570427_S22	South Africa	South Africa	Haarlem	S	S	S	S	S	S	S	S
BF01391370_S28	South Africa	South Africa	Beijing	S	S	S	S	S	S	S	S
GH00373068_S23	South Africa	South Africa	lineage4	S	S	S	S	S	S	S	S
BF01330892_S26	South Africa	South Africa	LAM	S	S	S	S	S	S	S	S
BF01227089_S16	South Africa	South Africa	Beijing	S	S	S	S	S	S	S	S
TRL0038616_S12	South Africa	South Africa	Beijing	R	R	S	S	R	R	S	S
TRL0113427_S13	South Africa	South Africa	Haarlem	R	R	S	R	R	R	S	R
BF01353408_S31	South Africa	South Africa	Haarlem	S	S	S	S	S	S	S	S
TRL0114845_S3	South Africa	South Africa	LAM	n/a	R	R	n/a	R	R	R	F
BF01491999_S23	South Africa	South Africa	S-type	S	S	S	S	S	S	S	S
BF01498045_S22	South Africa	South Africa	LAM	S	S	S	S	U	S	S	S
DG00646980_S4	South Africa	South Africa	Beijing	S	S	S	S	S	S	S	S
CD00958113_S26	South Africa	South Africa	X-type	S	S	S	S	S	S	S	S
CE01825547_S19	South Africa	South Africa	Beijing	S	S	S	S	S	S	S	S
BF01355993_S32	South Africa	South Africa	S-type	R	S	S	S	S	S	S	S
CD00862479_S16	South Africa	South Africa	LAM	R	R	R	R	R	R	R	R
TRL0073196_S25	South Africa	South Africa	X-type	R	R	S	R	R	R	R	R
BQ00030105_S31	South Africa	South Africa	LAM	S	S	S	S	S	S	S	S
DH00741625_S10	South Africa	South Africa	Beijing	S	S	S	n/a	S	S	S	S
BT00028734_S4	South Africa	South Africa	Beijing	S	S	S	S	F	S	S	S
CG01611734_S28	South Africa	South Africa	X-type	S	S	S	S	S	S	U	S
ED00927883_S7	South Africa	South Africa	Beijing	S	S	S	S	S	S	S	S
BT00033633_S21	South Africa	South Africa	Beijing	S	S	S	S	S	S	S	S
TRL0022765_S16	South Africa	South Africa	Beijing	R	R	S	R	R	R	R	R
CE01707414_S3	South Africa	South Africa	Beijing	S	S	S	S	S	S	S	S
DH00628091_S22	South Africa	South Africa	Haarlem	S	S	S	S	S	S	S	S
TRL0088085_S26	South Africa	South Africa	Beijing	R	R	R	S	R	F	R	R
BK00081681_S19	South Africa	South Africa	Beijing	S	S	S	S	S	S	S	S
GH00383070_S2	South Africa	South Africa	Beijing	S	S	S	S	S	S	S	S
ED00927899_S1	South Africa	South Africa	LAM	S	S	S	S	S	S	S	S
BF01491881_S14	South Africa	South Africa	Beijing	S	S	S	S	S	S	S	S
BT00031324_S6	South Africa	South Africa	Beijing	S	S	S	S	F	S	S	F
TRL0113347_S12	South Africa	South Africa	S-type	R	R	R	R	R	R	R	R
PO00232748_S14	South Africa	South Africa	LAM	S	S	S	S	S	S	S	S
DG00497214_S7	South Africa	South Africa	X-type	S	S	S	S	S	S	S	S
BH00935270_S22	South Africa	South Africa	Beijing	S	S	S	S	S	S	S	S
CG01283125_S32	South Africa	South Africa	EAI	S	S	S	S	S	U	S	S
BT00030782_S26	South Africa	South Africa	X-type	S	S	S	S	S	U	S	S
DG00674781_S19	South Africa	South Africa	LAM	S	S	S	S	U	S	S	S
DH00563988_S26	South Africa	South Africa	lineage4	S	S	S	S	S	S	S	S
BF01462180_S24	South Africa	South Africa	Beijing	S	S	S	S	S	S	S	S
TRL0115510_S14	South Africa	South Africa	LAM	n/a	R	R	n/a	R	R	R	R
CH00382796_S10	South Africa	South Africa	X-type	S	S	S	S	S	S	S	S
BF01165217_S2	South Africa	South Africa	LAM	S	S	S	S	S	S	S	S
TRL0115399_S7	South Africa	South Africa	Beijing	n/a	R	R	n/a	R	R	R	R
JG00421329_S17	South Africa	South Africa	lineage4	S	R	S	S	S	F	S	S
BK00088622_S21	South Africa	South Africa	Beijing	S	S	S	S	S	S	S	S
TRL0115885_S21	South Africa	South Africa	Beijing	n/a	R	S	n/a	R	R	S	S
BK00092074_S31	South Africa	South Africa	Beijing	S	S	S	S	S	S	S	S
BK00092214_S19	South Africa	South Africa	Beijing	S	S	S	S	S	S	S	S
BF01182050_S12	South Africa	South Africa	LAM	S	S	S	S	S	S	S	S
OI00115044_S8	South Africa	South Africa	lineage4	S	R	S	S	S	R	S	S
BD00535343_S6	South Africa	South Africa	Beijing	S	S	S	S	S	S	S	S
TRL0115627_S17	South Africa	South Africa	Beijing	n/a	R	R	n/a	R	R	R	R
JG00407001_S7	South Africa	South Africa	S-type	S	S	S	S	S	S	S	S
TRL0117027_S18	South Africa	South Africa	Beijing	n/a	R	S	n/a	S	F	S	S
BE01336541_S20	South Africa	South Africa	Beijing	S	S	S	S	S	S	S	S
TRL0114849_S9	South Africa	South Africa	Haarlem	n/a	R	S	n/a	R	R	S	R
ED00865824_S23	South Africa	South Africa	Beijing	S	S	S	S	S	S	S	S
TRL0116938_S15	South Africa	South Africa	X-type	n/a	R	R	n/a	R	R	S	R
DG00574978_S20	South Africa	South Africa	lineage4	S	S	S	S	S	S	S	S
DH00748222_S23	South Africa	South Africa	S-type	S	S	S	S	S	S	S	S
EI00656774_S9	South Africa	South Africa	lineage4	S	S	S	S	S	S	S	S
BT00026108_S4	South Africa	South Africa	LAM	S	S	S	S	S	S	S	S
PM00232791_S2	South Africa	South Africa	EAI	R	R	R	R	R	R	R	R
GH00391555_S28	South Africa	South Africa	Haarlem	S	S	S	S	S	S	S	S
TRL0115207_S18	South Africa	South Africa	LAM	n/a	R	R	n/a	R	R	R	R
TRL0101901_S6	South Africa	South Africa	X-type	R	R	S	R	R	R	R	R
TRL0113512_S7	South Africa	South Africa	X-type	R	R	R	S	R	R	R	U
RB00104514_S10	South Africa	South Africa	lineage4	R	R	S	S	R	F	S	S
PI00170885_S5	South Africa	South Africa	EAI	R	R	R	S	R	R	R	R

CE01554674_S11	South Africa	South Africa	LAM	S	S	S	S	S	S	S	S
TRL0113615_S13	South Africa	South Africa	LAM	S	R	S	S	S	R	S	S
DH00599289_S31	South Africa	South Africa	LAM	S	S	S	S	S	S	S	S
JG00423359_S8	South Africa	South Africa	Beijing	S	S	S	S	S	U	S	S
CE01607859_S23	South Africa	South Africa	lineage4	R	R	R	S	R	R	R	R
TRL0117139_S14	South Africa	South Africa	LAM	S	R	S	n/a	S	F	S	S
BF01457863_S17	South Africa	South Africa	Haarlem	S	S	S	S	S	S	S	S
TRL0038641_S31	South Africa	South Africa	Beijing	S	R	S	R	S	R	S	S
EI00664009_S15	South Africa	South Africa	LAM	S	S	S	S	S	S	S	S
TRL0114541_S38	South Africa	South Africa	Beijing	n/a	R	S	n/a	S	R	S	S
ED00778346_S30	South Africa	South Africa	Haarlem	S	S	S	S	S	S	S	S
TRL0112305_S21	South Africa	South Africa	EAI	S	S	S	S	S	S	U	S
TRL0114478_S1	South Africa	South Africa	Haarlem	n/a	R	R	n/a	R	R	R	R
TRL0090177_S27	South Africa	South Africa	Beijing	S	R	S	S	S	R	S	S
GH00318902_S11	South Africa	South Africa	LAM	S	S	S	S	S	S	S	S
BE01203367_S13	South Africa	South Africa	Haarlem	S	S	S	S	S	F	S	S
BT00030402_S31	South Africa	South Africa	Beijing	S	S	S	S	S	S	S	S
ED00922760_S24	South Africa	South Africa	Delhi	S	S	S	S	S	S	S	S
FB00459122_S20	South Africa	South Africa	S-type	S	S	S	S	S	S	S	S
CE01831847_S28	South Africa	South Africa	Beijing	S	S	S	S	S	S	S	S
PA00082962_S7	South Africa	South Africa	Beijing	S	R	S	S	U	R	S	S
EI00622581_S5	South Africa	South Africa	Haarlem	S	S	R	S	S	S	S	S
TRL0074347_S14	South Africa	South Africa	Beijing	R	R	S	R	R	R	R	U
CD00719929_S23	South Africa	South Africa	Beijing	S	S	S	S	S	S	S	S
TRL0116137_S16	South Africa	South Africa	Beijing	n/a	R	R	n/a	R	R	R	S
BF01140560_S19	South Africa	South Africa	LAM	S	S	S	S	S	S	S	S
CE01717296_S22	South Africa	South Africa	LAM	R	S	S	S	R	S	S	S
PS00263922_S4	South Africa	South Africa	Beijing	R	R	R	S	R	R	R	S
CE01578442_S31	South Africa	South Africa	Beijing	S	S	S	S	S	S	S	S
EB00181559_S10	South Africa	South Africa	Beijing	S	S	S	S	S	S	S	S
DH00699469_S18	South Africa	South Africa	lineage4	S	S	S	S	S	S	S	S
DH00698631_S3	South Africa	South Africa	Beijing	S	S	S	S	S	S	S	S
DH00668045_S9	South Africa	South Africa	EAI	S	S	S	S	S	U	U	S
OK00054059_S28	South Africa	South Africa	X-type	S	R	S	S	S	R	U	S
BQ00032365_S10	South Africa	South Africa	X-type	S	S	S	S	S	S	S	S
PK00524261_S29	South Africa	South Africa	lineage4	R	R	S	S	R	R	R	R
BK00089243_S26	South Africa	South Africa	Beijing	R	S	S	S	R	S	S	S
TRL0115767_S13	South Africa	South Africa	X-type	n/a	R	R	n/a	R	R	R	U
BF01298771_S19	South Africa	South Africa	Beijing	S	R	S	S	S	R	S	S
EB00198368_S29	South Africa	South Africa	Beijing	S	S	S	S	S	S	S	S
TRL0085876_S27	South Africa	South Africa	LAM	R	R	S	S	R	R	S	S
TRL0111034_S3	South Africa	South Africa	LAM	R	R	R	R	R	R	R	R
CG01490116_S15	South Africa	South Africa	lineage4	S	S	S	S	S	S	S	S
ED00926802_S31	South Africa	South Africa	Haarlem	S	S	S	S	F	S	S	S
EF00226974_S29	South Africa	South Africa	LAM	S	S	S	S	S	S	S	S
EF00237922_S30	South Africa	South Africa	LAM	S	S	S	S	S	S	S	S
DH00593017_S30	South Africa	South Africa	LAM	S	S	S	S	S	U	S	S
TRL0090962_S8	South Africa	South Africa	Beijing	S	R	S	S	S	R	S	S
BK00092314_S2	South Africa	South Africa	Beijing	S	S	S	S	S	S	S	S
BF01338209_S28	South Africa	South Africa	Beijing	S	S	S	S	S	S	S	S
BR00085359_S19	South Africa	South Africa	LAM	S	S	S	S	S	S	U	S
EI00670575_S16	South Africa	South Africa	LAM	R	S	R	R	R	R	R	R
BE01141175_S8	South Africa	South Africa	S-type	S	S	S	S	S	S	S	S
CD00810261_S27	South Africa	South Africa	LAM	S	S	S	S	S	S	S	S
BK00092575_S2	South Africa	South Africa	Haarlem	S	S	S	S	S	S	U	S
ED00958940_S13	South Africa	South Africa	Delhi	S	S	S	S	S	S	S	S
TRL0115684_S19	South Africa	South Africa	LAM	n/a	R	R	n/a	R	R	S	R
BF01238870_S3	South Africa	South Africa	LAM	S	S	S	S	S	S	S	S
PK00537387_S12	South Africa	South Africa	LAM	R	R	S	R	R	R	R	R
BE01210845_S24	South Africa	South Africa	lineage4	R	S	S	S	S	S	S	S
FD00214542_S18	South Africa	South Africa	lineage4	R	R	S	R	R	R	S	R
TRL0113640_S43	South Africa	South Africa	S-type	R	R	R	R	R	R	R	R
TRL0115584_S2	South Africa	South Africa	LAM	n/a	R	R	n/a	R	R	R	R
BN00046706_S8	South Africa	South Africa	Haarlem	S	S	S	S	S	S	S	S
GH00373074_S7	South Africa	South Africa	Delhi	S	S	S	S	S	S	S	S
BN00034754_S19	South Africa	South Africa	LAM	S	S	S	S	S	S	S	S
TRL0116131_S3	South Africa	South Africa	S-type	n/a	S	S	n/a	S	S	S	S
BE01408890_S6	South Africa	South Africa	Beijing	S	S	S	S	S	S	S	S
TRL0071026_S23	South Africa	South Africa	Beijing	S	R	S	S	S	R	S	S
BF01077380_S24	South Africa	South Africa	Beijing	S	S	S	S	S	S	S	S
TRL0115152_S14	South Africa	South Africa	Beijing	n/a	R	R	n/a	R	R	R	R
BR00093393_S5	South Africa	South Africa	Beijing	S	S	S	S	S	S	S	S
EB00190563_S32	South Africa	South Africa	Haarlem	S	S	S	S	S	S	S	S
BN00047437_S8	South Africa	South Africa	S-type	S	S	S	S	S	S	S	S
TRL0113018_S23	South Africa	South Africa	LAM	R	R	S	R	R	R	R	R
EI00660934_S19	South Africa	South Africa	S-type	S	S	S	S	S	S	S	S
TRL0115878_S23	South Africa	South Africa	X-type	n/a	R	R	n/a	R	R	R	U
EI00613661_S19	South Africa	South Africa	LAM	S	S	R	S	U	S	R	S
BF01303317_S12	South Africa	South Africa	Beijing	R	S	S	n/a	S	S	S	S
EI00668681_S5	South Africa	South Africa	S-type	S	S	S	S	S	S	S	S
DH00686929_S26	South Africa	South Africa	S-type	S	S	S	S	S	S	S	S
DH00727632_S1	South Africa	South Africa	LAM	S	S	S	n/a	S	S	S	S
BQ00025312_S26	South Africa	South Africa	Beijing	S	S	S	S	S	S	S	S
BQ00025777_S9	South Africa	South Africa	LAM	S	S	S	S	S	S	S	S
CE01671817_S27	South Africa	South Africa	Beijing	S	S	S	S	S	S	S	S
DH00683997_S6	South Africa	South Africa	S-type	S	S	S	S	S	S	S	S
CE01938197_S27	South Africa	South Africa	X-type	S	S	S	S	S	S	S	S
JA00127310_S1	South Africa	South Africa	Beijing	S	R	R	S	U	R	R	S
CD00804167_S25	South Africa	South Africa	Delhi	S	S	S	S	S	S	S	S
KD00184898_S16	South Africa	South Africa	Haarlem	S	R	S	S	S	F	S	S
ED00788365_S22	South Africa	South Africa	Beijing	R	S	S	S	S	S	S	S
BT00028429_S12	South Africa	South Africa	LAM	S	S	S	S	S	S	S	S
TRL0113491_S9	South Africa	South Africa	Beijing	S	R	S	S	S	R	S	S
RJ00263599_S11	South Africa	South Africa	Beijing	S	R	S	S	S	S	S	S
BQ00031148_S31	South Africa	South Africa	S-type	S	S	S	S	S	S	S	S
BT00031968_S17	South Africa	South Africa	LAM	S	S	S	S	S	S	S	F
OB00115404_S32	South Africa	South Africa	LAM	R	R	S	R	R	R	R	R
CE01835349_S28	South Africa	South Africa	Beijing	S	S	S	S	S	S	S	S
BT00030879_S21	South Africa	South Africa	Beijing	S	S	S	S	S	U	S	S
UH00163533_S8	South Africa	South Africa	Beijing	R	R	S	S	R	R	R	R
BK00091788_S23	South Africa	South Africa	lineage4	R	S	S	S	S	S	S	S
EI00587085_S6	South Africa	South Africa	Beijing	S	S	S	S	S	S	S	S
JF00065486_S4	South Africa	South Africa	S-type	S	S	S	S	S	S	S	S
BF01140550_S32	South Africa	South Africa	S-type	S	S	S	S	S	S	S	S
CH00427792_S8	South Africa	South Africa	Beijing	S	S	S	S	S	S	S	S
DG00561785_S15	South Africa	South Africa	Delhi	S	S	S	S	S	S	U	S
CD00741689_S25	South Africa	South Africa	M. bovis	S	S	S	R	S	S	S	R
DH00742746_S18	South Africa	South Africa	LAM	S	S	S	S	S	S	S	S
BK00092681_S23	South Africa	South Africa	Beijing	S	S	S	S	S	S	S	S
TRL0116379_S22	South Africa	South Africa	Beijing	n/a	R	R	n/a	R	R	R	S
DH00724524_S23	South Africa	South Africa	Beijing	S	S	S	S	S	S	S	S
BF01183602_S13	South Africa	South Africa	lineage4	S	S	S	S	S	S	S	S
CG01343657_S3	South Africa	South Africa	S-type	S	S	S	R	S	S	S	S
CE01907646_S23	South Africa	South Africa	Beijing	S	S	S	S	S	S	S	S
TRL0043835_S18	South Africa	South Africa	S-type	S	R	S	S	S	S	S	S
CE01925232_S11	South Africa	South Africa	Beijing	S	S	S	S	S	S	S	S

BF01381806_S25	South Africa	South Africa	EAI	R	R	R	R	R	R	R	R
VG00072668_S28	South Africa	South Africa	Beijing	R	R	R	R	R	R	R	R
CD00910298_S12	South Africa	South Africa	S-type	S	S	S	S	S	S	S	S
DH00680066_S18	South Africa	South Africa	Beijing	S	S	S	S	S	S	S	S
CD00956688_S13	South Africa	South Africa	Beijing	S	S	S	S	S	S	S	S
BF01462160_S23	South Africa	South Africa	Haarlem	S	S	S	S	S	S	S	S
EI00671944_S17	South Africa	South Africa	lineage4	S	S	S	S	S	S	S	S
BF01375035_S30	South Africa	South Africa	lineage4	S	S	S	S	S	S	S	S
TRL0115518_S23	South Africa	South Africa	Beijing	n/a	R	R	n/a	R	R	R	R
BF01298605_S30	South Africa	South Africa	X-type	R	R	R	R	R	R	R	R
TRL0114976_S5	South Africa	South Africa	X-type	n/a	R	R	n/a	R	R	R	R
EI00681448_S18	South Africa	South Africa	S-type	S	S	S	S	S	S	S	S
BE01391328_S19	South Africa	South Africa	LAM	S	S	S	S	S	S	S	S
FD00218610_S27	South Africa	South Africa	Beijing	S	S	S	S	S	S	S	S
TRL0115007_S10	South Africa	South Africa	X-type	n/a	R	R	n/a	R	R	R	U
DH00720643_S16	South Africa	South Africa	S-type	S	S	S	S	S	S	S	S
BT00030753_S10	South Africa	South Africa	Beijing	S	S	S	S	S	S	S	S
TRL0037975_S12	South Africa	South Africa	lineage4	R	R	S	S	S	R	S	S
CG01442001_S4	South Africa	South Africa	LAM	S	S	S	S	S	S	U	S
BF01114045_S28	South Africa	South Africa	lineage4	S	S	S	S	S	F	S	S
JC00272056_S3	South Africa	South Africa	lineage4	S	S	S	S	S	S	S	S
TRL0113344_S3	South Africa	South Africa	S-type	R	R	R	R	R	R	R	R
CH00423116_S13	South Africa	South Africa	Delhi	S	S	R	S	S	S	S	S
TRL0097309_S23	South Africa	South Africa	Beijing	R	R	R	R	R	R	R	R
BH00962935_S12	South Africa	South Africa	Haarlem	S	S	S	S	S	S	S	S
BD00638311_S6	South Africa	South Africa	Beijing	S	S	S	S	S	S	S	S
BH00962938_S13	South Africa	South Africa	Beijing	R	S	S	S	S	S	S	F
EF00224395_S27	South Africa	South Africa	LAM	S	S	S	S	S	F	S	S
CD00729150_S30	South Africa	South Africa	S-type	S	S	S	S	S	S	S	S
GH00378509_S15	South Africa	South Africa	lineage4	S	S	S	S	S	S	S	S
BF01330889_S14	South Africa	South Africa	Beijing	S	S	S	S	S	S	S	S
EF00237909_S14	South Africa	South Africa	LAM	S	S	S	S	S	S	S	S
PJ00572527_S11	South Africa	South Africa	lineage4	S	S	S	S	S	S	S	S
PS00308033_S27	South Africa	South Africa	S-type	S	R	S	S	S	R	S	S
BR00088315_S17	South Africa	South Africa	Beijing	S	S	S	S	S	S	S	S
BF01443928_S15	South Africa	South Africa	lineage4	S	S	S	S	S	S	S	S
BK00089244_S27	South Africa	South Africa	Haarlem	S	S	S	S	S	U	S	S
BR00093737_S29	South Africa	South Africa	Beijing	S	S	S	S	S	S	S	S
DG00633179_S12	South Africa	South Africa	Beijing	S	S	S	S	S	S	U	S
DH00654552_S21	South Africa	South Africa	LAM	R	S	S	S	R	S	S	S
BK00092579_S3	South Africa	South Africa	Haarlem	S	S	S	S	S	S	U	S
PH00104960_S28	South Africa	South Africa	LAM	R	R	S	R	R	R	R	R
TRL0114647_S13	South Africa	South Africa	Beijing	n/a	R	S	n/a	R	R	S	R
TRL0115824_S12	South Africa	South Africa	EAI	n/a	R	R	n/a	R	R	R	R
DG00577274_S21	South Africa	South Africa	X-type	S	S	S	S	S	S	S	S
EF00248786_S5	South Africa	South Africa	S-type	S	S	S	S	S	S	S	S
EI00657490_S10	South Africa	South Africa	Beijing	S	S	S	S	S	S	S	S
TRL0090790_S26	South Africa	South Africa	Beijing	S	R	S	S	S	R	S	S
TRL0115051_S20	South Africa	South Africa	Beijing	n/a	R	R	n/a	R	R	R	R
TRL0115164_S16	South Africa	South Africa	lineage4	n/a	R	S	n/a	R	R	S	S
BR00088842_S24	South Africa	South Africa	Beijing	S	S	S	S	S	S	S	S
CE01938379_S1	South Africa	South Africa	Delhi	S	S	S	S	S	S	S	S
TH00083947_S19	South Africa	South Africa	Beijing	R	R	S	S	R	R	S	S
BD00678571_S1	South Africa	South Africa	LAM	S	S	S	S	S	S	S	S
BK00082889_S23	South Africa	South Africa	LAM	S	S	S	S	S	S	S	S
BF01445536_S20	South Africa	South Africa	Delhi	S	S	S	S	S	S	S	S
DG00603671_S24	South Africa	South Africa	Beijing	S	S	S	S	S	S	S	S
TRL0114671_S15	South Africa	South Africa	lineage4	n/a	R	R	n/a	R	R	U	F
BR00069990_S23	South Africa	South Africa	LAM	S	S	S	S	U	S	S	S
TRL0113629_S18	South Africa	South Africa	Beijing	R	R	S	S	S	S	S	S
BF01355969_S29	South Africa	South Africa	LAM	S	S	S	S	S	S	S	S
BK00083986_S22	South Africa	South Africa	Beijing	R	R	S	S	R	R	S	S
BF01492002_S17	South Africa	South Africa	Haarlem	S	S	S	S	S	S	S	F
BD00633856_S5	South Africa	South Africa	Beijing	S	S	S	S	S	S	S	S
EI00649150_S31	South Africa	South Africa	LAM	S	S	S	S	S	S	S	S
EF00222552_S26	South Africa	South Africa	S-type	S	S	S	S	S	S	S	S
CE01780342_S22	South Africa	South Africa	Haarlem	S	S	S	S	S	S	S	S
BT00031496_S9	South Africa	South Africa	Haarlem	S	S	S	S	S	S	S	S
TRL0079859_S29	South Africa	South Africa	Beijing	S	R	S	S	F	R	F	F
CE01925225_S30	South Africa	South Africa	LAM	R	S	S	S	S	S	S	S
TRL0114500_S22	South Africa	South Africa	Beijing	n/a	R	R	n/a	R	R	R	R
PO00214147_S2	South Africa	South Africa	Haarlem	R	R	R	R	R	R	R	R
JC00276287_S3	South Africa	South Africa	LAM	S	S	S	S	S	S	S	S
TRL0114650_S1	South Africa	South Africa	Beijing	n/a	R	R	n/a	R	R	R	R
DG00574983_S11	South Africa	South Africa	lineage4	S	S	S	S	U	S	S	S
TRL0114820_S18	South Africa	South Africa	X-type	n/a	R	R	n/a	R	R	R	R
CD00833444_S16	South Africa	South Africa	LAM	S	S	S	S	S	S	S	S
BF01306090_S21	South Africa	South Africa	Haarlem	S	S	S	S	S	S	S	S
BF01330871_S25	South Africa	South Africa	S-type	S	S	S	S	S	S	S	S
CH00420625_S32	South Africa	South Africa	Beijing	S	S	S	S	S	S	S	S
ED00795304_S20	South Africa	South Africa	Haarlem	S	S	S	S	S	S	S	S
PM00152727_S26	South Africa	South Africa	X-type	R	R	S	R	R	R	R	U
CE01938341_S25	South Africa	South Africa	X-type	S	S	S	S	S	S	S	S
TRL0113519_S20	South Africa	South Africa	Haarlem	R	R	R	R	R	R	R	R
EN00017245_S19	South Africa	South Africa	Delhi	S	S	S	S	S	S	S	S
BD00703465_S6	South Africa	South Africa	Beijing	S	S	S	S	S	S	S	S
BF01344988_S15	South Africa	South Africa	EAI	S	S	S	S	S	S	S	S
DH00593002_S28	South Africa	South Africa	Beijing	S	S	S	S	S	S	S	S
TRL0091794_S8	South Africa	South Africa	X-type	R	R	S	S	R	R	R	S
TRL0115183_S17	South Africa	South Africa	LAM	n/a	R	R	n/a	R	R	R	R
EF00227866_S23	South Africa	South Africa	Beijing	S	S	S	S	S	S	S	S
CD00962085_S15	South Africa	South Africa	S-type	S	S	S	S	S	S	S	S
DH00752822_S27	South Africa	South Africa	LAM	S	S	S	S	S	S	S	S
EI00718954_S6	South Africa	South Africa	Delhi	S	S	S	S	S	S	S	S
TRL0112980_S21	South Africa	South Africa	LAM	S	R	S	S	S	R	S	S
BQ00025556_S7	South Africa	South Africa	LAM	R	S	S	S	S	S	S	S
TRL0086437_S6	South Africa	South Africa	LAM	R	R	R	R	R	R	R	R
TRL0116230_S6	South Africa	South Africa	X-type	n/a	R	R	n/a	R	R	R	R
BH00984850_S8	South Africa	South Africa	lineage4	S	S	S	S	S	S	S	S
BQ00029615_S20	South Africa	South Africa	Beijing	S	S	S	S	S	S	S	S
TRL0074450_S32	South Africa	South Africa	LAM	S	R	S	S	S	F	S	S
CG01334682_S2	South Africa	South Africa	LAM	S	S	S	S	S	S	S	S
TRL0022086_S15	South Africa	South Africa	S-type	R	R	R	S	R	R	R	R
ED00874424_S22	South Africa	South Africa	S-type	S	S	S	S	S	S	S	S
TRL0115412_S22	South Africa	South Africa	S-type	S	S	S	n/a	S	S	S	S
TRL0057671_S18	South Africa	South Africa	Beijing	R	R	S	S	R	R	S	S
UJ00125495_S24	South Africa	South Africa	Beijing	R	R	R	R	R	R	R	R
BT00026048_S12	South Africa	South Africa	LAM	S	S	S	S	S	S	S	S
BK00092684_S23	South Africa	South Africa	Beijing	S	S	S	S	S	S	S	S
CE01825534_S2	South Africa	South Africa	Haarlem	S	S	S	S	S	S	S	S
BE01210097_S15	South Africa	South Africa	Beijing	S	S	S	S	S	S	S	S
DH00659004_S17	South Africa	South Africa	LAM	S	S	S	S	S	S	S	S
TRL0085451_S24	South Africa	South Africa	Haarlem	R	R	n/a	R	R	R	R	R
TRL0115652_S40	South Africa	South Africa	Beijing	n/a	R	R	n/a	R	R	R	R
JA00214410_S4	South Africa	South Africa	lineage4	S	R	S	S	S	R	S	S
BF01114045_S1	South Africa	South Africa	lineage4	S	S	S	S	S	S	S	S

BK00082371_S21	South Africa	South Africa	Beijing	S	S	S	S	S	S	S	S
TRL0114825_S19	South Africa	South Africa	Haarlem	n/a	R	R	n/a	R	R	R	R
TRL0115400_S13	South Africa	South Africa	Beijing	n/a	R	R	n/a	R	R	R	R
TRL0114583_S12	South Africa	South Africa	Haarlem	n/a	R	R	n/a	R	R	R	R
TRL0114455_S23	South Africa	South Africa	Beijing	n/a	R	R	n/a	R	R	R	R
BR00077729_S24	South Africa	South Africa	Beijing	S	S	S	S	S	S	S	S
EF00234899_S31	South Africa	South Africa	S-type	S	S	S	S	S	S	S	S
TRL0115469_S6	South Africa	South Africa	X-type	n/a	R	R	n/a	R	R	R	R
BH00815045_S6	South Africa	South Africa	LAM	S	S	S	S	S	S	S	S
CG01628752_S1	South Africa	South Africa	Haarlem	S	S	S	S	S	S	S	S
TRL0115095_S12	South Africa	South Africa	X-type	n/a	R	R	n/a	R	R	R	U
BK00078859_S16	South Africa	South Africa	X-type	S	S	S	S	S	S	S	S
BF01506399_S20	South Africa	South Africa	lineage4	S	S	S	S	S	S	S	S
BK00082596_S22	South Africa	South Africa	Beijing	S	S	S	S	S	S	S	S
BF01375032_S27	South Africa	South Africa	lineage4	S	S	S	S	S	S	S	S
BN00034572_S2	South Africa	South Africa	Beijing	S	S	S	S	S	S	S	S
ND00124877_S3	South Africa	South Africa	lineage4	R	R	S	S	R	R	R	S
BE01369677_S11	South Africa	South Africa	Beijing	S	S	S	S	S	S	S	S
BT00031958_S15	South Africa	South Africa	LAM	S	S	S	S	S	S	S	S
TRL0101440_S5	South Africa	South Africa	Beijing	R	R	S	R	R	R	R	R
BE01460858_S30	South Africa	South Africa	Beijing	S	S	S	S	S	S	S	S
CH00435709_S12	South Africa	South Africa	LAM	S	S	S	S	S	S	S	S
TRL0115487_S9	South Africa	South Africa	Beijing	n/a	R	R	n/a	R	R	R	R
BH00973542_S9	South Africa	South Africa	Delhi	S	S	S	S	S	S	S	S
NF00130189_S31	South Africa	South Africa	LAM	S	R	S	S	S	R	S	S
TRL0069553_S22	South Africa	South Africa	lineage4	R	R	S	R	R	R	U	R
BT00033626_S19	South Africa	South Africa		S	S	S	S	S	S	S	S
TRL0113465_S6	South Africa	South Africa	LAM	R	R	R	R	R	R	R	R
TRL0113483_S7	South Africa	South Africa	X-type	R	R	S	R	R	R	R	R
EI00712484_S16	South Africa	South Africa	LAM	S	S	S	S	S	S	S	S
CG01595930_S6	South Africa	South Africa	Beijing	S	S	S	S	S	S	S	S
GH00377594_S8	South Africa	South Africa	LAM	S	S	S	S	S	S	S	S
BF01178146_S9	South Africa	South Africa	Beijing	S	S	S	S	S	S	S	S
EF00237066_S21	South Africa	South Africa	LAM	S	S	S	S	U	S	S	S
EF00266177_S19	South Africa	South Africa	LAM	S	S	n/a	S	S	S	S	S
CE01705388_S30	South Africa	South Africa	LAM	R	R	R	R	F	F	F	S
TRL0023983_S1	South Africa	South Africa	Haarlem	S	R	S	S	R	R	S	S
FB00478424_S22	South Africa	South Africa	Haarlem	S	S	S	S	S	S	S	S
BR00088729_S20	South Africa	South Africa	Beijing	S	S	S	S	S	S	S	S
CD00793787_S24	South Africa	South Africa	S-type	S	S	S	S	S	S	S	S
DA00230689_S12	South Africa	South Africa	Beijing	S	S	S	S	S	S	S	S
TRL0113326_S2	South Africa	South Africa	lineage4	S	R	S	S	S	R	S	S
BF01498044_S30	South Africa	South Africa	Tur	S	S	S	S	S	S	S	S
BE01493568_S4	South Africa	South Africa	lineage4	S	S	S	S	S	S	S	S
BD00717578_S19	South Africa	South Africa	Beijing	S	S	S	S	S	R	S	S
BK00090322_S27	South Africa	South Africa	LAM	S	S	S	S	S	S	S	S
TRL0113579_S16	South Africa	South Africa	Beijing	R	R	S	S	R	R	R	S
TRL0113476_S5	South Africa	South Africa	LAM	R	R	S	S	R	R	S	S
BT00031569_S11	South Africa	South Africa	LAM	S	S	S	S	S	S	S	S
TRL0115027_S9	South Africa	South Africa	S-type	n/a	R	R	n/a	R	R	R	U
BF01430245_S13	South Africa	South Africa	S-type	R	R	S	S	R	R	R	S
DH00713165_S6	South Africa	South Africa	Beijing	S	S	S	S	S	S	S	S
BN00034198_S21	South Africa	South Africa	LAM	S	S	S	S	S	S	S	S
TRL0115418_S17	South Africa	South Africa	X-type	n/a	R	R	n/a	R	R	R	R
BF01527857_S10	South Africa	South Africa	Beijing	S	S	S	n/a	S	S	S	S
DH00571957_S27	South Africa	South Africa	LAM	S	S	S	S	S	S	S	S
PL00048658_S9	South Africa	South Africa	EAI	R	R	R	R	R	R	R	R
BF01176828_S8	South Africa	South Africa	Beijing	S	S	S	S	S	S	S	S
ON00035801_S6	South Africa	South Africa	S-type	S	R	S	S	S	S	S	S
CE01896637_S15	South Africa	South Africa	S-type	S	S	S	S	S	S	S	S
TRL0117085_S13	South Africa	South Africa	Beijing	n/a	R	S	n/a	F	R	S	S
PE00291237_S30	South Africa	South Africa	EAI	R	R	R	S	R	R	R	R
BE01454196_S32	South Africa	South Africa	LAM	S	S	S	S	S	S	S	S
TRL0110907_S30	South Africa	South Africa	Beijing	R	R	R	n/a	R	R	R	R
DG00529724_S18	South Africa	South Africa	lineage4	S	S	S	S	S	S	S	S
CE01634476_S1	South Africa	South Africa	lineage4	S	S	S	S	S	S	S	S
CG01433802_S32	South Africa	South Africa	Beijing	S	S	S	S	S	S	S	S
BK00092406_S1	South Africa	South Africa	Beijing	S	S	S	S	S	S	S	S
BR00089840_S22	South Africa	South Africa	Beijing	S	S	S	S	S	S	S	S
CE01825542_S10	South Africa	South Africa	Beijing	S	S	S	S	S	S	S	S
BD00717563_S18	South Africa	South Africa	Beijing	S	S	S	S	S	S	F	S
TRL0114836_S8	South Africa	South Africa	Beijing	n/a	R	R	n/a	R	R	R	R
EI00661002_S11	South Africa	South Africa	LAM	S	S	S	S	S	S	S	S
TRL0114666_S11	South Africa	South Africa	Beijing	n/a	R	R	n/a	R	R	R	U
KG00082202_S6	South Africa	South Africa	Beijing	S	R	S	S	R	R	S	S
BE01453084_S18	South Africa	South Africa	Beijing	S	S	S	R	S	S	S	R
OO00095615_S5	South Africa	South Africa	X-type	R	R	S	S	U	R	S	S
TRL0107371_S31	South Africa	South Africa	LAM	R	R	S	R	R	R	R	U
ED00890569_S24	South Africa	South Africa	LAM	S	S	S	S	S	F	S	S
QE00093878_S13	South Africa	South Africa	LAM	R	R	S	S	R	R	S	S
TRL0115006_S7	South Africa	South Africa	Haarlem	n/a	R	R	n/a	R	R	R	R
DH00749851_S26	South Africa	South Africa	lineage4	S	S	S	S	S	S	S	S
BQ00024961_S9	South Africa	South Africa	LAM	S	n/a	S	S	S	S	S	S
QF00383820_S1	South Africa	South Africa	X-type	S	R	S	S	U	R	S	S
BF01522217_S16	South Africa	South Africa	Haarlem	S	S	S	S	S	S	S	S
BF01497981_S12	South Africa	South Africa	Beijing	S	S	S	S	S	S	S	S
CE01678834_S30	South Africa	South Africa	Beijing	S	S	S	S	S	S	S	S
OC00061697_S7	South Africa	South Africa	S-type	S	R	S	S	S	R	S	S
BF01175381_S6	South Africa	South Africa	S-type	S	S	S	S	S	S	S	S
BT00027013_S3	South Africa	South Africa	LAM	S	S	S	S	S	S	S	S
DH00680828_S12	South Africa	South Africa	Beijing	R	R	S	S	R	R	R	S
BE01468444_S32	South Africa	South Africa	Beijing	S	S	S	S	S	S	S	S
JG00406558_S5	South Africa	South Africa	LAM	S	S	S	S	S	S	S	S
EB00198607_S8	South Africa	South Africa	Beijing	S	S	S	S	S	S	S	S
CH00388401_S12	South Africa	South Africa	Beijing	S	S	S	S	S	S	S	S
TRL0113061_S24	South Africa	South Africa	LAM	R	R	R	R	R	R	R	R
EB00160449_S28	South Africa	South Africa	Delhi	S	S	S	S	S	R	S	S
TRL0078924_S15	South Africa	South Africa	Beijing	R	R	R	R	R	R	R	R
BK00075150_S6	South Africa	South Africa	S-type	S	S	S	S	S	S	S	S
PD00160471_S10	South Africa	South Africa	Beijing	S	R	S	S	S	R	S	S
FB00525549_S16	South Africa	South Africa	LAM	S	S	S	R	S	S	S	S
ED00795230_S19	South Africa	South Africa	Beijing	S	S	S	S	S	S	U	S
CE01912219_S14	South Africa	South Africa	Beijing	S	S	S	S	S	S	S	S
BF01475106_S3	South Africa	South Africa	Delhi	S	S	S	S	S	U	S	S
JD00128975_S5	South Africa	South Africa	Delhi	R	R	S	S	R	R	S	S
ED00868728_S21	South Africa	South Africa	Beijing	S	S	S	S	S	S	S	S
CE01476125_S3	South Africa	South Africa	Haarlem	S	S	S	S	S	S	S	S
BF01310182_S29	South Africa	South Africa	lineage4	S	S	S	S	S	S	S	S
BF01099205_S27	South Africa	South Africa	Beijing	S	S	S	S	S	S	S	S
FD00221831_S1	South Africa	South Africa	Delhi	S	S	S	S	S	S	S	S
OS00120406_S3	South Africa	South Africa	Haarlem	S	R	S	S	S	S	S	S
BQ00028721_S5	South Africa	South Africa	lineage4	S	S	S	S	S	S	S	S
BN00045763_S11	South Africa	South Africa	lineage4	S	S	S	S	S	S	U	S
BF01445461_S20	South Africa	South Africa	Beijing	S	S	S	S	S	S	S	S
KD00166042_S9	South Africa	South Africa	Beijing	S	R	S	S	S	S	S	S
CE01400812_S28	South Africa	South Africa	LAM	S	S	S	S	S	S	S	S

TRL0055385_S29	South Africa	South Africa	Beijing	R	S	S	S	R	S	S	S
BD00734082_S7	South Africa	South Africa	LAM	S	S	n/a	S	S	F	S	S
TRL0113572_S11	South Africa	South Africa	Beijing	R	R	S	R	R	R	R	U
DA00304169_S13	South Africa	South Africa	Beijing	S	S	S	S	S	S	S	S
EF00236514_S32	South Africa	South Africa	Beijing	S	S	S	S	S	S	S	S
TRL0114153_S9	South Africa	South Africa	Beijing	n/a	R	S	n/a	R	R	S	R
TRL0063310_S32	South Africa	South Africa	LAM	S	S	S	S	S	S	S	S
TRL0114786_S17	South Africa	South Africa	Beijing	n/a	S	S	n/a	S	R	S	S
TRL0095023_S9	South Africa	South Africa	Haarlem	R	R	S	S	R	R	U	S
BT00030778_S26	South Africa	South Africa	LAM	S	S	S	S	S	S	S	S
BH00849239_S10	South Africa	South Africa	lineage4	R	R	S	R	R	R	R	R
DH00634111_S32	South Africa	South Africa	LAM	R	S	S	S	S	S	S	S
DG00678506_S30	South Africa	South Africa	LAM	S	S	S	S	S	S	S	S
BE01233785_S17	South Africa	South Africa	Beijing	S	S	S	S	S	S	S	S
BQ00025602_S8	South Africa	South Africa	EAI	R	S	S	S	S	S	U	S
GH00357231_S5	South Africa	South Africa	S-type	S	S	S	S	S	S	S	S
TRL0115707_S20	South Africa	South Africa	Beijing	n/a	R	R	n/a	R	R	R	U
VG00075805_S1	South Africa	South Africa	Beijing	R	R	S	S	S	S	S	S
BF01178187_S10	South Africa	South Africa	LAM	S	S	S	S	S	F	S	S
DA00302840_S25	South Africa	South Africa	LAM	S	S	S	S	S	S	S	S
DG00561812_S15	South Africa	South Africa	Beijing	S	S	S	S	S	R	S	S
TRL0113484_S8	South Africa	South Africa	LAM	R	R	R	R	R	R	R	R
TRL0115691_S5	South Africa	South Africa	Beijing	n/a	R	R	n/a	R	R	R	R
BT00031764_S13	South Africa	South Africa	lineage4	S	S	S	S	F	S	S	S
CE01544376_S28	South Africa	South Africa	Beijing	S	R	S	S	S	R	S	S
BK00086651_S24	South Africa	South Africa	LAM	S	S	S	S	S	S	S	S
DG00547345_S18	South Africa	South Africa	X-type	S	S	S	S	S	S	S	S
DH00711253_S21	South Africa	South Africa	LAM	S	S	S	S	S	S	S	S
DH00593016_S15	South Africa	South Africa	X-type	S	S	S	R	F	S	S	R
PE00318284_S6	South Africa	South Africa	LAM	S	R	S	n/a	S	R	S	S
BQ00030098_S6	South Africa	South Africa	LAM	S	S	S	S	S	S	S	S
BK00092578_S27	South Africa	South Africa	S-type	S	S	S	S	S	R	S	S
TRL0116263_S21	South Africa	South Africa	Beijing	R	R	R	R	R	R	R	R
EI00623324_S27	South Africa	South Africa	Delhi	S	S	S	S	S	S	S	S
TRL0065126_S26	South Africa	South Africa	LAM	R	R	S	S	S	S	S	S
NE00095715_S4	South Africa	South Africa	Beijing	S	R	S	S	S	R	S	S
TRL0115420_S14	South Africa	South Africa	Haarlem	n/a	R	R	n/a	R	R	R	R
CD00862502_S27	South Africa	South Africa	Beijing	R	R	R	R	R	R	R	R
BE01402458_S3	South Africa	South Africa	Beijing	S	S	S	S	S	S	S	S
CG01601252_S21	South Africa	South Africa	LAM	S	S	S	S	S	S	S	S
TRL0115591_S16	South Africa	South Africa	LAM	n/a	R	R	n/a	R	R	R	R
UH00236601_S7	South Africa	South Africa	Delhi	S	S	S	S	S	S	S	S
OB00122369_S4	South Africa	South Africa	Beijing	R	R	S	S	R	R	R	S
EF00278139_S16	South Africa	South Africa	Beijing	S	S	S	n/a	S	S	S	S
TRL0115834_S6	South Africa	South Africa	X-type	n/a	R	S	n/a	S	R	S	S
JG00225783_S4	South Africa	South Africa	lineage4	R	R	S	S	R	R	S	S
TRL0113626_S15	South Africa	South Africa	EAI	R	R	R	S	R	R	R	R
BF01381844_S3	South Africa	South Africa	Beijing	S	S	S	S	S	S	S	S
CE01678827_S24	South Africa	South Africa	Beijing	R	S	S	R	R	S	S	S
DH00600299_S32	South Africa	South Africa	Haarlem	S	S	S	S	S	S	S	S
BF01311721_S24	South Africa	South Africa	Beijing	S	S	S	S	S	S	S	S
TRL0114343_S22	South Africa	South Africa	LAM	n/a	R	R	n/a	R	R	R	R
TRL0112306_S9	South Africa	South Africa	S-type	S	S	S	S	S	S	S	S
TRL0115322_S20	South Africa	South Africa	lineage4	n/a	R	R	n/a	R	R	R	R
BD00708969_S4	South Africa	South Africa	S-type	S	S	S	S	S	S	S	S
UH00100518_S14	South Africa	South Africa	Beijing	R	R	R	R	R	R	R	U
TRL0113166_S24	South Africa	South Africa	LAM	R	R	S	R	R	R	S	R
BF01330899_S29	South Africa	South Africa	EAI	S	S	S	S	S	S	U	S
TRL0061144_S30	South Africa	South Africa	Beijing	R	S	S	S	S	S	S	S
BF01080700_S10	South Africa	South Africa	Haarlem	S	S	S	S	S	S	S	S
JC00272049_S2	South Africa	South Africa	lineage4	S	S	S	S	S	S	S	S
JA00213685_S2	South Africa	South Africa	lineage4	S	S	S	S	S	S	S	S
BE01215199_S16	South Africa	South Africa	S-type	S	S	S	S	S	S	U	S
BF01179984_S11	South Africa	South Africa	Beijing	S	S	S	S	S	S	S	S
DH00682387_S12	South Africa	South Africa	Beijing	S	S	S	S	R	S	S	S
TRL0116843_S10	South Africa	South Africa	Beijing	n/a	R	R	n/a	R	R	R	R
EB00195736_S15	South Africa	South Africa	Beijing	S	S	S	S	U	F	S	S
CD00854301_S27	South Africa	South Africa	Beijing	S	S	S	S	S	S	S	S
BD00678560_S29	South Africa	South Africa	Beijing	S	S	S	S	S	S	S	S
BF01491990_S22	South Africa	South Africa	Delhi	S	S	S	S	S	S	S	S
EI00684928_S20	South Africa	South Africa	Haarlem	S	S	S	S	S	S	S	S
TRL0037452_S11	South Africa	South Africa	S-type	S	R	S	S	S	S	S	S
EI00664001_S14	South Africa	South Africa	Haarlem	S	S	S	S	S	S	S	S
TRL0114680_S2	South Africa	South Africa	LAM	n/a	R	R	n/a	R	R	R	R
PA00070896_S17	South Africa	South Africa	EAI	R	R	R	R	R	R	R	R
DG00571046_S13	South Africa	South Africa	Beijing	S	S	S	S	S	S	S	S
CE01938178_S20	South Africa	South Africa	Beijing	S	S	S	S	S	S	S	S
DH00679710_S1	South Africa	South Africa	Beijing	S	S	S	S	S	S	S	S
TRL0105148_S3	South Africa	South Africa	lineage4	S	R	S	S	R	R	S	S
BR00075466_S15	South Africa	South Africa	LAM	S	S	S	S	S	S	S	S
DG00589683_S23	South Africa	South Africa	lineage4	S	S	S	S	S	S	S	S
BK00079405_S17	South Africa	South Africa	Beijing	S	S	S	S	S	S	S	S
EI00681447_S8	South Africa	South Africa	Beijing	S	S	S	S	S	S	S	S
BR00099466_S2	South Africa	South Africa	Beijing	S	S	S	S	S	S	S	S
BK00092677_S4	South Africa	South Africa	LAM	S	S	S	S	F	S	S	S
BF01192128_S30	South Africa	South Africa	lineage4	S	S	S	S	S	S	S	S
TRL0116084_S13	South Africa	South Africa	Beijing	n/a	R	S	n/a	U	R	S	S
CD00793789_S24	South Africa	South Africa	Beijing	S	S	S	S	S	S	S	F
RJ00284466_S16	South Africa	South Africa	X-type	S	S	S	S	S	S	S	S
DH00743562_S11	South Africa	South Africa	LAM	S	S	S	S	S	S	S	S
DA00256304_S13	South Africa	South Africa	Beijing	R	S	S	S	F	S	S	S
TRL0114661_S4	South Africa	South Africa	X-type	n/a	R	R	n/a	R	R	R	U
BD00610612_S1	South Africa	South Africa	LAM	S	S	S	S	S	S	S	S
DH00730364_S4	South Africa	South Africa	LAM	S	S	S	S	S	S	S	S
TRL0114831_S7	South Africa	South Africa	LAM	n/a	R	R	n/a	R	R	R	R
TRL0113702_S19	South Africa	South Africa	EAI	R	R	R	R	R	R	R	R
BF01315327_S26	South Africa	South Africa	Beijing	S	S	S	S	S	S	S	S
BF01135051_S31	South Africa	South Africa	LAM	S	S	S	S	S	S	S	S
QH00194828_S9	South Africa	South Africa	LAM	R	R	S	S	R	R	S	U
TRL0114477_S10	South Africa	South Africa	EAI	n/a	R	R	n/a	R	R	R	R
CG01771594_S24	South Africa	South Africa	Beijing	S	S	S	S	S	S	S	S
FD00253167_S20	South Africa	South Africa	lineage4	S	S	S	S	F	S	F	S
BT00031585_S12	South Africa	South Africa	Beijing	S	S	S	S	S	S	S	S
TRL0115659_S4	South Africa	South Africa	X-type	S	S	S	n/a	S	S	S	S
BF01114065_S29	South Africa	South Africa	Beijing	S	S	S	S	F	S	S	S
BT00025770_S31	South Africa	South Africa	LAM	S	S	S	S	S	S	U	S
CE01540942_S1	South Africa	South Africa	Tur	S	S	S	S	S	S	S	S
TRL0114480_S11	South Africa	South Africa	Beijing	n/a	R	R	n/a	R	R	R	R
CE01611555_S31	South Africa	South Africa	Beijing	S	S	S	S	S	S	S	S
TRL0115445_S4	South Africa	South Africa	Beijing	n/a	R	S	n/a	S	R	S	S
EB00197215_S29	South Africa	South Africa	LAM	S	S	S	S	S	S	S	S
BF01473247_S18	South Africa	South Africa	S-type	S	S	S	S	S	S	S	S
PM00233485_S12	South Africa	South Africa	Beijing	S	S	S	S	S	S	S	S
ED00943149_S23	South Africa	South Africa	Beijing	S	S	S	S	S	U	S	S
BF01503171_S19	South Africa	South Africa	Haarlem	S	S	S	S	S	S	S	S
CE01632305_S30	South Africa	South Africa	LAM	S	S	S	S	S	S	S	S

CD00789586_S20	South Africa	South Africa	S-type	S	S	S	S	S	S	S	S
GH00382137_S24	South Africa	South Africa	lineage4	S	S	S	S	S	S	S	S
CH00361136_S9	South Africa	South Africa	LAM	S	S	S	S	S	S	S	S
GH00353950_S29	South Africa	South Africa	S-type	S	S	S	S	S	S	S	S
BR00088636_S3	South Africa	South Africa	Beijing	S	S	S	S	S	S	S	F
BF01375020_S28	South Africa	South Africa	LAM	S	S	S	S	S	S	S	S
BF01428659_S30	South Africa	South Africa	S-type	S	S	S	S	S	S	S	S
TRL0115096_S13	South Africa	South Africa	X-type	n/a	R	R	n/a	R	R	R	U
DA00307287_S20	South Africa	South Africa	S-type	S	S	S	S	S	S	S	S
CG01490105_S17	South Africa	South Africa	Beijing	S	S	S	S	S	S	S	S
DA00304536_S14	South Africa	South Africa	Beijing	S	S	S	S	S	S	S	S
BE01478470_S22	South Africa	South Africa	lineage4	S	S	S	S	S	S	S	S
DH00649810_S10	South Africa	South Africa	Beijing	S	S	S	S	S	S	S	F
BT00026180_S25	South Africa	South Africa	X-type	S	S	S	S	S	S	S	S
TRL0114774_S16	South Africa	South Africa	Beijing	n/a	R	R	n/a	R	R	R	R
BK00092078_S32	South Africa	South Africa	S-type	R	n/a	S	n/a	R	S	S	S
TRL0115669_S18	South Africa	South Africa	Beijing	n/a	R	R	n/a	R	R	R	R
FD00247663_S28	South Africa	South Africa	lineage4	S	S	S	S	S	S	S	S
BQ00027337_S11	South Africa	South Africa	S-type	R	S	S	n/a	R	S	U	S
DH00618155_S28	South Africa	South Africa	Haarlem	S	S	S	S	S	S	S	S
TRL0114962_S4	South Africa	South Africa	Beijing	n/a	R	R	n/a	R	R	R	R
BT00031791_S14	South Africa	South Africa	S-type	S	S	S	S	S	S	S	S
CG01593846_S4	South Africa	South Africa	Beijing	S	S	S	S	S	S	S	S
DG00561814_S22	South Africa	South Africa	LAM	R	S	S	S	R	S	S	S
DH00741562_S9	South Africa	South Africa	lineage4	S	S	S	S	S	S	S	S
BF01421069_S15	South Africa	South Africa	LAM	S	S	S	S	U	S	S	S
CE01772649_S13	South Africa	South Africa	Beijing	R	S	S	S	S	S	S	S
BT00028618_S7	South Africa	South Africa	Delhi	S	S	S	S	S	S	S	S
BN00034640_S29	South Africa	South Africa	Haarlem	S	S	S	S	S	S	S	S
BD00703460_S15	South Africa	South Africa	Beijing	S	S	S	S	S	F	S	S
BQ00029771_S27	South Africa	South Africa	LAM	S	S	S	S	S	S	S	S
BF01330971_S12	South Africa	South Africa	X-type	S	S	S	S	S	S	U	S
PL00097805_S26	South Africa	South Africa	LAM	R	R	S	S	R	R	S	S
EI00623322_S7	South Africa	South Africa	lineage4	R	S	S	S	U	S	S	S
CH00387331_S11	South Africa	South Africa	Beijing	S	S	S	S	S	S	S	S
BK00081000_S18	South Africa	South Africa	Beijing	S	S	S	S	S	F	S	S
BD00678576_S22	South Africa	South Africa	LAM	S	S	S	S	S	S	S	S
PO00209791_S11	South Africa	South Africa	Haarlem	S	R	S	S	S	R	S	S
TRL0060765_S13	South Africa	South Africa	S-type	S	R	S	S	S	R	S	S
DH00634083_S30	South Africa	South Africa	Beijing	R	S	S	S	R	S	S	S
PS00217691_S10	South Africa	South Africa	LAM	R	R	S	S	R	R	S	S
TRL0115383_S15	South Africa	South Africa	Beijing	n/a	R	S	n/a	F	R	U	S
BT00029097_S19	South Africa	South Africa	Haarlem	S	S	S	S	S	S	S	S
DH00680833_S20	South Africa	South Africa	Beijing	S	S	S	S	S	S	S	S
BF01202447_S12	South Africa	South Africa	lineage4	S	S	S	S	S	S	S	S
TRL0114616_S24	South Africa	South Africa	Beijing	n/a	R	R	n/a	R	R	R	R
GH00363341_S30	South Africa	South Africa	lineage4	S	S	S	S	S	S	S	S
CH00341127_S8	South Africa	South Africa	LAM	S	S	S	S	S	S	S	S
TRL0114833_S12	South Africa	South Africa	Beijing	n/a	R	R	n/a	R	R	R	R
QE00098167_S8	South Africa	South Africa	X-type	R	R	S	S	R	R	U	S
CH00420650_S30	South Africa	South Africa	LAM	S	S	S	S	S	S	S	S
JG00406968_S6	South Africa	South Africa	X-type	S	S	S	S	S	S	S	S
BN00037979_S5	South Africa	South Africa	Beijing	S	S	S	S	S	S	S	S
TRL0115868_S7	South Africa	South Africa	X-type	n/a	S	R	n/a	R	R	R	U
BH00946140_S18	South Africa	South Africa	Beijing	S	S	S	S	S	S	S	S
TRL0103669_S13	South Africa	South Africa	Beijing	R	R	S	S	R	R	S	S
BK00079668_S19	South Africa	South Africa	LAM	S	S	S	S	S	S	S	S
BF01355975_S30	South Africa	South Africa	X-type	S	S	S	S	S	S	S	S
BQ00030933_S4	South Africa	South Africa	LAM	S	S	S	S	S	S	S	S
TRL0116109_S14	South Africa	South Africa	EAI	n/a	S	S	n/a	S	S	S	S
BH00794082_S26	South Africa	South Africa	S-type	S	S	S	S	S	S	S	S
BT00033627_S20	South Africa	South Africa	lineage4	S	S	S	S	F	S	S	S
BR00096736_S27	South Africa	South Africa	X-type	R	R	S	S	R	R	S	S
TRL0065056_S25	South Africa	South Africa	LAM	S	R	S	S	S	S	S	S
EI00666607_S29	South Africa	South Africa	Delhi	S	S	S	S	F	S	S	F
BQ00027771_S12	South Africa	South Africa	Beijing	S	S	S	S	S	S	S	S
DA00306193_S14	South Africa	South Africa	Beijing	S	S	S	S	S	S	S	S
BE01379864_S15	South Africa	South Africa	Beijing	S	S	S	S	S	S	S	S
BF01274561_S17	South Africa	South Africa	Beijing	S	S	S	S	S	S	S	S
BD00629308_S32	South Africa	South Africa	Beijing	S	S	S	S	S	S	S	S
ED00909054_S12	South Africa	South Africa	LAM	S	S	S	S	S	S	S	S
TRL0066006_S26	South Africa	South Africa	X-type	S	R	S	S	S	S	S	S
DH00605289_S27	South Africa	South Africa	LAM	S	S	S	S	S	S	S	S
BF01355978_S31	South Africa	South Africa	Haarlem	S	S	S	S	S	S	S	S
DH00680829_S13	South Africa	South Africa	LAM	R	S	S	S	S	S	S	S
BN00045767_S7	South Africa	South Africa	Beijing	S	S	S	S	S	S	S	S
GH00373073_S9	South Africa	South Africa	LAM	S	S	S	S	S	S	S	S
BF01175378_S5	South Africa	South Africa	Haarlem	S	S	S	S	S	S	S	S
BT00029901_S26	South Africa	South Africa	S-type	S	S	S	S	S	S	S	S
TRL0115581_S15	South Africa	South Africa	Beijing	n/a	R	R	n/a	R	R	R	R
DH00625221_S26	South Africa	South Africa	S-type	S	S	S	S	S	S	U	S
JG00432562_S9	South Africa	South Africa	lineage4	S	S	S	S	S	S	S	S
CD00970213_S4	South Africa	South Africa	Beijing	S	S	S	S	S	S	S	S
DG00618197_S25	South Africa	South Africa	Beijing	S	S	S	S	S	S	S	S
TRL0112877_S20	South Africa	South Africa	LAM	R	R	S	R	R	R	S	R
GH00389342_S1	South Africa	South Africa	Beijing	S	S	S	S	S	S	S	S
CG01554911_S5	South Africa	South Africa	LAM	S	S	S	S	S	S	S	S
BK00088728_S25	South Africa	South Africa	LAM	S	S	S	S	S	S	S	S
GH00350830_S17	South Africa	South Africa	LAM	S	R	S	S	S	R	S	S
BF01483411_S2	South Africa	South Africa	LAM	S	S	S	S	S	S	S	S
WG00050823_S8	South Africa	South Africa	S-type	S	S	S	S	S	S	S	S
TRL0114792_S4	South Africa	South Africa	Beijing	n/a	R	R	n/a	R	R	R	R
TRL0116715_S9	South Africa	South Africa	Beijing	S	R	S	n/a	S	R	S	S
BH00824877_S8	South Africa	South Africa	X-type	S	S	S	S	S	S	S	S
EF00280850_S29	South Africa	South Africa	Beijing	S	S	S	S	S	S	S	S
EI00660938_S25	South Africa	South Africa	lineage4	S	S	S	S	S	S	S	S
TRL0113671_S18	South Africa	South Africa	LAM	S	R	S	S	S	R	S	S
CG01279230_S31	South Africa	South Africa	LAM	S	S	S	S	S	S	S	S
PK00599668_S16	South Africa	South Africa	LAM	R	R	R	S	R	R	R	S
TRL0116115_S15	South Africa	South Africa	LAM	n/a	S	S	n/a	S	S	S	S
BK00093448_S32	South Africa	South Africa	LAM	S	S	S	S	S	S	S	S
TRL0114658_S14	South Africa	South Africa	Beijing	n/a	R	R	n/a	R	R	R	R
BF01310203_S13	South Africa	South Africa	lineage4	S	S	S	S	S	S	S	S
BF01318054_S23	South Africa	South Africa	S-type	S	S	S	S	S	S	S	S
BF01196907_S14	South Africa	South Africa	LAM	S	S	S	S	S	S	S	S
TRL0115030_S10	South Africa	South Africa	LAM	n/a	R	R	n/a	R	R	R	R
BE01207626_S14	South Africa	South Africa	Beijing	S	S	S	S	S	S	S	S
TRL0115633_S3	South Africa	South Africa	lineage4	n/a	R	R	n/a	R	R	R	U
BF01216702_S16	South Africa	South Africa	LAM	S	S	S	S	S	S	S	S
GH00389343_S18	South Africa	South Africa	lineage4	S	S	S	S	S	S	S	S
DG00530312_S20	South Africa	South Africa	lineage4	S	S	S	S	S	S	S	S
BD00490075_S5	South Africa	South Africa	Beijing	S	S	S	S	S	S	S	S
TRL0116726_S16	South Africa	South Africa	lineage4	R	R	S	n/a	R	F	S	S
DH00634109_S16	South Africa	South Africa	LAM	S	S	S	S	S	S	S	S
TRL0113569_S45	South Africa	South Africa	Beijing	R	R	R	R	R	R	R	R
TRL0113098_S23	South Africa	South Africa	S-type	R	R	R	S	R	R	R	S

BD00703455_S16	South Africa	South Africa	Beijing	S	S	S	S	S	S	S	S
PE00190349_S18	South Africa	South Africa	LAM	R	R	R	S	R	R	R	R
TRL0056535_S30	South Africa	South Africa	Haarlem	R	R	S	S	R	R	U	S
TRL0112827_S19	South Africa	South Africa	LAM	R	R	S	S	R	R	S	S
BE01487154_S24	South Africa	South Africa	LAM	S	S	S	S	S	S	S	S
BD00514520_S1	South Africa	South Africa	Beijing	S	S	S	S	S	S	S	S
BE01100694_S7	South Africa	South Africa	LAM	S	S	S	S	S	S	S	S
BE01141152_S9	South Africa	South Africa	Beijing	S	S	S	S	S	S	S	S
BF01492006_S20	South Africa	South Africa	Beijing	S	S	S	n/a	S	S	S	S
BE01371373_S21	South Africa	South Africa	lineage4	S	S	S	S	S	S	S	S
DG00611914_S7	South Africa	South Africa	Delhi	S	S	R	S	S	S	S	S
BN00040586_S18	South Africa	South Africa	Beijing	S	S	S	S	S	S	S	S
BF01330879_S10	South Africa	South Africa	Beijing	S	S	S	S	S	S	S	S
RF00127752_S27	South Africa	South Africa	LAM	R	R	R	R	R	R	R	R
TRL0109157_S20	South Africa	South Africa	Beijing	R	R	R	S	R	R	R	S
JG00224441_S4	South Africa	South Africa	lineage4	R	R	R	S	R	R	R	U
DH00625230_S28	South Africa	South Africa	LAM	S	S	S	S	S	S	S	S
TRL0113005_S21	South Africa	South Africa	S-type	R	R	S	R	R	R	R	R
BH00794084_S5	South Africa	South Africa	lineage4	S	S	S	S	S	S	S	S
CE01538627_S9	South Africa	South Africa	S-type	S	S	S	S	S	F	S	S
BE01314329_S19	South Africa	South Africa	LAM	S	S	S	S	S	S	S	S
EI00664567_S21	South Africa	South Africa	LAM	R	R	S	S	R	R	S	S
BE01284388_S18	South Africa	South Africa	lineage4	S	S	S	R	S	S	S	S
BK00079216_S7	South Africa	South Africa	S-type	S	S	S	S	S	S	U	S
BF01178152_S11	South Africa	South Africa	S-type	S	S	S	S	S	S	S	S
BK00091915_S29	South Africa	South Africa	LAM	S	S	S	S	S	S	S	S
OC00001135_S5	South Africa	South Africa	X-type	S	S	S	S	S	S	S	S
PK00685617_S23	South Africa	South Africa	EAI	R	R	R	S	R	R	R	R
TRL0105138_S19	South Africa	South Africa	Beijing	S	S	S	S	S	S	S	S
CG01388002_S15	South Africa	South Africa	LAM	S	S	S	S	S	S	S	S
TRL0115197_S15	South Africa	South Africa	Beijing	n/a	R	R	n/a	R	R	R	R
CE01907630_S9	South Africa	South Africa	S-type	S	S	S	S	S	S	S	S
BR00093908_S21	South Africa	South Africa	lineage4	S	S	S	S	S	S	S	S
EF00225306_S28	South Africa	South Africa	LAM	S	S	S	S	S	S	S	S
JD00110569_S17	South Africa	South Africa	lineage4	S	R	S	S	R	R	S	S
PP00150885_S15	South Africa	South Africa	LAM	S	S	S	S	S	S	S	S
TRL0116187_S19	South Africa	South Africa	lineage4	n/a	R	R	n/a	R	R	R	R
DG00593793_S15	South Africa	South Africa	Beijing	S	S	S	S	S	S	S	S
ED00958949_S30	South Africa	South Africa	LAM	S	S	S	S	S	S	S	S
BR00072530_S18	South Africa	South Africa	Beijing	S	S	S	R	S	S	S	S
BR00079236_S18	South Africa	South Africa	EAI	S	S	S	S	S	S	S	S
BH00815106_S7	South Africa	South Africa	Haarlem	R	S	S	S	S	S	S	S
BT00028780_S21	South Africa	South Africa	S-type	S	S	S	S	S	S	S	S
TRL0108514_S6	South Africa	South Africa	lineage4	R	R	R	S	R	R	R	U
TRL0083468_S23	South Africa	South Africa	LAM	S	S	S	S	S	S	S	F
CD00831317_S26	South Africa	South Africa	Beijing	S	S	S	S	S	S	S	S
EF00273309_S5	South Africa	South Africa	lineage4	S	S	S	S	S	S	S	S
PJ00162888_S8	South Africa	South Africa	Haarlem	R	R	R	R	R	R	R	R
TRL0085353_S11	South Africa	South Africa	S-type	S	R	S	S	S	S	S	S
ED00957471_S19	South Africa	South Africa	LAM	R	S	S	S	S	S	U	S
DH00649794_S9	South Africa	South Africa	X-type	S	S	S	S	F	S	U	S
BF01506396_S17	South Africa	South Africa	EAI	S	S	S	S	S	S	S	S
BF01381848_S13	South Africa	South Africa	Beijing	S	S	S	S	S	S	S	S
TRL0116880_S12	South Africa	South Africa	LAM	n/a	R	R	n/a	R	R	R	R
BH00904043_S11	South Africa	South Africa	EAI	S	S	S	S	S	S	U	S
TRL0114479_S10	South Africa	South Africa	X-type	n/a	R	R	n/a	R	R	R	U
FD00212241_S26	South Africa	South Africa	lineage4	S	S	S	S	S	S	S	S
BN00037636_S26	South Africa	South Africa	lineage4	S	S	S	S	S	S	S	S
BR00093118_S28	South Africa	South Africa	Haarlem	R	S	S	S	F	S	S	S
BF01386435_S10	South Africa	South Africa	Haarlem	S	S	S	S	S	S	S	S
TRL0031160_S6	South Africa	South Africa	lineage4	R	R	R	R	R	R	R	R
TRL0114667_S2	South Africa	South Africa	Beijing	n/a	R	R	n/a	R	R	R	R
TRL0113095_S10	South Africa	South Africa	S-type	R	R	R	R	R	R	R	R
BF01175387_S7	South Africa	South Africa	S-type	S	S	S	S	S	S	S	S
BF01522222_S19	South Africa	South Africa	LAM	S	S	S	S	F	F	S	S
EN00013592_S25	South Africa	South Africa	LAM	S	S	S	S	S	S	S	S
TRL0108114_S4	South Africa	South Africa	LAM	R	R	S	S	R	R	S	S
BR00092662_S34	South Africa	South Africa	LAM	S	S	S	S	S	S	S	S
PK00651442_S30	South Africa	South Africa	Beijing	R	R	S	S	R	R	R	S
TRL0113469_S14	South Africa	South Africa	LAM	R	R	S	S	R	R	S	S
TRL0114829_S6	South Africa	South Africa	Beijing	n/a	R	R	n/a	R	R	R	R
BH00936808_S8	South Africa	South Africa	Beijing	S	R	S	S	S	F	S	S
TRL0099205_S15	South Africa	South Africa	LAM	S	R	S	S	S	R	S	S
BN00042248_S28	South Africa	South Africa	LAM	R	R	S	R	R	R	R	R
BF01321448_S14	South Africa	South Africa	Beijing	S	S	S	S	S	S	S	S
TRL0113487_S15	South Africa	South Africa	LAM	R	R	S	S	R	R	R	S
BE01203293_S12	South Africa	South Africa	Beijing	R	S	S	S	R	S	S	S
TRL0115283_S19	South Africa	South Africa	Beijing	n/a	R	R	n/a	R	R	R	R
BF01311702_S23	South Africa	South Africa	Haarlem	S	S	S	S	S	S	S	S
TRL0116593_S24	South Africa	South Africa	Beijing	n/a	R	R	n/a	R	R	R	F
BE01457107_S22	South Africa	South Africa	S-type	S	S	S	S	S	S	S	S
BD00561502_S18	South Africa	South Africa	LAM	S	S	S	S	S	S	S	S
TRL0114821_S5	South Africa	South Africa	Haarlem	n/a	R	R	n/a	R	R	R	R
TRL0117008_S16	South Africa	South Africa	Beijing	R	R	R	n/a	R	R	R	U
TRL0115846_S10	South Africa	South Africa	Beijing	n/a	R	R	n/a	R	R	R	R
TRL0103300_S15	South Africa	South Africa	X-type	R	R	S	S	R	R	R	S
BT00031965_S16	South Africa	South Africa	Beijing	S	S	S	S	S	S	S	S
BR00093905_S11	South Africa	South Africa	LAM	S	S	S	S	S	S	S	S
TRL0115018_S8	South Africa	South Africa	Haarlem	n/a	R	R	n/a	R	R	R	R
TRL0090417_S19	South Africa	South Africa	Beijing	R	R	R	S	R	R	R	S
TRL0023642_S1	South Africa	South Africa	X-type	R	R	R	R	R	R	R	R
DG00518772_S15	South Africa	South Africa	LAM	S	S	S	R	S	S	S	S
BF01236695_S29	South Africa	South Africa	S-type	R	S	S	R	R	S	S	R
CD00792640_S24	South Africa	South Africa	lineage4	S	S	S	S	S	S	S	S
TRL0086755_S25	South Africa	South Africa	X-type	S	R	S	S	S	R	S	S
JA00225792_S1	South Africa	South Africa	lineage4	S	S	S	S	S	S	S	S
TRL0112235_S18	South Africa	South Africa	LAM	R	R	R	R	R	R	R	R
EB00188206_S4	South Africa	South Africa	Beijing	S	S	S	S	S	S	S	S
BF01330972_S27	South Africa	South Africa	Beijing	S	S	S	S	S	S	S	S
TRL0113496_S6	South Africa	South Africa	lineage4	S	R	S	S	S	R	S	S
BQ00026414_S10	South Africa	South Africa	X-type	S	S	S	S	S	S	S	S
BF01445513_S21	South Africa	South Africa	lineage4	S	S	S	S	S	S	S	S
CD00937617_S4	South Africa	South Africa	LAM	R	S	S	S	R	S	S	S
BF01407000_S1	South Africa	South Africa	Beijing	R	S	S	S	R	S	S	S
EI00662151_S13	South Africa	South Africa	Beijing	S	S	S	S	S	S	S	S
CG01633852_S29	South Africa	South Africa	Beijing	S	S	S	S	S	S	S	F
TRL0115368_S21	South Africa	South Africa	S-type	n/a	S	S	n/a	S	S	F	S
DH00742250_S25	South Africa	South Africa	X-type	S	S	S	S	S	S	S	S
BF01199882_S15	South Africa	South Africa	lineage4	R	R	S	R	R	R	U	R
BF01481932_S24	South Africa	South Africa	LAM	S	S	S	S	S	S	S	S
TRL0115039_S11	South Africa	South Africa	Haarlem	n/a	R	R	n/a	R	R	R	R
BE01194858_S11	South Africa	South Africa	Beijing	S	S	S	S	S	S	S	S
BK00077058_S14	South Africa	South Africa	S-type	R	R	R	R	R	R	R	R
CE01786354_S3	South Africa	South Africa	lineage4	S	S	S	S	S	S	S	S
TRL0107570_S5	South Africa	South Africa	Beijing	R	R	R	n/a	R	R	R	S
DG00683532_S26	South Africa	South Africa	Delhi	R	S	S	R	R	S	S	U

GH00346926_S27	South Africa	South Africa	LAM		S	S	S	S	S	S	S	S
DH00692417_S9	South Africa	South Africa	LAM		S	S	S	S	S	S	S	S
BD00483478_S32	South Africa	South Africa			S	S	S	S	S	S	S	S
BD00708941_S17	South Africa	South Africa	lineage4		S	S	S	S	S	S	S	S
TRL0115404_S16	South Africa	South Africa	Beijing		n/a	R	R	n/a	R	R	R	R
CG01593892_S13	South Africa	South Africa	lineage4		S	S	S	S	S	S	S	S
TRL0113733_S20	South Africa	South Africa	EAI		R	R	R	R	R	R	R	R
BR00095142_S20	South Africa	South Africa	LAM		S	S	S	S	S	S	S	S
CD00922304_S1	South Africa	South Africa	LAM		S	S	S	n/a	S	S	S	S
TRL0113580_S17	South Africa	South Africa	LAM		R	R	S	S	R	R	R	S
BT00030991_S5	South Africa	South Africa	X-type		S	S	S	S	S	S	S	S
DG00502193_S9	South Africa	South Africa	Beijing		S	S	S	S	S	S	S	S
TRL0114137_S20	South Africa	South Africa	Beijing		n/a	R	R	n/a	R	R	R	R
TRL0115115_S14	South Africa	South Africa	Beijing		n/a	R	R	n/a	R	R	R	R
BH00952428_S22	South Africa	South Africa	S-type		S	S	S	S	S	S	S	S
TRL0115694_S19	South Africa	South Africa	Beijing		n/a	R	R	n/a	R	R	R	R
PM00210785_S30	South Africa	South Africa	lineage4		R	R	R	S	R	F	F	F
TRL0116883_S13	South Africa	South Africa	lineage4		n/a	R	R	n/a	R	R	R	R
TRL0113022_S22	South Africa	South Africa	LAM		R	R	R	R	R	R	R	F
BF01457875_S14	South Africa	South Africa	lineage4		R	R	S	R	R	R	R	R
EF00227905_S30	South Africa	South Africa	lineage4		S	S	S	S	S	S	S	S
BE01453006_S31	South Africa	South Africa	Beijing		S	S	S	S	S	S	S	S
DH00668064_S31	South Africa	South Africa	LAM		R	S	S	S	R	S	S	S
DH00704921_S24	South Africa	South Africa	LAM		S	R	S	S	S	R	S	S
BR00079705_S3	South Africa	South Africa	EAI		S	S	S	S	S	S	S	S
CE01938357_S30	South Africa	South Africa	Haarlem		S	S	S	S	S	S	S	S
TRL0116692_S8	South Africa	South Africa	EAI		n/a	R	S	n/a	R	R	S	R
BF01227167_S25	South Africa	South Africa	Beijing		S	R	S	S	S	R	S	S
BD00734087_S18	South Africa	South Africa	Beijing		S	S	S	n/a	S	S	S	S
DG00618192_S12	South Africa	South Africa	Beijing		S	S	S	S	S	S	S	S
CG01404388_S4	South Africa	South Africa	X-type		S	S	S	S	S	S	S	S
DH00634104_S31	South Africa	South Africa	S-type		S	S	S	S	S	S	S	S
BE01468979_S17	South Africa	South Africa	LAM		S	S	S	S	S	S	S	S
EI00717034_S17	South Africa	South Africa	Beijing		S	S	S	S	S	S	S	S
CD00862519_S13	South Africa	South Africa	X-type		S	S	S	S	S	S	S	S
NC00179466_S10	South Africa	South Africa	lineage4		S	S	S	S	S	S	S	S
BN00046702_S6	South Africa	South Africa	Beijing		S	S	S	S	S	S	S	S
CG01461768_S32	South Africa	South Africa	lineage4		S	S	S	S	S	S	S	S
BT00023835_S10	South Africa	South Africa	LAM		S	S	S	S	S	S	S	S
TRL0039481_S20	South Africa	South Africa	Haarlem		S	R	S	S	S	R	S	S
VB00026760_S17	South Africa	South Africa	Haarlem		R	R	R	R	R	R	R	R
BF01375053_S16	South Africa	South Africa	X-type		S	S	S	S	S	S	S	S
BF01363898_S2	South Africa	South Africa	LAM		S	S	S	S	S	S	S	S
CH00418283_S25	South Africa	South Africa	Beijing		S	S	S	S	S	S	S	S
BN00045762_S25	South Africa	South Africa	Beijing		S	S	S	S	S	S	S	S
BR00091242_S29	South Africa	South Africa	LAM		S	S	S	S	S	S	S	S
UH00107527_S30	South Africa	South Africa	Beijing		S	R	S	S	S	R	S	S
BF01298698_S31	South Africa	South Africa	LAM		S	S	S	S	S	S	S	S
BH00936810_S26	South Africa	South Africa	S-type		S	S	S	S	S	S	S	S
BR00073167_S14	South Africa	South Africa	Beijing		S	S	S	S	S	S	S	S
BN00036996_S26	South Africa	South Africa	LAM		S	S	R	S	S	S	S	S
G188	Spain	Spain	EAI		S	S	S	S	S	S	S	S
G139	Spain	Spain	Haarlem		S	S	S	S	S	S	S	S
G641	Spain	Spain	LAM		S	S	S	S	S	S	U	S
G190	Spain	Spain	lineage4		S	S	S	S	S	U	S	S
G766	Spain	Spain	Haarlem		S	S	S	S	S	S	S	S
G768	Spain	Spain	lineage4		S	S	S	S	S	S	U	S
G909	Spain	Spain	Haarlem		S	S	S	S	S	S	S	S
G621	Spain	Spain	lineage4		S	S	S	S	S	S	S	S
G192	Spain	Spain	Haarlem		S	S	S	S	S	S	S	S
FE21	Spain	Spain	Haarlem		S	R	S	S	S	F	S	S
G350	Spain	Spain	LAM		S	S	S	S	S	S	U	S
G338	Spain	Spain	Haarlem		S	S	S	S	S	S	S	S
G637	Spain	Spain	lineage4		S	S	S	S	S	S	S	S
G634	Spain	Spain	lineage4		S	S	S	S	S	S	S	S
G630	Spain	Spain	Cameroon		S	S	S	S	S	S	S	S
G186	Spain	Spain	LAM		S	S	S	S	S	S	S	S
G358	Spain	Spain	Haarlem		S	S	S	S	S	S	S	S
G208	Spain	Spain	lineage4		S	S	S	S	S	S	S	S
G644	Spain	Spain	LAM		S	S	S	n/a	S	S	S	S
G183	Spain	Spain	lineage4		S	S	S	S	S	S	S	S
FE40	Spain	Spain	LAM		S	S	S	S	S	S	U	S
G143	Spain	Spain	lineage4		S	R	S	S	S	R	S	S
FE03	Spain	Spain	LAM		S	S	S	S	S	S	U	S
FE23	Spain	Spain	Haarlem		R	R	S	S	F	F	S	S
G191	Spain	Spain	lineage4		S	S	S	S	S	S	S	S
G773	Spain	Spain	Beijing		S	S	S	S	S	S	S	S
G348	Spain	Spain	LAM		S	S	S	S	S	S	S	S
G701	Spain	Spain	Haarlem		R	R	R	S	R	R	U	S
G195	Spain	Spain	Delhi		S	S	S	S	S	S	S	S
G725	Spain	Spain	lineage4		S	S	S	S	S	S	S	S
FE41	Spain	Spain	Haarlem		R	S	S	S	R	S	S	S
G202	Spain	Spain	LAM		S	S	S	S	S	S	S	S
G778	Spain	Spain	lineage4		S	S	S	S	S	S	S	S
FE29	Spain	Spain	LAM		S	S	S	R	S	S	S	S
FE34	Spain	Spain	lineage4		R	R	R	R	R	R	R	R
G356	Spain	Spain	Haarlem		S	S	S	S	S	U	S	S
FE24	Spain	Spain	Beijing		R	R	R	R	R	R	R	R
G594	Spain	Spain	LAM		S	S	S	S	S	S	S	S
G209	Spain	Spain	LAM		S	S	S	S	S	S	S	S
G180m	Spain	Spain	LAM		S	S	S	n/a	U	S	S	S
G204	Spain	Spain	Haarlem		S	S	S	S	S	S	S	S
FE20	Spain	Spain	Beijing		R	R	S	S	R	R	S	S
G182	Spain	Spain	Haarlem		S	S	S	S	S	S	S	S
FE14	Spain	Spain	Haarlem		S	S	S	S	S	S	U	S
G200	Spain	Spain	S-type		S	S	S	n/a	S	S	S	S
FE27	Spain	Spain	lineage4		S	S	S	S	S	S	S	S
G201	Spain	Spain	lineage4		S	S	S	S	S	S	S	S
G623	Spain	Spain	lineage4		S	S	S	S	S	S	S	S
G357	Spain	Spain	lineage4		R	S	S	S	U	S	S	S
FE37	Spain	Spain	Beijing		R	S	R	S	R	S	R	S
FE01	Spain	Spain	lineage4		R	R	S	S	R	R	S	S
G179	Spain	Spain	Delhi		R	S	S	S	R	S	S	S
G910	Spain	Spain	Haarlem		S	S	S	S	S	S	S	S
G142	Spain	Spain	LAM		S	S	S	S	S	S	S	S
G757	Spain	Spain	lineage4		S	S	S	S	S	S	S	S
G205	Spain	Spain	Haarlem		S	S	S	S	S	S	S	S
FE28	Spain	Spain	lineage4		R	S	S	S	R	S	S	S
G323	Spain	Spain	Haarlem		S	S	S	S	S	S	S	S
FE05	Spain	Spain	Beijing		R	R	S	S	R	R	U	S
FE36	Spain	Spain	LAM		R	R	R	R	R	R	U	U
G770	Spain	Spain	Ghana		S	S	S	S	S	S	S	S
G322	Spain	Spain	LAM		S	S	S	S	S	S	S	S
G196	Spain	Spain	LAM		S	S	S	S	S	S	S	S
G627	Spain	Spain	lineage4		S	S	S	S	S	S	U	S
8783-09	MSF	Swaziland	EAI	ERR553020 ERR	R	R	R	R	R	R	R	R

431-10	MSF	Swaziland	S-type	ERR552491	R	R	R	R	R	R	R	R
10453-09	MSF	Swaziland	LAM	ERR551231	R	R	S	S	U	F	F	S
7713-09	MSF	Swaziland	Beijing	ERR551650	R	R	R	S	R	R	R	S
6955-09	MSF	Swaziland	S-type	ERR552572 ERR	R	R	R	R	R	R	R	R
4347-09	MSF	Swaziland	EAI	ERR2199956	S	S	S	n/a	S	S	S	S
6243-09	MSF	Swaziland	X-type	ERR2200074	S	S	S	n/a	S	S	S	S
6085-09	MSF	Swaziland	lineage4	ERR2200067	S	S	S	n/a	S	S	S	S
5750-09	MSF	Swaziland	EAI	ERR2200048 ER	R	S	S	R	R	R	U	S
8585-09	MSF	Swaziland	LAM	ERR552789	R	R	S	R	R	R	R	R
5088-09	MSF	Swaziland	S-type	ERR2199994 ER	R	R	S	R	R	R	R	R
4727-09	MSF	Swaziland	X-type	ERR2199979	S	S	S	n/a	S	S	U	S
4298-09	MSF	Swaziland	S-type	ERR551194 ERR	R	n/a	R	R	R	R	R	R
1060-10	MSF	Swaziland	EAI	ERR2199774 ER	S	S	S	n/a	S	S	S	S
6059-09	MSF	Swaziland	X-type	ERR2200064 ER	R	R	R	R	R	R	R	U
118-10	MSF	Swaziland	LAM	ERR552221	R	S	S	S	R	S	S	S
1054-10	MSF	Swaziland	LAM	ERR498238	R	S	S	S	R	S	S	S
5093-09	MSF	Swaziland	EAI	ERR2199995	S	S	S	n/a	S	S	S	S
603-10	MSF	Swaziland	S-type	ERR552763	R	R	R	R	R	R	R	R
5582-09	MSF	Swaziland	Haarlem	ERR2200040	S	S	S	n/a	S	S	S	S
438-10	MSF	Swaziland	S-type	ERR553357	R	R	S	R	R	R	R	R
10301-09	MSF	Swaziland	lineage4	ERR2199758 ER	S	R	S	S	S	F	S	S
5111-09	MSF	Swaziland	Delhi	ERR2200002	S	S	S	n/a	S	S	S	S
5755-09	MSF	Swaziland	Haarlem	ERR552010	R	R	S	R	R	R	S	R
433-10	MSF	Swaziland	S-type	ERR552754	R	R	R	R	R	R	R	R
4723-09	MSF	Swaziland	S-type	ERR552083	R	n/a	R	R	R	R	R	R
7187-09	MSF	Swaziland	Beijing	ERR551979 ERR	S	S	S	n/a	S	S	S	S
4273-09	MSF	Swaziland	Haarlem	ERR2199936	S	S	S	n/a	S	S	S	S
5579-09	MSF	Swaziland	S-type	ERR552764 ERR	R	R	R	R	R	R	R	R
4344-09	MSF	Swaziland	LAM	ERR2199953 ER	S	S	S	n/a	S	S	U	S
10121-09	MSF	Swaziland	X-type	ERR2199750 ER	R	R	R	R	R	R	R	U
10702-09	MSF	Swaziland	X-type	ERR552074 ERR	R	R	R	n/a	R	R	R	U
7175-09	MSF	Swaziland	S-type	ERR552612	R	R	R	R	R	R	R	R
1057-10	MSF	Swaziland	LAM	ERR553350	R	R	R	S	R	R	U	S
4711-09	MSF	Swaziland	lineage4	ERR2199975	S	S	S	n/a	S	S	S	S
5126-09	MSF	Swaziland	LAM	ERR2200008	S	S	S	n/a	U	S	S	S
5099-09	MSF	Swaziland	Haarlem	ERR2199996	S	S	S	n/a	S	S	S	S
5106-09	MSF	Swaziland	EAI	ERR552115	R	R	R	R	R	R	R	R
10436-09	MSF	Swaziland	S-type	ERR2199766 ER	R	n/a	R	R	R	R	R	R
4285-09	MSF	Swaziland	X-type	ERR551941 ERR	S	S	S	n/a	S	S	S	S
5121-09	MSF	Swaziland	lineage4	ERR2200006	S	S	S	n/a	S	S	S	S
7186-09	MSF	Swaziland	lineage4	ERR553182	R	S	S	R	R	S	S	R
6070-09	MSF	Swaziland	X-type	ERR2200065	S	S	S	n/a	S	S	S	S
5079-09	MSF	Swaziland	Haarlem	ERR2199992	S	S	S	n/a	S	S	S	S
4724-09	MSF	Swaziland	S-type	ERR552821 ERR	R	n/a	R	R	R	R	R	R
1053-10	MSF	Swaziland	X-type	ERR550959	R	R	R	n/a	R	R	R	U
10452-09	MSF	Swaziland	S-type	ERR552253 ERR	R	R	R	R	R	R	R	R
4695-09	MSF	Swaziland	LAM	ERR2199970	S	S	S	n/a	U	S	S	S
6643-09	MSF	Swaziland	X-type	ERR2200087	S	S	S	n/a	S	S	S	S
4339-09	MSF	Swaziland	lineage4	ERR552710	R	R	S	R	R	R	R	R
5758-09	MSF	Swaziland	S-type	ERR552981	R	R	S	S	R	R	S	S
10298-09	MSF	Swaziland	S-type	ERR552875 ERR	R	R	R	R	R	R	R	R
4697-09	MSF	Swaziland	S-type	ERR2199972	S	S	S	n/a	S	S	S	S
5072-09	MSF	Swaziland	LAM	ERR2199989	S	S	S	n/a	U	S	S	S
5114-09	MSF	Swaziland	Beijing	ERR551545	R	R	R	R	R	R	R	U
11237-09	MSF	Swaziland	EAI	ERR551739	R	R	R	R	R	R	R	R
4256-09	MSF	Swaziland	X-type	ERR2199924	S	S	S	n/a	S	S	S	S
5556-09	MSF	Swaziland	LAM	ERR2200038	S	S	S	n/a	S	S	S	S
4255-09	MSF	Swaziland	EAI	ERR2199923	S	S	S	n/a	S	S	S	S
5752-09	MSF	Swaziland	LAM	ERR2200049	S	S	S	n/a	R	S	S	S
11238-09	MSF	Swaziland	EAI	ERR552000	R	R	R	R	R	R	R	R
10438-09	MSF	Swaziland	S-type	ERR552405	R	R	R	R	R	R	R	R
4269-09	MSF	Swaziland	Beijing	ERR2199932	S	S	S	n/a	S	S	S	S
5130-09	MSF	Swaziland	EAI	ERR2200009	S	S	S	n/a	S	S	U	S
10706-09	MSF	Swaziland	X-type	ERR2199778 ER	R	R	R	n/a	R	R	R	U
9085-09	MSF	Swaziland	S-type	ERR551298 ERR	R	R	S	S	R	R	S	S
10292-09	MSF	Swaziland	X-type	ERR552619 ERR	S	S	S	n/a	S	S	S	S
5105-09	MSF	Swaziland	LAM	ERR2199999	S	S	S	n/a	S	S	S	S
9562-09	MSF	Swaziland	X-type	ERR550863 ERR	R	R	R	R	R	R	R	U
7173-09	MSF	Swaziland	X-type	ERR553342 ERR	R	R	R	n/a	R	R	R	U
8786-09	MSF	Swaziland	Haarlem	ERR550657	R	S	S	S	R	S	S	S
4258-09	MSF	Swaziland	LAM	ERR2199926	S	S	S	n/a	S	S	S	S
11015-09	MSF	Swaziland	S-type	ERR550861	R	R	R	R	R	R	R	R
4253-09	MSF	Swaziland	LAM	ERR2199922	S	S	S	n/a	S	S	S	S
4267-09	MSF	Swaziland	S-type	ERR2199930	S	S	S	n/a	S	S	S	S
4713-09	MSF	Swaziland	X-type	ERR553105	R	R	R	n/a	R	R	R	U
604-10	MSF	Swaziland	Beijing	ERR553241	S	R	S	S	S	R	S	S
5250-09	MSF	Swaziland	Beijing	ERR2200015	S	S	S	n/a	S	S	S	S
4691-09	MSF	Swaziland	EAI	ERR2199969	S	S	S	n/a	S	S	S	S
5083-09	MSF	Swaziland	LAM	ERR2199993	S	S	S	n/a	S	S	S	S
1207-10	MSF	Swaziland	Beijing	ERR2199807	S	S	S	n/a	S	S	S	S
8313-09	MSF	Swaziland	LAM	ERR552989 ERR	R	R	S	S	R	R	S	S
4688-09	MSF	Swaziland	Beijing	ERR551079	S	S	S	n/a	S	S	S	S
10451-09	MSF	Swaziland	S-type	ERR552672	R	R	R	R	R	R	R	R
5100-09	MSF	Swaziland	lineage4	ERR2199997 ER	R	R	R	R	R	R	R	R
4268-09	MSF	Swaziland	LAM	ERR2199931	S	S	S	n/a	U	S	S	S
6948-09	MSF	Swaziland	X-type	ERR2200096	S	S	S	n/a	S	S	S	S
117-10	MSF	Swaziland	Haarlem	ERR553053	R	R	R	R	R	R	R	R
9080-09	MSF	Swaziland	S-type	ERR551536	R	R	R	R	R	R	R	R
4731-09	MSF	Swaziland	S-type	ERR552396 ERR	S	S	S	n/a	S	S	S	S
8318-09	MSF	Swaziland	X-type	ERR551240 ERR	R	R	R	n/a	R	R	R	U
11016-09	MSF	Swaziland	X-type	ERR552720	R	R	R	n/a	R	R	R	U
110-10	MSF	Swaziland	EAI	ERR2199787	S	S	S	n/a	S	S	U	S
5741-09	MSF	Swaziland	lineage4	ERR552929	R	S	S	S	R	S	S	S
5572-09	MSF	Swaziland	lineage4	ERR2200039	S	S	S	n/a	S	S	S	S
8779-09	MSF	Swaziland	X-type	ERR552916 ERR	R	R	R	n/a	R	R	R	U
10513-09	MSF	Swaziland	LAM	ERR2199771	S	S	S	n/a	S	S	S	S
8592-09	MSF	Swaziland	LAM	ERR2200119	S	S	S	n/a	R	S	S	S
6078-09	MSF	Swaziland	Beijing	ERR2200066 ER	R	R	S	R	R	R	R	R
7704-09	MSF	Swaziland	X-type	ERR552151	R	R	S	R	R	R	R	U
6251-09	MSF	Swaziland	Beijing	ERR2200077	S	S	S	n/a	S	S	S	S
4283-09	MSF	Swaziland	Haarlem	ERR551045	R	R	S	R	R	R	S	R
5122-09	MSF	Swaziland	lineage4	ERR2200007	S	S	S	n/a	S	S	S	S
8303-09	MSF	Swaziland	EAI	ERR553134	R	R	R	R	R	R	R	R
5737-09	MSF	Swaziland	Beijing	ERR2200043	S	S	S	n/a	S	S	S	S
1202-10	MSF	Swaziland	X-type	ERR551692	R	R	R	R	R	R	R	R
7178-09	MSF	Swaziland	X-type	ERR552361	R	R	R	n/a	R	R	R	U
5102-09	MSF	Swaziland	LAM	ERR2199998	S	S	S	n/a	S	S	S	S
5268-09	MSF	Swaziland	X-type	ERR2200019	S	S	S	n/a	S	S	U	S
10704-09	MSF	Swaziland	S-type	ERR551280	R	R	R	R	R	R	R	R
4722-09	MSF	Swaziland	Beijing	ERR2199977	S	S	S	n/a	S	S	S	S
4293-09	MSF	Swaziland	EAI	ERR2199942	S	S	S	n/a	S	S	U	S
5246-09	MSF	Swaziland	S-type	ERR551242	R	R	R	R	R	R	R	R
4696-09	MSF	Swaziland	EAI	ERR2199971	S	S	S	n/a	S	S	S	S
6949-09	MSF	Swaziland	S-type	ERR551757 ERR	R	R	R	R	R	R	R	R
5108-09	MSF	Swaziland	X-type	ERR2200001	S	S	S	n/a	S	S	S	S
10505-09	MSF	Swaziland	X-type	ERR551246	R	R	R	n/a	R	R	R	U

8302-09	MSF	Swaziland	X-type	ERR550813	R	R	R	n/a	R	R	R	U
8587-09	MSF	Swaziland	Haarlem	ERR2200118	S	S	S	n/a	S	S	S	S
5264-09	MSF	Swaziland	LAM	ERR2200018	S	S	S	n/a	S	S	S	S
596-10	MSF	Swaziland	X-type	ERR551393	R	R	R	n/a	R	R	R	U
4277-09	MSF	Swaziland	Beijing	ERR2199938	S	S	S	n/a	S	S	S	S
5243-09	MSF	Swaziland	Beijing	ERR2200014	S	S	S	n/a	S	S	S	S
7185-09	MSF	Swaziland	S-type	ERR552124	R	R	R	R	R	R	R	R
5549-09	MSF	Swaziland	Haarlem	ERR2200034	S	S	S	n/a	S	S	S	S
4296-09	MSF	Swaziland	Beijing	ERR551854 ERR	S	S	S	n/a	S	S	S	S
5256-09	MSF	Swaziland	lineage4	ERR2200017	S	S	S	n/a	S	S	S	S
8316-09	MSF	Swaziland	EAI	ERR2200115	S	S	S	n/a	S	U	U	S
10701-09	MSF	Swaziland	S-type	ERR552870	R	n/a	R	R	R	R	R	R
4730-09	MSF	Swaziland	LAM	ERR2199981	S	S	S	n/a	U	S	S	S
7716-09	MSF	Swaziland	X-type	ERR553185 ERR	R	R	R	n/a	R	R	R	U
10293-09	MSF	Swaziland	X-type	ERR552295 ERR	R	R	R	n/a	R	R	R	U
11020-09	MSF	Swaziland	lineage4	ERR551456	R	R	S	R	R	R	S	R
6266-09	MSF	Swaziland	LAM	ERR552071	R	S	S	S	R	S	S	S
9565-09	MSF	Swaziland	X-type	ERR2200139	S	S	S	n/a	S	S	S	S
5274-09	MSF	Swaziland	Haarlem	ERR550872	R	S	S	S	S	S	S	S
5149-09	MSF	Swaziland	Beijing	ERR2200011 ER	R	S	S	n/a	R	S	S	S
173-10	MSF	Swaziland	LAM	ERR2199834	S	S	S	n/a	S	S	S	S
4288-09	MSF	Swaziland	Beijing	ERR553058	R	R	R	S	R	R	R	R
4248-09	MSF	Swaziland	S-type	ERR552193	R	R	R	R	R	R	R	R
597-10	MSF	Swaziland	S-type	ERR2200058 ER	R	R	R	R	R	R	R	R
601-10	MSF	Swaziland	X-type	ERR2200060 ER	R	R	R	R	R	R	R	U
5148-09	MSF	Swaziland	Beijing	ERR2200010	S	S	S	n/a	S	S	S	S
4716-09	MSF	Swaziland	LAM	ERR2199976	S	S	S	n/a	S	S	U	S
5569-09	MSF	Swaziland	Beijing	ERR553156 ERR	S	S	S	n/a	S	S	S	S
5797-09	MSF	Swaziland	LAM	ERR551391	R	S	S	S	R	S	S	S
4699-09	MSF	Swaziland	lineage4	ERR2199973	S	S	S	n/a	S	S	S	S
4290-09	MSF	Swaziland	EAI	ERR2199940	S	S	S	n/a	S	S	S	S
4259-09	MSF	Swaziland	LAM	ERR2199927	S	S	S	n/a	S	S	S	S
4257-09	MSF	Swaziland	Beijing	ERR2199925	S	S	S	n/a	R	S	S	S
7179-09	MSF	Swaziland	EAI	ERR552495	R	R	R	R	R	R	R	R
4249-09	MSF	Swaziland	Haarlem	ERR2199920	S	S	S	n/a	S	S	S	S
4721-09	MSF	Swaziland	S-type	ERR553230	R	R	R	R	R	R	R	R
5275-09	MSF	Swaziland	S-type	ERR2200022 ER	R	R	R	R	R	R	R	R
5273-09	MSF	Swaziland	Beijing	ERR2200021	S	S	S	n/a	S	S	S	S
10114-09	MSF	Swaziland	lineage4	ERR551166	R	R	R	R	R	R	R	R
119-10	MSF	Swaziland	X-type	ERR2199805 ER	R	R	R	R	R	R	R	U
4286-09	MSF	Swaziland	X-type	ERR2199939	S	S	S	n/a	S	S	U	S
4271-09	MSF	Swaziland	LAM	ERR2199934	S	S	S	n/a	R	S	S	S
6945-09	MSF	Swaziland	EAI	ERR2200095 ER	R	R	R	R	R	R	R	R
4709-09	MSF	Swaziland	S-type	ERR550741	R	R	R	R	R	R	R	R
111-10	MSF	Swaziland	lineage4	ERR551953	R	R	R	R	R	R	R	R
4297-09	MSF	Swaziland	EAI	ERR2199945	S	S	S	n/a	S	S	S	S
6652-09	MSF	Swaziland	X-type	ERR552073	R	R	R	R	R	R	R	U
1210-10	MSF	Swaziland	S-type	ERR551488	R	R	R	R	R	R	R	R
177-10	MSF	Swaziland	LAM	ERR2199842	S	S	S	n/a	R	S	S	S
7703-09	MSF	Swaziland	S-type	ERR552149 ERR	R	R	R	R	R	R	R	R
10703-09	MSF	Swaziland	lineage4	ERR551289	R	R	R	S	R	R	R	R
5104-09	MSF	Swaziland	LAM	ERR550794	R	S	S	S	R	S	S	S
4294-09	MSF	Swaziland	LAM	ERR2199943	S	S	S	n/a	S	S	U	S
9081-09	MSF	Swaziland	S-type	ERR552986 ERR	R	R	R	R	R	R	R	R
7699-09	MSF	Swaziland	Delhi	ERR2200105	S	S	S	n/a	S	S	S	S
5743-09	MSF	Swaziland	Beijing	ERR2200044	S	S	S	n/a	S	S	S	S
4335-09	MSF	Swaziland	X-type	ERR552108 ERR	S	S	S	n/a	S	S	S	S
4250-09	MSF	Swaziland	X-type	ERR2199921	S	S	S	n/a	S	S	S	S
5251-09	MSF	Swaziland	LAM	ERR553366	S	R	S	S	U	R	S	S
6637-09	MSF	Swaziland	EAI	ERR550869	R	R	R	R	R	R	R	R
4687-09	MSF	Swaziland	LAM	ERR2199968 ER	S	S	S	S	R	S	S	S
4291-09	MSF	Swaziland	LAM	ERR2199941	S	S	S	n/a	U	S	S	S
5107-09	MSF	Swaziland	S-type	ERR2200000	S	S	S	n/a	S	S	S	S
7708-09	MSF	Swaziland	lineage4	ERR552780	R	R	R	n/a	R	R	R	R
7712-09	MSF	Swaziland	lineage4	ERR551689	R	S	S	S	R	S	S	S
5551-09	MSF	Swaziland	lineage4	ERR2200035	S	S	S	n/a	S	S	S	S
9564-09	MSF	Swaziland	lineage4	ERR553001 ERR	R	R	R	R	R	R	F	R
175-10	MSF	Swaziland	EAI	ERR551801	R	R	R	R	R	R	R	R
4276-09	MSF	Swaziland	Beijing	ERR553324	S	S	S	n/a	S	S	U	S
5748-09	MSF	Swaziland	EAI	ERR2200046 ER	R	R	R	R	R	R	R	R
5537-09	MSF	Swaziland	lineage4	ERR2200030 ER	R	S	S	S	R	S	S	S
6247-09	MSF	Swaziland	Beijing	ERR2200075 ER	R	R	R	S	R	R	R	S
10108-09	MSF	Swaziland	S-type	ERR2199747 ER	R	R	R	R	R	R	R	R
5076-09	MSF	Swaziland	S-type	ERR2199991	S	S	S	n/a	S	S	U	S
11239-09	MSF	Swaziland	EAI	ERR553321	R	R	R	R	R	R	R	R
5282-09	MSF	Swaziland	S-type	ERR2200024	S	S	S	n/a	S	S	S	S
4333-09	MSF	Swaziland	EAI	ERR2199950	S	S	S	n/a	S	S	S	S
6944-09	MSF	Swaziland	S-type	ERR2200094 ER	R	R	R	R	R	R	R	R
4295-09	MSF	Swaziland	lineage4	ERR2199944	S	S	S	n/a	S	S	S	S
4287-09	MSF	Swaziland	Beijing	ERR619080	S	S	S	n/a	R	S	S	S
11023-09	MSF	Swaziland	S-type	ERR551014	R	R	R	R	R	R	R	R
5746-09	MSF	Swaziland	lineage4	ERR2200045	S	S	S	n/a	S	S	S	S
10504-09	MSF	Swaziland	lineage4	ERR552273	R	R	R	R	R	R	R	R
4301-09	MSF	Swaziland	lineage4	ERR2199947	S	S	S	n/a	S	S	S	S
7181-09	MSF	Swaziland	LAM	ERR553358	R	R	R	R	R	R	R	R
5115-09	MSF	Swaziland	S-type	ERR2200004	S	S	S	n/a	S	S	S	S
5759-09	MSF	Swaziland	Beijing	ERR2200051	S	S	S	n/a	S	S	S	S
1215-10	MSF	Swaziland	lineage4	ERR2199813 ER	S	S	S	n/a	S	S	S	S
4337-09	MSF	Swaziland	S-type	ERR2199951	S	S	S	n/a	S	S	S	S
5239-09	MSF	Swaziland	EAI	ERR2200013	S	S	S	n/a	S	S	U	S
4261-09	MSF	Swaziland	lineage4	ERR2199928	S	S	S	n/a	S	S	S	S
5771-09	MSF	Swaziland	Beijing	ERR2200052	S	S	S	n/a	S	S	S	S
4262-09	MSF	Swaziland	LAM	ERR2199929	S	S	S	n/a	S	S	S	S
10507-09	MSF	Swaziland	Beijing	ERR553313	R	R	R	R	R	R	R	U
5554-09	MSF	Swaziland	Haarlem	ERR2200037	S	S	S	n/a	S	S	S	S
7700-09	MSF	Swaziland	S-type	ERR552745	R	R	R	R	R	R	R	R
4700-09	MSF	Swaziland	S-type	ERR551609	R	n/a	R	R	R	R	R	R
5146-09	MSF	Swaziland	lineage4	ERR552701	R	R	S	R	R	R	R	R
5109-09	MSF	Swaziland	S-type	ERR552540	R	R	S	R	R	R	R	R
4345-09	MSF	Swaziland	EAI	ERR2199955	S	S	S	n/a	R	S	S	S
5075-09	MSF	Swaziland	lineage4	ERR2199990	S	S	S	n/a	S	S	S	S
5287-09	MSF	Swaziland	Beijing	ERR2200025	S	S	S	n/a	S	R	S	S
10512-09	MSF	Swaziland	EAI	ERR551319	R	R	R	R	R	R	R	R
6255-09	MSF	Swaziland	X-type	ERR551937 ERR	R	R	R	R	R	R	R	U
7192-09	MSF	Swaziland	Haarlem	ERR551578	R	R	S	S	R	R	U	S
4330-09	MSF	Swaziland	lineage4	ERR552456	R	R	S	R	R	R	R	R
10115-09	MSF	Swaziland	S-type	ERR2199748 ER	R	R	R	R	R	R	R	R
4252-09	MSF	Swaziland	X-type	ERR550619	R	R	R	R	R	R	R	R
5073-09	MSF	Swaziland	X-type	ERR552751	R	R	R	n/a	R	R	R	U
113-10	MSF	Swaziland	Haarlem	ERR551537	R	R	S	R	F	F	S	F
11234-09	MSF	Swaziland	EAI	ERR2199793 ER	R	R	R	R	R	R	R	R
5269-09	MSF	Swaziland	Haarlem	ERR2200020	S	S	S	n/a	S	S	S	S
8591-09	MSF	Swaziland	X-type	ERR552181 ERR	R	R	R	n/a	R	R	R	U
11236-09	MSF	Swaziland	EAI	ERR550754	R	R	R	R	R	R	R	R
8580-09	MSF	Swaziland	LAM	ERR2200117	S	S	S	n/a	S	S	S	S
5117-09	MSF	Swaziland	lineage4	ERR2200005	S	S	S	n/a	S	S	S	S

6653-09	MSF	Swaziland	LAM	ERR2200088	S	S	S	n/a	S	S	S	S
5069-09	MSF	Swaziland	X-type	ERR2199988 ER	R	R	R	R	R	F	F	F
5558-09	MSF	Swaziland	EAI	ERR551899	R	R	R	R	R	R	R	R
6087-09	MSF	Swaziland	X-type	ERR2200068 ER	S	S	S	n/a	S	S	S	S
8784-09	MSF	Swaziland	lineage4	ERR552809	R	R	R	R	R	R	R	R
7172-09	MSF	Swaziland	lineage4	ERR2200103	S	S	S	n/a	S	S	S	S
4272-09	MSF	Swaziland	X-type	ERR2199935	S	S	S	n/a	S	S	S	S
10294-09	MSF	Swaziland	S-type	ERR2199757 ER	R	R	R	R	R	R	R	R
6256-09	MSF	Swaziland	X-type	ERR2200078	S	S	S	n/a	S	S	S	S
4712-09	MSF	Swaziland	X-type	ERR552518	R	R	R	R	R	R	R	R
6268-09	MSF	Swaziland	Haarlem	ERR2200080	S	S	S	n/a	S	S	S	S
6267-09	MSF	Swaziland	X-type	ERR552993 ERR	R	R	R	R	R	R	R	U
5539-09	MSF	Swaziland	lineage4	ERR2200032	S	S	S	n/a	S	S	S	S
5544-09	MSF	Swaziland	X-type	ERR2200033	S	S	S	n/a	S	S	S	S
6269-09	MSF	Swaziland	Haarlem	ERR553216	R	R	R	R	R	R	R	R
1213-10	MSF	Swaziland	Haarlem	ERR2199811 ER	S	S	S	S	S	S	S	S
7171-09	MSF	Swaziland	X-type	ERR553264	R	R	R	n/a	R	R	R	U
5118-09	MSF	Swaziland	S-type	ERR550867 ERR	S	S	S	n/a	S	S	S	S
6265-09	MSF	Swaziland	Beijing	ERR552743	S	S	S	n/a	S	S	S	S
4270-09	MSF	Swaziland	Beijing	ERR2199933	S	S	S	n/a	S	S	S	S
5080-09	MSF	Swaziland	S-type	ERR552599	R	R	R	R	R	R	R	R
5772-09	MSF	Swaziland	X-type	ERR550734	R	R	R	R	R	R	R	U
8581-09	MSF	Swaziland	lineage4	ERR552463	R	R	S	R	R	R	R	R
4289-09	MSF	Swaziland	LAM	ERR552352	R	S	S	R	R	S	S	R
4725-09	MSF	Swaziland	S-type	ERR552637	R	n/a	R	R	R	R	R	R
8781-09	MSF	Swaziland	S-type	ERR552070	R	R	R	R	R	R	R	R
10116-09	MSF	Swaziland	S-type	ERR2199749	S	S	S	n/a	S	S	S	S
4275-09	MSF	Swaziland	LAM	ERR2199937	S	S	S	n/a	S	S	S	S
4328-09	MSF	Swaziland	EAI	ERR2199949	S	S	S	n/a	S	S	S	S
4703-09	MSF	Swaziland	Beijing	ERR551071	S	S	S	n/a	S	S	S	S
6249-09	MSF	Swaziland	Haarlem	ERR2200076	S	S	S	n/a	S	S	S	S
6263-09	MSF	Swaziland	Haarlem	ERR2200079	S	S	S	n/a	S	S	S	S
8304-09	MSF	Swaziland	Beijing	ERR553116	S	R	S	S	S	R	S	S
10442-09	MSF	Swaziland	lineage4	ERR2199768 ER	R	R	S	R	R	R	S	R
5753-09	MSF	Swaziland	S-type	ERR552484	R	R	R	R	R	R	R	R
8576-09	MSF	Swaziland	S-type	ERR553282	R	R	R	R	R	R	R	R
5276-09	MSF	Swaziland	LAM	ERR2200023	S	S	S	n/a	S	S	S	S
4735-09	MSF	Swaziland	LAM	ERR2199982	S	S	S	n/a	S	S	S	S
4300-09	MSF	Swaziland	EAI	ERR2199946	S	S	S	n/a	S	S	S	S
4343-09	MSF	Swaziland	LAM	ERR2199952	S	S	S	n/a	S	S	S	S
WMB430/43-12700	Thailand via University of Singapore	Thailand	Beijing	SAMN07236448	S	S	S	n/a	S	S	S	S
WMB476/46-10698	Thailand via University of Singapore	Thailand	Beijing	SAMN07236487	S	S	S	n/a	S	S	S	S
WMB261/DS-20512	Thailand via University of Singapore	Thailand	Beijing	SAMN07236285	R	R	R	n/a	R	R	R	R
WMB422/42-19187	Thailand via University of Singapore	Thailand	Beijing	SAMN07236440	R	S	S	n/a	R	S	S	S
WMB322/DS-15901	Thailand via University of Singapore	Thailand	Beijing	SAMN07360950	R	R	S	n/a	R	F	S	S
WMB238/DS-08775	Thailand via University of Singapore	Thailand	Beijing	SAMN07236262	R	R	R	n/a	R	R	R	U
WBB284/DS-31231	Thailand via University of Singapore	Thailand	Beijing	SAMN07236538	R	R	R	n/a	R	R	R	R
WMB321/DS-15862	Thailand via University of Singapore	Thailand	Beijing	SAMN07236343	R	R	S	n/a	F	F	S	S
WMB464/CSF-3542	Thailand via University of Singapore	Thailand	Beijing	SAMN07236477	S	S	S	n/a	S	S	U	S
WMB388/DS-30902	Thailand via University of Singapore	Thailand	Beijing	SAMN07236406	R	R	S	n/a	R	R	S	S
WBB280/DS-29147	Thailand via University of Singapore	Thailand	Beijing	SAMN07236534	R	R	R	n/a	R	R	R	R
WMB300/DS-13819	Thailand via University of Singapore	Thailand	Beijing	SAMN07236322	R	R	R	n/a	R	R	R	R
WMB296/DS-33048	Thailand via University of Singapore	Thailand	Beijing	SAMN07236318	R	R	R	n/a	R	R	R	R
WBB264/DS-17092	Thailand via University of Singapore	Thailand	Beijing	SAMN07236520	R	R	S	n/a	U	R	R	R
WMB382/DS-25482	Thailand via University of Singapore	Thailand	Beijing	SAMN07236400	R	R	n/a	n/a	R	R	S	U
WMB366/DS-23415	Thailand via University of Singapore	Thailand	Beijing	SAMN07236385	R	R	R	n/a	R	R	R	R
WMB454/CSF-3382	Thailand via University of Singapore	Thailand	Beijing	SAMN07236469	S	S	S	n/a	S	S	S	S
WMB287/DS-30777	Thailand via University of Singapore	Thailand	Beijing	SAMN07236309	R	R	S	n/a	R	R	R	R
WMB447/CSF-2894	Thailand via University of Singapore	Thailand	Beijing	SAMN07236464	S	S	S	n/a	S	S	S	S
WMB370/DS-24060	Thailand via University of Singapore	Thailand	Beijing	SAMN07236389	R	R	R	n/a	R	R	R	R
WMB340/DS-19383	Thailand via University of Singapore	Thailand	Beijing	SAMN07236360	R	R	S	n/a	R	R	S	S
WMB292/DS-32101	Thailand via University of Singapore	Thailand	Beijing	SAMN07236314	R	R	R	n/a	R	R	R	R
WMB423/43-01910	Thailand via University of Singapore	Thailand	EAI	SAMN07236441	S	S	S	n/a	S	U	S	S
WMB417/DS-20257	Thailand via University of Singapore	Thailand	Beijing	SAMN07236435	S	S	S	n/a	S	S	S	S
WBB260/DS-16780	Thailand via University of Singapore	Thailand	Beijing	SAMN07236516	R	R	S	n/a	R	R	R	U
WMB282/DS-29788	Thailand via University of Singapore	Thailand	Beijing	SAMN07236304	S	R	S	n/a	U	F	S	U
WMB229/DS-16496	Thailand via University of Singapore	Thailand	Beijing	SAMN07236255	R	R	R	n/a	R	R	R	R
WMB409/DS-15367	Thailand via University of Singapore	Thailand	Beijing	SAMN07236427	S	S	S	n/a	S	S	S	S
WMB315/DS-14459	Thailand via University of Singapore	Thailand		SAMN07236337	R	R	R	n/a	F	F	F	F
WMB295/DS-32505	Thailand via University of Singapore	Thailand	Beijing	SAMN07236317	R	R	S	n/a	R	F	U	R
WMB361/DS-21625	Thailand via University of Singapore	Thailand	Beijing	SAMN07236380	R	R	n/a	n/a	R	R	S	S
WMB222/DS-06765	Thailand via University of Singapore	Thailand	Beijing	SAMN07236248	R	R	R	n/a	R	R	R	R
WMB446/CSF-2498	Thailand via University of Singapore	Thailand	Beijing	SAMN07236463	S	S	S	n/a	S	S	S	S
WMB482/48-10231	Thailand via University of Singapore	Thailand	Beijing	SAMN07236491	R	S	S	n/a	R	S	S	S
WMB342/DS-20136	Thailand via University of Singapore	Thailand	EAI	SAMN07236362	R	R	R	n/a	R	R	S	S
WMB247/DS-11861	Thailand via University of Singapore	Thailand	Beijing	SAMN07236271	R	R	R	n/a	R	R	R	R
WBB261/DS-16825	Thailand via University of Singapore	Thailand	Beijing	SAMN07236517	R	R	R	n/a	R	S	U	R
WMB367/DS-23522	Thailand via University of Singapore	Thailand	Beijing	SAMN07236386	R	R	n/a	n/a	R	R	R	S
WMB375/DS-24447	Thailand via University of Singapore	Thailand	Beijing	SAMN07236393	R	R	R	n/a	R	R	R	F
WMB224/DS-13006	Thailand via University of Singapore	Thailand	EAI	SAMN07236250	R	R	S	n/a	R	R	U	S
WMB400/DS-12158	Thailand via University of Singapore	Thailand	Beijing	SAMN07236418	R	R	S	n/a	R	R	R	R
WMB470/CSF-3681	Thailand via University of Singapore	Thailand	Beijing	SAMN07236482	S	S	S	n/a	S	S	S	S
WMB407/DS-15177	Thailand via University of Singapore	Thailand	Beijing	SAMN07236425	S	S	S	n/a	S	S	S	S
WMB479/CSF-3922	Thailand via University of Singapore	Thailand	EAI	SAMN07236489	S	S	S	n/a	U	S	S	S
WMB269/DS-21261	Thailand via University of Singapore	Thailand	Beijing	SAMN07236293	R	R	n/a	n/a	R	R	U	R
WMB318/DS-15492	Thailand via University of Singapore	Thailand	Beijing	SAMN07236340	R	R	S	n/a	R	R	R	S
WMB297/DS-33051	Thailand via University of Singapore	Thailand	Beijing	SAMN07236319	R	R	R	n/a	R	R	U	R
WMB439/46-18357	Thailand via University of Singapore	Thailand	EAI	SAMN07236457	S	S	S	n/a	S	S	S	S
WMB289/DS-31524	Thailand via University of Singapore	Thailand	Beijing	SAMN07236311	R	R	R	n/a	R	R	R	R
WMB279/DS-28473	Thailand via University of Singapore	Thailand	Beijing	SAMN07360949	R	R	R	n/a	R	R	R	S
WMB272/DS-24901	Thailand via University of Singapore	Thailand	Beijing	SAMN07236296	R	R	R	n/a	R	R	U	R
WMB254/DS-18227	Thailand via University of Singapore	Thailand	Beijing	SAMN07236278	R	R	R	n/a	R	R	R	R
WMB316/DS-14460	Thailand via University of Singapore	Thailand	Beijing	SAMN07236338	R	R	S	n/a	R	R	R	R
WMB472/CSF-3718	Thailand via University of Singapore	Thailand	EAI	SAMN07236484	S	S	S	n/a	S	S	S	S
WMB244/DS-15773	Thailand via University of Singapore	Thailand	Beijing	SAMN07236268	R	R	S	n/a	R	R	R	R
WMB405/DS-14607	Thailand via University of Singapore	Thailand	Beijing	SAMN07236423	S	S	S	n/a	S	S	S	S
WMB267/DS-21129	Thailand via University of Singapore	Thailand	Beijing	SAMN07236291	R	R	R	n/a	R	R	R	R
WMB415/DS-19997	Thailand via University of Singapore	Thailand	EAI	SAMN07236433	S	S	S	n/a	S	S	U	S
WMB346/DS-20225	Thailand via University of Singapore	Thailand	Beijing	SAMN07236365	R	R	R	n/a	R	R	R	R
WMB291/DS-31852	Thailand via University of Singapore	Thailand	Beijing	SAMN07236313	R	R	R	n/a	R	R	R	S
WMB355/DS-20915	Thailand via University of Singapore	Thailand	Beijing	SAMN07236374	R	R	n/a	n/a	R	R	R	U
WMB274/DS-25520	Thailand via University of Singapore	Thailand	Beijing	SAMN07236297	R	R	S	n/a	R	R	R	S
WMB386/DS-25946	Thailand via University of Singapore	Thailand	Beijing	SAMN07236404	R	R	n/a	n/a	R	R	F	S
WMB259/DS-19810	Thailand via University of Singapore	Thailand	EAI	SAMN07236283	R	R	R	n/a	R	F	R	F
WMB357/DS-21031	Thailand via University of Singapore	Thailand	Beijing	SAMN07236376	R	R	R	n/a	R	R	R	S
WBB259/DS-16220	Thailand via University of Singapore	Thailand	Beijing	SAMN07236515	R	R	R	n/a	R	R	R	U
WMB418/DS-20316	Thailand via University of Singapore	Thailand	Beijing	SAMN07236436	S	S	S	n/a	S	S	S	S
WMB276/DS-26452	Thailand via University of Singapore	Thailand	Beijing	SAMN07236299	R	R	n/a	n/a	R	R	R	F
WMB260/DS-20000	Thailand via University of Singapore	Thailand	Ural	SAMN07236284	R	R	R	n/a	R	R	R	R
WMB377/DS-24593	Thailand via University of Singapore	Thailand	EAI	SAMN07236395	R	R	S	n/a	R	R	S	S
WMB275/DS-26102	Thailand via University of Singapore	Thailand	Beijing	SAMN07236298	S	R	n/a	n/a	S	S	S	S
WMB414/DS-19861	Thailand via University of Singapore	Thailand	Beijing	SAMN07236432	S	S	S	n/a	S	S	S	S
WMB332/DS-19168	Thailand via University of Singapore	Thailand	EAI	SAMN07236352	R	R	R	n/a	R	R	S	S
WMB480/48-10563	Thailand via University of Singapore	Thailand	Beijing	SAMN07236490	R	S	S	n/a	R	S	S	S
WMB389/DS-30951	Thailand via University of Singapore	Thailand	Beijing	SAMN07236407	R	R	S	n/a	F	R	S	S
WMB271/DS-21363	Thailand via University of Singapore	Thailand	Beijing	SAMN07236295	R	R	n/a	n/a	R	R	R	R

WMB273/DS-25474	Thailand via University of Singapore	Thailand	Beijing	SAMN07360948	R	R	n/a	n/a	R	R	R	U
WBB263/DS-17016	Thailand via University of Singapore	Thailand	Beijing	SAMN07236519	R	R	R	n/a	R	R	R	F
WMB314/DS-14418	Thailand via University of Singapore	Thailand	Beijing	SAMN07236336	R	R	S	n/a	R	R	R	R
WMB319/DS-15504	Thailand via University of Singapore	Thailand	Beijing	SAMN07236341	R	R	R	n/a	R	R	U	S
WMB453/47-5556	Thailand via University of Singapore	Thailand	Beijing	SAMN07236468	S	S	S	n/a	S	S	S	S
WMB406/DS-14963	Thailand via University of Singapore	Thailand	EAI	SAMN07236424	S	S	S	n/a	S	S	S	S
WMB268/DS-21256	Thailand via University of Singapore	Thailand	Beijing	SAMN07236292	R	R	n/a	n/a	R	R	R	U
WMB387/DS-26071	Thailand via University of Singapore	Thailand	Beijing	SAMN07236405	R	R	n/a	n/a	R	R	R	R
WMB460/CSF-3520	Thailand via University of Singapore	Thailand	Beijing	SAMN07236473	S	S	S	n/a	S	S	S	S
WMB228/DS-13085	Thailand via University of Singapore	Thailand	EAI	SAMN07236254	R	R	S	n/a	R	R	U	S
WMB302/DS-13922	Thailand via University of Singapore	Thailand	Beijing	SAMN07236324	R	R	R	n/a	R	R	U	R
WBB257/DS-15966	Thailand via University of Singapore	Thailand	Beijing	SAMN07236513	R	R	R	n/a	R	R	R	R
WMB383/DS-25553	Thailand via University of Singapore	Thailand	Beijing	SAMN07236401	R	R	n/a	n/a	R	R	R	R
WMB416/DS-20072	Thailand via University of Singapore	Thailand	EAI	SAMN07236434	S	S	S	n/a	S	S	S	S
WMB288/DS-30870	Thailand via University of Singapore	Thailand	EAI	SAMN07236310	S	S	S	n/a	S	S	S	S
WMB429/43-12683	Thailand via University of Singapore	Thailand	lineage4	SAMN07236447	S	S	S	n/a	S	S	S	S
WMB253/DS-18044	Thailand via University of Singapore	Thailand	Ural	SAMN07236277	R	R	R	n/a	R	R	R	R
WMB283/DS-30254	Thailand via University of Singapore	Thailand	Beijing	SAMN07236305	R	R	S	n/a	R	R	R	R
WMB394/DS-31799	Thailand via University of Singapore	Thailand	Beijing	SAMN07236412	R	R	R	n/a	R	R	U	S
WMB232/DS-13458	Thailand via University of Singapore	Thailand	S-type	SAMN07236257	R	R	R	n/a	R	R	F	F
WMB363/DS-21768	Thailand via University of Singapore	Thailand	Beijing	SAMN07236382	R	R	n/a	n/a	R	R	R	F
WMB484/CSF-4014	Thailand via University of Singapore	Thailand	EAI	SAMN07236493	R	S	S	n/a	R	U	U	R
WMB469/42-20464	Thailand via University of Singapore	Thailand	Beijing	SAMN07236481	S	S	S	n/a	S	S	S	S
WMB330/DS-18964	Thailand via University of Singapore	Thailand	Beijing	SAMN07236350	R	R	R	n/a	R	R	R	S
WMB368/DS-23682	Thailand via University of Singapore	Thailand	Beijing	SAMN07236387	R	R	R	n/a	R	R	F	S
WMB341/DS-20092	Thailand via University of Singapore	Thailand	Beijing	SAMN07236361	R	R	R	n/a	R	F	F	F
WMB245/DS-17305	Thailand via University of Singapore	Thailand	EAI	SAMN07236269	R	R	R	n/a	R	R	R	F
WMB478/48-4979	Thailand via University of Singapore	Thailand	Beijing	SAMN07236488	S	S	S	n/a	S	S	S	S
WMB435/43-19186	Thailand via University of Singapore	Thailand	EAI	SAMN07236453	S	S	S	n/a	S	S	U	S
WMB251/DS-12236	Thailand via University of Singapore	Thailand	Beijing	SAMN07236275	R	R	R	n/a	R	R	R	R
WMB401/DS-16616	Thailand via University of Singapore	Thailand	Beijing	SAMN07236419	R	R	R	n/a	R	R	S	S
WBB283/DS-30971	Thailand via University of Singapore	Thailand	Beijing	SAMN07236537	R	R	R	n/a	R	R	R	U
WMB249/DS-17658	Thailand via University of Singapore	Thailand	Beijing	SAMN07236273	R	R	R	n/a	R	R	R	R
WBB278/DS-27535	Thailand via University of Singapore	Thailand	Beijing	SAMN07236532	R	R	n/a	n/a	R	R	R	R
WMB248/DS-15837	Thailand via University of Singapore	Thailand	EAI	SAMN07236272	R	R	R	n/a	R	R	R	R
WMB463/CSF-3541	Thailand via University of Singapore	Thailand	EAI	SAMN07236476	S	S	S	n/a	S	S	S	S
WMB413/DS-19477	Thailand via University of Singapore	Thailand	lineage4	SAMN07236431	S	S	S	n/a	S	U	U	S
WMB290/DS-31545	Thailand via University of Singapore	Thailand	Beijing	SAMN07236312	R	R	R	n/a	R	R	R	R
WMB496/CSF-4650	Thailand via University of Singapore	Thailand	EAI	SAMN07236505	R	S	S	n/a	S	S	S	S
WMB264/DS-20910	Thailand via University of Singapore	Thailand	Beijing	SAMN07236288	R	R	n/a	n/a	R	R	R	U
WMB250/DS-10275	Thailand via University of Singapore	Thailand	Beijing	SAMN07236274	R	R	R	n/a	R	R	R	R
WMB280/DS-29208	Thailand via University of Singapore	Thailand	Beijing	SAMN07236302	R	R	R	n/a	R	R	R	R
WMB421/DS-20202	Thailand via University of Singapore	Thailand	S-type	SAMN07236439	S	S	S	n/a	S	S	S	S
WMB326/DS-16282	Thailand via University of Singapore	Thailand	Beijing	SAMN07236347	R	R	S	n/a	R	R	S	S
WMB468/42-14745	Thailand via University of Singapore	Thailand	S-type	SAMN07236480	S	S	S	n/a	S	S	S	S
WBB281/DS-29366	Thailand via University of Singapore	Thailand	Beijing	SAMN07236535	R	R	R	n/a	R	R	R	U
WBB285/DS-32449	Thailand via University of Singapore	Thailand	Beijing	SAMN07236539	R	R	R	n/a	R	R	R	U
WBB265/DS-17653	Thailand via University of Singapore	Thailand	Beijing	SAMN07236521	R	R	R	n/a	R	R	U	S
WMB328/DS-16314	Thailand via University of Singapore	Thailand	Beijing	SAMN07236349	R	R	S	n/a	S	S	S	S
WMB408/DS-15203	Thailand via University of Singapore	Thailand	Beijing	SAMN07236426	S	S	S	n/a	S	S	S	S
WMB376/DS-24457	Thailand via University of Singapore	Thailand	EAI	SAMN07236394	R	R	S	n/a	S	S	S	S
WMB381/DS-25234	Thailand via University of Singapore	Thailand	Beijing	SAMN07236399	R	R	R	n/a	R	R	R	R
WMB462/CSF-3538	Thailand via University of Singapore	Thailand	EAI	SAMN07236475	R	R	R	n/a	R	R	R	R
WBB270/DS-19048	Thailand via University of Singapore	Thailand	Beijing	SAMN07236525	R	R	R	n/a	R	R	R	S
WBB271/DS-19109	Thailand via University of Singapore	Thailand	Beijing	SAMN07236526	R	R	R	n/a	R	R	R	U
WMB362/DS-21639	Thailand via University of Singapore	Thailand	Beijing	SAMN07236381	R	R	n/a	n/a	R	R	R	S
WMB286/DS-30535	Thailand via University of Singapore	Thailand	Beijing	SAMN07236308	R	R	S	n/a	R	R	R	R
WMB378/DS-24900	Thailand via University of Singapore	Thailand	Beijing	SAMN07236396	R	R	R	n/a	R	R	R	S
WMB393/DS-31665	Thailand via University of Singapore	Thailand	Beijing	SAMN07236411	R	R	R	n/a	R	R	R	R
WBB276/DS-26339	Thailand via University of Singapore	Thailand	EAI	SAMN07236530	R	R	n/a	n/a	S	S	U	S
WMB285/DS-30442	Thailand via University of Singapore	Thailand	Beijing	SAMN07236307	S	R	S	n/a	S	R	S	S
WMB351/DS-20550	Thailand via University of Singapore	Thailand	Beijing	SAMN07236370	R	R	R	n/a	R	R	R	S
WMB379/DS-24957	Thailand via University of Singapore	Thailand	Tur	SAMN07236397	R	R	R	n/a	R	R	R	S
WMB335/DS-19259	Thailand via University of Singapore	Thailand	Beijing	SAMN07236355	R	R	R	n/a	R	R	R	S
WMB448/CSF-3053	Thailand via University of Singapore	Thailand	EAI	SAMN07236465	S	S	S	n/a	S	S	S	S
WMB452/CSF-3367	Thailand via University of Singapore	Thailand	Beijing	SAMN07236467	S	S	S	n/a	S	S	S	S
WMB427/43-11467	Thailand via University of Singapore	Thailand	Beijing	SAMN07236445	S	S	S	n/a	S	S	S	S
WMB457/47-10904	Thailand via University of Singapore	Thailand	Beijing	SAMN07236471	S	S	S	n/a	S	S	S	S
WMB237/DS-16630	Thailand via University of Singapore	Thailand	Beijing	SAMN07236261	R	R	R	n/a	R	R	R	U
WMB334/DS-19200	Thailand via University of Singapore	Thailand	Beijing	SAMN07236354	R	R	R	n/a	R	R	U	R
WMB392/DS-31586	Thailand via University of Singapore	Thailand	Tur	SAMN07236410	R	R	S	n/a	R	R	U	S
WMB309/DS-16144	Thailand via University of Singapore	Thailand	Beijing	SAMN07236331	R	R	R	n/a	R	R	R	R
WMB440/46-19260	Thailand via University of Singapore	Thailand	lineage4	SAMN07236458	S	S	S	n/a	S	S	S	S
WMB475/46-5069	Thailand via University of Singapore	Thailand	EAI	SAMN07236486	S	S	S	n/a	S	S	S	S
WMB236/DS-13671	Thailand via University of Singapore	Thailand	Beijing	SAMN07236260	R	R	R	n/a	U	R	R	U
WMB331/DS-19155	Thailand via University of Singapore	Thailand	Beijing	SAMN07236351	R	R	R	n/a	R	R	S	S
WMB380/DS-25029	Thailand via University of Singapore	Thailand	Beijing	SAMN07236398	R	R	R	n/a	R	R	R	S
WMB384/DS-25707	Thailand via University of Singapore	Thailand	Beijing	SAMN07236402	R	R	n/a	n/a	R	R	S	S
WMB403/DS-18613	Thailand via University of Singapore	Thailand	Beijing	SAMN07236421	R	R	S	n/a	R	R	R	R
WMB266/DS-21034	Thailand via University of Singapore	Thailand	Beijing	SAMN07236290	R	R	R	n/a	R	R	R	R
WMB235/DS-11221	Thailand via University of Singapore	Thailand	Beijing	SAMN07236259	R	R	R	n/a	R	R	R	S
WBB262/DS-17012	Thailand via University of Singapore	Thailand	Beijing	SAMN07236518	R	R	R	n/a	F	F	F	U
WBB272/DS-20120	Thailand via University of Singapore	Thailand	Beijing	SAMN07236527	S	R	n/a	n/a	U	R	R	S
WMB293/DS-32317	Thailand via University of Singapore	Thailand	EAI	SAMN07236315	R	R	S	n/a	R	R	U	S
WMB350/DS-20503	Thailand via University of Singapore	Thailand	EAI	SAMN07236369	R	R	R	n/a	S	U	S	S
WMB347/DS-20259	Thailand via University of Singapore	Thailand	Beijing	SAMN07236366	R	R	R	n/a	R	R	S	S
WMB364/DS-22290	Thailand via University of Singapore	Thailand	Beijing	SAMN07236383	R	R	n/a	n/a	R	R	R	R
WMB471/CSF-3711	Thailand via University of Singapore	Thailand	EAI	SAMN07236483	S	S	S	n/a	S	S	S	S
WMB354/DS-20815	Thailand via University of Singapore	Thailand	Beijing	SAMN07236373	R	R	n/a	n/a	R	R	S	S
WMB306/DS-16226	Thailand via University of Singapore	Thailand	Beijing	SAMN07236328	R	R	R	n/a	R	R	R	S
WMB420/DS-20168	Thailand via University of Singapore	Thailand	EAI	SAMN07236438	S	S	S	n/a	S	S	S	S
WBB256/DS-6882	Thailand via University of Singapore	Thailand	Beijing	SAMN07236512	R	R	R	n/a	R	R	R	R
WMB345/DS-20224	Thailand via University of Singapore	Thailand	Beijing	SAMN07360951	R	R	R	n/a	R	R	R	R
WMB225/DS-16260	Thailand via University of Singapore	Thailand	EAI	SAMN07236251	R	R	R	n/a	R	R	R	R
WMB339/DS-19355	Thailand via University of Singapore	Thailand	Beijing	SAMN07236359	R	R	S	n/a	R	R	S	S
WMB371/DS-24258	Thailand via University of Singapore	Thailand	Beijing	SAMN07236390	R	R	R	n/a	R	R	R	R
WBB266/DS-17688	Thailand via University of Singapore	Thailand	Beijing	SAMN07236522	R	R	R	n/a	R	R	R	S
WMB223/DS-10284	Thailand via University of Singapore	Thailand	Beijing	SAMN07236249	R	R	R	n/a	R	R	U	S
WBB255/DS-6265	Thailand via University of Singapore	Thailand	LAM	SAMN07236511	R	R	R	n/a	R	R	R	S
WMB397/DS-32630	Thailand via University of Singapore	Thailand	Beijing	SAMN07236415	R	R	R	n/a	R	R	R	R
WMB312/DS-13295	Thailand via University of Singapore	Thailand	EAI	SAMN07236334	R	R	R	n/a	R	R	R	R
WMB255/DS-18938	Thailand via University of Singapore	Thailand	Beijing	SAMN07236279	R	R	R	n/a	R	R	R	U
WMB410/DS-15561	Thailand via University of Singapore	Thailand	EAI	SAMN07236428	S	S	S	n/a	S	S	S	S
WMB359/DS-21517	Thailand via University of Singapore	Thailand	Beijing	SAMN07236378	R	R	n/a	n/a	R	R	R	R
WMB374/DS-24426	Thailand via University of Singapore	Thailand	lineage4	SAMN07236392	R	R	S	n/a	S	S	S	S
WMB303/DS-14490	Thailand via University of Singapore	Thailand	Beijing	SAMN07236325	R	R	R	n/a	R	R	R	R
WMB262/DS-20535	Thailand via University of Singapore	Thailand	Beijing	SAMN07236286	R	R	n/a	n/a	R	R	R	R
WMB320/DS-15559	Thailand via University of Singapore	Thailand	Beijing	SAMN07236342	R	R	S	n/a	R	F	F	S
WMB298/CSF-4374	Thailand via University of Singapore	Thailand	EAI	SAMN07236320	R	R	S	n/a	S	S	S	S
WMB465/47-17522	Thailand via University of Singapore	Thailand	Beijing	SAMN07236478	S	S	S	n/a	S	S	S	S
WMB431/43-13037	Thailand via University of Singapore	Thailand	EAI	SAMN07236449	R	S	S	n/a	R	U	S	S
WMB299/DS-34062	Thailand via University of Singapore	Thailand	Beijing	SAMN07236321	R	R	R	n/a	R	R	U	S
WMB353/DS-20813	Thailand via University of Singapore	Thailand	Beijing	SAMN07236372	R	R	n/a	n/a	R	R	S	S
WMB352/DS-20670	Thailand via University of Singapore	Thailand	Beijing	SAMN07236371	R	R	R	n/a	R	R	R	R
WMB257/DS-19488	Thailand via University of Singapore	Thailand	Beijing	SAMN07236281	R	R	R	n/a	R	R	R	S
WBB268/DS-17984	Thailand via University of Singapore	Thailand	Beijing	SAMN07236523	R	R	S	n/a	R	R	S	S

WMB358/DS-21330	Thailand via University of Singapore	Thailand	Beijing	SAMN07236377	R	R	n/a	n/a	R	R	R	R
WMB390/DS-31067	Thailand via University of Singapore	Thailand	Beijing	SAMN07236408	R	R	S	n/a	R	R	R	U
WMB281/DS-29456	Thailand via University of Singapore	Thailand	Beijing	SAMN07236303	R	R	R	n/a	R	R	R	R
WMB348/DS-20433	Thailand via University of Singapore	Thailand	lineage4	SAMN07236367	R	R	R	n/a	R	F	S	S
WBB258/DS-16179	Thailand via University of Singapore	Thailand	Beijing	SAMN07236514	R	R	S	n/a	R	R	U	F
WMB395/DS-32353	Thailand via University of Singapore	Thailand	Beijing	SAMN07236413	R	R	R	n/a	R	R	R	U
WMB226/DS-07687	Thailand via University of Singapore	Thailand	Beijing	SAMN07236252	R	R	R	n/a	R	R	R	R
WMB246/DS-10200	Thailand via University of Singapore	Thailand	Beijing	SAMN07236270	R	R	R	n/a	R	R	U	F
WMB411/DS-15599	Thailand via University of Singapore	Thailand	EAI	SAMN07236429	S	S	S	n/a	S	S	S	S
WMB391/DS-31224	Thailand via University of Singapore	Thailand	Beijing	SAMN07236409	R	R	R	n/a	R	R	R	U
WMB284/DS-30312	Thailand via University of Singapore	Thailand	Beijing	SAMN07236306	R	R	R	n/a	R	R	R	S
WMB442/CSF-1449	Thailand via University of Singapore	Thailand	S-type	SAMN07236459	S	S	S	n/a	U	S	S	S
WMB233/DS-16503	Thailand via University of Singapore	Thailand	Beijing	SAMN07236258	R	R	R	n/a	R	F	U	S
WMB313/DS-13949	Thailand via University of Singapore	Thailand	Beijing	SAMN07236335	R	R	R	n/a	R	R	F	S
WMB412/DS-16280	Thailand via University of Singapore	Thailand	EAI	SAMN07236430	S	S	S	n/a	S	S	S	S
WMB338/DS-19351	Thailand via University of Singapore	Thailand	Beijing	SAMN07236358	R	R	S	n/a	R	R	S	S
WMB242/DS-10110	Thailand via University of Singapore	Thailand	Beijing	SAMN07236266	R	R	R	n/a	R	R	R	S
WMB230/DS-08230	Thailand via University of Singapore	Thailand	Beijing	SAMN07360947	R	R	R	n/a	R	R	R	U
WMB399/DS-33474	Thailand via University of Singapore	Thailand	S-type	SAMN07236417	R	R	R	n/a	R	R	R	U
WMB369/DS-23747	Thailand via University of Singapore	Thailand	Beijing	SAMN07236388	R	R	R	n/a	R	R	R	S
WMB373/DS-24418	Thailand via University of Singapore	Thailand	S-type	SAMN07236391	R	R	S	n/a	S	S	S	S
WMB252/DS-16224	Thailand via University of Singapore	Thailand	Beijing	SAMN07236276	R	R	R	n/a	R	R	R	S
WMB459/CSF-3518	Thailand via University of Singapore	Thailand	Beijing	SAMN07236472	S	S	S	n/a	S	S	S	S
WMB437/CSF-3943	Thailand via University of Singapore	Thailand	EAI	SAMN07236455	S	S	S	n/a	S	S	S	S
WMB336/DS-19290	Thailand via University of Singapore	Thailand	Beijing	SAMN07236356	R	R	S	n/a	R	R	R	S
WMB263/DS-20908	Thailand via University of Singapore	Thailand	Beijing	SAMN07236287	R	R	n/a	n/a	R	R	R	F
WMB270/DS-21358	Thailand via University of Singapore	Thailand	Beijing	SAMN07236294	R	R	n/a	n/a	R	F	S	S
WMB402/DS-18501	Thailand via University of Singapore	Thailand	Beijing	SAMN07236420	R	R	S	n/a	R	R	S	S
WMB372/DS-24268	Thailand via University of Singapore	Thailand	Beijing	SAMN07360952	R	R	R	n/a	R	R	R	R
WBB282/DS-30056	Thailand via University of Singapore	Thailand	Beijing	SAMN07236536	R	R	R	n/a	R	R	R	R
WMB424/43-05286	Thailand via University of Singapore	Thailand	Beijing	SAMN07236442	S	S	S	n/a	S	S	S	S
WMB241/DS-17290	Thailand via University of Singapore	Thailand	Beijing	SAMN07236265	R	R	R	n/a	R	R	R	R
WMB243/DS-11745	Thailand via University of Singapore	Thailand	Beijing	SAMN07236267	R	R	R	n/a	R	R	R	R
WMB426/43-11359	Thailand via University of Singapore	Thailand	EAI	SAMN07236444	R	R	S	n/a	R	R	S	S
WMB455/CSF-3384	Thailand via University of Singapore	Thailand	Beijing	SAMN07236470	S	S	S	n/a	S	S	S	S
WMB360/DS-21566	Thailand via University of Singapore	Thailand	Beijing	SAMN07236379	R	R	n/a	n/a	R	R	R	R
WMB239/DS-11380	Thailand via University of Singapore	Thailand	EAI	SAMN07236263	R	R	R	n/a	S	R	R	R
WMB434/43-17298	Thailand via University of Singapore	Thailand	EAI	SAMN07236452	S	S	S	n/a	S	S	S	S
WMB337/DS-19305	Thailand via University of Singapore	Thailand	Beijing	SAMN07236357	R	R	R	n/a	R	R	R	R
WMB240/DS-15684	Thailand via University of Singapore	Thailand	Beijing	SAMN07236264	R	R	R	n/a	R	R	R	R
WMB308/DS-14191	Thailand via University of Singapore	Thailand	Beijing	SAMN07236330	R	R	R	n/a	R	R	R	R
WMB324/DS-16059	Thailand via University of Singapore	Thailand	Beijing	SAMN07236345	R	R	S	n/a	R	R	S	S
WMB438/45-12339	Thailand via University of Singapore	Thailand	EAI	SAMN07236456	R	S	S	n/a	R	S	S	S
WBB279/DS-29128	Thailand via University of Singapore	Thailand	Beijing	SAMN07236533	R	R	R	n/a	R	R	R	U
WMB433/43-15038	Thailand via University of Singapore	Thailand	EAI	SAMN07236451	S	S	S	n/a	S	S	S	S
WMB349/DS-20500	Thailand via University of Singapore	Thailand	Beijing	SAMN07236368	R	R	R	n/a	R	R	F	R
WMB483/48-10477	Thailand via University of Singapore	Thailand	Beijing	SAMN07236492	R	R	S	n/a	R	R	S	S
WBB267/DS-17841	Thailand via University of Singapore	Thailand	Beijing	SAMN07360953	R	R	R	n/a	R	R	R	U
WMB258/DS-19501	Thailand via University of Singapore	Thailand	lineage4	SAMN07236282	R	R	R	n/a	R	R	S	S
WMB425/43-08441	Thailand via University of Singapore	Thailand	Beijing	SAMN07236443	S	S	S	n/a	S	S	S	S
WBB269/DS-18810	Thailand via University of Singapore	Thailand	Beijing	SAMN07236524	R	R	R	n/a	R	R	R	R
WMB365/DS-23018	Thailand via University of Singapore	Thailand	EAI	SAMN07236384	R	R	S	n/a	R	R	S	S
WBB286/DS-32512	Thailand via University of Singapore	Thailand	Beijing	SAMN07236540	R	R	S	n/a	R	R	S	R
WMB325/DS-16080	Thailand via University of Singapore	Thailand	Tur	SAMN07236346	R	R	S	n/a	R	R	R	U
WMB428/43-11791	Thailand via University of Singapore	Thailand	Beijing	SAMN07236446	S	S	S	n/a	S	S	S	S
WMB317/DS-15310	Thailand via University of Singapore	Thailand	Beijing	SAMN07236339	R	R	S	n/a	R	R	R	F
WMB327/DS-16288	Thailand via University of Singapore	Thailand	EAI	SAMN07236348	R	R	S	n/a	R	S	U	S
WBB277/DS-27099	Thailand via University of Singapore	Thailand	Beijing	SAMN07236531	R	R	n/a	n/a	S	S	S	S
WMB301/DS-13878	Thailand via University of Singapore	Thailand	S-type	SAMN07236323	R	R	R	n/a	R	R	R	F
WMB307/DS-25358	Thailand via University of Singapore	Thailand	Beijing	SAMN07236329	R	R	n/a	n/a	R	R	S	S
15.0609734	Birmingham	UK	LAM		S	S	S	S	S	S	S	S
16.0617190	Birmingham	UK	Delhi		S	S	S	S	S	S	S	S
13.0614462	Birmingham	UK	Delhi		S	S	S	S	S	S	S	S
14.0615374	Birmingham	UK	Delhi		S	S	S	S	U	S	S	S
14.0609451	Birmingham	UK	Beijing		R	R	R	R	R	R	R	R
13.0613340	Birmingham	UK	Delhi		R	S	S	S	R	S	S	S
10.0603178	Birmingham	UK	Delhi		S	S	S	S	S	S	S	S
16.0609105	Birmingham	UK	EAI		S	S	S	n/a	S	S	S	S
12.0617128	Birmingham	UK	X-type		S	S	S	S	S	F	S	S
13.0613511	Birmingham	UK	LAM		S	S	S	S	S	S	S	S
14.0603170	Birmingham	UK	Haarlem		S	S	S	S	S	S	U	S
13.0611347	Birmingham	UK	EAI		S	S	S	S	S	S	U	S
13.0609916	Birmingham	UK	X-type		S	S	S	S	S	S	S	S
12.0611900	Birmingham	UK	Delhi		S	S	S	S	S	S	S	S
16.0617443	Birmingham	UK	lineage4		S	S	S	S	S	S	U	S
11.0609336	Birmingham	UK	Delhi		S	S	S	S	S	S	S	S
15.0607641	Birmingham	UK	West Africar		S	S	S	S	S	U	U	S
13.0601558	Birmingham	UK	Delhi		S	S	S	S	S	S	S	S
13.0611337	Birmingham	UK	Beijing		S	S	S	S	S	S	S	S
16.0601559	Birmingham	UK	Delhi		S	S	S	S	S	U	S	S
16.0618558	Birmingham	UK	EAI		S	S	S	S	S	S	S	S
12.0610317	Birmingham	UK	Delhi		S	S	S	S	S	S	S	S
16.0607896	Birmingham	UK	lineage4		S	S	S	S	S	S	S	S
16.0601742	Birmingham	UK	S-type		S	S	S	S	S	S	S	S
12.0612511	Birmingham	UK	LAM		S	S	S	S	S	S	S	S
12.0617758	Birmingham	UK	Delhi		S	S	S	S	S	S	S	S
15.0607188	Birmingham	UK	Delhi		S	S	S	S	S	S	S	S
13.0612204	Birmingham	UK	Ural		R	R	S	R	R	R	R	U
15.0613372	Birmingham	UK	LAM		S	S	S	S	S	S	U	S
15.0601103	Birmingham	UK	Delhi		S	S	S	S	S	U	U	S
16.0601196	Birmingham	UK	X-type		S	S	S	S	S	S	S	S
16.0607929	Birmingham	UK	lineage4		S	S	S	S	S	S	S	S
16.0607049	Birmingham	UK	lineage4		S	S	S	S	S	S	S	S
15.0604529	Birmingham	UK	Haarlem		S	S	S	S	S	S	S	S
16.0601071	Birmingham	UK	lineage4		S	S	S	S	U	S	S	S
16.0607449	Birmingham	UK	Beijing		S	S	S	S	S	S	S	S
14.0609826	Birmingham	UK	Delhi		S	S	S	S	S	S	S	S
13.0613477	Birmingham	UK	Haarlem		S	S	S	S	S	S	S	S
11.0604679	Birmingham	UK	Haarlem		S	S	S	S	S	S	S	S
15.0601840	Birmingham	UK	Beijing		S	S	S	S	S	S	S	S
12.0613631	Birmingham	UK	Beijing		S	S	S	S	S	S	S	S
15.0601518	Birmingham	UK	Delhi		S	S	S	S	S	S	S	S
16.0606831	Birmingham	UK			S	S	S	S	S	S	S	S
13.0601536	Birmingham	UK	X-type		S	S	S	S	S	S	S	S
13.0613192	Birmingham	UK	Haarlem		S	S	S	S	S	S	S	S
16.0618427	Birmingham	UK	Delhi		S	S	S	S	S	S	S	F
14.0602189	Birmingham	UK	Delhi		S	S	S	S	S	S	S	S
13.0608469	Birmingham	UK	Delhi		S	S	S	S	S	S	S	S
13.0602359	Birmingham	UK	Delhi		R	R	S	S	R	R	S	S
15.0601102	Birmingham	UK	Delhi		S	S	S	S	S	S	S	S
12.0616369	Birmingham	UK	Delhi		S	S	S	S	S	S	S	S
13.0606210	Birmingham	UK	Beijing		S	S	S	S	S	S	S	S
16.0607897	Birmingham	UK	EAI		S	S	S	n/a	S	S	S	S
12.0615235	Birmingham	UK	Delhi		S	S	S	S	S	S	S	S
15.0601760	Birmingham	UK	M. orygis		S	S	S	S	S	S	S	S
12.0615275	Birmingham	UK	Delhi		S	S	S	S	S	S	S	S

15.0601892	Birmingham	UK	Haarlem	S	S	S	S	S	S	S	S
14.0600769	Birmingham	UK	Delhi	S	S	S	S	S	S	S	S
13.0609028	Birmingham	UK	Beijing	S	S	S	S	S	S	U	S
15.0601193	Birmingham	UK	lineage4	S	S	S	S	S	S	S	S
15.0607395	Birmingham	UK	Delhi	n/a	S	S	n/a	S	S	U	S
12.0610917	Birmingham	UK	Delhi	S	S	S	S	S	S	S	S
15.0607739	Birmingham	UK	Beijing	R	R	S	R	R	R	R	R
15.0613559	Birmingham	UK	LAM	S	S	S	S	S	S	S	S
16.0600326	Birmingham	UK	LAM	S	S	S	S	S	S	S	S
13.0613595	Birmingham	UK	Beijing	n/a	S	S	S	S	S	S	S
13.0607701	Birmingham	UK	EAI	S	S	S	S	S	S	S	S
13.0603777	Birmingham	UK	Delhi	S	S	S	S	S	S	S	S
14.0604828	Birmingham	UK	lineage4	S	S	S	S	S	S	S	S
14.0600008	Birmingham	UK	Delhi	S	S	S	S	S	S	S	S
14.0600712	Birmingham	UK	EAI	S	S	S	S	U	S	S	S
13.0605719	Birmingham	UK	EAI	S	S	S	S	F	S	S	S
14.0600197	Birmingham	UK	Delhi	S	S	S	S	S	S	S	S
12.0614468	Birmingham	UK	LAM	S	S	S	S	S	S	S	S
16.0602257	Birmingham	UK	Delhi	S	S	S	S	S	S	S	S
15.0612584	Birmingham	UK	Beijing	R	R	R	n/a	R	R	R	R
15.0607036	Birmingham	UK	Delhi	S	S	S	S	S	S	S	S
14.0615598	Birmingham	UK	Delhi	S	S	S	S	S	S	S	S
11.0601116	Birmingham	UK	EAI	R	S	S	S	S	S	S	S
13.0610517	Birmingham	UK	Delhi	S	S	S	S	S	S	S	S
14.0609620	Birmingham	UK	lineage4	S	S	S	S	S	S	S	S
15.0607222	Birmingham	UK	Haarlem	S	S	S	S	S	S	S	S
13.0613589	Birmingham	UK	Beijing	S	S	S	S	S	S	S	S
14.0602321	Birmingham	UK	lineage4	S	S	S	S	S	S	S	S
11.0600740	Birmingham	UK	Delhi	S	S	S	S	S	S	S	S
16.0603575	Birmingham	UK		S	S	S	S	S	S	S	S
15.0601928	Birmingham	UK	EAI	S	S	S	S	S	S	S	S
16.0607574	Birmingham	UK	lineage4	S	S	S	S	U	S	S	S
16.0618549	Birmingham	UK	LAM	S	S	S	S	S	S	S	S
13.0604541	Birmingham	UK	LAM	S	S	S	S	S	S	S	S
16.0607003	Birmingham	UK	Delhi	S	S	S	S	S	S	S	S
12.0616140	Birmingham	UK	Delhi	S	S	S	S	S	S	S	S
14.0611651	Birmingham	UK	EAI	S	S	S	S	S	S	S	S
15.0601074	Birmingham	UK	lineage4	S	S	S	S	S	S	S	S
13.0608430	Birmingham	UK	Delhi	S	S	S	S	S	S	S	S
16.0601769	Birmingham	UK	LAM	S	S	S	S	S	S	U	S
16.0610889	Birmingham	UK		S	S	S	S	S	R	S	S
15.0601842	Birmingham	UK		S	S	S	S	S	S	S	S
16.0618261	Birmingham	UK	LAM	S	S	S	S	S	S	S	S
15.0601447	Birmingham	UK	M. bovis	S	S	S	R	S	S	S	R
15.0604401	Birmingham	UK	LAM	S	S	S	S	S	S	S	S
14.0603683	Birmingham	UK	Ural	S	S	S	S	S	S	S	S
16.0607144	Birmingham	UK	Delhi	S	S	S	S	S	S	S	S
16.0601564	Birmingham	UK	Cameroon	S	S	S	S	S	S	S	S
12.0617742	Birmingham	UK	EAI	S	S	S	S	S	S	S	S
13.0613123	Birmingham	UK		S	S	S	S	S	S	S	S
15.0614224	Birmingham	UK	LAM	S	S	S	S	S	S	S	S
14.0616153	Birmingham	UK	Delhi	n/a	n/a	n/a	S	F	F	S	S
13.0610752	Birmingham	UK	Tur	S	S	S	S	S	S	S	S
13.0613546	Birmingham	UK	Cameroon	S	S	S	S	S	S	S	S
16.0607899	Birmingham	UK	Haarlem	S	S	S	S	S	S	S	S
15.0603719	Birmingham	UK	Haarlem	S	S	S	S	S	S	S	S
13.0605677	Birmingham	UK	lineage4	S	S	S	S	S	S	S	S
13.0613247	Birmingham	UK	Beijing	S	S	S	S	S	S	S	S
14.0600841	Birmingham	UK	EAI	S	S	S	S	S	S	S	S
13.0607981	Birmingham	UK	Delhi	S	S	S	S	S	S	S	S
13.0602105	Birmingham	UK		S	S	S	S	S	S	S	S
14.0600953	Birmingham	UK	Delhi	S	S	S	S	S	S	U	S
16.0607683	Birmingham	UK	X-type	S	S	S	S	S	S	S	S
14.0609787	Birmingham	UK	Delhi	S	S	S	S	S	F	S	S
14.0600279	Birmingham	UK	X-type	S	S	S	S	S	S	U	S
12.0927693	Birmingham	UK	Delhi	S	S	S	S	S	S	S	S
16.0602864	Birmingham	UK	S-type	S	S	S	S	S	S	S	S
12.0601002	Birmingham	UK	Haarlem	S	S	S	S	S	S	S	S
14.0613755	Birmingham	UK	Haarlem	S	S	S	S	S	S	S	S
14.0600824	Birmingham	UK	lineage4	S	S	S	S	S	S	S	S
15.0609511	Birmingham	UK	Beijing	R	R	S	n/a	R	R	S	F
15.0613441	Birmingham	UK	X-type	S	S	S	S	S	S	S	S
14.0609935	Birmingham	UK	Uganda	S	S	S	S	S	S	S	S
16.0607712	Birmingham	UK	Delhi	S	S	S	S	S	S	S	S
14.0603481	Birmingham	UK	Delhi	R	R	R	S	R	R	R	R
13.0604905	Birmingham	UK	LAM	S	S	S	S	S	S	S	S
10.0603374	Birmingham	UK	lineage4	S	S	S	S	S	S	S	S
16.0610784	Birmingham	UK	Delhi	S	S	S	S	S	S	S	S
16.0609808	Birmingham	UK	lineage4	S	S	S	S	S	U	S	S
16.0601587	Birmingham	UK	EAI	S	S	S	n/a	S	S	S	S
16.0618265	Birmingham	UK	Cameroon	S	S	S	S	S	S	U	S
13.0608439	Birmingham	UK	LAM	S	S	S	S	S	S	S	S
16.0611493	Birmingham	UK	Cameroon	S	S	S	S	S	S	S	S
14.0607096	Birmingham	UK	X-type	S	S	S	S	S	S	S	S
13.0606680	Birmingham	UK	Delhi	S	S	S	S	S	S	S	S
10.0605209	Birmingham	UK	Uganda	S	S	S	S	S	S	S	S
15.0601380	Birmingham	UK	West Africar	S	S	S	S	S	S	U	S
12.0612131	Birmingham	UK	EAI	S	S	S	S	S	S	U	S
14.0616048	Birmingham	UK	lineage4	S	S	S	S	S	S	S	S
14.0603921	Birmingham	UK	X-type	R	S	R	R	F	R	R	F
16.0610279	Birmingham	UK	Delhi	S	S	S	S	S	S	S	S
16.0601106	Birmingham	UK	Cameroon	R	S	S	S	R	S	S	S
15.0605480	Birmingham	UK	X-type	R	R	S	n/a	R	R	R	F
14.0600157	Birmingham	UK	lineage4	S	S	S	S	S	S	S	S
14.0611575	Birmingham	UK	Delhi	S	S	S	S	S	S	S	S
12.0612892	Birmingham	UK	Beijing	S	S	S	S	S	S	S	S
15.0607659	Birmingham	UK	Delhi	R	R	S	S	R	R	S	S
12.0611870	Birmingham	UK	M. orygis	S	S	S	S	S	S	S	S
15.0607802	Birmingham	UK	Beijing	R	R	n/a	R	R	R	R	R
16.0607131	Birmingham	UK	Ural	R	S	S	S	R	S	S	S
13.0607356	Birmingham	UK	Beijing	S	S	S	S	S	S	S	S
14.0612987	Birmingham	UK	Delhi	S	S	S	S	S	S	S	S
15.0607066	Birmingham	UK	Delhi	S	S	S	S	S	S	S	S
15.0601621	Birmingham	UK	EAI	R	S	S	S	S	S	S	S
14.0612764	Birmingham	UK	Delhi	S	S	S	S	S	S	U	S
16.0618537	Birmingham	UK	Beijing	S	S	S	S	S	S	U	S
12.0614420	Birmingham	UK	Delhi	S	S	S	S	S	S	S	S
15.0601553	Birmingham	UK	Beijing	S	S	S	S	S	S	S	S
16.0604896	Birmingham	UK	S-type	S	S	S	S	S	S	S	S
16.0618494	Birmingham	UK	BCG	S	S	S	R	S	S	S	R
15.0601154	Birmingham	UK	Beijing	S	S	S	S	S	S	U	S
15.0607073	Birmingham	UK	X-type	n/a	n/a	S	n/a	S	F	S	S
15.0615230	Birmingham	UK	Haarlem	S	S	S	S	S	S	S	S
12.0616799	Birmingham	UK	Delhi	S	S	S	S	S	S	S	S
13.0607893	Birmingham	UK	Beijing	S	S	S	S	S	S	S	S
16.0601745	Birmingham	UK	Haarlem	S	S	S	S	S	S	S	S

14.0609751	Birmingham	UK	Delhi	S	S	S	S	S	S	S	S
13.0611150	Birmingham	UK	EAI	S	S	S	S	S	S	S	S
15.0613384	Birmingham	UK	lineage4	S	S	S	S	S	S	S	S
16.0618501	Birmingham	UK	Delhi	S	S	S	S	S	S	S	S
16.0618346	Birmingham	UK	Delhi	S	S	S	S	S	S	S	S
16.0604301	Birmingham	UK	Delhi	S	S	S	S	S	S	S	S
14.0600715	Birmingham	UK	lineage4	S	S	S	S	S	S	S	S
14.0600760	Birmingham	UK	lineage4	S	S	S	S	S	S	S	S
13.0609451	Birmingham	UK	EAI	S	S	S	S	S	S	S	S
12.0613656	Birmingham	UK	Beijing	S	S	S	S	S	S	S	S
13.0609878	Birmingham	UK	Delhi	S	S	S	S	S	S	S	S
14.0600791	Birmingham	UK	Delhi	S	S	S	S	S	S	S	S
13.0613207	Birmingham	UK	Delhi	S	S	S	S	S	S	S	S
15.0601116	Birmingham	UK	lineage4	S	S	S	S	S	S	U	S
15.0611664	Birmingham	UK	Delhi	S	S	S	S	S	S	S	S
13.0613552	Birmingham	UK	Delhi	S	S	S	S	S	S	U	S
16.0618518	Birmingham	UK	lineage4	S	S	S	S	S	S	S	S
12.0615228	Birmingham	UK	Delhi	S	S	S	S	S	S	S	S
15.0607160	Birmingham	UK	Delhi	S	S	S	S	S	S	S	S
16.0604639	Birmingham	UK	M. bovis	S	S	S	R	S	S	S	R
14.0609326	Birmingham	UK	LAM	S	S	S	S	S	S	S	S
16.0610438	Birmingham	UK	Delhi	S	S	S	S	S	S	S	S
10.0604992	Birmingham	UK	S-type	S	S	S	S	S	S	S	S
14.0602981	Birmingham	UK	Delhi	S	S	S	S	S	S	S	S
14.0600232	Birmingham	UK	Delhi	S	S	S	S	S	S	S	S
14.0600968	Birmingham	UK		S	S	S	S	S	S	U	S
13.0608572	Birmingham	UK	X-type	S	S	S	S	S	S	S	S
15.0607529	Birmingham	UK	EAI	S	S	S	S	S	U	S	S
16.0601859	Birmingham	UK	Delhi	S	S	S	S	S	S	S	S
15.0601192	Birmingham	UK	Haarlem	S	S	S	S	S	S	S	S
12.0610875	Birmingham	UK	Delhi	S	S	S	S	S	S	S	S
14.0609245	Birmingham	UK	Delhi	S	S	S	S	S	S	S	S
16.0603317	Birmingham	UK	lineage4	S	S	S	S	S	S	S	S
14.0600418	Birmingham	UK	Haarlem	S	S	S	S	S	S	S	S
14.0602059	Birmingham	UK	LAM	R	S	S	S	R	S	S	S
15.0607338	Birmingham	UK	EAI	S	S	S	S	S	S	S	S
12.0612788	Birmingham	UK	LAM	S	S	S	S	S	S	S	S
14.0609529	Birmingham	UK	EAI	S	S	S	S	S	S	S	S
12.0615219	Birmingham	UK	Tur	R	S	S	S	R	S	S	S
13.0601211	Birmingham	UK	Tur	S	S	S	S	S	S	S	S
14.0600472	Birmingham	UK	LAM	S	S	S	S	S	S	S	S
15.0606095	Birmingham	UK	EAI	R	S	S	n/a	R	S	S	S
12.0616381	Birmingham	UK	EAI	S	S	S	S	S	S	U	S
16.0601830	Birmingham	UK	Uganda	S	S	S	S	S	S	S	S
15.0607931	Birmingham	UK	lineage4	S	S	S	S	S	S	S	S
14.0609481	Birmingham	UK	Beijing	R	R	R	R	R	R	R	R
16.0617386	Birmingham	UK	Beijing	S	S	S	S	S	S	S	S
12.0614867	Birmingham	UK	Ural	S	S	S	S	S	S	S	S
15.0601455	Birmingham	UK	Delhi	S	S	S	S	S	S	S	S
13.0613205	Birmingham	UK	EAI	R	S	S	S	R	S	U	S
13.0615665	Birmingham	UK	Beijing	R	R	S	n/a	R	R	R	U
15.0601460	Birmingham	UK	Tur	S	S	S	S	S	S	S	S
16.0607992	Birmingham	UK	EAI	S	S	S	S	S	S	S	S
15.0601431	Birmingham	UK	EAI	S	S	S	S	S	S	S	S
15.0601591	Birmingham	UK	Delhi	S	S	S	S	S	S	S	S
13.0610508	Birmingham	UK	Cameroon	R	S	S	S	R	S	S	S
16.0618344	Birmingham	UK	EAI	S	S	S	n/a	S	S	S	S
14.0603958	Birmingham	UK	lineage4	S	S	S	S	S	S	S	S
14.0600834	Birmingham	UK	Delhi	S	S	S	S	S	S	S	S
16.0617147	Birmingham	UK	Delhi	S	S	S	S	S	S	S	S
14.0600072	Birmingham	UK	EAI	S	S	S	S	S	S	U	S
15.0600271	Birmingham	UK	Delhi	R	S	S	S	R	F	S	S
12.0927474	Birmingham	UK	Beijing	S	S	S	S	S	S	S	S
16.0607369	Birmingham	UK	lineage4	S	S	S	S	U	S	S	S
13.0606625	Birmingham	UK	X-type	S	S	S	S	S	S	S	S
13.0607323	Birmingham	UK	Uganda	S	S	S	S	S	S	S	S
12.0615645	Birmingham	UK	Delhi	S	S	S	S	S	S	S	S
13.0607994	Birmingham	UK	Haarlem	S	S	S	S	S	S	S	S
15.0607082	Birmingham	UK	Delhi	S	S	S	S	S	S	S	S
15.0601419	Birmingham	UK	Haarlem	S	S	S	S	S	S	S	S
13.0613313	Birmingham	UK	Haarlem	S	S	S	S	S	S	S	S
16.0617570	Birmingham	UK	Delhi	S	S	S	S	S	F	S	S
14.0600890	Birmingham	UK	Delhi	S	S	S	S	S	S	S	S
15.0607837	Birmingham	UK	Beijing	S	S	S	S	S	S	S	S
14.0600822	Birmingham	UK	X-type	S	S	S	S	S	S	S	S
14.0600646	Birmingham	UK	Delhi	S	S	S	S	S	S	S	S
13.0608692	Birmingham	UK	Cameroon	S	S	S	S	S	S	S	S
14.0606710	Birmingham	UK	LAM	S	S	S	S	S	S	S	S
16.0618566	Birmingham	UK	lineage4	S	S	S	S	S	S	S	S
16.0600854	Birmingham	UK	Delhi	S	S	S	S	S	S	S	S
13.0610479	Birmingham	UK	Delhi	S	S	S	S	S	S	S	S
15.0607157	Birmingham	UK	Tur	R	S	S	S	R	S	S	S
15.0613496	Birmingham	UK	Delhi	S	S	S	S	S	S	S	S
16.0608290	Birmingham	UK		S	S	S	S	S	S	S	S
16.0607011	Birmingham	UK	LAM	S	S	S	S	S	S	S	S
15.0608047	Birmingham	UK	Delhi	S	S	S	S	S	S	S	S
13.0613183	Birmingham	UK	X-type	S	n/a	S	S	S	R	S	S
14.0609667	Birmingham	UK	lineage4	S	S	S	S	S	S	S	S
13.0605682	Birmingham	UK	S-type	S	S	S	S	S	S	S	S
15.0607921	Birmingham	UK	EAI	S	S	S	S	S	S	S	S
14.0609303	Birmingham	UK	Delhi	S	S	n/a	S	S	S	S	S
16.0607257	Birmingham	UK	lineage4	S	S	S	S	S	S	S	S
15.0601461	Birmingham	UK	EAI	S	S	S	S	S	S	S	S
09.0600421	Birmingham	UK	Delhi	S	S	S	S	S	S	S	S
16.0605013	Birmingham	UK	West Africar	S	S	S	S	S	U	U	S
13.0605720	Birmingham	UK	Haarlem	S	S	S	S	S	S	S	S
13.0607499	Birmingham	UK	Beijing	S	S	S	S	S	S	S	S
16.0601660	Birmingham	UK	Delhi	S	S	S	S	S	S	S	S
13.0607383	Birmingham	UK	lineage4	S	S	S	S	S	S	S	S
16.0605385	Birmingham	UK	Delhi	S	S	S	S	S	S	S	S
14.0600211	Birmingham	UK	lineage4	S	S	S	S	S	S	S	S
16.0601329	Birmingham	UK	Delhi	S	S	S	S	S	U	S	S
15.0601933	Birmingham	UK	Delhi	R	R	S	S	R	R	S	S
16.0601171	Birmingham	UK	Delhi	S	S	S	S	S	S	S	S
15.0607835	Birmingham	UK	Delhi	S	S	S	S	S	F	S	S
13.0613046	Birmingham	UK	lineage4	S	S	S	S	S	S	S	S
15.0602904	Birmingham	UK	Beijing	R	R	S	S	R	R	S	F
16.0613084	Birmingham	UK	S-type	S	S	S	n/a	S	S	S	R
16.0607442	Birmingham	UK	Delhi	S	S	S	S	S	S	S	S
14.0607804	Birmingham	UK		S	S	S	S	S	S	S	S
14.0600406	Birmingham	UK	Haarlem	S	S	S	S	S	S	S	S
12.0926048	Birmingham	UK	S-type	S	S	S	S	S	S	S	S
14.0608109	Birmingham	UK	M. bovis	S	S	S	R	S	S	S	R
13.0609268	Birmingham	UK	Delhi	S	S	S	S	S	S	S	S
14.0600309	Birmingham	UK	EAI	S	S	S	S	S	S	S	S
15.0614506	Birmingham	UK	Beijing	R	S	S	n/a	R	S	R	S

14.0600881	Birmingham	UK	X-type	S	S	S	S	S	S	S	S
13.0610729	Birmingham	UK	Delhi	S	S	S	S	S	S	S	S
13.0614500	Birmingham	UK	LAM	S	S	S	S	S	S	S	S
16.0601527	Birmingham	UK	Beijing	S	S	S	S	S	S	S	S
12.0608580	Birmingham	UK	Delhi	S	S	S	S	S	U	S	S
16.0607609	Birmingham	UK	Haarlem	S	S	S	S	S	S	S	S
16.0606604	Birmingham	UK	Haarlem	S	S	S	S	S	S	S	S
16.0601999	Birmingham	UK	Delhi	S	S	S	S	S	S	S	S
16.0607165	Birmingham	UK	Delhi	S	S	S	S	S	S	S	S
12.0927699	Birmingham	UK	lineage4	S	S	S	S	S	S	S	S
16.0607977	Birmingham	UK	Delhi	S	S	S	S	S	S	S	S
15.0601633	Birmingham	UK	Delhi	S	S	S	S	S	S	S	S
13.0607961	Birmingham	UK	LAM	S	S	S	S	S	U	U	S
14.0609960	Birmingham	UK	Beijing	R	S	S	S	R	S	R	S
13.0617294	Birmingham	UK	Delhi	S	S	S	n/a	S	S	S	S
12.0613638	Birmingham	UK	lineage4	S	S	S	S	S	S	S	S
12.0612203	Birmingham	UK	EAI	S	S	S	S	S	S	S	S
16.0601794	Birmingham	UK	Uganda	S	S	S	S	S	S	S	S
13.0604896	Birmingham	UK	X-type	S	S	S	S	S	S	S	S
13.0615631	Birmingham	UK	West Africar	S	S	S	S	S	S	U	S
13.0602577	Birmingham	UK	Delhi	S	S	S	S	S	S	S	S
15.0615401	Birmingham	UK	Delhi	S	S	S	S	S	S	S	S
13.0613309	Birmingham	UK	Delhi	S	S	S	S	S	S	S	S
15.0602762	Birmingham	UK	lineage4	S	S	S	S	S	S	S	S
14.0600565	Birmingham	UK	lineage4	S	S	S	S	S	S	S	S
14.0600070	Birmingham	UK	Haarlem	S	S	S	S	S	S	S	S
15.0601441	Birmingham	UK	Haarlem	S	S	S	S	S	S	U	S
15.0607698	Birmingham	UK	lineage4	S	S	S	S	S	S	S	S
14.0609132	Birmingham	UK	M. bovis	S	S	S	R	S	S	U	R
14.0600910	Birmingham	UK	M. bovis	S	S	S	R	F	S	U	R
16.0617500	Birmingham	UK	Delhi	S	S	S	S	S	S	S	S
13.0610759	Birmingham	UK	EAI	S	S	S	S	S	S	S	S
14.0616171	Birmingham	UK	Delhi	S	S	S	S	S	S	S	S
13.0605180	Birmingham	UK	Delhi	S	S	S	S	S	S	S	U
14.0600622	Birmingham	UK	Beijing	S	S	S	S	S	S	S	S
16.0601530	Birmingham	UK	West Africar	S	S	S	S	S	U	U	S
15.0601298	Birmingham	UK	Delhi	S	S	S	S	S	S	U	S
14.0600129	Birmingham	UK	Delhi	S	S	S	S	S	S	S	S
11.0607091	Birmingham	UK	Haarlem	n/a	S	S	S	S	S	S	S
13.0604053	Birmingham	UK	Delhi	S	S	S	S	S	S	S	S
14.0600534	Birmingham	UK	X-type	S	S	S	S	S	S	S	S
15.0601979	Birmingham	UK	Delhi	S	S	S	S	S	S	S	S
13.0618062	Birmingham	UK	Cameroon	S	S	S	S	S	S	U	S
13.0600045	Birmingham	UK	Delhi	S	S	S	S	S	S	S	S
15.0614148	Birmingham	UK	lineage4	S	S	S	S	S	S	U	S
16.0617384	Birmingham	UK	LAM	S	S	S	S	S	S	U	S
14.0600401	Birmingham	UK	EAI	S	S	S	S	S	S	S	S
15.0607151	Birmingham	UK	lineage4	S	S	S	S	S	S	S	S
14.0601000	Birmingham	UK	Cameroon	S	S	S	S	S	S	S	S
14.0600167	Birmingham	UK	EAI	S	S	S	S	S	S	S	S
16.0607357	Birmingham	UK	EAI	S	S	S	S	S	S	S	S
16.0600501	Birmingham	UK	lineage4	S	S	S	S	S	S	S	S
16.0601716	Birmingham	UK	LAM	S	S	S	S	S	S	S	S
13.0607866	Birmingham	UK	Delhi	S	S	S	S	S	S	S	S
10.0609409	Birmingham	UK	Delhi	S	S	S	S	S	S	U	S
12.0612519	Birmingham	UK	Delhi	R	S	S	S	R	S	U	S
16.0618433	Birmingham	UK	EAI	S	S	S	S	S	S	S	S
16.0618281	Birmingham	UK	EAI	S	S	S	S	S	S	S	S
15.0601101	Birmingham	UK	Beijing	R	R	R	R	R	R	R	R
12.0614150	Birmingham	UK	X-type	S	S	S	S	S	S	S	S
15.0613478	Birmingham	UK	Haarlem	S	S	S	S	S	S	S	S
14.0600585	Birmingham	UK	Cameroon	S	S	S	S	S	S	S	S
13.0609260	Birmingham	UK	Delhi	S	S	S	S	S	S	S	S
14.0613579	Birmingham	UK	LAM	S	S	S	S	S	S	S	S
13.0609437	Birmingham	UK	Delhi	S	S	S	S	S	S	S	S
13.0616622	Birmingham	UK	lineage4	S	S	S	S	S	S	S	S
13.0617139	Birmingham	UK	Delhi	S	S	S	S	S	R	S	S
14.0609470	Birmingham	UK	Delhi	S	S	S	S	S	S	S	S
14.0600468	Birmingham	UK	Delhi	S	S	S	S	S	S	S	S
13.0613203	Birmingham	UK	lineage4	S	S	S	S	S	S	S	S
12.0611024	Birmingham	UK	lineage4	S	S	S	S	S	S	S	S
13.0600579	Birmingham	UK	Delhi	S	S	S	S	F	S	S	S
12.0614993	Birmingham	UK	LAM	S	S	S	S	S	S	S	S
15.0601360	Birmingham	UK	Beijing	S	S	S	S	S	S	S	S
16.0606962	Birmingham	UK	lineage4	S	S	S	S	S	S	S	S
15.0608181	Birmingham	UK	Haarlem	S	S	S	S	S	S	U	S
16.0617145	Birmingham	UK	Cameroon	n/a	S	S	S	R	S	S	S
13.0609450	Birmingham	UK	Delhi	S	S	S	S	S	S	S	S
14.0613536	Birmingham	UK	Beijing	R	R	S	R	R	R	S	F
14.0609731	Birmingham	UK	lineage4	S	S	S	S	S	S	S	S
11.0600002	Birmingham	UK	Delhi	S	S	S	S	S	S	U	S
13.0605670	Birmingham	UK	Delhi	S	S	S	S	S	S	S	S
13.0605667	Birmingham	UK	Delhi	S	S	S	S	S	S	S	S
13.0604518	Birmingham	UK	S-type	S	S	S	S	S	U	S	S
16.0606291	Birmingham	UK	EAI	S	S	S	S	S	S	S	S
16.0611579	Birmingham	UK	lineage4	S	S	S	n/a	S	S	S	S
15.0613463	Birmingham	UK	EAI	S	S	S	n/a	S	S	S	S
14.0616088	Birmingham	UK	EAI	S	S	S	S	S	S	U	S
13.0608099	Birmingham	UK	Delhi	S	S	S	S	S	S	S	S
15.0607946	Birmingham	UK	X-type	S	S	S	S	S	S	S	S
14.0600481	Birmingham	UK	Delhi	S	S	S	S	S	S	S	S
14.0600972	Birmingham	UK	Delhi	R	R	R	R	R	R	R	R
15.0601750	Birmingham	UK	X-type	S	S	S	S	S	S	S	S
13.0610477	Birmingham	UK	EAI	S	S	S	S	S	S	S	S
16.0617041	Birmingham	UK	M. bovis	S	S	S	R	S	S	U	R
15.0607640	Birmingham	UK	Delhi	S	S	S	S	S	S	S	S
16.0601008	Birmingham	UK	Cameroon	S	S	S	S	S	S	S	S
16.0617611	Birmingham	UK	X-type	S	S	S	S	S	S	S	S
15.0613346	Birmingham	UK	EAI	S	S	S	n/a	S	S	S	S
14.0609622	Birmingham	UK	lineage4	S	S	S	S	S	S	S	S
15.0611900	Birmingham	UK	lineage4	S	S	S	S	S	S	S	S
16.0601923	Birmingham	UK	LAM	S	S	S	S	S	S	S	S
15.0607333	Birmingham	UK	Tur	S	S	S	S	S	S	S	S
09.0601167	Birmingham	UK	Beijing	S	S	S	S	S	S	S	F
16.0601373	Birmingham	UK	EAI	S	S	S	S	S	S	S	S
16.0614143	Birmingham	UK	Delhi	S	S	S	S	S	S	S	S
14.0611813	Birmingham	UK		S	S	S	S	S	S	S	S
14.0609424	Birmingham	UK	lineage4	S	S	S	S	S	S	S	S
13.0608332	Birmingham	UK	X-type	S	S	S	S	S	S	S	S
16.0618305	Birmingham	UK	Delhi	S	S	S	S	S	S	S	S
11.0611138	Birmingham	UK	Beijing	S	S	S	S	S	S	S	S
16.0618273	Birmingham	UK	lineage4	S	S	S	S	S	S	S	S
13.0609635	Birmingham	UK	Haarlem	S	S	S	S	R	S	S	S
13.0604524	Birmingham	UK	Delhi	S	S	S	S	S	S	S	S
15.0613347	Birmingham	UK	Haarlem	S	S	S	S	S	S	S	S
14.0600093	Birmingham	UK	Beijing	S	S	S	R	S	S	S	R

15.0607258	Birmingham	UK	X-type	S	S	S	S	S	S	S	S
12.0600591	Birmingham	UK	EAI	S	S	S	S	S	S	S	S
16.0601270	Birmingham	UK	Beijing	S	S	S	S	U	S	S	S
14.0609844	Birmingham	UK	Delhi	S	S	S	S	S	S	S	S
16.0601513	Birmingham	UK	EAI	S	S	S	n/a	S	S	S	S
15.0603311	Birmingham	UK	Haarlem	S	S	S	S	S	S	S	S
15.0604197	Birmingham	UK	X-type	S	S	S	S	S	S	S	S
14.0600496	Birmingham	UK	LAM	S	S	S	S	S	S	S	S
14.0612288	Birmingham	UK	LAM	R	S	S	S	R	S	S	S
16.0607093	Birmingham	UK	lineage4	S	S	S	S	S	S	S	S
15.0601260	Birmingham	UK	X-type	S	S	S	S	S	S	S	S
15.0607410	Birmingham	UK	X-type	S	S	S	S	S	F	S	S
13.0609886	Birmingham	UK	M. bovis	S	S	S	R	S	S	S	R
16.0618519	Birmingham	UK	Delhi	S	S	S	S	S	S	S	S
15.0601659	Birmingham	UK	Delhi	S	S	S	S	S	S	S	S
13.0606647	Birmingham	UK	LAM	S	S	S	S	S	S	S	S
13.0617512	Birmingham	UK	Beijing	S	S	S	S	S	S	U	S
14.0600048	Birmingham	UK	Beijing	S	S	S	S	S	R	S	S
14.0609769	Birmingham	UK	lineage4	S	S	S	S	S	S	S	S
11.0600416	Birmingham	UK	Delhi	S	S	S	S	S	S	S	S
15.0604807	Birmingham	UK	Delhi	S	S	S	S	S	S	S	S
13.0613161	Birmingham	UK	EAI	S	S	S	S	S	S	S	S
13.0613573	Birmingham	UK	lineage4	S	S	S	S	S	S	U	S
12.0616920	Birmingham	UK	Delhi	S	S	S	S	S	S	S	S
14.0609836	Birmingham	UK	Delhi	S	S	S	S	F	S	S	S
15.0601744	Birmingham	UK	Haarlem	S	S	S	S	S	S	S	S
16.0617571	Birmingham	UK	lineage4	S	S	S	n/a	S	S	S	S
14.0610487	Birmingham	UK	Delhi	S	S	S	S	S	S	S	S
16.0607148	Birmingham	UK	EAI	S	S	S	n/a	S	S	S	S
16.0607835	Birmingham	UK	Ural	S	S	S	S	F	F	F	F
12.0613050	Birmingham	UK	EAI	S	S	S	S	S	S	S	S
12.0605913	Birmingham	UK	Delhi	S	S	S	S	S	S	S	S
17.0601255	Birmingham	UK	Haarlem	S	S	S	S	S	S	S	S
15.0601858	Birmingham	UK	Delhi	S	S	S	S	S	S	U	S
16.0607688	Birmingham	UK	Delhi	S	S	S	S	S	S	S	S
15.0607278	Birmingham	UK	Tur	S	S	S	S	S	S	S	S
16.0601536	Birmingham	UK	Ural	S	S	S	S	S	S	S	S
12.0615525	Birmingham	UK	Delhi	S	S	S	S	S	S	S	S
14.0609190	Birmingham	UK	Beijing	S	S	S	S	S	S	S	S
14.0600255	Birmingham	UK	lineage4	S	S	S	S	S	S	S	S
14.0613142	Birmingham	UK	Cameroon	S	S	S	S	S	S	U	S
16.0601518	Birmingham	UK		R	S	S	S	F	U	U	S
12.0617768	Birmingham	UK	Delhi	n/a	n/a	n/a	S	S	S	U	S
12.0616137	Birmingham	UK	lineage4	S	S	S	S	S	S	S	S
15.0607765	Birmingham	UK	lineage4	S	S	S	S	S	S	S	S
16.0617206	Birmingham	UK	Ural	S	S	S	S	S	S	S	S
12.0612605	Birmingham	UK	Delhi	S	S	S	S	S	S	S	S
14.0600829	Birmingham	UK	EAI	S	S	S	S	S	S	S	S
13.0611006	Birmingham	UK	lineage4	S	S	S	S	S	S	F	S
09.0611913	Birmingham	UK	Delhi	S	S	S	S	S	S	S	S
14.0609337	Birmingham	UK	M. orygis	S	S	S	S	S	S	S	S
14.0616187	Birmingham	UK	Delhi	S	S	S	S	S	S	S	S
13.0604079	Birmingham	UK	Delhi	S	S	S	S	S	S	S	S
14.0609840	Birmingham	UK	EAI	S	S	S	S	S	S	S	S
13.0608446	Birmingham	UK	Ural	S	S	S	S	S	S	S	S
12.0612537	Birmingham	UK	Delhi	S	S	S	S	S	S	S	S
12.0617272	Birmingham	UK	LAM	S	S	S	S	S	S	S	S
15.0601911	Birmingham	UK	EAI	S	S	S	S	S	S	S	S
13.0607719	Birmingham	UK	Delhi	S	S	S	S	S	S	S	S
16.0601126	Birmingham	UK	Delhi	S	S	S	S	S	S	S	S
14.0609055	Birmingham	UK	Delhi	S	S	S	S	S	S	S	S
16.0611979	Birmingham	UK	Delhi	n/a	S	S	S	S	S	S	S
15.0607932	Birmingham	UK	Delhi	S	S	S	S	S	S	S	S
13.0613279	Birmingham	UK	Haarlem	S	S	S	S	S	S	S	S
12.0927688	Birmingham	UK	EAI	S	S	S	S	F	S	U	S
14.0600872	Birmingham	UK	EAI	S	S	S	S	S	S	S	S
13.0601979	Birmingham	UK	Delhi	S	S	S	S	S	F	S	F
14.0609714	Birmingham	UK	Delhi	R	S	S	S	R	S	U	S
14.0600732	Birmingham	UK	Delhi	S	S	S	S	S	S	S	S
16.0617394	Birmingham	UK	EAI	S	S	S	S	S	S	S	S
13.0612203	Birmingham	UK	lineage4	S	S	S	S	S	S	S	S
14.0600366	Birmingham	UK	EAI	S	S	S	S	S	S	S	S
12.0615205	Birmingham	UK	Delhi	S	S	S	S	S	S	U	S
14.0615435	Birmingham	UK	EAI	n/a	n/a	n/a	S	U	S	S	S
12.0614243	Birmingham	UK	Delhi	S	S	S	S	S	S	S	S
15.0601917	Birmingham	UK	EAI	S	S	S	S	S	S	S	S
15.0613414	Birmingham	UK	lineage4	S	S	S	S	S	S	S	S
14.0600013	Birmingham	UK	X-type	S	S	S	S	S	S	S	S
16.0606668	Birmingham	UK	Delhi	S	S	S	S	S	S	S	S
16.0618323	Birmingham	UK	Delhi	R	S	S	S	R	S	S	S
12.0614976	Birmingham	UK	Delhi	S	S	S	S	S	S	S	S
15.0607392	Birmingham	UK	Delhi	n/a	n/a	n/a	S	S	S	S	S
14.0600300	Birmingham	UK	lineage4	S	S	S	S	S	S	S	S
14.0614166	Birmingham	UK	Haarlem	S	S	S	S	S	S	S	S
12.0600662	Birmingham	UK	Delhi	S	S	S	S	S	S	S	S
16.0617087	Birmingham	UK	Ural	S	S	S	S	S	S	S	S
15.0601379	Birmingham	UK	Delhi	S	S	S	S	S	S	U	S
13.0605635	Birmingham	UK	LAM	S	S	S	S	S	S	S	S
15.0610969	Birmingham	UK	Delhi	S	S	S	S	S	S	S	S
16.0601185	Birmingham	UK	Delhi	S	S	S	S	S	F	S	S
13.0608432	Birmingham	UK	Delhi	S	S	S	S	S	S	S	S
15.0601090	Birmingham	UK	Delhi	R	S	S	S	R	R	R	R
14.0600978	Birmingham	UK	EAI	S	S	S	S	S	S	S	S
13.0606199	Birmingham	UK	Delhi	S	S	S	S	S	S	S	S
14.0609557	Birmingham	UK	Delhi	R	S	S	S	R	S	U	S
16.0617354	Birmingham	UK	Delhi	S	S	S	S	S	S	R	S
16.0601834	Birmingham	UK	M. orygis	n/a	n/a	n/a	S	S	S	S	S
15.0607357	Birmingham	UK	EAI	S	S	S	S	S	S	S	S
13.0609735	Birmingham	UK	Delhi	S	S	S	S	S	S	S	S
15.0601202	Birmingham	UK	Delhi	S	S	S	S	S	S	S	S
15.0612195	Birmingham	UK	Delhi	S	S	S	S	S	S	U	S
16.0607182	Birmingham	UK	Delhi	S	S	S	S	S	S	S	S
15.0601935	Birmingham	UK	Tur	n/a	S	S	S	R	S	S	S
14.0600573	Birmingham	UK	Delhi	S	S	S	S	S	S	U	S
13.0606682	Birmingham	UK	lineage4	S	S	S	S	S	S	S	S
16.0600998	Birmingham	UK	Cameroon	S	S	S	S	S	S	S	S
15.0613120	Birmingham	UK	Haarlem	S	S	S	S	S	S	S	S
14.0614276	Birmingham	UK	LAM	S	S	S	S	F	S	S	S
13.0604517	Birmingham	UK	Delhi	S	S	S	S	S	S	S	S
15.0605364	Birmingham	UK	Beijing	S	S	S	S	S	S	S	S
13.0608407	Birmingham	UK	X-type	S	S	S	S	S	S	S	S
13.0607819	Birmingham	UK	Haarlem	S	S	S	S	S	S	S	S
13.0604056	Birmingham	UK	Beijing	S	S	S	S	S	S	S	S
16.0601662	Birmingham	UK	Haarlem	S	S	S	S	S	S	S	S
15.0601569	Birmingham	UK	M. bovis	S	S	S	R	S	S	S	R
15.0601841	Birmingham	UK	Delhi	S	S	S	S	S	S	U	S

12.0614542	Birmingham	UK	M. bovis	S	S	S	R	S	S	S	R
13.0610345	Birmingham	UK	EAI	S	S	S	S	S	S	S	S
16.0604035	Birmingham	UK	Beijing	R	S	R	n/a	R	S	R	S
17.0601143	Birmingham	UK	LAM	S	S	S	S	F	S	F	S
15.0601722	Birmingham	UK	EAI	S	S	S	S	S	S	U	S
13.0601463	Birmingham	UK	Haarlem	S	S	S	S	S	S	S	S
14.0600042	Birmingham	UK	X-type	S	S	S	S	S	S	S	S
13.0613433	Birmingham	UK	LAM	S	S	S	S	S	S	S	S
16.0607659	Birmingham	UK	Haarlem	S	S	S	S	U	S	S	S
16.0607477	Birmingham	UK	EAI	S	S	S	S	S	S	S	S
16.0607466	Birmingham	UK	EAI	S	S	S	S	S	S	S	S
15.0601128	Birmingham	UK	EAI	S	S	S	S	S	F	S	S
17.0601016	Birmingham	UK	lineage4	S	S	S	S	S	S	S	S
09.0605581	Birmingham	UK	Beijing	n/a	S	S	S	R	R	S	S
13.0611889	Birmingham	UK	lineage4	S	S	S	S	S	S	S	S
14.0609802	Birmingham	UK	LAM	S	S	S	S	S	S	S	S
13.0606721	Birmingham	UK	Delhi	S	S	S	S	S	S	U	S
15.0613404	Birmingham	UK	LAM	R	S	S	S	R	S	S	S
16.0607978	Birmingham	UK	lineage4	S	S	S	S	S	S	U	S
15.0611236	Birmingham	UK	Delhi	S	S	S	S	S	S	S	S
14.0600488	Birmingham	UK	Delhi	S	S	S	S	S	S	S	S
12.0617718	Birmingham	UK	Delhi	S	S	S	S	S	S	S	S
16.0601369	Birmingham	UK	M. bovis	S	S	S	R	S	S	S	R
16.0602934	Birmingham	UK	Haarlem	S	S	S	S	S	S	S	S
15.0613244	Birmingham	UK	EAI	S	S	S	S	S	F	S	S
14.0609392	Birmingham	UK	M. orygis	S	S	S	S	S	S	U	S
14.0609659	Birmingham	UK	Delhi	S	S	S	S	S	S	S	S
13.0610904	Birmingham	UK		S	S	S	S	S	S	S	S
15.0601435	Birmingham	UK	lineage4	S	S	S	S	S	S	S	S
16.0618093	Birmingham	UK	Haarlem	S	S	S	S	S	S	S	S
13.0615441	Birmingham	UK	Cameroon	S	S	S	S	S	S	S	S
15.0601290	Birmingham	UK	Haarlem	S	S	S	S	S	S	S	S
15.0601635	Birmingham	UK	Haarlem	S	S	S	S	S	S	U	S
13.0605096	Birmingham	UK	Delhi	R	S	S	S	R	S	S	S
13.0607130	Birmingham	UK	lineage4	S	S	S	S	S	S	S	S
15.0601991	Birmingham	UK	Delhi	S	S	S	S	S	S	S	S
14.0609249	Birmingham	UK	Haarlem	R	S	S	S	R	S	S	S
16.0607015	Birmingham	UK	LAM	S	S	S	S	S	S	S	S
15.0609021	Birmingham	UK	Haarlem	S	S	S	S	S	S	S	S
16.0601689	Birmingham	UK	Ural	S	S	S	S	S	S	S	S
14.0609179	Birmingham	UK	Delhi	S	S	S	S	S	S	S	S
13.0611887	Birmingham	UK	Haarlem	S	S	S	S	S	S	S	S
15.0601031	Birmingham	UK	Delhi	S	S	S	S	S	S	S	S
12.0927476	Birmingham	UK	Delhi	n/a	n/a	n/a	S	R	S	S	S
16.0601900	Birmingham	UK	Delhi	S	S	S	S	S	S	S	S
12.0613180	Birmingham	UK	EAI	S	S	S	S	S	S	U	S
12.0616628	Birmingham	UK	Beijing	S	S	S	S	S	S	S	S
16.0618015	Birmingham	UK	lineage4	S	S	S	S	S	S	S	S
15.0612580	Birmingham	UK	Delhi	S	S	S	S	S	S	U	S
15.0605231	Birmingham	UK	M. bovis	R	n/a	n/a	R	S	S	U	R
13.0604061	Birmingham	UK	Delhi	S	S	S	S	S	S	S	S
15.0607729	Birmingham	UK	Beijing	R	R	R	R	R	R	R	R
16.0618613	Birmingham	UK	lineage4	n/a	n/a	n/a	S	S	S	U	S
14.0609823	Birmingham	UK	Cameroon	S	S	S	S	S	S	S	S
13.0613584	Birmingham	UK	X-type	S	S	S	S	S	S	S	S
13.0606631	Birmingham	UK	Haarlem	S	S	S	S	S	S	S	S
15.0607991	Birmingham	UK	X-type	S	S	S	S	S	S	S	S
13.0615550	Birmingham	UK	Delhi	S	S	S	S	S	S	S	S
14.0609900	Birmingham	UK	lineage4	S	S	S	S	S	S	S	S
16.0601458	Birmingham	UK	Haarlem	S	S	S	S	S	U	S	S
15.0607042	Birmingham	UK	lineage4	S	S	S	n/a	S	S	S	S
12.0617759	Birmingham	UK	Delhi	S	S	S	S	S	U	S	S
15.0601185	Birmingham	UK	LAM	S	S	S	S	S	S	S	S
12.0612685	Birmingham	UK	lineage4	S	S	S	S	S	S	S	S
16.0618435	Birmingham	UK	Haarlem	S	S	S	S	S	S	S	S
14.0600394	Birmingham	UK	Tur	S	S	S	S	S	S	S	S
14.0600302	Birmingham	UK	LAM	S	S	S	S	S	S	S	S
13.0613508	Birmingham	UK	Tur	S	S	S	S	S	S	S	S
13.0609805	Birmingham	UK	LAM	S	S	S	S	S	S	S	S
16.0601883	Birmingham	UK	Delhi	S	S	S	S	S	S	S	S
14.0609567	Birmingham	UK	Haarlem	S	S	S	S	S	S	S	S
14.0600726	Birmingham	UK	Delhi	S	S	S	S	S	S	S	S
15.0607229	Birmingham	UK	Delhi	S	S	S	S	S	S	S	S
16.0617175	Birmingham	UK	LAM	S	S	S	S	S	S	S	S
12.0614547	Birmingham	UK	lineage4	S	S	S	S	S	S	S	S
13.0607683	Birmingham	UK	lineage4	S	S	S	S	S	S	S	S
15.0608401	Birmingham	UK	lineage4	S	S	S	S	S	S	S	S
15.0607025	Birmingham	UK	EAI	S	S	S	S	S	S	S	S
13.0613379	Birmingham	UK	Ural	S	S	S	S	S	S	S	S
16.0608333	Birmingham	UK	Haarlem	S	S	S	S	S	S	S	S
13.0602832	Birmingham	UK	lineage4	S	S	S	S	S	S	S	S
14.0600001	Birmingham	UK	Delhi	S	S	S	S	S	S	S	S
12.0927272	Birmingham	UK	Delhi	R	S	S	S	R	S	S	S
15.0613024	Birmingham	UK	lineage4	S	S	S	S	S	S	S	S
16.0601456	Birmingham	UK	Delhi	S	S	S	S	S	S	S	S
13.0600601	Birmingham	UK	Delhi	S	S	S	S	F	S	S	S
13.0607127	Birmingham	UK	Delhi	S	S	S	S	S	S	S	S
15.0613356	Birmingham	UK	Delhi	S	S	S	S	S	F	S	S
12.0615704	Birmingham	UK	lineage4	S	S	S	S	F	S	S	S
15.0605833	Birmingham	UK	Delhi	S	S	S	S	S	S	S	S
16.0617279	Birmingham	UK	Haarlem	S	S	S	S	S	S	S	S
16.0607203	Birmingham	UK	Cameroon	S	S	S	S	S	S	S	S
13.0607135	Birmingham	UK	Tur	S	S	S	S	S	S	S	S
14.0609965	Birmingham	UK	Beijing	S	S	S	S	S	S	S	S
13.0612981	Birmingham	UK	EAI	S	S	S	S	S	S	U	S
13.0611027	Birmingham	UK	Delhi	S	S	S	S	S	S	S	S
15.0613219	Birmingham	UK	Delhi	S	S	S	S	S	U	S	S
12.0614218	Birmingham	UK	LAM	S	S	S	S	S	S	S	S
13.0609011	Birmingham	UK	Delhi	S	S	S	S	S	S	S	S
14.0600891	Birmingham	UK	Delhi	S	S	S	S	S	S	S	S
12.0612850	Birmingham	UK	Haarlem	S	S	S	S	S	S	S	S
16.0604876	Birmingham	UK	M. bovis	S	S	S	S	S	S	S	R
15.0613443	Birmingham	UK	Delhi	S	S	S	S	S	U	S	S
16.0607795	Birmingham	UK	Haarlem	S	S	S	S	S	S	S	S
16.0606663	Birmingham	UK	Delhi	S	S	S	n/a	S	S	S	S
14.0611503	Birmingham	UK	Haarlem	S	S	S	S	S	S	U	S
12.0605836	Birmingham	UK	Delhi	S	S	S	S	S	S	S	S
10.0604880	Birmingham	UK	EAI	n/a	n/a	n/a	S	U	S	S	S
15.0601847	Birmingham	UK	S-type	S	S	S	S	S	S	S	S
16.0601398	Birmingham	UK	Delhi	S	S	S	S	S	S	S	S
15.0607391	Birmingham	UK	Haarlem	S	S	S	S	S	S	S	S
13.0609905	Birmingham	UK	Delhi	S	S	S	S	S	S	S	S
12.0615281	Birmingham	UK	Delhi	S	S	S	S	S	S	S	S
15.0613151	Birmingham	UK	Delhi	S	S	S	S	S	F	F	S
16.0601239	Birmingham	UK	Delhi	S	S	S	S	S	S	S	S
15.0607063	Birmingham	UK	Delhi	S	S	S	S	S	S	S	S

14.0601755	Birmingham	UK	Beijing	R	R	S	S	R	R	S	F
15.0603336	Birmingham	UK	M. bovis	S	S	S	R	S	S	U	R
14.0600998	Birmingham	UK	Delhi	S	S	S	S	S	S	S	S
16.0601241	Birmingham	UK	EAI	S	S	S	n/a	S	S	U	S
12.0616138	Birmingham	UK	lineage4	S	S	S	S	S	S	U	S
16.0608062	Birmingham	UK	Delhi	S	S	S	S	S	S	S	S
09.0601060	Birmingham	UK	EAI	S	S	S	S	S	S	S	S
15.0607432	Birmingham	UK	lineage4	S	S	S	S	S	S	S	S
15.0601927	Birmingham	UK	Haarlem	S	S	S	S	S	S	S	S
16.0601035	Birmingham	UK	Delhi	S	S	S	n/a	S	S	S	R
16.0618488	Birmingham	UK	Beijing	R	R	R	S	R	R	R	S
15.0613062	Birmingham	UK	EAI	S	S	S	S	S	S	S	S
16.0600875	Birmingham	UK	Delhi	S	S	S	S	S	S	S	S
16.0607550	Birmingham	UK	EAI	S	S	S	S	S	S	S	S
13.0606482	Birmingham	UK	Delhi	S	S	S	S	F	F	S	S
14.0600780	Birmingham	UK	Beijing	S	S	S	S	F	S	S	S
13.0600595	Birmingham	UK	LAM	S	S	S	S	S	S	S	S
17.0601259	Birmingham	UK	EAI	S	S	S	n/a	S	F	S	S
14.0605843	Birmingham	UK	M. orygis	S	S	S	S	S	S	U	S
16.0618536	Birmingham	UK	Delhi	S	S	S	S	S	S	S	S
14.0609421	Birmingham	UK	EAI	S	S	S	S	S	S	S	S
15.0607437	Birmingham	UK	Delhi	S	S	S	S	S	S	S	S
13.0614172	Birmingham	UK	Haarlem	S	S	S	S	S	S	S	S
13.0607527	Birmingham	UK	Delhi	S	S	S	S	S	S	S	S
16.0617043	Birmingham	UK	X-type	S	S	S	S	S	S	S	S
16.0607506	Birmingham	UK	Delhi	S	S	S	S	S	S	S	S
15.0607343	Birmingham	UK	EAI	S	S	S	n/a	S	S	S	S
15.0605568	Birmingham	UK	Delhi	S	S	S	S	S	S	S	S
15.0600135	Birmingham	UK	Delhi	S	S	S	S	S	S	S	S
13.0601240	Birmingham	UK	X-type	S	S	S	S	S	S	S	S
13.0606209	Birmingham	UK	Delhi	S	S	S	S	S	S	U	S
14.0600326	Birmingham	UK	S-type	S	S	S	S	S	S	S	S
13.0613413	Birmingham	UK	Delhi	S	S	S	S	S	S	S	S
16.0601754	Birmingham	UK	M. bovis	S	S	S	R	S	S	U	R
16.0606189	Birmingham	UK	Delhi	S	S	S	S	S	S	S	S
14.0607127	Birmingham	UK		S	S	S	R	S	S	U	R
09.0609880	Birmingham	UK	Delhi	S	S	S	S	S	S	U	S
15.0601389	Birmingham	UK	Delhi	S	S	S	S	S	U	S	S
15.0609332	Birmingham	UK	EAI	S	S	S	S	S	S	S	S
13.0600321	Birmingham	UK	EAI	S	S	S	S	S	S	U	S
16.0617066	Birmingham	UK	lineage4	S	S	S	S	S	S	S	S
16.0607247	Birmingham	UK	lineage4	S	S	S	S	S	S	S	S
11.0604779	Birmingham	UK	M. bovis	S	S	S	R	S	S	U	R
15.0605033	Birmingham	UK	X-type	S	S	S	S	S	S	S	S
13.0604533	Birmingham	UK	EAI	S	S	S	S	S	S	S	S
15.0613287	Birmingham	UK	lineage4	S	S	S	S	S	S	U	S
16.0601799	Birmingham	UK	Haarlem	S	S	S	S	S	S	S	S
14.0614827	Birmingham	UK	lineage4	S	S	S	S	S	S	S	S
15.0613546	Birmingham	UK	Delhi	S	S	S	S	S	S	S	S
13.0609919	Birmingham	UK	X-type	S	S	S	S	S	S	S	S
16.0607711	Birmingham	UK	Uganda	S	S	S	S	S	S	S	S
13.0605097	Birmingham	UK	Delhi	S	S	S	S	S	S	S	S
14.0602632	Birmingham	UK	EAI	S	S	S	S	S	S	S	S
16.0618353	Birmingham	UK	lineage4	S	S	S	S	S	S	S	S
13.0606695	Birmingham	UK	lineage4	S	S	S	S	S	S	S	S
16.0607581	Birmingham	UK	EAI	S	S	S	n/a	S	F	S	S
14.0609341	Birmingham	UK	Delhi	S	S	S	S	S	S	S	S
15.0607742	Birmingham	UK	LAM	R	R	R	R	R	R	R	R
16.0607168	Birmingham	UK	Delhi	S	S	S	S	S	S	S	S
14.0600794	Birmingham	UK	Cameroon	S	S	S	S	S	S	S	S
14.0609024	Birmingham	UK	Delhi	S	S	S	S	S	U	S	S
15.0614334	Birmingham	UK	lineage4	S	S	S	S	S	S	S	S
12.0612563	Birmingham	UK	Beijing	R	R	R	R	R	R	R	R
12.0614972	Birmingham	UK	X-type	S	S	S	S	S	S	S	S
13.0611011	Birmingham	UK	lineage4	S	S	S	S	S	S	S	S
14.0609107	Birmingham	UK	Cameroon	S	S	S	S	S	S	S	S
14.0616102	Birmingham	UK	lineage4	S	S	S	S	S	S	S	S
15.0608983	Birmingham	UK	LAM	S	S	S	S	S	S	S	S
15.0613288	Birmingham	UK	M. orygis	S	S	S	S	S	S	S	S
12.0614235	Birmingham	UK	EAI	S	S	S	S	S	S	S	S
14.0609921	Birmingham	UK	Delhi	S	S	S	S	S	F	S	S
16.0618345	Birmingham	UK	lineage4	S	S	S	S	U	S	U	S
12.0605242	Birmingham	UK	lineage4	S	S	S	S	S	S	S	S
15.0607419	Birmingham	UK	Beijing	S	S	S	S	S	S	S	S
15.0610340	Birmingham	UK	EAI	S	S	S	S	F	F	F	S
14.0600643	Birmingham	UK	LAM	S	S	S	S	S	S	S	S
16.0601632	Birmingham	UK	Delhi	S	S	S	S	S	S	S	S
16.0603586	Birmingham	UK	EAI	S	S	S	S	S	S	S	S
13.0607995	Birmingham	UK	EAI	S	S	S	S	S	S	S	S
15.0601848	Birmingham	UK	Delhi	S	S	S	S	S	S	S	S
14.0614925	Birmingham	UK	Delhi	R	S	S	S	R	S	U	S
16.0609912	Birmingham	UK	Delhi	S	S	S	S	S	S	S	S
13.0611915	Birmingham	UK	Delhi	S	S	S	n/a	S	S	S	S
14.0600424	Birmingham	UK	LAM	S	S	S	S	S	S	S	S
13.0608412	Birmingham	UK	Beijing	S	S	S	S	S	S	S	S
16.0601435	Birmingham	UK	lineage4	S	S	S	S	S	S	S	S
13.0611680	Birmingham	UK	EAI	S	S	S	S	S	F	S	S
15.0613100	Birmingham	UK	Delhi	S	S	S	S	S	S	S	S
13.0607675	Birmingham	UK	Haarlem	S	S	S	S	S	S	S	S
14.0609715	Birmingham	UK	Haarlem	R	S	R	S	R	S	R	S
16.0601602	Birmingham	UK		S	S	S	S	S	S	S	S
15.0607684	Birmingham	UK	LAM	S	S	S	S	S	S	S	S
09.0603362	Birmingham	UK	lineage4	S	S	S	S	S	S	S	S
13.0610344	Birmingham	UK	LAM	S	S	S	S	S	S	S	S
16.0617176	Birmingham	UK	LAM	S	S	S	S	S	S	U	S
15.0602862	Birmingham	UK	Cameroon	S	S	S	S	S	S	S	S
16.0618607	Birmingham	UK	Ural	S	S	S	S	S	S	S	S
14.0616094	Birmingham	UK	EAI	S	S	S	S	S	S	S	S
15.0606896	Birmingham	UK	Delhi	S	S	S	S	S	S	S	S
12.0615308	Birmingham	UK	lineage4	S	S	S	S	S	S	S	S
17.0601092	Birmingham	UK	Delhi	n/a	S	S	S	R	S	S	S
14.0600038	Birmingham	UK	Beijing	S	S	S	S	S	S	S	S
16.0618512	Birmingham	UK	Delhi	n/a	n/a	n/a	S	S	S	U	S
13.0616797	Birmingham	UK	lineage4	S	S	S	S	S	S	S	S
14.0616078	Birmingham	UK	S-type	S	S	S	S	S	S	S	S
15.0607448	Birmingham	UK	Delhi	S	S	S	S	S	S	S	S
12.0613184	Birmingham	UK		S	S	S	S	S	S	S	S
17.0601139	Birmingham	UK	LAM	S	S	S	S	S	S	U	S
16.0607564	Birmingham	UK	lineage4	S	S	S	S	S	S	F	S
14.0600940	Birmingham	UK	Beijing	S	S	S	S	S	S	U	S
16.0618214	Birmingham	UK	Delhi	S	S	S	S	S	S	S	S
14.0600700	Birmingham	UK	Haarlem	S	S	S	S	S	S	S	S
16.0618552	Birmingham	UK	Haarlem	S	S	S	S	S	S	S	S
12.0615200	Birmingham	UK	EAI	R	S	S	S	R	S	S	S
15.0607403	Birmingham	UK	X-type	S	S	S	S	S	S	S	S
12.0610248	Birmingham	UK	Delhi	S	S	S	S	S	S	S	S

15.0613061	Birmingham	UK	lineage4	S	S	S	S	S	S	S	S
14.0607878	Birmingham	UK		S	S	S	S	U	S	S	S
15.0613262	Birmingham	UK	Haarlem	S	S	S	S	S	S	S	S
13.0605136	Birmingham	UK	Delhi	S	S	S	S	S	S	S	S
16.0607383	Birmingham	UK	Haarlem	S	S	S	S	S	S	S	S
16.0601357	Birmingham	UK	Delhi	S	S	S	S	S	S	S	S
16.0601835	Birmingham	UK	X-type	S	S	S	n/a	S	S	S	S
15.0608108	Birmingham	UK	lineage4	S	S	S	S	S	S	S	S
14.0610939	Birmingham	UK	EAI	R	R	S	S	R	R	U	S
13.0612201	Birmingham	UK	Delhi	S	S	S	S	S	S	S	S
15.0601798	Birmingham	UK	Delhi	S	S	S	S	S	S	S	S
13.0613448	Birmingham	UK	LAM	S	S	S	S	S	U	S	S
14.0600784	Birmingham	UK	EAI	S	S	S	S	S	S	S	S
11.0601403	Birmingham	UK	Delhi	S	S	S	S	S	S	U	S
15.0601663	Birmingham	UK	EAI	S	S	S	S	U	S	U	S
13.0613500	Birmingham	UK	lineage4	S	S	S	S	S	S	S	S
12.0610997	Birmingham	UK	M. bovis	S	S	S	R	S	S	S	R
12.0615282	Birmingham	UK	Delhi	S	S	S	S	S	S	S	S
16.0601023	Birmingham	UK	Delhi	S	S	S	S	S	S	S	S
15.0604991	Birmingham	UK	Delhi	S	S	S	S	S	S	U	S
15.0607701	Birmingham	UK	Haarlem	S	S	S	S	S	S	S	S
14.0609518	Birmingham	UK	Haarlem	S	S	S	S	S	S	S	S
15.0607047	Birmingham	UK	Haarlem	S	S	S	S	S	S	S	S
15.0607399	Birmingham	UK	lineage4	S	S	S	S	U	S	S	S
14.0609895	Birmingham	UK	X-type	S	S	S	S	S	S	S	S
13.0613617	Birmingham	UK	EAI	S	S	S	S	S	S	S	R
14.0600863	Birmingham	UK	Ural	S	S	S	S	S	S	S	S
14.0600958	Birmingham	UK	Haarlem	S	S	S	S	S	S	S	S
14.0616200	Birmingham	UK	Haarlem	S	S	S	S	S	S	S	S
15.0607770	Birmingham	UK	Haarlem	S	S	S	S	S	S	S	S
15.0611450	Birmingham	UK	Beijing	R	R	S	n/a	R	R	S	R
16.0617638	Birmingham	UK	Delhi	n/a	n/a	n/a	S	S	S	U	S
16.0606653	Birmingham	UK	LAM	S	S	S	S	S	S	S	S
16.0607556	Birmingham	UK		S	S	S	S	U	S	S	S
13.0613250	Birmingham	UK	Delhi	S	S	S	S	S	R	S	S
14.0609920	Birmingham	UK	EAI	S	S	S	S	S	S	S	S
16.0607059	Birmingham	UK	Delhi	S	S	S	S	S	S	S	S
13.0609241	Birmingham	UK	Delhi	S	S	S	S	S	U	S	S
15.0607732	Birmingham	UK	Haarlem	S	S	S	S	S	S	S	S
14.0612094	Birmingham	UK	Tur	S	S	S	S	S	S	S	S
16.0607396	Birmingham	UK	LAM	S	S	S	S	S	S	S	S
15.0605286	Birmingham	UK	M. orygis	S	S	S	S	S	S	S	S
14.0609058	Birmingham	UK	Delhi	S	S	S	S	S	S	S	S
16.0607988	Birmingham	UK	Tur	S	S	S	S	F	S	S	S
14.0600208	Birmingham	UK	Delhi	S	S	S	S	S	S	S	S
15.0607595	Birmingham	UK	EAI	S	S	S	n/a	S	S	U	S
15.0601631	Birmingham	UK	Haarlem	R	S	R	S	R	S	R	S
15.0608954	Birmingham	UK	LAM	S	S	S	S	S	S	S	S
12.0613635	Birmingham	UK	EAI	S	S	S	S	S	S	S	S
16.0600539	Birmingham	UK	LAM	S	S	S	S	S	S	S	S
12.0611832	Birmingham	UK	Delhi	S	S	S	S	S	S	S	S
15.0601272	Birmingham	UK	lineage4	S	S	S	S	S	S	S	S
14.0600516	Birmingham	UK	lineage4	S	S	S	S	S	S	S	S
16.0607920	Birmingham	UK	EAI	S	S	S	S	S	S	S	S
14.0600730	Birmingham	UK	Delhi	S	S	S	S	S	S	S	S
16.0607963	Birmingham	UK	Beijing	S	S	S	S	S	S	S	S
15.0600750	Birmingham	UK	Delhi	S	S	S	S	S	S	S	S
16.0601347	Birmingham	UK	Delhi	S	S	S	S	S	S	S	S
15.0613370	Birmingham	UK	EAI	S	S	S	S	S	S	S	S
15.0601385	Birmingham	UK	Delhi	S	S	S	S	S	S	S	S
13.0612831	Birmingham	UK	Haarlem	S	S	S	S	S	S	U	S
15.0601879	Birmingham	UK	lineage4	S	S	S	S	S	S	S	S
16.0617388	Birmingham	UK	lineage4	n/a	n/a	n/a	S	S	S	S	S
13.0609442	Birmingham	UK	Delhi	S	S	S	S	S	S	S	S
15.0614933	Birmingham	UK	Beijing	R	R	R	R	R	R	R	R
13.0613548	Birmingham	UK	Haarlem	S	S	S	S	S	S	S	S
16.0601771	Birmingham	UK	EAI	S	S	S	S	S	S	U	S
16.0617076	Birmingham	UK	EAI	S	S	S	n/a	S	S	S	S
14.0607716	Birmingham	UK	Beijing	S	S	S	S	S	S	S	S
13.0612770	Birmingham	UK	Delhi	S	S	S	S	S	S	S	S
12.0610525	Birmingham	UK	Delhi	S	S	S	S	S	S	S	S
15.0611714	Birmingham	UK	Beijing	S	R	S	S	S	R	S	S
14.0609584	Birmingham	UK	Delhi	S	S	S	S	S	S	S	S
14.0609906	Birmingham	UK	lineage4	S	S	S	S	S	S	S	S
12.0611219	Birmingham	UK	Delhi	S	S	S	S	S	S	S	S
16.0618511	Birmingham	UK	Haarlem	S	S	S	S	S	S	S	S
09.0600641	Birmingham	UK	X-type	S	S	S	S	S	S	S	S
14.0605845	Birmingham	UK	Haarlem	S	S	S	S	S	S	S	S
13.0610351	Birmingham	UK	lineage4	S	S	S	S	S	S	U	S
15.0605427	Birmingham	UK	Delhi	S	S	S	S	S	S	S	S
15.0601191	Birmingham	UK	Haarlem	S	S	S	S	S	F	S	S
15.0607867	Birmingham	UK	EAI	S	S	S	S	S	S	S	S
12.0613148	Birmingham	UK	Beijing	S	S	S	S	S	S	U	S
15.0601216	Birmingham	UK	Haarlem	S	S	S	S	S	S	S	S
12.0614635	Birmingham	UK	EAI	R	S	S	S	R	S	S	S
14.0600641	Birmingham	UK	Delhi	S	S	S	S	S	S	S	S
14.0610228	Birmingham	UK		S	S	S	S	S	S	S	S
16.0607360	Birmingham	UK	EAI	S	S	S	S	S	S	S	S
16.0606030	Birmingham	UK	Delhi	S	S	S	S	S	S	S	S
14.0609537	Birmingham	UK	LAM	S	S	S	S	S	S	S	S
15.0601810	Birmingham	UK	Beijing	R	R	R	n/a	R	R	R	R
14.0609517	Birmingham	UK	lineage4	S	S	S	S	S	S	S	S
15.0613507	Birmingham	UK	Haarlem	S	S	S	S	S	S	S	S
16.0601905	Birmingham	UK	EAI	S	S	S	S	S	F	S	S
15.0607906	Birmingham	UK	Cameroon	S	S	S	S	S	U	U	S
14.0600043	Birmingham	UK	EAI	S	S	S	S	U	S	S	S
13.0607955	Birmingham	UK	Beijing	S	S	S	S	S	S	S	S
13.0613456	Birmingham	UK	LAM	S	S	S	S	S	S	S	S
12.0609859	Birmingham	UK	lineage4	S	S	S	S	S	S	S	S
16.0601442	Birmingham	UK	Ural	S	S	S	S	S	S	S	S
12.0616367	Birmingham	UK	Delhi	S	S	S	S	S	S	S	S
16.0617475	Birmingham	UK	X-type	S	S	S	S	S	S	S	S
13.0611346	Birmingham	UK	Delhi	R	S	S	S	R	S	S	S
14.0601099	Birmingham	UK	Beijing	S	S	S	S	S	S	U	S
16.0618377	Birmingham	UK	Delhi	S	S	S	S	F	S	F	S
12.0612882	Birmingham	UK	LAM	S	S	S	S	S	S	S	S
13.0608593	Birmingham	UK	X-type	S	S	S	S	S	S	S	S
14.0610966	Birmingham	UK		R	R	S	n/a	R	R	S	S
15.0607195	Birmingham	UK	S-type	S	S	S	S	S	S	S	S
13.0613072	Birmingham	UK	X-type	S	S	S	S	S	S	S	S
15.0607584	Birmingham	UK	lineage4	R	S	S	S	R	S	S	S
16.0601610	Birmingham	UK		S	S	S	S	S	S	U	S
16.0614730	Birmingham	UK	Tur	S	S	S	S	S	S	S	S
15.0601396	Birmingham	UK	S-type	S	S	S	S	S	S	S	S
16.0618456	Birmingham	UK	Delhi	S	S	S	S	S	F	S	S
14.0600201	Birmingham	UK	Delhi	S	S	S	S	S	S	S	S

13.0608441	Birmingham	UK	Haarlem	S	S	S	S	S	S	S	S
15.0613509	Birmingham	UK	Cameroon	S	S	S	S	S	S	S	S
12.0927853	Birmingham	UK	Delhi	S	S	S	S	S	S	S	S
11.0600317	Birmingham	UK	EAI	R	S	S	S	R	S	S	S
15.0607944	Birmingham	UK	Delhi	S	S	S	S	S	S	S	F
13.0614070	Birmingham	UK	lineage4	S	S	S	S	S	S	S	S
13.0609444	Birmingham	UK	LAM	S	S	S	S	S	S	S	S
14.0609585	Birmingham	UK	Beijing	S	S	S	S	S	S	S	S
16.0618457	Birmingham	UK	Delhi	S	S	S	S	S	S	U	S
14.0600405	Birmingham	UK	Delhi	S	S	S	S	S	S	S	S
13.0613315	Birmingham	UK	Delhi	S	S	S	S	S	S	U	S
13.0603562	Birmingham	UK	Beijing	S	S	S	S	S	S	S	S
16.0601829	Birmingham	UK	Delhi	S	S	S	S	F	U	F	S
13.0613248	Birmingham	UK	EAI	R	S	S	S	R	S	S	S
13.0617351	Birmingham	UK	Cameroon	S	S	S	S	S	S	U	S
17.0601074	Birmingham	UK	EAI	S	S	S	S	S	S	U	S
16.0607541	Birmingham	UK	Beijing	S	S	S	S	S	S	S	S
15.0606851	Birmingham	UK	LAM	R	R	S	R	R	R	R	R
14.0600335	Birmingham	UK	Delhi	S	S	S	S	S	S	S	S
14.0600293	Birmingham	UK	X-type	S	S	S	S	S	S	S	S
13.0615771	Birmingham	UK	Delhi	S	S	S	S	S	R	S	S
16.0607628	Birmingham	UK	Beijing	S	S	S	S	S	S	S	S
14.0600765	Birmingham	UK	EAI	S	S	S	S	S	S	S	R
15.0607485	Birmingham	UK	Haarlem	S	S	S	S	S	S	S	S
13.0612965	Birmingham	UK	Delhi	S	S	S	S	S	S	S	S
15.0607870	Birmingham	UK	Delhi	S	S	S	S	S	S	S	S
13.0613128	Birmingham	UK	EAI	S	S	S	S	S	S	S	R
11.0606544	Birmingham	UK	Delhi	S	S	S	S	S	S	U	S
14.0600886	Birmingham	UK	Delhi	S	S	S	S	S	S	S	S
16.0604402	Birmingham	UK	Delhi	S	S	S	S	S	S	F	S
15.0603499	Birmingham	UK	M. bovis	S	S	S	R	S	S	S	R
13.0613608	Birmingham	UK	lineage4	S	S	S	S	S	S	S	S
16.0618309	Birmingham	UK	LAM	S	S	S	S	S	S	S	S
14.0600095	Birmingham	UK	Delhi	S	S	S	S	S	S	S	S
14.0600131	Birmingham	UK	Cameroon	S	S	S	S	U	S	S	S
15.0612665	Birmingham	UK	Delhi	S	S	S	S	S	S	S	S
14.0613807	Birmingham	UK	Delhi	S	S	S	S	S	S	S	S
16.0618134	Birmingham	UK	EAI	S	S	S	S	S	S	S	S
15.0607688	Birmingham	UK	Delhi	S	S	S	S	S	S	S	S
14.0608882	Birmingham	UK		R	S	S	S	R	S	S	S
16.0601781	Birmingham	UK	lineage4	S	S	S	S	U	S	S	S
14.0600584	Birmingham	UK	Delhi	S	S	S	S	S	S	S	S
14.0609336	Birmingham	UK	LAM	S	S	S	S	S	S	S	S
16.0617289	Birmingham	UK	Beijing	S	S	S	S	S	S	S	S
14.0600085	Birmingham	UK	lineage4	S	S	S	S	S	S	S	S
15.0607720	Birmingham	UK	EAI	S	S	S	S	S	S	U	S
13.0601982	Birmingham	UK	LAM	S	S	S	S	S	S	S	S
16.0601425	Birmingham	UK	Tur	R	R	S	S	R	R	S	S
15.0601414	Birmingham	UK		S	S	S	S	S	S	S	S
13.0601208	Birmingham	UK	Ural	R	S	S	S	R	S	S	S
09.0606195	Birmingham	UK	Beijing	S	S	S	S	S	S	S	S
16.0603595	Birmingham	UK	lineage4	S	S	S	S	S	S	S	S
13.0611350	Birmingham	UK	Delhi	S	S	S	S	S	S	S	S
13.0608436	Birmingham	UK	Beijing	S	S	S	S	S	S	S	S
12.0614208	Birmingham	UK	EAI	S	S	S	S	S	S	S	S
16.0607164	Birmingham	UK	Delhi	S	S	S	S	S	S	U	S
16.0617708	Birmingham	UK	Delhi	R	S	n/a	n/a	R	R	R	R
12.0611202	Birmingham	UK	Beijing	S	S	S	S	S	S	S	S
14.0600006	Birmingham	UK	LAM	S	S	S	S	S	S	S	S
14.0615554	Birmingham	UK	Delhi	S	S	S	n/a	S	S	S	S
11.0605136	Birmingham	UK	Delhi	S	S	S	S	S	S	S	S
16.0618378	Birmingham	UK	Beijing	R	R	S	S	R	R	R	S
12.0616387	Birmingham	UK	X-type	S	S	S	S	F	U	S	S
14.0609724	Birmingham	UK	lineage4	S	S	S	S	S	S	S	S
16.0617972	Birmingham	UK	Ural	S	S	S	S	S	S	S	S
15.0601022	Birmingham	UK	Delhi	S	S	S	S	S	S	S	S
14.0609784	Birmingham	UK	LAM	S	S	S	S	S	S	S	S
14.0609214	Birmingham	UK	Delhi	S	S	S	S	S	S	S	S
16.0601633	Birmingham	UK	Delhi	S	S	S	S	S	S	S	S
13.0602580	Birmingham	UK	Haarlem	S	S	S	S	F	S	S	S
13.0613581	Birmingham	UK	Beijing	S	S	S	S	S	S	U	S
15.0606076	Birmingham	UK	lineage4	S	S	S	S	S	S	S	S
13.0603691	Birmingham	UK	lineage4	S	S	S	S	S	S	S	S
14.0609684	Birmingham	UK	EAI	S	S	S	S	S	U	S	S
16.0610284	Birmingham	UK	LAM	S	S	S	S	S	S	S	S
13.0613327	Birmingham	UK	EAI	S	S	S	S	U	S	S	S
15.0607128	Birmingham	UK	Delhi	R	R	S	R	R	R	R	R
16.0618491	Birmingham	UK	Cameroon	n/a	n/a	n/a	S	S	S	S	S
15.0600191	Birmingham	UK	Delhi	S	S	S	S	S	S	S	S
16.0618087	Birmingham	UK	lineage4	S	S	S	n/a	S	S	S	S
14.0609977	Birmingham	UK	Delhi	S	S	S	S	S	S	S	S
16.0601728	Birmingham	UK	Delhi	S	S	S	S	S	S	S	S
14.0609611	Birmingham	UK	Beijing	S	S	S	S	S	S	U	S
14.0607443	Birmingham	UK	X-type	S	S	S	S	S	S	S	S
12.0607473	Birmingham	UK	Haarlem	S	S	S	S	S	S	U	S
15.0613340	Birmingham	UK	lineage4	S	S	S	S	S	S	S	S
16.0617269	Birmingham	UK	Haarlem	S	S	S	S	S	S	S	S
14.0600975	Birmingham	UK	lineage4	S	S	S	S	S	S	S	S
13.0601235	Birmingham	UK	Beijing	S	S	S	S	S	S	S	S
13.0609497	Birmingham	UK	Delhi	S	S	S	S	S	S	S	S
14.0600558	Birmingham	UK	Delhi	S	S	S	S	S	S	S	S
15.0607065	Birmingham	UK	lineage4	S	S	S	S	S	S	S	S
14.0602472	Birmingham	UK	lineage4	S	S	S	S	S	S	S	S
16.0601500	Birmingham	UK	lineage4	S	S	S	S	U	S	S	S
15.0613321	Birmingham	UK	Delhi	S	S	S	S	S	U	S	S
14.0616157	Birmingham	UK	M. bovis	S	S	S	R	S	S	S	R
14.0613908	Birmingham	UK	M. orygis	S	S	S	S	U	S	S	S
14.0600661	Birmingham	UK	Delhi	S	S	S	S	S	S	S	S
14.0600898	Birmingham	UK	EAI	S	S	S	S	S	S	S	S
14.0600793	Birmingham	UK	Delhi	S	S	S	S	S	S	S	S
13.0612774	Birmingham	UK	Beijing	S	S	S	S	S	S	S	S
15.0601612	Birmingham	UK	Beijing	S	S	S	S	S	S	S	S
14.0607149	Birmingham	UK	Delhi	S	S	S	S	S	S	S	S
13.0600577	Birmingham	UK	Haarlem	S	S	S	S	S	S	S	S
14.0600353	Birmingham	UK	EAI	S	S	S	S	S	U	U	S
15.0607309	Birmingham	UK	Ghana	S	S	S	S	S	S	S	S
16.0617065	Birmingham	UK	EAI	S	S	S	S	S	S	S	S
14.0600525	Birmingham	UK	Cameroon	S	S	S	S	S	S	S	S
12.0602488	Birmingham	UK	Delhi	S	S	S	S	S	S	S	S
14.0600648	Birmingham	UK	Delhi	S	S	S	S	S	S	U	S
13.0608402	Birmingham	UK	Delhi	S	S	S	S	S	S	S	S
15.0607697	Birmingham	UK	Haarlem	S	S	S	S	S	S	S	S
13.0604510	Birmingham	UK	lineage4	S	S	S	S	S	S	S	S
16.0610249	Birmingham	UK		S	S	S	S	S	S	S	S
12.0617795	Birmingham	UK	lineage4	S	S	S	S	S	S	S	S
12.0611895	Birmingham	UK	Delhi	S	S	S	S	S	S	S	S

15.0607777	Birmingham	UK	Delhi	S	S	S	n/a	S	S	S	R
15.0613131	Birmingham	UK	West Africar	n/a	n/a	n/a	S	S	F	U	S
13.0604881	Birmingham	UK	X-type	S	S	S	S	S	S	S	S
12.0613618	Birmingham	UK	LAM	S	S	S	S	S	S	S	S
13.0610340	Birmingham	UK	Ural	R	R	S	R	R	R	R	U
14.0613032	Birmingham	UK	Delhi	S	S	S	S	S	S	S	S
14.0603310	Birmingham	UK	Beijing	R	R	S	S	R	R	S	F
16.0618304	Birmingham	UK	lineage4	S	S	S	S	F	S	S	F
14.0600487	Birmingham	UK	Beijing	R	S	S	S	R	S	S	S
13.0613246	Birmingham	UK	Delhi	R	S	S	S	R	S	S	S
13.0603119	Birmingham	UK	lineage4	S	S	S	S	S	S	S	S
15.0607738	Birmingham	UK	lineage4	R	R	S	R	R	R	R	R
13.0612469	Birmingham	UK	EAI	S	S	S	S	S	S	S	S
16.0610525	Birmingham	UK	Haarlem	S	S	S	S	S	S	S	S
13.0605676	Birmingham	UK	Delhi	R	S	S	S	R	S	S	S
13.0604904	Birmingham	UK	EAI	S	S	S	S	U	S	S	S
15.0607202	Birmingham	UK	EAI	S	S	S	S	S	S	S	S
13.0613129	Birmingham	UK	Delhi	S	S	S	S	S	S	S	S
09.0601854	Birmingham	UK	LAM	S	S	S	S	S	S	S	S
16.0607678	Birmingham	UK		R	R	R	n/a	R	F	R	F
13.0600560	Birmingham	UK	Beijing	S	S	S	S	S	S	S	S
16.0618443	Birmingham	UK	LAM	S	S	S	S	S	S	S	S
12.0613640	Birmingham	UK	Cameroon	R	S	S	S	R	S	S	S
16.0610390	Birmingham	UK	Delhi	S	S	S	S	S	S	S	S
13.0601572	Birmingham	UK	Delhi	S	S	S	S	U	S	S	S
13.0610753	Birmingham	UK	EAI	S	S	S	S	S	S	S	S
13.0610487	Birmingham	UK	lineage4	S	S	S	S	S	S	S	S
16.0617134	Birmingham	UK	Delhi	S	S	S	S	S	S	S	S
14.0609177	Birmingham	UK	LAM	S	S	S	S	S	S	S	S
13.0610505	Birmingham	UK	Delhi	S	S	S	S	S	S	S	S
16.0601832	Birmingham	UK	Beijing	R	R	R	S	R	R	R	S
13.0603114	Birmingham	UK	Beijing	R	R	S	R	R	R	R	R
15.0601637	Birmingham	UK	Delhi	S	S	S	S	S	S	S	S
15.0601497	Birmingham	UK	Delhi	S	S	S	S	S	S	S	S
14.0600065	Birmingham	UK	lineage4	S	S	S	S	S	S	S	S
15.0601709	Birmingham	UK	EAI	S	S	S	S	S	S	S	S
15.0601395	Birmingham	UK	Delhi	S	S	S	S	S	S	S	S
13.0613536	Birmingham	UK	Haarlem	S	S	S	S	S	S	S	S
14.0607822	Birmingham	UK	lineage4	S	S	S	S	S	S	S	S
16.0607887	Birmingham	UK	Delhi	S	S	S	S	S	S	S	S
15.0601891	Birmingham	UK	Delhi	S	S	S	S	S	S	S	S
15.0601660	Birmingham	UK	Delhi	S	S	S	S	S	S	S	S
14.0609767	Birmingham	UK	lineage4	S	S	S	S	S	S	S	S
15.0607990	Birmingham	UK	LAM	S	S	S	S	S	S	S	S
16.0613240	Birmingham	UK	Delhi	S	S	S	S	S	S	S	S
15.0613136	Birmingham	UK	lineage4	S	S	S	S	S	S	S	S
13.0614444	Birmingham	UK	Tur	S	S	S	S	S	S	S	S
14.0604921	Birmingham	UK	X-type	S	S	S	S	S	S	S	S
15.0601684	Birmingham	UK	Delhi	S	S	S	S	S	S	S	S
16.0618470	Birmingham	UK	Cameroon	S	S	S	S	U	S	S	S
16.0601201	Birmingham	UK	Tur	S	S	S	S	S	S	S	S
11.0606202	Birmingham	UK	Beijing	S	S	S	S	S	S	S	S
16.0607953	Birmingham	UK	lineage4	S	S	S	S	S	S	S	S
16.0612693	Birmingham	UK	Delhi	S	S	S	S	S	S	S	S
15.0609392	Birmingham	UK	EAI	S	S	S	S	S	S	S	S
13.0608466	Birmingham	UK	Delhi	S	S	S	S	U	S	U	S
10.0605284	Birmingham	UK	Beijing	S	S	S	S	S	S	S	S
15.0607787	Birmingham	UK	Delhi	S	S	S	S	S	U	S	S
15.0607086	Birmingham	UK	lineage4	n/a	S	S	S	R	S	S	S
14.0609503	Birmingham	UK	X-type	S	S	S	S	S	S	S	S
16.0617460	Birmingham	UK	Haarlem	S	S	S	n/a	S	S	S	S
13.0613215	Birmingham	UK	Haarlem	S	S	S	S	S	S	S	S
16.0618555	Birmingham	UK	LAM	S	S	S	S	S	S	S	S
13.0607381	Birmingham	UK	Delhi	S	S	S	S	S	S	S	S
15.0607180	Birmingham	UK	Delhi	S	S	S	S	S	S	S	S
15.0607020	Birmingham	UK	X-type	S	S	S	S	S	S	S	S
14.0602107	Birmingham	UK	Delhi	S	S	S	S	U	S	S	S
13.0613392	Birmingham	UK	lineage4	R	S	S	S	R	S	S	S
13.0613485	Birmingham	UK	X-type	S	S	S	S	S	S	S	S
15.0606354	Birmingham	UK	Delhi	S	S	S	S	S	S	S	S
10.0612331	Birmingham	UK	Delhi	S	S	S	S	S	S	S	S
16.0601962	Birmingham	UK	Haarlem	S	S	S	S	R	S	S	F
13.0607380	Birmingham	UK	lineage4	R	R	S	n/a	R	R	R	U
11.0600990	Birmingham	UK	lineage4	S	S	S	S	U	S	S	S
16.0605670	Birmingham	UK	Haarlem	S	S	S	S	S	S	S	S
13.0602845	Birmingham	UK	Cameroon	S	S	S	S	S	S	S	S
16.0618605	Birmingham	UK	LAM	S	S	S	S	S	S	S	S
13.0604532	Birmingham	UK	Delhi	S	S	S	S	S	S	S	S
13.0611318	Birmingham	UK	lineage4	S	S	S	S	S	S	S	S
13.0613052	Birmingham	UK	lineage4	S	S	S	S	S	S	S	S
15.0601748	Birmingham	UK	Delhi	S	S	S	S	S	S	S	S
14.0600942	Birmingham	UK	X-type	S	S	S	S	S	S	S	S
13.0610349	Birmingham	UK	lineage4	S	S	S	S	S	S	S	S
13.0613254	Birmingham	UK	lineage4	S	S	S	S	S	S	S	S
15.0601751	Birmingham	UK	Beijing	S	S	S	S	S	S	S	S
13.0613278	Birmingham	UK	Beijing	S	S	S	S	S	S	S	S
15.0601906	Birmingham	UK	lineage4	R	S	S	S	R	S	S	S
16.0601752	Birmingham	UK		S	S	S	S	F	F	F	F
12.0615206	Birmingham	UK	EAI	S	S	S	S	S	S	S	S
15.0607480	Birmingham	UK	Haarlem	S	S	S	S	F	S	S	F
14.0602480	Birmingham	UK	Delhi	S	S	S	S	S	S	S	S
15.0601742	Birmingham	UK	lineage4	S	S	S	S	S	S	S	S
13.0610346	Birmingham	UK	Delhi	S	S	S	S	S	S	S	S
13.0609466	Birmingham	UK	EAI	S	S	S	S	S	S	S	S
13.0604064	Birmingham	UK	lineage4	S	S	S	S	S	S	S	S
15.0605124	Birmingham	UK	EAI	R	n/a	S	S	R	R	U	S
14.0609182	Birmingham	UK	Delhi	S	S	S	S	S	S	S	S
13.0607137	Birmingham	UK	EAI	S	S	S	S	F	S	S	S
16.0607813	Birmingham	UK	lineage4	S	S	S	S	S	S	S	S
14.0609876	Birmingham	UK	LAM	S	S	S	S	S	S	S	S
15.0607173	Birmingham	UK	Uganda	R	S	S	S	R	S	S	S
12.0601810	Birmingham	UK	EAI	S	S	S	S	R	S	S	S
12.0616935	Birmingham	UK	lineage4	S	S	S	S	S	S	S	S
13.0617089	Birmingham	UK	Beijing	R	R	R	n/a	R	R	R	U
17.0601018	Birmingham	UK	Haarlem	S	S	S	S	S	S	S	S
16.0601687	Birmingham	UK	Delhi	S	S	S	S	S	S	S	S
15.0608693	Birmingham	UK	LAM	S	S	S	S	S	S	S	S
15.0601317	Birmingham	UK	lineage4	S	S	S	S	S	F	S	S
14.0600904	Birmingham	UK	lineage4	S	S	S	S	S	S	S	S
14.0609462	Birmingham	UK		S	S	S	S	S	S	S	S
12.0617275	Birmingham	UK	Delhi	S	S	S	S	S	S	S	S
16.0601953	Birmingham	UK	M. bovis	S	S	S	R	S	S	S	R
14.0609776	Birmingham	UK	lineage4	S	S	S	S	U	S	S	S
13.0604567	Birmingham	UK	EAI	S	S	S	S	S	S	S	S
15.0601961	Birmingham	UK	EAI	n/a	n/a	n/a	S	R	S	S	S
14.0609640	Birmingham	UK	Cameroon	S	S	S	S	S	U	S	S

14.0609535	Birmingham	UK	Haarlem	S	S	S	S	S	S	S	S
14.0616219	Birmingham	UK	Beijing	S	S	S	S	S	S	U	S
13.0610730	Birmingham	UK	Delhi	S	S	S	S	S	U	S	S
14.0600785	Birmingham	UK	Ural	S	S	S	S	S	S	S	S
13.0601569	Birmingham	UK	Delhi	S	S	S	S	S	S	S	S
15.0601160	Birmingham	UK	lineage4	S	S	S	S	S	S	S	S
12.0617575	Birmingham	UK	Delhi	S	S	S	S	S	S	S	S
15.0601366	Birmingham	UK	Delhi	S	S	S	S	S	S	S	S
13.0601220	Birmingham	UK	Haarlem	S	S	S	S	S	S	U	S
16.0601062	Birmingham	UK	Cameroon	S	S	S	S	S	S	S	S
13.0612972	Birmingham	UK	Delhi	S	S	S	S	S	U	S	S
14.0613174	Birmingham	UK	Delhi	S	S	S	S	S	S	S	S
16.0618455	Birmingham	UK	lineage4	S	S	S	S	S	U	S	S
15.0606741	Birmingham	UK	Delhi	S	S	S	S	S	F	S	S
15.0607149	Birmingham	UK	Delhi	S	S	S	S	S	F	S	S
16.0601950	Birmingham	UK	lineage4	S	S	S	S	S	S	U	S
14.0609143	Birmingham	UK	Beijing	S	S	S	S	S	S	S	S
11.0607157	Birmingham	UK	Uganda	S	S	S	S	S	S	S	S
15.0607585	Birmingham	UK	Beijing	S	R	S	S	S	R	S	S
15.0607970	Birmingham	UK	Delhi	S	S	S	S	S	S	S	S
14.0600957	Birmingham	UK	Delhi	S	S	S	S	S	S	S	S
15.0607425	Birmingham	UK	EAI	R	S	S	S	R	S	S	S
14.0600764	Birmingham	UK	X-type	S	S	S	S	S	S	S	S
11.0615356	Birmingham	UK	Delhi	S	S	S	S	S	S	S	S
15.0601319	Birmingham	UK	lineage4	S	S	S	S	S	S	S	S
13.0602972	Birmingham	UK	Delhi	S	S	S	S	S	S	S	S
12.0605695	Birmingham	UK	West Africar	S	S	S	S	U	U	U	S
14.0600033	Birmingham	UK	Delhi	S	S	S	S	S	S	S	S
16.0612916	Birmingham	UK	Cameroon	S	S	S	S	S	S	S	S
13.0604696	Birmingham	UK	Delhi	S	S	S	S	S	S	S	S
14.0600490	Birmingham	UK	Delhi	S	S	S	S	S	S	S	S
13.0601238	Birmingham	UK	Delhi	S	S	S	S	S	S	S	S
16.0607686	Birmingham	UK	Delhi	n/a	S	S	S	S	S	S	S
15.0605015	Birmingham	UK	Beijing	S	S	S	S	S	S	S	S
14.0600960	Birmingham	UK	Delhi	S	S	S	S	S	S	S	S
15.0607455	Birmingham	UK	Delhi	S	S	S	S	S	S	S	S
16.0618040	Birmingham	UK	Ural	S	S	S	S	S	S	S	S
15.0600444	Birmingham	UK	lineage4	S	S	S	S	S	S	S	S
13.0617132	Birmingham	UK	Cameroon	S	S	S	S	S	S	S	S
13.0608460	Birmingham	UK	lineage4	S	S	S	S	S	S	S	S
16.0607365	Birmingham	UK	lineage4	S	S	S	S	S	S	U	S
12.0610888	Birmingham	UK	EAI	S	S	S	S	S	S	S	S
11.0602455	Birmingham	UK	lineage4	S	S	S	S	S	S	S	S
15.0607142	Birmingham	UK	Delhi	S	S	S	S	S	S	U	S
12.0608700	Birmingham	UK	S-type	S	S	S	S	S	S	S	S
15.0613438	Birmingham	UK	lineage4	S	S	S	S	S	S	S	S
16.0607094	Birmingham	UK	Haarlem	R	S	S	S	R	S	S	S
11.0607052	Birmingham	UK	Haarlem	S	S	S	S	S	S	S	S
16.0604287	Birmingham	UK	LAM	S	S	S	S	S	S	S	S
13.0603078	Birmingham	UK	lineage4	S	S	S	S	S	S	S	S
13.0601264	Birmingham	UK	Delhi	S	S	S	S	S	S	S	S
16.0607435	Birmingham	UK	Delhi	S	S	S	S	S	F	F	F
15.0601123	Birmingham	UK	lineage4	S	S	S	S	S	S	S	S
12.0611838	Birmingham	UK	Cameroon	S	S	S	S	S	S	S	S
14.0600012	Birmingham	UK	X-type	S	S	S	S	S	S	S	S
10.0610060	Birmingham	UK	S-type	R	R	R	R	R	R	R	R
13.0609259	Birmingham	UK	Delhi	S	S	S	S	S	S	U	S
16.0601691	Birmingham	UK	Delhi	S	S	S	S	F	F	S	S
12.0614965	Birmingham	UK	lineage4	S	S	S	S	S	S	S	S
16.0610120	Birmingham	UK	lineage4	S	S	S	S	S	S	S	S
13.0616591	Birmingham	UK	Delhi	S	S	S	S	S	S	S	S
12.0614862	Birmingham	UK	EAI	S	S	S	S	R	S	S	S
13.0608229	Birmingham	UK	EAI	S	S	S	S	S	S	S	S
15.0607878	Birmingham	UK	LAM	S	S	S	S	S	S	S	S
15.0601300	Birmingham	UK	Haarlem	S	S	S	S	S	S	S	S
16.0618493	Birmingham	UK	Delhi	S	S	S	S	S	S	S	S
13.0613286	Birmingham	UK	Delhi	S	S	S	S	S	S	S	S
13.0602851	Birmingham	UK	lineage4	S	S	S	S	S	S	S	S
16.0601286	Birmingham	UK	EAI	S	S	S	n/a	S	S	S	S
15.0602702	Birmingham	UK	Haarlem	S	S	S	S	S	S	S	S
14.0609266	Birmingham	UK	Haarlem	S	S	S	S	S	S	S	S
15.0601932	Birmingham	UK	Delhi	S	S	S	S	S	S	S	S
14.0600498	Birmingham	UK	Delhi	S	S	S	S	S	S	S	S
15.0607706	Birmingham	UK	lineage4	S	S	S	S	S	S	S	S
13.0605100	Birmingham	UK	Delhi	S	S	S	S	S	S	S	S
12.0611851	Birmingham	UK	EAI	S	S	S	S	S	S	S	S
13.0601405	Birmingham	UK	Delhi	S	S	S	S	S	S	S	S
12.0612895	Birmingham	UK	Delhi	S	S	S	S	S	S	S	S
16.0601508	Birmingham	UK	LAM	S	S	S	S	S	S	S	S
13.0606320	Birmingham	UK	Delhi	S	S	S	S	S	S	S	S
12.0604987	Birmingham	UK	Delhi	S	S	S	S	S	S	S	S
12.0612947	Birmingham	UK	Delhi	S	S	S	S	S	S	S	S
13.0611313	Birmingham	UK	Delhi	S	S	S	S	S	S	S	S
13.0608428	Birmingham	UK	lineage4	S	S	S	S	S	S	S	S
13.0600598	Birmingham	UK	EAI	S	S	S	S	S	S	S	S
14.0609884	Birmingham	UK	Delhi	S	S	S	S	S	F	S	S
14.0610087	Birmingham	UK	EAI	n/a	n/a	n/a	S	S	S	S	S
13.0604511	Birmingham	UK	lineage4	S	S	S	S	S	S	S	S
14.0609396	Birmingham	UK	LAM	S	S	S	S	S	S	S	S
16.0617099	Birmingham	UK	Delhi	S	S	S	S	S	S	S	S
16.0607320	Birmingham	UK	lineage4	S	S	S	S	S	S	F	S
14.0600878	Birmingham	UK	Beijing	S	S	S	S	S	S	U	S
14.0600929	Birmingham	UK	EAI	S	S	S	S	S	S	S	S
14.0613230	Birmingham	UK	Delhi	S	S	S	S	S	S	S	S
15.0615980	Birmingham	UK	Delhi	S	S	S	S	S	F	S	S
13.0613166	Birmingham	UK	Beijing	S	S	S	S	U	S	S	S
14.0609779	Birmingham	UK	Delhi	S	S	S	S	S	S	S	S
13.0600253	Birmingham	UK	Haarlem	S	S	S	S	S	S	S	S
14.0600478	Birmingham	UK	Beijing	S	S	S	S	S	S	S	S
14.0616114	Birmingham	UK	LAM	S	S	S	S	S	S	S	S
14.0600198	Birmingham	UK	Cameroon	S	S	S	S	S	S	S	S
14.0616098	Birmingham	UK	Delhi	S	S	S	S	S	S	S	S
15.0607364	Birmingham	UK	Delhi	S	S	S	S	S	S	S	S
15.0607080	Birmingham	UK	Delhi	R	S	S	S	S	S	S	S
13.0609008	Birmingham	UK	EAI	S	S	S	S	S	S	S	S
15.0601715	Birmingham	UK	Haarlem	S	S	S	S	S	S	U	S
16.0618585	Birmingham	UK	lineage4	n/a	S	n/a	n/a	S	S	S	S
16.0609811	Birmingham	UK	lineage4	S	S	S	S	S	U	S	S
15.0607452	Birmingham	UK	Delhi	S	S	S	S	S	S	S	S
16.0601607	Birmingham	UK	Ural	S	S	S	S	S	S	S	S
16.0607742	Birmingham	UK	Beijing	S	S	S	S	S	S	S	S
13.0613530	Birmingham	UK	EAI	S	S	S	S	S	S	S	S
14.0614114	Birmingham	UK	Delhi	R	S	S	S	R	F	S	S
13.0604545	Birmingham	UK	X-type	S	S	S	S	S	S	S	S
16.0601474	Birmingham	UK	Delhi	S	S	S	S	F	F	F	S
16.0601417	Birmingham	UK		S	S	S	S	S	S	U	S

13.0613390	Birmingham	UK	EAI	S	S	S	S	S	S	S	S
13.0607681	Birmingham	UK	Haarlem	S	S	S	S	S	U	S	S
13.0613283	Birmingham	UK	Beijing	S	S	S	S	U	U	S	S
16.0601708	Birmingham	UK	Delhi	S	S	S	S	S	S	S	S
13.0613363	Birmingham	UK	LAM	S	S	S	S	S	S	S	S
15.0607091	Birmingham	UK	Haarlem	S	S	S	S	S	S	S	S
15.0601039	Birmingham	UK	Beijing	R	R	R	R	R	R	R	R
12.0616150	Birmingham	UK	Delhi	S	S	S	S	S	S	S	S
15.0607977	Birmingham	UK	lineage4	S	S	S	S	S	S	S	S
14.0600483	Birmingham	UK	Delhi	S	S	S	S	S	S	S	S
16.0601858	Birmingham	UK	LAM	S	S	S	S	S	S	S	S
16.0601807	Birmingham	UK	Delhi	S	S	S	S	S	S	S	S
13.0610501	Birmingham	UK	Delhi	S	S	S	S	S	S	U	S
16.0607971	Birmingham	UK	LAM	S	S	S	S	S	S	S	S
15.0601947	Birmingham	UK	Beijing	R	S	S	S	R	S	S	S
16.0607623	Birmingham	UK	Delhi	S	S	S	S	S	S	S	S
15.0607361	Birmingham	UK	EAI	n/a	S	S	n/a	R	S	S	S
16.0609590	Birmingham	UK	Ural	S	S	S	S	S	S	S	S
13.0612199	Birmingham	UK	Delhi	S	S	S	S	S	S	S	S
15.0603457	Birmingham	UK	X-type	S	S	S	S	S	S	S	S
16.0618594	Birmingham	UK	EAI	S	S	n/a	S	S	S	S	S
12.0610033	Birmingham	UK	lineage4	S	S	S	S	S	S	S	S
15.0607293	Birmingham	UK	Cameroon	S	S	S	S	S	S	U	S
15.0605004	Birmingham	UK	Beijing	S	S	S	S	S	S	S	S
15.0601085	Birmingham	UK	Delhi	S	S	S	S	S	S	U	S
16.0601242	Birmingham	UK	EAI	S	S	S	S	S	S	S	S
13.0611286	Birmingham	UK	LAM	S	S	S	S	S	S	S	S
14.0610078	Birmingham	UK	Delhi	S	S	S	S	S	S	S	S
15.0613003	Birmingham	UK	Haarlem	S	S	S	S	S	S	S	S
15.0601424	Birmingham	UK	LAM	S	S	S	S	S	S	S	S
13.0608404	Birmingham	UK	Beijing	S	S	S	S	S	S	S	S
15.0601507	Birmingham	UK	M. bovis	S	S	S	R	S	S	S	R
14.0600672	Birmingham	UK	EAI	S	S	S	S	S	S	S	S
16.0601983	Birmingham	UK	Haarlem	S	S	S	S	S	S	S	S
16.0609809	Birmingham	UK	lineage4	S	S	S	S	S	U	U	S
14.0601636	Birmingham	UK	EAI	S	S	S	S	S	S	S	S
13.0607686	Birmingham	UK	Delhi	S	S	S	S	S	S	U	S
15.0613464	Birmingham	UK	lineage4	S	S	S	S	S	S	S	S
11.0612674	Birmingham	UK	M. bovis	S	S	S	R	S	S	U	R
15.0601763	Birmingham	UK	lineage4	S	S	S	S	S	U	S	S
16.0600783	Birmingham	UK	Beijing	S	S	S	S	S	S	S	S
12.0616403	Birmingham	UK	Delhi	S	S	S	S	S	S	S	S
16.0601848	Birmingham	UK	Beijing	R	R	R	n/a	R	R	R	R
12.0615274	Birmingham	UK	EAI	S	S	S	S	S	S	U	S
15.0608004	Birmingham	UK	X-type	S	S	S	n/a	S	S	S	R
16.0609115	Birmingham	UK	Haarlem	S	S	S	S	S	F	U	S
15.0601214	Birmingham	UK	Delhi	S	S	S	S	S	S	S	S
14.0600541	Birmingham	UK	Delhi	S	S	S	S	S	S	S	S
15.0601846	Birmingham	UK	EAI	S	S	S	S	S	S	S	S
16.0601846	Birmingham	UK	X-type	S	S	S	S	S	S	S	S
15.0613408	Birmingham	UK	Delhi	S	S	S	S	S	F	S	F
13.0608231	Birmingham	UK	Beijing	R	R	R	S	R	R	R	R
13.0603885	Birmingham	UK	Delhi	S	S	S	S	S	S	S	S
16.0606170	Birmingham	UK	LAM	S	S	S	S	S	F	S	S
13.0613359	Birmingham	UK	EAI	S	S	S	S	S	S	S	S
13.0606487	Birmingham	UK	lineage4	S	S	S	S	S	S	S	S
14.0600959	Birmingham	UK	Delhi	S	S	S	S	S	S	S	S
16.0607966	Birmingham	UK	Beijing	S	S	S	S	S	S	S	S
16.0617111	Birmingham	UK	Delhi	S	S	S	S	S	S	S	S
14.0600512	Birmingham	UK	Delhi	S	S	S	S	S	S	S	S
15.0601496	Birmingham	UK	EAI	S	S	S	S	S	F	S	S
16.0601346	Birmingham	UK	Delhi	S	S	S	S	S	S	S	S
15.0601479	Birmingham	UK	LAM	S	S	S	S	S	F	S	S
12.0611175	Birmingham	UK	lineage4	S	S	S	S	S	S	S	S
14.0604248	Birmingham	UK	Delhi	S	S	S	S	S	S	S	S
14.0609922	Birmingham	UK	lineage4	S	S	S	S	S	S	S	S
12.0614472	Birmingham	UK	Delhi	S	S	S	S	S	S	S	S
12.0617719	Birmingham	UK	Cameroon	S	S	S	S	S	S	U	S
12.0610293	Birmingham	UK	lineage4	S	S	S	S	S	S	U	S
15.0610529	Birmingham	UK	EAI	S	S	S	S	S	S	S	S
14.0616050	Birmingham	UK	Uganda	S	S	S	S	S	S	S	S
15.0601082	Birmingham	UK	EAI	S	S	S	S	S	S	S	S
14.0600946	Birmingham	UK	Beijing	R	R	R	R	R	R	R	R
13.0601227	Birmingham	UK	Delhi	S	S	S	S	S	S	S	S
15.0601639	Birmingham	UK	Delhi	S	S	S	S	S	S	S	S
12.0605653	Birmingham	UK	EAI	S	S	S	S	R	R	S	S
14.0600570	Birmingham	UK	EAI	S	S	S	S	S	S	U	S
13.0607982	Birmingham	UK	lineage4	S	S	S	S	S	S	S	S
12.0612856	Birmingham	UK	Delhi	S	S	S	S	S	S	S	S
15.0607898	Birmingham	UK	S-type	S	S	S	S	S	U	S	S
16.0604675	Birmingham	UK	EAI	S	S	S	S	S	S	S	F
15.0607266	Birmingham	UK	EAI	S	S	S	S	S	S	S	S
12.0614645	Birmingham	UK	Delhi	S	S	S	S	S	S	S	S
14.0600897	Birmingham	UK	Delhi	S	S	S	S	S	S	U	S
15.0613289	Birmingham	UK	Tur	S	S	S	S	F	F	F	F
13.0609341	Birmingham	UK	Beijing	S	S	S	S	S	S	S	S
15.0607972	Birmingham	UK	X-type	S	S	S	S	S	S	S	S
16.0602006	Birmingham	UK	X-type	S	S	S	S	S	S	U	S
12.0927508	Birmingham	UK	Delhi	S	S	S	S	S	S	S	S
15.0613326	Birmingham	UK	Delhi	S	S	S	S	S	F	S	S
17.0601042	Birmingham	UK	Delhi	S	S	S	S	S	S	S	S
13.0610650	Birmingham	UK	Haarlem	S	S	S	S	S	S	U	S
15.0607489	Birmingham	UK	M. orygis	S	S	S	S	S	S	U	S
13.0613585	Birmingham	UK	Delhi	S	S	S	S	S	S	S	S
14.0609988	Birmingham	UK	Haarlem	S	S	S	S	S	S	S	S
13.0613516	Birmingham	UK	lineage4	S	S	S	S	S	S	U	S
16.0607631	Birmingham	UK	S-type	S	S	S	S	S	S	S	S
15.0611200	Birmingham	UK	Delhi	R	R	S	S	R	R	S	S
12.0613012	Birmingham	UK	EAI	S	S	S	S	S	S	S	S
14.0600128	Birmingham	UK	Ural	S	S	S	S	S	S	S	S
14.0614275	Birmingham	UK	LAM	S	S	S	S	S	S	S	S
13.0611326	Birmingham	UK	lineage4	S	S	S	S	S	S	S	S
16.0609437	Birmingham	UK	Haarlem	S	S	S	S	S	S	S	S
13.0613281	Birmingham	UK	S-type	S	S	S	S	U	S	U	S
15.0601925	Birmingham	UK	Delhi	S	S	S	S	S	S	S	S
13.0607693	Birmingham	UK	Delhi	S	S	S	S	S	S	S	S
13.0606204	Birmingham	UK	X-type	S	S	S	S	S	S	S	S
13.0602828	Birmingham	UK	EAI	S	S	S	S	S	S	S	S
15.0601041	Birmingham	UK	LAM	R	S	S	S	R	R	S	S
16.0601485	Birmingham	UK	lineage4	S	S	S	S	S	S	S	S
15.0604486	Birmingham	UK	EAI	S	S	S	S	S	S	S	S
13.0606212	Birmingham	UK	Haarlem	S	S	S	S	S	S	U	U
15.0601346	Birmingham	UK	Delhi	S	S	S	S	S	S	S	S
15.0607280	Birmingham	UK	EAI	S	S	S	S	S	S	S	S
16.0618131	Birmingham	UK	Delhi	S	S	S	S	S	S	S	S
12.0611336	Birmingham	UK	Delhi	S	S	S	S	S	S	S	S

13.0601533	Birmingham	UK	Delhi	S	S	S	S	S	S	S	S
09.0600086	Birmingham	UK	Delhi	S	S	S	S	S	S	S	S
15.0600714	Birmingham	UK	lineage4	S	S	S	S	S	S	S	S
15.0601595	Birmingham	UK	Delhi	S	S	S	S	S	S	S	S
16.0618275	Birmingham	UK	lineage4	S	S	S	S	S	S	S	S
11.0601764	Birmingham	UK	X-type	S	S	S	S	S	F	U	S
12.0616916	Birmingham	UK	Delhi	S	S	S	S	S	S	S	S
16.0617033	Birmingham	UK	Haarlem	S	S	S	S	S	S	S	S
16.0617332	Birmingham	UK	X-type	S	S	S	S	S	S	S	S
15.0601563	Birmingham	UK	Ural	S	S	S	S	S	S	U	S
13.0616964	Birmingham	UK	Delhi	S	S	S	S	S	S	S	S
16.0607174	Birmingham	UK	Delhi	S	S	S	S	S	S	S	S
14.0600005	Birmingham	UK	Cameroon	S	S	S	S	S	S	S	S
15.0607691	Birmingham	UK	EAI	S	S	S	n/a	S	F	S	S
12.0613649	Birmingham	UK	Delhi	S	S	S	S	S	S	U	S
13.0600087	Birmingham	UK	EAI	S	S	S	S	F	S	S	S
15.0601291	Birmingham	UK	Delhi	S	S	S	S	S	S	S	S
16.0618320	Birmingham	UK	Delhi	S	S	n/a	n/a	S	S	U	S
13.0605637	Birmingham	UK	Delhi	S	S	S	S	U	S	S	S
13.0607992	Birmingham	UK	Haarlem	S	S	S	n/a	S	S	S	S
15.0615243	Birmingham	UK	Delhi	S	S	S	S	S	S	S	S
16.0618609	Birmingham	UK	Uganda	S	S	S	S	S	S	S	F
14.0602707	Birmingham	UK	Delhi	S	S	S	S	S	S	S	S
12.0610286	Birmingham	UK	Delhi	S	S	S	S	S	S	S	S
15.0601757	Birmingham	UK		S	S	S	R	S	S	S	R
15.0607824	Birmingham	UK	LAM	S	S	S	S	S	S	S	S
15.0613448	Birmingham	UK	Delhi	S	S	S	S	S	S	S	S
15.0607797	Birmingham	UK	EAI	S	S	S	S	S	U	S	S
14.0600220	Birmingham	UK	Cameroon	S	S	S	S	S	S	S	S
12.0614857	Birmingham	UK	Delhi	S	S	S	S	S	S	S	S
16.0601541	Birmingham	UK	Beijing	S	S	S	S	S	S	S	S
14.0616096	Birmingham	UK	lineage4	n/a	n/a	n/a	S	S	S	S	S
14.0604994	Birmingham	UK	X-type	S	S	S	S	S	S	S	S
13.0606726	Birmingham	UK	EAI	S	S	S	S	S	S	S	S
15.0601638	Birmingham	UK	S-type	S	S	S	S	S	S	S	S
14.0609476	Birmingham	UK	Delhi	S	S	S	S	S	S	S	S
15.0601883	Birmingham	UK	Delhi	S	S	S	S	S	S	S	S
16.0602436	Birmingham	UK	LAM	S	S	S	S	S	S	S	S
15.0601417	Birmingham	UK	Ural	S	S	S	S	S	S	S	S
12.0615290	Birmingham	UK	Haarlem	S	S	S	S	S	S	S	S
13.0609012	Birmingham	UK	lineage4	S	S	S	S	S	S	S	S
14.0611676	Birmingham	UK	Delhi	S	S	S	S	U	S	S	S
15.0607528	Birmingham	UK	Uganda	S	S	S	S	S	S	S	S
13.0602088	Birmingham	UK	EAI	S	S	S	S	F	S	S	S
13.0605704	Birmingham	UK	Delhi	S	S	S	S	S	S	U	S
15.0607978	Birmingham	UK	Delhi	S	S	S	S	S	U	S	S
14.0609856	Birmingham	UK	Ural	S	S	S	S	S	S	S	S
13.0611717	Birmingham	UK	EAI	S	S	S	S	S	S	S	S
15.0609023	Birmingham	UK	Haarlem	S	S	S	S	S	S	S	S
14.0604679	Birmingham	UK	Haarlem	S	S	S	S	S	S	S	S
13.0602060	Birmingham	UK	Delhi	S	S	S	S	S	S	S	S
16.0601281	Birmingham	UK	EAI	S	S	S	S	S	S	U	S
14.0609152	Birmingham	UK	Delhi	S	S	S	S	S	S	S	S
15.0607823	Birmingham	UK	X-type	S	S	S	S	S	S	S	S
16.0607377	Birmingham	UK	Haarlem	S	S	S	S	S	S	S	S
17.0601020	Birmingham	UK	Haarlem	S	S	S	S	S	S	S	S
14.0600283	Birmingham	UK	Beijing	S	S	S	S	S	S	U	S
16.0607455	Birmingham	UK	Beijing	S	S	S	S	S	S	S	S
16.0601958	Birmingham	UK	lineage4	S	S	S	S	S	S	S	S
12.0616562	Birmingham	UK	EAI	S	S	S	S	S	S	S	S
14.0600251	Birmingham	UK	X-type	S	S	S	S	S	S	S	S
16.0618201	Birmingham	UK	Delhi	S	S	S	S	S	S	S	S
16.0601074	Birmingham	UK	Delhi	S	S	S	n/a	U	S	S	S
15.0613009	Birmingham	UK	lineage4	S	S	S	S	S	S	S	S
15.0607719	Birmingham	UK		S	S	S	S	S	S	S	S
14.0600384	Birmingham	UK	X-type	S	S	S	S	S	S	S	S
12.0612484	Birmingham	UK	Delhi	R	S	S	S	U	S	S	S
15.0611635	Birmingham	UK	EAI	S	S	S	n/a	S	S	U	S
14.0600647	Birmingham	UK	Beijing	S	S	S	S	R	S	S	S
16.0617167	Birmingham	UK	LAM	S	S	S	S	S	S	S	S
14.0616099	Birmingham	UK	Delhi	S	S	S	S	S	S	S	S
12.0613641	Birmingham	UK	Cameroon	S	S	S	S	R	S	S	S
13.0601212	Birmingham	UK	Delhi	S	S	S	S	S	S	S	S
16.0602460	Birmingham	UK	Haarlem	S	S	S	S	S	S	U	S
12.0927475	Birmingham	UK	LAM	S	S	S	S	S	S	S	S
14.0607894	Birmingham	UK	EAI	S	S	S	S	S	S	S	S
12.0604645	Birmingham	UK	Delhi	S	S	S	S	S	S	S	S
11.0612559	Birmingham	UK	Delhi	S	S	S	S	S	U	U	S
15.0609310	Birmingham	UK	Delhi	S	S	S	S	S	S	S	S
13.0604894	Birmingham	UK	Delhi	S	S	S	S	U	S	S	S
13.0609877	Birmingham	UK	Delhi	S	S	S	S	S	S	S	S
12.0611203	Birmingham	UK	Delhi	S	S	S	S	S	S	S	S
13.0609263	Birmingham	UK	EAI	S	S	S	S	S	S	S	S
14.0600049	Birmingham	UK	X-type	S	S	S	S	S	S	S	S
16.0605810	Birmingham	UK	Delhi	S	S	S	S	S	S	S	S
13.0617617	Birmingham	UK	lineage4	S	S	S	S	S	U	S	S
16.0618395	Birmingham	UK	lineage4	S	S	S	S	S	S	U	S
16.0601028	Birmingham	UK	S-type	S	S	S	S	S	S	S	S
13.0612210	Birmingham	UK	EAI	S	S	S	S	S	S	S	S
16.0607895	Birmingham	UK	Delhi	S	S	S	S	S	S	S	S
14.0616160	Birmingham	UK	Ural	S	S	S	S	S	S	S	S
15.0607213	Birmingham	UK	Delhi	S	S	S	S	S	S	U	S
16.0618520	Birmingham	UK	Cameroon	S	S	S	n/a	S	S	U	S
15.0609411	Birmingham	UK	LAM	S	S	S	S	S	S	S	S
14.0609450	Birmingham	UK	Beijing	S	S	S	S	S	S	U	S
13.0600307	Birmingham	UK	Delhi	S	S	S	S	S	S	S	S
15.0601415	Birmingham	UK	X-type	S	S	S	S	S	S	S	S
14.0615356	Birmingham	UK	Delhi	S	S	S	S	S	S	S	S
13.0613507	Birmingham	UK	Cameroon	S	S	S	S	S	S	S	S
16.0601570	Birmingham	UK	Cameroon	S	S	S	S	S	S	S	S
14.0616195	Birmingham	UK	Beijing	n/a	n/a	n/a	S	R	R	R	R
16.0601837	Birmingham	UK	Delhi	S	S	S	S	S	S	S	S
14.0613841	Birmingham	UK	Delhi	S	S	S	S	S	S	S	S
13.0609459	Birmingham	UK	Delhi	S	S	S	S	S	S	S	S
15.0613064	Birmingham	UK	Delhi	S	S	S	S	S	S	S	S
16.0603813	Birmingham	UK	Delhi	S	S	S	S	S	S	S	S
14.0600200	Birmingham	UK	Delhi	S	S	S	S	S	S	S	S
15.0613472	Birmingham	UK	lineage4	S	S	S	S	S	S	U	S
15.0612112	Birmingham	UK	Delhi	S	S	S	S	S	S	S	S
13.0605186	Birmingham	UK	LAM	S	S	S	S	S	S	S	S
11.0612877	Birmingham	UK	Delhi	S	S	S	S	S	S	S	S
15.0601413	Birmingham	UK	EAI	S	S	S	S	F	S	F	S
15.0607666	Birmingham	UK	lineage4	S	S	S	S	S	S	S	S
14.0609144	Birmingham	UK	lineage4	S	S	S	S	S	S	S	S
15.0600566	Birmingham	UK	Delhi	S	S	S	S	S	S	S	S
13.0601980	Birmingham	UK	Beijing	S	S	S	S	S	S	S	S

12.0617576	Birmingham	UK	EAI	S	S	S	S	S	S	S	S
13.0603862	Birmingham	UK	EAI	S	S	S	S	S	S	S	S
12.0608757	Birmingham	UK	Delhi	S	S	S	S	S	S	S	S
13.0607993	Birmingham	UK	lineage4	S	S	S	S	S	S	S	S
16.0601943	Birmingham	UK	Delhi	S	S	S	S	S	S	S	S
15.0613403	Birmingham	UK	Delhi	S	S	S	S	S	F	S	S
16.0602000	Birmingham	UK	Delhi	S	S	S	S	U	S	S	S
14.0600442	Birmingham	UK	Delhi	S	S	S	S	S	S	S	S
16.0607907	Birmingham	UK	lineage4	S	S	S	S	S	S	S	S
16.0601994	Birmingham	UK	Delhi	S	S	S	S	S	S	S	S
14.0616025	Birmingham	UK	Delhi	S	S	S	S	S	S	S	S
13.0612457	Birmingham	UK	lineage4	S	S	S	S	S	S	S	S
14.0609953	Birmingham	UK	EAI	S	S	S	S	S	S	S	S
16.0601580	Birmingham	UK	Delhi	S	S	S	S	S	S	S	S
14.0611632	Birmingham	UK	Delhi	S	S	S	S	S	S	S	S
15.0607621	Birmingham	UK	Beijing	S	S	S	n/a	S	S	S	S
13.0608405	Birmingham	UK	Delhi	S	S	S	S	S	S	S	S
15.0601486	Birmingham	UK	lineage4	S	S	S	S	S	S	S	S
12.0617565	Birmingham	UK	Delhi	S	S	S	S	S	S	S	S
14.0600447	Birmingham	UK	X-type	S	S	S	S	S	S	S	S
15.0603289	Birmingham	UK	Cameroon	S	S	S	S	S	S	S	S
14.0600911	Birmingham	UK	Delhi	S	S	S	S	S	S	S	S
16.0618311	Birmingham	UK	Delhi	n/a	n/a	n/a	S	S	S	U	S
13.0611341	Birmingham	UK	X-type	S	S	S	S	S	S	S	S
14.0613857	Birmingham	UK	M. bovis	n/a	S	S	R	S	S	S	R
14.0600265	Birmingham	UK	Ural	R	R	S	R	R	R	R	U
14.0613898	Birmingham	UK	LAM	S	S	S	S	S	S	S	S
14.0600716	Birmingham	UK	Delhi	S	S	S	S	F	S	S	S
16.0617565	Birmingham	UK	LAM	S	S	S	S	S	S	S	S
13.0609248	Birmingham	UK	Beijing	S	S	S	S	S	S	S	S
15.0601614	Birmingham	UK	Delhi	S	S	S	S	S	S	S	S
13.0606635	Birmingham	UK	EAI	S	S	S	S	S	S	U	S
13.0610747	Birmingham	UK	Delhi	S	S	S	S	S	S	S	S
13.0607916	Birmingham	UK	Delhi	S	S	S	S	S	S	S	S
13.0613127	Birmingham	UK	EAI	S	S	S	S	S	S	S	S
16.0618401	Birmingham	UK	Cameroon	S	S	S	S	S	S	S	S
15.0601306	Birmingham	UK	Delhi	S	S	S	S	S	S	S	S
16.0613389	Birmingham	UK	lineage4	S	S	S	S	S	S	S	S
12.0611891	Birmingham	UK	Delhi	S	S	S	S	S	S	S	S
16.0617385	Birmingham	UK	LAM	S	S	S	S	S	S	U	S
15.0613554	Birmingham	UK	Delhi	S	S	S	S	S	S	S	S
15.0607651	Birmingham	UK	Haarlem	S	S	S	S	S	S	S	S
14.0609943	Birmingham	UK	lineage4	S	S	S	S	S	S	S	S
13.0613593	Birmingham	UK	LAM	S	S	S	S	S	S	S	S
16.0601258	Birmingham	UK	lineage4	S	S	S	S	S	S	S	S
12.0615000	Birmingham	UK	Delhi	S	S	S	S	S	S	S	S
14.0615443	Birmingham	UK	M. orygis	n/a	S	S	S	U	S	S	S
16.0607379	Birmingham	UK	LAM	S	S	S	S	S	S	S	S
12.0616928	Birmingham	UK	lineage4	S	S	S	S	S	S	S	S
16.0618160	Birmingham	UK	Haarlem	S	S	S	S	F	S	U	S
15.0601466	Birmingham	UK	Delhi	S	S	S	S	S	S	S	S
12.0617714	Birmingham	UK	Delhi	S	S	S	S	F	S	S	S
15.0601658	Birmingham	UK	Delhi	S	S	S	S	S	S	S	S
15.0602905	Birmingham	UK	X-type	R	R	R	n/a	R	R	R	F
16.0618376	Birmingham	UK	lineage4	S	S	S	S	S	S	S	S
16.0602771	Birmingham	UK	Delhi	S	S	S	S	S	S	U	S
13.0602841	Birmingham	UK	Delhi	S	S	S	S	S	S	S	S
16.0601331	Birmingham	UK	Delhi	R	S	S	S	R	S	S	S
13.0618224	Birmingham	UK	Delhi	S	S	S	S	S	S	S	S
12.0611350	Birmingham	UK	Delhi	S	S	S	S	S	S	S	S
13.0605248	Birmingham	UK	Delhi	S	S	S	S	S	S	S	S
14.0609485	Birmingham	UK	Delhi	S	S	S	S	S	S	S	S
16.0617110	Birmingham	UK	X-type	S	S	S	n/a	S	S	S	S
15.0613447	Birmingham	UK		S	S	S	S	S	S	S	S
16.0607591	Birmingham	UK	lineage4	S	S	S	S	S	S	S	S
14.0600390	Birmingham	UK	X-type	S	S	S	S	S	F	S	S
14.0600505	Birmingham	UK	Delhi	S	S	S	S	S	S	S	R
16.0601588	Birmingham	UK	Delhi	S	S	S	S	S	S	S	S
14.0616179	Birmingham	UK	West Africar	S	S	S	S	S	S	U	S
14.0609805	Birmingham	UK	EAI	S	S	S	S	S	S	S	S
11.0605441	Birmingham	UK	lineage4	S	S	S	S	S	S	S	S
15.0607544	Birmingham	UK	Delhi	S	S	S	S	S	S	S	S
14.0607524	Birmingham	UK	Delhi	S	S	S	S	S	S	S	S
13.0605182	Birmingham	UK	Uganda	S	S	S	S	S	S	S	S
12.0610867	Birmingham	UK	Delhi	S	S	S	S	S	S	S	S
16.0607561	Birmingham	UK	X-type	S	S	S	S	S	S	S	S
15.0611196	Birmingham	UK	X-type	S	S	S	S	S	S	S	S
15.0607500	Birmingham	UK	Delhi	S	S	S	S	S	S	S	S
15.0601487	Birmingham	UK	lineage4	S	S	S	S	S	S	S	S
12.0927149	Birmingham	UK	lineage4	S	S	S	S	S	S	S	S
15.0604059	Birmingham	UK	Delhi	S	S	S	S	S	S	S	S
16.0603746	Birmingham	UK	LAM	S	S	S	n/a	S	S	S	S
15.0607130	Birmingham	UK	Beijing	S	S	S	S	S	S	S	S
15.0607737	Birmingham	UK	LAM	R	R	R	R	R	R	R	R
16.0618601	Birmingham	UK	EAI	S	S	n/a	S	S	S	S	S
16.0618354	Birmingham	UK	EAI	S	S	S	n/a	S	S	S	S
15.0615809	Birmingham	UK	Delhi	S	S	S	S	S	S	S	S
13.0604363	Birmingham	UK	X-type	S	S	S	S	S	S	S	S
14.0602202	Birmingham	UK	Delhi	S	S	S	S	S	S	S	S
16.0618545	Birmingham	UK	lineage4	S	S	S	S	S	S	S	S
17.0601106	Birmingham	UK	LAM	n/a	n/a	n/a	S	S	S	S	S
16.0607074	Birmingham	UK	X-type	n/a	n/a	n/a	S	S	S	S	S
16.0618240	Birmingham	UK	Delhi	S	S	S	S	S	S	S	S
16.0607861	Birmingham	UK	Delhi	S	S	S	S	S	S	U	S
14.0609501	Birmingham	UK	Delhi	S	S	S	S	S	S	S	S
14.0609343	Birmingham	UK	Beijing	S	S	S	S	S	S	U	S
11.0604389	Birmingham	UK	lineage4	S	S	S	S	S	S	S	S
16.0607177	Birmingham	UK	EAI	S	S	S	S	S	S	S	S
16.0618151	Birmingham	UK	EAI	S	S	S	S	S	S	S	S
13.0607957	Birmingham	UK	M. orygis	n/a	n/a	n/a	S	S	S	S	S
16.0617625	Birmingham	UK	Delhi	n/a	S	S	S	R	S	S	S
11.0603912	Birmingham	UK	lineage4	S	S	S	S	S	S	S	S
14.0600308	Birmingham	UK	Delhi	S	S	S	S	S	S	S	S
13.0613142	Birmingham	UK	Beijing	S	S	S	S	S	S	S	S
13.0608457	Birmingham	UK	lineage4	S	S	S	S	S	S	U	S
12.0610841	Birmingham	UK	X-type	S	S	S	S	S	S	S	S
15.0607530	Birmingham	UK	lineage4	S	S	S	S	S	S	S	S
13.0607978	Birmingham	UK	Delhi	S	S	S	S	S	S	S	S
13.0603877	Birmingham	UK	Delhi	S	S	S	S	S	S	S	S
15.0607673	Birmingham	UK	Delhi	S	S	S	S	S	S	S	S
14.0609799	Birmingham	UK	EAI	S	S	S	S	F	F	S	F
15.0602409	Birmingham	UK	Delhi	S	S	S	S	S	S	U	S
16.0601528	Birmingham	UK	Delhi	S	S	S	S	S	S	S	S
09.0600447	Birmingham	UK	Delhi	S	S	S	S	S	S	S	S
12.0615271	Birmingham	UK	Delhi	S	S	S	S	S	S	S	S
16.0617398	Birmingham	UK	Tur	S	S	S	S	S	S	S	S

13.0614577	Birmingham	UK	EAI	S	S	S	S	S	S	S	S
16.0601565	Birmingham	UK	EAI	S	S	S	S	S	S	S	S
13.0613385	Birmingham	UK	Delhi	S	S	S	S	S	S	S	S
14.0609893	Birmingham	UK	lineage4	S	S	S	S	S	S	S	S
15.0607228	Birmingham	UK	Delhi	S	S	S	S	S	S	S	S
14.0607266	Birmingham	UK	Delhi	S	S	S	S	S	S	S	S
13.0608435	Birmingham	UK	LAM	S	S	S	S	S	S	S	S
12.0614865	Birmingham	UK	X-type	S	S	S	S	S	S	S	S
13.0603129	Birmingham	UK	EAI	S	S	S	S	S	S	S	S
15.0607424	Birmingham	UK	Tur	S	S	S	S	S	F	S	S
12.0616801	Birmingham	UK	LAM	S	S	S	S	S	S	S	S
12.0614223	Birmingham	UK	EAI	S	S	S	S	S	U	S	S
15.0601251	Birmingham	UK	Haarlem	S	S	S	S	S	S	S	S
12.0605156	Birmingham	UK	Beijing	S	S	S	S	S	S	S	S
12.0608436	Birmingham	UK	Delhi	S	S	S	S	S	S	S	S
15.0601292	Birmingham	UK	Haarlem	S	S	S	S	S	S	S	S
15.0607441	Birmingham	UK	Delhi	S	S	S	S	S	S	S	S
16.0618267	Birmingham	UK	S-type	S	S	S	S	S	S	S	S
14.0616145	Birmingham	UK	Beijing	S	S	S	S	S	S	S	S
14.0612015	Birmingham	UK	lineage4	S	S	S	S	F	S	S	S
13.0613472	Birmingham	UK	Cameroon	S	S	S	S	S	S	S	S
16.0601785	Birmingham	UK	lineage4	S	S	S	S	S	S	S	S
16.0607158	Birmingham	UK	Delhi	S	S	S	S	S	S	U	S
13.0611322	Birmingham	UK	lineage4	S	S	S	S	S	S	S	S
14.0602863	Birmingham	UK	Delhi	S	S	S	S	S	S	S	S
15.0611433	Birmingham	UK	Beijing	R	R	R	n/a	R	R	R	R
13.0613490	Birmingham	UK	X-type	S	S	S	S	S	S	S	S
16.0618577	Birmingham	UK	Beijing	S	S	S	S	S	S	S	S
16.0617189	Birmingham	UK	lineage4	S	S	S	S	S	S	S	S
15.0601313	Birmingham	UK	Haarlem	R	S	S	S	R	S	R	S
14.0613749	Birmingham	UK	EAI	S	S	S	S	U	S	S	S
13.0601230	Birmingham	UK	Cameroon	S	S	S	S	S	S	S	S
15.0601151	Birmingham	UK	lineage4	S	S	S	S	S	S	S	S
13.0610327	Birmingham	UK	Beijing	S	S	S	S	S	S	S	S
13.0613586	Birmingham	UK	Delhi	S	S	S	S	S	S	U	S
16.0601430	Birmingham	UK		S	S	S	S	S	S	S	S
16.0607717	Birmingham	UK	Beijing	S	S	S	S	F	S	S	F
16.0611544	Birmingham	UK	Cameroon	S	S	S	S	S	S	S	S
12.0607008	Birmingham	UK	Delhi	S	S	S	S	S	S	S	S
13.0613509	Birmingham	UK	Delhi	S	S	S	R	S	S	S	R
12.0614091	Birmingham	UK	Tur	S	S	S	S	S	S	S	S
13.0611297	Birmingham	UK	lineage4	S	S	S	S	S	S	S	S
15.0605888	Birmingham	UK	LAM	S	S	S	S	S	S	S	S
16.0601667	Birmingham	UK	Delhi	S	S	S	S	S	S	S	S
15.0607389	Birmingham	UK	lineage4	S	S	S	S	S	S	S	S
15.0613466	Birmingham	UK	LAM	S	S	S	S	S	U	U	S
15.0607836	Birmingham	UK	EAI	S	S	S	S	S	S	S	S
11.0614563	Birmingham	UK	West Africar	S	S	S	S	S	S	U	S
13.0607393	Birmingham	UK	Delhi	S	S	S	S	S	S	S	S
13.0605714	Birmingham	UK	lineage4	S	S	S	S	S	S	S	S
15.0601454	Birmingham	UK	Delhi	S	S	S	S	S	S	S	S
15.0607568	Birmingham	UK	Delhi	S	S	S	S	S	S	S	S
14.0609170	Birmingham	UK	Delhi	S	S	S	S	S	S	S	S
13.0603087	Birmingham	UK	Delhi	R	S	S	S	R	S	S	S
12.0612520	Birmingham	UK	lineage4	S	S	S	S	S	S	S	S
13.0613544	Birmingham	UK	Delhi	S	S	S	S	U	S	S	S
13.0604897	Birmingham	UK	EAI	S	S	S	S	S	S	U	S
15.0601228	Birmingham	UK	Haarlem	S	S	S	S	S	S	S	S
17.0601233	Birmingham	UK	Haarlem	n/a	n/a	n/a	S	S	S	S	S
15.0604955	Birmingham	UK	Cameroon	S	S	S	S	S	S	S	S
13.0609003	Birmingham	UK	EAI	S	S	S	S	S	S	S	S
16.0617363	Birmingham	UK	lineage4	S	S	S	S	S	S	S	S
16.0618282	Birmingham	UK	EAI	S	S	S	S	S	F	S	S
14.0609509	Birmingham	UK	EAI	S	S	S	S	S	S	S	S
14.0600301	Birmingham	UK	Tur	S	S	S	S	S	S	S	S
14.0600068	Birmingham	UK	Uganda	S	S	S	S	S	S	S	S
16.0601666	Birmingham	UK	lineage4	R	R	R	n/a	R	R	R	F
16.0601133	Birmingham	UK	Delhi	S	S	S	S	S	S	U	S
16.0605889	Birmingham	UK	Haarlem	S	S	S	S	S	S	S	S
16.0601963	Birmingham	UK	Haarlem	S	S	S	S	S	S	S	S
13.0613206	Birmingham	UK	lineage4	S	S	S	S	S	S	S	S
16.0601901	Birmingham	UK	Delhi	S	S	S	S	S	F	S	S
13.0603154	Birmingham	UK	LAM	S	S	S	S	S	S	S	S
16.0614420	Birmingham	UK	Delhi	S	S	S	S	S	S	S	S
12.0611897	Birmingham	UK	Delhi	S	S	S	S	S	S	S	S
16.0618621	Birmingham	UK	Delhi	S	S	S	n/a	S	S	U	S
12.0613195	Birmingham	UK	Delhi	S	S	S	S	S	S	S	S
15.0601963	Birmingham	UK	Delhi	S	S	S	S	S	S	S	S
14.0600815	Birmingham	UK	lineage4	R	S	S	S	R	S	S	S
14.0600174	Birmingham	UK	EAI	S	S	S	S	S	S	S	S
14.0600303	Birmingham	UK	Haarlem	S	S	S	S	S	S	S	S
16.0611291	Birmingham	UK	lineage4	S	S	S	S	S	S	S	S
14.0604808	Birmingham	UK	Delhi	S	S	S	S	S	S	S	S
14.0613802	Birmingham	UK	lineage4	S	S	S	S	S	S	S	S
16.0608644	Birmingham	UK	LAM	S	S	S	S	S	S	S	S
14.0606215	Birmingham	UK		S	S	S	S	S	S	S	S
12.0607547	Birmingham	UK	Delhi	S	S	S	S	S	S	S	S
13.0613111	Birmingham	UK	Delhi	S	S	S	S	S	S	S	S
17.0601252	Birmingham	UK	Delhi	S	S	S	S	S	S	S	S
16.0601231	Birmingham	UK	Beijing	S	S	S	S	S	S	S	S
13.0609458	Birmingham	UK	LAM	S	S	S	S	S	S	S	S
15.0605349	Birmingham	UK	EAI	R	n/a	S	S	R	R	U	S
17.0601220	Birmingham	UK	Delhi	S	S	S	S	S	S	S	S
12.0611179	Birmingham	UK	Beijing	S	S	S	S	S	S	S	S
15.0601910	Birmingham	UK	Delhi	S	S	S	S	S	S	U	S
16.0607979	Birmingham	UK	Delhi	S	S	S	S	S	S	S	S
16.0607117	Birmingham	UK	Delhi	S	S	S	S	S	S	F	S
14.0600708	Birmingham	UK	Delhi	S	S	S	S	S	S	S	S
13.0610493	Birmingham	UK	Delhi	S	S	S	S	S	S	S	S
14.0616146	Birmingham	UK	Beijing	R	R	R	n/a	R	R	R	R
15.0607243	Birmingham	UK	Delhi	S	S	S	S	S	S	S	S
12.0927951	Birmingham	UK	LAM	S	S	S	S	S	S	S	S
15.0607089	Birmingham	UK	EAI	S	S	S	S	S	S	S	S
14.0600736	Birmingham	UK	Cameroon	S	S	S	S	S	S	S	S
16.0601247	Birmingham	UK	Delhi	S	S	S	S	S	S	S	S
16.0613570	Birmingham	UK	Haarlem	S	S	S	S	S	S	S	S
15.0607969	Birmingham	UK	M. bovis	S	S	S	R	S	S	S	R
14.0609773	Birmingham	UK	Delhi	S	S	S	S	S	S	S	S
14.0608174	Birmingham	UK	Delhi	S	S	S	S	S	R	S	S
11.0614843	Birmingham	UK	EAI	S	S	S	n/a	S	S	S	S
14.0600893	Birmingham	UK	EAI	S	S	S	S	S	S	S	S
14.0609957	Birmingham	UK	Delhi	S	S	S	S	S	S	S	S
15.0603618	Birmingham	UK	Haarlem	S	S	S	S	S	S	U	S
15.0607522	Birmingham	UK	EAI	S	S	S	S	S	F	S	S
14.0610264	Birmingham	UK	Delhi	S	S	S	S	S	S	S	S
12.0615221	Birmingham	UK	EAI	S	S	S	S	F	S	S	S

16.0601784	Birmingham	UK	Beijing	S	S	S	S	S	R	S	S
16.0601321	Birmingham	UK	EAI	S	S	S	n/a	S	S	U	S
12.0612550	Birmingham	UK	LAM	S	S	S	S	S	S	S	S
14.0602933	Birmingham	UK	Delhi	S	S	S	S	S	S	U	S
09.0605986	Birmingham	UK	Haarlem	R	S	S	S	R	S	S	R
16.0601324	Birmingham	UK	Haarlem	S	S	S	S	U	S	S	S
15.0607597	Birmingham	UK	lineage4	S	S	S	S	S	S	S	S
13.0604526	Birmingham	UK	Delhi	S	S	S	S	S	S	S	S
12.0612533	Birmingham	UK	Beijing	R	R	R	R	R	R	R	R
13.0606727	Birmingham	UK	Delhi	S	S	S	S	S	S	S	S
15.0607097	Birmingham	UK	Delhi	S	S	S	S	S	S	S	S
14.0609052	Birmingham	UK	Delhi	S	S	S	S	S	S	S	S
16.0601135	Birmingham	UK	Delhi	S	S	S	S	S	S	S	S
15.0601163	Birmingham	UK	lineage4	R	S	S	S	R	S	S	S
16.0618509	Birmingham	UK	LAM	S	S	S	S	S	S	S	S
13.0613182	Birmingham	UK	X-type	S	n/a	S	S	S	R	S	S
15.0607231	Birmingham	UK	EAI	S	S	S	S	S	S	S	R
14.0609001	Birmingham	UK	LAM	S	S	S	S	S	S	S	S
14.0609598	Birmingham	UK	Delhi	S	S	S	S	S	S	S	S
17.0601129	Birmingham	UK	lineage4	S	S	S	n/a	S	S	S	S
16.0607911	Birmingham	UK	lineage4	S	S	S	S	S	S	S	S
16.0601641	Birmingham	UK	EAI	S	S	S	S	S	S	S	S
13.0608328	Birmingham	UK	Delhi	S	S	S	S	S	S	S	S
11.0609727	Birmingham	UK	Beijing	S	S	S	S	S	S	S	S
12.0612488	Birmingham	UK	Delhi	S	S	S	S	S	S	S	S
14.0601200	Birmingham	UK	Delhi	S	S	S	S	S	S	S	S
16.0608008	Birmingham	UK	Delhi	S	S	S	S	S	S	S	S
09.0601062	Birmingham	UK	Delhi	S	S	S	S	S	S	S	S
16.0607547	Birmingham	UK	Delhi	S	S	S	S	U	S	U	F
13.0605698	Birmingham	UK	lineage4	S	S	S	S	S	S	S	S
09.0603199	Birmingham	UK	Delhi	S	S	S	S	S	S	S	S
15.0613331	Birmingham	UK	X-type	S	S	S	S	S	S	S	S
14.0600949	Birmingham	UK	lineage4	R	S	S	S	R	S	S	S
15.0601524	Birmingham	UK	Haarlem	S	S	S	S	S	S	S	S
13.0616904	Birmingham	UK	Delhi	S	S	S	S	S	S	S	S
13.0603234	Birmingham	UK	S-type	S	S	S	S	S	S	S	S
13.0604055	Birmingham	UK	M. bovis	n/a	n/a	n/a	R	U	S	S	R
15.0602266	Birmingham	UK	lineage4	S	S	S	S	S	S	S	S
13.0609244	Birmingham	UK	LAM	S	S	S	S	S	S	S	S
14.0609411	Birmingham	UK	LAM	S	S	S	S	S	S	S	S
14.0609862	Birmingham	UK	EAI	S	S	S	S	S	S	S	S
12.0606001	Birmingham	UK	Delhi	S	S	S	S	S	S	S	S
16.0607865	Birmingham	UK	lineage4	S	S	S	S	S	S	S	S
14.0600248	Birmingham	UK	X-type	S	S	S	S	S	S	S	S
14.0609898	Birmingham	UK	LAM	R	R	S	S	R	F	R	R
16.0601510	Birmingham	UK	EAI	S	S	S	n/a	S	S	S	S
15.0607685	Birmingham	UK	EAI	R	S	S	S	R	S	U	S
13.0618415	Birmingham	UK	Delhi	S	S	S	S	S	S	S	S
12.0612052	Birmingham	UK	lineage4	S	S	S	S	S	S	S	S
15.0605917	Birmingham	UK	Beijing	S	S	S	S	S	S	S	S
14.0600224	Birmingham	UK	Haarlem	S	S	S	S	S	S	S	S
13.0601559	Birmingham	UK	lineage4	S	S	S	S	S	S	S	S
15.0607634	Birmingham	UK	LAM	R	R	n/a	n/a	R	R	R	R
14.0600804	Birmingham	UK	EAI	S	S	S	S	S	S	S	S
14.0616008	Birmingham	UK	Delhi	n/a	n/a	n/a	S	S	S	S	S
14.0600701	Birmingham	UK	Delhi	S	S	S	S	S	S	S	S
14.0600969	Birmingham	UK		S	S	S	S	S	S	S	S
14.0609550	Birmingham	UK	S-type	S	S	S	S	S	S	S	S
16.0604070	Birmingham	UK	lineage4	S	S	S	S	S	F	S	S
15.0607144	Birmingham	UK	Haarlem	R	S	S	S	R	S	S	S
15.0607743	Birmingham	UK	LAM	R	R	R	R	R	R	R	R
13.0609500	Birmingham	UK	LAM	S	S	S	S	S	U	S	S
16.0601249	Birmingham	UK	Ural	S	S	S	S	S	S	S	S
13.0611898	Birmingham	UK	Delhi	S	S	S	S	S	S	S	S
15.0613468	Birmingham	UK	Haarlem	R	S	S	S	R	S	R	S
16.0607634	Birmingham	UK	Delhi	S	S	S	S	S	S	S	S
15.0610065	Birmingham	UK	Haarlem	S	S	S	S	S	S	S	S
13.0610490	Birmingham	UK	S-type	S	S	S	S	S	S	S	S
15.0607583	Birmingham	UK	EAI	R	S	S	S	U	S	S	S
15.0602378	Birmingham	UK	Delhi	S	S	S	S	S	S	S	S
16.0607713	Birmingham	UK	Haarlem	S	S	S	S	S	S	S	S
14.0607272	Birmingham	UK	Delhi	S	S	S	S	S	S	S	S
12.0617750	Birmingham	UK	lineage4	S	S	S	S	S	S	S	S
16.0606627	Birmingham	UK	Delhi	S	S	S	S	S	S	S	S
15.0607519	Birmingham	UK	lineage4	S	S	S	S	S	S	S	S
16.0607204	Birmingham	UK	X-type	S	S	S	S	S	S	S	S
16.0607413	Birmingham	UK	Haarlem	S	S	S	S	S	S	S	S
16.0601283	Birmingham	UK	Delhi	S	S	S	S	S	S	S	S
13.0603125	Birmingham	UK	Tur	S	S	S	S	S	S	S	S
13.0603882	Birmingham	UK	Cameroon	S	S	S	S	S	S	S	S
13.0607382	Birmingham	UK	EAI	S	S	S	S	S	S	S	S
14.0609511	Birmingham	UK	Delhi	S	S	S	S	S	S	S	S
16.0602777	Birmingham	UK		S	S	S	S	S	U	S	S
14.0600695	Birmingham	UK	Delhi	S	S	S	S	S	S	S	S
15.0613121	Birmingham	UK	lineage4	S	S	S	S	S	S	U	S
16.0610387	Birmingham	UK	Beijing	R	R	n/a	n/a	R	R	S	R
13.0616398	Birmingham	UK	EAI	S	S	S	S	S	S	S	S
16.0601253	Birmingham	UK	S-type	S	S	S	S	S	S	S	S
15.0601189	Birmingham	UK	Beijing	R	R	R	S	R	R	R	R
14.0600740	Birmingham	UK	Delhi	S	S	S	S	S	S	S	S
09.0605864	Birmingham	UK	X-type	S	S	S	S	S	S	S	S
13.0613443	Birmingham	UK	Delhi	S	S	S	S	S	S	S	S
13.0600311	Birmingham	UK	Delhi	S	S	S	S	S	S	S	S
12.0927696	Birmingham	UK	EAI	S	S	S	S	S	S	S	S
15.0613149	Birmingham	UK	Haarlem	R	S	S	S	R	S	S	S
13.0603101	Birmingham	UK	lineage4	S	S	S	S	S	S	U	S
11.0613271	Birmingham	UK	X-type	S	S	S	S	S	F	F	S
13.0608308	Birmingham	UK	Delhi	S	S	S	S	S	S	S	S
16.0618527	Birmingham	UK	lineage4	S	S	S	S	S	S	S	S
14.0609307	Birmingham	UK	lineage4	S	S	S	S	F	F	S	S
15.0613194	Birmingham	UK	Delhi	S	S	S	S	S	F	S	S
16.0601323	Birmingham	UK	Delhi	S	S	S	S	S	S	S	S
14.0600438	Birmingham	UK	EAI	S	S	S	S	S	S	S	S
13.0613582	Birmingham	UK	lineage4	S	S	S	S	S	S	S	S
15.0601764	Birmingham	UK	LAM	S	S	S	S	S	S	S	S
13.0611008	Birmingham	UK	Haarlem	S	S	S	S	S	S	S	S
16.0607551	Birmingham	UK	Beijing	S	S	S	S	S	S	S	S
12.0604241	Birmingham	UK	Delhi	S	S	S	S	S	S	S	S
15.0601634	Birmingham	UK	Delhi	S	S	S	S	S	S	S	S
13.0614099	Birmingham	UK	lineage4	S	S	S	S	S	S	S	S
12.0616629	Birmingham	UK	Delhi	S	S	S	S	S	S	S	S
15.0614758	Birmingham	UK	Delhi	S	S	S	S	S	S	S	S
15.0607730	Birmingham	UK	Delhi	R	R	S	S	U	R	S	S
15.0601197	Birmingham	UK	Delhi	S	S	S	S	S	S	S	S
13.0607969	Birmingham	UK	lineage4	S	S	S	S	S	S	S	S
16.0618191	Birmingham	UK	lineage4	S	S	S	S	S	S	S	S

14.0600850	Birmingham	UK	X-type	S	S	S	S	S	S	S	S
15.0601400	Birmingham	UK	Delhi	S	S	S	S	S	S	S	S
16.0607479	Birmingham	UK	Tur	S	S	S	S	S	S	S	S
15.0609816	Birmingham	UK	lineage4	S	S	S	S	S	S	S	S
16.0601274	Birmingham	UK	lineage4	S	S	S	S	S	S	S	S
14.0609234	Birmingham	UK	lineage4	S	S	S	S	S	U	S	S
10.0600320	Birmingham	UK	Beijing	R	R	R	S	R	R	R	R
13.0602718	Birmingham	UK	lineage4	S	S	S	S	S	S	S	S
14.0609908	Birmingham	UK	LAM	S	S	S	S	S	S	S	S
16.0607316	Birmingham	UK	Delhi	S	S	S	S	S	S	S	S
16.0607451	Birmingham	UK	Delhi	S	S	S	S	S	S	S	S
13.0606728	Birmingham	UK	EAI	S	S	S	S	S	S	S	S
12.0617770	Birmingham	UK	EAI	S	S	S	S	S	S	S	S
15.0613542	Birmingham	UK	lineage4	S	S	S	S	S	S	S	S
14.0600543	Birmingham	UK	Delhi	S	S	S	S	S	S	S	S
12.0617127	Birmingham	UK	Beijing	S	S	S	S	S	S	S	S
13.0608935	Birmingham	UK	Delhi	S	S	S	S	S	S	S	S
13.0613537	Birmingham	UK	Delhi	S	S	S	S	S	U	S	S
12.0611237	Birmingham	UK	LAM	S	S	S	S	U	S	S	S
13.0607141	Birmingham	UK	lineage4	S	S	S	S	S	S	S	S
16.0601116	Birmingham	UK	LAM	n/a	n/a	n/a	S	S	S	S	S
12.0610131	Birmingham	UK	Delhi	S	S	S	S	S	S	S	S
12.0617788	Birmingham	UK	Delhi	S	S	S	S	S	S	S	S
15.0613383	Birmingham	UK	Delhi	S	S	S	S	S	S	S	S
16.0607463	Birmingham	UK	lineage4	S	S	S	S	S	S	U	S
10.0603200	Birmingham	UK	Delhi	S	S	S	S	S	S	S	S
16.0617387	Birmingham	UK	Beijing	S	S	S	S	S	S	U	S
15.0607884	Birmingham	UK	lineage4	S	S	S	S	S	S	S	S
13.0612340	Birmingham	UK	Delhi	R	S	S	S	R	S	S	S
13.0611032	Birmingham	UK	lineage4	S	S	S	S	S	S	S	S
16.0617174	Birmingham	UK	Delhi	S	S	S	S	S	S	S	S
13.0606636	Birmingham	UK	EAI	S	S	S	S	S	S	S	S
16.0617626	Birmingham	UK	Beijing	S	S	S	S	S	S	S	S
16.0617180	Birmingham	UK	S-type	S	S	S	S	S	S	S	S
15.0614985	Birmingham	UK	Delhi	S	S	S	S	S	S	S	S
16.0607050	Birmingham	UK	Delhi	S	S	S	S	S	F	S	S
15.0601004	Birmingham	UK	X-type	S	S	S	S	S	S	S	S
13.0611039	Birmingham	UK	Delhi	S	S	S	S	S	S	S	S
12.0613160	Birmingham	UK	Haarlem	S	S	S	S	S	S	S	S
13.0603127	Birmingham	UK	X-type	S	S	S	S	S	S	S	S
09.0600593	Birmingham	UK	Haarlem	S	S	S	S	S	S	S	S
16.0602416	Birmingham	UK	Delhi	S	S	n/a	S	S	S	S	S
15.0601229	Birmingham	UK	Delhi	S	S	S	S	S	S	U	S
15.0601900	Birmingham	UK	lineage4	S	S	S	S	S	S	S	S
15.0608493	Birmingham	UK	lineage4	S	S	S	S	S	S	S	S
16.0618565	Birmingham	UK	Delhi	S	S	S	S	S	S	S	S
16.0608724	Birmingham	UK	lineage4	S	S	S	S	S	S	S	S
15.0613205	Birmingham	UK	EAI	S	S	S	S	S	S	U	S
13.0609448	Birmingham	UK	Delhi	S	S	S	S	S	S	S	S
09.0605405	Birmingham	UK	S-type	R	S	S	S	R	S	S	S
12.0927952	Birmingham	UK	lineage4	S	S	S	S	S	S	S	S
13.0616902	Birmingham	UK	Delhi	S	S	S	S	S	S	S	S
12.0614205	Birmingham	UK	Haarlem	S	S	S	S	S	S	S	S
15.0613250	Birmingham	UK	Delhi	S	S	S	S	S	S	S	S
15.0607920	Birmingham	UK	Haarlem	R	R	R	n/a	R	R	R	R
09.0600141	Birmingham	UK	Delhi	S	S	S	S	S	S	S	S
16.0618578	Birmingham	UK	Cameroon	R	S	S	S	R	S	S	S
09.0602405	Birmingham	UK	LAM	S	S	S	S	S	S	S	S
11.0614093	Birmingham	UK	Delhi	S	S	S	S	S	S	S	S
15.0601787	Birmingham	UK	Beijing	S	S	S	S	S	S	S	S
15.0607650	Birmingham	UK	Beijing	S	S	S	S	S	S	S	S
15.0609973	Birmingham	UK	LAM	S	S	S	S	S	S	S	S
14.0609405	Birmingham	UK	EAI	S	S	S	S	S	S	S	S
15.0603619	Birmingham	UK		S	S	S	S	S	S	U	S
16.0601275	Birmingham	UK	Delhi	S	S	S	S	S	S	S	S
16.0607548	Birmingham	UK	lineage4	S	S	S	S	S	F	S	S
16.0618259	Birmingham	UK	Delhi	S	S	S	S	S	S	S	S
13.0609885	Birmingham	UK	EAI	S	S	S	S	S	S	S	S
13.0613543	Birmingham	UK	Haarlem	S	S	S	S	S	S	S	S
12.0615299	Birmingham	UK	Delhi	S	S	S	S	F	S	S	S
16.0607510	Birmingham	UK	lineage4	S	S	S	S	S	S	S	S
13.0613549	Birmingham	UK	Haarlem	S	S	S	S	S	S	S	S
13.0613389	Birmingham	UK	Haarlem	S	S	S	S	S	S	S	S
15.0601254	Birmingham	UK	lineage4	S	S	S	S	S	S	S	S
15.0613171	Birmingham	UK	Beijing	S	S	S	S	S	S	S	S
13.0605163	Birmingham	UK	EAI	S	S	S	S	S	S	S	S
15.0607506	Birmingham	UK	LAM	R	R	n/a	R	R	R	R	R
12.0615391	Birmingham	UK	S-type	S	S	S	S	S	S	S	S
13.0609469	Birmingham	UK	Beijing	S	S	S	S	S	S	S	S
15.0601368	Birmingham	UK	Beijing	S	S	S	S	S	S	S	S
15.0601859	Birmingham	UK	lineage4	S	S	S	S	U	S	U	S
13.0613271	Birmingham	UK	lineage4	S	S	S	S	S	S	S	S
13.0610503	Birmingham	UK	Delhi	S	S	S	S	S	S	S	S
14.0611573	Birmingham	UK		S	S	S	S	S	S	S	S
13.0608000	Birmingham	UK	Delhi	S	S	S	S	S	S	S	S
15.0607186	Birmingham	UK	LAM	S	S	S	S	S	S	S	S
16.0601737	Birmingham	UK	Delhi	S	S	S	S	S	S	S	S
15.0607492	Birmingham	UK	X-type	S	S	S	S	S	S	S	S
15.0607284	Birmingham	UK	EAI	S	S	S	S	S	U	S	S
15.0601223	Birmingham	UK	Delhi	S	S	S	S	S	S	S	S
12.0617796	Birmingham	UK	EAI	S	S	S	S	U	S	S	S
12.0602344	Birmingham	UK	X-type	S	S	S	S	S	R	R	R
15.0613522	Birmingham	UK	X-type	S	S	S	S	S	S	S	S
09.0600736	Birmingham	UK	EAI	S	S	S	S	S	S	S	S
14.0613993	Birmingham	UK	LAM	S	S	S	S	S	S	S	S
12.0617785	Birmingham	UK	lineage4	S	S	S	S	S	S	S	S
16.0618336	Birmingham	UK	EAI	S	S	S	S	S	S	S	S
14.0606563	Birmingham	UK	Delhi	S	S	S	S	S	S	S	S
16.0607864	Birmingham	UK	Beijing	R	R	R	S	R	R	R	S
15.0601215	Birmingham	UK	Beijing	S	S	S	S	S	S	S	S
12.0611861	Birmingham	UK	Delhi	S	S	S	S	S	S	S	S
16.0607817	Birmingham	UK	S-type	S	S	S	S	S	S	S	S
15.0607727	Birmingham	UK	Delhi	R	R	S	S	U	R	S	S
16.0617122	Birmingham	UK	LAM	S	S	S	S	S	S	S	S
12.0616911	Birmingham	UK	Tur	S	S	S	S	S	S	S	S
14.0611909	Birmingham	UK	X-type	R	n/a	S	S	R	R	R	R
13.0615143	Birmingham	UK		S	S	S	S	S	S	S	S
15.0615037	Birmingham	UK	Delhi	S	S	S	S	S	S	S	S
17.0601243	Birmingham	UK	lineage4	S	S	S	n/a	S	S	S	S
14.0600165	Birmingham	UK	Haarlem	S	S	S	S	S	S	S	S
16.0607518	Birmingham	UK	Delhi	R	S	S	S	R	S	S	F
12.0606337	Birmingham	UK	Delhi	S	S	S	S	S	S	S	S
12.0603192	Birmingham	UK	Beijing	S	S	S	S	S	S	U	S
16.0618556	Birmingham	UK	Delhi	n/a	S	S	S	R	S	S	S
14.0610803	Birmingham	UK	EAI	S	S	S	S	S	S	S	S
15.0613452	Birmingham	UK		S	S	S	S	S	S	S	S

13.0611317	Birmingham	UK	lineage4	S	S	S	S	S	S	S	S
13.0616015	Birmingham	UK	lineage4	S	S	S	S	S	S	S	S
15.0607295	Birmingham	UK	Beijing	S	S	S	S	S	S	S	S
15.0601812	Birmingham	UK	lineage4	S	S	S	S	S	S	S	S
16.0608345	Birmingham	UK	lineage4	S	S	S	S	S	S	S	S
16.0601600	Birmingham	UK	Haarlem	S	S	S	S	S	S	S	S
16.0601847	Birmingham	UK	lineage4	S	S	S	S	S	S	S	S
16.0604944	Birmingham	UK	Delhi	S	S	S	S	S	S	S	S
14.0609414	Birmingham	UK	Delhi	S	S	S	S	S	S	U	S
12.0616374	Birmingham	UK	Delhi	S	S	S	S	S	S	S	S
15.0611156	Birmingham	UK	Delhi	S	S	S	n/a	S	S	S	R
16.0601050	Birmingham	UK	Delhi	S	S	S	S	S	F	S	S
15.0601023	Birmingham	UK	lineage4	S	S	S	S	S	S	S	S
17.0601096	Birmingham	UK	Tur	S	S	n/a	S	S	S	S	S
15.0613074	Birmingham	UK	Delhi	S	S	S	S	S	S	S	S
11.0613996	Birmingham	UK	EAI	S	S	S	S	S	S	S	S
14.0600349	Birmingham	UK	Delhi	S	S	S	S	S	S	S	S
14.0600196	Birmingham	UK	lineage4	S	S	S	S	S	S	S	R
13.0605835	Birmingham	UK	EAI	S	S	S	S	S	S	S	U
14.0609829	Birmingham	UK	Delhi	S	S	S	S	S	S	S	S
12.0614690	Birmingham	UK	Tur	S	S	S	S	S	S	S	S
14.0609201	Birmingham	UK	EAI	S	S	S	S	S	S	S	S
15.0607453	Birmingham	UK	Delhi	S	S	S	S	S	S	S	S
15.0613390	Birmingham	UK	Tur	S	S	S	S	S	S	S	S
14.0609551	Birmingham	UK	Ural	S	S	S	S	S	S	S	S
14.0603272	Birmingham	UK	lineage4	S	S	S	S	S	S	S	S
14.0609979	Birmingham	UK	S-type	S	S	S	S	S	S	S	S
13.0615983	Birmingham	UK	LAM	S	S	S	S	S	S	S	S
16.0605745	Birmingham	UK	Beijing	S	S	S	S	S	S	S	S
15.0613343	Birmingham	UK	lineage4	S	S	S	S	S	S	S	S
14.0608723	Birmingham	UK	Beijing	R	R	S	S	R	F	R	S
13.0611321	Birmingham	UK	Delhi	S	S	S	S	S	S	S	S
16.0604453	Birmingham	UK	Delhi	S	S	S	S	S	S	S	S
15.0610473	Birmingham	UK	Delhi	R	S	S	S	R	S	S	S
15.0607420	Birmingham	UK	LAM	R	S	S	S	R	S	S	F
13.0608772	Birmingham	UK	lineage4	S	S	S	S	S	S	S	S
16.0601670	Birmingham	UK	Beijing	S	S	S	S	S	S	S	S
14.0609094	Birmingham	UK	Beijing	S	S	S	S	S	U	S	S
12.0609705	Birmingham	UK	Beijing	S	S	S	S	S	S	S	S
16.0618145	Birmingham	UK	Beijing	R	R	R	R	R	R	R	R
16.0601833	Birmingham	UK	lineage4	S	S	S	S	S	S	S	S
15.0607291	Birmingham	UK	lineage4	S	S	S	S	S	S	S	S
12.0613608	Birmingham	UK	Delhi	S	S	S	S	S	S	S	S
13.0613618	Birmingham	UK	Delhi	S	S	S	S	S	S	S	S
13.0615147	Birmingham	UK	Ghana	R	S	S	S	R	S	S	S
16.0607397	Birmingham	UK	LAM	R	S	S	S	R	S	S	S
12.0612552	Birmingham	UK	Delhi	S	S	S	S	S	S	S	S
13.0601363	Birmingham	UK	Delhi	R	S	S	S	R	S	U	S
16.0617495	Birmingham	UK	Delhi	S	S	S	S	S	S	S	S
12.0617739	Birmingham	UK	Delhi	S	S	S	S	S	S	S	S
13.0613282	Birmingham	UK	Delhi	S	S	S	S	S	S	S	S
14.0607879	Birmingham	UK	Delhi	R	R	S	S	U	R	S	S
16.0606862	Birmingham	UK	EAI	n/a	S	S	S	R	S	S	S
12.0616143	Birmingham	UK	Delhi	S	S	S	S	U	S	S	S
15.0613065	Birmingham	UK	S-type	S	S	S	S	S	S	S	S
14.0608517	Birmingham	UK	X-type	R	S	S	R	R	F	R	R
09.0601059	Birmingham	UK	Delhi	S	S	S	S	S	S	S	S
14.0609573	Birmingham	UK	Delhi	S	S	S	S	S	S	S	S
16.0617396	Birmingham	UK	Delhi	S	S	S	S	S	S	S	S
16.0601522	Birmingham	UK	Beijing	S	S	S	S	S	S	U	S
16.0601352	Birmingham	UK	lineage4	S	S	S	S	S	S	S	S
15.0607369	Birmingham	UK	Delhi	S	S	S	S	S	S	S	S
15.0601696	Birmingham	UK	Delhi	S	S	S	S	S	S	U	S
12.0611800	Birmingham	UK	LAM	S	R	S	S	S	F	S	S
15.0607734	Birmingham	UK	Haarlem	S	S	S	S	S	S	S	S
14.0600216	Birmingham	UK	lineage4	S	S	S	S	S	S	U	S
16.0601204	Birmingham	UK	Beijing	S	S	S	S	U	S	S	S
13.0604551	Birmingham	UK	Delhi	S	S	S	S	S	S	S	S
16.0607993	Birmingham	UK	lineage4	S	S	S	S	S	S	S	S
16.0618382	Birmingham	UK	Haarlem	S	S	S	S	S	S	S	S
13.0603076	Birmingham	UK	LAM	S	S	S	S	S	S	S	S
12.0611898	Birmingham	UK	Beijing	S	S	S	S	S	S	S	S
09.0602071	Birmingham	UK	EAI	R	S	S	S	R	S	S	S
16.0617077	Birmingham	UK	Haarlem	S	S	S	n/a	S	S	U	S
12.0611194	Birmingham	UK	Delhi	S	S	S	S	S	U	S	S
15.0601543	Birmingham	UK	Delhi	S	S	S	S	S	S	S	S
13.0604540	Birmingham	UK	Beijing	S	S	S	S	S	S	S	S
13.0603888	Birmingham	UK	Delhi	S	S	S	S	S	S	S	S
12.0617278	Birmingham	UK	Haarlem	S	S	S	S	S	S	F	S
15.0600819	Birmingham	UK	lineage4	R	S	S	S	R	S	S	S
13.0605708	Birmingham	UK	Haarlem	S	S	S	S	S	S	S	S
13.0602831	Birmingham	UK	Cameroon	S	S	S	S	S	S	S	S
16.0618318	Birmingham	UK	EAI	S	S	S	S	S	S	S	S
13.0611906	Birmingham	UK	Haarlem	S	S	S	S	S	S	S	S
15.0607709	Birmingham	UK	lineage4	S	S	S	S	S	S	S	S
12.0613611	Birmingham	UK	EAI	S	S	S	S	S	S	S	S
12.0616608	Birmingham	UK	Delhi	S	S	S	S	S	S	S	S
13.0607663	Birmingham	UK	EAI	S	S	S	S	S	S	S	S
15.0607749	Birmingham	UK	lineage4	S	S	S	S	S	S	S	S
11.0606369	Birmingham	UK	Delhi	S	S	S	S	S	S	S	S
14.0600750	Birmingham	UK	Delhi	S	S	S	S	S	S	S	S
14.0616213	Birmingham	UK	Delhi	S	S	S	S	S	S	U	S
13.0610480	Birmingham	UK	Delhi	R	S	S	S	R	S	S	S
16.0607818	Birmingham	UK	Delhi	S	S	S	S	S	S	S	S
14.0616166	Birmingham	UK	EAI	S	S	S	S	S	S	S	S
15.0613153	Birmingham	UK	Delhi	S	S	S	S	S	S	S	S
11.0602930	Birmingham	UK	lineage4	n/a	S	S	S	R	S	S	S
14.0609098	Birmingham	UK	lineage4	S	S	S	S	S	S	S	S
13.0601984	Birmingham	UK	lineage4	S	S	S	S	S	S	S	S
13.0613560	Birmingham	UK	Delhi	S	S	S	S	S	S	S	S
12.0611850	Birmingham	UK	lineage4	S	S	S	S	S	S	S	S
15.0601293	Birmingham	UK	Delhi	S	S	S	S	S	S	S	S
12.0611238	Birmingham	UK	Haarlem	S	S	S	S	F	S	U	S
15.0613143	Birmingham	UK	lineage4	S	S	S	S	S	S	S	S
16.0601791	Birmingham	UK		S	S	S	S	S	S	S	S
13.0607359	Birmingham	UK	Delhi	S	S	S	S	S	S	S	S
14.0600703	Birmingham	UK	EAI	S	S	S	S	S	S	S	S
16.0607143	Birmingham	UK	S-type	S	S	S	S	S	S	S	S
16.0602160	Birmingham	UK	LAM	S	S	S	S	S	S	S	S
13.0616370	Birmingham	UK	Cameroon	S	S	S	S	S	S	S	S
12.0927851	Birmingham	UK	Haarlem	S	S	S	S	S	S	S	S
13.0602078	Birmingham	UK	lineage4	S	S	S	S	S	S	S	S
14.0600981	Birmingham	UK		S	S	S	S	S	S	S	S
15.0607976	Birmingham	UK	LAM	S	S	S	S	S	S	S	F
15.0601054	Birmingham	UK		R	S	S	S	R	S	S	S
13.0601215	Birmingham	UK	Delhi	S	S	S	S	S	S	S	S

16.0601750	Birmingham	UK	Delhi	S	S	S	S	S	S	S	S
13.0610930	Birmingham	UK	Haarlem	S	S	S	S	S	S	S	S
15.0607794	Birmingham	UK	Beijing	S	S	S	S	S	R	S	S
15.0613049	Birmingham	UK	Delhi	S	S	S	S	S	U	S	S
14.0612496	Birmingham	UK	LAM	S	S	S	S	S	S	S	S
16.0607380	Birmingham	UK	lineage4	S	S	S	S	S	S	S	S
12.0611992	Birmingham	UK	Delhi	S	S	S	S	S	S	U	S
16.0607611	Birmingham	UK	Delhi	S	S	S	S	S	S	S	S
14.0609320	Birmingham	UK	X-type	S	S	S	S	S	S	S	F
15.0601515	Birmingham	UK	EAI	S	S	S	S	U	S	S	S
15.0606997	Birmingham	UK	Delhi	S	S	S	S	S	S	S	S
14.0600155	Birmingham	UK	Haarlem	S	S	S	S	S	S	U	S
15.0607805	Birmingham	UK	Delhi	S	S	S	S	S	S	S	S
13.0604890	Birmingham	UK	lineage4	S	S	S	S	S	S	S	S
16.0601189	Birmingham	UK	EAI	S	S	S	S	S	S	S	S
15.0607874	Birmingham	UK	Delhi	R	S	S	S	R	S	S	S
15.0602613	Birmingham	UK	Ural	R	S	S	n/a	R	S	S	S
13.0600608	Birmingham	UK	Ural	S	S	S	S	S	S	S	S
12.0615291	Birmingham	UK	Delhi	S	S	S	S	F	S	S	S
12.0610314	Birmingham	UK	Delhi	S	S	S	S	S	S	S	S
16.0607367	Birmingham	UK	Haarlem	S	S	S	S	S	S	S	S
17.0600112	Birmingham	UK	Delhi	S	S	S	S	S	S	S	S
15.0607083	Birmingham	UK	Beijing	S	S	S	S	S	S	S	S
16.0605632	Birmingham	UK	Delhi	S	S	S	S	S	S	S	S
13.0603151	Birmingham	UK	Delhi	S	S	S	S	F	S	S	S
15.0607438	Birmingham	UK	Delhi	S	S	S	S	S	S	S	S
12.0611591	Birmingham	UK	Haarlem	S	S	S	S	S	S	U	S
15.0607503	Birmingham	UK	lineage4	S	S	S	S	S	S	U	S
15.0607074	Birmingham	UK	Delhi	S	S	S	S	S	S	S	S
15.0607353	Birmingham	UK	Delhi	S	S	S	S	S	S	S	S
16.0611790	Birmingham	UK	X-type	S	S	S	S	S	F	S	S
14.0600986	Birmingham	UK	X-type	S	S	S	S	S	S	S	S
16.0607989	Birmingham	UK	Delhi	R	R	S	S	R	R	S	S
16.0601515	Birmingham	UK	lineage4	S	S	S	S	S	S	S	S
13.0600255	Birmingham	UK	Cameroon	S	S	S	S	S	S	S	S
16.0601606	Birmingham	UK	S-type	S	S	S	S	S	S	S	S
13.0612462	Birmingham	UK	LAM	S	S	S	S	S	S	S	S
13.0600572	Birmingham	UK	Delhi	S	S	S	S	S	S	S	S
15.0613389	Birmingham	UK	Haarlem	S	S	S	S	S	S	U	S
15.0613333	Birmingham	UK	lineage4	S	S	S	S	S	S	U	S
14.0603754	Birmingham	UK	Delhi	S	S	S	S	S	S	S	S
13.0602587	Birmingham	UK	Delhi	S	S	S	S	S	S	U	S
15.0610391	Birmingham	UK	Delhi	R	S	S	S	R	S	S	S
16.0618387	Birmingham	UK	Delhi	S	S	S	S	S	S	S	S
16.0607888	Birmingham	UK	LAM	S	S	S	S	S	S	S	S
13.0604513	Birmingham	UK	Tur	S	S	S	S	S	S	S	S
15.0601391	Birmingham	UK	Delhi	S	S	S	S	S	S	S	S
16.0601418	Birmingham	UK	LAM	n/a	n/a	n/a	S	S	S	S	S
16.0601487	Birmingham	UK	M. bovis	S	S	S	R	S	S	S	R
15.0613462	Birmingham	UK	Delhi	S	S	S	S	S	S	S	S
16.0601668	Birmingham	UK	Beijing	R	S	R	S	R	S	R	S
13.0615676	Birmingham	UK	EAI	S	S	S	S	S	S	S	S
16.0607991	Birmingham	UK	EAI	S	S	S	S	S	S	S	S
15.0601490	Birmingham	UK	lineage4	S	S	S	S	S	S	S	S
16.0601751	Birmingham	UK	EAI	S	S	S	n/a	S	S	S	S
14.0609961	Birmingham	UK	Haarlem	S	S	S	S	S	S	S	S
14.0600713	Birmingham	UK	lineage4	R	S	S	S	R	S	S	S
14.0609079	Birmingham	UK		S	S	S	S	S	S	S	S
14.0600341	Birmingham	UK	Haarlem	S	S	S	S	S	S	U	S
14.0600980	Birmingham	UK	Beijing	R	R	S	R	R	R	R	R
15.0601178	Birmingham	UK	Haarlem	S	S	S	S	S	S	S	S
16.0606071	Birmingham	UK	Tur	S	S	S	S	S	S	S	S
09.0600851	Birmingham	UK	Delhi	S	S	S	S	S	S	S	S
14.0600348	Birmingham	UK	LAM	S	S	S	S	S	S	S	S
15.0601747	Birmingham	UK	EAI	S	S	S	S	S	S	S	S
14.0609148	Birmingham	UK	Haarlem	S	S	S	S	S	S	S	S
14.0617134	Birmingham	UK	Delhi	S	S	S	S	S	S	S	S
13.0615873	Birmingham	UK	lineage4	S	S	S	S	S	S	S	S
11.0607519	Birmingham	UK	Delhi	S	S	S	S	U	S	S	S
16.0601077	Birmingham	UK		S	S	S	S	S	S	S	S
15.0603414	Birmingham	UK	Delhi	S	S	S	S	S	S	S	S
16.0618186	Birmingham	UK	Delhi	R	R	S	S	R	R	R	S
15.0614167	Birmingham	UK	Haarlem	S	S	S	S	S	S	S	S
15.0603042	Birmingham	UK	EAI	S	S	S	S	S	S	S	S
17.0601150	Birmingham	UK	lineage4	S	S	S	S	S	S	S	S
16.0607577	Birmingham	UK	Beijing	S	S	S	S	S	S	S	S
12.0603488	Birmingham	UK	EAI	S	S	S	S	S	S	S	S
15.0601464	Birmingham	UK	LAM	S	S	S	S	S	S	U	S
15.0601087	Birmingham	UK	lineage4	S	S	S	S	S	S	S	S
12.0611245	Birmingham	UK	lineage4	S	S	S	S	S	S	S	S
13.0610998	Birmingham	UK	LAM	S	S	S	S	S	S	S	S
15.0609599	Birmingham	UK	EAI	S	S	S	S	S	S	S	S
12.0615222	Birmingham	UK	Delhi	S	S	S	S	S	S	S	S
16.0604350	Birmingham	UK	S-type	S	S	S	S	S	S	S	S
15.0601902	Birmingham	UK	EAI	S	S	S	S	S	S	S	S
14.0617333	Birmingham	UK	X-type	S	S	S	S	S	S	S	S
16.0601433	Birmingham	UK	Cameroon	S	S	S	S	S	S	S	S
16.0606072	Birmingham	UK	Cameroon	S	S	S	S	S	S	S	S
16.0601738	Birmingham	UK	Ural	R	S	S	S	R	S	S	S
14.0600320	Birmingham	UK	Delhi	S	S	S	S	S	S	S	S
14.0610986	Birmingham	UK	Delhi	S	S	S	S	S	S	U	S
12.0611854	Birmingham	UK	LAM	S	S	S	S	S	S	S	S
14.0603920	Birmingham	UK	Beijing	R	R	R	S	R	R	R	U
16.0608817	Birmingham	UK	Beijing	S	S	S	S	S	S	S	S
16.0601203	Birmingham	UK	S-type	S	S	S	S	S	S	S	S
15.0607721	Birmingham	UK	X-type	S	S	S	S	S	S	S	S
15.0609621	Birmingham	UK	Delhi	S	S	S	S	U	S	S	S
16.0609467	Birmingham	UK	Delhi	S	S	S	S	S	S	S	S
16.0607021	Birmingham	UK	Cameroon	S	S	S	S	S	S	S	S
13.0603086	Birmingham	UK	Delhi	S	S	S	S	S	S	S	S
13.0605666	Birmingham	UK	West Africar	S	S	S	S	S	S	U	S
13.0604549	Birmingham	UK	LAM	S	S	S	S	S	S	S	S
14.0606539	Birmingham	UK	LAM	S	S	S	S	S	S	S	S
13.0607143	Birmingham	UK	Delhi	S	S	S	S	S	S	S	S
13.0615934	Birmingham	UK	Cameroon	S	S	S	S	S	S	S	S
14.0609500	Birmingham	UK	LAM	S	S	S	S	S	S	S	S
15.0607031	Birmingham	UK	lineage4	S	S	S	S	S	S	S	S
15.0611576	Birmingham	UK	lineage4	S	S	S	S	S	S	S	S
13.0612178	Birmingham	UK	Delhi	S	S	S	S	S	S	S	S
14.0610315	Birmingham	UK	Delhi	S	S	S	S	S	S	S	S
15.0601806	Birmingham	UK	Delhi	S	S	S	n/a	S	S	S	S
14.0608099	Birmingham	UK	Delhi	S	S	S	S	S	S	S	S
15.0613555	Birmingham	UK	Delhi	S	S	S	S	S	S	S	S
16.0608342	Birmingham	UK	Beijing	n/a	n/a	S	S	R	R	S	R
13.0613460	Birmingham	UK	Haarlem	S	S	S	S	S	S	S	S
16.0617188	Birmingham	UK	Delhi	S	S	S	S	U	S	U	S

13.0602829	Birmingham	UK	Haarlem	S	S	S	S	S	S	S	S
14.0608783	Birmingham	UK	LAM	S	S	S	S	S	S	S	S
13.0606483	Birmingham	UK	Delhi	S	S	S	S	S	S	U	S
15.0607440	Birmingham	UK	Delhi	S	S	S	S	S	S	S	S
16.0607478	Birmingham	UK	EAI	R	S	S	S	R	S	S	S
12.0612396	Birmingham	UK	Delhi	S	S	S	S	S	S	S	S
15.0607276	Birmingham	UK	Delhi	S	S	S	S	S	S	S	S
12.0609606	Birmingham	UK	M. bovis	S	S	S	R	S	S	S	R
16.0600263	Birmingham	UK	lineage4	S	S	S	S	S	S	S	S
15.0607847	Birmingham	UK	Delhi	S	S	S	S	S	S	S	S
12.0611908	Birmingham	UK	lineage4	S	S	S	S	S	S	S	S
14.0602314	Birmingham	UK	M. orygis	S	S	S	S	S	S	S	S
16.0618554	Birmingham	UK	Haarlem	S	S	S	S	S	S	S	S
13.0615042	Birmingham	UK	lineage4	S	S	S	S	S	S	S	S
16.0606603	Birmingham	UK	Haarlem	S	S	S	S	S	S	S	S
13.0600320	Birmingham	UK	Delhi	S	S	S	S	S	S	U	S
14.0600079	Birmingham	UK	EAI	S	S	S	S	S	S	S	S
16.0600486	Birmingham	UK	LAM	S	S	S	S	S	S	S	S
15.0607028	Birmingham	UK	Tur	S	S	S	S	S	S	S	S
14.0617190	Birmingham	UK	Delhi	S	S	S	S	S	S	S	R
13.0611314	Birmingham	UK	Delhi	S	S	S	S	S	S	S	S
13.0606634	Birmingham	UK	Delhi	S	S	S	S	S	S	S	S
16.0601471	Birmingham	UK	Delhi	S	S	S	S	S	U	S	S
14.0604530	Birmingham	UK	Haarlem	S	S	S	S	S	S	S	S
13.0611014	Birmingham	UK	EAI	S	S	S	S	S	S	S	S
14.0611712	Birmingham	UK	EAI	S	S	S	S	S	S	S	S
13.0611912	Birmingham	UK	X-type	R	S	R	S	R	S	R	S
13.0616046	Birmingham	UK	S-type	R	S	S	S	R	S	U	S
16.0601693	Birmingham	UK	Beijing	R	S	S	S	R	S	S	F
16.0601130	Birmingham	UK	lineage4	R	n/a	R	R	R	R	R	F
15.0601903	Birmingham	UK	X-type	S	S	S	S	S	S	U	S
09.0603987	Birmingham	UK	Delhi	S	S	S	S	S	S	S	S
14.0605673	Birmingham	UK	Beijing	R	R	S	S	R	F	R	S
14.0600457	Birmingham	UK	LAM	S	S	S	S	S	S	S	S
16.0618129	Birmingham	UK	Haarlem	S	S	S	S	S	S	U	S
13.0602600	Birmingham	UK	EAI	S	S	S	S	S	S	S	S
13.0601883	Birmingham	UK	LAM	S	S	S	S	S	S	S	S
15.0611017	Birmingham	UK	Delhi	S	S	n/a	S	S	S	S	S
15.0613308	Birmingham	UK	X-type	S	S	S	S	S	S	S	S
13.0614128	Birmingham	UK	EAI	S	S	S	S	S	S	S	S
15.0607398	Birmingham	UK	lineage4	S	S	S	S	S	F	S	S
13.0606640	Birmingham	UK	Delhi	S	S	S	S	S	S	S	S
14.0605112	Birmingham	UK	Delhi	S	S	S	S	S	S	S	S
15.0601978	Birmingham	UK	Delhi	S	S	S	S	S	S	S	S
16.0601709	Birmingham	UK	LAM	S	S	S	S	S	S	S	S
16.0601214	Birmingham	UK	Beijing	R	S	S	S	R	S	F	F
12.0613610	Birmingham	UK	lineage4	S	S	S	S	S	S	S	S
16.0607592	Birmingham	UK	Haarlem	S	S	S	S	S	S	S	S
13.0607377	Birmingham	UK	Delhi	S	S	S	S	S	S	S	S
13.0612300	Birmingham	UK	Haarlem	S	S	S	S	S	S	S	S
15.0601344	Birmingham	UK	Delhi	S	S	S	S	S	S	S	S
14.0616061	Birmingham	UK	LAM	S	S	S	S	S	S	S	S
15.0609362	Birmingham	UK	LAM	S	S	S	S	S	S	S	S
13.0612134	Birmingham	UK	M. orygis	S	S	S	S	S	S	S	S
15.0607723	Birmingham	UK	lineage4	S	S	S	S	S	S	S	S
15.0601297	Birmingham	UK	lineage4	S	S	S	S	S	F	S	S
15.0608042	Birmingham	UK	Haarlem	S	S	S	S	S	S	S	S
15.0601129	Birmingham	UK	Tur	S	S	S	S	S	S	U	S
13.0613350	Birmingham	UK	LAM	S	S	S	S	S	S	S	S
15.0613082	Birmingham	UK		S	S	S	S	S	S	S	S
14.0600044	Birmingham	UK	Delhi	S	S	S	S	S	S	S	S
12.0611244	Birmingham	UK	lineage4	S	S	S	S	R	S	S	S
16.0618065	Birmingham	UK	M. orygis	n/a	n/a	n/a	S	S	F	U	S
13.0611305	Birmingham	UK	Delhi	S	S	S	S	S	S	S	S
12.0609357	Birmingham	UK	LAM	S	S	S	S	S	S	S	S
15.0601208	Birmingham	UK	EAI	S	S	S	S	S	F	U	S
14.0616190	Birmingham	UK	Delhi	S	S	S	S	S	S	S	S
16.0602593	Birmingham	UK	Delhi	S	S	S	S	S	S	S	S
12.0927948	Birmingham	UK	Delhi	S	S	S	S	S	S	U	S
15.0607352	Birmingham	UK		n/a	n/a	n/a	S	S	S	S	S
13.0611004	Birmingham	UK	Beijing	S	S	S	S	S	S	S	S
15.0607995	Birmingham	UK	lineage4	S	S	S	S	S	S	S	S
12.0612427	Birmingham	UK	X-type	S	S	S	S	S	S	S	S
12.0613161	Birmingham	UK	X-type	S	S	S	S	S	S	S	S
12.0601794	Birmingham	UK	Delhi	R	S	S	S	R	S	S	S
14.0600954	Birmingham	UK	lineage4	S	S	S	S	S	S	S	S
09.0607277	Birmingham	UK	EAI	R	S	S	S	R	S	S	S
15.0602437	Birmingham	UK	M. bovis	S	S	S	R	S	S	U	R
13.0609490	Birmingham	UK	EAI	S	S	S	S	S	S	S	S
16.0610042	Birmingham	UK	M. caprae	S	S	S	S	F	F	S	S
16.0600367	Birmingham	UK	Delhi	S	S	S	S	S	S	S	S
15.0607150	Birmingham	UK	Beijing	S	S	S	S	S	F	S	S
15.0613374	Birmingham	UK	Cameroon	S	S	S	S	S	S	S	S
14.0615239	Birmingham	UK	X-type	S	S	S	S	S	S	S	S
12.0611833	Birmingham	UK	Delhi	S	S	S	S	S	S	S	S
14.0609327	Birmingham	UK	LAM	S	S	S	S	S	S	S	S
13.0603131	Birmingham	UK	EAI	S	S	S	S	S	S	U	F
10.0603693	Birmingham	UK	lineage4	S	S	S	S	S	S	S	S
15.0613240	Birmingham	UK	Delhi	S	S	S	S	S	S	S	S
12.0617130	Birmingham	UK	X-type	S	S	S	S	S	S	S	S
16.0618058	Birmingham	UK	EAI	R	S	S	S	R	S	S	S
12.0616915	Birmingham	UK	Haarlem	S	S	S	S	S	S	S	S
15.0613098	Birmingham	UK	X-type	S	S	S	S	S	S	S	S
13.0603889	Birmingham	UK	EAI	S	S	S	S	S	S	S	S
13.0613233	Birmingham	UK	Haarlem	S	S	S	S	S	S	S	S
14.0609944	Birmingham	UK	EAI	S	S	S	S	S	S	U	S
15.0607109	Birmingham	UK	M. orygis	S	S	S	n/a	S	S	U	S
15.0601384	Birmingham	UK	West Africar	S	S	S	S	S	S	U	S
12.0926034	Birmingham	UK	West Africar	S	S	S	S	S	U	U	S
16.0605316	Birmingham	UK	LAM	S	S	S	S	S	S	S	S
12.0610727	Birmingham	UK	lineage4	S	S	S	S	S	S	S	S
13.0613258	Birmingham	UK	Ural	S	S	S	S	S	S	S	S
16.0607471	Birmingham	UK	EAI	S	S	S	n/a	S	S	S	S
14.0609775	Birmingham	UK	Delhi	S	S	S	S	S	S	S	S
15.0607799	Birmingham	UK	Delhi	S	S	S	S	S	S	S	S
15.0613025	Birmingham	UK	lineage4	S	S	S	S	S	S	S	S
13.0611248	Birmingham	UK	S-type	S	S	S	S	S	S	S	S
14.0614424	Birmingham	UK	Delhi	S	S	S	S	S	S	S	S
13.0613513	Birmingham	UK	lineage4	S	S	S	S	S	S	S	S
16.0618114	Birmingham	UK	Beijing	S	S	S	S	S	U	S	S
14.0615390	Birmingham	UK	Delhi	S	S	S	S	F	S	S	F
16.0601429	Birmingham	UK	Delhi	S	S	S	S	S	S	S	S
16.0605928	Birmingham	UK	Delhi	S	S	S	S	S	S	S	S
16.0603458	Birmingham	UK	Delhi	S	S	S	S	S	S	S	S
16.0618452	Birmingham	UK	lineage4	S	S	S	S	S	S	S	S
15.0613274	Birmingham	UK	Haarlem	S	S	S	S	S	S	S	S

13.0608751	Birmingham	UK	Cameroon	S	S	S	S	S	S	U	S
15.0601567	Birmingham	UK	Ural	R	S	S	S	R	S	S	S
12.0611799	Birmingham	UK	EAI	S	S	n/a	S	S	S	S	S
14.0600486	Birmingham	UK	Haarlem	S	S	S	S	S	S	S	S
14.0613401	Birmingham	UK	EAI	S	S	S	S	S	S	S	S
12.0927461	Birmingham	UK	Delhi	S	S	S	S	S	S	U	S
15.0613377	Birmingham	UK	Delhi	S	S	S	S	S	S	S	U
13.0609796	Birmingham	UK	Delhi	S	S	S	S	S	S	S	S
15.0613357	Birmingham	UK	Delhi	S	S	S	S	S	U	S	S
12.0608703	Birmingham	UK	Delhi	S	S	S	S	S	S	U	S
13.0610990	Birmingham	UK	EAI	S	S	S	S	S	S	S	S
14.0610389	Birmingham	UK	Beijing	S	S	S	S	S	S	S	S
13.0607698	Birmingham	UK	Delhi	S	S	S	S	S	S	S	S
16.0601836	Birmingham	UK	lineage4	S	S	S	S	S	S	U	S
12.0609803	Birmingham	UK	EAI	S	S	S	S	S	S	S	S
16.0606923	Birmingham	UK	Delhi	S	S	S	n/a	S	S	S	S
15.0601042	Birmingham	UK	lineage4	S	S	S	S	S	S	S	S
16.0600445	Birmingham	UK	S-type	S	S	S	S	S	S	S	S
14.0609651	Birmingham	UK	Tur	S	S	S	S	S	S	S	S
15.0601234	Birmingham	UK	Delhi	S	S	S	S	S	S	S	S
16.0602240	Birmingham	UK	LAM	S	S	S	S	S	S	S	S
14.0600247	Birmingham	UK	lineage4	S	S	S	S	S	S	S	S
13.0606720	Birmingham	UK	Delhi	S	S	S	S	S	S	S	S
12.0610018	Birmingham	UK	Delhi	S	S	S	S	S	S	S	S
14.0600999	Birmingham	UK	EAI	S	S	S	S	S	S	S	S
14.0607955	Birmingham	UK	Delhi	S	S	S	S	S	S	S	S
15.0609429	Birmingham	UK	Beijing	R	R	S	n/a	R	R	S	R
14.0600262	Birmingham	UK	lineage4	S	S	S	S	S	S	S	S
15.0607726	Birmingham	UK	Beijing	R	R	R	R	R	R	R	R
15.0601690	Birmingham	UK	lineage4	S	S	S	S	S	S	S	S
15.0605467	Birmingham	UK	LAM	S	S	S	S	S	S	S	S
12.0616927	Birmingham	UK	Delhi	S	S	S	S	S	S	S	S
14.0600847	Birmingham	UK	lineage4	S	S	S	S	S	S	S	S
13.0613569	Birmingham	UK	Delhi	R	S	S	S	R	S	S	S
13.0612190	Birmingham	UK	Delhi	S	S	S	S	S	S	S	U
13.0605695	Birmingham	UK	lineage4	S	S	S	S	S	S	S	S
15.0601954	Birmingham	UK	Ghana	R	S	S	S	S	S	S	S
16.0607415	Birmingham	UK	Delhi	S	S	S	S	S	S	S	S
11.0610283	Birmingham	UK	EAI	S	S	S	S	S	S	S	S
13.0613558	Birmingham	UK	Haarlem	S	S	S	S	S	S	S	S
10.0608154	Birmingham	UK	lineage4	S	S	S	S	S	S	S	S
15.0616215	Birmingham	UK	Beijing	R	R	R	n/a	R	R	R	R
16.0618432	Birmingham	UK	Delhi	S	S	S	S	S	S	S	S
09.0600944	Birmingham	UK	Beijing	S	S	S	S	S	S	S	S
15.0601749	Birmingham	UK	EAI	S	S	S	S	S	S	S	S
14.0602433	Birmingham	UK	lineage4	S	S	S	S	S	S	S	S
14.0604817	Birmingham	UK		S	S	S	S	S	S	S	S
13.0611012	Birmingham	UK	Delhi	S	S	S	S	S	S	S	S
10.0603635	Birmingham	UK	EAI	S	S	S	S	S	S	S	S
16.0607806	Birmingham	UK	Delhi	S	S	S	S	S	S	S	S
13.0608452	Birmingham	UK	Delhi	S	S	S	S	S	S	S	S
16.0603258	Birmingham	UK	Delhi	S	S	S	S	F	S	S	S
13.0600081	Birmingham	UK	EAI	S	S	S	S	F	S	S	S
15.0607907	Birmingham	UK	Delhi	S	S	S	S	S	S	S	S
13.0603120	Birmingham	UK	EAI	S	S	S	S	S	S	U	S
16.0601595	Birmingham	UK	lineage4	S	S	S	S	S	S	S	S
13.0612963	Birmingham	UK	EAI	S	S	S	S	S	S	S	S
16.0618136	Birmingham	UK	Delhi	S	S	S	S	S	U	S	S
13.0602595	Birmingham	UK	lineage4	S	S	S	S	S	S	S	S
16.0601783	Birmingham	UK	Haarlem	S	S	S	S	S	S	S	S
13.0610738	Birmingham	UK	Delhi	S	S	S	S	S	S	S	S
15.0607828	Birmingham	UK	X-type	S	S	S	S	S	S	U	S
13.0605102	Birmingham	UK	Delhi	S	S	S	S	S	S	S	S
14.0601953	Birmingham	UK	Delhi	S	S	S	S	S	S	S	S
13.0615533	Birmingham	UK	Beijing	R	R	S	S	R	F	R	U
13.0613447	Birmingham	UK	lineage4	S	S	S	S	S	S	S	S
14.0609187	Birmingham	UK	Delhi	S	S	S	S	S	S	S	S
16.0618482	Birmingham	UK	Beijing	S	S	S	S	S	S	S	S
13.0610414	Birmingham	UK	Delhi	S	S	S	S	S	S	S	S
15.0601442	Birmingham	UK	Delhi	S	S	S	S	S	S	S	S
15.0607282	Birmingham	UK	Haarlem	S	S	S	S	S	S	S	S
16.0607746	Birmingham	UK	LAM	S	S	S	S	S	S	S	S
14.0600249	Birmingham	UK	lineage4	S	S	S	S	S	S	S	S
16.0607886	Birmingham	UK	lineage4	S	S	S	S	S	S	S	S
15.0602331	Birmingham	UK	lineage4	n/a	n/a	n/a	S	S	S	S	S
16.0607624	Birmingham	UK	lineage4	S	S	S	S	S	S	S	S
16.0607060	Birmingham	UK	Haarlem	S	S	S	S	S	F	S	S
14.0616112	Birmingham	UK	Delhi	S	S	S	S	S	S	S	S
14.0605394	Birmingham	UK	Delhi	S	S	S	S	S	S	S	S
12.0611871	Birmingham	UK	lineage4	S	S	S	S	S	S	U	S
15.0607356	Birmingham	UK	M. bovis	S	S	S	R	S	S	S	R
16.0607739	Birmingham	UK	LAM	S	S	S	S	S	S	S	S
14.0600763	Birmingham	UK	Beijing	S	S	S	S	S	S	S	S
14.0614124	Birmingham	UK	Delhi	S	S	S	S	S	S	S	S
10.0611737	Birmingham	UK	Delhi	S	S	S	S	S	S	S	S
13.0613108	Birmingham	UK	M. bovis	S	S	S	R	S	S	U	R
13.0600594	Birmingham	UK	Delhi	S	S	S	S	S	S	S	S
15.0601761	Birmingham	UK	Delhi	S	S	S	S	S	S	S	S
15.0607496	Birmingham	UK	Delhi	S	S	S	S	S	S	S	S
14.0600403	Birmingham	UK	Delhi	S	S	S	S	S	S	S	S
15.0602317	Birmingham	UK	Beijing	R	S	R	S	R	S	R	S
13.0613398	Birmingham	UK	Delhi	S	S	S	S	S	S	S	S
16.0602440	Birmingham	UK	LAM	S	S	S	S	S	S	S	S
15.0613467	Birmingham	UK	Delhi	S	S	S	S	S	F	S	F
16.0601574	Birmingham	UK	Haarlem	S	S	S	S	S	S	S	S
16.0617159	Birmingham	UK	Ural	R	S	S	S	R	S	S	S
14.0609262	Birmingham	UK	Delhi	S	S	S	S	S	S	S	S
15.0601860	Birmingham	UK	S-type	S	S	S	S	S	S	U	S
10.0605875	Birmingham	UK	EAI	R	S	S	S	R	S	S	S
14.0600508	Birmingham	UK	EAI	S	S	S	S	S	S	S	S
14.0605011	Birmingham	UK	Delhi	R	S	S	S	R	S	S	S
14.0600837	Birmingham	UK	Delhi	S	S	S	S	S	S	S	S
14.0600257	Birmingham	UK	Delhi	S	S	S	S	S	S	S	S
15.0601481	Birmingham	UK	EAI	S	S	S	S	S	S	S	S
14.0602173	Birmingham	UK	Cameroon	S	S	S	S	S	S	S	S
13.0605489	Birmingham	UK	lineage4	S	S	S	S	S	S	S	S
15.0607499	Birmingham	UK	Delhi	S	S	S	S	S	S	S	S
14.0600939	Birmingham	UK	Delhi	S	S	S	S	S	S	S	S
16.0607802	Birmingham	UK	Beijing	S	S	S	S	S	S	U	U
14.0609469	Birmingham	UK	Cameroon	S	S	S	S	S	S	S	S
16.0601887	Birmingham	UK	EAI	S	S	S	S	S	S	S	S
14.0609103	Birmingham	UK	Haarlem	S	S	S	S	S	S	S	S
16.0607723	Birmingham	UK	Delhi	S	S	S	S	S	S	S	S
15.0601958	Birmingham	UK	EAI	S	S	S	n/a	S	S	S	S
16.0604554	Birmingham	UK	X-type	S	S	S	S	S	S	U	S
13.0601564	Birmingham	UK	Haarlem	S	S	S	S	S	S	S	S

16.0618611	Birmingham	UK	Delhi	S	S	S	S	S	S	S	S
15.0607053	Birmingham	UK	LAM	S	S	S	S	S	S	S	S
12.0608996	Birmingham	UK	Delhi	S	S	S	S	S	S	S	S
16.0603633	Birmingham	UK	X-type	S	S	S	S	S	S	S	S
13.0609851	Birmingham	UK	lineage4	S	S	S	S	S	S	S	S
12.0612506	Birmingham	UK	S-type	S	S	S	S	S	S	S	S
14.0600029	Birmingham	UK	lineage4	S	S	S	S	S	S	S	S
14.0600698	Birmingham	UK	S-type	S	S	S	S	S	S	U	S
16.0617531	Birmingham	UK	Delhi	S	S	S	S	S	S	S	S
16.0618429	Birmingham	UK	X-type	S	S	S	S	S	S	S	S
15.0601367	Birmingham	UK	EAI	S	S	S	S	S	S	S	S
14.0610446	Birmingham	UK	Delhi	S	S	S	S	S	S	S	S
15.0600510	Birmingham	UK	LAM	S	S	S	S	S	S	S	S
12.0927854	Birmingham	UK	LAM	S	S	S	S	S	S	S	S
13.0611009	Birmingham	UK	Delhi	S	S	S	S	S	S	S	S
15.0611920	Birmingham	UK	lineage4	S	S	S	S	S	S	S	S
15.0601914	Birmingham	UK	Haarlem	S	S	S	S	S	S	U	S
12.0927024	Birmingham	UK	lineage4	S	S	S	S	S	S	S	S
12.0611788	Birmingham	UK	Delhi	S	S	S	S	S	S	S	S
13.0613521	Birmingham	UK	lineage4	S	S	S	S	S	S	U	S
16.0618193	Birmingham	UK	Delhi	S	S	S	S	S	S	S	S
16.0601070	Birmingham	UK		S	S	S	S	S	S	S	S
15.0601745	Birmingham	UK	lineage4	R	S	S	S	F	F	F	F
16.0617146	Birmingham	UK	Haarlem	S	S	S	S	S	S	S	S
13.0609429	Birmingham	UK	Delhi	S	S	S	S	S	S	S	S
14.0615561	Birmingham	UK	Delhi	S	S	S	n/a	S	S	S	S
14.0600604	Birmingham	UK	X-type	S	S	S	S	S	S	S	S
10.0607622	Birmingham	UK	EAI	S	S	S	S	S	S	S	S
15.0607700	Birmingham	UK		S	S	S	S	F	S	S	S
13.0613502	Birmingham	UK	LAM	S	S	S	S	S	S	S	S
11.0601003	Birmingham	UK	Delhi	S	S	S	S	S	S	S	S
16.0607382	Birmingham	UK	X-type	S	S	S	S	S	S	S	S
13.0616419	Birmingham	UK	EAI	S	S	S	S	S	S	S	S
12.0614543	Birmingham	UK	lineage4	S	S	S	S	S	S	U	S
13.0607977	Birmingham	UK	Delhi	S	S	S	S	S	S	S	S
12.0613614	Birmingham	UK	lineage4	S	S	S	S	S	S	U	S
13.0602840	Birmingham	UK	lineage4	S	S	S	S	S	S	S	S
15.0607504	Birmingham	UK	Delhi	S	S	S	S	S	S	S	S
15.0601363	Birmingham	UK	Delhi	S	S	S	S	S	S	S	S
15.0613217	Birmingham	UK	S-type	S	S	S	S	S	S	S	S
15.0607497	Birmingham	UK	Delhi	S	S	S	S	S	S	S	S
15.0601033	Birmingham	UK	LAM	R	S	S	S	R	S	S	U
15.0601889	Birmingham	UK	LAM	S	S	S	S	S	S	S	S
13.0606701	Birmingham	UK	EAI	S	S	S	S	S	S	S	S
14.0603858	Birmingham	UK	Delhi	S	S	S	S	S	S	S	S
16.0601327	Birmingham	UK	Delhi	S	S	S	S	S	S	S	S
16.0601974	Birmingham	UK	Delhi	S	S	S	S	S	S	S	S
14.0609455	Birmingham	UK	Haarlem	S	S	S	S	S	S	S	S
16.0601980	Birmingham	UK	lineage4	S	S	S	S	S	F	S	S
15.0607326	Birmingham	UK	lineage4	S	S	S	S	S	S	S	S
12.0617313	Birmingham	UK	S-type	S	S	S	S	S	S	S	S
15.0601032	Birmingham	UK	Delhi	S	S	S	S	S	S	S	S
12.0612555	Birmingham	UK	West Africar	S	S	S	S	S	U	U	S
16.0618624	Birmingham	UK	Delhi	n/a	S	S	n/a	S	S	S	S
15.0607238	Birmingham	UK	Delhi	S	S	S	S	S	S	S	S
17.0601226	Birmingham	UK	Delhi	S	S	S	S	S	F	S	S
16.0617291	Birmingham	UK	M. bovis	S	S	S	R	S	S	S	R
15.0609387	Birmingham	UK	LAM	S	S	S	S	S	S	S	S
13.0603097	Birmingham	UK	LAM	S	S	S	S	S	S	S	F
16.0607046	Birmingham	UK	Delhi	n/a	S	n/a	S	R	S	U	S
13.0606213	Birmingham	UK	Beijing	S	S	S	S	S	S	S	S
15.0613043	Birmingham	UK	S-type	S	S	S	S	S	S	S	S
15.0607494	Birmingham	UK	Delhi	S	S	S	S	S	S	S	S
15.0613336	Birmingham	UK	LAM	S	S	S	S	S	S	S	S
14.0601935	Birmingham	UK	lineage4	S	S	S	S	S	S	S	S
13.0605770	Birmingham	UK	lineage4	S	S	S	S	S	S	S	S
16.0607519	Birmingham	UK	Beijing	S	S	S	S	S	S	U	S
13.0612115	Birmingham	UK	Delhi	S	S	S	S	S	S	S	S
12.0611454	Birmingham	UK	S-type	S	S	S	S	S	S	S	R
13.0610491	Birmingham	UK	Tur	S	S	S	S	S	S	S	S
16.0607017	Birmingham	UK	Delhi	S	S	S	S	S	S	S	S
14.0600521	Birmingham	UK	Beijing	n/a	n/a	n/a	S	S	S	S	S
14.0600537	Birmingham	UK	Delhi	S	S	S	S	S	S	S	S
15.0604342	Birmingham	UK	lineage4	S	S	S	S	S	S	S	S
15.0613195	Birmingham	UK	lineage4	S	S	S	S	S	S	S	S
14.0609004	Birmingham	UK	lineage4	S	S	S	S	S	S	S	R
16.0603017	Birmingham	UK	Delhi	S	S	S	S	S	S	S	S
16.0601439	Birmingham	UK	Ghana	R	S	S	S	R	S	S	S
12.0616622	Birmingham	UK	lineage4	S	S	S	S	S	S	S	S
14.0616168	Birmingham	UK	lineage4	S	S	S	S	S	S	S	S
15.0607865	Birmingham	UK	X-type	S	S	S	S	S	S	S	S
16.0601538	Birmingham	UK	Delhi	S	S	S	S	S	S	S	S
14.0600807	Birmingham	UK	LAM	S	S	S	S	S	S	S	S
12.0612525	Birmingham	UK	EAI	S	S	S	S	S	S	U	S
14.0614425	Birmingham	UK	Beijing	S	S	S	S	S	S	S	S
15.0608520	Birmingham	UK	X-type	S	S	S	n/a	S	S	S	R
11.0602681	Birmingham	UK	Delhi	S	S	S	S	S	S	S	S
11.0612547	Birmingham	UK	S-type	S	S	S	S	S	S	S	S
14.0609114	Birmingham	UK	lineage4	S	S	S	S	S	S	U	S
16.0601250	Birmingham	UK	Haarlem	R	S	S	S	R	S	R	S
12.0616631	Birmingham	UK	Haarlem	S	S	S	S	S	S	S	S
16.0601658	Birmingham	UK	lineage4	S	S	S	S	U	S	S	S
12.0614963	Birmingham	UK	lineage4	S	S	S	S	S	S	S	S
14.0609008	Birmingham	UK	lineage4	S	S	S	n/a	S	S	S	S
11.0615660	Birmingham	UK	Delhi	S	S	S	S	S	S	S	S
16.0601304	Birmingham	UK	Delhi	S	S	S	S	S	S	S	S
15.0602532	Birmingham	UK	Haarlem	R	S	S	S	R	S	S	S
14.0600783	Birmingham	UK	Haarlem	S	S	S	S	S	S	S	S
14.0600803	Birmingham	UK	EAI	S	S	S	S	S	S	S	S
12.0927480	Birmingham	UK	Delhi	S	S	S	S	S	S	U	S
14.0612233	Birmingham	UK	EAI	S	S	S	S	S	S	U	S
15.0607106	Birmingham	UK	EAI	S	S	S	S	S	S	S	S
14.0600568	Birmingham	UK	LAM	S	S	S	S	S	S	S	S
12.0616918	Birmingham	UK	Delhi	S	S	S	S	S	S	U	S
15.0601824	Birmingham	UK	Delhi	S	S	S	S	S	S	S	S
13.0608728	Birmingham	UK	Delhi	n/a	n/a	n/a	S	S	S	S	S
16.0607212	Birmingham	UK	Delhi	S	S	S	S	S	F	S	S
16.0618385	Birmingham	UK	Delhi	S	S	S	n/a	F	F	U	S
12.0613605	Birmingham	UK	West Africar	S	S	S	S	S	U	U	S
14.0600933	Birmingham	UK	EAI	S	S	S	S	S	S	S	S
15.0600918	Birmingham	UK	X-type	S	S	S	S	S	S	S	S
16.0601766	Birmingham	UK	LAM	S	S	S	S	S	S	S	S
15.0607741	Birmingham	UK	lineage4	R	R	S	R	R	R	R	R
12.0610882	Birmingham	UK	X-type	S	S	S	S	U	S	S	S
14.0600597	Birmingham	UK		S	S	S	S	S	S	S	S
16.0607152	Birmingham	UK	Beijing	R	R	n/a	S	R	R	R	S

12.0617268	Birmingham	UK	Delhi	S	S	S	S	F	S	S	S
16.0601539	Birmingham	UK	X-type	S	S	S	S	S	S	U	S
13.0607677	Birmingham	UK	Delhi	S	S	S	S	S	S	S	S
12.0614228	Birmingham	UK	Haarlem	S	S	S	S	S	S	S	S
15.0607206	Birmingham	UK	Beijing	S	S	S	S	S	S	S	S
16.0607018	Birmingham	UK	West Africar	S	S	S	S	S	U	U	S
14.0609875	Birmingham	UK	Delhi	S	S	S	S	S	S	S	S
14.0600744	Birmingham	UK	EAI	S	S	S	S	S	S	S	S
13.0602029	Birmingham	UK	lineage4	S	S	S	S	S	S	S	S
12.0612885	Birmingham	UK	Beijing	S	S	S	S	S	S	S	S
14.0613590	Birmingham	UK	Beijing	R	R	R	R	R	R	R	R
16.0607084	Birmingham	UK	lineage4	S	S	S	S	S	S	S	S
16.0617013	Birmingham	UK	Delhi	S	S	S	S	S	S	U	S
16.0602610	Birmingham	UK	lineage4	S	S	S	S	S	S	U	S
16.0607106	Birmingham	UK	lineage4	S	S	S	S	S	S	S	S
15.0607094	Birmingham	UK	Delhi	S	S	S	S	S	S	S	S
15.0607510	Birmingham	UK	lineage4	S	S	S	S	S	S	S	S
15.0601438	Birmingham	UK	lineage4	S	S	S	S	S	S	S	S
15.0607728	Birmingham	UK	Haarlem	S	R	S	S	S	R	S	S
15.0601081	Birmingham	UK	Delhi	S	S	S	S	S	S	S	S
15.0607637	Birmingham	UK	Haarlem	S	S	S	S	S	S	S	S
13.0607533	Birmingham	UK	Delhi	S	S	S	S	S	S	S	S
16.0601360	Birmingham	UK	lineage4	R	S	S	S	R	S	S	S
16.0618428	Birmingham	UK	lineage4	S	S	S	S	S	S	S	S
11.0611836	Birmingham	UK	Delhi	S	S	S	S	S	S	S	S
14.0604728	Birmingham	UK	Delhi	S	S	S	S	S	S	S	S
16.0607032	Birmingham	UK	lineage4	S	S	S	S	S	S	S	S
13.0612472	Birmingham	UK	Haarlem	S	S	S	S	S	S	S	S
16.0601296	Birmingham	UK	M. bovis	S	S	S	R	S	S	S	R
09.0606605	Birmingham	UK	X-type	S	S	S	S	S	S	S	S
14.0611289	Birmingham	UK	EAI	S	S	S	S	S	S	U	S
15.0607736	Birmingham	UK	Beijing	R	R	R	R	R	R	R	R
13.0609452	Birmingham	UK	Haarlem	S	S	S	S	S	S	S	S
15.0600734	Birmingham	UK	X-type	R	S	R	S	R	R	R	F
16.0601420	Birmingham	UK	lineage4	S	S	S	S	S	S	S	S
15.0601494	Birmingham	UK	Delhi	S	S	S	S	S	S	S	S
14.0609453	Birmingham	UK	Delhi	S	S	S	S	S	S	S	S
16.0607885	Birmingham	UK	EAI	S	S	S	S	S	S	S	S
14.0609700	Birmingham	UK	Ural	S	S	S	S	S	S	S	S
13.0612473	Birmingham	UK	X-type	S	S	S	S	S	S	S	S
14.0600536	Birmingham	UK		S	S	S	S	S	F	S	S
14.0609271	Birmingham	UK	Delhi	S	S	S	S	S	S	S	S
15.0605804	Birmingham	UK	LAM	S	S	S	S	S	S	S	S
09.0605673	Birmingham	UK	EAI	S	S	S	S	S	S	S	S
13.0613499	Birmingham	UK	X-type	S	S	S	S	S	S	S	S
13.0609551	Birmingham	UK	Haarlem	S	S	S	S	S	S	U	S
15.0613199	Birmingham	UK	Delhi	R	R	n/a	S	R	R	R	R
16.0601724	Birmingham	UK	lineage4	S	S	S	S	S	S	S	S
13.0613557	Birmingham	UK	Haarlem	S	S	S	S	S	S	S	S
15.0601771	Birmingham	UK	Delhi	S	S	S	S	S	F	S	S
12.0616397	Birmingham	UK	EAI	S	S	S	S	U	S	S	S
15.0601968	Birmingham	UK	EAI	S	S	S	S	S	S	S	S
13.0603128	Birmingham	UK	Delhi	S	S	S	S	S	S	S	S
15.0614445	Birmingham	UK	Delhi	S	S	S	S	U	S	S	S
14.0609914	Birmingham	UK	lineage4	S	S	S	S	U	S	S	S
15.0601105	Birmingham	UK	Delhi	S	S	S	S	U	S	S	S
13.0612211	Birmingham	UK	Delhi	S	S	S	S	S	S	S	S
14.0609034	Birmingham	UK	Delhi	S	S	S	S	F	S	S	S
15.0616130	Birmingham	UK	EAI	S	S	S	S	F	S	S	S
16.0601359	Birmingham	UK	X-type	S	S	S	S	S	S	S	S
16.0618181	Birmingham	UK	X-type	S	S	S	S	S	S	S	S
14.0609319	Birmingham	UK	S-type	S	S	S	S	F	S	S	S
15.0613345	Birmingham	UK	Delhi	S	S	S	S	S	U	S	S
14.0600985	Birmingham	UK	lineage4	S	S	S	S	S	S	S	S
14.0600542	Birmingham	UK	Beijing	S	S	S	S	S	S	S	S
16.0618437	Birmingham	UK	Ural	S	S	S	S	S	S	S	S
14.0600464	Birmingham	UK	LAM	S	S	S	S	S	S	S	S
13.0612213	Birmingham	UK	LAM	S	S	S	S	S	S	S	S
14.0600252	Birmingham	UK	Delhi	S	S	S	S	S	S	S	S
15.0607902	Birmingham	UK	M. orygis	S	S	S	S	S	S	S	S
14.0609431	Birmingham	UK	EAI	S	S	S	S	S	S	S	S
16.0601516	Birmingham	UK	Haarlem	R	S	R	R	R	R	R	S
12.0605218	Birmingham	UK	Delhi	S	S	S	S	S	S	S	S
12.0605641	Birmingham	UK	X-type	S	S	S	S	S	S	S	S
15.0610739	Birmingham	UK	Haarlem	S	S	S	S	S	S	U	S
15.0602483	Birmingham	UK	lineage4	S	S	S	S	S	S	S	S
16.0603791	Birmingham	UK	Delhi	S	S	S	S	S	S	S	F
12.0617799	Birmingham	UK	Delhi	S	S	S	S	S	S	S	S
14.0616206	Birmingham	UK	lineage4	S	S	S	S	S	S	U	S
14.0600996	Birmingham	UK	Beijing	S	S	S	S	S	S	S	S
12.0616797	Birmingham	UK	Delhi	S	S	S	S	S	S	S	S
17.0601075	Birmingham	UK	Beijing	S	S	S	S	S	S	S	S
14.0600470	Birmingham	UK	Delhi	S	S	S	S	S	S	S	S
12.0611896	Birmingham	UK	Delhi	S	S	S	S	S	S	S	S
14.0600971	Birmingham	UK	S-type	S	S	S	S	S	S	S	S
14.0604054	Birmingham	UK	Delhi	S	S	S	S	S	S	S	S
15.0613220	Birmingham	UK	Haarlem	S	S	S	n/a	S	S	S	S
13.0611016	Birmingham	UK	Delhi	S	S	S	S	S	S	S	S
13.0611315	Birmingham	UK	Delhi	S	S	S	S	S	S	S	S
12.0603731	Birmingham	UK	Delhi	S	S	S	S	S	S	S	S
15.0601738	Birmingham	UK	EAI	S	S	S	S	S	S	S	S
13.0613029	Birmingham	UK	Delhi	S	S	S	S	S	S	U	S
14.0609404	Birmingham	UK	Delhi	S	S	S	S	S	S	S	S
11.0608172	Birmingham	UK	Delhi	S	S	S	S	S	S	S	S
14.0600092	Birmingham	UK	Delhi	S	S	S	S	S	S	S	S
14.0610922	Birmingham	UK	Delhi	S	S	S	S	S	S	S	S
13.0613329	Birmingham	UK	Haarlem	S	S	S	S	S	S	U	S
13.0608410	Birmingham	UK	Delhi	S	S	S	S	S	S	S	S
12.0611602	Birmingham	UK	LAM	S	S	S	S	S	S	S	S
15.0613108	Birmingham	UK	Haarlem	S	S	S	S	S	U	S	S
15.0601629	Birmingham	UK	Beijing	S	S	S	S	S	S	S	S
16.0607974	Birmingham	UK	LAM	S	S	S	S	S	S	S	S
16.0600456	Birmingham	UK	Delhi	S	S	S	S	F	F	S	F
15.0611953	Birmingham	UK	Delhi	S	S	S	S	S	S	U	S
16.0607323	Birmingham	UK	Haarlem	S	S	S	S	S	S	U	S
15.0601627	Birmingham	UK	Haarlem	S	S	S	S	S	S	S	S
13.0607668	Birmingham	UK	LAM	S	S	S	S	S	S	S	S
16.0607800	Birmingham	UK	lineage4	S	S	S	S	S	S	S	S
12.0611205	Birmingham	UK	lineage4	S	S	S	S	U	S	S	S
16.0601582	Birmingham	UK	Delhi	S	S	S	S	S	S	S	S
16.0600499	Birmingham	UK	LAM	S	S	S	S	S	S	S	S
10.0612076	Birmingham	UK	Delhi	S	S	S	S	S	S	S	S
14.0609429	Birmingham	UK	EAI	S	S	S	R	U	S	S	R
13.0608437	Birmingham	UK	Uganda	S	S	S	S	S	S	S	S
16.0617651	Birmingham	UK	lineage4	S	S	S	S	S	S	S	S
15.0601753	Birmingham	UK	Delhi	S	S	S	S	S	S	S	S

14.0610278	Birmingham	UK	Haarlem	S	S	S	S	S	S	S	U
15.0607746	Birmingham	UK	LAM	R	R	R	R	R	R	R	R
16.0618588	Birmingham	UK	lineage4	n/a	S	S	S	S	S	S	S
13.0612961	Birmingham	UK	LAM	S	S	S	S	S	S	S	S
14.0600223	Birmingham	UK	Haarlem	S	S	S	S	S	S	S	S
15.0607638	Birmingham	UK	Beijing	S	S	S	S	S	S	S	S
15.0607876	Birmingham	UK	EAI	S	S	S	S	S	S	S	S
15.0607936	Birmingham	UK	Cameroon	S	S	S	S	S	S	S	S
15.0611869	Birmingham	UK	Haarlem	S	S	S	S	S	S	S	F
16.0607867	Birmingham	UK	EAI	S	S	S	S	S	S	S	S
14.0600724	Birmingham	UK	lineage4	S	S	S	S	S	S	S	S
14.0609941	Birmingham	UK	LAM	S	S	S	S	S	S	S	S
14.0609917	Birmingham	UK	EAI	S	S	S	S	S	S	S	S
16.0601226	Birmingham	UK	lineage4	S	S	S	S	S	S	S	S
14.0609968	Birmingham	UK	LAM	S	S	S	S	S	S	S	S
15.0613152	Birmingham	UK	Haarlem	S	S	S	S	S	S	S	S
12.0613629	Birmingham	UK	Haarlem	S	S	S	S	S	S	S	S
14.0609757	Birmingham	UK	Ghana	S	S	S	S	S	S	S	S
16.0601396	Birmingham	UK	lineage4	S	S	S	S	S	S	S	S
13.0604721	Birmingham	UK	Delhi	S	S	S	S	S	S	S	S
13.0609245	Birmingham	UK		S	S	S	S	S	S	S	S
12.0611827	Birmingham	UK	EAI	S	S	S	S	S	S	S	S
12.0615253	Birmingham	UK		S	S	S	S	S	S	S	S
16.0601797	Birmingham	UK	lineage4	S	S	S	S	S	S	S	S
16.0617966	Birmingham	UK	LAM	R	R	R	S	R	R	R	R
13.0604198	Birmingham	UK	X-type	S	S	S	S	S	S	S	S
14.0600680	Birmingham	UK	Tur	S	S	S	S	S	S	S	S
16.0601041	Birmingham	UK	EAI	R	S	S	S	R	S	U	S
12.0600898	Birmingham	UK	West Africar	S	S	S	S	S	S	U	S
15.0607475	Birmingham	UK	Delhi	R	R	S	n/a	R	R	R	R
14.0600950	Birmingham	UK	Delhi	S	S	S	S	S	S	S	S
13.0602072	Birmingham	UK	lineage4	S	S	S	S	S	S	S	S
14.0616077	Birmingham	UK	Delhi	S	S	S	S	S	S	S	S
15.0600950	Birmingham	UK	Beijing	S	S	S	S	S	S	U	S
12.0604332	Birmingham	UK	Uganda	S	S	S	S	S	S	S	S
14.0609733	Birmingham	UK	Delhi	S	S	S	S	S	S	S	S
12.0614540	Birmingham	UK	lineage4	S	S	S	S	F	S	S	S
16.0618502	Birmingham	UK	lineage4	S	S	S	S	S	S	S	S
17.0601213	Birmingham	UK	Delhi	S	S	S	n/a	S	U	S	S
16.0607705	Birmingham	UK	Ural	S	S	S	S	S	F	F	S
15.0601459	Birmingham	UK	Delhi	S	S	S	S	S	S	S	S
13.0604519	Birmingham	UK	Delhi	S	S	S	S	S	S	S	S
13.0602086	Birmingham	UK	lineage4	S	S	S	S	S	S	S	S
12.0616110	Birmingham	UK	lineage4	S	S	S	S	S	S	S	S
13.0613004	Birmingham	UK	LAM	S	S	S	S	S	S	S	S
12.0610801	Birmingham	UK	X-type	S	S	S	S	S	S	S	S
15.0607193	Birmingham	UK	LAM	S	S	S	S	S	F	S	S
15.0613490	Birmingham	UK	Tur	S	S	S	S	S	S	S	S
17.0601155	Birmingham	UK	lineage4	S	S	S	S	S	S	S	S
15.0601929	Birmingham	UK	Beijing	S	S	S	S	S	S	S	S
14.0614648	Birmingham	UK	Delhi	n/a	n/a	n/a	S	S	S	S	S
14.0600528	Birmingham	UK	Delhi	S	S	S	S	S	S	S	S
15.0613069	Birmingham	UK	lineage4	S	S	S	S	S	U	S	S
14.0609026	Birmingham	UK	Delhi	S	S	S	S	S	S	S	S
16.0618614	Birmingham	UK	lineage4	S	S	S	S	S	S	S	S
13.0609002	Birmingham	UK	Beijing	S	S	S	S	S	S	S	S
12.0612489	Birmingham	UK	S-type	S	S	S	S	S	S	S	S
16.0601680	Birmingham	UK	Haarlem	S	S	S	S	S	S	S	S
15.0613461	Birmingham	UK		S	S	S	S	S	S	U	S
14.0609601	Birmingham	UK	lineage4	S	S	S	S	S	S	S	S
15.0607614	Birmingham	UK	lineage4	S	S	S	S	S	S	S	S
14.0600310	Birmingham	UK	EAI	S	S	S	S	S	S	S	S
13.0607129	Birmingham	UK	Delhi	S	S	S	S	S	S	S	S
13.0612192	Birmingham	UK	lineage4	S	S	S	S	S	S	S	S
15.0601141	Birmingham	UK	lineage4	S	S	S	S	S	F	S	S
14.0600694	Birmingham	UK	Haarlem	S	S	S	S	S	S	S	S
16.0618187	Birmingham	UK	Delhi	n/a	S	S	S	F	F	U	S
12.0616534	Birmingham	UK	Cameroon	R	S	S	S	R	S	S	S
14.0609466	Birmingham	UK	Delhi	S	S	S	S	S	S	S	S
11.0613616	Birmingham	UK	Delhi	S	S	S	S	U	S	S	S
15.0613233	Birmingham	UK	EAI	R	S	S	S	R	S	U	S
14.0614226	Birmingham	UK	Delhi	S	S	S	S	S	S	S	S
16.0613983	Birmingham	UK	Delhi	S	S	S	S	S	S	S	S
16.0601316	Birmingham	UK	lineage4	S	S	S	S	S	S	S	S
14.0600058	Birmingham	UK	Delhi	S	S	S	S	S	S	S	S
15.0607658	Birmingham	UK	X-type	S	S	S	S	S	S	U	S
12.0614237	Birmingham	UK	Delhi	S	S	S	S	S	S	S	S
13.0603145	Birmingham	UK	Delhi	S	S	S	S	S	S	U	S
14.0600805	Birmingham	UK	Delhi	S	S	S	S	S	S	S	S
12.0617738	Birmingham	UK	M. bovis	S	S	S	R	S	S	S	R
16.0607248	Birmingham	UK	Delhi	S	S	S	S	R	S	S	S
15.0607226	Birmingham	UK	Beijing	S	S	S	S	S	S	S	S
16.0601579	Birmingham	UK	EAI	S	S	S	n/a	S	S	S	S
15.0601780	Birmingham	UK	lineage4	S	S	S	S	S	U	S	S
15.0607192	Birmingham	UK	Delhi	S	S	S	S	S	S	S	S
15.0607767	Birmingham	UK	Cameroon	S	S	S	S	S	S	U	S
12.0611852	Birmingham	UK	Beijing	S	S	S	S	S	S	S	S
15.0602907	Birmingham	UK	Delhi	R	R	S	S	R	F	S	S
16.0607441	Birmingham	UK	lineage4	S	S	S	S	S	S	S	S
16.0601276	Birmingham	UK	lineage4	S	S	S	S	S	S	S	S
16.0604314	Birmingham	UK	Delhi	S	S	S	S	F	U	F	F
14.0616035	Birmingham	UK	lineage4	S	S	S	S	S	S	S	S
12.0611277	Birmingham	UK	Haarlem	S	S	S	S	S	S	S	S
16.0617205	Birmingham	UK	LAM	S	S	S	S	S	S	S	S
14.0609940	Birmingham	UK	Delhi	S	S	S	S	S	S	S	S
16.0607890	Birmingham	UK	lineage4	n/a	n/a	n/a	S	S	S	S	S
13.0604070	Birmingham	UK	X-type	S	S	S	S	S	S	S	S
16.0612679	Birmingham	UK	lineage4	R	S	S	S	R	S	S	S
16.0601681	Birmingham	UK	Haarlem	n/a	S	S	n/a	R	R	R	S
15.0607381	Birmingham	UK	M. orygis	n/a	S	S	n/a	F	S	U	F
14.0600396	Birmingham	UK	LAM	S	S	S	S	S	S	S	S
14.0610839	Birmingham	UK	lineage4	S	S	S	S	S	S	S	S
15.0613188	Birmingham	UK	M. orygis	n/a	n/a	n/a	S	S	S	S	S
14.0603568	Birmingham	UK	Delhi	S	S	S	S	S	S	S	S
15.0607016	Birmingham	UK	EAI	S	S	S	S	S	S	S	S
14.0609388	Birmingham	UK	Delhi	S	S	S	S	S	S	S	S
14.0600482	Birmingham	UK	lineage4	S	S	S	S	S	S	S	S
15.0611364	Birmingham	UK	Delhi	S	S	S	S	S	S	S	S
16.0601260	Birmingham	UK	Cameroon	R	S	S	S	R	S	S	S
14.0600609	Birmingham	UK	Ghana	R	S	S	S	R	S	S	S
15.0601875	Birmingham	UK	EAI	S	S	S	S	S	S	S	S
15.0607891	Birmingham	UK	EAI	S	S	S	n/a	S	S	S	S
15.0601628	Birmingham	UK	Delhi	S	S	S	S	S	U	S	S
13.0606480	Birmingham	UK	Delhi	S	S	S	S	F	S	S	S
15.0604813	Birmingham	UK	Delhi	S	S	S	S	U	S	S	S
16.0600545	Birmingham	UK	lineage4	S	S	S	S	S	S	S	S

13.0607717	Birmingham	UK	Haarlem	S	S	S	S	S	S	U	S
16.0601190	Birmingham	UK	Delhi	S	S	S	S	S	S	S	S
12.0614204	Birmingham	UK	lineage4	S	S	S	S	S	S	S	S
15.0601871	Birmingham	UK	lineage4	S	S	S	S	S	S	S	S
13.0602075	Birmingham	UK	Delhi	S	S	S	S	S	S	S	S
16.0601046	Birmingham	UK	LAM	S	S	S	S	S	S	S	S
14.0600970	Birmingham	UK	EAI	S	S	S	S	S	S	S	S
13.0607959	Birmingham	UK	EAI	S	S	S	S	S	S	S	S
15.0607778	Birmingham	UK	lineage4	S	S	S	S	S	S	S	S
14.0600566	Birmingham	UK	Delhi	S	S	S	S	S	S	S	S
14.0600788	Birmingham	UK	Delhi	S	S	S	S	S	S	S	S
14.0609149	Birmingham	UK	lineage4	S	S	S	S	S	S	S	S
16.0604778	Birmingham	UK	Delhi	S	S	S	S	S	R	S	S
15.0601597	Birmingham	UK	lineage4	S	S	S	S	S	S	S	S
13.0604889	Birmingham	UK	Delhi	S	S	S	S	S	S	S	S
16.0607766	Birmingham	UK	M. orygis	S	S	S	S	S	S	S	S
15.0607140	Birmingham	UK	Haarlem	S	S	S	S	S	S	S	S
17.0601058	Birmingham	UK	Delhi	S	S	S	S	S	S	S	S
15.0607298	Birmingham	UK	Delhi	S	S	S	S	S	S	U	S
16.0601922	Birmingham	UK	Delhi	R	S	S	S	R	S	S	S
14.0600259	Birmingham	UK	EAI	S	S	S	S	S	S	U	S
15.0613282	Birmingham	UK	Delhi	S	S	S	S	S	S	S	S
15.0606897	Birmingham	UK	Cameroon	S	S	S	S	S	S	S	S
14.0600802	Birmingham	UK	LAM	S	S	S	S	S	S	S	S
11.0604393	Birmingham	UK	M. bovis	S	S	S	R	S	S	U	R
13.0610746	Birmingham	UK	Delhi	S	S	S	S	S	S	S	S
13.0611884	Birmingham	UK	LAM	S	S	S	S	S	S	S	S
15.0601833	Birmingham	UK	LAM	S	S	S	S	S	S	S	S
14.0600692	Birmingham	UK	S-type	S	S	S	S	S	S	S	S
09.0602214	Birmingham	UK	Beijing	S	S	S	S	S	S	S	S
15.0607446	Birmingham	UK	LAM	S	S	S	S	S	S	S	S
13.0606481	Birmingham	UK	lineage4	S	S	S	S	S	S	S	S
09.0600998	Birmingham	UK	Delhi	S	S	S	S	S	S	U	S
16.0607570	Birmingham	UK	X-type	S	S	S	S	S	S	U	S
14.0606979	Birmingham	UK	Beijing	n/a	n/a	n/a	S	R	F	R	S
16.0611103	Birmingham	UK	lineage4	S	S	S	S	S	S	S	S
13.0605713	Birmingham	UK	Delhi	S	S	S	S	S	S	S	S
12.0615273	Birmingham	UK	EAI	S	S	S	S	S	S	U	S
13.0600614	Birmingham	UK	LAM	S	S	S	S	S	S	U	S
13.0613605	Birmingham	UK	Delhi	S	S	S	S	S	S	S	S
15.0601457	Birmingham	UK	EAI	S	S	n/a	S	S	S	S	S
13.0609970	Birmingham	UK	Delhi	S	S	S	S	S	S	S	S
12.0615237	Birmingham	UK	Delhi	S	S	S	S	S	S	S	S
17.0601028	Birmingham	UK	Delhi	S	S	S	n/a	S	S	S	S
12.0610321	Birmingham	UK	EAI	S	S	S	S	S	S	S	S
14.0609538	Birmingham	UK	Delhi	S	S	S	S	S	S	S	S
14.0609546	Birmingham	UK	Delhi	S	S	S	S	S	S	S	S
11.0614819	Birmingham	UK	Delhi	S	S	S	S	S	S	S	S
14.0609727	Birmingham	UK	Delhi	S	S	S	S	S	S	S	S
13.0604065	Birmingham	UK	LAM	S	S	S	S	S	S	S	S
14.0609402	Birmingham	UK	EAI	S	S	S	S	S	S	S	S
13.0611312	Birmingham	UK	lineage4	S	S	S	S	S	S	S	S
16.0601463	Birmingham	UK	X-type	S	S	S	S	S	S	S	S
14.0616130	Birmingham	UK	EAI	S	S	S	S	S	S	S	S
14.0614975	Birmingham	UK	Delhi	R	S	S	S	R	S	S	S
12.0613586	Birmingham	UK	LAM	S	S	S	S	S	S	S	S
13.0610905	Birmingham	UK	lineage4	S	S	S	S	S	S	S	S
14.0600022	Birmingham	UK	Delhi	S	S	S	S	S	S	S	S
14.0616019	Birmingham	UK	lineage4	S	S	S	S	S	S	S	S
16.0601118	Birmingham	UK	Beijing	S	S	S	S	S	S	S	S
09.0600120	Birmingham	UK	lineage4	S	S	S	S	S	S	S	S
12.0617129	Birmingham	UK	lineage4	S	S	S	S	S	S	U	S
12.0612858	Birmingham	UK	EAI	S	S	S	S	S	S	S	S
16.0617216	Birmingham	UK	Delhi	S	S	S	S	S	S	S	S
15.0611783	Birmingham	UK	Haarlem	S	S	S	S	S	S	S	S
09.0604962	Birmingham	UK	EAI	R	S	S	S	R	S	S	S
15.0614226	Birmingham	UK		S	S	S	S	S	S	S	S
15.0601694	Birmingham	UK	Delhi	S	S	S	S	S	S	S	S
12.0608968	Birmingham	UK	Delhi	S	S	S	S	S	S	S	S
14.0609027	Birmingham	UK	Haarlem	S	S	S	S	S	S	S	S
15.0607678	Birmingham	UK	Delhi	S	S	S	S	S	S	S	S
15.0607388	Birmingham	UK	Delhi	S	S	S	S	F	S	S	S
15.0613052	Birmingham	UK	Delhi	S	S	S	S	S	S	S	S
14.0604927	Birmingham	UK	Delhi	S	S	S	S	S	S	S	S
16.0601967	Birmingham	UK	lineage4	n/a	n/a	n/a	S	S	S	S	S
14.0613979	Birmingham	UK	Delhi	S	S	S	S	S	S	S	S
13.0611291	Birmingham	UK	Delhi	S	S	S	S	S	S	S	S
15.0607409	Birmingham	UK	Delhi	S	S	S	S	S	U	S	F
13.0612513	Birmingham	UK	S-type	S	S	S	S	S	S	S	S
14.0609903	Birmingham	UK	Delhi	S	S	S	S	S	S	S	S
16.0618469	Birmingham	UK	EAI	S	S	S	S	S	S	S	S
13.0603425	Birmingham	UK	EAI	R	S	S	S	R	S	S	S
14.0609854	Birmingham	UK	Delhi	S	S	S	S	S	S	S	S
12.0927646	Birmingham	UK	lineage4	R	S	S	S	R	S	S	S
13.0611316	Birmingham	UK	Delhi	R	S	S	S	R	S	S	S
15.0607745	Birmingham	UK	LAM	R	R	R	R	R	R	R	U
13.0613458	Birmingham	UK	Delhi	S	S	S	S	S	S	S	S
15.0601542	Birmingham	UK		S	S	S	S	S	S	S	S
15.0613506	Birmingham	UK	Delhi	S	S	S	S	S	S	S	S
13.0609498	Birmingham	UK	EAI	S	S	S	S	S	S	S	S
15.0601607	Birmingham	UK	Haarlem	S	S	S	S	S	S	S	S
15.0601876	Birmingham	UK	Delhi	R	R	S	S	R	R	S	S
15.0615367	Birmingham	UK	Delhi	S	S	S	S	S	S	S	S
16.0603276	Birmingham	UK	Beijing	S	S	S	S	S	S	S	S
15.0607254	Birmingham	UK	lineage4	S	S	S	S	S	S	S	S
14.0600273	Birmingham	UK	EAI	S	S	S	S	S	S	S	S
16.0618329	Birmingham	UK	Ural	S	S	S	S	S	S	S	S
13.0609069	Birmingham	UK	lineage4	S	S	S	S	S	S	S	S
10.0605084	Birmingham	UK	EAI	S	S	S	S	U	S	S	S
16.0601224	Birmingham	UK	X-type	R	S	S	S	R	S	S	S
15.0607511	Birmingham	UK	LAM	S	S	S	S	S	S	S	S
10.0611022	Birmingham	UK	Delhi	S	S	S	S	S	S	S	S
13.0601556	Birmingham	UK	Delhi	S	S	S	S	S	S	S	S
16.0601593	Birmingham	UK	lineage4	S	S	S	S	S	S	S	S
14.0600014	Birmingham	UK	Delhi	S	S	S	S	S	S	S	S
14.0609976	Birmingham	UK	LAM	S	S	S	S	S	F	S	S
16.0604915	Birmingham	UK	Beijing	R	R	S	n/a	R	R	S	F
11.0611394	Birmingham	UK	Delhi	S	S	S	S	S	S	S	S
13.0605101	Birmingham	UK	Delhi	S	S	S	S	S	S	S	S
16.0607436	Birmingham	UK	Haarlem	S	S	S	S	S	S	S	S
13.0606112	Birmingham	UK	Haarlem	S	S	S	S	S	S	S	S
14.0609261	Birmingham	UK	lineage4	S	S	S	S	S	S	S	S
15.0601450	Birmingham	UK	lineage4	S	S	S	S	S	S	S	S
14.0609534	Birmingham	UK	Delhi	S	S	S	S	S	S	S	S
14.0600675	Birmingham	UK	X-type	S	S	S	S	S	S	U	S
16.0618092	Birmingham	UK	Delhi	S	S	S	S	S	F	S	S

15.0601907	Birmingham	UK	EAI	S	S	S	S	U	S	U	S
10.0612351	Birmingham	UK	lineage4	S	S	S	S	S	S	S	S
15.0613245	Birmingham	UK	M. bovis	S	S	S	n/a	S	S	S	R
15.0601037	Birmingham	UK	Delhi	S	S	S	S	S	S	S	S
15.0607013	Birmingham	UK	Delhi	S	S	S	S	U	S	S	S
16.0605800	Birmingham	UK	Beijing	S	S	S	S	S	S	S	S
15.0607259	Birmingham	UK	Delhi	S	S	S	S	S	S	S	S
16.0601755	Birmingham	UK	EAI	S	S	S	S	S	U	S	S
16.0607810	Birmingham	UK	Delhi	S	S	S	S	S	S	S	S
15.0607382	Birmingham	UK	M. orygis	S	S	S	S	S	S	S	S
15.0601594	Birmingham	UK	Delhi	S	S	S	S	S	S	S	S
10.0612476	Birmingham	UK	X-type	S	S	S	S	S	S	S	S
13.0601939	Birmingham	UK	lineage4	S	S	S	S	S	S	S	S
13.0600593	Birmingham	UK	lineage4	S	S	S	S	S	S	S	S
16.0617634	Birmingham	UK	Delhi	S	S	S	S	S	F	S	F
12.0616803	Birmingham	UK	X-type	S	S	S	R	F	F	R	F
15.0609692	Birmingham	UK	Delhi	S	S	S	S	S	S	S	S
15.0601390	Birmingham	UK	lineage4	S	S	S	S	S	S	S	F
15.0609939	Birmingham	UK	Delhi	S	S	S	S	S	S	S	S
13.0605679	Birmingham	UK	Beijing	S	S	S	S	S	S	S	S
13.0613231	Birmingham	UK	Haarlem	S	S	S	S	S	S	S	S
13.0607363	Birmingham	UK	Haarlem	S	S	S	S	S	S	S	S
14.0609461	Birmingham	UK	LAM	S	S	S	S	F	S	F	S
16.0618063	Birmingham	UK	Haarlem	S	S	S	S	S	S	S	S
13.0611349	Birmingham	UK	lineage4	S	S	S	S	S	S	S	S
14.0611995	Birmingham	UK	Delhi	S	S	S	S	S	S	S	S
14.0609672	Birmingham	UK	Delhi	S	S	S	S	S	S	S	S
16.0607206	Birmingham	UK		S	S	S	S	S	S	S	S
13.0607687	Birmingham	UK	Delhi	S	S	S	S	S	S	S	S
17.0601169	Birmingham	UK	Beijing	R	S	S	S	R	S	S	S
12.0611665	Birmingham	UK	LAM	S	S	S	S	S	S	S	S
16.0607214	Birmingham	UK	lineage4	S	S	S	S	S	S	S	S
15.0602264	Birmingham	UK	Delhi	S	S	S	S	S	S	S	S
14.0600549	Birmingham	UK	LAM	S	S	S	S	S	S	S	S
14.0609609	Birmingham	UK	Delhi	S	S	S	S	S	S	S	S
12.0611829	Birmingham	UK	X-type	S	S	S	S	S	S	S	S
15.0601078	Birmingham	UK	EAI	S	S	S	S	S	S	S	S
16.0607081	Birmingham	UK	lineage4	S	S	S	S	S	S	S	S
15.0601691	Birmingham	UK	Beijing	S	S	S	S	S	S	U	S
09.0609881	Birmingham	UK	Delhi	S	S	S	S	S	S	U	S
16.0601004	Birmingham	UK	lineage4	S	S	S	S	S	S	S	S
16.0606527	Birmingham	UK	EAI	S	S	n/a	n/a	U	S	S	S
16.0609380	Birmingham	UK	EAI	S	S	S	n/a	S	S	U	S
15.0607211	Birmingham	UK	Delhi	S	S	S	S	S	S	U	S
15.0604533	Birmingham	UK	Haarlem	S	S	S	S	S	S	S	S
14.0609663	Birmingham	UK	X-type	S	S	S	S	S	S	S	S
16.0601743	Birmingham	UK	EAI	S	S	S	n/a	F	F	F	F
14.0600051	Birmingham	UK	Delhi	S	S	S	S	S	S	U	S
13.0603879	Birmingham	UK	Beijing	S	S	S	S	S	S	U	S
17.0601031	Birmingham	UK	Delhi	n/a	S	n/a	S	S	S	S	S
16.0601459	Birmingham	UK	lineage4	S	S	S	S	S	F	S	S
14.0600094	Birmingham	UK	lineage4	S	S	S	S	S	S	S	S
12.0610932	Birmingham	UK	Delhi	S	S	S	S	S	S	S	S
13.0609918	Birmingham	UK	Cameroon	S	S	S	S	S	S	S	S
15.0607307	Birmingham	UK	Delhi	S	S	S	S	S	S	U	S
14.0609088	Birmingham	UK	EAI	S	S	S	S	S	S	S	S
16.0600206	Birmingham	UK	lineage4	S	S	S	S	S	F	U	S
15.0613137	Birmingham	UK	EAI	R	S	S	S	R	S	S	S
14.0616036	Birmingham	UK	Cameroon	S	S	S	S	S	S	S	S
12.0611823	Birmingham	UK	EAI	S	S	S	S	S	S	S	S
16.0618370	Birmingham	UK	Haarlem	S	S	S	S	S	S	S	S
16.0601107	Birmingham	UK	lineage4	S	S	S	S	S	S	S	S
15.0607245	Birmingham	UK	Delhi	S	S	S	S	S	S	S	S
16.0607918	Birmingham	UK	lineage4	S	S	S	S	S	S	S	S
12.0611612	Birmingham	UK	EAI	S	S	S	S	S	S	S	S
15.0601100	Birmingham	UK	EAI	S	S	S	S	S	S	S	S
12.0612540	Birmingham	UK	X-type	S	S	S	S	S	S	S	S
16.0618357	Birmingham	UK	lineage4	S	S	S	S	S	S	S	S
16.0618564	Birmingham	UK	Ural	S	S	S	S	S	S	S	S
16.0601379	Birmingham	UK	Delhi	R	S	n/a	S	R	S	U	S
13.0612058	Birmingham	UK	Delhi	S	S	S	S	S	S	U	S
16.0601803	Birmingham	UK	Haarlem	S	S	S	n/a	S	S	S	S
14.0614437	Birmingham	UK	lineage4	S	S	S	S	S	S	U	S
15.0609506	Birmingham	UK	Beijing	S	S	S	S	S	S	U	S
16.0604851	Birmingham	UK	Haarlem	S	S	S	S	S	S	S	S
15.0601539	Birmingham	UK	Beijing	S	S	S	S	S	S	S	S
13.0609880	Birmingham	UK	LAM	S	S	S	S	S	S	S	S
15.0600494	Birmingham	UK	lineage4	S	S	S	S	S	S	S	S
16.0602155	Birmingham	UK	LAM	S	S	S	S	S	S	S	S
13.0601204	Birmingham	UK	Haarlem	S	S	S	S	S	S	S	S
15.0613226	Birmingham	UK	Beijing	S	S	S	S	S	S	S	S
16.0607904	Birmingham	UK	EAI	S	S	S	S	S	S	S	S
14.0600894	Birmingham	UK	lineage4	S	S	S	S	S	S	S	S
12.0611899	Birmingham	UK	LAM	S	S	S	S	S	S	S	S
16.0614933	Birmingham	UK	Delhi	S	S	S	S	S	S	S	S
14.0600344	Birmingham	UK	EAI	S	S	S	S	S	S	S	S
12.0612512	Birmingham	UK	EAI	S	S	S	S	U	S	S	S
13.0610745	Birmingham	UK	Haarlem	S	S	S	S	S	S	S	S
13.0610275	Birmingham	UK	Delhi	S	S	S	S	S	S	S	S
16.0610558	Birmingham	UK	Delhi	S	S	S	S	S	S	S	S
17.0601057	Birmingham	UK	Tur	S	S	S	S	S	S	S	S
15.0601618	Birmingham	UK	lineage4	S	S	S	S	S	S	S	S
14.0600660	Birmingham	UK	EAI	S	S	S	S	S	S	S	S
15.0601869	Birmingham	UK	Beijing	R	S	S	S	R	S	S	S
12.0614990	Birmingham	UK	LAM	S	S	S	S	S	S	S	S
15.0609125	Birmingham	UK	EAI	n/a	S	S	S	R	S	S	S
13.0610401	Birmingham	UK	Delhi	S	S	S	S	S	S	S	S
13.0613495	Birmingham	UK	Delhi	S	S	S	S	S	S	S	S
16.0617376	Birmingham	UK	Delhi	S	S	S	S	U	S	S	S
15.0613500	Birmingham	UK	EAI	S	S	S	S	U	S	S	S
16.0601572	Birmingham	UK	Delhi	S	S	S	S	S	S	S	S
13.0608970	Birmingham	UK	Delhi	R	S	S	S	R	R	R	F
14.0609932	Birmingham	UK	LAM	S	S	S	S	S	S	S	S
13.0609922	Birmingham	UK	lineage4	S	S	S	S	S	S	S	S
14.0600845	Birmingham	UK	lineage4	S	S	S	S	S	S	S	S
16.0610187	Birmingham	UK	EAI	S	S	S	n/a	S	S	S	S
13.0612197	Birmingham	UK	X-type	S	S	S	S	S	S	S	S
16.0607002	Birmingham	UK	Beijing	S	S	S	n/a	S	S	S	S
16.0601223	Birmingham	UK	lineage4	S	S	S	S	S	S	S	S
14.0600359	Birmingham	UK	Delhi	S	S	S	S	S	S	S	S
13.0609556	Birmingham	UK	Delhi	S	S	S	S	S	S	S	S
16.0607677	Birmingham	UK	M. orygis	S	S	S	S	S	S	S	S
16.0605525	Birmingham	UK	lineage4	S	S	S	S	S	S	S	S
15.0607521	Birmingham	UK	Delhi	S	S	S	n/a	S	S	S	S
16.0607549	Birmingham	UK	Delhi	n/a	S	S	n/a	S	S	S	S
15.0601328	Birmingham	UK	Delhi	S	S	S	S	S	S	S	S

15.0601922	Birmingham	UK	lineage4	S	S	S	n/a	S	S	U	S
14.0609934	Birmingham	UK	Delhi	S	S	S	S	S	S	S	S
16.0607339	Birmingham	UK	LAM	S	S	S	S	S	S	S	S
16.0601930	Birmingham	UK	Delhi	S	S	S	S	S	S	S	S
16.0601483	Birmingham	UK	Delhi	S	S	S	S	S	S	S	S
12.0610289	Birmingham	UK	X-type	S	S	S	S	S	S	S	S
14.0609496	Birmingham	UK	EAI	R	S	S	S	R	S	S	S
16.0601182	Birmingham	UK	S-type	S	S	S	S	S	S	S	S
16.0609975	Birmingham	UK	Delhi	S	S	S	S	S	S	S	S
15.0615577	Birmingham	UK	S-type	S	S	S	S	S	U	U	S
16.0617037	Birmingham	UK	Delhi	S	S	S	S	S	S	S	S
15.0607414	Birmingham	UK	Delhi	S	S	S	S	S	S	S	S
13.0612468	Birmingham	UK	EAI	S	S	S	S	S	S	S	S
16.0607576	Birmingham	UK	lineage4	S	S	S	S	R	S	S	S
14.0617126	Birmingham	UK	Delhi	S	S	S	S	S	U	S	S
15.0606017	Birmingham	UK	Haarlem	S	S	S	S	S	S	U	S
12.0617193	Birmingham	UK	EAI	S	S	S	S	U	S	U	S
09.0600078	Birmingham	UK	EAI	S	S	S	S	S	S	S	S
15.0601227	Birmingham	UK	Delhi	S	S	S	S	S	S	S	S
15.0607540	Birmingham	UK	EAI	R	R	S	S	R	R	S	S
15.0607875	Birmingham	UK	Delhi	S	S	S	S	S	S	S	S
15.0606178	Birmingham	UK	Delhi	S	S	S	S	S	S	S	S
16.0618507	Birmingham	UK	Haarlem	S	S	S	S	S	S	S	S
15.0607917	Birmingham	UK	Delhi	S	S	S	S	S	S	S	S
14.0609983	Birmingham	UK	lineage4	S	S	S	S	S	S	S	S
15.0607331	Birmingham	UK	S-type	R	S	S	S	R	S	S	S
16.0617705	Birmingham	UK	Delhi	R	R	R	S	R	R	R	S
14.0609729	Birmingham	UK	lineage4	S	S	S	S	S	S	S	S
13.0617839	Birmingham	UK	lineage4	S	S	S	S	S	S	S	S
16.0601207	Birmingham	UK	lineage4	S	S	S	S	S	S	S	S
13.0607146	Birmingham	UK	lineage4	S	S	S	S	S	S	S	S
14.0609809	Birmingham	UK	Delhi	R	S	S	S	R	S	S	S
16.0604924	Birmingham	UK	Ural	S	S	S	S	S	S	S	S
15.0601196	Birmingham	UK	X-type	S	S	S	S	S	S	S	F
16.0601262	Birmingham	UK	LAM	S	S	S	S	S	S	S	S
16.0607760	Birmingham	UK	lineage4	S	S	S	n/a	S	S	S	S
15.0613386	Birmingham	UK	EAI	S	S	S	S	S	S	S	S
14.0609017	Birmingham	UK	LAM	S	S	S	S	S	S	S	S
14.0600126	Birmingham	UK	Delhi	S	S	S	S	S	S	S	S
12.0612305	Birmingham	UK	EAI	S	S	S	S	S	S	S	S
17.0601034	Birmingham	UK	lineage4	S	S	S	S	S	F	S	S
16.0607951	Birmingham	UK	Delhi	S	S	S	S	S	S	S	S
13.0605712	Birmingham	UK	LAM	S	S	S	S	S	S	S	S
13.0604544	Birmingham	UK	Beijing	S	S	S	S	S	S	S	S
11.0608173	Birmingham	UK	EAI	S	S	S	S	S	S	S	S
11.0603999	Birmingham	UK		S	S	S	S	S	S	S	S
10.0607425	Birmingham	UK	Tur	S	S	S	S	S	S	S	S
13.0607670	Birmingham	UK	Delhi	S	S	S	S	S	S	S	S
16.0607439	Birmingham	UK		S	S	S	S	U	S	S	S
13.0610363	Birmingham	UK	Delhi	S	S	S	n/a	S	S	S	S
14.0600813	Birmingham	UK	Beijing	S	S	S	S	S	S	U	S
15.0607533	Birmingham	UK	Beijing	R	S	S	S	R	S	S	S
13.0611336	Birmingham	UK	Delhi	S	S	S	S	S	S	S	S
11.0608782	Birmingham	UK	Delhi	S	S	S	S	S	S	S	S
12.0615293	Birmingham	UK	Delhi	S	S	S	S	S	S	S	S
14.0609039	Birmingham	UK	S-type	S	S	S	S	S	S	S	S
14.0600828	Birmingham	UK	lineage4	S	S	S	S	S	S	S	S
13.0613598	Birmingham	UK	Haarlem	S	S	S	S	S	S	S	S
15.0607233	Birmingham	UK	EAI	S	S	S	S	S	S	S	S
14.0600127	Birmingham	UK	Delhi	S	S	S	S	S	S	S	S
16.0604337	Birmingham	UK	lineage4	S	S	S	S	S	S	S	S
16.0607526	Birmingham	UK		S	S	S	S	S	S	S	S
13.0601244	Birmingham	UK	LAM	S	S	S	S	S	S	S	S
16.0601437	Birmingham	UK	Delhi	S	S	S	S	S	S	S	S
11.0609893	Birmingham	UK	lineage4	S	S	S	S	S	U	S	S
14.0600180	Birmingham	UK	Haarlem	S	S	S	S	S	S	S	S
13.0613395	Birmingham	UK	EAI	S	S	S	S	S	S	S	S
16.0601884	Birmingham	UK	Delhi	S	S	S	S	S	S	S	S
16.0618115	Birmingham	UK	Haarlem	S	S	S	S	S	S	U	S
13.0603107	Birmingham	UK	Beijing	S	S	S	S	S	S	S	S
16.0606652	Birmingham	UK	LAM	S	S	S	S	S	S	S	S
15.0610103	Birmingham	UK	lineage4	S	S	S	S	U	S	S	S
16.0601637	Birmingham	UK	Ural	S	S	S	S	S	S	S	S
14.0610425	Birmingham	UK	Delhi	S	S	S	S	S	S	S	S
13.0610114	Birmingham	UK	Delhi	S	S	S	S	S	S	S	S
14.0603544	Birmingham	UK	LAM	R	S	S	S	R	S	S	S
14.0600448	Birmingham	UK	EAI	S	S	S	S	S	F	S	S
13.0613501	Birmingham	UK	Haarlem	S	S	S	S	S	S	S	S
16.0618365	Birmingham	UK	Cameroon	S	S	S	S	S	S	S	S
15.0607116	Birmingham	UK	lineage4	S	S	S	S	S	S	S	S
12.0611226	Birmingham	UK	Haarlem	S	S	S	S	S	S	S	S
14.0609305	Birmingham	UK	EAI	S	S	S	S	S	S	S	S
16.0607889	Birmingham	UK	Delhi	S	S	S	n/a	S	S	S	S
14.0605039	Birmingham	UK	lineage4	S	S	S	S	S	S	S	S
12.0927695	Birmingham	UK	EAI	S	S	S	S	F	S	S	S
14.0600311	Birmingham	UK	Beijing	S	S	S	S	S	S	S	S
14.0605971	Birmingham	UK		S	S	S	S	S	S	S	S
15.0601724	Birmingham	UK	Delhi	S	S	S	S	S	S	U	S
16.0601581	Birmingham	UK	Delhi	S	S	S	S	S	S	S	S
14.0609193	Birmingham	UK	EAI	S	S	S	S	U	S	S	S
16.0617618	Birmingham	UK	Haarlem	S	S	S	S	S	S	S	S
15.0614762	Birmingham	UK	Delhi	S	S	S	n/a	S	S	S	S
13.0604046	Birmingham	UK	Beijing	S	S	S	S	S	S	S	S
16.0607005	Birmingham	UK	EAI	S	S	S	n/a	S	S	S	S
14.0611757	Birmingham	UK	Delhi	S	S	S	S	U	S	S	S
15.0605050	Birmingham	UK	Beijing	S	S	S	S	S	S	S	S
15.0607647	Birmingham	UK	EAI	S	S	S	S	S	S	S	S
15.0601579	Birmingham	UK	EAI	S	S	S	S	U	S	U	S
15.0607339	Birmingham	UK	LAM	S	S	S	S	S	S	S	S
12.0611796	Birmingham	UK	Haarlem	S	S	S	S	S	S	S	S
15.0601916	Birmingham	UK	lineage4	S	S	S	S	S	S	S	S
14.0609754	Birmingham	UK	EAI	S	S	S	S	S	S	U	S
14.0600083	Birmingham	UK	Delhi	S	S	S	S	S	S	S	S
15.0607220	Birmingham	UK	Beijing	S	S	S	S	F	F	F	F
16.0601482	Birmingham	UK	Haarlem	S	S	S	S	S	F	F	S
14.0604083	Birmingham	UK	lineage4	S	S	S	S	S	S	S	S
16.0607150	Birmingham	UK	X-type	n/a	n/a	n/a	S	S	S	S	S
16.0617061	Birmingham	UK	Tur	S	S	S	S	S	S	S	S
15.0609819	Birmingham	UK	lineage4	S	S	S	S	S	S	S	S
09.0603467	Birmingham	UK	Tur	S	S	S	S	S	F	S	S
13.0606478	Birmingham	UK	Beijing	S	S	S	S	S	S	S	S
13.0612756	Birmingham	UK	X-type	S	S	S	S	S	S	S	S
15.0601714	Birmingham	UK	Beijing	S	S	S	S	S	S	S	S
13.0604076	Birmingham	UK	Delhi	S	S	S	S	S	S	S	S
15.0607332	Birmingham	UK	lineage4	S	S	S	S	S	S	S	S
12.0609354	Birmingham	UK	Delhi	S	S	S	S	S	S	S	S

15.0613013	Birmingham	UK	Haarlem	S	S	S	S	S	S	S	S
15.0601184	Birmingham	UK	Delhi	S	S	S	S	S	S	S	S
13.0614470	Birmingham	UK	Delhi	S	S	S	S	S	S	S	S
13.0604885	Birmingham	UK	Beijing	S	S	S	S	S	S	S	S
16.0618451	Birmingham	UK	BCG	S	S	S	R	S	S	S	R
13.0614325	Birmingham	UK	Delhi	S	S	S	S	S	S	S	S
15.0611234	Birmingham	UK	Haarlem	S	S	S	S	S	S	S	S
15.0607304	Birmingham	UK	LAM	S	S	S	S	S	S	S	S
14.0609878	Birmingham	UK	lineage4	S	S	S	S	S	U	S	S
12.0612864	Birmingham	UK	Delhi	S	S	S	S	S	S	S	S
16.0617186	Birmingham	UK	Delhi	S	S	S	S	S	S	S	S
16.0602435	Birmingham	UK	Delhi	S	S	S	S	S	S	S	S
16.0608037	Birmingham	UK	M. bovis	S	S	S	R	S	S	S	R
14.0617424	Birmingham	UK	lineage4	S	S	S	S	S	S	S	S
11.0613488	Birmingham	UK	Delhi	S	S	S	S	S	S	S	S
15.0613473	Birmingham	UK	lineage4	S	S	S	S	S	S	S	S
15.0613290	Birmingham	UK	Delhi	S	S	S	S	S	S	S	S
15.0607145	Birmingham	UK	EAI	S	S	S	S	S	S	S	S
16.0607171	Birmingham	UK	Delhi	S	S	S	S	S	S	S	S
16.0607580	Birmingham	UK	Delhi	S	S	S	S	U	S	S	S
13.0601530	Birmingham	UK	Beijing	R	S	S	S	R	S	S	S
15.0613325	Birmingham	UK	LAM	S	S	S	S	S	S	S	S
13.0615544	Birmingham	UK		S	S	S	S	S	S	S	S
13.0612923	Birmingham	UK	EAI	S	S	S	S	S	S	S	S
13.0613027	Birmingham	UK	LAM	S	S	S	S	S	S	S	S
13.0604078	Birmingham	UK	Delhi	S	S	S	S	S	S	S	S
12.0617748	Birmingham	UK	Delhi	S	S	S	S	S	S	S	S
16.0601561	Birmingham	UK	Beijing	R	S	S	S	R	S	S	S
15.0607810	Birmingham	UK	Delhi	S	S	S	S	S	S	U	S
16.0601183	Birmingham	UK	Delhi	S	S	S	S	S	S	S	S
14.0600162	Birmingham	UK	Haarlem	S	S	S	S	S	S	S	S
13.0605715	Birmingham	UK	Beijing	S	S	S	S	S	S	S	S
16.0601899	Birmingham	UK	Delhi	S	S	S	S	S	S	S	S
16.0618062	Birmingham	UK	Haarlem	S	S	S	S	S	S	S	S
14.0609882	Birmingham	UK	S-type	S	S	S	S	S	S	S	S
16.0601782	Birmingham	UK	LAM	S	S	S	S	S	F	S	S
15.0601222	Birmingham	UK	lineage4	S	S	S	S	S	S	S	S
12.0613175	Birmingham	UK	Delhi	S	S	S	S	S	S	S	S
17.0601017	Birmingham	UK	Beijing	S	S	S	S	S	S	U	S
16.0617288	Birmingham	UK	Beijing	S	S	S	S	S	S	S	S
16.0601903	Birmingham	UK	EAI	S	S	S	n/a	S	S	S	S
15.0605986	Birmingham	UK	LAM	S	S	S	S	S	S	U	S
13.0606187	Birmingham	UK	EAI	S	S	S	S	S	S	S	S
13.0602541	Birmingham	UK	lineage4	S	S	S	S	S	S	U	S
16.0617144	Birmingham	UK	LAM	S	S	S	S	S	S	S	S
13.0612189	Birmingham	UK	Delhi	S	S	S	S	S	S	S	S
16.0607990	Birmingham	UK	EAI	S	S	S	n/a	S	S	S	S
15.0601656	Birmingham	UK	Tur	S	S	S	S	S	S	S	S
15.0607043	Birmingham	UK	lineage4	S	S	S	S	S	S	S	S
13.0613404	Birmingham	UK	EAI	S	S	S	S	S	S	S	S
16.0608489	Birmingham	UK	Beijing	S	S	S	S	S	S	S	S
16.0601039	Birmingham	UK	S-type	S	S	S	S	S	S	S	S
14.0616095	Birmingham	UK	Delhi	R	R	S	n/a	R	R	R	R
13.0613044	Birmingham	UK	Delhi	S	S	S	S	S	S	S	S
16.0601659	Birmingham	UK	Delhi	S	S	S	S	S	S	S	S
09.0600182	Birmingham	UK	Delhi	S	S	S	S	S	S	S	S
15.0607831	Birmingham	UK	lineage4	S	S	S	S	S	S	S	S
13.0604169	Birmingham	UK	Delhi	S	S	S	S	S	S	S	S
12.0611439	Birmingham	UK	Delhi	S	S	S	S	S	S	S	S
15.0601334	Birmingham	UK	Delhi	S	S	S	S	F	S	S	F
16.0601105	Birmingham	UK	Cameroon	R	S	S	S	R	S	S	S
12.0927462	Birmingham	UK	Delhi	S	S	S	S	S	S	S	S
14.0600078	Birmingham	UK	Haarlem	S	S	S	S	S	S	S	S
13.0609436	Birmingham	UK	Delhi	S	S	S	S	S	S	S	S
13.0608450	Birmingham	UK	Delhi	S	S	S	S	S	S	S	S
15.0607803	Birmingham	UK	lineage4	S	S	S	S	S	S	S	S
16.0601055	Birmingham	UK	Haarlem	n/a	S	S	n/a	F	S	F	S
15.0613281	Birmingham	UK	LAM	S	S	S	S	S	S	S	S
15.0613076	Birmingham	UK	Delhi	S	S	S	S	S	S	S	S
15.0601401	Birmingham	UK	lineage4	n/a	n/a	n/a	S	S	S	S	S
13.0607968	Birmingham	UK	lineage4	S	S	S	S	S	S	S	S
14.0600123	Birmingham	UK	LAM	S	S	S	S	S	S	S	S
16.0604979	Birmingham	UK	Tur	S	S	S	S	S	S	S	S
13.0604530	Birmingham	UK	lineage4	S	S	S	S	S	S	S	S
16.0604235	Birmingham	UK	LAM	S	S	S	S	S	S	S	S
16.0601240	Birmingham	UK	lineage4	R	S	S	S	R	S	S	S
12.0612658	Birmingham	UK	Beijing	S	S	S	S	S	S	S	S
14.0600799	Birmingham	UK	lineage4	S	S	S	S	S	S	S	S
12.0613594	Birmingham	UK	lineage4	S	S	S	S	S	S	S	S
16.0601919	Birmingham	UK	LAM	S	S	S	S	S	S	S	S
09.0603647	Birmingham	UK	EAI	R	S	S	S	R	S	S	S
15.0607733	Birmingham	UK	Beijing	R	R	R	R	R	R	R	R
13.0604687	Birmingham	UK	Delhi	S	S	S	S	S	S	S	S
13.0611031	Birmingham	UK	lineage4	S	S	S	S	S	S	S	S
12.0608505	Birmingham	UK	lineage4	S	S	S	S	S	S	S	S
16.0607952	Birmingham	UK	Beijing	S	S	S	S	S	S	U	S
13.0613527	Birmingham	UK	Delhi	S	S	S	S	S	S	S	S
13.0607131	Birmingham	UK	Delhi	S	S	S	S	S	S	S	S
14.0603394	Birmingham	UK	Delhi	S	S	S	S	S	S	S	S
13.0617739	Birmingham	UK	lineage4	S	S	S	S	S	S	S	S
15.0603913	Birmingham	UK	Delhi	S	S	S	S	S	S	U	S
15.0607340	Birmingham	UK	Tur	R	S	S	S	R	S	S	S
15.0607006	Birmingham	UK	X-type	S	S	S	S	S	S	S	S
14.0607298	Birmingham	UK	LAM	S	S	S	S	S	S	S	S
16.0607338	Birmingham	UK	EAI	S	S	S	S	S	S	S	S
16.0608352	Birmingham	UK	lineage4	S	S	S	S	S	S	S	S
12.0611207	Birmingham	UK	lineage4	S	S	S	S	S	S	S	S
12.0927694	Birmingham	UK	Tur	S	S	S	S	S	S	S	S
15.0601166	Birmingham	UK	LAM	S	S	S	S	S	S	S	S
12.0606314	Birmingham	UK	Delhi	S	S	S	S	S	S	S	S
15.0607904	Birmingham	UK	Haarlem	S	S	S	S	S	S	S	S
16.0607086	Birmingham	UK	Delhi	S	S	S	S	S	S	S	S
12.0613159	Birmingham	UK	lineage4	S	S	S	S	S	S	S	S
15.0607764	Birmingham	UK	Delhi	R	S	S	S	R	S	S	S
14.0609013	Birmingham	UK	X-type	S	S	S	S	S	S	U	S
16.0612109	Birmingham	UK	lineage4	S	S	S	S	S	S	S	S
16.0607112	Birmingham	UK	Delhi	S	S	S	S	S	U	S	S
13.0610506	Birmingham	UK	S-type	R	S	S	S	R	S	S	S
16.0601235	Birmingham	UK	LAM	S	S	S	S	S	S	S	S
14.0604540	Birmingham	UK	LAM	S	S	S	S	S	S	S	S
14.0609173	Birmingham	UK		S	S	S	S	U	S	S	S
13.0603123	Birmingham	UK	lineage4	S	S	S	S	S	S	S	S
16.0600164	Birmingham	UK	lineage4	S	S	S	S	S	S	S	S
13.0613523	Birmingham	UK	lineage4	S	S	S	S	S	S	S	S
16.0601534	Birmingham	UK	Delhi	S	S	S	S	S	S	S	S
09.0600324	Birmingham	UK	Delhi	S	S	S	S	S	S	S	S

15.0601957	Birmingham	UK	lineage4	S	S	S	S	S	S	S	S
13.0612474	Birmingham	UK	Beijing	S	S	S	S	S	S	S	S
14.0600355	Birmingham	UK	EAI	S	S	S	S	S	S	U	S
13.0613442	Birmingham	UK	Delhi	S	S	S	S	S	S	U	S
13.0609882	Birmingham	UK	West Africar	S	S	S	S	S	U	U	S
15.0613045	Birmingham	UK	Beijing	S	S	S	S	F	R	S	S
15.0607541	Birmingham	UK	Haarlem	R	S	S	S	R	S	R	S
13.0600564	Birmingham	UK	EAI	S	S	S	S	S	S	S	S
15.0601465	Birmingham	UK	EAI	S	S	S	S	S	S	S	S
12.0609172	Birmingham	UK	Delhi	S	S	S	S	S	S	S	S
12.0614871	Birmingham	UK	EAI	S	S	S	S	S	S	S	S
16.0601984	Birmingham	UK	Delhi	S	S	S	S	S	S	S	S
15.0616126	Birmingham	UK		S	S	S	S	S	S	S	S
14.0602969	Birmingham	UK	Delhi	S	S	S	S	S	S	S	S
15.0606556	Birmingham	UK	LAM	S	S	S	S	S	S	S	S
15.0606890	Birmingham	UK	Delhi	R	S	S	S	R	S	S	S
15.0602296	Birmingham	UK	Delhi	S	S	S	S	S	S	U	S
16.0607975	Birmingham	UK	Cameroon	S	S	S	S	S	F	S	S
13.0613434	Birmingham	UK	EAI	S	S	S	S	S	S	S	S
15.0613142	Birmingham	UK	EAI	S	S	S	S	S	S	F	S
16.0605734	Birmingham	UK	Beijing	S	S	S	S	S	S	S	S
15.0601404	Birmingham	UK	EAI	R	S	S	S	S	S	U	F
12.0615423	Birmingham	UK	Delhi	S	S	S	S	S	S	S	S
13.0612477	Birmingham	UK	Beijing	S	S	S	S	S	S	S	S
13.0609250	Birmingham	UK	Delhi	S	S	S	S	S	S	S	S
12.0607013	Birmingham	UK	Haarlem	S	S	S	S	S	S	S	S
14.0605428	Birmingham	UK	X-type	S	S	S	S	S	S	S	S
13.0609881	Birmingham	UK	Delhi	S	S	S	S	S	S	S	S
13.0604781	Birmingham	UK	Delhi	R	S	S	S	R	S	S	S
12.0610292	Birmingham	UK	lineage4	S	S	S	S	S	S	S	S
14.0600685	Birmingham	UK	lineage4	S	S	S	S	S	S	S	S
16.0601075	Birmingham	UK		S	S	S	S	S	S	S	S
16.0601920	Birmingham	UK	Delhi	S	S	S	S	S	S	S	S
16.0601301	Birmingham	UK	LAM	S	S	S	S	S	S	S	S
15.0605089	Birmingham	UK	EAI	S	S	S	S	S	S	S	S
13.0613535	Birmingham	UK	Beijing	S	S	S	S	S	S	S	S
14.0603656	Birmingham	UK	Delhi	S	S	S	S	S	S	S	S
16.0601840	Birmingham	UK	lineage4	S	S	S	S	S	S	S	S
15.0601269	Birmingham	UK	Delhi	S	S	S	S	S	S	S	S
13.0611282	Birmingham	UK	Beijing	S	S	S	S	S	R	S	S
15.0602254	Birmingham	UK	lineage4	S	S	S	S	S	S	S	S
14.0609759	Birmingham	UK	Delhi	S	S	S	S	S	U	S	S
13.0615163	Birmingham	UK	Delhi	R	S	S	S	R	S	S	S
12.0611225	Birmingham	UK	Delhi	S	S	S	S	S	S	S	S
15.0601176	Birmingham	UK	X-type	R	S	S	S	R	R	R	F
14.0609113	Birmingham	UK	X-type	S	S	S	R	S	S	S	R
16.0607333	Birmingham	UK	Delhi	S	S	S	S	S	F	S	S
13.0613531	Birmingham	UK	Delhi	S	S	S	S	S	R	U	S
14.0600875	Birmingham	UK	Haarlem	S	S	S	S	S	S	S	S
16.0603216	Birmingham	UK	EAI	S	S	S	n/a	S	S	S	S
16.0618292	Birmingham	UK	Haarlem	S	S	S	S	S	S	S	S
16.0609637	Birmingham	UK	Delhi	S	S	S	S	U	S	S	S
14.0609208	Birmingham	UK	M. bovis	S	S	S	R	S	S	U	R
14.0609069	Birmingham	UK	Haarlem	S	S	S	S	S	S	S	S
13.0607958	Birmingham	UK	M. orygis	S	S	S	S	S	S	S	S
13.0603158	Birmingham	UK	Delhi	S	S	S	S	S	S	S	S
14.0609250	Birmingham	UK	Ural	S	S	S	S	S	S	S	S
13.0606713	Birmingham	UK	Delhi	S	S	S	S	S	S	S	S
15.0603908	Birmingham	UK	LAM	S	S	S	S	S	S	S	S
16.0612329	Birmingham	UK	S-type	S	S	S	n/a	S	S	S	R
12.0606859	Birmingham	UK	EAI	R	S	S	S	S	S	S	S
12.0616372	Birmingham	UK	EAI	S	S	S	S	S	S	U	S
13.0603103	Birmingham	UK	Delhi	S	S	S	S	S	S	S	S
13.0609677	Birmingham	UK	Delhi	S	S	S	S	S	S	S	S
13.0613396	Birmingham	UK	Delhi	S	S	S	S	S	S	S	S
15.0607686	Birmingham	UK	LAM	S	S	S	S	S	S	S	S
11.0602205	Birmingham	UK	Delhi	S	S	S	S	S	S	S	S
13.0613538	Birmingham	UK	LAM	S	S	S	S	S	S	S	S
12.0612302	Birmingham	UK	Delhi	S	S	S	S	S	S	S	S
14.0600404	Birmingham	UK	lineage4	S	S	S	S	S	S	S	S
16.0618001	Birmingham	UK	Cameroon	S	S	S	S	S	S	S	S
16.0601244	Birmingham	UK	Ural	R	S	S	S	R	S	S	S
15.0607643	Birmingham	UK	Delhi	S	S	S	S	S	S	S	S
12.0609018	Birmingham	UK	Delhi	S	S	S	S	S	S	S	S
12.0615702	Birmingham	UK	X-type	S	S	S	S	S	S	S	S
16.0614065	Birmingham	UK	Delhi	S	S	S	S	S	S	S	S
13.0605724	Birmingham	UK	Delhi	S	S	S	S	S	S	S	S
14.0612382	Birmingham	UK	M. bovis	n/a	n/a	n/a	R	S	S	S	R
13.0604884	Birmingham	UK	lineage4	S	S	S	S	S	S	S	S
17.0601172	Birmingham	UK	Beijing	n/a	S	S	S	R	S	S	S
14.0602761	Birmingham	UK	X-type	R	S	S	R	R	R	R	F
13.0607710	Birmingham	UK	Delhi	R	S	S	S	R	R	R	S
13.0606693	Birmingham	UK	Delhi	S	S	S	S	S	S	S	S
13.0609242	Birmingham	UK	Tur	S	S	S	S	S	U	U	S
13.0613432	Birmingham	UK	Delhi	S	S	S	S	S	S	S	S
15.0613365	Birmingham	UK	Delhi	S	S	S	S	S	F	S	S
13.0606700	Birmingham	UK	lineage4	S	S	S	S	S	S	S	S
13.0613384	Birmingham	UK	Beijing	S	S	S	S	S	S	S	S
09.0600986	Birmingham	UK	Haarlem	S	S	S	n/a	S	S	S	S
16.0601254	Birmingham	UK	lineage4	S	S	S	S	S	S	S	S
13.0606638	Birmingham	UK	Delhi	S	S	S	S	S	S	S	S
15.0613339	Birmingham	UK	EAI	S	S	S	n/a	U	S	S	S
14.0609264	Birmingham	UK	EAI	S	S	S	S	S	S	S	S
10.0609538	Birmingham	UK	Tur	S	S	S	S	S	S	S	S
11.0611029	Birmingham	UK	Delhi	S	S	S	S	S	S	S	S
14.0600906	Birmingham	UK	EAI	S	S	S	S	S	S	S	S
13.0607691	Birmingham	UK	Delhi	R	S	R	S	R	S	R	S
16.0618557	Birmingham	UK	lineage4	S	S	S	S	S	S	S	S
13.0610756	Birmingham	UK	EAI	S	S	S	n/a	S	S	S	S
13.0607979	Birmingham	UK	LAM	S	S	S	S	S	S	S	S
16.0607374	Birmingham	UK	Haarlem	S	S	S	S	S	S	S	S
12.0615227	Birmingham	UK	lineage4	S	S	S	S	S	S	U	S
12.0611233	Birmingham	UK	Delhi	S	S	S	S	S	S	U	S
16.0611540	Birmingham	UK	lineage4	S	S	S	S	S	S	S	S
13.0613345	Birmingham	UK	X-type	S	S	S	S	S	S	S	S
13.0604188	Birmingham	UK	lineage4	S	S	S	S	S	S	S	S
13.0605721	Birmingham	UK	Delhi	S	S	S	S	S	S	U	S
13.0611015	Birmingham	UK	lineage4	S	S	S	S	S	S	S	S
15.0613109	Birmingham	UK	LAM	S	S	S	S	S	S	S	S
14.0600548	Birmingham	UK	Delhi	S	S	S	S	S	S	S	S
16.0607866	Birmingham	UK	Delhi	S	S	S	S	S	F	S	S
15.0601930	Birmingham	UK	Delhi	R	S	S	S	R	U	S	S
13.0602081	Birmingham	UK	Delhi	S	S	S	S	S	S	S	S
14.0600826	Birmingham	UK	LAM	S	S	S	S	S	S	S	S
15.0607780	Birmingham	UK	Delhi	n/a	n/a	S	n/a	S	S	S	S
15.0613480	Birmingham	UK	Delhi	S	S	S	S	S	S	U	S

13.0613399	Birmingham	UK	lineage4	S	S	S	S	S	S	S	S
14.0614289	Birmingham	UK	Haarlem	S	S	S	S	S	S	U	S
12.0612492	Birmingham	UK	Delhi	S	S	S	S	U	S	S	S
15.0613012	Birmingham	UK	lineage4	S	S	S	S	S	S	S	S
14.0603011	Birmingham	UK	Delhi	S	S	S	S	S	S	S	S
15.0607570	Birmingham	UK	Beijing	S	S	S	S	S	S	S	S
16.0608201	Birmingham	UK	Beijing	S	S	S	S	S	S	S	S
13.0613522	Birmingham	UK	X-type	n/a	n/a	n/a	S	S	S	S	S
15.0607445	Birmingham	UK	lineage4	S	S	S	S	S	S	S	S
13.0604550	Birmingham	UK	LAM	S	S	S	S	S	S	S	S
12.0612518	Birmingham	UK		S	S	S	S	S	S	S	S
15.0601337	Birmingham	UK	M. bovis	S	S	S	R	S	S	S	R
16.0618390	Birmingham	UK	Beijing	S	S	S	S	S	S	S	S
13.0609468	Birmingham	UK	Delhi	S	S	S	S	S	S	S	S
13.0608235	Birmingham	UK	lineage4	S	S	S	S	S	S	S	S
13.0600576	Birmingham	UK	Delhi	S	S	S	S	S	S	S	S
14.0616029	Birmingham	UK	EAI	S	S	S	S	S	S	S	S
16.0617298	Birmingham	UK	Delhi	S	S	S	S	S	S	S	S
15.0613457	Birmingham	UK	lineage4	S	S	S	S	U	S	S	S
12.0615229	Birmingham	UK		S	S	S	S	S	S	S	S
14.0601551	Birmingham	UK	Delhi	R	S	S	S	R	S	R	S
16.0601257	Birmingham	UK	Tur	S	S	S	S	S	S	S	S
14.0600871	Birmingham	UK	Uganda	S	S	S	S	S	S	U	S
16.0607745	Birmingham	UK	LAM	S	S	S	S	S	F	S	S
15.0613528	Birmingham	UK	M. orygis	S	S	S	S	S	S	S	S
16.0607560	Birmingham	UK	Haarlem	S	S	S	S	S	S	S	S
12.0615244	Birmingham	UK	Delhi	S	S	S	S	S	S	S	S
13.0613227	Birmingham	UK	LAM	S	S	S	S	S	S	S	S
16.0601449	Birmingham	UK	EAI	S	S	S	S	S	S	S	S
12.0611801	Birmingham	UK	lineage4	S	S	S	S	S	S	U	S
13.0613121	Birmingham	UK		S	S	S	S	S	S	S	S
16.0601137	Birmingham	UK	Haarlem	S	S	S	S	S	S	S	S
14.0600830	Birmingham	UK	LAM	S	S	S	S	S	S	S	S
14.0609770	Birmingham	UK	X-type	S	S	S	S	S	S	S	S
16.0601987	Birmingham	UK	Delhi	S	S	S	S	F	F	S	F
15.0601347	Birmingham	UK	Delhi	S	S	S	S	S	S	S	S
14.0602055	Birmingham	UK	Delhi	S	S	S	S	S	S	S	S
14.0616073	Birmingham	UK	Haarlem	S	S	S	S	S	S	S	S
15.0601024	Birmingham	UK	EAI	S	S	n/a	n/a	S	S	S	S
15.0613067	Birmingham	UK	Beijing	S	S	S	S	S	S	S	S
15.0607098	Birmingham	UK	S-type	S	S	S	S	S	S	S	S
16.0605422	Birmingham	UK		S	S	S	R	S	S	S	R
12.0614971	Birmingham	UK	Delhi	S	S	S	S	S	S	S	S
14.0600050	Birmingham	UK	Delhi	S	S	S	S	S	S	S	S
11.0606009	Birmingham	UK	LAM	S	S	S	S	S	S	S	S
16.0617169	Birmingham	UK	Haarlem	S	S	S	S	S	S	S	S
15.0607731	Birmingham	UK	Haarlem	S	R	S	S	S	R	S	S
16.0607908	Birmingham	UK	lineage4	S	S	S	S	S	S	S	S
15.0607735	Birmingham	UK	Beijing	R	R	R	R	R	R	R	R
13.0613324	Birmingham	UK	Delhi	S	S	S	S	S	S	S	S
14.0600181	Birmingham	UK	lineage4	S	S	S	S	S	S	S	S
12.0611182	Birmingham	UK	lineage4	S	S	S	S	S	S	S	S
09.0601720	Birmingham	UK	Delhi	S	S	S	S	S	S	S	S
15.0607474	Birmingham	UK	Delhi	S	S	S	S	S	S	S	S
14.0609552	Birmingham	UK	lineage4	S	S	S	S	S	S	S	S
15.0601210	Birmingham	UK	EAI	S	S	S	S	S	S	S	S
14.0609366	Birmingham	UK	Delhi	S	S	S	S	S	S	S	S
15.0601945	Birmingham	UK	Beijing	S	S	S	S	S	S	S	S
12.0607577	Birmingham	UK	EAI	S	S	S	S	S	S	S	S
15.0606898	Birmingham	UK	Cameroon	S	S	S	S	S	S	S	S
15.0607136	Birmingham	UK	Delhi	S	S	S	S	S	S	S	S
13.0608423	Birmingham	UK	X-type	S	S	S	S	S	S	S	S
14.0610316	Birmingham	UK	M. orygis	S	S	S	S	S	S	S	S
10.0605692	Birmingham	UK	lineage4	S	S	S	S	S	S	S	S
15.0601756	Birmingham	UK	Haarlem	S	S	S	S	S	S	U	S
12.0613642	Birmingham	UK	Delhi	S	S	S	S	F	S	S	S
12.0612708	Birmingham	UK	Beijing	S	S	S	S	S	S	S	S
14.0603751	Birmingham	UK	lineage4	S	S	S	S	S	S	S	S
16.0601645	Birmingham	UK	Delhi	S	S	S	S	S	F	S	F
15.0607090	Birmingham	UK	X-type	S	S	S	S	S	S	U	S
12.0616904	Birmingham	UK	Delhi	S	S	S	S	S	S	S	S
12.0602124	Birmingham	UK	Uganda	S	S	S	S	S	S	S	S
15.0607274	Birmingham	UK	EAI	S	S	S	S	S	S	S	S
13.0611339	Birmingham	UK	Delhi	S	S	S	S	S	S	S	S
15.0602291	Birmingham	UK	Delhi	S	S	S	S	S	S	U	S
15.0613405	Birmingham	UK	Delhi	S	S	S	S	S	S	S	S
12.0614984	Birmingham	UK	X-type	S	S	S	S	S	S	S	S
16.0617290	Birmingham	UK	Beijing	S	S	S	S	S	S	S	S
16.0600444	Birmingham	UK	Haarlem	S	S	S	S	S	S	S	S
14.0609740	Birmingham	UK	lineage4	S	S	S	S	S	S	U	S
14.0616065	Birmingham	UK	Beijing	n/a	n/a	n/a	S	S	S	U	S
14.0609732	Birmingham	UK	LAM	S	S	S	S	S	S	S	S
11.0606156	Birmingham	UK	EAI	S	S	S	S	S	S	S	S
15.0607061	Birmingham	UK	lineage4	R	S	S	S	R	S	S	S
13.0607711	Birmingham	UK	Delhi	S	S	S	S	S	S	S	S
13.0607999	Birmingham	UK	Haarlem	S	S	S	S	S	S	S	S
16.0607605	Birmingham	UK	S-type	S	S	S	S	S	S	S	S
15.0607952	Birmingham	UK	Delhi	S	S	S	S	F	F	F	F
16.0601165	Birmingham	UK	Haarlem	S	S	S	S	S	S	S	S
13.0605001	Birmingham	UK	M. orygis	S	S	S	S	U	S	S	S
17.0601218	Birmingham	UK	Delhi	S	S	S	S	S	S	S	S
16.0601259	Birmingham	UK	lineage4	S	S	S	S	S	S	S	S
13.0610173	Birmingham	UK	Ural	R	S	S	S	R	S	S	S
13.0613117	Birmingham	UK	X-type	S	S	S	S	S	S	S	U
14.0600679	Birmingham	UK	Delhi	S	S	S	S	S	S	S	S
14.0600944	Birmingham	UK	Beijing	S	S	S	S	S	S	U	S
09.0601719	Birmingham	UK	Delhi	S	S	S	S	S	S	S	S
16.0617328	Birmingham	UK	Delhi	S	S	S	S	S	S	S	S
15.0601613	Birmingham	UK	X-type	S	S	S	S	S	S	S	S
16.0607960	Birmingham	UK	Delhi	S	S	S	S	S	S	S	S
12.0614539	Birmingham	UK	Delhi	S	S	S	S	S	S	S	S
15.0613015	Birmingham	UK	Beijing	S	R	S	S	S	R	S	S
13.0606645	Birmingham	UK	lineage4	S	S	S	S	S	S	U	S
16.0603866	Birmingham	UK	Delhi	S	S	S	S	S	S	S	S
16.0607410	Birmingham	UK	Beijing	S	S	S	S	S	S	S	S
15.0607829	Birmingham	UK	EAI	S	S	S	S	S	S	S	S
14.0613000	Birmingham	UK	Delhi	R	S	S	S	R	S	S	S
15.0613020	Birmingham	UK	Delhi	R	n/a	S	S	R	S	S	S
14.0600768	Birmingham	UK	Haarlem	S	S	S	S	S	S	U	S
16.0601040	Birmingham	UK	Haarlem	S	S	S	S	S	S	S	S
15.0613519	Birmingham	UK	Ural	R	S	S	S	F	S	U	F
14.0600071	Birmingham	UK	Delhi	S	S	S	S	S	S	S	S
16.0601230	Birmingham	UK	lineage4	S	S	S	S	S	S	S	S
12.0611798	Birmingham	UK	EAI	S	S	S	S	S	S	U	S
16.0614260	Birmingham	UK	EAI	n/a	n/a	n/a	S	S	S	S	S
14.0609524	Birmingham	UK	EAI	S	S	S	S	S	S	S	S

16.0618082	Birmingham	UK	Delhi	S	S	S	S	S	S	S	S
13.0610489	Birmingham	UK	West Africar	S	S	S	S	S	S	U	S
14.0600801	Birmingham	UK	Beijing	S	S	S	n/a	S	S	S	S
12.0601948	Birmingham	UK	S-type	S	S	S	S	S	S	S	S
16.0607004	Birmingham	UK	lineage4	S	S	S	S	S	S	S	S
12.0611810	Birmingham	UK	lineage4	S	S	S	S	S	S	S	S
13.0608467	Birmingham	UK	Delhi	R	S	S	S	R	S	S	S
14.0600796	Birmingham	UK	Haarlem	S	S	S	S	S	S	S	S
13.0609467	Birmingham	UK	Delhi	S	S	S	S	U	S	S	S
14.0609526	Birmingham	UK	Delhi	S	S	S	S	S	S	S	S
14.0600789	Birmingham	UK	Delhi	R	S	S	S	R	S	S	S
13.0607148	Birmingham	UK	Delhi	R	S	S	S	R	S	S	S
11.0610105	Birmingham	UK	Delhi	S	S	S	S	S	S	S	S
13.0602613	Birmingham	UK	Cameroon	S	S	S	S	S	S	S	S
16.0605229	Birmingham	UK	Delhi	S	S	S	S	S	S	S	S
7627736	Leeds	UK	Beijing	S	S	S	S	S	S	U	S
7620040	Leeds	UK	Delhi	S	S	S	S	S	S	U	S
7620018	Leeds	UK	LAM	S	S	S	S	S	S	U	S
7627124	Leeds	UK	Delhi	S	S	S	S	S	S	U	S
7627400	Leeds	UK	lineage4	S	S	S	S	S	S	U	S
7626694	Leeds	UK	Delhi	S	S	S	S	S	S	F	S
7626571	Leeds	UK	EAI	S	S	S	S	F	F	S	F
7627900	Leeds	UK	Delhi	S	S	S	S	S	S	U	S
7628147	Leeds	UK	EAI	S	S	S	S	S	F	F	S
7626930	Leeds	UK	Beijing	R	R	R	S	R	R	R	R
7626833	Leeds	UK	Delhi	S	S	S	S	S	S	S	F
7628121	Leeds	UK	Delhi	S	S	S	S	S	S	U	S
7620062	Leeds	UK	X-type	S	S	S	S	S	S	U	S
7628143	Leeds	UK	Haarlem	S	S	S	S	S	S	S	S
7626675	Leeds	UK	Beijing	R	R	R	S	R	R	R	R
7626725	Leeds	UK	Beijing	S	S	S	S	S	S	S	S
7627574	Leeds	UK	Delhi	S	S	S	S	S	S	U	S
7626555	Leeds	UK	Delhi	S	S	S	S	S	S	U	S
7620149	Leeds	UK	Delhi	S	S	S	S	S	S	S	S
7626433	Leeds	UK	lineage4	S	S	S	S	S	F	F	S
7627886	Leeds	UK	Cameroon	S	S	S	S	U	S	U	S
7620009	Leeds	UK	Delhi	S	S	S	S	S	S	U	S
7627572	Leeds	UK	Delhi	S	S	S	S	S	S	S	S
7627226	Leeds	UK	Beijing	R	R	R	S	R	R	R	R
7626487	Leeds	UK	Delhi	S	S	S	S	S	S	S	S
7627636	Leeds	UK	Haarlem	S	S	S	S	S	S	U	F
7627434	Leeds	UK	Delhi	S	S	S	S	U	S	U	S
7626752	Leeds	UK	Delhi	S	S	S	S	S	S	F	S
7626878	Leeds	UK	lineage4	S	S	S	S	S	S	S	S
7627116	Leeds	UK	lineage4	S	S	S	S	S	S	U	S
7626713	Leeds	UK	lineage4	S	S	S	S	S	S	S	S
H124740138	London	UK	Haarlem	R	R	S	S	R	R	R	S
H141600041	London	UK	Beijing	S	S	S	S	S	S	S	S
H112780034	London	UK	Ural	R	R	R	S	R	R	U	S
H101920168	London	UK	Delhi	R	R	n/a	n/a	R	R	R	S
H121260031	London	UK	Ural	R	n/a	S	S	R	R	S	S
H132740033	London	UK	Delhi	R	R	S	S	R	R	F	S
H125000072	London	UK	Delhi	R	R	S	S	R	R	U	S
H133920162	London	UK	Delhi	R	R	n/a	S	R	R	R	S
H142040333	London	UK	Beijing	R	R	R	S	R	R	R	R
H123360011	London	UK	Beijing	R	R	n/a	S	R	R	R	U
H120580018	London	UK	Delhi	R	R	S	S	R	R	R	R
H101620117	London	UK	EAI	R	R	n/a	S	R	R	R	R
H102500147	London	UK	LAM	R	R	n/a	R	R	R	R	R
H135000214	London	UK	EAI	R	R	S	S	R	R	S	U
H103180114	London	UK	Delhi	R	R	S	S	R	R	S	S
H124040080	London	UK	Delhi	R	S	S	S	R	F	S	S
H114980032	London	UK	Haarlem	R	R	n/a	n/a	R	R	R	R
H134640235	London	UK	Tur	R	R	R	R	R	R	R	U
H102780066	London	UK	Cameroon	R	R	S	S	R	R	S	S
H131820987	London	UK	Beijing	R	R	n/a	R	R	R	R	R
H141800023	London	UK	Cameroon	R	R	S	n/a	R	R	S	S
H144680079	London	UK	S-type	R	R	n/a	S	R	R	R	S
H121320026	London	UK	Beijing	S	n/a	S	S	S	S	S	S
H101880206	London	UK	EAI	R	R	n/a	S	R	R	R	R
H112140033	London	UK	Delhi	R	R	n/a	S	R	R	R	S
H131680029	London	UK	Beijing	R	R	n/a	n/a	R	R	R	R
H133140029	London	UK	lineage4	R	R	n/a	S	R	R	R	S
H114280002	London	UK	Ural	R	R	R	S	R	R	U	S
H122480042	London	UK	X-type	R	R	n/a	n/a	R	R	S	R
H110340018	London	UK	Beijing	R	R	n/a	R	R	R	U	R
H133180021	London	UK	Beijing	R	R	n/a	S	R	R	R	S
H115160021	London	UK	EAI	R	R	n/a	n/a	S	R	S	S
H114920015	London	UK	Beijing	R	R	n/a	R	R	R	R	R
H102740017	London	UK	Beijing	R	R	n/a	n/a	R	R	S	S
H124080087	London	UK	Haarlem	R	R	R	R	R	R	R	U
H102540112	London	UK	Beijing	R	R	n/a	R	R	R	U	R
H140820208	London	UK	Delhi	R	S	S	S	F	S	S	F
H105080006	London	UK	Beijing	R	R	n/a	R	R	R	R	R
H112980114	London	UK	LAM	R	R	n/a	S	R	R	U	S
H140640110	London	UK	Beijing	R	R	R	S	R	R	R	U
H105240002	London	UK	Beijing	R	R	n/a	S	R	R	R	R
H114600114	London	UK	Beijing	R	R	R	S	R	R	R	R
H111860011	London	UK	Delhi	R	R	S	S	R	R	S	R
H121040031	London	UK	LAM	R	R	S	R	R	R	R	S
H114440480	London	UK	Beijing	R	R	R	R	R	R	R	R
H140800280	London	UK	Beijing	n/a	R	S	S	R	F	S	S
H122800024	London	UK	EAI	R	R	S	S	R	R	S	R
H140820241	London	UK	Beijing	R	R	R	R	R	R	R	R
H132140021	London	UK	Beijing	R	R	R	R	R	R	R	R
H143080030	London	UK	EAI	R	R	S	S	R	U	S	S
H102080138	London	UK	Delhi	R	R	n/a	n/a	R	R	R	S
H123300022	London	UK	Delhi	R	R	n/a	S	R	R	R	S
H130140043	London	UK	LAM	R	R	R	S	R	R	R	S
H121160050	London	UK	Beijing	R	R	R	S	R	R	U	S
H110580062	London	UK	Delhi	R	n/a	S	S	R	R	S	S
H130660134	London	UK	Beijing	R	R	R	S	R	R	R	R
H111720040	London	UK	Beijing	S	S	S	S	S	S	S	S
H131080024	London	UK	LAM	R	n/a	n/a	R	R	R	R	R
H140640101	London	UK	Beijing	R	R	R	R	R	R	R	U
H142440027	London	UK	Beijing	R	R	R	S	R	R	R	R
H131520071	London	UK	Delhi	R	R	n/a	n/a	R	R	R	S
H114780024	London	UK	Beijing	R	R	R	R	R	R	F	F
H111380011	London	UK	EAI	R	R	n/a	R	R	R	U	R
H121380012	London	UK	EAI	R	R	n/a	n/a	R	R	S	S
H103060020	London	UK	Beijing	R	R	S	S	R	R	U	S
H110560102	London	UK	Beijing	R	R	R	R	R	R	R	R
H120460024	London	UK	Beijing	R	R	n/a	n/a	R	R	R	R
H121640036	London	UK	lineage4	R	R	n/a	S	R	R	R	S
H102060482	London	UK	LAM	R	R	S	S	R	R	R	U
H121340004	London	UK	Delhi	R	R	R	S	R	R	R	S

H103920462	London	UK	lineage4	R	R	S	R	R	R	R	R
H112600014	London	UK	Ural	R	R	R	S	R	R	R	R
H143340175	London	UK	Beijing	R	R	R	R	R	R	R	R
H130280011	London	UK	Beijing	R	R	n/a	S	R	R	R	U
H111840007	London	UK	Delhi	R	R	S	S	R	R	S	S
H113540015	London	UK	Delhi	R	R	n/a	n/a	R	R	R	S
H143740205	London	UK	X-type	n/a	n/a	n/a	S	R	R	R	R
H111940054	London	UK	Beijing	R	R	S	R	R	R	S	R
H131000008	London	UK	Delhi	R	R	S	S	R	R	S	S
H121320033	London	UK	EAI	R	R	R	S	R	R	R	S
H131560009	London	UK	Delhi	R	R	S	S	R	R	R	R
H120420019	London	UK	Delhi	R	R	R	S	R	R	R	R
H144480021	London	UK	Beijing	R	R	R	R	R	R	R	R
H111560029	London	UK	Beijing	R	R	S	S	R	R	U	R
H112000008	London	UK	Beijing	R	R	R	R	R	R	R	R
H120340011	London	UK	Beijing	R	R	R	R	R	R	R	R
H123200010	London	UK	Beijing	R	R	n/a	S	R	R	R	R
H142600033	London	UK	Delhi	R	R	S	R	R	R	F	S
H110540031	London	UK	EAI	n/a	R	R	S	R	R	R	R
H110800117	London	UK	Beijing	R	R	R	R	R	R	R	R
H121200024	London	UK	Delhi	S	S	S	S	F	F	U	S
H134920401	London	UK	X-type	R	R	n/a	R	F	R	R	F
H102860140	London	UK	Delhi	R	R	S	S	R	R	S	S
H102880194	London	UK	EAI	R	R	n/a	S	R	R	R	R
H111560023	London	UK	Cameroon	R	R	S	S	R	R	S	S
H141780187	London	UK	Beijing	R	R	n/a	S	R	R	U	S
H123320024	London	UK	Cameroon	R	R	n/a	n/a	R	R	S	S
H134780024	London	UK	Delhi	R	n/a	S	S	R	R	R	S
H143160013	London	UK	Beijing	R	R	n/a	S	R	R	R	R
H112040018	London	UK	Delhi	R	R	n/a	S	R	R	R	U
H120720148	London	UK	LAM	R	R	n/a	R	R	R	R	R
H113100007	London	UK	Beijing	R	R	R	R	R	R	R	R
H130820016	London	UK	Beijing	R	R	n/a	R	R	R	R	R
H110820014	London	UK	Beijing	R	R	R	S	R	R	R	R
H103340621	London	UK	Beijing	R	R	R	S	R	R	R	R
H102960052	London	UK	Beijing	R	R	S	R	R	R	R	R
H114540002	London	UK	Beijing	R	R	R	R	R	R	R	R
H111380213	London	UK	Beijing	R	R	n/a	S	R	R	R	S
H102640088	London	UK	Delhi	R	R	n/a	S	R	R	R	S
H104480455	London	UK	lineage4	R	R	n/a	S	S	R	R	S
H111540011	London	UK	Ghana	R	R	n/a	n/a	R	R	R	S
H142420003	London	UK	Ural	R	R	n/a	R	R	R	U	R
H112440007	London	UK	Beijing	R	R	n/a	n/a	R	R	R	R
H131220032	London	UK	Beijing	R	R	n/a	n/a	R	R	R	R
H143480223	London	UK	Beijing	R	R	R	R	R	R	R	R
H143620222	London	UK	Delhi	R	R	S	S	U	S	S	S
H134240034	London	UK	Beijing	R	R	R	S	R	R	R	S
H140300086	London	UK	LAM	R	R	n/a	S	R	R	R	S
H122540026	London	UK	Beijing	R	R	n/a	n/a	R	R	R	S
H121320021	London	UK	Haarlem	R	R	S	S	R	R	S	S
H143180015	London	UK	Delhi	R	S	S	S	R	S	U	F
H102580404	London	UK	Beijing	R	R	n/a	S	R	R	R	S
H105160024	London	UK	Beijing	R	R	n/a	S	R	R	R	S
H120840306	London	UK	Beijing	R	R	R	S	R	R	R	S
H134900365	London	UK	EAI	R	n/a	S	S	R	R	S	S
H132060332	London	UK	Beijing	R	R	n/a	S	R	R	R	R
H123680040	London	UK	Beijing	R	R	n/a	S	R	R	R	R
H110560002	London	UK	Beijing	R	R	n/a	R	R	R	R	R
H122040011	London	UK	Tur	R	R	R	R	R	R	R	R
H133300021	London	UK	Delhi	S	S	S	S	F	S	S	F
H130640097	London	UK	EAI	R	R	R	R	R	R	R	R
H112160033	London	UK	Delhi	R	R	n/a	S	R	R	R	U
H131680038	London	UK	Beijing	R	R	R	R	R	R	R	R
H130900010	London	UK	Delhi	S	S	S	S	S	S	S	F
H123720020	London	UK	Delhi	R	R	n/a	S	R	R	R	S
H122740073	London	UK	Beijing	R	R	R	R	R	R	R	R
H145040031	London	UK	Beijing	R	R	n/a	S	R	R	R	S
H123340049	London	UK	X-type	R	R	n/a	n/a	R	R	S	R
H123220062	London	UK	Delhi	R	R	S	S	R	R	R	S
H134280018	London	UK	Delhi	R	R	R	S	R	R	R	S
H112240073	London	UK	Beijing	R	R	n/a	R	R	R	R	R
H102240043	London	UK	EAI	R	R	n/a	S	R	R	R	R
H132480029	London	UK	Beijing	R	R	n/a	R	R	R	R	R
H102800105	London	UK	Delhi	R	R	n/a	n/a	R	R	R	R
H150280020	London	UK	Tur	R	R	R	S	R	R	R	S
H123300023	London	UK	LAM	R	R	n/a	S	R	R	R	S
H112560226	London	UK	Beijing	R	R	R	S	R	R	R	R
H142100097	London	UK	Beijing	n/a	R	S	S	R	R	S	S
H131680035	London	UK	Beijing	R	R	R	S	R	R	R	R
H120580035	London	UK	Cameroon	S	n/a	S	S	S	S	S	S
H143100018	London	UK	Delhi	R	R	n/a	R	R	R	R	R
H104120008	London	UK	Beijing	R	R	R	R	R	R	R	R
H143780227	London	UK	EAI	R	R	n/a	S	R	U	S	S
H133240016	London	UK	Beijing	R	R	n/a	R	R	R	R	R
H113140103	London	UK	Delhi	R	R	n/a	S	R	R	R	S
H130760079	London	UK	Beijing	R	R	n/a	R	R	R	R	U
H144880098	London	UK	EAI	R	R	S	S	R	R	S	S
H122040022	London	UK	Beijing	R	R	n/a	n/a	R	R	R	S
H113180014	London	UK	Beijing	R	R	R	R	R	R	R	R
H111620021	London	UK	Beijing	R	R	R	R	R	R	R	R
H144320050	London	UK	Beijing	R	R	R	S	R	R	R	S
H102500146	London	UK	LAM	R	R	n/a	R	R	R	R	R
H123120211	London	UK	LAM	R	R	n/a	S	R	R	R	S
H144300224	London	UK	Delhi	R	R	S	S	R	R	S	S
H125180101	London	UK	Tur	R	R	S	S	R	R	U	S
H112380031	London	UK	Beijing	R	R	n/a	S	R	R	R	R
H134980271	London	UK	Beijing	R	R	n/a	S	R	R	R	F
H133160689	London	UK	LAM	R	R	n/a	S	R	R	R	S
H120660003	London	UK	Delhi	R	R	n/a	R	R	R	R	R
H112040005	London	UK	Haarlem	R	R	S	S	R	R	S	S
H113140110	London	UK	Beijing	R	R	R	R	R	R	R	R
H112580018	London	UK	Beijing	R	R	R	R	R	R	R	R
H103460397	London	UK	EAI	R	R	S	S	R	R	S	S
H111520011	London	UK	lineage4	R	R	S	S	R	R	R	S
H130380158	London	UK	Tur	R	R	S	R	R	R	R	R
H111960022	London	UK	Delhi	R	R	n/a	n/a	R	R	S	S
H143240163	London	UK	Beijing	R	R	R	S	R	R	R	R
H102720013	London	UK	Beijing	R	R	n/a	R	R	R	R	U
H143140060	London	UK	Delhi	R	R	S	S	U	R	S	S
H140880224	London	UK	Delhi	R	S	S	S	U	S	S	S
H114620029	London	UK	Haarlem	R	R	R	n/a	R	R	R	R
H124820130	London	UK	Beijing	R	R	n/a	n/a	R	R	R	R
H114980033	London	UK	Beijing	R	R	n/a	n/a	R	R	R	R
H143480205	London	UK	Delhi	R	R	S	S	R	R	S	S
H145020032	London	UK	Tur	R	R	R	S	R	R	R	S
H120160025	London	UK	Delhi	R	R	S	S	R	R	R	S

H120640698	London	UK	Beijing	R	R	R	R	R	R	R	R
H133300002	London	UK	Beijing	R	R	n/a	R	R	R	R	R
H112580012	London	UK	Delhi	R	R	S	S	R	R	S	R
H102260124	London	UK	EAI	R	R	R	R	R	R	R	R
H111900036	London	UK	Cameroon	S	S	S	S	S	S	S	S
H131020017	London	UK	Delhi	R	R	n/a	S	R	R	R	S
H120840023	London	UK	lineage4	R	R	n/a	S	R	R	R	S
H114840036	London	UK	Delhi	R	R	S	S	R	R	R	R
H114360011	London	UK	LAM	R	R	S	R	R	R	R	S
H101920694	London	UK	Beijing	R	R	R	R	R	R	R	U
H140260305	London	UK	Beijing	R	n/a	n/a	S	R	R	R	U
H114700009	London	UK	Delhi	R	R	S	S	R	R	R	S
H132540044	London	UK	lineage4	R	R	n/a	n/a	R	R	R	U
H104020004	London	UK	lineage4	R	R	R	R	R	R	R	R
H123460050	London	UK	Delhi	R	R	n/a	R	R	R	R	R
H112880076	London	UK	EAI	R	R	S	S	R	R	S	S
H113020004	London	UK	lineage4	R	R	n/a	S	R	R	R	S
H123460044	London	UK	Beijing	R	R	R	S	R	R	R	R
H122020037	London	UK	Tur	R	R	S	S	R	R	S	S
H102640056	London	UK	lineage4	R	R	n/a	S	R	R	R	R
H114400013	London	UK	LAM	R	R	n/a	S	R	R	R	R
H114740004	London	UK	Delhi	R	R	n/a	S	R	R	R	R
H144400047	London	UK	EAI	R	R	S	S	R	R	S	S
H111540004	London	UK	Beijing	R	R	n/a	R	R	R	R	R
H112860025	London	UK	Beijing	R	R	R	S	R	R	R	R
H122860049	London	UK	LAM	R	R	n/a	S	R	R	R	S
H105160152	London	UK	Cameroon	S	S	S	S	S	S	S	S
H131780007	London	UK	Beijing	R	R	S	S	R	R	R	R
H140300080	London	UK	LAM	R	R	n/a	S	R	R	R	S
H144000191	London	UK	LAM	R	R	n/a	R	R	R	U	R
H124160071	London	UK	Delhi	R	R	n/a	S	R	R	R	S
H110200006	London	UK	Beijing	S	n/a	S	S	S	S	S	S
H133020037	London	UK	Cameroon	R	R	n/a	n/a	R	R	S	S
H131320020	London	UK	Beijing	R	R	R	R	R	R	R	R
H110720021	London	UK	Beijing	R	R	R	R	R	R	R	R
H123660058	London	UK	X-type	R	R	n/a	S	R	R	R	R
H122100024	London	UK	Tur	R	R	S	S	R	R	S	S
H132560017	London	UK	Beijing	R	R	R	S	R	R	R	R
H124720152	London	UK	LAM	R	R	R	S	R	R	R	R
H144800311	London	UK	Beijing	R	R	S	R	R	R	R	U
H104480049	London	UK	Beijing	S	S	S	S	S	S	S	S
H130600165	London	UK	lineage4	R	R	n/a	S	R	R	R	S
H104500470	London	UK	Beijing	R	R	R	R	R	R	R	R
H120800010	London	UK	Cameroon	R	R	n/a	S	R	R	R	U
H144720288	London	UK	S-type	R	R	R	S	R	R	R	S
H143120831	London	UK	Delhi	R	R	S	R	R	R	R	R
H141860035	London	UK	Tur	R	R	S	S	R	R	S	S
H130260030	London	UK	Beijing	R	R	R	R	R	R	R	R
H104840010	London	UK	Cameroon	R	R	n/a	S	R	R	R	S
H122660008	London	UK	Haarlem	R	R	R	S	R	R	R	S
H130560193	London	UK	Beijing	R	R	S	S	R	R	R	F
H131460076	London	UK	Beijing	R	R	n/a	R	R	R	R	U
H103540353	London	UK	Beijing	R	R	n/a	R	R	R	R	R
H112980113	London	UK	Beijing	R	R	n/a	R	R	R	R	R
H122280029	London	UK	lineage4	R	R	R	S	R	R	S	U
H112700034	London	UK	Beijing	R	R	R	S	R	R	R	R
H104560351	London	UK	Beijing	R	R	R	R	R	R	R	R
H130380145	London	UK	Beijing	R	R	n/a	R	R	R	R	R
H114920016	London	UK	Beijing	R	R	n/a	S	R	R	R	R
H125180095	London	UK	Cameroon	R	R	R	R	R	R	F	R
H132100029	London	UK	EAI	R	R	n/a	S	R	R	R	S
H133600015	London	UK	Cameroon	R	R	S	S	R	R	S	S
H112760022	London	UK	Delhi	R	R	S	S	R	R	S	S
H101460141	London	UK	Delhi	R	R	n/a	n/a	R	R	R	S
H102040119	London	UK	LAM	R	R	n/a	R	R	R	R	R
H144060098	London	UK	Beijing	R	R	R	S	R	R	R	R
H114240009	London	UK	Cameroon	S	S	S	S	S	S	S	S
H122280030	London	UK	Haarlem	R	R	R	R	R	R	R	U
H112260025	London	UK	Beijing	R	R	R	R	R	R	R	R
H150240214	London	UK	Ural	R	R	n/a	R	R	R	R	R
H134560426	London	UK	Delhi	R	R	n/a	R	R	R	R	R
H131740058	London	UK	EAI	R	R	n/a	S	R	R	R	S
H141220064	London	UK	Beijing	R	R	R	R	R	R	R	R
H133260002	London	UK	Delhi	R	n/a	S	S	R	R	R	S
H115120020	London	UK	Beijing	R	R	R	R	R	R	R	R
H131440121	London	UK	Haarlem	R	R	n/a	n/a	R	R	U	R
H123660062	London	UK	Delhi	R	R	n/a	S	R	R	R	R
H134340142	London	UK	Delhi	R	R	n/a	R	R	R	R	R
H111880072	London	UK	Beijing	R	R	R	S	R	R	R	R
H122780021	London	UK	Beijing	R	R	R	R	R	R	R	R
H143880210	London	UK	Beijing	R	R	n/a	S	R	R	R	R
H122020014	London	UK	Haarlem	R	R	S	S	R	R	S	S
H133700018	London	UK	lineage4	R	R	R	R	R	R	R	R
H134460397	London	UK	Beijing	R	R	n/a	R	R	R	R	R
H111060034	London	UK	Beijing	R	R	n/a	R	R	R	R	R
H130520152	London	UK	Beijing	R	R	R	R	R	R	R	R
H134060565	London	UK	Delhi	R	R	R	S	R	R	R	S
H115180008	London	UK	Beijing	R	R	R	R	R	R	R	R
H142680039	London	UK	Beijing	R	R	R	R	R	R	R	R
H133460009	London	UK	Beijing	R	R	n/a	R	R	R	R	R
H121400003	London	UK	Beijing	R	R	R	R	R	R	R	R
H102060503	London	UK	Delhi	R	R	n/a	S	R	R	R	S
H120320024	London	UK	EAI	R	R	R	R	R	R	R	R
H103380436	London	UK	lineage4	R	R	R	R	R	R	R	R
H141480065	London	UK	Delhi	R	R	n/a	S	R	R	R	R
H101480093	London	UK	Cameroon	R	R	S	S	R	R	S	S
H101320216	London	UK	Beijing	R	R	S	R	R	R	S	S
H142640017	London	UK	Delhi	R	R	R	S	R	R	R	R
H150140251	London	UK	Delhi	R	R	n/a	S	R	R	U	U
H133740011	London	UK	Cameroon	R	R	S	S	R	R	S	S
H121460028	London	UK	Delhi	R	R	S	S	R	R	S	S
H142180021	London	UK	Beijing	R	R	S	S	R	R	S	S
H140120166	London	UK	Cameroon	R	R	S	S	R	R	S	S
H110380009	London	UK	Ural	R	R	S	S	R	R	S	S
H111100025	London	UK	Cameroon	S	S	S	S	S	S	S	S
H130140042	London	UK	EAI	R	R	R	S	R	R	R	S
H132340027	London	UK	Beijing	R	R	S	R	R	R	R	U
H112260020	London	UK	Beijing	R	R	n/a	R	R	R	U	R
H145180186	London	UK	Delhi	R	R	S	S	U	S	S	S
H143340168	London	UK	Beijing	R	R	S	S	R	R	S	R
H141960036	London	UK	Beijing	R	R	S	S	R	R	S	F
H122240093	London	UK	Beijing	R	R	S	S	R	R	S	S
H102800104	London	UK	Beijing	R	R	n/a	S	R	R	R	S
H131140031	London	UK	Delhi	R	R	S	S	R	R	S	S
H150280022	London	UK	LAM	R	R	n/a	S	R	R	U	R
H113760006	London	UK	Delhi	R	R	n/a	R	R	R	R	U

H112420348	London	UK	lineage4	R	R	S	S	R	R	R	S
H144400036	London	UK	Delhi	R	R	S	S	R	R	S	S
H104640024	London	UK	LAM	R	R	S	n/a	R	R	R	U
H104420157	London	UK	S-type	R	R	n/a	R	R	R	R	R
H130980067	London	UK	Beijing	R	R	n/a	R	R	R	R	R
H112580038	London	UK	Beijing	R	R	R	R	R	R	R	U
H142480019	London	UK	Delhi	n/a	S	S	S	F	S	S	F
H132920061	London	UK	Beijing	R	R	n/a	S	R	R	R	R
H122680006	London	UK	Beijing	S	S	S	S	S	S	S	S
H131020009	London	UK	Beijing	S	S	S	S	S	S	S	S
H122800063	London	UK	Beijing	R	R	n/a	S	R	R	R	S
H143540187	London	UK	Beijing	R	R	R	R	R	R	R	R
H112900016	London	UK	Beijing	n/a	R	S	R	R	R	S	R
H114840035	London	UK	Delhi	R	R	n/a	R	R	R	R	R
H111200010	London	UK	Cameroon	R	R	n/a	S	R	R	R	S
H111540283	London	UK	lineage4	R	R	n/a	S	R	R	R	S
H123320016	London	UK	Delhi	S	S	S	S	F	S	F	S
H124940231	London	UK	Beijing	R	R	R	S	R	R	R	S
H122160005	London	UK	Delhi	R	R	S	S	R	R	U	S
H110800119	London	UK	Beijing	S	S	S	S	S	S	S	S
H102500149	London	UK	LAM	R	R	n/a	S	R	R	R	U
H103060006	London	UK	Delhi	R	R	S	R	R	R	R	R
H120380038	London	UK	Uganda	R	R	n/a	S	R	R	S	S
H130240005	London	UK	Ural	R	n/a	S	S	R	R	S	S
H102360099	London	UK	EAI	R	R	R	S	R	R	R	S
H111140023	London	UK	Delhi	R	R	n/a	R	R	R	R	R
H101740076	London	UK	Delhi	R	R	S	S	R	R	S	S
H124600163	London	UK	Tur	R	R	S	R	R	R	R	R
H132740087	London	UK	Beijing	R	R	R	S	R	R	R	R
H131920005	London	UK	Beijing	R	R	n/a	R	R	R	R	R
H142440048	London	UK	Beijing	S	S	S	S	S	S	S	S
H121760053	London	UK	Cameroon	n/a	R	S	S	R	R	S	S
H105100002	London	UK	Delhi	R	R	S	R	R	R	R	R
H114440076	London	UK	LAM	R	R	n/a	S	R	R	R	R
H112540008	London	UK	Cameroon	R	R	n/a	S	R	R	R	S
H102560096	London	UK	lineage4	R	R	n/a	n/a	R	R	R	R
H103860005	London	UK	EAI	R	R	S	S	R	R	S	S
H110740021	London	UK	Beijing	R	R	S	S	R	R	S	S
H133000022	London	UK	lineage4	R	R	n/a	S	R	R	R	S
H123240040	London	UK	Delhi	R	R	n/a	S	R	R	R	S
H113980021	London	UK	EAI	R	R	R	S	R	R	R	R
H131820988	London	UK	Beijing	R	R	n/a	R	R	R	R	R
H104800203	London	UK	lineage4	R	R	n/a	S	S	R	R	S
H112020004	London	UK	Cameroon	R	R	S	S	R	R	S	S
H111100143	London	UK	EAI	R	R	n/a	n/a	R	R	U	R
H114960023	London	UK	Beijing	R	n/a	R	R	R	R	R	R
H131360146	London	UK	Haarlem	R	R	S	S	R	R	S	S
H103520338	London	UK	Beijing	R	R	R	S	R	R	R	R
H112640132	London	UK	Beijing	R	R	R	S	R	R	R	S
H142980133	London	UK	Delhi	R	R	S	S	R	R	S	S
H130600143	London	UK	Beijing	R	R	R	R	R	R	R	R
H110580004	London	UK	Delhi	R	R	n/a	S	R	R	R	R
H121920020	London	UK	Cameroon	R	R	S	S	R	R	S	S
H131440141	London	UK	Delhi	R	R	n/a	S	R	R	R	R
H131280121	London	UK	Beijing	R	R	n/a	n/a	R	F	S	R
H101880135	London	UK	Beijing	R	R	n/a	R	R	R	R	U
H132620007	London	UK	Beijing	R	R	n/a	S	R	R	R	R
H143580008	London	UK	Beijing	R	R	n/a	R	R	R	R	R
H130600152	London	UK	Tur	R	R	n/a	R	R	R	F	R
H104960117	London	UK	lineage4	R	R	n/a	S	S	R	R	S
H142060040	London	UK	M. orygis	S	S	S	S	F	S	S	S
H111960042	London	UK	EAI	R	R	S	S	R	R	S	S
H130160090	London	UK	EAI	R	R	S	S	R	R	S	S
H140700024	London	UK	Beijing	S	S	S	S	S	S	S	S
H115260013	London	UK	Beijing	R	R	n/a	n/a	R	R	R	S
H120160158	London	UK	LAM	R	R	R	S	R	R	R	S
H133580033	London	UK	Beijing	R	R	R	R	R	R	R	R
H112020026	London	UK	Delhi	S	S	S	S	S	F	S	S
H110640051	London	UK	Delhi	R	R	S	S	R	R	S	S
H120840035	London	UK	Haarlem	R	R	S	S	R	R	S	S
H113560505	London	UK	Beijing	R	R	n/a	R	R	R	U	R
H130800564	London	UK	EAI	R	R	n/a	S	R	R	U	S
H113240071	London	UK	Beijing	R	R	n/a	n/a	R	R	R	S
H125180079	London	UK	Haarlem	R	R	S	S	R	R	S	S
H121580062	London	UK	Delhi	R	R	n/a	n/a	R	R	R	R
H130800044	London	UK	Beijing	R	R	n/a	S	R	R	R	R
H102760384	London	UK	Beijing	R	R	R	R	R	R	R	R
H130840124	London	UK	Beijing	R	R	n/a	S	R	R	S	S
H101860438	London	UK	lineage4	R	R	S	S	R	R	S	S
H120640724	London	UK	EAI	R	R	S	S	R	R	S	S
H133020025	London	UK	Beijing	R	R	R	R	R	R	R	R
H112440006	London	UK	Delhi	R	R	S	R	R	R	R	R
H130260029	London	UK	EAI	R	R	S	S	R	R	S	S
H113240102	London	UK	Beijing	R	R	n/a	R	R	R	R	R
T24097_OxRet_57	Oxford	UK	Beijing	S	S	S	S	U	F	U	S
H65996	Oxford	UK	LAM	S	S	S	S	S	S	U	S
L91007	Oxford	UK	Delhi	S	S	S	S	S	F	S	S
H38567_GrandRound_1	Oxford	UK	Delhi	S	S	S	S	F	S	S	S
H61918	Oxford	UK	Beijing	S	S	S	S	S	S	S	S
M43317	Oxford	UK	Delhi	S	S	S	S	S	S	S	S
L89004	Oxford	UK	Beijing	R	R	n/a	S	R	R	R	S
W73987	Oxford	UK	Delhi	S	S	S	S	S	S	S	S
W80004	Oxford	UK	Delhi	S	S	S	S	S	S	S	S
W67458	Oxford	UK	Beijing	S	S	S	S	S	S	S	S
H38567_OxPro_068	Oxford	UK	Delhi	S	S	S	S	S	S	S	S
OxTb-431	Oxford	UK	Delhi	S	S	S	S	S	S	S	S
H72959	Oxford	UK	Delhi	S	S	S	S	S	S	S	S
L70379	Oxford	UK	lineage4	R	S	S	S	U	S	S	S
M29220_OxPro_054	Oxford	UK	X-type	S	S	S	S	S	S	S	S
L99100	Oxford	UK	Beijing	S	S	S	S	S	S	S	S
M37617	Oxford	UK	lineage4	S	S	S	S	S	S	S	S
H69550	Oxford	UK	Beijing	S	S	S	S	S	S	S	S
W2224	Oxford	UK	Beijing	S	S	S	S	S	S	S	S
L93729	Oxford	UK	Delhi	S	S	S	S	F	F	U	S
M46124	Oxford	UK	LAM	R	S	S	S	R	S	S	S
OxTb-427	Oxford	UK	EAI	S	S	S	S	S	S	U	S
OxTb-430	Oxford	UK	Beijing	S	S	S	S	S	S	U	S
F70275_E2	Oxford	UK	Beijing	S	S	S	S	F	S	S	F
W78132	Oxford	UK	Delhi	S	S	S	S	S	S	S	S
F41766_OxRet_216	Oxford	UK	EAI	S	S	S	S	S	S	S	S
W78432	Oxford	UK	Beijing	R	R	R	S	R	R	R	R
W73990_E3	Oxford	UK	Delhi	S	S	S	S	S	S	S	S
L99797	Oxford	UK	lineage4	R	S	S	S	U	S	S	S
W39592_OxPro_9	Oxford	UK	X-type	S	S	S	S	F	S	S	F
L94062	Oxford	UK	lineage4	R	S	S	S	U	S	S	S
T28530	Oxford	UK	lineage4	S	S	S	S	S	S	U	S

H74998	Oxford	UK	Beijing			S	S	S	S	S	S	S	S
T20779	Oxford	UK	Delhi	SRR6367395	PRJNA393378	S	S	S	S	S	S	S	S
T23882	Oxford	UK	Delhi	SRR6339663	PRJNA393378	S	S	S	S	U	S	S	F

S2

Gene coordinates based on NC_000962.2, with 100 nucleotide positions upstream of each gene read as well.
Mutations characterised as 'S' in Walker *et al* but as 'R' by another source, were characterised as 'R'.
Insertions and deletions characterise in Walker *et al* were re-computed from that data for this study to ensure that the same version of Cortex was used for both data sets. These indels may therefore differ a little from those published in Walker *et al.*

Drug	Mutation	Details of insertion/deletion	Characterisation	Source
Ethambutol	embA_2723_indel	embA_2723_2724_ins_GGG	S	Walker TM et. al., Lancet Infect Dis. 2015 Jun 23
Ethambutol	embA_A1015T		S	Walker TM et. al., Lancet Infect Dis. 2015 Jun 23
Ethambutol	embA_A1016S		S	Walker TM et. al., Lancet Infect Dis. 2015 Jun 23
Ethambutol	embA_A109T		S	Walker TM et. al., Lancet Infect Dis. 2015 Jun 23
Ethambutol	embA_A201T		S	Walker TM et. al., Lancet Infect Dis. 2015 Jun 23
Ethambutol	embA_A255V		S	Walker TM et. al., Lancet Infect Dis. 2015 Jun 23
Ethambutol	embA_A312V		S	Walker TM et. al., Lancet Infect Dis. 2015 Jun 23
Ethambutol	embA_A331P		S	Walker TM et. al., Lancet Infect Dis. 2015 Jun 23
Ethambutol	embA_A428V		S	Walker TM et. al., Lancet Infect Dis. 2015 Jun 23
Ethambutol	embA_A438V		S	Walker TM et. al., Lancet Infect Dis. 2015 Jun 23
Ethambutol	embA_A460T		S	Walker TM et. al., Lancet Infect Dis. 2015 Jun 23
Ethambutol	embA_A545P		S	Walker TM et. al., Lancet Infect Dis. 2015 Jun 23
Ethambutol	embA_A576T		S	Walker TM et. al., Lancet Infect Dis. 2015 Jun 23
Ethambutol	embA_A586T		S	Walker TM et. al., Lancet Infect Dis. 2015 Jun 23
Ethambutol	embA_A734T		S	Walker TM et. al., Lancet Infect Dis. 2015 Jun 23
Ethambutol	embA_A852S		S	Walker TM et. al., Lancet Infect Dis. 2015 Jun 23
Ethambutol	embA_A852V		S	Walker TM et. al., Lancet Infect Dis. 2015 Jun 23
Ethambutol	embA_C-12T		R	Walker TM et. al., Lancet Infect Dis. 2015 Jun 23
Ethambutol	embA_C-15G		S	Walker TM et. al., Lancet Infect Dis. 2015 Jun 23
Ethambutol	embA_C-16G		R	Walker TM et. al., Lancet Infect Dis. 2015 Jun 23
Ethambutol	embA_C-16T		R	Walker TM et. al., Lancet Infect Dis. 2015 Jun 23
Ethambutol	embA_C-59A		S	Walker TM et. al., Lancet Infect Dis. 2015 Jun 23
Ethambutol	embA_C-73T		S	Walker TM et. al., Lancet Infect Dis. 2015 Jun 23
Ethambutol	embA_C335W		S	Walker TM et. al., Lancet Infect Dis. 2015 Jun 23
Ethambutol	embA_D1053G		S	Walker TM et. al., Lancet Infect Dis. 2015 Jun 23
Ethambutol	embA_D176A		S	Walker TM et. al., Lancet Infect Dis. 2015 Jun 23
Ethambutol	embA_D678N		S	Walker TM et. al., Lancet Infect Dis. 2015 Jun 23
Ethambutol	embA_D761G		S	Walker TM et. al., Lancet Infect Dis. 2015 Jun 23
Ethambutol	embA_D784N		S	Walker TM et. al., Lancet Infect Dis. 2015 Jun 23
Ethambutol	embA_D865N		S	Walker TM et. al., Lancet Infect Dis. 2015 Jun 23
Ethambutol	embA_E7K		S	Walker TM et. al., Lancet Infect Dis. 2015 Jun 23
Ethambutol	embA_E951D		S	Walker TM et. al., Lancet Infect Dis. 2015 Jun 23
Ethambutol	embA_G-17A		S	Walker TM et. al., Lancet Infect Dis. 2015 Jun 23
Ethambutol	embA_G-43C		S	Walker TM et. al., Lancet Infect Dis. 2015 Jun 23
Ethambutol	embA_G1085S		S	Walker TM et. al., Lancet Infect Dis. 2015 Jun 23
Ethambutol	embA_G154S		S	Walker TM et. al., Lancet Infect Dis. 2015 Jun 23
Ethambutol	embA_G157C		S	Walker TM et. al., Lancet Infect Dis. 2015 Jun 23
Ethambutol	embA_G21R		S	Walker TM et. al., Lancet Infect Dis. 2015 Jun 23
Ethambutol	embA_G352S		S	Walker TM et. al., Lancet Infect Dis. 2015 Jun 23
Ethambutol	embA_G596S		S	Walker TM et. al., Lancet Infect Dis. 2015 Jun 23
Ethambutol	embA_G884D		S	Walker TM et. al., Lancet Infect Dis. 2015 Jun 23
Ethambutol	embA_H665R		S	Walker TM et. al., Lancet Infect Dis. 2015 Jun 23
Ethambutol	embA_H665Y		S	Walker TM et. al., Lancet Infect Dis. 2015 Jun 23
Ethambutol	embA_H673R		S	Walker TM et. al., Lancet Infect Dis. 2015 Jun 23
Ethambutol	embA_I191V		S	Walker TM et. al., Lancet Infect Dis. 2015 Jun 23
Ethambutol	embA_I595V		S	Walker TM et. al., Lancet Infect Dis. 2015 Jun 23
Ethambutol	embA_K166N		S	Walker TM et. al., Lancet Infect Dis. 2015 Jun 23
Ethambutol	embA_K773N		S	Walker TM et. al., Lancet Infect Dis. 2015 Jun 23
Ethambutol	embA_L1008P		S	Walker TM et. al., Lancet Infect Dis. 2015 Jun 23
Ethambutol	embA_L215M		S	Walker TM et. al., Lancet Infect Dis. 2015 Jun 23
Ethambutol	embA_L215P		S	Walker TM et. al., Lancet Infect Dis. 2015 Jun 23
Ethambutol	embA_L233M		S	Walker TM et. al., Lancet Infect Dis. 2015 Jun 23
Ethambutol	embA_L263F		S	Walker TM et. al., Lancet Infect Dis. 2015 Jun 23
Ethambutol	embA_L263P		S	Walker TM et. al., Lancet Infect Dis. 2015 Jun 23
Ethambutol	embA_L314P		S	Walker TM et. al., Lancet Infect Dis. 2015 Jun 23
Ethambutol	embA_L373P		S	Walker TM et. al., Lancet Infect Dis. 2015 Jun 23
Ethambutol	embA_L659F		S	Walker TM et. al., Lancet Infect Dis. 2015 Jun 23
Ethambutol	embA_M697V		S	Walker TM et. al., Lancet Infect Dis. 2015 Jun 23
Ethambutol	embA_N54S		S	Walker TM et. al., Lancet Infect Dis. 2015 Jun 23
Ethambutol	embA_P1094S		S	Walker TM et. al., Lancet Infect Dis. 2015 Jun 23
Ethambutol	embA_P327H		S	Walker TM et. al., Lancet Infect Dis. 2015 Jun 23
Ethambutol	embA_P327L		S	Walker TM et. al., Lancet Infect Dis. 2015 Jun 23
Ethambutol	embA_P35Q		S	Walker TM et. al., Lancet Infect Dis. 2015 Jun 23
Ethambutol	embA_P689L		S	Walker TM et. al., Lancet Infect Dis. 2015 Jun 23
Ethambutol	embA_P75L		S	Walker TM et. al., Lancet Infect Dis. 2015 Jun 23
Ethambutol	embA_P860L		S	Walker TM et. al., Lancet Infect Dis. 2015 Jun 23
Ethambutol	embA_P901L		S	Walker TM et. al., Lancet Infect Dis. 2015 Jun 23
Ethambutol	embA_Q1004P		S	Walker TM et. al., Lancet Infect Dis. 2015 Jun 23
Ethambutol	embA_Q57E		S	Walker TM et. al., Lancet Infect Dis. 2015 Jun 23
Ethambutol	embA_R683L		S	Walker TM et. al., Lancet Infect Dis. 2015 Jun 23
Ethambutol	embA_S1017A		S	Walker TM et. al., Lancet Infect Dis. 2015 Jun 23
Ethambutol	embA_S20A		S	Walker TM et. al., Lancet Infect Dis. 2015 Jun 23
Ethambutol	embA_S20P		S	Walker TM et. al., Lancet Infect Dis. 2015 Jun 23
Ethambutol	embA_S49R		S	Walker TM et. al., Lancet Infect Dis. 2015 Jun 23
Ethambutol	embA_S49T		S	Walker TM et. al., Lancet Infect Dis. 2015 Jun 23

Ethambutol	embA_S77P	S	Walker TM et. al., Lancet Infect Dis. 2015 Jun 23
Ethambutol	embA_T113R	S	Walker TM et. al., Lancet Infect Dis. 2015 Jun 23
Ethambutol	embA_T161I	S	Walker TM et. al., Lancet Infect Dis. 2015 Jun 23
Ethambutol	embA_T238N	S	Walker TM et. al., Lancet Infect Dis. 2015 Jun 23
Ethambutol	embA_T238S	S	Walker TM et. al., Lancet Infect Dis. 2015 Jun 23
Ethambutol	embA_T308A	S	Walker TM et. al., Lancet Infect Dis. 2015 Jun 23
Ethambutol	embA_T363I	S	Walker TM et. al., Lancet Infect Dis. 2015 Jun 23
Ethambutol	embA_T478A	S	Walker TM et. al., Lancet Infect Dis. 2015 Jun 23
Ethambutol	embA_T56P	S	Walker TM et. al., Lancet Infect Dis. 2015 Jun 23
Ethambutol	embA_T591A	S	Walker TM et. al., Lancet Infect Dis. 2015 Jun 23
Ethambutol	embA_T591P	S	Walker TM et. al., Lancet Infect Dis. 2015 Jun 23
Ethambutol	embA_T728I	S	Walker TM et. al., Lancet Infect Dis. 2015 Jun 23
Ethambutol	embA_T983A	S	Walker TM et. al., Lancet Infect Dis. 2015 Jun 23
Ethambutol	embA_V1078M	S	Walker TM et. al., Lancet Infect Dis. 2015 Jun 23
Ethambutol	embA_V31A	S	Walker TM et. al., Lancet Infect Dis. 2015 Jun 23
Ethambutol	embA_V391I	S	Walker TM et. al., Lancet Infect Dis. 2015 Jun 23
Ethambutol	embA_V414M	S	Walker TM et. al., Lancet Infect Dis. 2015 Jun 23
Ethambutol	embA_V698A	S	Walker TM et. al., Lancet Infect Dis. 2015 Jun 23
Ethambutol	embA_V698I	S	Walker TM et. al., Lancet Infect Dis. 2015 Jun 23
Ethambutol	embA_V911I	S	Walker TM et. al., Lancet Infect Dis. 2015 Jun 23
Ethambutol	embA_W1050G	S	Walker TM et. al., Lancet Infect Dis. 2015 Jun 23
Ethambutol	embA_W306R	S	Walker TM et. al., Lancet Infect Dis. 2015 Jun 23
Ethambutol	embA_Y296H	S	Walker TM et. al., Lancet Infect Dis. 2015 Jun 23
Ethambutol	embA_Y307H	S	Walker TM et. al., Lancet Infect Dis. 2015 Jun 23
Ethambutol	embB_2943_indel embB_2943_2945_del_GCA	S	Walker TM et. al., Lancet Infect Dis. 2015 Jun 23
Ethambutol	embB_A221T	S	Walker TM et. al., Lancet Infect Dis. 2015 Jun 23
Ethambutol	embB_A259V	S	Walker TM et. al., Lancet Infect Dis. 2015 Jun 23
Ethambutol	embB_A386E	S	Walker TM et. al., Lancet Infect Dis. 2015 Jun 23
Ethambutol	embB_A388V	S	Walker TM et. al., Lancet Infect Dis. 2015 Jun 23
Ethambutol	embB_A438T	S	Walker TM et. al., Lancet Infect Dis. 2015 Jun 23
Ethambutol	embB_A454T	S	Walker TM et. al., Lancet Infect Dis. 2015 Jun 23
Ethambutol	embB_A510T	S	Walker TM et. al., Lancet Infect Dis. 2015 Jun 23
Ethambutol	embB_A547S	S	Walker TM et. al., Lancet Infect Dis. 2015 Jun 23
Ethambutol	embB_A680T	S	Walker TM et. al., Lancet Infect Dis. 2015 Jun 23
Ethambutol	embB_A683V	S	Walker TM et. al., Lancet Infect Dis. 2015 Jun 23
Ethambutol	embB_A693T	S	Walker TM et. al., Lancet Infect Dis. 2015 Jun 23
Ethambutol	embB_A701T	S	Walker TM et. al., Lancet Infect Dis. 2015 Jun 23
Ethambutol	embB_A840P	S	Walker TM et. al., Lancet Infect Dis. 2015 Jun 23
Ethambutol	embB_A861T	S	Walker TM et. al., Lancet Infect Dis. 2015 Jun 23
Ethambutol	embB_A913V	S	Walker TM et. al., Lancet Infect Dis. 2015 Jun 23
Ethambutol	embB_A950V	S	Walker TM et. al., Lancet Infect Dis. 2015 Jun 23
Ethambutol	embB_C-2T	S	Walker TM et. al., Lancet Infect Dis. 2015 Jun 23
Ethambutol	embB_C361S	S	Walker TM et. al., Lancet Infect Dis. 2015 Jun 23
Ethambutol	embB_D1024N	S	Walker TM et. al., Lancet Infect Dis. 2015 Jun 23
Ethambutol	embB_D1056E	S	Walker TM et. al., Lancet Infect Dis. 2015 Jun 23
Ethambutol	embB_D311A	S	Walker TM et. al., Lancet Infect Dis. 2015 Jun 23
Ethambutol	embB_D328H	S	Walker TM et. al., Lancet Infect Dis. 2015 Jun 23
Ethambutol	embB_D328Y	R	Walker TM et. al., Lancet Infect Dis. 2015 Jun 23
Ethambutol	embB_D354A	R	Walker TM et. al., Lancet Infect Dis. 2015 Jun 23
Ethambutol	embB_D354G	S	Walker TM et. al., Lancet Infect Dis. 2015 Jun 23
Ethambutol	embB_D78E	S	Walker TM et. al., Lancet Infect Dis. 2015 Jun 23
Ethambutol	embB_D78G	S	Walker TM et. al., Lancet Infect Dis. 2015 Jun 23
Ethambutol	embB_D869H	S	Walker TM et. al., Lancet Infect Dis. 2015 Jun 23
Ethambutol	embB_D86N	S	Walker TM et. al., Lancet Infect Dis. 2015 Jun 23
Ethambutol	embB_D870N	S	Walker TM et. al., Lancet Infect Dis. 2015 Jun 23
Ethambutol	embB_E504D	S	Walker TM et. al., Lancet Infect Dis. 2015 Jun 23
Ethambutol	embB_E951Q	S	Walker TM et. al., Lancet Infect Dis. 2015 Jun 23
Ethambutol	embB_F1012L	S	Walker TM et. al., Lancet Infect Dis. 2015 Jun 23
Ethambutol	embB_F161L	S	Walker TM et. al., Lancet Infect Dis. 2015 Jun 23
Ethambutol	embB_F285L	S	Walker TM et. al., Lancet Infect Dis. 2015 Jun 23
Ethambutol	embB_F628S	S	Walker TM et. al., Lancet Infect Dis. 2015 Jun 23
Ethambutol	embB_F676S	S	Walker TM et. al., Lancet Infect Dis. 2015 Jun 23
Ethambutol	embB_G-29A	S	Walker TM et. al., Lancet Infect Dis. 2015 Jun 23
Ethambutol	embB_G-50A	S	Walker TM et. al., Lancet Infect Dis. 2015 Jun 23
Ethambutol	embB_G-6A	S	Walker TM et. al., Lancet Infect Dis. 2015 Jun 23
Ethambutol	embB_G-90A	S	Walker TM et. al., Lancet Infect Dis. 2015 Jun 23
Ethambutol	embB_G100S	S	Walker TM et. al., Lancet Infect Dis. 2015 Jun 23
Ethambutol	embB_G1097S	S	Walker TM et. al., Lancet Infect Dis. 2015 Jun 23
Ethambutol	embB_G246R	S	Walker TM et. al., Lancet Infect Dis. 2015 Jun 23
Ethambutol	embB_G263R	S	Walker TM et. al., Lancet Infect Dis. 2015 Jun 23
Ethambutol	embB_G305C	S	Walker TM et. al., Lancet Infect Dis. 2015 Jun 23
Ethambutol	embB_G37S	S	Walker TM et. al., Lancet Infect Dis. 2015 Jun 23
Ethambutol	embB_G406A	R	Walker TM et. al., Lancet Infect Dis. 2015 Jun 23
Ethambutol	embB_G406D	R	Walker TM et. al., Lancet Infect Dis. 2015 Jun 23
Ethambutol	embB_G406S	R	Walker TM et. al., Lancet Infect Dis. 2015 Jun 23
Ethambutol	embB_G603R	S	Walker TM et. al., Lancet Infect Dis. 2015 Jun 23
Ethambutol	embB_G62R	S	Walker TM et. al., Lancet Infect Dis. 2015 Jun 23
Ethambutol	embB_G665R	S	Walker TM et. al., Lancet Infect Dis. 2015 Jun 23
Ethambutol	embB_G694S	S	Walker TM et. al., Lancet Infect Dis. 2015 Jun 23
Ethambutol	embB_G748E	S	Walker TM et. al., Lancet Infect Dis. 2015 Jun 23
Ethambutol	embB_G836R	S	Walker TM et. al., Lancet Infect Dis. 2015 Jun 23

Ethambutol	embB_H1002R	S	Walker TM et. al., Lancet Infect Dis. 2015 Jun 23
Ethambutol	embB_H342N	S	Walker TM et. al., Lancet Infect Dis. 2015 Jun 23
Ethambutol	embB_I1006M	S	Walker TM et. al., Lancet Infect Dis. 2015 Jun 23
Ethambutol	embB_I16L	S	Walker TM et. al., Lancet Infect Dis. 2015 Jun 23
Ethambutol	embB_K107R	S	Walker TM et. al., Lancet Infect Dis. 2015 Jun 23
Ethambutol	embB_K820T	S	Walker TM et. al., Lancet Infect Dis. 2015 Jun 23
Ethambutol	embB_K882T	S	Walker TM et. al., Lancet Infect Dis. 2015 Jun 23
Ethambutol	embB_L1037I	S	Walker TM et. al., Lancet Infect Dis. 2015 Jun 23
Ethambutol	embB_L253I	S	Walker TM et. al., Lancet Infect Dis. 2015 Jun 23
Ethambutol	embB_L348P	S	Walker TM et. al., Lancet Infect Dis. 2015 Jun 23
Ethambutol	embB_L370R	S	Walker TM et. al., Lancet Infect Dis. 2015 Jun 23
Ethambutol	embB_L638F	S	Walker TM et. al., Lancet Infect Dis. 2015 Jun 23
Ethambutol	embB_L686P	S	Walker TM et. al., Lancet Infect Dis. 2015 Jun 23
Ethambutol	embB_L971M	S	Walker TM et. al., Lancet Infect Dis. 2015 Jun 23
Ethambutol	embB_M1049I	S	Walker TM et. al., Lancet Infect Dis. 2015 Jun 23
Ethambutol	embB_M306I	R	Walker TM et. al., Lancet Infect Dis. 2015 Jun 23
Ethambutol	embB_M306L	S	Walker TM et. al., Lancet Infect Dis. 2015 Jun 23
Ethambutol	embB_M306V	R	Walker TM et. al., Lancet Infect Dis. 2015 Jun 23
Ethambutol	embB_M340I	S	Walker TM et. al., Lancet Infect Dis. 2015 Jun 23
Ethambutol	embB_M350T	S	Walker TM et. al., Lancet Infect Dis. 2015 Jun 23
Ethambutol	embB_M462L	S	Walker TM et. al., Lancet Infect Dis. 2015 Jun 23
Ethambutol	embB_M462T	S	Walker TM et. al., Lancet Infect Dis. 2015 Jun 23
Ethambutol	embB_M557I	S	Walker TM et. al., Lancet Infect Dis. 2015 Jun 23
Ethambutol	embB_M91I	S	Walker TM et. al., Lancet Infect Dis. 2015 Jun 23
Ethambutol	embB_N1004T	S	Walker TM et. al., Lancet Infect Dis. 2015 Jun 23
Ethambutol	embB_N1033K	R	Walker TM et. al., Lancet Infect Dis. 2015 Jun 23
Ethambutol	embB_N13S	S	Walker TM et. al., Lancet Infect Dis. 2015 Jun 23
Ethambutol	embB_N400S	S	Walker TM et. al., Lancet Infect Dis. 2015 Jun 23
Ethambutol	embB_N657D	S	Walker TM et. al., Lancet Infect Dis. 2015 Jun 23
Ethambutol	embB_P103T	S	Walker TM et. al., Lancet Infect Dis. 2015 Jun 23
Ethambutol	embB_P375S	S	Walker TM et. al., Lancet Infect Dis. 2015 Jun 23
Ethambutol	embB_P430L	S	Walker TM et. al., Lancet Infect Dis. 2015 Jun 23
Ethambutol	embB_P655Q	S	Walker TM et. al., Lancet Infect Dis. 2015 Jun 23
Ethambutol	embB_P731L	S	Walker TM et. al., Lancet Infect Dis. 2015 Jun 23
Ethambutol	embB_P776L	S	Walker TM et. al., Lancet Infect Dis. 2015 Jun 23
Ethambutol	embB_P93L	S	Walker TM et. al., Lancet Infect Dis. 2015 Jun 23
Ethambutol	embB_Q445R	S	Walker TM et. al., Lancet Infect Dis. 2015 Jun 23
Ethambutol	embB_Q497K	R	Walker TM et. al., Lancet Infect Dis. 2015 Jun 23
Ethambutol	embB_Q497P	S	Walker TM et. al., Lancet Infect Dis. 2015 Jun 23
Ethambutol	embB_Q497R	R	Walker TM et. al., Lancet Infect Dis. 2015 Jun 23
Ethambutol	embB_Q51P	S	Walker TM et. al., Lancet Infect Dis. 2015 Jun 23
Ethambutol	embB_Q853P	S	Walker TM et. al., Lancet Infect Dis. 2015 Jun 23
Ethambutol	embB_R1059P	S	Walker TM et. al., Lancet Infect Dis. 2015 Jun 23
Ethambutol	embB_R147C	S	Walker TM et. al., Lancet Infect Dis. 2015 Jun 23
Ethambutol	embB_R14Q	S	Walker TM et. al., Lancet Infect Dis. 2015 Jun 23
Ethambutol	embB_R182C	S	Walker TM et. al., Lancet Infect Dis. 2015 Jun 23
Ethambutol	embB_R620C	S	Walker TM et. al., Lancet Infect Dis. 2015 Jun 23
Ethambutol	embB_R7T	S	Walker TM et. al., Lancet Infect Dis. 2015 Jun 23
Ethambutol	embB_R930H	S	Walker TM et. al., Lancet Infect Dis. 2015 Jun 23
Ethambutol	embB_S1054P	S	Walker TM et. al., Lancet Infect Dis. 2015 Jun 23
Ethambutol	embB_S119N	S	Walker TM et. al., Lancet Infect Dis. 2015 Jun 23
Ethambutol	embB_S203L	S	Walker TM et. al., Lancet Infect Dis. 2015 Jun 23
Ethambutol	embB_S317F	S	Walker TM et. al., Lancet Infect Dis. 2015 Jun 23
Ethambutol	embB_S344R	S	Walker TM et. al., Lancet Infect Dis. 2015 Jun 23
Ethambutol	embB_S422P	S	Walker TM et. al., Lancet Infect Dis. 2015 Jun 23
Ethambutol	embB_S658N	S	Walker TM et. al., Lancet Infect Dis. 2015 Jun 23
Ethambutol	embB_S658R	S	Walker TM et. al., Lancet Infect Dis. 2015 Jun 23
Ethambutol	embB_S823R	S	Walker TM et. al., Lancet Infect Dis. 2015 Jun 23
Ethambutol	embB_T1069P	S	Walker TM et. al., Lancet Infect Dis. 2015 Jun 23
Ethambutol	embB_T1082A	S	Walker TM et. al., Lancet Infect Dis. 2015 Jun 23
Ethambutol	embB_T208I	S	Walker TM et. al., Lancet Infect Dis. 2015 Jun 23
Ethambutol	embB_T341A	S	Walker TM et. al., Lancet Infect Dis. 2015 Jun 23
Ethambutol	embB_T341I	S	Walker TM et. al., Lancet Infect Dis. 2015 Jun 23
Ethambutol	embB_T341N	S	Walker TM et. al., Lancet Infect Dis. 2015 Jun 23
Ethambutol	embB_T498N	S	Walker TM et. al., Lancet Infect Dis. 2015 Jun 23
Ethambutol	embB_T546A	S	Walker TM et. al., Lancet Infect Dis. 2015 Jun 23
Ethambutol	embB_T797M	S	Walker TM et. al., Lancet Infect Dis. 2015 Jun 23
Ethambutol	embB_V1048I	S	Walker TM et. al., Lancet Infect Dis. 2015 Jun 23
Ethambutol	embB_V1071F	S	Walker TM et. al., Lancet Infect Dis. 2015 Jun 23
Ethambutol	embB_V131M	S	Walker TM et. al., Lancet Infect Dis. 2015 Jun 23
Ethambutol	embB_V135M	S	Walker TM et. al., Lancet Infect Dis. 2015 Jun 23
Ethambutol	embB_V188A	S	Walker TM et. al., Lancet Infect Dis. 2015 Jun 23
Ethambutol	embB_V230A	S	Walker TM et. al., Lancet Infect Dis. 2015 Jun 23
Ethambutol	embB_V231I	S	Walker TM et. al., Lancet Infect Dis. 2015 Jun 23
Ethambutol	embB_V283M	S	Walker TM et. al., Lancet Infect Dis. 2015 Jun 23
Ethambutol	embB_V566M	S	Walker TM et. al., Lancet Infect Dis. 2015 Jun 23
Ethambutol	embB_V602A	S	Walker TM et. al., Lancet Infect Dis. 2015 Jun 23
Ethambutol	embB_V602I	S	Walker TM et. al., Lancet Infect Dis. 2015 Jun 23
Ethambutol	embB_V668I	S	Walker TM et. al., Lancet Infect Dis. 2015 Jun 23
Ethambutol	embB_V67L	S	Walker TM et. al., Lancet Infect Dis. 2015 Jun 23
Ethambutol	embB_W273L	S	Walker TM et. al., Lancet Infect Dis. 2015 Jun 23

Ethambutol	embB_W332R	S	Walker TM et. al., Lancet Infect Dis. 2015 Jun 23
Ethambutol	embC_A1046S	S	Walker TM et. al., Lancet Infect Dis. 2015 Jun 23
Ethambutol	embC_A1073T	S	Walker TM et. al., Lancet Infect Dis. 2015 Jun 23
Ethambutol	embC_A116T	S	Walker TM et. al., Lancet Infect Dis. 2015 Jun 23
Ethambutol	embC_A232V	S	Walker TM et. al., Lancet Infect Dis. 2015 Jun 23
Ethambutol	embC_A307T	S	Walker TM et. al., Lancet Infect Dis. 2015 Jun 23
Ethambutol	embC_A33T	S	Walker TM et. al., Lancet Infect Dis. 2015 Jun 23
Ethambutol	embC_A46V	S	Walker TM et. al., Lancet Infect Dis. 2015 Jun 23
Ethambutol	embC_A597T	S	Walker TM et. al., Lancet Infect Dis. 2015 Jun 23
Ethambutol	embC_A611T	S	Walker TM et. al., Lancet Infect Dis. 2015 Jun 23
Ethambutol	embC_A711T	S	Walker TM et. al., Lancet Infect Dis. 2015 Jun 23
Ethambutol	embC_A774S	S	Walker TM et. al., Lancet Infect Dis. 2015 Jun 23
Ethambutol	embB: Any amino acid substitution at codon 306	R	WHO endorsed line probe assays
Ethambutol	embC_A887T	S	Walker TM et. al., Lancet Infect Dis. 2015 Jun 23
Ethambutol	embC_A925T	S	Walker TM et. al., Lancet Infect Dis. 2015 Jun 23
Ethambutol	embC_A940S	S	Walker TM et. al., Lancet Infect Dis. 2015 Jun 23
Ethambutol	embC_C-100T	S	Walker TM et. al., Lancet Infect Dis. 2015 Jun 23
Ethambutol	embC_C-27T	S	Walker TM et. al., Lancet Infect Dis. 2015 Jun 23
Ethambutol	embC_C355F	S	Walker TM et. al., Lancet Infect Dis. 2015 Jun 23
Ethambutol	embC_C411R	S	Walker TM et. al., Lancet Infect Dis. 2015 Jun 23
Ethambutol	embC_D252H	S	Walker TM et. al., Lancet Infect Dis. 2015 Jun 23
Ethambutol	embC_D329N	S	Walker TM et. al., Lancet Infect Dis. 2015 Jun 23
Ethambutol	embC_D490G	S	Walker TM et. al., Lancet Infect Dis. 2015 Jun 23
Ethambutol	embC_D775N	S	Walker TM et. al., Lancet Infect Dis. 2015 Jun 23
Ethambutol	embC_D948N	S	Walker TM et. al., Lancet Infect Dis. 2015 Jun 23
Ethambutol	embC_F286L	S	Walker TM et. al., Lancet Infect Dis. 2015 Jun 23
Ethambutol	embC_F67V	S	Walker TM et. al., Lancet Infect Dis. 2015 Jun 23
Ethambutol	embC_F982I	S	Walker TM et. al., Lancet Infect Dis. 2015 Jun 23
Ethambutol	embC_G-66A	S	Walker TM et. al., Lancet Infect Dis. 2015 Jun 23
Ethambutol	embC_G101A	S	Walker TM et. al., Lancet Infect Dis. 2015 Jun 23
Ethambutol	embC_G237A	S	Walker TM et. al., Lancet Infect Dis. 2015 Jun 23
Ethambutol	embC_G383D	S	Walker TM et. al., Lancet Infect Dis. 2015 Jun 23
Ethambutol	embC_G764V	S	Walker TM et. al., Lancet Infect Dis. 2015 Jun 23
Ethambutol	embC_G817D	S	Walker TM et. al., Lancet Infect Dis. 2015 Jun 23
Ethambutol	embC_G831R	S	Walker TM et. al., Lancet Infect Dis. 2015 Jun 23
Ethambutol	embC_G857S	S	Walker TM et. al., Lancet Infect Dis. 2015 Jun 23
Ethambutol	embC_G909E	S	Walker TM et. al., Lancet Infect Dis. 2015 Jun 23
Ethambutol	embC_H369R	S	Walker TM et. al., Lancet Infect Dis. 2015 Jun 23
Ethambutol	embC_H910N	S	Walker TM et. al., Lancet Infect Dis. 2015 Jun 23
Ethambutol	embC_I28V	S	Walker TM et. al., Lancet Infect Dis. 2015 Jun 23
Ethambutol	embC_I459V	S	Walker TM et. al., Lancet Infect Dis. 2015 Jun 23
Ethambutol	embC_I497M	S	Walker TM et. al., Lancet Infect Dis. 2015 Jun 23
Ethambutol	embC_I573T	S	Walker TM et. al., Lancet Infect Dis. 2015 Jun 23
Ethambutol	embC_I936V	S	Walker TM et. al., Lancet Infect Dis. 2015 Jun 23
Ethambutol	embC_K511N	S	Walker TM et. al., Lancet Infect Dis. 2015 Jun 23
Ethambutol	embC_K656N	S	Walker TM et. al., Lancet Infect Dis. 2015 Jun 23
Ethambutol	embC_L236P	S	Walker TM et. al., Lancet Infect Dis. 2015 Jun 23
Ethambutol	embC_L240P	S	Walker TM et. al., Lancet Infect Dis. 2015 Jun 23
Ethambutol	embC_L434F	S	Walker TM et. al., Lancet Infect Dis. 2015 Jun 23
Ethambutol	embC_L460R	S	Walker TM et. al., Lancet Infect Dis. 2015 Jun 23
Ethambutol	embC_M1040T	S	Walker TM et. al., Lancet Infect Dis. 2015 Jun 23
Ethambutol	embC_M234L	S	Walker TM et. al., Lancet Infect Dis. 2015 Jun 23
Ethambutol	embC_M727V	S	Walker TM et. al., Lancet Infect Dis. 2015 Jun 23
Ethambutol	embC_N127S	S	Walker TM et. al., Lancet Infect Dis. 2015 Jun 23
Ethambutol	embC_N176K	S	Walker TM et. al., Lancet Infect Dis. 2015 Jun 23
Ethambutol	embC_P1065L	S	Walker TM et. al., Lancet Infect Dis. 2015 Jun 23
Ethambutol	embC_P210S	S	Walker TM et. al., Lancet Infect Dis. 2015 Jun 23
Ethambutol	embC_P486T	S	Walker TM et. al., Lancet Infect Dis. 2015 Jun 23
Ethambutol	embC_P558R	S	Walker TM et. al., Lancet Infect Dis. 2015 Jun 23
Ethambutol	embC_P732A	S	Walker TM et. al., Lancet Infect Dis. 2015 Jun 23
Ethambutol	embC_P791T	S	Walker TM et. al., Lancet Infect Dis. 2015 Jun 23
Ethambutol	embC_P861L	S	Walker TM et. al., Lancet Infect Dis. 2015 Jun 23
Ethambutol	embC_Q1061H	S	Walker TM et. al., Lancet Infect Dis. 2015 Jun 23
Ethambutol	embC_Q491H	S	Walker TM et. al., Lancet Infect Dis. 2015 Jun 23
Ethambutol	embC_Q54H	S	Walker TM et. al., Lancet Infect Dis. 2015 Jun 23
Ethambutol	embC_Q730H	S	Walker TM et. al., Lancet Infect Dis. 2015 Jun 23
Ethambutol	embC_Q742H	S	Walker TM et. al., Lancet Infect Dis. 2015 Jun 23
Ethambutol	embC_R221C	S	Walker TM et. al., Lancet Infect Dis. 2015 Jun 23
Ethambutol	embC_R695W	S	Walker TM et. al., Lancet Infect Dis. 2015 Jun 23
Ethambutol	embC_R738W	S	Walker TM et. al., Lancet Infect Dis. 2015 Jun 23
Ethambutol	embC_R877W	S	Walker TM et. al., Lancet Infect Dis. 2015 Jun 23
Ethambutol	embC_R927H	S	Walker TM et. al., Lancet Infect Dis. 2015 Jun 23
Ethambutol	embC_R9H	S	Walker TM et. al., Lancet Infect Dis. 2015 Jun 23
Ethambutol	embC_S18F	S	Walker TM et. al., Lancet Infect Dis. 2015 Jun 23
Ethambutol	embC_S213N	S	Walker TM et. al., Lancet Infect Dis. 2015 Jun 23
Ethambutol	embC_S225R	S	Walker TM et. al., Lancet Infect Dis. 2015 Jun 23
Ethambutol	embC_T1044M	S	Walker TM et. al., Lancet Infect Dis. 2015 Jun 23
Ethambutol	embC_T1044P	S	Walker TM et. al., Lancet Infect Dis. 2015 Jun 23
Ethambutol	embC_T1079A	S	Walker TM et. al., Lancet Infect Dis. 2015 Jun 23
Ethambutol	embC_T712A	S	Walker TM et. al., Lancet Infect Dis. 2015 Jun 23
Ethambutol	embC_T786A	S	Walker TM et. al., Lancet Infect Dis. 2015 Jun 23

Ethambutol	embC_T941A		S	Walker TM et. al., Lancet Infect Dis. 2015 Jun 23
Ethambutol	embC_V218A		S	Walker TM et. al., Lancet Infect Dis. 2015 Jun 23
Ethambutol	embC_V303L		S	Walker TM et. al., Lancet Infect Dis. 2015 Jun 23
Ethambutol	embC_V337I		S	Walker TM et. al., Lancet Infect Dis. 2015 Jun 23
Ethambutol	embC_V363I		S	Walker TM et. al., Lancet Infect Dis. 2015 Jun 23
Isoniazid	ahpC_-3_indel	ahpC_-3_-2_ins_T	S	Walker TM et. al., Lancet Infect Dis. 2015 Jun 23
Isoniazid	ahpC_-34_indel	ahpC_-34_-34_del_A	S	Walker TM et. al., Lancet Infect Dis. 2015 Jun 23
Isoniazid	ahpC_-400_indel	ahpC_-400_588+586_del_GGTGGCCAGCCACACCGCCCGGGTGTTG	S	Walker TM et. al., Lancet Infect Dis. 2015 Jun 23
Isoniazid	ahpC_213_indel	ahpC_213_214_ins_A	S	Walker TM et. al., Lancet Infect Dis. 2015 Jun 23
Isoniazid	ahpC_249_indel	ahpC_249_249_del_G	S	Walker TM et. al., Lancet Infect Dis. 2015 Jun 23
Isoniazid	ahpC_A-80G		S	Walker TM et. al., Lancet Infect Dis. 2015 Jun 23
Isoniazid	ahpC_A-83G		S	Walker TM et. al., Lancet Infect Dis. 2015 Jun 23
Isoniazid	ahpC_A-98C		S	Walker TM et. al., Lancet Infect Dis. 2015 Jun 23
Isoniazid	ahpC_A122T		S	Walker TM et. al., Lancet Infect Dis. 2015 Jun 23
Isoniazid	ahpC_A187P		S	Walker TM et. al., Lancet Infect Dis. 2015 Jun 23
Isoniazid	ahpC_A195S		S	Walker TM et. al., Lancet Infect Dis. 2015 Jun 23
Isoniazid	ahpC_C-52T		S	Walker TM et. al., Lancet Infect Dis. 2015 Jun 23
Isoniazid	ahpC_C-57T		R	Walker TM et. al., Lancet Infect Dis. 2015 Jun 23
Isoniazid	ahpC_C-72T		R	Walker TM et. al., Lancet Infect Dis. 2015 Jun 23
Isoniazid	ahpC_C-79T		S	Walker TM et. al., Lancet Infect Dis. 2015 Jun 23
Isoniazid	ahpC_C-81T		S	Walker TM et. al., Lancet Infect Dis. 2015 Jun 23
Isoniazid	ahpC_D182G		S	Walker TM et. al., Lancet Infect Dis. 2015 Jun 23
Isoniazid	ahpC_E160K		S	Walker TM et. al., Lancet Infect Dis. 2015 Jun 23
Isoniazid	ahpC_G-48A		R	Walker TM et. al., Lancet Infect Dis. 2015 Jun 23
Isoniazid	ahpC_G-88A		S (Phylogenetic SN	Walker TM et. al., Lancet Infect Dis. 2015 Jun 23
Isoniazid	ahpC_G45S		S	Walker TM et. al., Lancet Infect Dis. 2015 Jun 23
Isoniazid	ahpC_K192N		S	Walker TM et. al., Lancet Infect Dis. 2015 Jun 23
Isoniazid	ahpC_L191F		S	Walker TM et. al., Lancet Infect Dis. 2015 Jun 23
Isoniazid	ahpC_P44R		S	Walker TM et. al., Lancet Infect Dis. 2015 Jun 23
Isoniazid	ahpC_P62H		S	Walker TM et. al., Lancet Infect Dis. 2015 Jun 23
Isoniazid	ahpC_T105M		S	Walker TM et. al., Lancet Infect Dis. 2015 Jun 23
Isoniazid	ahpC_V158F		S	Walker TM et. al., Lancet Infect Dis. 2015 Jun 23
Isoniazid	ahpC_Y34C		S	Walker TM et. al., Lancet Infect Dis. 2015 Jun 23
Isoniazid	ahpC: All synonymous mutations		S	Walker TM et. al., Lancet Infect Dis. 2015 Jun 23
Isoniazid	fabG1_A215T		S	Walker TM et. al., Lancet Infect Dis. 2015 Jun 23
Isoniazid	fabG1_C-15T		R	Walker TM et. al., Lancet Infect Dis. 2015 Jun 23
Isoniazid	fabG1_E7K		S	Walker TM et. al., Lancet Infect Dis. 2015 Jun 23
Isoniazid	fabG1_G-17T		R	Walker TM et. al., Lancet Infect Dis. 2015 Jun 23
Isoniazid	fabG1_G-47C		S	Walker TM et. al., Lancet Infect Dis. 2015 Jun 23
Isoniazid	fabG1_G-77A		S	Walker TM et. al., Lancet Infect Dis. 2015 Jun 23
Isoniazid	fabG1_L203L		R	Walker TM et. al., Lancet Infect Dis. 2015 Jun 23
Isoniazid	fabG1_N24S		S	Walker TM et. al., Lancet Infect Dis. 2015 Jun 23
Isoniazid	fabG1_P81A		S	Walker TM et. al., Lancet Infect Dis. 2015 Jun 23
Isoniazid	fabG1_S126N		S	Walker TM et. al., Lancet Infect Dis. 2015 Jun 23
Isoniazid	fabG1_T-8C		R	Walker TM et. al., Lancet Infect Dis. 2015 Jun 23
Isoniazid	fabG1_T4I		S	Walker TM et. al., Lancet Infect Dis. 2015 Jun 23
Isoniazid	fabG1: All synonymous mutations other than L203L		S	Walker TM et. al., Lancet Infect Dis. 2015 Jun 23
Isoniazid	inhA_A-14G		S	Walker TM et. al., Lancet Infect Dis. 2015 Jun 23
Isoniazid	inhA_C-40T		S	Walker TM et. al., Lancet Infect Dis. 2015 Jun 23
Isoniazid	inhA_C-67T		S	Walker TM et. al., Lancet Infect Dis. 2015 Jun 23
Isoniazid	inhA_G141R		S	Walker TM et. al., Lancet Infect Dis. 2015 Jun 23
Isoniazid	inhA_G183R		S	Walker TM et. al., Lancet Infect Dis. 2015 Jun 23
Isoniazid	inhA_I194T		R	Walker TM et. al., Lancet Infect Dis. 2015 Jun 23
Isoniazid	inhA_I21T		R	Walker TM et. al., Lancet Infect Dis. 2015 Jun 23
Isoniazid	inhA_I228V		S	Walker TM et. al., Lancet Infect Dis. 2015 Jun 23
Isoniazid	inhA_S94A		R	Walker TM et. al., Lancet Infect Dis. 2015 Jun 23
Isoniazid	inhA: All synonymous mutations		S	Walker TM et. al., Lancet Infect Dis. 2015 Jun 23
Isoniazid	katG_1286_indel	katG_1286_1288_del_CGC	R	Walker TM et. al., Lancet Infect Dis. 2015 Jun 23
Isoniazid	katG_1339_indel	katG_1339_1350_del_CACGACCTCGTC	R	Walker TM et. al., Lancet Infect Dis. 2015 Jun 23
Isoniazid	katG_1365_indel	katG_1365_1365_del_T	R	Walker TM et. al., Lancet Infect Dis. 2015 Jun 23
Isoniazid	katG_1804_indel	katG_1804_1810_del_AACCCGT	R	Walker TM et. al., Lancet Infect Dis. 2015 Jun 23
Isoniazid	katG_1900_indel	katG_1900_1901_ins_C	R	Walker TM et. al., Lancet Infect Dis. 2015 Jun 23
Isoniazid	katG_21_indel	katG_21_22_ins_T	R	Walker TM et. al., Lancet Infect Dis. 2015 Jun 23
Isoniazid	katG_371_indel	katG_371_371_del_G	R	Walker TM et. al., Lancet Infect Dis. 2015 Jun 23
Isoniazid	katG_A-35G		S	Walker TM et. al., Lancet Infect Dis. 2015 Jun 23
Isoniazid	katG_A109V		R	Walker TM et. al., Lancet Infect Dis. 2015 Jun 23
Isoniazid	fabG1_C-15T		R	ReSeqTB systematic review
Isoniazid	katG_A162V		S	Walker TM et. al., Lancet Infect Dis. 2015 Jun 23
Isoniazid	katG_A16V		S	Walker TM et. al., Lancet Infect Dis. 2015 Jun 23
Isoniazid	katG_A281T		S	Walker TM et. al., Lancet Infect Dis. 2015 Jun 23
Isoniazid	katG_A480S		S	Walker TM et. al., Lancet Infect Dis. 2015 Jun 23
Isoniazid	katG_A532P		S	Walker TM et. al., Lancet Infect Dis. 2015 Jun 23
Isoniazid	katG_A551S		S	Walker TM et. al., Lancet Infect Dis. 2015 Jun 23
Isoniazid	katG_A591T		S	Walker TM et. al., Lancet Infect Dis. 2015 Jun 23
Isoniazid	katG_A606T		S	Walker TM et. al., Lancet Infect Dis. 2015 Jun 23
Isoniazid	katG_A614E		R	Walker TM et. al., Lancet Infect Dis. 2015 Jun 23
Isoniazid	katG_C-79T		S	Walker TM et. al., Lancet Infect Dis. 2015 Jun 23
Isoniazid	katG_C-85T		S	Walker TM et. al., Lancet Infect Dis. 2015 Jun 23
Isoniazid	fabG1: Any nucleotide mutation at positions -16, -15 and -8 in the operator region		R	WHO endorsed line probe assays
Isoniazid	katG_D142G		R	Walker TM et. al., Lancet Infect Dis. 2015 Jun 23
Isoniazid	katG_D194N		S	Walker TM et. al., Lancet Infect Dis. 2015 Jun 23

Isoniazid	katG_D215E		S	Walker TM et. al., Lancet Infect Dis. 2015 Jun 23
Isoniazid	katG_D406G		S	Walker TM et. al., Lancet Infect Dis. 2015 Jun 23
Isoniazid	katG_D511N		S	Walker TM et. al., Lancet Infect Dis. 2015 Jun 23
Isoniazid	katG_E340D		S	Walker TM et. al., Lancet Infect Dis. 2015 Jun 23
Isoniazid	katG_E522K		S	Walker TM et. al., Lancet Infect Dis. 2015 Jun 23
Isoniazid	katG_E523D		S	Walker TM et. al., Lancet Infect Dis. 2015 Jun 23
Isoniazid	katG_G-76A		S	Walker TM et. al., Lancet Infect Dis. 2015 Jun 23
Isoniazid	katG_G-89A		S	Walker TM et. al., Lancet Infect Dis. 2015 Jun 23
Isoniazid	katG_G121S		S	Walker TM et. al., Lancet Infect Dis. 2015 Jun 23
Isoniazid	katG_G123R		S	Walker TM et. al., Lancet Infect Dis. 2015 Jun 23
Isoniazid	katG_G124A		S	Walker TM et. al., Lancet Infect Dis. 2015 Jun 23
Isoniazid	katG_G124D		S	Walker TM et. al., Lancet Infect Dis. 2015 Jun 23
Isoniazid	katG_G125D		R	Walker TM et. al., Lancet Infect Dis. 2015 Jun 23
Isoniazid	katG_G182R		R	Walker TM et. al., Lancet Infect Dis. 2015 Jun 23
Isoniazid	katG_G237A		S	Walker TM et. al., Lancet Infect Dis. 2015 Jun 23
Isoniazid	katG_G285V		S	Walker TM et. al., Lancet Infect Dis. 2015 Jun 23
Isoniazid	katG_G297V		R	Walker TM et. al., Lancet Infect Dis. 2015 Jun 23
Isoniazid	katG_G534R		S	Walker TM et. al., Lancet Infect Dis. 2015 Jun 23
Isoniazid	katG_K433T		S	Walker TM et. al., Lancet Infect Dis. 2015 Jun 23
Isoniazid	katG_K537E		S	Walker TM et. al., Lancet Infect Dis. 2015 Jun 23
Isoniazid	katG_K557N		S	Walker TM et. al., Lancet Infect Dis. 2015 Jun 23
Isoniazid	katG_L141F		R	Walker TM et. al., Lancet Infect Dis. 2015 Jun 23
Isoniazid	katG_L159P		R	Walker TM et. al., Lancet Infect Dis. 2015 Jun 23
Isoniazid	katG_L205R		S	Walker TM et. al., Lancet Infect Dis. 2015 Jun 23
Isoniazid	katG_L398R		S	Walker TM et. al., Lancet Infect Dis. 2015 Jun 23
Isoniazid	katG_L598R		S	Walker TM et. al., Lancet Infect Dis. 2015 Jun 23
Isoniazid	katG_L696Q		S	Walker TM et. al., Lancet Infect Dis. 2015 Jun 23
Isoniazid	katG_L704S		R	Walker TM et. al., Lancet Infect Dis. 2015 Jun 23
Isoniazid	katG_M257I		S	Walker TM et. al., Lancet Infect Dis. 2015 Jun 23
Isoniazid	katG_M609T		S	Walker TM et. al., Lancet Infect Dis. 2015 Jun 23
Isoniazid	katG_N323S		S	Walker TM et. al., Lancet Infect Dis. 2015 Jun 23
Isoniazid	katG_N562H		S	Walker TM et. al., Lancet Infect Dis. 2015 Jun 23
Isoniazid	katG_P232R		R	Walker TM et. al., Lancet Infect Dis. 2015 Jun 23
Isoniazid	katG_P432T		S	Walker TM et. al., Lancet Infect Dis. 2015 Jun 23
Isoniazid	katG_P510A		S	Walker TM et. al., Lancet Infect Dis. 2015 Jun 23
Isoniazid	katG_P6S		S	Walker TM et. al., Lancet Infect Dis. 2015 Jun 23
Isoniazid	katG_Q295A		S	Walker TM et. al., Lancet Infect Dis. 2015 Jun 23
Isoniazid	katG_Q36P		S	Walker TM et. al., Lancet Infect Dis. 2015 Jun 23
Isoniazid	katG_Q525K		S	Walker TM et. al., Lancet Infect Dis. 2015 Jun 23
Isoniazid	katG_Q525S		S	Walker TM et. al., Lancet Infect Dis. 2015 Jun 23
Isoniazid	katG_Q88E		S	Walker TM et. al., Lancet Infect Dis. 2015 Jun 23
Isoniazid	katG_R104Q		R	Walker TM et. al., Lancet Infect Dis. 2015 Jun 23
Isoniazid	katG_R519H		S	Walker TM et. al., Lancet Infect Dis. 2015 Jun 23
Isoniazid	katG_S446N		S	Walker TM et. al., Lancet Infect Dis. 2015 Jun 23
Isoniazid	katG_S481L		R	Walker TM et. al., Lancet Infect Dis. 2015 Jun 23
Isoniazid	katG_S527L		S	Walker TM et. al., Lancet Infect Dis. 2015 Jun 23
Isoniazid	katG_S700P		R	Walker TM et. al., Lancet Infect Dis. 2015 Jun 23
Isoniazid	katG_T-13C		S	Walker TM et. al., Lancet Infect Dis. 2015 Jun 23
Isoniazid	katG_T12A		S	Walker TM et. al., Lancet Infect Dis. 2015 Jun 23
Isoniazid	katG_T180K		R	Walker TM et. al., Lancet Infect Dis. 2015 Jun 23
Isoniazid	katG_T308A		S	Walker TM et. al., Lancet Infect Dis. 2015 Jun 23
Isoniazid	katG_T394A		S	Walker TM et. al., Lancet Infect Dis. 2015 Jun 23
Isoniazid	katG_T475I		S	Walker TM et. al., Lancet Infect Dis. 2015 Jun 23
Isoniazid	katG_T625A		S	Walker TM et. al., Lancet Infect Dis. 2015 Jun 23
Isoniazid	katG_T667I		S	Walker TM et. al., Lancet Infect Dis. 2015 Jun 23
Isoniazid	katG_T77R		S	Walker TM et. al., Lancet Infect Dis. 2015 Jun 23
Isoniazid	katG_V23L		S	Walker TM et. al., Lancet Infect Dis. 2015 Jun 23
Isoniazid	katG_V260I		S	Walker TM et. al., Lancet Infect Dis. 2015 Jun 23
Isoniazid	katG_V445I		S	Walker TM et. al., Lancet Infect Dis. 2015 Jun 23
Isoniazid	katG_V469L		S	Walker TM et. al., Lancet Infect Dis. 2015 Jun 23
Isoniazid	katG_V473L		S	Walker TM et. al., Lancet Infect Dis. 2015 Jun 23
Isoniazid	katG_V47I		S	Walker TM et. al., Lancet Infect Dis. 2015 Jun 23
Isoniazid	katG_V507I		S	Walker TM et. al., Lancet Infect Dis. 2015 Jun 23
Isoniazid	katG_V633A		R	Walker TM et. al., Lancet Infect Dis. 2015 Jun 23
Isoniazid	katG_S315I		R	ReSeqTB systematic review
Isoniazid	katG_S315N		R	ReSeqTB systematic review
Isoniazid	katG_S315T		R	ReSeqTB systematic review
Isoniazid	katG_V68G		S	Walker TM et. al., Lancet Infect Dis. 2015 Jun 23
Isoniazid	katG_V739M		S	Walker TM et. al., Lancet Infect Dis. 2015 Jun 23
Isoniazid	katG_W191G		R	Walker TM et. al., Lancet Infect Dis. 2015 Jun 23
Isoniazid	katG_W191R		R	Walker TM et. al., Lancet Infect Dis. 2015 Jun 23
Isoniazid	katG_W300C		R	Walker TM et. al., Lancet Infect Dis. 2015 Jun 23
Isoniazid	katG_W328L		R	Walker TM et. al., Lancet Infect Dis. 2015 Jun 23
Isoniazid	katG_W505Stop		R	Walker TM et. al., Lancet Infect Dis. 2015 Jun 23
Isoniazid	katG_W90R		R	Walker TM et. al., Lancet Infect Dis. 2015 Jun 23
Isoniazid	katG: All synonymous mutations		S	Walker TM et. al., Lancet Infect Dis. 2015 Jun 23
Pyrazinamide	pncA_-1526_indel	pncA_-1526_561+4428_del_GCGTTGGGGTGTCTTGACCTGTCGTCC	R	Walker TM et. al., Lancet Infect Dis. 2015 Jun 23
Pyrazinamide	pncA_-3_indel	pncA_-3_-2_ins_C	S	Walker TM et. al., Lancet Infect Dis. 2015 Jun 23
Pyrazinamide	pncA_-745_indel	pncA_-745_492_del_TGCGCTGGTCGGGTTTCGGCGCCACCCATGCC	R	Walker TM et. al., Lancet Infect Dis. 2015 Jun 23
Pyrazinamide	pncA_*187Stop		S	Walker TM et. al., Lancet Infect Dis. 2015 Jun 23
Pyrazinamide	pncA_145_indel	pncA_145_155_del_GACTTCCACAT	S	Walker TM et. al., Lancet Infect Dis. 2015 Jun 23

Pyrazinamide	pncA_185_indel	pncA_185_186_ins_A	R	Walker TM et. al., Lancet Infect Dis. 2015 Jun 23
Pyrazinamide	pncA_189_indel	pncA_189_200_del_CTATTCCTCGTC	S	Walker TM et. al., Lancet Infect Dis. 2015 Jun 23
Pyrazinamide	pncA_192_indel	pncA_192_193_ins_A	R	Walker TM et. al., Lancet Infect Dis. 2015 Jun 23
Pyrazinamide	pncA_231_indel	pncA_231_231_del_C	S	Walker TM et. al., Lancet Infect Dis. 2015 Jun 23
Pyrazinamide	pncA_306_indel	pncA_306_309_del_GTAC	R	Walker TM et. al., Lancet Infect Dis. 2015 Jun 23
Pyrazinamide	pncA_338_indel	pncA_338_346_del_GCACGCCAC	S	Walker TM et. al., Lancet Infect Dis. 2015 Jun 23
Pyrazinamide	pncA_386_indel	pncA_386_389_del_ATGT	R	Walker TM et. al., Lancet Infect Dis. 2015 Jun 23
Pyrazinamide	pncA_395_indel	pncA_395_527_del_GTATTGCCACCGATCATTGTGTGCGCCAGACG	S	Walker TM et. al., Lancet Infect Dis. 2015 Jun 23
Pyrazinamide	pncA_416_indel	pncA_416_416_del_T	S	Walker TM et. al., Lancet Infect Dis. 2015 Jun 23
Pyrazinamide	pncA_428_indel	pncA_428_429_ins_GCCAGACGGC	R	Walker TM et. al., Lancet Infect Dis. 2015 Jun 23
Pyrazinamide	pncA_456_indel	pncA_456_457_ins_C	R	Walker TM et. al., Lancet Infect Dis. 2015 Jun 23
Pyrazinamide	pncA_463_indel	pncA_463_464_ins_G	R	Walker TM et. al., Lancet Infect Dis. 2015 Jun 23
Pyrazinamide	pncA_47_indel	pncA_47_48_ins_G	S	Walker TM et. al., Lancet Infect Dis. 2015 Jun 23
Pyrazinamide	pncA_470_indel	pncA_470_471_ins_A	R	Walker TM et. al., Lancet Infect Dis. 2015 Jun 23
Pyrazinamide	pncA_48_indel	pncA_48_74_del_TGGCTCGCTGGCGGTAACCGGTGGCGC	R	Walker TM et. al., Lancet Infect Dis. 2015 Jun 23
Pyrazinamide	pncA_517_indel	pncA_517_518_ins_G	R	Walker TM et. al., Lancet Infect Dis. 2015 Jun 23
Isoniazid	katG: Any amino acid substitution at codon 315		R	WHO endorsed line probe assays
Isoniazid	katG: Any frame shift insertion or deletion		R	ReSeqTB systematic review
Pyrazinamide	pncA_553_indel	pncA_553_561+3_del_AGCTCCTGATGG	R	Walker TM et. al., Lancet Infect Dis. 2015 Jun 23
Pyrazinamide	pncA_A102P		S	Walker TM et. al., Lancet Infect Dis. 2015 Jun 23
Pyrazinamide	pncA_A134D		S	Walker TM et. al., Lancet Infect Dis. 2015 Jun 23
Pyrazinamide	pncA_A143V		S	Walker TM et. al., Lancet Infect Dis. 2015 Jun 23
Pyrazinamide	pncA_A146T		S	Walker TM et. al., Lancet Infect Dis. 2015 Jun 23
Pyrazinamide	pncA_A79T		S	Walker TM et. al., Lancet Infect Dis. 2015 Jun 23
Pyrazinamide	pncA_C-19T		S	Walker TM et. al., Lancet Infect Dis. 2015 Jun 23
Pyrazinamide	pncA_C138R		R	Walker TM et. al., Lancet Infect Dis. 2015 Jun 23
Pyrazinamide	pncA_D129N		S	Walker TM et. al., Lancet Infect Dis. 2015 Jun 23
Pyrazinamide	pncA_D136N		R	Walker TM et. al., Lancet Infect Dis. 2015 Jun 23
Pyrazinamide	pncA_D53E		S	Walker TM et. al., Lancet Infect Dis. 2015 Jun 23
Pyrazinamide	pncA_D63A		S	Walker TM et. al., Lancet Infect Dis. 2015 Jun 23
Pyrazinamide	pncA_E37V		S	Walker TM et. al., Lancet Infect Dis. 2015 Jun 23
Pyrazinamide	pncA_F81V		R	Walker TM et. al., Lancet Infect Dis. 2015 Jun 23
Pyrazinamide	pncA_G-33A		S	Walker TM et. al., Lancet Infect Dis. 2015 Jun 23
Pyrazinamide	pncA_G17S		S	Walker TM et. al., Lancet Infect Dis. 2015 Jun 23
Pyrazinamide	pncA_G78C		R	Walker TM et. al., Lancet Infect Dis. 2015 Jun 23
Pyrazinamide	pncA_G97R		R	Walker TM et. al., Lancet Infect Dis. 2015 Jun 23
Pyrazinamide	pncA_H137R		R	Walker TM et. al., Lancet Infect Dis. 2015 Jun 23
Pyrazinamide	pncA_H82D		S	Walker TM et. al., Lancet Infect Dis. 2015 Jun 23
Pyrazinamide	pncA_K48E		R	Walker TM et. al., Lancet Infect Dis. 2015 Jun 23
Pyrazinamide	pncA_L159V		R	Walker TM et. al., Lancet Infect Dis. 2015 Jun 23
Pyrazinamide	pncA_A-11G		R	ReSeqTB systematic review
Pyrazinamide	pncA_L182S		S	Walker TM et. al., Lancet Infect Dis. 2015 Jun 23
Pyrazinamide	pncA_L27P		R	Walker TM et. al., Lancet Infect Dis. 2015 Jun 23
Pyrazinamide	pncA_A134V		R	ReSeqTB systematic review
Pyrazinamide	pncA_L35R		S	Walker TM et. al., Lancet Infect Dis. 2015 Jun 23
Pyrazinamide	pncA_M1I		S	Walker TM et. al., Lancet Infect Dis. 2015 Jun 23
Pyrazinamide	pncA_A171E		R	ReSeqTB systematic review
Pyrazinamide	pncA_A3E		R	ReSeqTB systematic review
Pyrazinamide	pncA_A46V		R	ReSeqTB systematic review
Pyrazinamide	pncA_P54Q		R	Walker TM et. al., Lancet Infect Dis. 2015 Jun 23
Pyrazinamide	pncA_P69S		S	Walker TM et. al., Lancet Infect Dis. 2015 Jun 23
Pyrazinamide	pncA_P77L		S	Walker TM et. al., Lancet Infect Dis. 2015 Jun 23
Pyrazinamide	pncA_C138Y		R	ReSeqTB systematic review
Pyrazinamide	pncA_C14R		R	ReSeqTB systematic review
Pyrazinamide	pncA_C72R		R	ReSeqTB systematic review
Pyrazinamide	pncA_Q10Stop		R	Walker TM et. al., Lancet Infect Dis. 2015 Jun 23
Pyrazinamide	pncA_D12A		R	ReSeqTB systematic review
Pyrazinamide	pncA_D12G		R	ReSeqTB systematic review
Pyrazinamide	pncA_D12N		R	ReSeqTB systematic review
Pyrazinamide	pncA_Q141Stop		R	Walker TM et. al., Lancet Infect Dis. 2015 Jun 23
Pyrazinamide	pncA_D49G		R	ReSeqTB systematic review
Pyrazinamide	pncA_D49N		R	ReSeqTB systematic review
Pyrazinamide	pncA_R121Q		S	Walker TM et. al., Lancet Infect Dis. 2015 Jun 23
Pyrazinamide	pncA_S104G		R	Walker TM et. al., Lancet Infect Dis. 2015 Jun 23
Pyrazinamide	pncA_D63G		R	ReSeqTB systematic review
Pyrazinamide	pncA_D8E		R	ReSeqTB systematic review
Pyrazinamide	pncA_D8G		R	ReSeqTB systematic review
Pyrazinamide	pncA_D8N		R	ReSeqTB systematic review
Pyrazinamide	pncA_S18P		S	Walker TM et. al., Lancet Infect Dis. 2015 Jun 23
Pyrazinamide	pncA_F58L		R	ReSeqTB systematic review
Pyrazinamide	pncA_S32I		R	Walker TM et. al., Lancet Infect Dis. 2015 Jun 23
Pyrazinamide	pncA_F94L		R	ReSeqTB systematic review
Pyrazinamide	pncA_F94S		R	ReSeqTB systematic review
Pyrazinamide	pncA_S65P		S	Walker TM et. al., Lancet Infect Dis. 2015 Jun 23
Pyrazinamide	pncA_G108R		R	ReSeqTB systematic review
Pyrazinamide	pncA_G132A		R	ReSeqTB systematic review
Pyrazinamide	pncA_G132D		R	ReSeqTB systematic review
Pyrazinamide	pncA_G132S		R	ReSeqTB systematic review
Pyrazinamide	pncA_G162D		R	ReSeqTB systematic review
Pyrazinamide	pncA_G17D		R	ReSeqTB systematic review
Pyrazinamide	pncA_S66L		S	Walker TM et. al., Lancet Infect Dis. 2015 Jun 23

Pyrazinamide	pncA_G24D		R	ReSeqTB systematic review
Pyrazinamide	pncA_T-60G		S	Walker TM et. al., Lancet Infect Dis. 2015 Jun 23
Pyrazinamide	pncA_G97C		R	ReSeqTB systematic review
Pyrazinamide	pncA_G97D		R	ReSeqTB systematic review
Pyrazinamide	pncA_T114M		S	Walker TM et. al., Lancet Infect Dis. 2015 Jun 23
Pyrazinamide	pncA_G97S		R	ReSeqTB systematic review
Pyrazinamide	pncA_H137P		R	ReSeqTB systematic review
Pyrazinamide	pncA_T114P		R	Walker TM et. al., Lancet Infect Dis. 2015 Jun 23
Pyrazinamide	pncA_H51Q		R	ReSeqTB systematic review
Pyrazinamide	pncA_H51R		R	ReSeqTB systematic review
Pyrazinamide	pncA_H57D		R	ReSeqTB systematic review
Pyrazinamide	pncA_H57P		R	ReSeqTB systematic review
Pyrazinamide	pncA_H57R		R	ReSeqTB systematic review
Pyrazinamide	pncA_H57Y		R	ReSeqTB systematic review
Pyrazinamide	pncA_H71D		R	ReSeqTB systematic review
Pyrazinamide	pncA_H71Q		R	ReSeqTB systematic review
Pyrazinamide	pncA_H71R		R	ReSeqTB systematic review
Pyrazinamide	pncA_H71Y		R	ReSeqTB systematic review
Pyrazinamide	pncA_T168I		S	Walker TM et. al., Lancet Infect Dis. 2015 Jun 23
Pyrazinamide	pncA_H82R		R	ReSeqTB systematic review
Pyrazinamide	pncA_I133T		R	ReSeqTB systematic review
Pyrazinamide	pncA_I6T		R	ReSeqTB systematic review
Pyrazinamide	pncA_T168S		S	Walker TM et. al., Lancet Infect Dis. 2015 Jun 23
Pyrazinamide	pncA_K96E		R	ReSeqTB systematic review
Pyrazinamide	pncA_K96N		R	ReSeqTB systematic review
Pyrazinamide	pncA_K96R		R	ReSeqTB systematic review
Pyrazinamide	pncA_K96T		R	ReSeqTB systematic review
Pyrazinamide	pncA_L116P		R	ReSeqTB systematic review
Pyrazinamide	pncA_L116R		R	ReSeqTB systematic review
Pyrazinamide	pncA_L120P		R	ReSeqTB systematic review
Pyrazinamide	pncA_L151S		R	ReSeqTB systematic review
Pyrazinamide	pncA_L159P		R	ReSeqTB systematic review
Pyrazinamide	pncA_T47A		R	Walker TM et. al., Lancet Infect Dis. 2015 Jun 23
Pyrazinamide	pncA_L172P		R	ReSeqTB systematic review
Pyrazinamide	pncA_T47I		S	Walker TM et. al., Lancet Infect Dis. 2015 Jun 23
Pyrazinamide	pncA_L19P		R	ReSeqTB systematic review
Pyrazinamide	pncA_T87M		S	Walker TM et. al., Lancet Infect Dis. 2015 Jun 23
Pyrazinamide	pncA_V163G		S	Walker TM et. al., Lancet Infect Dis. 2015 Jun 23
Pyrazinamide	pncA_L4S		R	ReSeqTB systematic review
Pyrazinamide	pncA_L85P		R	ReSeqTB systematic review
Pyrazinamide	pncA_L85R		R	ReSeqTB systematic review
Pyrazinamide	pncA_M175I		R	ReSeqTB systematic review
Pyrazinamide	pncA_M175T		R	ReSeqTB systematic review
Pyrazinamide	pncA_M175V		R	ReSeqTB systematic review
Pyrazinamide	pncA_V183L		S	Walker TM et. al., Lancet Infect Dis. 2015 Jun 23
Pyrazinamide	pncA_P54L		R	ReSeqTB systematic review
Pyrazinamide	pncA_V21G		R	Walker TM et. al., Lancet Infect Dis. 2015 Jun 23
Pyrazinamide	pncA_P54S		R	ReSeqTB systematic review
Pyrazinamide	pncA_P62L		R	ReSeqTB systematic review
Pyrazinamide	pncA_P62Q		R	ReSeqTB systematic review
Pyrazinamide	pncA_V45A		S	Walker TM et. al., Lancet Infect Dis. 2015 Jun 23
Pyrazinamide	pncA_V7L		R	Walker TM et. al., Lancet Infect Dis. 2015 Jun 23
Pyrazinamide	pncA_Q10P		R	ReSeqTB systematic review
Pyrazinamide	pncA_Q10R		R	ReSeqTB systematic review
Pyrazinamide	pncA_Y64D		S	Walker TM et. al., Lancet Infect Dis. 2015 Jun 23
Pyrazinamide	pncA_Q141P		R	ReSeqTB systematic review
Pyrazinamide	pncA_Y99Stop		R	Walker TM et. al., Lancet Infect Dis. 2015 Jun 23
Rifampicin	rpoB_1278_indel	rpoB_1278_1286_del_CACCAGCCA	R	Walker TM et. al., Lancet Infect Dis. 2015 Jun 23
Pyrazinamide	pncA_R123P		R	ReSeqTB systematic review
Rifampicin	rpoB_1292_indel	rpoB_1292_1293_ins_CCA	R	Walker TM et. al., Lancet Infect Dis. 2015 Jun 23
Pyrazinamide	pncA_S104R		R	ReSeqTB systematic review
Rifampicin	rpoB_1295_indel	rpoB_1295_1303_del_AATTCATGG	R	Walker TM et. al., Lancet Infect Dis. 2015 Jun 23
Rifampicin	rpoB_1296_indel	rpoB_1296_1297_ins_TTC	R	Walker TM et. al., Lancet Infect Dis. 2015 Jun 23
Pyrazinamide	pncA_S59P		R	ReSeqTB systematic review
Rifampicin	rpoB_1299_indel	rpoB_1299_1304_del_CATGGA	R	Walker TM et. al., Lancet Infect Dis. 2015 Jun 23
Rifampicin	rpoB_1328_indel	rpoB_1328_1337_delTGACCCACAAinsGGCCCCA	R	Walker TM et. al., Lancet Infect Dis. 2015 Jun 23
Pyrazinamide	pncA_S66P		R	ReSeqTB systematic review
Pyrazinamide	pncA_S67P		R	ReSeqTB systematic review
Pyrazinamide	pncA_T-12C		R	ReSeqTB systematic review
Rifampicin	rpoB_2546_indel	rpoB_2546_2547_ins_CGAGGA	S	Walker TM et. al., Lancet Infect Dis. 2015 Jun 23
Pyrazinamide	pncA_T-7C		R	ReSeqTB systematic review
Rifampicin	rpoB_A-53G		S	Walker TM et. al., Lancet Infect Dis. 2015 Jun 23
Rifampicin	rpoB_A334D		S	Walker TM et. al., Lancet Infect Dis. 2015 Jun 23
Pyrazinamide	pncA_T135N		R	ReSeqTB systematic review
Pyrazinamide	pncA_T135P		R	ReSeqTB systematic review
Pyrazinamide	pncA_T142A		R	ReSeqTB systematic review
Pyrazinamide	pncA_T142K		R	ReSeqTB systematic review
Pyrazinamide	pncA_T142M		R	ReSeqTB systematic review
Pyrazinamide	pncA_T160P		R	ReSeqTB systematic review
Rifampicin	rpoB_A544V		S	Walker TM et. al., Lancet Infect Dis. 2015 Jun 23
Pyrazinamide	pncA_T168P		R	ReSeqTB systematic review

Rifampicin	rpoB_A69P	S	Walker TM et. al., Lancet Infect Dis. 2015 Jun 23
Rifampicin	rpoB_A857T	S	Walker TM et. al., Lancet Infect Dis. 2015 Jun 23
Rifampicin	rpoB_A998V	S	Walker TM et. al., Lancet Infect Dis. 2015 Jun 23
Pyrazinamide	pncA_T76P	R	ReSeqTB systematic review
Rifampicin	rpoB_C-62T	S	Walker TM et. al., Lancet Infect Dis. 2015 Jun 23
Pyrazinamide	pncA_V125F	R	ReSeqTB systematic review
Pyrazinamide	pncA_V125G	R	ReSeqTB systematic review
Pyrazinamide	pncA_V128G	R	ReSeqTB systematic review
Pyrazinamide	pncA_V139A	R	ReSeqTB systematic review
Pyrazinamide	pncA_V139G	R	ReSeqTB systematic review
Pyrazinamide	pncA_V139L	R	ReSeqTB systematic review
Pyrazinamide	pncA_V155G	R	ReSeqTB systematic review
Rifampicin	rpoB_C-73T	S	Walker TM et. al., Lancet Infect Dis. 2015 Jun 23
Pyrazinamide	pncA_V180F	R	ReSeqTB systematic review
Pyrazinamide	pncA_V180G	R	ReSeqTB systematic review
Rifampicin	rpoB_D270E	S	Walker TM et. al., Lancet Infect Dis. 2015 Jun 23
Rifampicin	rpoB_D362H	S	Walker TM et. al., Lancet Infect Dis. 2015 Jun 23
Rifampicin	rpoB_D3G	S	Walker TM et. al., Lancet Infect Dis. 2015 Jun 23
Pyrazinamide	pncA_V7G	R	ReSeqTB systematic review
Rifampicin	rpoB_D515Y	S	Walker TM et. al., Lancet Infect Dis. 2015 Jun 23
Pyrazinamide	pncA_W68C	R	ReSeqTB systematic review
Pyrazinamide	pncA_W68G	R	ReSeqTB systematic review
Pyrazinamide	pncA_W68R	R	ReSeqTB systematic review
Pyrazinamide	pncA_Y103H	R	ReSeqTB systematic review
Pyrazinamide	pncA_Y34D	R	ReSeqTB systematic review
Rifampicin	rpoB_D53N	S	Walker TM et. al., Lancet Infect Dis. 2015 Jun 23
Rifampicin	rpoB_D545A	S	Walker TM et. al., Lancet Infect Dis. 2015 Jun 23
Rifampicin	rpoB_D634G	S	Walker TM et. al., Lancet Infect Dis. 2015 Jun 23
Pyrazinamide	pncA: Any frame shift insertion or deletion	R	ReSeqTB systematic review
Rifampicin	rpoB_D851G	S	Walker TM et. al., Lancet Infect Dis. 2015 Jun 23
Rifampicin	rpoB_E1169A	S	Walker TM et. al., Lancet Infect Dis. 2015 Jun 23
Rifampicin	rpoB_E132D	S	Walker TM et. al., Lancet Infect Dis. 2015 Jun 23
Rifampicin	rpoB_E563D	S	Walker TM et. al., Lancet Infect Dis. 2015 Jun 23
Rifampicin	rpoB_E639G	S	Walker TM et. al., Lancet Infect Dis. 2015 Jun 23
Rifampicin	rpoB_E639Q	S	Walker TM et. al., Lancet Infect Dis. 2015 Jun 23
Rifampicin	rpoB_E66K	S	Walker TM et. al., Lancet Infect Dis. 2015 Jun 23
Rifampicin	rpoB_E825G	S	Walker TM et. al., Lancet Infect Dis. 2015 Jun 23
Rifampicin	rpoB_G-96A	S	Walker TM et. al., Lancet Infect Dis. 2015 Jun 23
Rifampicin	rpoB_G28R	S	Walker TM et. al., Lancet Infect Dis. 2015 Jun 23
Rifampicin	rpoB_G890D	S	Walker TM et. al., Lancet Infect Dis. 2015 Jun 23
Rifampicin	rpoB_G981D	R	Walker TM et. al., Lancet Infect Dis. 2015 Jun 23
Rifampicin	rpoB_H343Q	S	Walker TM et. al., Lancet Infect Dis. 2015 Jun 23
Rifampicin	rpoB_H674Q	S	Walker TM et. al., Lancet Infect Dis. 2015 Jun 23
Rifampicin	rpoB_K944E	S	Walker TM et. al., Lancet Infect Dis. 2015 Jun 23
Rifampicin	rpoB_L314V	S	Walker TM et. al., Lancet Infect Dis. 2015 Jun 23
Rifampicin	rpoB_L316V	S	Walker TM et. al., Lancet Infect Dis. 2015 Jun 23
Rifampicin	rpoB_L443F	S	Walker TM et. al., Lancet Infect Dis. 2015 Jun 23
Rifampicin	rpoB_L80V	S	Walker TM et. al., Lancet Infect Dis. 2015 Jun 23
Rifampicin	rpoB_L893R	S	Walker TM et. al., Lancet Infect Dis. 2015 Jun 23
Rifampicin	rpoB_M121I	S	Walker TM et. al., Lancet Infect Dis. 2015 Jun 23
Rifampicin	rpoB_M153T	S	Walker TM et. al., Lancet Infect Dis. 2015 Jun 23
Rifampicin	rpoB_M390T	S	Walker TM et. al., Lancet Infect Dis. 2015 Jun 23
Rifampicin	rpoB_M434I	R	Walker TM et. al., Lancet Infect Dis. 2015 Jun 23
Rifampicin	rpoB_N24D	S	Walker TM et. al., Lancet Infect Dis. 2015 Jun 23
Rifampicin	rpoB_P30S	S	Walker TM et. al., Lancet Infect Dis. 2015 Jun 23
Rifampicin	rpoB_P358L	S	Walker TM et. al., Lancet Infect Dis. 2015 Jun 23
Rifampicin	rpoB_P454L	S	Walker TM et. al., Lancet Infect Dis. 2015 Jun 23
Rifampicin	rpoB_P682T	S	Walker TM et. al., Lancet Infect Dis. 2015 Jun 23
Rifampicin	rpoB_P834L	S	Walker TM et. al., Lancet Infect Dis. 2015 Jun 23
Rifampicin	rpoB_P89L	S	Walker TM et. al., Lancet Infect Dis. 2015 Jun 23
Rifampicin	rpoB_R511L	S	Walker TM et. al., Lancet Infect Dis. 2015 Jun 23
Rifampicin	rpoB_R661Q	S	Walker TM et. al., Lancet Infect Dis. 2015 Jun 23
Rifampicin	rpoB_S1124A	S	Walker TM et. al., Lancet Infect Dis. 2015 Jun 23
Rifampicin	rpoB_S195R	S	Walker TM et. al., Lancet Infect Dis. 2015 Jun 23
Rifampicin	rpoB_S21F	S	Walker TM et. al., Lancet Infect Dis. 2015 Jun 23
Rifampicin	rpoB_S388L	S	Walker TM et. al., Lancet Infect Dis. 2015 Jun 23
Rifampicin	rpoB_T-6C	S	Walker TM et. al., Lancet Infect Dis. 2015 Jun 23
Rifampicin	rpoB_T350I	S	Walker TM et. al., Lancet Infect Dis. 2015 Jun 23
Rifampicin	rpoB_T526S	S	Walker TM et. al., Lancet Infect Dis. 2015 Jun 23
Rifampicin	rpoB_T676P	R	Walker TM et. al., Lancet Infect Dis. 2015 Jun 23
Rifampicin	rpoB_V109I	S	Walker TM et. al., Lancet Infect Dis. 2015 Jun 23
Rifampicin	rpoB_V1117L	S	Walker TM et. al., Lancet Infect Dis. 2015 Jun 23
Rifampicin	rpoB_V1129A	S	Walker TM et. al., Lancet Infect Dis. 2015 Jun 23
Rifampicin	rpoB_V113I	S	Walker TM et. al., Lancet Infect Dis. 2015 Jun 23
Rifampicin	rpoB_V170F	R	Walker TM et. al., Lancet Infect Dis. 2015 Jun 23
Rifampicin	rpoB_V179A	S	Walker TM et. al., Lancet Infect Dis. 2015 Jun 23
Rifampicin	rpoB_V359A	R	Walker TM et. al., Lancet Infect Dis. 2015 Jun 23
Rifampicin	rpoB_V695L	S	Walker TM et. al., Lancet Infect Dis. 2015 Jun 23
Rifampicin	rpoB_V77M	S	Walker TM et. al., Lancet Infect Dis. 2015 Jun 23
Ethambutol	embA_M153T	S (Phylogenetic SN	Walker TM et. al., Lancet Infect Dis. 2015 Jun 23
Rifampicin	rpoB: All synonymous mutations	S	Walker TM et. al., Lancet Infect Dis. 2015 Jun 23

Ethambutol	embA_P913S	S (Phylogenetic SN	Walker TM et. al., Lancet Infect Dis. 2015 Jun 23
Ethambutol	embB: All synonymous mutations	S	Walker TM et. al., Lancet Infect Dis. 2015 Jun 23
Ethambutol	embA_S49R	S (Phylogenetic SN	Walker TM et. al., Lancet Infect Dis. 2015 Jun 23
Ethambutol	embA: All synonymous mutations	S	Walker TM et. al., Lancet Infect Dis. 2015 Jun 23
Ethambutol	embA_T608N	S (Phylogenetic SN	Walker TM et. al., Lancet Infect Dis. 2015 Jun 23
Ethambutol	embC: All synonymous mutations	S	Walker TM et. al., Lancet Infect Dis. 2015 Jun 23
Ethambutol	embA_V206M	S (Phylogenetic SN	Walker TM et. al., Lancet Infect Dis. 2015 Jun 23
Pyrazinamide	pncA: All synonymous mutations	S	Walker TM et. al., Lancet Infect Dis. 2015 Jun 23
Ethambutol	embB_E378A	S (Phylogenetic SN	Walker TM et. al., Lancet Infect Dis. 2015 Jun 23
Ethambutol	embB_K561R	S (Phylogenetic SN	Walker TM et. al., Lancet Infect Dis. 2015 Jun 23
Ethambutol	embB_Q139H	S (Phylogenetic SN	Walker TM et. al., Lancet Infect Dis. 2015 Jun 23
Ethambutol	embB_S565G	S (Phylogenetic SN	Walker TM et. al., Lancet Infect Dis. 2015 Jun 23
Ethambutol	embC_N394D	S (Phylogenetic SN	Walker TM et. al., Lancet Infect Dis. 2015 Jun 23
Ethambutol	embC_R567H	S (Phylogenetic SN	Walker TM et. al., Lancet Infect Dis. 2015 Jun 23
Ethambutol	embC_R738Q	S (Phylogenetic SN	Walker TM et. al., Lancet Infect Dis. 2015 Jun 23
Ethambutol	embC_T270I	S (Phylogenetic SN	Walker TM et. al., Lancet Infect Dis. 2015 Jun 23
Ethambutol	embC_V104M	S (Phylogenetic SN	Walker TM et. al., Lancet Infect Dis. 2015 Jun 23
Ethambutol	embC_V981L	S (Phylogenetic SN	Walker TM et. al., Lancet Infect Dis. 2015 Jun 23
Isoniazid	inhA_P107S	S (Phylogenetic SN	Walker TM et. al., Lancet Infect Dis. 2015 Jun 23
Isoniazid	inhA_V78A	S (Phylogenetic SN	Walker TM et. al., Lancet Infect Dis. 2015 Jun 23
Isoniazid	katG_R463L	S (Phylogenetic SN	Walker TM et. al., Lancet Infect Dis. 2015 Jun 23
Rifampicin	rpoB_C-61T	S (Phylogenetic SN	Walker TM et. al., Lancet Infect Dis. 2015 Jun 23
Rifampicin	rpoB: Any amino acid substitution or insertion/deletion from codon 425 to codon 45	R	WHO endorsed line probe assays / Xpert MTB/RIF
Pyrazinamide	pncA_E37Stop	R	Yadon et. al., Nature Communications 2017 Sep 19
Pyrazinamide	pncA_E91Stop	R	Yadon et. al., Nature Communications 2017 Sep 19
Pyrazinamide	pncA_F106S	R	Yadon et. al., Nature Communications 2017 Sep 19
Pyrazinamide	pncA_F106Y	R	Yadon et. al., Nature Communications 2017 Sep 19
Pyrazinamide	pncA_F13I	R	Yadon et. al., Nature Communications 2017 Sep 19
Pyrazinamide	pncA_F13V	R	Yadon et. al., Nature Communications 2017 Sep 19
Pyrazinamide	pncA_F13Y	R	Yadon et. al., Nature Communications 2017 Sep 19
Pyrazinamide	pncA_F50F	R	Yadon et. al., Nature Communications 2017 Sep 19
Pyrazinamide	pncA_F58I	R	Yadon et. al., Nature Communications 2017 Sep 19
Pyrazinamide	pncA_F58S	R	Yadon et. al., Nature Communications 2017 Sep 19
Pyrazinamide	pncA_F81S	R	Yadon et. al., Nature Communications 2017 Sep 19
Pyrazinamide	pncA_F94C	R	Yadon et. al., Nature Communications 2017 Sep 19
Pyrazinamide	pncA_F94F	R	Yadon et. al., Nature Communications 2017 Sep 19
Pyrazinamide	pncA_G101Stop	R	Yadon et. al., Nature Communications 2017 Sep 19
Pyrazinamide	pncA_G101E	R	Yadon et. al., Nature Communications 2017 Sep 19
Pyrazinamide	pncA_G105D	R	Yadon et. al., Nature Communications 2017 Sep 19
Pyrazinamide	pncA_G105R	R	Yadon et. al., Nature Communications 2017 Sep 19
Pyrazinamide	pncA_G105V	R	Yadon et. al., Nature Communications 2017 Sep 19
Pyrazinamide	pncA_G108Stop	R	Yadon et. al., Nature Communications 2017 Sep 19
Pyrazinamide	pncA_G108E	R	Yadon et. al., Nature Communications 2017 Sep 19
Pyrazinamide	pncA_G124S	R	Yadon et. al., Nature Communications 2017 Sep 19
Pyrazinamide	pncA_G132C	R	Yadon et. al., Nature Communications 2017 Sep 19
Pyrazinamide	pncA_G132R	R	Yadon et. al., Nature Communications 2017 Sep 19
Pyrazinamide	pncA_G132V	R	Yadon et. al., Nature Communications 2017 Sep 19
Pyrazinamide	pncA_G150G	R	Yadon et. al., Nature Communications 2017 Sep 19
Pyrazinamide	pncA_G162A	R	Yadon et. al., Nature Communications 2017 Sep 19
Pyrazinamide	pncA_G162V	R	Yadon et. al., Nature Communications 2017 Sep 19
Pyrazinamide	pncA_G16G	R	Yadon et. al., Nature Communications 2017 Sep 19
Pyrazinamide	pncA_G16R	R	Yadon et. al., Nature Communications 2017 Sep 19
Pyrazinamide	pncA_G16V	R	Yadon et. al., Nature Communications 2017 Sep 19
Pyrazinamide	pncA_G17C	R	Yadon et. al., Nature Communications 2017 Sep 19
Pyrazinamide	pncA_G23V	R	Yadon et. al., Nature Communications 2017 Sep 19
Pyrazinamide	pncA_G24V	R	Yadon et. al., Nature Communications 2017 Sep 19
Pyrazinamide	pncA_G55C	R	Yadon et. al., Nature Communications 2017 Sep 19
Pyrazinamide	pncA_G75V	R	Yadon et. al., Nature Communications 2017 Sep 19
Pyrazinamide	pncA_G78D	R	Yadon et. al., Nature Communications 2017 Sep 19
Pyrazinamide	pncA_G78V	R	Yadon et. al., Nature Communications 2017 Sep 19
Pyrazinamide	pncA_H137D	R	Yadon et. al., Nature Communications 2017 Sep 19
Pyrazinamide	pncA_H42N	R	Yadon et. al., Nature Communications 2017 Sep 19
Pyrazinamide	pncA_H42Q	R	Yadon et. al., Nature Communications 2017 Sep 19
Pyrazinamide	pncA_H43P	R	Yadon et. al., Nature Communications 2017 Sep 19
Pyrazinamide	pncA_H43Q	R	Yadon et. al., Nature Communications 2017 Sep 19
Pyrazinamide	pncA_H51D	R	Yadon et. al., Nature Communications 2017 Sep 19
Pyrazinamide	pncA_H51L	R	Yadon et. al., Nature Communications 2017 Sep 19
Pyrazinamide	pncA_H51N	R	Yadon et. al., Nature Communications 2017 Sep 19
Pyrazinamide	pncA_H51Y	R	Yadon et. al., Nature Communications 2017 Sep 19
Pyrazinamide	pncA_H57L	R	Yadon et. al., Nature Communications 2017 Sep 19
Pyrazinamide	pncA_H57N	R	Yadon et. al., Nature Communications 2017 Sep 19
Pyrazinamide	pncA_H57Q	R	Yadon et. al., Nature Communications 2017 Sep 19
Pyrazinamide	pncA_H71H	R	Yadon et. al., Nature Communications 2017 Sep 19
Pyrazinamide	pncA_H71L	R	Yadon et. al., Nature Communications 2017 Sep 19
Pyrazinamide	pncA_H71N	R	Yadon et. al., Nature Communications 2017 Sep 19
Pyrazinamide	pncA_H82L	R	Yadon et. al., Nature Communications 2017 Sep 19
Pyrazinamide	pncA_I133F	R	Yadon et. al., Nature Communications 2017 Sep 19
Pyrazinamide	pncA_I133N	R	Yadon et. al., Nature Communications 2017 Sep 19
Pyrazinamide	pncA_I31F	R	Yadon et. al., Nature Communications 2017 Sep 19
Pyrazinamide	pncA_I31N	R	Yadon et. al., Nature Communications 2017 Sep 19
Pyrazinamide	pncA_I31T	R	Yadon et. al., Nature Communications 2017 Sep 19

Pyrazinamide	pncA_I52T	R	Yadon et. al., Nature Communications 2017 Sep 19
Pyrazinamide	pncA_I5F	R	Yadon et. al., Nature Communications 2017 Sep 19
Pyrazinamide	pncA_I5M	R	Yadon et. al., Nature Communications 2017 Sep 19
Pyrazinamide	pncA_I5N	R	Yadon et. al., Nature Communications 2017 Sep 19
Pyrazinamide	pncA_I5T	R	Yadon et. al., Nature Communications 2017 Sep 19
Pyrazinamide	pncA_I6F	R	Yadon et. al., Nature Communications 2017 Sep 19
Pyrazinamide	pncA_I6M	R	Yadon et. al., Nature Communications 2017 Sep 19
Pyrazinamide	pncA_K48Stop	R	Yadon et. al., Nature Communications 2017 Sep 19
Pyrazinamide	pncA_K48T	R	Yadon et. al., Nature Communications 2017 Sep 19
Pyrazinamide	pncA_K96Stop	R	Yadon et. al., Nature Communications 2017 Sep 19
Pyrazinamide	pncA_K96M	R	Yadon et. al., Nature Communications 2017 Sep 19
Pyrazinamide	pncA_K96Q	R	Yadon et. al., Nature Communications 2017 Sep 19
Pyrazinamide	pncA_L116Q	R	Yadon et. al., Nature Communications 2017 Sep 19
Pyrazinamide	pncA_L117Q	R	Yadon et. al., Nature Communications 2017 Sep 19
Pyrazinamide	pncA_L120Q	R	Yadon et. al., Nature Communications 2017 Sep 19
Pyrazinamide	pncA_L151Stop	R	Yadon et. al., Nature Communications 2017 Sep 19
Pyrazinamide	pncA_L156P	R	Yadon et. al., Nature Communications 2017 Sep 19
Pyrazinamide	pncA_L156R	R	Yadon et. al., Nature Communications 2017 Sep 19
Pyrazinamide	pncA_L159Q	R	Yadon et. al., Nature Communications 2017 Sep 19
Pyrazinamide	pncA_L19L	R	Yadon et. al., Nature Communications 2017 Sep 19
Pyrazinamide	pncA_L19Q	R	Yadon et. al., Nature Communications 2017 Sep 19
Pyrazinamide	pncA_L27Q	R	Yadon et. al., Nature Communications 2017 Sep 19
Pyrazinamide	pncA_L35P	R	Yadon et. al., Nature Communications 2017 Sep 19
Pyrazinamide	pncA_L4Stop	R	Yadon et. al., Nature Communications 2017 Sep 19
Pyrazinamide	pncA_L4F	R	Yadon et. al., Nature Communications 2017 Sep 19
Pyrazinamide	pncA_L4V	R	Yadon et. al., Nature Communications 2017 Sep 19
Pyrazinamide	pncA_L4W	R	Yadon et. al., Nature Communications 2017 Sep 19
Pyrazinamide	pncA_L85Q	R	Yadon et. al., Nature Communications 2017 Sep 19
Pyrazinamide	pncA_M175K	R	Yadon et. al., Nature Communications 2017 Sep 19
Pyrazinamide	pncA_M1L	R	Yadon et. al., Nature Communications 2017 Sep 19
Pyrazinamide	pncA_N112S	R	Yadon et. al., Nature Communications 2017 Sep 19
Pyrazinamide	pncA_N118N	R	Yadon et. al., Nature Communications 2017 Sep 19
Pyrazinamide	pncA_A134P	R	Yadon et. al., Nature Communications 2017 Sep 19
Pyrazinamide	pncA_N118Y	R	Yadon et. al., Nature Communications 2017 Sep 19
Pyrazinamide	pncA_N11T	R	Yadon et. al., Nature Communications 2017 Sep 19
Pyrazinamide	pncA_N149I	R	Yadon et. al., Nature Communications 2017 Sep 19
Pyrazinamide	pncA_N149Y	R	Yadon et. al., Nature Communications 2017 Sep 19
Pyrazinamide	pncA_P115A	R	Yadon et. al., Nature Communications 2017 Sep 19
Pyrazinamide	pncA_P115R	R	Yadon et. al., Nature Communications 2017 Sep 19
Pyrazinamide	pncA_P54R	R	Yadon et. al., Nature Communications 2017 Sep 19
Pyrazinamide	pncA_P54T	R	Yadon et. al., Nature Communications 2017 Sep 19
Pyrazinamide	pncA_P62A	R	Yadon et. al., Nature Communications 2017 Sep 19
Pyrazinamide	pncA_P62P	R	Yadon et. al., Nature Communications 2017 Sep 19
Pyrazinamide	pncA_A143D	R	Yadon et. al., Nature Communications 2017 Sep 19
Pyrazinamide	pncA_P62R	R	Yadon et. al., Nature Communications 2017 Sep 19
Pyrazinamide	pncA_P62S	R	Yadon et. al., Nature Communications 2017 Sep 19
Pyrazinamide	pncA_P69L	R	Yadon et. al., Nature Communications 2017 Sep 19
Pyrazinamide	pncA_P69Q	R	Yadon et. al., Nature Communications 2017 Sep 19
Pyrazinamide	pncA_P70A	R	Yadon et. al., Nature Communications 2017 Sep 19
Pyrazinamide	pncA_P70S	R	Yadon et. al., Nature Communications 2017 Sep 19
Pyrazinamide	pncA_P77S	R	Yadon et. al., Nature Communications 2017 Sep 19
Pyrazinamide	pncA_Q10Stop	R	Yadon et. al., Nature Communications 2017 Sep 19
Pyrazinamide	pncA_Q10E	R	Yadon et. al., Nature Communications 2017 Sep 19
Pyrazinamide	pncA_Q10H	R	Yadon et. al., Nature Communications 2017 Sep 19
Pyrazinamide	pncA_A146E	R	Yadon et. al., Nature Communications 2017 Sep 19
Pyrazinamide	pncA_Q10L	R	Yadon et. al., Nature Communications 2017 Sep 19
Pyrazinamide	pncA_Q122Stop	R	Yadon et. al., Nature Communications 2017 Sep 19
Pyrazinamide	pncA_Q122K	R	Yadon et. al., Nature Communications 2017 Sep 19
Pyrazinamide	pncA_Q122Q	R	Yadon et. al., Nature Communications 2017 Sep 19
Pyrazinamide	pncA_R123S	R	Yadon et. al., Nature Communications 2017 Sep 19
Pyrazinamide	pncA_R140P	R	Yadon et. al., Nature Communications 2017 Sep 19
Pyrazinamide	pncA_R154M	R	Yadon et. al., Nature Communications 2017 Sep 19
Pyrazinamide	pncA_R176P	R	Yadon et. al., Nature Communications 2017 Sep 19
Pyrazinamide	pncA_R29C	R	Yadon et. al., Nature Communications 2017 Sep 19
Pyrazinamide	pncA_S104C	R	Yadon et. al., Nature Communications 2017 Sep 19
Pyrazinamide	pncA_A146S	R	Yadon et. al., Nature Communications 2017 Sep 19
Pyrazinamide	pncA_S164Stop	R	Yadon et. al., Nature Communications 2017 Sep 19
Pyrazinamide	pncA_S164P	R	Yadon et. al., Nature Communications 2017 Sep 19
Pyrazinamide	pncA_S164S	R	Yadon et. al., Nature Communications 2017 Sep 19
Pyrazinamide	pncA_S164T	R	Yadon et. al., Nature Communications 2017 Sep 19
Pyrazinamide	pncA_S179N	R	Yadon et. al., Nature Communications 2017 Sep 19
Pyrazinamide	pncA_S18Stop	R	Yadon et. al., Nature Communications 2017 Sep 19
Pyrazinamide	pncA_S186F	R	Yadon et. al., Nature Communications 2017 Sep 19
Pyrazinamide	pncA_S18L	R	Yadon et. al., Nature Communications 2017 Sep 19
Pyrazinamide	pncA_S18T	R	Yadon et. al., Nature Communications 2017 Sep 19
Pyrazinamide	pncA_S32N	R	Yadon et. al., Nature Communications 2017 Sep 19
Pyrazinamide	pncA_A146V	R	Yadon et. al., Nature Communications 2017 Sep 19
Pyrazinamide	pncA_S59F	R	Yadon et. al., Nature Communications 2017 Sep 19
Pyrazinamide	pncA_S65F	R	Yadon et. al., Nature Communications 2017 Sep 19
Pyrazinamide	pncA_S66Stop	R	Yadon et. al., Nature Communications 2017 Sep 19
Pyrazinamide	pncA_S67Stop	R	Yadon et. al., Nature Communications 2017 Sep 19

Pyrazinamide	pncA_S88Stop	R	Yadon et. al., Nature Communications 2017 Sep 19
Pyrazinamide	pncA_S88T	R	Yadon et. al., Nature Communications 2017 Sep 19
Pyrazinamide	pncA_T114A	R	Yadon et. al., Nature Communications 2017 Sep 19
Pyrazinamide	pncA_T114T	R	Yadon et. al., Nature Communications 2017 Sep 19
Pyrazinamide	pncA_T135I	R	Yadon et. al., Nature Communications 2017 Sep 19
Pyrazinamide	pncA_T135S	R	Yadon et. al., Nature Communications 2017 Sep 19
Pyrazinamide	pncA_A152V	R	Yadon et. al., Nature Communications 2017 Sep 19
Pyrazinamide	pncA_T142R	R	Yadon et. al., Nature Communications 2017 Sep 19
Pyrazinamide	pncA_T142T	R	Yadon et. al., Nature Communications 2017 Sep 19
Pyrazinamide	pncA_T153N	R	Yadon et. al., Nature Communications 2017 Sep 19
Pyrazinamide	pncA_T160K	R	Yadon et. al., Nature Communications 2017 Sep 19
Pyrazinamide	pncA_T160R	R	Yadon et. al., Nature Communications 2017 Sep 19
Pyrazinamide	pncA_T177T	R	Yadon et. al., Nature Communications 2017 Sep 19
Pyrazinamide	pncA_T22I	R	Yadon et. al., Nature Communications 2017 Sep 19
Pyrazinamide	pncA_T47S	R	Yadon et. al., Nature Communications 2017 Sep 19
Pyrazinamide	pncA_T76I	R	Yadon et. al., Nature Communications 2017 Sep 19
Pyrazinamide	pncA_T76S	R	Yadon et. al., Nature Communications 2017 Sep 19
Pyrazinamide	pncA_A161P	R	Yadon et. al., Nature Communications 2017 Sep 19
Pyrazinamide	pncA_V109L	R	Yadon et. al., Nature Communications 2017 Sep 19
Pyrazinamide	pncA_V125A	R	Yadon et. al., Nature Communications 2017 Sep 19
Pyrazinamide	pncA_V125D	R	Yadon et. al., Nature Communications 2017 Sep 19
Pyrazinamide	pncA_V125V	R	Yadon et. al., Nature Communications 2017 Sep 19
Pyrazinamide	pncA_V128A	R	Yadon et. al., Nature Communications 2017 Sep 19
Pyrazinamide	pncA_V128D	R	Yadon et. al., Nature Communications 2017 Sep 19
Pyrazinamide	pncA_V130E	R	Yadon et. al., Nature Communications 2017 Sep 19
Pyrazinamide	pncA_V131D	R	Yadon et. al., Nature Communications 2017 Sep 19
Pyrazinamide	pncA_V131F	R	Yadon et. al., Nature Communications 2017 Sep 19
Pyrazinamide	pncA_V131I	R	Yadon et. al., Nature Communications 2017 Sep 19
Pyrazinamide	pncA_A165D	R	Yadon et. al., Nature Communications 2017 Sep 19
Pyrazinamide	pncA_V139E	R	Yadon et. al., Nature Communications 2017 Sep 19
Pyrazinamide	pncA_V139M	R	Yadon et. al., Nature Communications 2017 Sep 19
Pyrazinamide	pncA_V147A	R	Yadon et. al., Nature Communications 2017 Sep 19
Pyrazinamide	pncA_V155E	R	Yadon et. al., Nature Communications 2017 Sep 19
Pyrazinamide	pncA_V155M	R	Yadon et. al., Nature Communications 2017 Sep 19
Pyrazinamide	pncA_V163E	R	Yadon et. al., Nature Communications 2017 Sep 19
Pyrazinamide	pncA_V180D	R	Yadon et. al., Nature Communications 2017 Sep 19
Pyrazinamide	pncA_V183D	R	Yadon et. al., Nature Communications 2017 Sep 19
Pyrazinamide	pncA_V183V	R	Yadon et. al., Nature Communications 2017 Sep 19
Pyrazinamide	pncA_V21A	R	Yadon et. al., Nature Communications 2017 Sep 19
Pyrazinamide	pncA_A170S	R	Yadon et. al., Nature Communications 2017 Sep 19
Pyrazinamide	pncA_V21E	R	Yadon et. al., Nature Communications 2017 Sep 19
Pyrazinamide	pncA_V44D	R	Yadon et. al., Nature Communications 2017 Sep 19
Pyrazinamide	pncA_V7A	R	Yadon et. al., Nature Communications 2017 Sep 19
Pyrazinamide	pncA_V7D	R	Yadon et. al., Nature Communications 2017 Sep 19
Pyrazinamide	pncA_V7F	R	Yadon et. al., Nature Communications 2017 Sep 19
Pyrazinamide	pncA_V93E	R	Yadon et. al., Nature Communications 2017 Sep 19
Pyrazinamide	pncA_V93L	R	Yadon et. al., Nature Communications 2017 Sep 19
Pyrazinamide	pncA_W119Stop	R	Yadon et. al., Nature Communications 2017 Sep 19
Pyrazinamide	pncA_W119G	R	Yadon et. al., Nature Communications 2017 Sep 19
Pyrazinamide	pncA_W119L	R	Yadon et. al., Nature Communications 2017 Sep 19
Pyrazinamide	pncA_A170V	R	Yadon et. al., Nature Communications 2017 Sep 19
Pyrazinamide	pncA_W119R	R	Yadon et. al., Nature Communications 2017 Sep 19
Pyrazinamide	pncA_W68Stop	R	Yadon et. al., Nature Communications 2017 Sep 19
Pyrazinamide	pncA_W68L	R	Yadon et. al., Nature Communications 2017 Sep 19
Pyrazinamide	pncA_Y103Stop	R	Yadon et. al., Nature Communications 2017 Sep 19
Pyrazinamide	pncA_Y34Y	R	Yadon et. al., Nature Communications 2017 Sep 19
Pyrazinamide	pncA_Y41Stop	R	Yadon et. al., Nature Communications 2017 Sep 19
Pyrazinamide	pncA_Y41H	R	Yadon et. al., Nature Communications 2017 Sep 19
Pyrazinamide	pncA_Y41Y	R	Yadon et. al., Nature Communications 2017 Sep 19
Pyrazinamide	pncA_Y64Stop	R	Yadon et. al., Nature Communications 2017 Sep 19
Pyrazinamide	pncA_Y95Stop	R	Yadon et. al., Nature Communications 2017 Sep 19
Pyrazinamide	pncA_A171A	R	Yadon et. al., Nature Communications 2017 Sep 19
Rifampicin	rpoB_1300_ins	R	ReSeqTB systematic review
Rifampicin	rpoB_1306_ins	R	ReSeqTB systematic review
Rifampicin	rpoB_1309_del	R	ReSeqTB systematic review
Pyrazinamide	pncA_A171T	R	Yadon et. al., Nature Communications 2017 Sep 19
Pyrazinamide	pncA_A26V	R	Yadon et. al., Nature Communications 2017 Sep 19
Pyrazinamide	pncA_A28S	R	Yadon et. al., Nature Communications 2017 Sep 19
Pyrazinamide	pncA_A30P	R	Yadon et. al., Nature Communications 2017 Sep 19
Pyrazinamide	pncA_A30S	R	Yadon et. al., Nature Communications 2017 Sep 19
Pyrazinamide	pncA_A30V	R	Yadon et. al., Nature Communications 2017 Sep 19
Pyrazinamide	pncA_A36D	R	Yadon et. al., Nature Communications 2017 Sep 19
Pyrazinamide	pncA_A36S	R	Yadon et. al., Nature Communications 2017 Sep 19
Pyrazinamide	pncA_A38A	R	Yadon et. al., Nature Communications 2017 Sep 19
Pyrazinamide	pncA_A38G	R	Yadon et. al., Nature Communications 2017 Sep 19
Pyrazinamide	pncA_A38S	R	Yadon et. al., Nature Communications 2017 Sep 19
Pyrazinamide	pncA_A3A	R	Yadon et. al., Nature Communications 2017 Sep 19
Pyrazinamide	pncA_A46A	R	Yadon et. al., Nature Communications 2017 Sep 19
Pyrazinamide	pncA_A46E	R	Yadon et. al., Nature Communications 2017 Sep 19
Rifampicin	rpoB_D435A	R	ReSeqTB systematic review
Rifampicin	rpoB_D435F	R	ReSeqTB systematic review

Rifampicin	rpoB_D435G	R	ReSeqTB systematic review
Rifampicin	rpoB_D435N	R	ReSeqTB systematic review
Rifampicin	rpoB_D435V	R	ReSeqTB systematic review
Rifampicin	rpoB_D435Y	R	ReSeqTB systematic review
Pyrazinamide	pncA_A46P	R	Yadon et. al., Nature Communications 2017 Sep 19
Pyrazinamide	pncA_A79A	R	Yadon et. al., Nature Communications 2017 Sep 19
Pyrazinamide	pncA_A79P	R	Yadon et. al., Nature Communications 2017 Sep 19
Rifampicin	rpoB_D545E	R	ReSeqTB systematic review
Pyrazinamide	pncA_A89V	R	Yadon et. al., Nature Communications 2017 Sep 19
Pyrazinamide	pncA_A92E	R	Yadon et. al., Nature Communications 2017 Sep 19
Pyrazinamide	pncA_C138Stop	R	Yadon et. al., Nature Communications 2017 Sep 19
Pyrazinamide	pncA_C138F	R	Yadon et. al., Nature Communications 2017 Sep 19
Pyrazinamide	pncA_C138G	R	Yadon et. al., Nature Communications 2017 Sep 19
Pyrazinamide	pncA_C138S	R	Yadon et. al., Nature Communications 2017 Sep 19
Pyrazinamide	pncA_C14Stop	R	Yadon et. al., Nature Communications 2017 Sep 19
Pyrazinamide	pncA_C14W	R	Yadon et. al., Nature Communications 2017 Sep 19
Pyrazinamide	pncA_C14Y	R	Yadon et. al., Nature Communications 2017 Sep 19
Pyrazinamide	pncA_C184R	R	Yadon et. al., Nature Communications 2017 Sep 19
Pyrazinamide	pncA_C72Stop	R	Yadon et. al., Nature Communications 2017 Sep 19
Pyrazinamide	pncA_C72F	R	Yadon et. al., Nature Communications 2017 Sep 19
Pyrazinamide	pncA_C72W	R	Yadon et. al., Nature Communications 2017 Sep 19
Pyrazinamide	pncA_C72Y	R	Yadon et. al., Nature Communications 2017 Sep 19
Rifampicin	rpoB_H445C	R	ReSeqTB systematic review
Rifampicin	rpoB_H445D	R	ReSeqTB systematic review
Rifampicin	rpoB_H445F	R	ReSeqTB systematic review
Rifampicin	rpoB_H445G	R	ReSeqTB systematic review
Rifampicin	rpoB_H445L	R	ReSeqTB systematic review
Rifampicin	rpoB_H445N	R	ReSeqTB systematic review
Rifampicin	rpoB_H445P	R	ReSeqTB systematic review
Rifampicin	rpoB_H445R	R	ReSeqTB systematic review
Rifampicin	rpoB_H445Y	R	ReSeqTB systematic review
Pyrazinamide	pncA_D110E	R	Yadon et. al., Nature Communications 2017 Sep 19
Rifampicin	rpoB_I491F	R	ReSeqTB systematic review
Pyrazinamide	pncA_D110H	R	Yadon et. al., Nature Communications 2017 Sep 19
Pyrazinamide	pncA_D110N	R	Yadon et. al., Nature Communications 2017 Sep 19
Pyrazinamide	pncA_D129E	R	Yadon et. al., Nature Communications 2017 Sep 19
Rifampicin	rpoB_L430P	R	ReSeqTB systematic review
Pyrazinamide	pncA_D12E	R	Yadon et. al., Nature Communications 2017 Sep 19
Rifampicin	rpoB_L452P	R	ReSeqTB systematic review
Pyrazinamide	pncA_D12H	R	Yadon et. al., Nature Communications 2017 Sep 19
Pyrazinamide	pncA_D12Y	R	Yadon et. al., Nature Communications 2017 Sep 19
Pyrazinamide	pncA_D136H	R	Yadon et. al., Nature Communications 2017 Sep 19
Pyrazinamide	pncA_D136V	R	Yadon et. al., Nature Communications 2017 Sep 19
Pyrazinamide	pncA_D136Y	R	Yadon et. al., Nature Communications 2017 Sep 19
Pyrazinamide	pncA_D145E	R	Yadon et. al., Nature Communications 2017 Sep 19
Pyrazinamide	pncA_D158G	R	Yadon et. al., Nature Communications 2017 Sep 19
Pyrazinamide	pncA_D166V	R	Yadon et. al., Nature Communications 2017 Sep 19
Pyrazinamide	pncA_D49E	R	Yadon et. al., Nature Communications 2017 Sep 19
Pyrazinamide	pncA_D49H	R	Yadon et. al., Nature Communications 2017 Sep 19
Pyrazinamide	pncA_D49V	R	Yadon et. al., Nature Communications 2017 Sep 19
Pyrazinamide	pncA_D49Y	R	Yadon et. al., Nature Communications 2017 Sep 19
Pyrazinamide	pncA_D56D	R	Yadon et. al., Nature Communications 2017 Sep 19
Rifampicin	rpoB_Q432K	R	ReSeqTB systematic review
Rifampicin	rpoB_Q432L	R	ReSeqTB systematic review
Rifampicin	rpoB_Q432P	R	ReSeqTB systematic review
Pyrazinamide	pncA_D63E	R	Yadon et. al., Nature Communications 2017 Sep 19
Pyrazinamide	pncA_D63H	R	Yadon et. al., Nature Communications 2017 Sep 19
Pyrazinamide	pncA_D63Y	R	Yadon et. al., Nature Communications 2017 Sep 19
Pyrazinamide	pncA_D80H	R	Yadon et. al., Nature Communications 2017 Sep 19
Pyrazinamide	pncA_D86V	R	Yadon et. al., Nature Communications 2017 Sep 19
Pyrazinamide	pncA_D8A	R	Yadon et. al., Nature Communications 2017 Sep 19
Rifampicin	rpoB_S431T	R	ReSeqTB systematic review
Rifampicin	rpoB_S441L	R	ReSeqTB systematic review
Rifampicin	rpoB_S441Q	R	ReSeqTB systematic review
Rifampicin	rpoB_S450F	R	ReSeqTB systematic review
Rifampicin	rpoB_S450L	R	ReSeqTB systematic review
Rifampicin	rpoB_S450Q	R	ReSeqTB systematic review
Rifampicin	rpoB_S450W	R	ReSeqTB systematic review
Rifampicin	rpoB_S450Y	R	ReSeqTB systematic review
Pyrazinamide	pncA_D8H	R	Yadon et. al., Nature Communications 2017 Sep 19
Pyrazinamide	pncA_D8V	R	Yadon et. al., Nature Communications 2017 Sep 19
Pyrazinamide	pncA_D8Y	R	Yadon et. al., Nature Communications 2017 Sep 19
Pyrazinamide	pncA_E107E	R	Yadon et. al., Nature Communications 2017 Sep 19
Pyrazinamide	pncA_E111Stop	R	Yadon et. al., Nature Communications 2017 Sep 19
Pyrazinamide	pncA_E127Stop	R	Yadon et. al., Nature Communications 2017 Sep 19
Pyrazinamide	pncA_E127D	R	Yadon et. al., Nature Communications 2017 Sep 19
Pyrazinamide	pncA_E144Stop	R	Yadon et. al., Nature Communications 2017 Sep 19
Pyrazinamide	pncA_E15Stop	R	Yadon et. al., Nature Communications 2017 Sep 19
Pyrazinamide	pncA_E173Stop	R	Yadon et. al., Nature Communications 2017 Sep 19
Pyrazinamide	pncA_E173G	R	Yadon et. al., Nature Communications 2017 Sep 19
Pyrazinamide	pncA_E174G	R	Yadon et. al., Nature Communications 2017 Sep 19

Pyrazinamide	pncA_E174K	R	Yadon et. al., Nature Communications 2017 Sep 19
Pyrazinamide	pncA_E181Stop	R	Yadon et. al., Nature Communications 2017 Sep 19
Pyrazinamide	pncA_E181K	R	Yadon et. al., Nature Communications 2017 Sep 19

S3

For each drug (for which the phenotype is marked with an * in the table below, and presented in the order 'isoniazid, rifampicin, ethambutol, pyrazinamide'), against each possible combination of phenotypes among the other three drugs, the negative predictive value was calculated for a prevelance of resistance to the drug in question between 1%-100%. The formula by which the NPV was calculated is: specificity*(1-prevlence)/(specificity*(1-prevlence)+(1-sensitivity)*prevalence)

-SSS				-RSS				-RRS				-SRS			
Prevalence of isoniazid resistance	Sensitivity	Specificity	Negative Predictive Value	Prevalence of isoniazid resistance	Sensitivity	Specificity	Negative Predictive Value	Prevalence of isoniazid resistance	Sensitivity	Specificity	Negative Predictive Value	Prevalence of isoniazid resistance	Sensitivity	Specificity	Negative Predictive Value
1	92.9	99.6	99.9	1	95.6	92.4	100.0	1	99.3	50.0	100.0	1	85.7	100.0	99.9
2	92.9	99.6	99.9	2	95.6	92.4	99.9	2	99.3	50.0	100.0	2	85.7	100.0	99.7
3	92.9	99.6	99.8	3	95.6	92.4	99.9	3	99.3	50.0	100.0	3	85.7	100.0	99.6
4	92.9	99.6	99.7	4	95.6	92.4	99.8	4	99.3	50.0	99.9	4	85.7	100.0	99.4
5	92.9	99.6	99.6	5	95.6	92.4	99.8	5	99.3	50.0	99.9	5	85.7	100.0	99.3
6	92.9	99.6	99.5	6	95.6	92.4	99.7	6	99.3	50.0	99.9	6	85.7	100.0	99.1
7	92.9	99.6	99.5	7	95.6	92.4	99.6	7	99.3	50.0	99.9	7	85.7	100.0	98.9
8	92.9	99.6	99.4	8	95.6	92.4	99.6	8	99.3	50.0	99.9	8	85.7	100.0	98.8
9	92.9	99.6	99.3	9	95.6	92.4	99.5	9	99.3	50.0	99.9	9	85.7	100.0	98.6
10	92.9	99.6	99.2	10	95.6	92.4	99.5	10	99.3	50.0	99.8	10	85.7	100.0	98.4
11	92.9	99.6	99.1	11	95.6	92.4	99.4	11	99.3	50.0	99.8	11	85.7	100.0	98.3
12	92.9	99.6	99.0	12	95.6	92.4	99.4	12	99.3	50.0	99.8	12	85.7	100.0	98.1
13	92.9	99.6	98.9	13	95.6	92.4	99.3	13	99.3	50.0	99.8	13	85.7	100.0	97.9
14	92.9	99.6	98.8	14	95.6	92.4	99.2	14	99.3	50.0	99.8	14	85.7	100.0	97.7
15	92.9	99.6	98.8	15	95.6	92.4	99.2	15	99.3	50.0	99.8	15	85.7	100.0	97.5
16	92.9	99.6	98.7	16	95.6	92.4	99.1	16	99.3	50.0	99.7	16	85.7	100.0	97.4
17	92.9	99.6	98.6	17	95.6	92.4	99.0	17	99.3	50.0	99.7	17	85.7	100.0	97.2
18	92.9	99.6	98.5	18	95.6	92.4	99.0	18	99.3	50.0	99.7	18	85.7	100.0	97.0
19	92.9	99.6	98.3	19	95.6	92.4	98.9	19	99.3	50.0	99.7	19	85.7	100.0	96.8
20	92.9	99.6	98.2	20	95.6	92.4	98.8	20	99.3	50.0	99.6	20	85.7	100.0	96.6
21	92.9	99.6	98.1	21	95.6	92.4	98.8	21	99.3	50.0	99.6	21	85.7	100.0	96.3
22	92.9	99.6	98.0	22	95.6	92.4	98.7	22	99.3	50.0	99.6	22	85.7	100.0	96.1
23	92.9	99.6	97.9	23	95.6	92.4	98.6	23	99.3	50.0	99.6	23	85.7	100.0	95.9
24	92.9	99.6	97.8	24	95.6	92.4	98.5	24	99.3	50.0	99.6	24	85.7	100.0	95.7
25	92.9	99.6	97.7	25	95.6	92.4	98.4	25	99.3	50.0	99.5	25	85.7	100.0	95.5
26	92.9	99.6	97.5	26	95.6	92.4	98.4	26	99.3	50.0	99.5	26	85.7	100.0	95.2
27	92.9	99.6	97.4	27	95.6	92.4	98.3	27	99.3	50.0	99.5	27	85.7	100.0	95.0
28	92.9	99.6	97.3	28	95.6	92.4	98.2	28	99.3	50.0	99.5	28	85.7	100.0	94.7
29	92.9	99.6	97.2	29	95.6	92.4	98.1	29	99.3	50.0	99.4	29	85.7	100.0	94.5
30	92.9	99.6	97.0	30	95.6	92.4	98.0	30	99.3	50.0	99.4	30	85.7	100.0	94.2
31	92.9	99.6	96.9	31	95.6	92.4	97.9	31	99.3	50.0	99.4	31	85.7	100.0	94.0
32	92.9	99.6	96.7	32	95.6	92.4	97.8	32	99.3	50.0	99.3	32	85.7	100.0	93.7
33	92.9	99.6	96.6	33	95.6	92.4	97.7	33	99.3	50.0	99.3	33	85.7	100.0	93.4
34	92.9	99.6	96.4	34	95.6	92.4	97.6	34	99.3	50.0	99.3	34	85.7	100.0	93.1
35	92.9	99.6	96.3	35	95.6	92.4	97.5	35	99.3	50.0	99.2	35	85.7	100.0	92.9
36	92.9	99.6	96.1	36	95.6	92.4	97.4	36	99.3	50.0	99.2	36	85.7	100.0	92.6
37	92.9	99.6	96.0	37	95.6	92.4	97.3	37	99.3	50.0	99.2	37	85.7	100.0	92.3
38	92.9	99.6	95.8	38	95.6	92.4	97.2	38	99.3	50.0	99.1	38	85.7	100.0	91.9
39	92.9	99.6	95.6	39	95.6	92.4	97.1	39	99.3	50.0	99.1	39	85.7	100.0	91.6
40	92.9	99.6	95.4	40	95.6	92.4	96.9	40	99.3	50.0	99.1	40	85.7	100.0	91.3
41	92.9	99.6	95.3	41	95.6	92.4	96.8	41	99.3	50.0	99.0	41	85.7	100.0	91.0
42	92.9	99.6	95.1	42	95.6	92.4	96.7	42	99.3	50.0	99.0	42	85.7	100.0	90.6
43	92.9	99.6	94.9	43	95.6	92.4	96.6	43	99.3	50.0	99.0	43	85.7	100.0	90.3
44	92.9	99.6	94.7	44	95.6	92.4	96.4	44	99.3	50.0	98.9	44	85.7	100.0	89.9
45	92.9	99.6	94.5	45	95.6	92.4	96.3	45	99.3	50.0	98.9	45	85.7	100.0	89.5
46	92.9	99.6	94.3	46	95.6	92.4	96.1	46	99.3	50.0	98.8	46	85.7	100.0	89.2
47	92.9	99.6	94.0	47	95.6	92.4	96.0	47	99.3	50.0	98.8	47	85.7	100.0	88.8
48	92.9	99.6	93.8	48	95.6	92.4	95.8	48	99.3	50.0	98.7	48	85.7	100.0	88.3
49	92.9	99.6	93.6	49	95.6	92.4	95.6	49	99.3	50.0	98.7	49	85.7	100.0	87.9
50	92.9	99.6	93.3	50	95.6	92.4	95.5	50	99.3	50.0	98.6	50	85.7	100.0	87.5
51	92.9	99.6	93.1	51	95.6	92.4	95.3	51	99.3	50.0	98.6	51	85.7	100.0	87.1
52	92.9	99.6	92.8	52	95.6	92.4	95.1	52	99.3	50.0	98.5	52	85.7	100.0	86.6
53	92.9	99.6	92.5	53	95.6	92.4	94.9	53	99.3	50.0	98.4	53	85.7	100.0	86.1
54	92.9	99.6	92.2	54	95.6	92.4	94.7	54	99.3	50.0	98.4	54	85.7	100.0	85.6
55	92.9	99.6	92.0	55	95.6	92.4	94.5	55	99.3	50.0	98.3	55	85.7	100.0	85.1
56	92.9	99.6	91.7	56	95.6	92.4	94.3	56	99.3	50.0	98.2	56	85.7	100.0	84.6
57	92.9	99.6	91.3	57	95.6	92.4	94.1	57	99.3	50.0	98.2	57	85.7	100.0	84.1
58	92.9	99.6	91.0	58	95.6	92.4	93.9	58	99.3	50.0	98.1	58	85.7	100.0	83.5
59	92.9	99.6	90.7	59	95.6	92.4	93.6	59	99.3	50.0	98.0	59	85.7	100.0	82.9
60	92.9	99.6	90.3	60	95.6	92.4	93.4	60	99.3	50.0	97.9	60	85.7	100.0	82.4
61	92.9	99.6	89.9	61	95.6	92.4	93.1	61	99.3	50.0	97.8	61	85.7	100.0	81.7
62	92.9	99.6	89.5	62	95.6	92.4	92.8	62	99.3	50.0	97.8	62	85.7	100.0	81.1
63	92.9	99.6	89.1	63	95.6	92.4	92.5	63	99.3	50.0	97.7	63	85.7	100.0	80.4
64	92.9	99.6	88.7	64	95.6	92.4	92.2	64	99.3	50.0	97.6	64	85.7	100.0	79.7
65	92.9	99.6	88.3	65	95.6	92.4	91.9	65	99.3	50.0	97.5	65	85.7	100.0	79.0
66	92.9	99.6	87.8	66	95.6	92.4	91.6	66	99.3	50.0	97.3	66	85.7	100.0	78.3
67	92.9	99.6	87.3	67	95.6	92.4	91.2	67	99.3	50.0	97.2	67	85.7	100.0	77.5
68	92.9	99.6	86.8	68	95.6	92.4	90.9	68	99.3	50.0	97.1	68	85.7	100.0	76.7
69	92.9	99.6	86.3	69	95.6	92.4	90.5	69	99.3	50.0	97.0	69	85.7	100.0	75.9
70	92.9	99.6	85.7	70	95.6	92.4	90.1	70	99.3	50.0	96.8	70	85.7	100.0	75.0
71	92.9	99.6	85.1	71	95.6	92.4	89.6	71	99.3	50.0	96.7	71	85.7	100.0	74.1
72	92.9	99.6	84.5	72	95.6	92.4	89.1	72	99.3	50.0	96.5	72	85.7	100.0	73.1
73	92.9	99.6	83.8	73	95.6	92.4	88.7	73	99.3	50.0	96.3	73	85.7	100.0	72.1
74	92.9	99.6	83.1	74	95.6	92.4	88.1	74	99.3	50.0	96.2	74	85.7	100.0	71.1
75	92.9	99.6	82.3	75	95.6	92.4	87.6	75	99.3	50.0	96.0	75	85.7	100.0	70.0
76	92.9	99.6	81.5	76	95.6	92.4	87.0	76	99.3	50.0	95.7	76	85.7	100.0	68.9
77	92.9	99.6	80.7	77	95.6	92.4	86.3	77	99.3	50.0	95.5	77	85.7	100.0	67.6
78	92.9	99.6	79.8	78	95.6	92.4	85.6	78	99.3	50.0	95.3	78	85.7	100.0	66.4
79	92.9	99.6	78.8	79	95.6	92.4	84.9	79	99.3	50.0	95.0	79	85.7	100.0	65.0
80	92.9	99.6	77.7	80	95.6	92.4	84.1	80	99.3	50.0	94.7	80	85.7	100.0	63.6
81	92.9	99.6	76.6	81	95.6	92.4	83.2	81	99.3	50.0	94.3	81	85.7	100.0	62.1
82	92.9	99.6	75.4	82	95.6	92.4	82.3	82	99.3	50.0	94.0	82	85.7	100.0	60.6
83	92.9	99.6	74.1	83	95.6	92.4	81.2	83	99.3	50.0	93.6	83	85.7	100.0	58.9
84	92.9	99.6	72.7	84	95.6	92.4	80.1	84	99.3	50.0	93.1	84	85.7	100.0	57.1
85	92.9	99.6	71.1	85	95.6	92.4	78.8	85	99.3	50.0	92.6	85	85.7	100.0	55.3
86	92.9	99.6	69.5	86	95.6	92.4	77.5	86	99.3	50.0	92.1	86	85.7	100.0	53.3
87	92.9	99.6	67.6	87	95.6	92.4	75.9	87	99.3	50.0	91.4	87	85.7	100.0	51.1
88	92.9	99.6	65.6	88	95.6	92.4	74.2	88	99.3	50.0	90.7	88	85.7	100.0	48.8
89	92.9	99.6	63.3	89	95.6	92.4	72.3	89	99.3	50.0	89.8	89	85.7	100.0	46.4
90	92.9	99.6	60.8	90	95.6	92.4	70.1	90	99.3	50.0	88.8	90	85.7	100.0	43.8

91	92.9	99.6	58.0
92	92.9	99.6	54.8
93	92.9	99.6	51.3
94	92.9	99.6	47.1
95	92.9	99.6	42.4
96	92.9	99.6	36.8
97	92.9	99.6	30.2
98	92.9	99.6	22.2
99	92.9	99.6	12.4
100	92.9	99.6	0.0

91	95.6	92.4	67.6
92	95.6	92.4	64.7
93	95.6	92.4	61.4
94	95.6	92.4	57.4
95	95.6	92.4	52.6
96	95.6	92.4	46.8
97	95.6	92.4	39.5
98	95.6	92.4	30.1
99	95.6	92.4	17.6
100	95.6	92.4	0.0

91	99.3	50.0	87.6
92	99.3	50.0	86.1
93	99.3	50.0	84.3
94	99.3	50.0	82.0
95	99.3	50.0	78.9
96	99.3	50.0	74.8
97	99.3	50.0	68.8
98	99.3	50.0	59.2
99	99.3	50.0	41.8
100	99.3	50.0	0.0

91	85.7	100.0	40.9
92	85.7	100.0	37.8
93	85.7	100.0	34.5
94	85.7	100.0	30.9
95	85.7	100.0	26.9
96	85.7	100.0	22.6
97	85.7	100.0	17.8
98	85.7	100.0	12.5
99	85.7	100.0	6.6
100	85.7	100.0	0.0

-SSR

Prevalence of isoniazid resistance	Sensitivity	Specificity	Negative Predictive Value
1	96.0	100.0	100.0
2	96.0	100.0	99.9
3	96.0	100.0	99.9
4	96.0	100.0	99.8
5	96.0	100.0	99.8
6	96.0	100.0	99.7
7	96.0	100.0	99.7
8	96.0	100.0	99.7
9	96.0	100.0	99.6
10	96.0	100.0	99.6
11	96.0	100.0	99.5
12	96.0	100.0	99.5
13	96.0	100.0	99.4
14	96.0	100.0	99.4
15	96.0	100.0	99.3
16	96.0	100.0	99.2
17	96.0	100.0	99.2
18	96.0	100.0	99.1
19	96.0	100.0	99.1
20	96.0	100.0	99.0
21	96.0	100.0	98.9
22	96.0	100.0	98.9
23	96.0	100.0	98.8
24	96.0	100.0	98.8
25	96.0	100.0	98.7
26	96.0	100.0	98.6
27	96.0	100.0	98.5
28	96.0	100.0	98.5
29	96.0	100.0	98.4
30	96.0	100.0	98.3
31	96.0	100.0	98.2
32	96.0	100.0	98.2
33	96.0	100.0	98.1
34	96.0	100.0	98.0
35	96.0	100.0	97.9
36	96.0	100.0	97.8
37	96.0	100.0	97.7
38	96.0	100.0	97.6
39	96.0	100.0	97.5
40	96.0	100.0	97.4
41	96.0	100.0	97.3
42	96.0	100.0	97.2
43	96.0	100.0	97.1
44	96.0	100.0	97.0
45	96.0	100.0	96.8
46	96.0	100.0	96.7
47	96.0	100.0	96.6
48	96.0	100.0	96.4
49	96.0	100.0	96.3
50	96.0	100.0	96.2
51	96.0	100.0	96.0
52	96.0	100.0	95.8
53	96.0	100.0	95.7
54	96.0	100.0	95.5
55	96.0	100.0	95.3
56	96.0	100.0	95.2
57	96.0	100.0	95.0
58	96.0	100.0	94.8
59	96.0	100.0	94.6
60	96.0	100.0	94.3
61	96.0	100.0	94.1
62	96.0	100.0	93.9
63	96.0	100.0	93.6
64	96.0	100.0	93.4
65	96.0	100.0	93.1
66	96.0	100.0	92.8
67	96.0	100.0	92.5
68	96.0	100.0	92.2
69	96.0	100.0	91.8
70	96.0	100.0	91.5
71	96.0	100.0	91.1
72	96.0	100.0	90.7
73	96.0	100.0	90.2
74	96.0	100.0	89.8
75	96.0	100.0	89.3
76	96.0	100.0	88.8
77	96.0	100.0	88.2
78	96.0	100.0	87.6
79	96.0	100.0	86.9
80	96.0	100.0	86.2
81	96.0	100.0	85.4
82	96.0	100.0	84.6

-RRR

Prevalence of isoniazid resistance	Sensitivity	Specificity	Negative Predictive Value
1	99.5	n/a	n/a
2	99.5	n/a	n/a
3	99.5	n/a	n/a
4	99.5	n/a	n/a
5	99.5	n/a	n/a
6	99.5	n/a	n/a
7	99.5	n/a	n/a
8	99.5	n/a	n/a
9	99.5	n/a	n/a
10	99.5	n/a	n/a
11	99.5	n/a	n/a
12	99.5	n/a	n/a
13	99.5	n/a	n/a
14	99.5	n/a	n/a
15	99.5	n/a	n/a
16	99.5	n/a	n/a
17	99.5	n/a	n/a
18	99.5	n/a	n/a
19	99.5	n/a	n/a
20	99.5	n/a	n/a
21	99.5	n/a	n/a
22	99.5	n/a	n/a
23	99.5	n/a	n/a
24	99.5	n/a	n/a
25	99.5	n/a	n/a
26	99.5	n/a	n/a
27	99.5	n/a	n/a
28	99.5	n/a	n/a
29	99.5	n/a	n/a
30	99.5	n/a	n/a
31	99.5	n/a	n/a
32	99.5	n/a	n/a
33	99.5	n/a	n/a
34	99.5	n/a	n/a
35	99.5	n/a	n/a
36	99.5	n/a	n/a
37	99.5	n/a	n/a
38	99.5	n/a	n/a
39	99.5	n/a	n/a
40	99.5	n/a	n/a
41	99.5	n/a	n/a
42	99.5	n/a	n/a
43	99.5	n/a	n/a
44	99.5	n/a	n/a
45	99.5	n/a	n/a
46	99.5	n/a	n/a
47	99.5	n/a	n/a
48	99.5	n/a	n/a
49	99.5	n/a	n/a
50	99.5	n/a	n/a
51	99.5	n/a	n/a
52	99.5	n/a	n/a
53	99.5	n/a	n/a
54	99.5	n/a	n/a
55	99.5	n/a	n/a
56	99.5	n/a	n/a
57	99.5	n/a	n/a
58	99.5	n/a	n/a
59	99.5	n/a	n/a
60	99.5	n/a	n/a
61	99.5	n/a	n/a
62	99.5	n/a	n/a
63	99.5	n/a	n/a
64	99.5	n/a	n/a
65	99.5	n/a	n/a
66	99.5	n/a	n/a
67	99.5	n/a	n/a
68	99.5	n/a	n/a
69	99.5	n/a	n/a
70	99.5	n/a	n/a
71	99.5	n/a	n/a
72	99.5	n/a	n/a
73	99.5	n/a	n/a
74	99.5	n/a	n/a
75	99.5	n/a	n/a
76	99.5	n/a	n/a
77	99.5	n/a	n/a
78	99.5	n/a	n/a
79	99.5	n/a	n/a
80	99.5	n/a	n/a
81	99.5	n/a	n/a
82	99.5	n/a	n/a

-RSR

Prevalence of isoniazid resistance	Sensitivity	Specificity	Negative Predictive Value
1	98.6	100.0	100.0
2	98.6	100.0	100.0
3	98.6	100.0	100.0
4	98.6	100.0	99.9
5	98.6	100.0	99.9
6	98.6	100.0	99.9
7	98.6	100.0	99.9
8	98.6	100.0	99.9
9	98.6	100.0	99.9
10	98.6	100.0	99.8
11	98.6	100.0	99.8
12	98.6	100.0	99.8
13	98.6	100.0	99.8
14	98.6	100.0	99.8
15	98.6	100.0	99.8
16	98.6	100.0	99.7
17	98.6	100.0	99.7
18	98.6	100.0	99.7
19	98.6	100.0	99.7
20	98.6	100.0	99.7
21	98.6	100.0	99.6
22	98.6	100.0	99.6
23	98.6	100.0	99.6
24	98.6	100.0	99.6
25	98.6	100.0	99.5
26	98.6	100.0	99.5
27	98.6	100.0	99.5
28	98.6	100.0	99.5
29	98.6	100.0	99.4
30	98.6	100.0	99.4
31	98.6	100.0	99.4
32	98.6	100.0	99.4
33	98.6	100.0	99.3
34	98.6	100.0	99.3
35	98.6	100.0	99.3
36	98.6	100.0	99.2
37	98.6	100.0	99.2
38	98.6	100.0	99.2
39	98.6	100.0	99.1
40	98.6	100.0	99.1
41	98.6	100.0	99.1
42	98.6	100.0	99.0
43	98.6	100.0	99.0
44	98.6	100.0	98.9
45	98.6	100.0	98.9
46	98.6	100.0	98.9
47	98.6	100.0	98.8
48	98.6	100.0	98.8
49	98.6	100.0	98.7
50	98.6	100.0	98.7
51	98.6	100.0	98.6
52	98.6	100.0	98.5
53	98.6	100.0	98.5
54	98.6	100.0	98.4
55	98.6	100.0	98.4
56	98.6	100.0	98.3
57	98.6	100.0	98.2
58	98.6	100.0	98.2
59	98.6	100.0	98.1
60	98.6	100.0	98.0
61	98.6	100.0	97.9
62	98.6	100.0	97.8
63	98.6	100.0	97.7
64	98.6	100.0	97.6
65	98.6	100.0	97.5
66	98.6	100.0	97.4
67	98.6	100.0	97.3
68	98.6	100.0	97.2
69	98.6	100.0	97.1
70	98.6	100.0	96.9
71	98.6	100.0	96.8
72	98.6	100.0	96.6
73	98.6	100.0	96.4
74	98.6	100.0	96.3
75	98.6	100.0	96.1
76	98.6	100.0	95.9
77	98.6	100.0	95.6
78	98.6	100.0	95.4
79	98.6	100.0	95.1
80	98.6	100.0	94.8
81	98.6	100.0	94.5
82	98.6	100.0	94.2

-SRR

Prevalence of isoniazid resistance	Sensitivity	Specificity	Negative Predictive Value
1	100.0	n/a	n/a
2	100.0	n/a	n/a
3	100.0	n/a	n/a
4	100.0	n/a	n/a
5	100.0	n/a	n/a
6	100.0	n/a	n/a
7	100.0	n/a	n/a
8	100.0	n/a	n/a
9	100.0	n/a	n/a
10	100.0	n/a	n/a
11	100.0	n/a	n/a
12	100.0	n/a	n/a
13	100.0	n/a	n/a
14	100.0	n/a	n/a
15	100.0	n/a	n/a
16	100.0	n/a	n/a
17	100.0	n/a	n/a
18	100.0	n/a	n/a
19	100.0	n/a	n/a
20	100.0	n/a	n/a
21	100.0	n/a	n/a
22	100.0	n/a	n/a
23	100.0	n/a	n/a
24	100.0	n/a	n/a
25	100.0	n/a	n/a
26	100.0	n/a	n/a
27	100.0	n/a	n/a
28	100.0	n/a	n/a
29	100.0	n/a	n/a
30	100.0	n/a	n/a
31	100.0	n/a	n/a
32	100.0	n/a	n/a
33	100.0	n/a	n/a
34	100.0	n/a	n/a
35	100.0	n/a	n/a
36	100.0	n/a	n/a
37	100.0	n/a	n/a
38	100.0	n/a	n/a
39	100.0	n/a	n/a
40	100.0	n/a	n/a
41	100.0	n/a	n/a
42	100.0	n/a	n/a
43	100.0	n/a	n/a
44	100.0	n/a	n/a
45	100.0	n/a	n/a
46	100.0	n/a	n/a
47	100.0	n/a	n/a
48	100.0	n/a	n/a
49	100.0	n/a	n/a
50	100.0	n/a	n/a
51	100.0	n/a	n/a
52	100.0	n/a	n/a
53	100.0	n/a	n/a
54	100.0	n/a	n/a
55	100.0	n/a	n/a
56	100.0	n/a	n/a
57	100.0	n/a	n/a
58	100.0	n/a	n/a
59	100.0	n/a	n/a
60	100.0	n/a	n/a
61	100.0	n/a	n/a
62	100.0	n/a	n/a
63	100.0	n/a	n/a
64	100.0	n/a	n/a
65	100.0	n/a	n/a
66	100.0	n/a	n/a
67	100.0	n/a	n/a
68	100.0	n/a	n/a
69	100.0	n/a	n/a
70	100.0	n/a	n/a
71	100.0	n/a	n/a
72	100.0	n/a	n/a
73	100.0	n/a	n/a
74	100.0	n/a	n/a
75	100.0	n/a	n/a
76	100.0	n/a	n/a
77	100.0	n/a	n/a
78	100.0	n/a	n/a
79	100.0	n/a	n/a
80	100.0	n/a	n/a
81	100.0	n/a	n/a
82	100.0	n/a	n/a

83	96.0	100.0	83.7
84	96.0	100.0	82.6
85	96.0	100.0	81.5
86	96.0	100.0	80.3
87	96.0	100.0	78.9
88	96.0	100.0	77.3
89	96.0	100.0	75.5
90	96.0	100.0	73.5
91	96.0	100.0	71.2
92	96.0	100.0	68.5
93	96.0	100.0	65.3
94	96.0	100.0	61.5
95	96.0	100.0	56.8
96	96.0	100.0	51.0
97	96.0	100.0	43.6
98	96.0	100.0	33.8
99	96.0	100.0	20.2
100	96.0	100.0	0.0

83	99.5	n/a	n/a
84	99.5	n/a	n/a
85	99.5	n/a	n/a
86	99.5	n/a	n/a
87	99.5	n/a	n/a
88	99.5	n/a	n/a
89	99.5	n/a	n/a
90	99.5	n/a	n/a
91	99.5	n/a	n/a
92	99.5	n/a	n/a
93	99.5	n/a	n/a
94	99.5	n/a	n/a
95	99.5	n/a	n/a
96	99.5	n/a	n/a
97	99.5	n/a	n/a
98	99.5	n/a	n/a
99	99.5	n/a	n/a
100	99.5	n/a	n/a

83	98.6	100.0	93.8
84	98.6	100.0	93.3
85	98.6	100.0	92.8
86	98.6	100.0	92.3
87	98.6	100.0	91.6
88	98.6	100.0	90.9
89	98.6	100.0	90.1
90	98.6	100.0	89.1
91	98.6	100.0	87.9
92	98.6	100.0	86.4
93	98.6	100.0	84.7
94	98.6	100.0	82.4
95	98.6	100.0	79.4
96	98.6	100.0	75.3
97	98.6	100.0	69.4
98	98.6	100.0	59.9
99	98.6	100.0	42.6
100	98.6	100.0	0.0

83	100.0	n/a	n/a
84	100.0	n/a	n/a
85	100.0	n/a	n/a
86	100.0	n/a	n/a
87	100.0	n/a	n/a
88	100.0	n/a	n/a
89	100.0	n/a	n/a
90	100.0	n/a	n/a
91	100.0	n/a	n/a
92	100.0	n/a	n/a
93	100.0	n/a	n/a
94	100.0	n/a	n/a
95	100.0	n/a	n/a
96	100.0	n/a	n/a
97	100.0	n/a	n/a
98	100.0	n/a	n/a
99	100.0	n/a	n/a
100	100.0	n/a	n/a

S-SS

Prevalence of rifampicin resistance	Sensitivity	Specificity	Negative Predictive Value
1	82.2	99.4	99.8
2	82.2	99.4	99.6
3	82.2	99.4	99.4
4	82.2	99.4	99.3
5	82.2	99.4	99.1
6	82.2	99.4	98.9
7	82.2	99.4	98.7
8	82.2	99.4	98.5
9	82.2	99.4	98.3
10	82.2	99.4	98.1
11	82.2	99.4	97.8
12	82.2	99.4	97.6
13	82.2	99.4	97.4
14	82.2	99.4	97.2
15	82.2	99.4	96.9
16	82.2	99.4	96.7
17	82.2	99.4	96.5
18	82.2	99.4	96.2
19	82.2	99.4	96.0
20	82.2	99.4	95.7
21	82.2	99.4	95.5
22	82.2	99.4	95.2
23	82.2	99.4	94.9
24	82.2	99.4	94.7
25	82.2	99.4	94.4
26	82.2	99.4	94.1
27	82.2	99.4	93.8
28	82.2	99.4	93.5
29	82.2	99.4	93.2
30	82.2	99.4	92.9
31	82.2	99.4	92.6
32	82.2	99.4	92.2
33	82.2	99.4	91.9
34	82.2	99.4	91.6
35	82.2	99.4	91.2
36	82.2	99.4	90.9
37	82.2	99.4	90.5
38	82.2	99.4	90.1
39	82.2	99.4	89.7
40	82.2	99.4	89.3
41	82.2	99.4	88.9
42	82.2	99.4	88.5
43	82.2	99.4	88.1
44	82.2	99.4	87.7
45	82.2	99.4	87.2
46	82.2	99.4	86.8
47	82.2	99.4	86.3
48	82.2	99.4	85.8
49	82.2	99.4	85.3
50	82.2	99.4	84.8
51	82.2	99.4	84.3
52	82.2	99.4	83.8
53	82.2	99.4	83.2
54	82.2	99.4	82.6
55	82.2	99.4	82.1
56	82.2	99.4	81.5
57	82.2	99.4	80.8
58	82.2	99.4	80.2
59	82.2	99.4	79.5
60	82.2	99.4	78.8
61	82.2	99.4	78.1
62	82.2	99.4	77.4
63	82.2	99.4	76.6
64	82.2	99.4	75.9
65	82.2	99.4	75.1
66	82.2	99.4	74.2
67	82.2	99.4	73.4
68	82.2	99.4	72.5
69	82.2	99.4	71.5
70	82.2	99.4	70.5
71	82.2	99.4	69.5
72	82.2	99.4	68.5
73	82.2	99.4	67.4

S-RS

Prevalence of rifampicin resistance	Sensitivity	Specificity	Negative Predictive Value
1	100.0	90.0	100.0
2	100.0	90.0	100.0
3	100.0	90.0	100.0
4	100.0	90.0	100.0
5	100.0	90.0	100.0
6	100.0	90.0	100.0
7	100.0	90.0	100.0
8	100.0	90.0	100.0
9	100.0	90.0	100.0
10	100.0	90.0	100.0
11	100.0	90.0	100.0
12	100.0	90.0	100.0
13	100.0	90.0	100.0
14	100.0	90.0	100.0
15	100.0	90.0	100.0
16	100.0	90.0	100.0
17	100.0	90.0	100.0
18	100.0	90.0	100.0
19	100.0	90.0	100.0
20	100.0	90.0	100.0
21	100.0	90.0	100.0
22	100.0	90.0	100.0
23	100.0	90.0	100.0
24	100.0	90.0	100.0
25	100.0	90.0	100.0
26	100.0	90.0	100.0
27	100.0	90.0	100.0
28	100.0	90.0	100.0
29	100.0	90.0	100.0
30	100.0	90.0	100.0
31	100.0	90.0	100.0
32	100.0	90.0	100.0
33	100.0	90.0	100.0
34	100.0	90.0	100.0
35	100.0	90.0	100.0
36	100.0	90.0	100.0
37	100.0	90.0	100.0
38	100.0	90.0	100.0
39	100.0	90.0	100.0
40	100.0	90.0	100.0
41	100.0	90.0	100.0
42	100.0	90.0	100.0
43	100.0	90.0	100.0
44	100.0	90.0	100.0
45	100.0	90.0	100.0
46	100.0	90.0	100.0
47	100.0	90.0	100.0
48	100.0	90.0	100.0
49	100.0	90.0	100.0
50	100.0	90.0	100.0
51	100.0	90.0	100.0
52	100.0	90.0	100.0
53	100.0	90.0	100.0
54	100.0	90.0	100.0
55	100.0	90.0	100.0
56	100.0	90.0	100.0
57	100.0	90.0	100.0
58	100.0	90.0	100.0
59	100.0	90.0	100.0
60	100.0	90.0	100.0
61	100.0	90.0	100.0
62	100.0	90.0	100.0
63	100.0	90.0	100.0
64	100.0	90.0	100.0
65	100.0	90.0	100.0
66	100.0	90.0	100.0
67	100.0	90.0	100.0
68	100.0	90.0	100.0
69	100.0	90.0	100.0
70	100.0	90.0	100.0
71	100.0	90.0	100.0
72	100.0	90.0	100.0
73	100.0	90.0	100.0

S-SR

Prevalence of rifampicin resistance	Sensitivity	Specificity	Negative Predictive Value
1	33.3	100.0	99.3
2	33.3	100.0	98.7
3	33.3	100.0	98.0
4	33.3	100.0	97.3
5	33.3	100.0	96.6
6	33.3	100.0	95.9
7	33.3	100.0	95.2
8	33.3	100.0	94.5
9	33.3	100.0	93.8
10	33.3	100.0	93.1
11	33.3	100.0	92.4
12	33.3	100.0	91.7
13	33.3	100.0	90.9
14	33.3	100.0	90.2
15	33.3	100.0	89.5
16	33.3	100.0	88.7
17	33.3	100.0	88.0
18	33.3	100.0	87.2
19	33.3	100.0	86.5
20	33.3	100.0	85.7
21	33.3	100.0	84.9
22	33.3	100.0	84.2
23	33.3	100.0	83.4
24	33.3	100.0	82.6
25	33.3	100.0	81.8
26	33.3	100.0	81.0
27	33.3	100.0	80.2
28	33.3	100.0	79.4
29	33.3	100.0	78.6
30	33.3	100.0	77.8
31	33.3	100.0	77.0
32	33.3	100.0	76.1
33	33.3	100.0	75.3
34	33.3	100.0	74.4
35	33.3	100.0	73.6
36	33.3	100.0	72.7
37	33.3	100.0	71.9
38	33.3	100.0	71.0
39	33.3	100.0	70.1
40	33.3	100.0	69.2
41	33.3	100.0	68.3
42	33.3	100.0	67.4
43	33.3	100.0	66.5
44	33.3	100.0	65.6
45	33.3	100.0	64.7
46	33.3	100.0	63.8
47	33.3	100.0	62.8
48	33.3	100.0	61.9
49	33.3	100.0	61.0
50	33.3	100.0	60.0
51	33.3	100.0	59.0
52	33.3	100.0	58.1
53	33.3	100.0	57.1
54	33.3	100.0	56.1
55	33.3	100.0	55.1
56	33.3	100.0	54.1
57	33.3	100.0	53.1
58	33.3	100.0	52.1
59	33.3	100.0	51.0
60	33.3	100.0	50.0
61	33.3	100.0	49.0
62	33.3	100.0	47.9
63	33.3	100.0	46.8
64	33.3	100.0	45.8
65	33.3	100.0	44.7
66	33.3	100.0	43.6
67	33.3	100.0	42.5
68	33.3	100.0	41.4
69	33.3	100.0	40.3
70	33.3	100.0	39.1
71	33.3	100.0	38.0
72	33.3	100.0	36.8
73	33.3	100.0	35.7

S-RR

Prevalence of rifampicin resistance	Sensitivity	Specificity	Negative Predictive Value
1	n/a	n/a	n/a
2	n/a	n/a	n/a
3	n/a	n/a	n/a
4	n/a	n/a	n/a
5	n/a	n/a	n/a
6	n/a	n/a	n/a
7	n/a	n/a	n/a
8	n/a	n/a	n/a
9	n/a	n/a	n/a
10	n/a	n/a	n/a
11	n/a	n/a	n/a
12	n/a	n/a	n/a
13	n/a	n/a	n/a
14	n/a	n/a	n/a
15	n/a	n/a	n/a
16	n/a	n/a	n/a
17	n/a	n/a	n/a
18	n/a	n/a	n/a
19	n/a	n/a	n/a
20	n/a	n/a	n/a
21	n/a	n/a	n/a
22	n/a	n/a	n/a
23	n/a	n/a	n/a
24	n/a	n/a	n/a
25	n/a	n/a	n/a
26	n/a	n/a	n/a
27	n/a	n/a	n/a
28	n/a	n/a	n/a
29	n/a	n/a	n/a
30	n/a	n/a	n/a
31	n/a	n/a	n/a
32	n/a	n/a	n/a
33	n/a	n/a	n/a
34	n/a	n/a	n/a
35	n/a	n/a	n/a
36	n/a	n/a	n/a
37	n/a	n/a	n/a
38	n/a	n/a	n/a
39	n/a	n/a	n/a
40	n/a	n/a	n/a
41	n/a	n/a	n/a
42	n/a	n/a	n/a
43	n/a	n/a	n/a
44	n/a	n/a	n/a
45	n/a	n/a	n/a
46	n/a	n/a	n/a
47	n/a	n/a	n/a
48	n/a	n/a	n/a
49	n/a	n/a	n/a
50	n/a	n/a	n/a
51	n/a	n/a	n/a
52	n/a	n/a	n/a
53	n/a	n/a	n/a
54	n/a	n/a	n/a
55	n/a	n/a	n/a
56	n/a	n/a	n/a
57	n/a	n/a	n/a
58	n/a	n/a	n/a
59	n/a	n/a	n/a
60	n/a	n/a	n/a
61	n/a	n/a	n/a
62	n/a	n/a	n/a
63	n/a	n/a	n/a
64	n/a	n/a	n/a
65	n/a	n/a	n/a
66	n/a	n/a	n/a
67	n/a	n/a	n/a
68	n/a	n/a	n/a
69	n/a	n/a	n/a
70	n/a	n/a	n/a
71	n/a	n/a	n/a
72	n/a	n/a	n/a
73	n/a	n/a	n/a

74	82.2	99.4	66.3	74	100.0	90.0	100.0	74	33.3	100.0	34.5	74	n/a	n/a	n/a
75	82.2	99.4	65.1	75	100.0	90.0	100.0	75	33.3	100.0	33.3	75	n/a	n/a	n/a
76	82.2	99.4	63.8	76	100.0	90.0	100.0	76	33.3	100.0	32.1	76	n/a	n/a	n/a
77	82.2	99.4	62.5	77	100.0	90.0	100.0	77	33.3	100.0	30.9	77	n/a	n/a	n/a
78	82.2	99.4	61.2	78	100.0	90.0	100.0	78	33.3	100.0	29.7	78	n/a	n/a	n/a
79	82.2	99.4	59.8	79	100.0	90.0	100.0	79	33.3	100.0	28.5	79	n/a	n/a	n/a
80	82.2	99.4	58.3	80	100.0	90.0	100.0	80	33.3	100.0	27.3	80	n/a	n/a	n/a
81	82.2	99.4	56.7	81	100.0	90.0	100.0	81	33.3	100.0	26.0	81	n/a	n/a	n/a
82	82.2	99.4	55.1	82	100.0	90.0	100.0	82	33.3	100.0	24.8	82	n/a	n/a	n/a
83	82.2	99.4	53.4	83	100.0	90.0	100.0	83	33.3	100.0	23.5	83	n/a	n/a	n/a
84	82.2	99.4	51.6	84	100.0	90.0	100.0	84	33.3	100.0	22.2	84	n/a	n/a	n/a
85	82.2	99.4	49.7	85	100.0	90.0	100.0	85	33.3	100.0	20.9	85	n/a	n/a	n/a
86	82.2	99.4	47.6	86	100.0	90.0	100.0	86	33.3	100.0	19.6	86	n/a	n/a	n/a
87	82.2	99.4	45.5	87	100.0	90.0	100.0	87	33.3	100.0	18.3	87	n/a	n/a	n/a
88	82.2	99.4	43.2	88	100.0	90.0	100.0	88	33.3	100.0	17.0	88	n/a	n/a	n/a
89	82.2	99.4	40.9	89	100.0	90.0	100.0	89	33.3	100.0	15.6	89	n/a	n/a	n/a
90	82.2	99.4	38.3	90	100.0	90.0	100.0	90	33.3	100.0	14.3	90	n/a	n/a	n/a
91	82.2	99.4	35.6	91	100.0	90.0	100.0	91	33.3	100.0	12.9	91	n/a	n/a	n/a
92	82.2	99.4	32.7	92	100.0	90.0	100.0	92	33.3	100.0	11.5	92	n/a	n/a	n/a
93	82.2	99.4	29.6	93	100.0	90.0	100.0	93	33.3	100.0	10.1	93	n/a	n/a	n/a
94	82.2	99.4	26.3	94	100.0	90.0	100.0	94	33.3	100.0	8.7	94	n/a	n/a	n/a
95	82.2	99.4	22.7	95	100.0	90.0	100.0	95	33.3	100.0	7.3	95	n/a	n/a	n/a
96	82.2	99.4	18.9	96	100.0	90.0	100.0	96	33.3	100.0	5.9	96	n/a	n/a	n/a
97	82.2	99.4	14.7	97	100.0	90.0	100.0	97	33.3	100.0	4.4	97	n/a	n/a	n/a
98	82.2	99.4	10.2	98	100.0	90.0	100.0	98	33.3	100.0	3.0	98	n/a	n/a	n/a
99	82.2	99.4	5.3	99	100.0	90.0	100.0	99	33.3	100.0	1.5	99	n/a	n/a	n/a
100	82.2	99.4	0.0	100	100.0	90.0	#DIV/0!	100	33.3	100.0	0.0	100	n/a	n/a	n/a

**R-SS**				**R-RS**				**R-SR**				**R-RR**			
Prevalence of rifampicin resistance	Sensitivity	Specificity	Negative Predictive Value	Prevalence of rifampicin resistance	Sensitivity	Specificity	Negative Predictive Value	Prevalence of rifampicin resistance	Sensitivity	Specificity	Negative Predictive Value	Prevalence of rifampicin resistance	Sensitivity	Specificity	Negative Predictive Value
1	95.9	95.9	100.0	1	98.4	86.2	100.0	1	98.2	74.1	100.0	1	99.7	23.1	100.0
2	95.9	95.9	99.9	2	98.4	86.2	100.0	2	98.2	74.1	100.0	2	99.7	23.1	100.0
3	95.9	95.9	99.9	3	98.4	86.2	99.9	3	98.2	74.1	99.9	3	99.7	23.1	100.0
4	95.9	95.9	99.8	4	98.4	86.2	99.9	4	98.2	74.1	99.9	4	99.7	23.1	99.9
5	95.9	95.9	99.8	5	98.4	86.2	99.9	5	98.2	74.1	99.9	5	99.7	23.1	99.9
6	95.9	95.9	99.7	6	98.4	86.2	99.9	6	98.2	74.1	99.8	6	99.7	23.1	99.9
7	95.9	95.9	99.7	7	98.4	86.2	99.9	7	98.2	74.1	99.8	7	99.7	23.1	99.9
8	95.9	95.9	99.6	8	98.4	86.2	99.8	8	98.2	74.1	99.8	8	99.7	23.1	99.9
9	95.9	95.9	99.6	9	98.4	86.2	99.8	9	98.2	74.1	99.8	9	99.7	23.1	99.9
10	95.9	95.9	99.5	10	98.4	86.2	99.8	10	98.2	74.1	99.7	10	99.7	23.1	99.9
11	95.9	95.9	99.5	11	98.4	86.2	99.8	11	98.2	74.1	99.7	11	99.7	23.1	99.8
12	95.9	95.9	99.4	12	98.4	86.2	99.7	12	98.2	74.1	99.7	12	99.7	23.1	99.8
13	95.9	95.9	99.4	13	98.4	86.2	99.7	13	98.2	74.1	99.6	13	99.7	23.1	99.8
14	95.9	95.9	99.3	14	98.4	86.2	99.7	14	98.2	74.1	99.6	14	99.7	23.1	99.8
15	95.9	95.9	99.2	15	98.4	86.2	99.7	15	98.2	74.1	99.6	15	99.7	23.1	99.8
16	95.9	95.9	99.2	16	98.4	86.2	99.6	16	98.2	74.1	99.5	16	99.7	23.1	99.8
17	95.9	95.9	99.1	17	98.4	86.2	99.6	17	98.2	74.1	99.5	17	99.7	23.1	99.7
18	95.9	95.9	99.1	18	98.4	86.2	99.6	18	98.2	74.1	99.5	18	99.7	23.1	99.7
19	95.9	95.9	99.0	19	98.4	86.2	99.6	19	98.2	74.1	99.4	19	99.7	23.1	99.7
20	95.9	95.9	98.9	20	98.4	86.2	99.5	20	98.2	74.1	99.4	20	99.7	23.1	99.7
21	95.9	95.9	98.9	21	98.4	86.2	99.5	21	98.2	74.1	99.4	21	99.7	23.1	99.7
22	95.9	95.9	98.8	22	98.4	86.2	99.5	22	98.2	74.1	99.3	22	99.7	23.1	99.6
23	95.9	95.9	98.7	23	98.4	86.2	99.4	23	98.2	74.1	99.3	23	99.7	23.1	99.6
24	95.9	95.9	98.7	24	98.4	86.2	99.4	24	98.2	74.1	99.2	24	99.7	23.1	99.6
25	95.9	95.9	98.6	25	98.4	86.2	99.4	25	98.2	74.1	99.2	25	99.7	23.1	99.6
26	95.9	95.9	98.5	26	98.4	86.2	99.3	26	98.2	74.1	99.2	26	99.7	23.1	99.5
27	95.9	95.9	98.4	27	98.4	86.2	99.3	27	98.2	74.1	99.1	27	99.7	23.1	99.5
28	95.9	95.9	98.4	28	98.4	86.2	99.3	28	98.2	74.1	99.1	28	99.7	23.1	99.5
29	95.9	95.9	98.3	29	98.4	86.2	99.2	29	98.2	74.1	99.0	29	99.7	23.1	99.5
30	95.9	95.9	98.2	30	98.4	86.2	99.2	30	98.2	74.1	99.0	30	99.7	23.1	99.4
31	95.9	95.9	98.1	31	98.4	86.2	99.2	31	98.2	74.1	98.9	31	99.7	23.1	99.4
32	95.9	95.9	98.0	32	98.4	86.2	99.1	32	98.2	74.1	98.9	32	99.7	23.1	99.4
33	95.9	95.9	97.9	33	98.4	86.2	99.1	33	98.2	74.1	98.8	33	99.7	23.1	99.4
34	95.9	95.9	97.8	34	98.4	86.2	99.0	34	98.2	74.1	98.8	34	99.7	23.1	99.3
35	95.9	95.9	97.7	35	98.4	86.2	99.0	35	98.2	74.1	98.7	35	99.7	23.1	99.3
36	95.9	95.9	97.6	36	98.4	86.2	99.0	36	98.2	74.1	98.7	36	99.7	23.1	99.3
37	95.9	95.9	97.5	37	98.4	86.2	98.9	37	98.2	74.1	98.6	37	99.7	23.1	99.2
38	95.9	95.9	97.4	38	98.4	86.2	98.9	38	98.2	74.1	98.5	38	99.7	23.1	99.2
39	95.9	95.9	97.3	39	98.4	86.2	98.8	39	98.2	74.1	98.5	39	99.7	23.1	99.2
40	95.9	95.9	97.2	40	98.4	86.2	98.8	40	98.2	74.1	98.4	40	99.7	23.1	99.1
41	95.9	95.9	97.1	41	98.4	86.2	98.7	41	98.2	74.1	98.3	41	99.7	23.1	99.1
42	95.9	95.9	97.0	42	98.4	86.2	98.7	42	98.2	74.1	98.3	42	99.7	23.1	99.1
43	95.9	95.9	96.9	43	98.4	86.2	98.6	43	98.2	74.1	98.2	43	99.7	23.1	99.0
44	95.9	95.9	96.7	44	98.4	86.2	98.5	44	98.2	74.1	98.1	44	99.7	23.1	99.0
45	95.9	95.9	96.6	45	98.4	86.2	98.5	45	98.2	74.1	98.0	45	99.7	23.1	98.9
46	95.9	95.9	96.5	46	98.4	86.2	98.4	46	98.2	74.1	98.0	46	99.7	23.1	98.9
47	95.9	95.9	96.3	47	98.4	86.2	98.4	47	98.2	74.1	97.9	47	99.7	23.1	98.9
48	95.9	95.9	96.2	48	98.4	86.2	98.3	48	98.2	74.1	97.8	48	99.7	23.1	98.8
49	95.9	95.9	96.0	49	98.4	86.2	98.2	49	98.2	74.1	97.7	49	99.7	23.1	98.8
50	95.9	95.9	95.9	50	98.4	86.2	98.2	50	98.2	74.1	97.6	50	99.7	23.1	98.7
51	95.9	95.9	95.7	51	98.4	86.2	98.1	51	98.2	74.1	97.5	51	99.7	23.1	98.7
52	95.9	95.9	95.5	52	98.4	86.2	98.0	52	98.2	74.1	97.4	52	99.7	23.1	98.6
53	95.9	95.9	95.4	53	98.4	86.2	97.9	53	98.2	74.1	97.3	53	99.7	23.1	98.6
54	95.9	95.9	95.2	54	98.4	86.2	97.8	54	98.2	74.1	97.2	54	99.7	23.1	98.5
55	95.9	95.9	95.0	55	98.4	86.2	97.7	55	98.2	74.1	97.1	55	99.7	23.1	98.4
56	95.9	95.9	94.8	56	98.4	86.2	97.7	56	98.2	74.1	97.0	56	99.7	23.1	98.4
57	95.9	95.9	94.6	57	98.4	86.2	97.6	57	98.2	74.1	96.9	57	99.7	23.1	98.3
58	95.9	95.9	94.4	58	98.4	86.2	97.5	58	98.2	74.1	96.8	58	99.7	23.1	98.2
59	95.9	95.9	94.2	59	98.4	86.2	97.4	59	98.2	74.1	96.6	59	99.7	23.1	98.2
60	95.9	95.9	93.9	60	98.4	86.2	97.3	60	98.2	74.1	96.5	60	99.7	23.1	98.1
61	95.9	95.9	93.7	61	98.4	86.2	97.1	61	98.2	74.1	96.3	61	99.7	23.1	98.0
62	95.9	95.9	93.4	62	98.4	86.2	97.0	62	98.2	74.1	96.2	62	99.7	23.1	97.9
63	95.9	95.9	93.2	63	98.4	86.2	96.9	63	98.2	74.1	96.0	63	99.7	23.1	97.8
64	95.9	95.9	92.9	64	98.4	86.2	96.8	64	98.2	74.1	95.9	64	99.7	23.1	97.7

65	95.9	95.9	92.6	65	98.4	86.2	96.6	65	98.2	74.1	95.7	65	99.7	23.1	97.6
66	95.9	95.9	92.3	66	98.4	86.2	96.5	66	98.2	74.1	95.5	66	99.7	23.1	97.5
67	95.9	95.9	92.0	67	98.4	86.2	96.3	67	98.2	74.1	95.3	67	99.7	23.1	97.4
68	95.9	95.9	91.6	68	98.4	86.2	96.2	68	98.2	74.1	95.1	68	99.7	23.1	97.3
69	95.9	95.9	91.3	69	98.4	86.2	96.0	69	98.2	74.1	94.9	69	99.7	23.1	97.2
70	95.9	95.9	90.9	70	98.4	86.2	95.8	70	98.2	74.1	94.6	70	99.7	23.1	97.1
71	95.9	95.9	90.5	71	98.4	86.2	95.6	71	98.2	74.1	94.4	71	99.7	23.1	96.9
72	95.9	95.9	90.0	72	98.4	86.2	95.4	72	98.2	74.1	94.1	72	99.7	23.1	96.8
73	95.9	95.9	89.6	73	98.4	86.2	95.2	73	98.2	74.1	93.8	73	99.7	23.1	96.6
74	95.9	95.9	89.1	74	98.4	86.2	94.9	74	98.2	74.1	93.5	74	99.7	23.1	96.4
75	95.9	95.9	88.6	75	98.4	86.2	94.7	75	98.2	74.1	93.2	75	99.7	23.1	96.2
76	95.9	95.9	88.0	76	98.4	86.2	94.4	76	98.2	74.1	92.8	76	99.7	23.1	96.0
77	95.9	95.9	87.4	77	98.4	86.2	94.1	77	98.2	74.1	92.5	77	99.7	23.1	95.8
78	95.9	95.9	86.8	78	98.4	86.2	93.7	78	98.2	74.1	92.1	78	99.7	23.1	95.6
79	95.9	95.9	86.1	79	98.4	86.2	93.4	79	98.2	74.1	91.6	79	99.7	23.1	95.3
80	95.9	95.9	85.3	80	98.4	86.2	93.0	80	98.2	74.1	91.1	80	99.7	23.1	95.1
81	95.9	95.9	84.5	81	98.4	86.2	92.6	81	98.2	74.1	90.6	81	99.7	23.1	94.8
82	95.9	95.9	83.6	82	98.4	86.2	92.1	82	98.2	74.1	90.0	82	99.7	23.1	94.4
83	95.9	95.9	82.6	83	98.4	86.2	91.6	83	98.2	74.1	89.4	83	99.7	23.1	94.0
84	95.9	95.9	81.6	84	98.4	86.2	91.0	84	98.2	74.1	88.7	84	99.7	23.1	93.6
85	95.9	95.9	80.4	85	98.4	86.2	90.4	85	98.2	74.1	87.9	85	99.7	23.1	93.1
86	95.9	95.9	79.1	86	98.4	86.2	89.6	86	98.2	74.1	87.0	86	99.7	23.1	92.6
87	95.9	95.9	77.6	87	98.4	86.2	88.8	87	98.2	74.1	86.0	87	99.7	23.1	92.0
88	95.9	95.9	76.0	88	98.4	86.2	87.9	88	98.2	74.1	84.9	88	99.7	23.1	91.3
89	95.9	95.9	74.2	89	98.4	86.2	86.8	89	98.2	74.1	83.6	89	99.7	23.1	90.5
90	95.9	95.9	72.1	90	98.4	86.2	85.5	90	98.2	74.1	82.0	90	99.7	23.1	89.5
91	95.9	95.9	69.7	91	98.4	86.2	84.0	91	98.2	74.1	80.3	91	99.7	23.1	88.4
92	95.9	95.9	66.9	92	98.4	86.2	82.2	92	98.2	74.1	78.1	92	99.7	23.1	87.0
93	95.9	95.9	63.6	93	98.4	86.2	80.0	93	98.2	74.1	75.6	93	99.7	23.1	85.3
94	95.9	95.9	59.7	94	98.4	86.2	77.2	94	98.2	74.1	72.4	94	99.7	23.1	83.1
95	95.9	95.9	55.0	95	98.4	86.2	73.6	95	98.2	74.1	68.4	95	99.7	23.1	80.2
96	95.9	95.9	49.2	96	98.4	86.2	68.9	96	98.2	74.1	63.1	96	99.7	23.1	76.2
97	95.9	95.9	41.8	97	98.4	86.2	62.1	97	98.2	74.1	56.0	97	99.7	23.1	70.4
98	95.9	95.9	32.1	98	98.4	86.2	52.0	98	98.2	74.1	45.6	98	99.7	23.1	61.1
99	95.9	95.9	19.0	99	98.4	86.2	34.9	99	98.2	74.1	29.3	99	99.7	23.1	43.7
100	95.9	95.9	0.0	100	98.4	86.2	0.0	100	98.2	74.1	0.0	100	99.7	23.1	0.0

SS-S				RS-S				SR-S				RR-S			
Prevalence of ethambutol resistance	Sensitivity	Specificity	Negative Predictive Value	Prevalence of ethambutol resistance	Sensitivity	Specificity	Negative Predictive Value	Prevalence of ethambutol resistance	Sensitivity	Specificity	Negative Predictive Value	Prevalence of ethambutol resistance	Sensitivity	Specificity	Negative Predictive Value
1	10.0	99.9	99.1	1	80.8	92.4	99.8	1	66.7	98.9	99.7	1	94.9	54.3	99.9
2	10.0	99.9	98.2	2	80.8	92.4	99.6	2	66.7	98.9	99.3	2	94.9	54.3	99.8
3	10.0	99.9	97.3	3	80.8	92.4	99.4	3	66.7	98.9	99.0	3	94.9	54.3	99.7
4	10.0	99.9	96.4	4	80.8	92.4	99.1	4	66.7	98.9	98.6	4	94.9	54.3	99.6
5	10.0	99.9	95.5	5	80.8	92.4	98.9	5	66.7	98.9	98.3	5	94.9	54.3	99.5
6	10.0	99.9	94.6	6	80.8	92.4	98.7	6	66.7	98.9	97.9	6	94.9	54.3	99.4
7	10.0	99.9	93.7	7	80.8	92.4	98.5	7	66.7	98.9	97.5	7	94.9	54.3	99.3
8	10.0	99.9	92.7	8	80.8	92.4	98.2	8	66.7	98.9	97.2	8	94.9	54.3	99.2
9	10.0	99.9	91.8	9	80.8	92.4	98.0	9	66.7	98.9	96.8	9	94.9	54.3	99.1
10	10.0	99.9	90.9	10	80.8	92.4	97.7	10	66.7	98.9	96.4	10	94.9	54.3	99.0
11	10.0	99.9	90.0	11	80.8	92.4	97.5	11	66.7	98.9	96.0	11	94.9	54.3	98.9
12	10.0	99.9	89.1	12	80.8	92.4	97.2	12	66.7	98.9	95.6	12	94.9	54.3	98.7
13	10.0	99.9	88.1	13	80.8	92.4	97.0	13	66.7	98.9	95.2	13	94.9	54.3	98.6
14	10.0	99.9	87.2	14	80.8	92.4	96.7	14	66.7	98.9	94.8	14	94.9	54.3	98.5
15	10.0	99.9	86.3	15	80.8	92.4	96.5	15	66.7	98.9	94.4	15	94.9	54.3	98.4
16	10.0	99.9	85.4	16	80.8	92.4	96.2	16	66.7	98.9	94.0	16	94.9	54.3	98.3
17	10.0	99.9	84.4	17	80.8	92.4	95.9	17	66.7	98.9	93.5	17	94.9	54.3	98.1
18	10.0	99.9	83.5	18	80.8	92.4	95.6	18	66.7	98.9	93.1	18	94.9	54.3	98.0
19	10.0	99.9	82.6	19	80.8	92.4	95.3	19	66.7	98.9	92.7	19	94.9	54.3	97.9
20	10.0	99.9	81.6	20	80.8	92.4	95.1	20	66.7	98.9	92.2	20	94.9	54.3	97.7
21	10.0	99.9	80.7	21	80.8	92.4	94.8	21	66.7	98.9	91.8	21	94.9	54.3	97.6
22	10.0	99.9	79.7	22	80.8	92.4	94.5	22	66.7	98.9	91.3	22	94.9	54.3	97.4
23	10.0	99.9	78.8	23	80.8	92.4	94.1	23	66.7	98.9	90.9	23	94.9	54.3	97.3
24	10.0	99.9	77.9	24	80.8	92.4	93.8	24	66.7	98.9	90.4	24	94.9	54.3	97.1
25	10.0	99.9	76.9	25	80.8	92.4	93.5	25	66.7	98.9	89.9	25	94.9	54.3	97.0
26	10.0	99.9	76.0	26	80.8	92.4	93.2	26	66.7	98.9	89.4	26	94.9	54.3	96.8
27	10.0	99.9	75.0	27	80.8	92.4	92.9	27	66.7	98.9	88.9	27	94.9	54.3	96.7
28	10.0	99.9	74.1	28	80.8	92.4	92.5	28	66.7	98.9	88.4	28	94.9	54.3	96.5
29	10.0	99.9	73.1	29	80.8	92.4	92.2	29	66.7	98.9	87.9	29	94.9	54.3	96.3
30	10.0	99.9	72.1	30	80.8	92.4	91.8	30	66.7	98.9	87.4	30	94.9	54.3	96.2
31	10.0	99.9	71.2	31	80.8	92.4	91.4	31	66.7	98.9	86.9	31	94.9	54.3	96.0
32	10.0	99.9	70.2	32	80.8	92.4	91.1	32	66.7	98.9	86.3	32	94.9	54.3	95.8
33	10.0	99.9	69.3	33	80.8	92.4	90.7	33	66.7	98.9	85.8	33	94.9	54.3	95.6
34	10.0	99.9	68.3	34	80.8	92.4	90.3	34	66.7	98.9	85.2	34	94.9	54.3	95.4
35	10.0	99.9	67.3	35	80.8	92.4	89.9	35	66.7	98.9	84.6	35	94.9	54.3	95.2
36	10.0	99.9	66.4	36	80.8	92.4	89.5	36	66.7	98.9	84.1	36	94.9	54.3	95.0
37	10.0	99.9	65.4	37	80.8	92.4	89.1	37	66.7	98.9	83.5	37	94.9	54.3	94.8
38	10.0	99.9	64.4	38	80.8	92.4	88.7	38	66.7	98.9	82.9	38	94.9	54.3	94.6
39	10.0	99.9	63.5	39	80.8	92.4	88.3	39	66.7	98.9	82.3	39	94.9	54.3	94.4
40	10.0	99.9	62.5	40	80.8	92.4	87.8	40	66.7	98.9	81.7	40	94.9	54.3	94.1
41	10.0	99.9	61.5	41	80.8	92.4	87.4	41	66.7	98.9	81.0	41	94.9	54.3	93.9
42	10.0	99.9	60.5	42	80.8	92.4	86.9	42	66.7	98.9	80.4	42	94.9	54.3	93.7
43	10.0	99.9	59.5	43	80.8	92.4	86.4	43	66.7	98.9	79.7	43	94.9	54.3	93.4
44	10.0	99.9	58.6	44	80.8	92.4	85.9	44	66.7	98.9	79.1	44	94.9	54.3	93.2
45	10.0	99.9	57.6	45	80.8	92.4	85.4	45	66.7	98.9	78.4	45	94.9	54.3	92.9
46	10.0	99.9	56.6	46	80.8	92.4	84.9	46	66.7	98.9	77.7	46	94.9	54.3	92.6
47	10.0	99.9	55.6	47	80.8	92.4	84.4	47	66.7	98.9	77.0	47	94.9	54.3	92.4
48	10.0	99.9	54.6	48	80.8	92.4	83.9	48	66.7	98.9	76.3	48	94.9	54.3	92.1
49	10.0	99.9	53.6	49	80.8	92.4	83.3	49	66.7	98.9	75.5	49	94.9	54.3	91.8
50	10.0	99.9	52.6	50	80.8	92.4	82.8	50	66.7	98.9	74.8	50	94.9	54.3	91.5
51	10.0	99.9	51.6	51	80.8	92.4	82.2	51	66.7	98.9	74.0	51	94.9	54.3	91.2
52	10.0	99.9	50.6	52	80.8	92.4	81.6	52	66.7	98.9	73.3	52	94.9	54.3	90.8
53	10.0	99.9	49.6	53	80.8	92.4	81.0	53	66.7	98.9	72.5	53	94.9	54.3	90.5
54	10.0	99.9	48.6	54	80.8	92.4	80.4	54	66.7	98.9	71.7	54	94.9	54.3	90.1
55	10.0	99.9	47.6	55	80.8	92.4	79.7	55	66.7	98.9	70.8	55	94.9	54.3	89.8

56	10.0	99.9	46.6	56	80.8	92.4	79.1	56	66.7	98.9	70.0	56	94.9	54.3	89.4
57	10.0	99.9	45.6	57	80.8	92.4	78.4	57	66.7	98.9	69.1	57	94.9	54.3	89.0
58	10.0	99.9	44.6	58	80.8	92.4	77.7	58	66.7	98.9	68.2	58	94.9	54.3	88.6
59	10.0	99.9	43.5	59	80.8	92.4	76.9	59	66.7	98.9	67.3	59	94.9	54.3	88.2
60	10.0	99.9	42.5	60	80.8	92.4	76.2	60	66.7	98.9	66.4	60	94.9	54.3	87.7
61	10.0	99.9	41.5	61	80.8	92.4	75.4	61	66.7	98.9	65.5	61	94.9	54.3	87.3
62	10.0	99.9	40.5	62	80.8	92.4	74.6	62	66.7	98.9	64.5	62	94.9	54.3	86.8
63	10.0	99.9	39.5	63	80.8	92.4	73.8	63	66.7	98.9	63.5	63	94.9	54.3	86.3
64	10.0	99.9	38.4	64	80.8	92.4	73.0	64	66.7	98.9	62.5	64	94.9	54.3	85.8
65	10.0	99.9	37.4	65	80.8	92.4	72.1	65	66.7	98.9	61.5	65	94.9	54.3	85.2
66	10.0	99.9	36.4	66	80.8	92.4	71.2	66	66.7	98.9	60.5	66	94.9	54.3	84.7
67	10.0	99.9	35.3	67	80.8	92.4	70.3	67	66.7	98.9	59.4	67	94.9	54.3	84.1
68	10.0	99.9	34.3	68	80.8	92.4	69.3	68	66.7	98.9	58.3	68	94.9	54.3	83.5
69	10.0	99.9	33.3	69	80.8	92.4	68.3	69	66.7	98.9	57.1	69	94.9	54.3	82.8
70	10.0	99.9	32.2	70	80.8	92.4	67.3	70	66.7	98.9	56.0	70	94.9	54.3	82.1
71	10.0	99.9	31.2	71	80.8	92.4	66.2	71	66.7	98.9	54.8	71	94.9	54.3	81.4
72	10.0	99.9	30.2	72	80.8	92.4	65.1	72	66.7	98.9	53.6	72	94.9	54.3	80.7
73	10.0	99.9	29.1	73	80.8	92.4	64.0	73	66.7	98.9	52.3	73	94.9	54.3	79.9
74	10.0	99.9	28.1	74	80.8	92.4	62.8	74	66.7	98.9	51.0	74	94.9	54.3	79.0
75	10.0	99.9	27.0	75	80.8	92.4	61.6	75	66.7	98.9	49.7	75	94.9	54.3	78.1
76	10.0	99.9	26.0	76	80.8	92.4	60.3	76	66.7	98.9	48.4	76	94.9	54.3	77.2
77	10.0	99.9	24.9	77	80.8	92.4	58.9	77	66.7	98.9	47.0	77	94.9	54.3	76.2
78	10.0	99.9	23.8	78	80.8	92.4	57.5	78	66.7	98.9	45.6	78	94.9	54.3	75.1
79	10.0	99.9	22.8	79	80.8	92.4	56.1	79	66.7	98.9	44.1	79	94.9	54.3	74.0
80	10.0	99.9	21.7	80	80.8	92.4	54.6	80	66.7	98.9	42.6	80	94.9	54.3	72.8
81	10.0	99.9	20.7	81	80.8	92.4	53.0	81	66.7	98.9	41.0	81	94.9	54.3	71.5
82	10.0	99.9	19.6	82	80.8	92.4	51.3	82	66.7	98.9	39.5	82	94.9	54.3	70.2
83	10.0	99.9	18.5	83	80.8	92.4	49.6	83	66.7	98.9	37.8	83	94.9	54.3	68.7
84	10.0	99.9	17.5	84	80.8	92.4	47.8	84	66.7	98.9	36.1	84	94.9	54.3	67.1
85	10.0	99.9	16.4	85	80.8	92.4	45.9	85	66.7	98.9	34.4	85	94.9	54.3	65.4
86	10.0	99.9	15.3	86	80.8	92.4	43.9	86	66.7	98.9	32.6	86	94.9	54.3	63.6
87	10.0	99.9	14.2	87	80.8	92.4	41.8	87	66.7	98.9	30.7	87	94.9	54.3	61.6
88	10.0	99.9	13.1	88	80.8	92.4	39.6	88	66.7	98.9	28.8	88	94.9	54.3	59.4
89	10.0	99.9	12.1	89	80.8	92.4	37.3	89	66.7	98.9	26.8	89	94.9	54.3	57.0
90	10.0	99.9	11.0	90	80.8	92.4	34.8	90	66.7	98.9	24.8	90	94.9	54.3	54.4
91	10.0	99.9	9.9	91	80.8	92.4	32.2	91	66.7	98.9	22.7	91	94.9	54.3	51.5
92	10.0	99.9	8.8	92	80.8	92.4	29.5	92	66.7	98.9	20.5	92	94.9	54.3	48.2
93	10.0	99.9	7.7	93	80.8	92.4	26.6	93	66.7	98.9	18.3	93	94.9	54.3	44.7
94	10.0	99.9	6.6	94	80.8	92.4	23.5	94	66.7	98.9	15.9	94	94.9	54.3	40.6
95	10.0	99.9	5.5	95	80.8	92.4	20.2	95	66.7	98.9	13.5	95	94.9	54.3	36.1
96	10.0	99.9	4.4	96	80.8	92.4	16.7	96	66.7	98.9	11.0	96	94.9	54.3	30.9
97	10.0	99.9	3.3	97	80.8	92.4	12.9	97	66.7	98.9	8.4	97	94.9	54.3	24.9
98	10.0	99.9	2.2	98	80.8	92.4	8.9	98	66.7	98.9	5.7	98	94.9	54.3	18.0
99	10.0	99.9	1.1	99	80.8	92.4	4.6	99	66.7	98.9	2.9	99	94.9	54.3	9.8
100	10.0	99.9	0.0	100	80.8	92.4	0.0	100	66.7	98.9	0.0	100	94.9	54.3	0.0

SS-R				RS-R				SR-R				RR-R			
Prevalence of ethambutol resistance	Sensitivity	Specificity	Negative Predictive Value	Prevalence of ethambutol resistance	Sensitivity	Specificity	Negative Predictive Value	Prevalence of ethambutol resistance	Sensitivity	Specificity	Negative Predictive Value	Prevalence of ethambutol resistance	Sensitivity	Specificity	Negative Predictive Value
1	n/a	98.8	n/a	1	85.7	74.1	99.8	1	n/a	100.0	n/a	1	98.6	25.0	99.9
2	n/a	98.8	n/a	2	85.7	74.1	99.6	2	n/a	100.0	n/a	2	98.6	25.0	99.9
3	n/a	98.8	n/a	3	85.7	74.1	99.4	3	n/a	100.0	n/a	3	98.6	25.0	99.8
4	n/a	98.8	n/a	4	85.7	74.1	99.2	4	n/a	100.0	n/a	4	98.6	25.0	99.8
5	n/a	98.8	n/a	5	85.7	74.1	99.0	5	n/a	100.0	n/a	5	98.6	25.0	99.7
6	n/a	98.8	n/a	6	85.7	74.1	98.8	6	n/a	100.0	n/a	6	98.6	25.0	99.6
7	n/a	98.8	n/a	7	85.7	74.1	98.6	7	n/a	100.0	n/a	7	98.6	25.0	99.6
8	n/a	98.8	n/a	8	85.7	74.1	98.4	8	n/a	100.0	n/a	8	98.6	25.0	99.5
9	n/a	98.8	n/a	9	85.7	74.1	98.1	9	n/a	100.0	n/a	9	98.6	25.0	99.4
10	n/a	98.8	n/a	10	85.7	74.1	97.9	10	n/a	100.0	n/a	10	98.6	25.0	99.4
11	n/a	98.8	n/a	11	85.7	74.1	97.7	11	n/a	100.0	n/a	11	98.6	25.0	99.3
12	n/a	98.8	n/a	12	85.7	74.1	97.4	12	n/a	100.0	n/a	12	98.6	25.0	99.2
13	n/a	98.8	n/a	13	85.7	74.1	97.2	13	n/a	100.0	n/a	13	98.6	25.0	99.2
14	n/a	98.8	n/a	14	85.7	74.1	97.0	14	n/a	100.0	n/a	14	98.6	25.0	99.1
15	n/a	98.8	n/a	15	85.7	74.1	96.7	15	n/a	100.0	n/a	15	98.6	25.0	99.0
16	n/a	98.8	n/a	16	85.7	74.1	96.5	16	n/a	100.0	n/a	16	98.6	25.0	98.9
17	n/a	98.8	n/a	17	85.7	74.1	96.2	17	n/a	100.0	n/a	17	98.6	25.0	98.9
18	n/a	98.8	n/a	18	85.7	74.1	95.9	18	n/a	100.0	n/a	18	98.6	25.0	98.8
19	n/a	98.8	n/a	19	85.7	74.1	95.7	19	n/a	100.0	n/a	19	98.6	25.0	98.7
20	n/a	98.8	n/a	20	85.7	74.1	95.4	20	n/a	100.0	n/a	20	98.6	25.0	98.6
21	n/a	98.8	n/a	21	85.7	74.1	95.1	21	n/a	100.0	n/a	21	98.6	25.0	98.5
22	n/a	98.8	n/a	22	85.7	74.1	94.8	22	n/a	100.0	n/a	22	98.6	25.0	98.4
23	n/a	98.8	n/a	23	85.7	74.1	94.6	23	n/a	100.0	n/a	23	98.6	25.0	98.3
24	n/a	98.8	n/a	24	85.7	74.1	94.3	24	n/a	100.0	n/a	24	98.6	25.0	98.2
25	n/a	98.8	n/a	25	85.7	74.1	94.0	25	n/a	100.0	n/a	25	98.6	25.0	98.1
26	n/a	98.8	n/a	26	85.7	74.1	93.7	26	n/a	100.0	n/a	26	98.6	25.0	98.0
27	n/a	98.8	n/a	27	85.7	74.1	93.3	27	n/a	100.0	n/a	27	98.6	25.0	97.9
28	n/a	98.8	n/a	28	85.7	74.1	93.0	28	n/a	100.0	n/a	28	98.6	25.0	97.8
29	n/a	98.8	n/a	29	85.7	74.1	92.7	29	n/a	100.0	n/a	29	98.6	25.0	97.7
30	n/a	98.8	n/a	30	85.7	74.1	92.4	30	n/a	100.0	n/a	30	98.6	25.0	97.6
31	n/a	98.8	n/a	31	85.7	74.1	92.0	31	n/a	100.0	n/a	31	98.6	25.0	97.5
32	n/a	98.8	n/a	32	85.7	74.1	91.7	32	n/a	100.0	n/a	32	98.6	25.0	97.4
33	n/a	98.8	n/a	33	85.7	74.1	91.3	33	n/a	100.0	n/a	33	98.6	25.0	97.3
34	n/a	98.8	n/a	34	85.7	74.1	91.0	34	n/a	100.0	n/a	34	98.6	25.0	97.2
35	n/a	98.8	n/a	35	85.7	74.1	90.6	35	n/a	100.0	n/a	35	98.6	25.0	97.0
36	n/a	98.8	n/a	36	85.7	74.1	90.2	36	n/a	100.0	n/a	36	98.6	25.0	96.9
37	n/a	98.8	n/a	37	85.7	74.1	89.8	37	n/a	100.0	n/a	37	98.6	25.0	96.8
38	n/a	98.8	n/a	38	85.7	74.1	89.4	38	n/a	100.0	n/a	38	98.6	25.0	96.6
39	n/a	98.8	n/a	39	85.7	74.1	89.0	39	n/a	100.0	n/a	39	98.6	25.0	96.5
40	n/a	98.8	n/a	40	85.7	74.1	88.6	40	n/a	100.0	n/a	40	98.6	25.0	96.4
41	n/a	98.8	n/a	41	85.7	74.1	88.2	41	n/a	100.0	n/a	41	98.6	25.0	96.2
42	n/a	98.8	n/a	42	85.7	74.1	87.7	42	n/a	100.0	n/a	42	98.6	25.0	96.1
43	n/a	98.8	n/a	43	85.7	74.1	87.3	43	n/a	100.0	n/a	43	98.6	25.0	95.9
44	n/a	98.8	n/a	44	85.7	74.1	86.8	44	n/a	100.0	n/a	44	98.6	25.0	95.7
45	n/a	98.8	n/a	45	85.7	74.1	86.4	45	n/a	100.0	n/a	45	98.6	25.0	95.6
46	n/a	98.8	n/a	46	85.7	74.1	85.9	46	n/a	100.0	n/a	46	98.6	25.0	95.4

47	n/a	98.8	n/a	47	85.7	74.1	85.4	47	n/a	100.0	n/a	47	98.6	25.0	95.2
48	n/a	98.8	n/a	48	85.7	74.1	84.9	48	n/a	100.0	n/a	48	98.6	25.0	95.0
49	n/a	98.8	n/a	49	85.7	74.1	84.4	49	n/a	100.0	n/a	49	98.6	25.0	94.8
50	n/a	98.8	n/a	50	85.7	74.1	83.8	50	n/a	100.0	n/a	50	98.6	25.0	94.6
51	n/a	98.8	n/a	51	85.7	74.1	83.3	51	n/a	100.0	n/a	51	98.6	25.0	94.4
52	n/a	98.8	n/a	52	85.7	74.1	82.7	52	n/a	100.0	n/a	52	98.6	25.0	94.2
53	n/a	98.8	n/a	53	85.7	74.1	82.1	53	n/a	100.0	n/a	53	98.6	25.0	94.0
54	n/a	98.8	n/a	54	85.7	74.1	81.5	54	n/a	100.0	n/a	54	98.6	25.0	93.8
55	n/a	98.8	n/a	55	85.7	74.1	80.9	55	n/a	100.0	n/a	55	98.6	25.0	93.5
56	n/a	98.8	n/a	56	85.7	74.1	80.3	56	n/a	100.0	n/a	56	98.6	25.0	93.3
57	n/a	98.8	n/a	57	85.7	74.1	79.6	57	n/a	100.0	n/a	57	98.6	25.0	93.0
58	n/a	98.8	n/a	58	85.7	74.1	79.0	58	n/a	100.0	n/a	58	98.6	25.0	92.7
59	n/a	98.8	n/a	59	85.7	74.1	78.3	59	n/a	100.0	n/a	59	98.6	25.0	92.4
60	n/a	98.8	n/a	60	85.7	74.1	77.6	60	n/a	100.0	n/a	60	98.6	25.0	92.2
61	n/a	98.8	n/a	61	85.7	74.1	76.8	61	n/a	100.0	n/a	61	98.6	25.0	91.8
62	n/a	98.8	n/a	62	85.7	74.1	76.1	62	n/a	100.0	n/a	62	98.6	25.0	91.5
63	n/a	98.8	n/a	63	85.7	74.1	75.3	63	n/a	100.0	n/a	63	98.6	25.0	91.2
64	n/a	98.8	n/a	64	85.7	74.1	74.5	64	n/a	100.0	n/a	64	98.6	25.0	90.8
65	n/a	98.8	n/a	65	85.7	74.1	73.6	65	n/a	100.0	n/a	65	98.6	25.0	90.5
66	n/a	98.8	n/a	66	85.7	74.1	72.8	66	n/a	100.0	n/a	66	98.6	25.0	90.1
67	n/a	98.8	n/a	67	85.7	74.1	71.9	67	n/a	100.0	n/a	67	98.6	25.0	89.7
68	n/a	98.8	n/a	68	85.7	74.1	70.9	68	n/a	100.0	n/a	68	98.6	25.0	89.2
69	n/a	98.8	n/a	69	85.7	74.1	70.0	69	n/a	100.0	n/a	69	98.6	25.0	88.8
70	n/a	98.8	n/a	70	85.7	74.1	69.0	70	n/a	100.0	n/a	70	98.6	25.0	88.3
71	n/a	98.8	n/a	71	85.7	74.1	67.9	71	n/a	100.0	n/a	71	98.6	25.0	87.8
72	n/a	98.8	n/a	72	85.7	74.1	66.8	72	n/a	100.0	n/a	72	98.6	25.0	87.3
73	n/a	98.8	n/a	73	85.7	74.1	65.7	73	n/a	100.0	n/a	73	98.6	25.0	86.7
74	n/a	98.8	n/a	74	85.7	74.1	64.6	74	n/a	100.0	n/a	74	98.6	25.0	86.1
75	n/a	98.8	n/a	75	85.7	74.1	63.3	75	n/a	100.0	n/a	75	98.6	25.0	85.4
76	n/a	98.8	n/a	76	85.7	74.1	62.1	76	n/a	100.0	n/a	76	98.6	25.0	84.8
77	n/a	98.8	n/a	77	85.7	74.1	60.8	77	n/a	100.0	n/a	77	98.6	25.0	84.0
78	n/a	98.8	n/a	78	85.7	74.1	59.4	78	n/a	100.0	n/a	78	98.6	25.0	83.2
79	n/a	98.8	n/a	79	85.7	74.1	58.0	79	n/a	100.0	n/a	79	98.6	25.0	82.4
80	n/a	98.8	n/a	80	85.7	74.1	56.5	80	n/a	100.0	n/a	80	98.6	25.0	81.5
81	n/a	98.8	n/a	81	85.7	74.1	54.9	81	n/a	100.0	n/a	81	98.6	25.0	80.5
82	n/a	98.8	n/a	82	85.7	74.1	53.2	82	n/a	100.0	n/a	82	98.6	25.0	79.4
83	n/a	98.8	n/a	83	85.7	74.1	51.5	83	n/a	100.0	n/a	83	98.6	25.0	78.3
84	n/a	98.8	n/a	84	85.7	74.1	49.7	84	n/a	100.0	n/a	84	98.6	25.0	77.0
85	n/a	98.8	n/a	85	85.7	74.1	47.8	85	n/a	100.0	n/a	85	98.6	25.0	75.7
86	n/a	98.8	n/a	86	85.7	74.1	45.8	86	n/a	100.0	n/a	86	98.6	25.0	74.1
87	n/a	98.8	n/a	87	85.7	74.1	43.7	87	n/a	100.0	n/a	87	98.6	25.0	72.5
88	n/a	98.8	n/a	88	85.7	74.1	41.4	88	n/a	100.0	n/a	88	98.6	25.0	70.6
89	n/a	98.8	n/a	89	85.7	74.1	39.1	89	n/a	100.0	n/a	89	98.6	25.0	68.5
90	n/a	98.8	n/a	90	85.7	74.1	36.6	90	n/a	100.0	n/a	90	98.6	25.0	66.2
91	n/a	98.8	n/a	91	85.7	74.1	33.9	91	n/a	100.0	n/a	91	98.6	25.0	63.5
92	n/a	98.8	n/a	92	85.7	74.1	31.1	92	n/a	100.0	n/a	92	98.6	25.0	60.5
93	n/a	98.8	n/a	93	85.7	74.1	28.1	93	n/a	100.0	n/a	93	98.6	25.0	57.0
94	n/a	98.8	n/a	94	85.7	74.1	24.9	94	n/a	100.0	n/a	94	98.6	25.0	52.9
95	n/a	98.8	n/a	95	85.7	74.1	21.4	95	n/a	100.0	n/a	95	98.6	25.0	48.1
96	n/a	98.8	n/a	96	85.7	74.1	17.8	96	n/a	100.0	n/a	96	98.6	25.0	42.3
97	n/a	98.8	n/a	97	85.7	74.1	13.8	97	n/a	100.0	n/a	97	98.6	25.0	35.3
98	n/a	98.8	n/a	98	85.7	74.1	9.6	98	n/a	100.0	n/a	98	98.6	25.0	26.4
99	n/a	98.8	n/a	99	85.7	74.1	5.0	99	n/a	100.0	n/a	99	98.6	25.0	15.1
100	n/a	98.8	n/a	100	85.7	74.1	0.0	100	n/a	100.0	n/a	100	98.6	25.0	0.0

SSS-				RSS-				RRS-				SRS-			
Prevalence of pyrazinamide resistance	Sensitivity	Specificity	Negative Predictive Value	Prevalence of pyrazinamide resistance	Sensitivity	Specificity	Negative Predictive Value	Prevalence of pyrazinamide resistance	Sensitivity	Specificity	Negative Predictive Value	Prevalence of pyrazinamide resistance	Sensitivity	Specificity	Negative Predictive Value
1	72.5	99.8	99.7	1	61.9	98.9	99.6	1	86.9	88.4	99.9	1	0.0	100.0	99.0
2	72.5	99.8	99.4	2	61.9	98.9	99.2	2	86.9	88.4	99.7	2	0.0	100.0	98.0
3	72.5	99.8	99.2	3	61.9	98.9	98.8	3	86.9	88.4	99.5	3	0.0	100.0	97.0
4	72.5	99.8	98.9	4	61.9	98.9	98.4	4	86.9	88.4	99.4	4	0.0	100.0	96.0
5	72.5	99.8	98.6	5	61.9	98.9	98.0	5	86.9	88.4	99.2	5	0.0	100.0	95.0
6	72.5	99.8	98.3	6	61.9	98.9	97.6	6	86.9	88.4	99.1	6	0.0	100.0	94.0
7	72.5	99.8	98.0	7	61.9	98.9	97.2	7	86.9	88.4	98.9	7	0.0	100.0	93.0
8	72.5	99.8	97.7	8	61.9	98.9	96.8	8	86.9	88.4	98.7	8	0.0	100.0	92.0
9	72.5	99.8	97.4	9	61.9	98.9	96.3	9	86.9	88.4	98.6	9	0.0	100.0	91.0
10	72.5	99.8	97.0	10	61.9	98.9	95.9	10	86.9	88.4	98.4	10	0.0	100.0	90.0
11	72.5	99.8	96.7	11	61.9	98.9	95.5	11	86.9	88.4	98.2	11	0.0	100.0	89.0
12	72.5	99.8	96.4	12	61.9	98.9	95.0	12	86.9	88.4	98.0	12	0.0	100.0	88.0
13	72.5	99.8	96.1	13	61.9	98.9	94.6	13	86.9	88.4	97.8	13	0.0	100.0	87.0
14	72.5	99.8	95.7	14	61.9	98.9	94.1	14	86.9	88.4	97.6	14	0.0	100.0	86.0
15	72.5	99.8	95.4	15	61.9	98.9	93.6	15	86.9	88.4	97.5	15	0.0	100.0	85.0
16	72.5	99.8	95.0	16	61.9	98.9	93.2	16	86.9	88.4	97.3	16	0.0	100.0	84.0
17	72.5	99.8	94.7	17	61.9	98.9	92.7	17	86.9	88.4	97.1	17	0.0	100.0	83.0
18	72.5	99.8	94.3	18	61.9	98.9	92.2	18	86.9	88.4	96.9	18	0.0	100.0	82.0
19	72.5	99.8	93.9	19	61.9	98.9	91.7	19	86.9	88.4	96.6	19	0.0	100.0	81.0
20	72.5	99.8	93.6	20	61.9	98.9	91.2	20	86.9	88.4	96.4	20	0.0	100.0	80.0
21	72.5	99.8	93.2	21	61.9	98.9	90.7	21	86.9	88.4	96.2	21	0.0	100.0	79.0
22	72.5	99.8	92.8	22	61.9	98.9	90.2	22	86.9	88.4	96.0	22	0.0	100.0	78.0
23	72.5	99.8	92.4	23	61.9	98.9	89.7	23	86.9	88.4	95.8	23	0.0	100.0	77.0
24	72.5	99.8	92.0	24	61.9	98.9	89.2	24	86.9	88.4	95.5	24	0.0	100.0	76.0
25	72.5	99.8	91.6	25	61.9	98.9	88.6	25	86.9	88.4	95.3	25	0.0	100.0	75.0
26	72.5	99.8	91.2	26	61.9	98.9	88.1	26	86.9	88.4	95.1	26	0.0	100.0	74.0
27	72.5	99.8	90.8	27	61.9	98.9	87.5	27	86.9	88.4	94.8	27	0.0	100.0	73.0
28	72.5	99.8	90.3	28	61.9	98.9	87.0	28	86.9	88.4	94.6	28	0.0	100.0	72.0
29	72.5	99.8	89.9	29	61.9	98.9	86.4	29	86.9	88.4	94.3	29	0.0	100.0	71.0
30	72.5	99.8	89.5	30	61.9	98.9	85.8	30	86.9	88.4	94.0	30	0.0	100.0	70.0
31	72.5	99.8	89.0	31	61.9	98.9	85.2	31	86.9	88.4	93.8	31	0.0	100.0	69.0
32	72.5	99.8	88.5	32	61.9	98.9	84.6	32	86.9	88.4	93.5	32	0.0	100.0	68.0
33	72.5	99.8	88.1	33	61.9	98.9	84.0	33	86.9	88.4	93.2	33	0.0	100.0	67.0
34	72.5	99.8	87.6	34	61.9	98.9	83.4	34	86.9	88.4	92.9	34	0.0	100.0	66.0
35	72.5	99.8	87.1	35	61.9	98.9	82.8	35	86.9	88.4	92.6	35	0.0	100.0	65.0
36	72.5	99.8	86.6	36	61.9	98.9	82.2	36	86.9	88.4	92.3	36	0.0	100.0	64.0

37	72.5	99.8	86.1	37	61.9	98.9	81.5	37	86.9	88.4	92.0	37	0.0	100.0	63.0
38	72.5	99.8	85.6	38	61.9	98.9	80.9	38	86.9	88.4	91.7	38	0.0	100.0	62.0
39	72.5	99.8	85.0	39	61.9	98.9	80.2	39	86.9	88.4	91.4	39	0.0	100.0	61.0
40	72.5	99.8	84.5	40	61.9	98.9	79.6	40	86.9	88.4	91.0	40	0.0	100.0	60.0
41	72.5	99.8	83.9	41	61.9	98.9	78.9	41	86.9	88.4	90.7	41	0.0	100.0	59.0
42	72.5	99.8	83.4	42	61.9	98.9	78.2	42	86.9	88.4	90.3	42	0.0	100.0	58.0
43	72.5	99.8	82.8	43	61.9	98.9	77.5	43	86.9	88.4	90.0	43	0.0	100.0	57.0
44	72.5	99.8	82.2	44	61.9	98.9	76.8	44	86.9	88.4	89.6	44	0.0	100.0	56.0
45	72.5	99.8	81.6	45	61.9	98.9	76.0	45	86.9	88.4	89.2	45	0.0	100.0	55.0
46	72.5	99.8	81.0	46	61.9	98.9	75.3	46	86.9	88.4	88.8	46	0.0	100.0	54.0
47	72.5	99.8	80.4	47	61.9	98.9	74.5	47	86.9	88.4	88.4	47	0.0	100.0	53.0
48	72.5	99.8	79.7	48	61.9	98.9	73.8	48	86.9	88.4	88.0	48	0.0	100.0	52.0
49	72.5	99.8	79.1	49	61.9	98.9	73.0	49	86.9	88.4	87.5	49	0.0	100.0	51.0
50	72.5	99.8	78.4	50	61.9	98.9	72.2	50	86.9	88.4	87.1	50	0.0	100.0	50.0
51	72.5	99.8	77.7	51	61.9	98.9	71.4	51	86.9	88.4	86.6	51	0.0	100.0	49.0
52	72.5	99.8	77.0	52	61.9	98.9	70.5	52	86.9	88.4	86.2	52	0.0	100.0	48.0
53	72.5	99.8	76.3	53	61.9	98.9	69.7	53	86.9	88.4	85.7	53	0.0	100.0	47.0
54	72.5	99.8	75.6	54	61.9	98.9	68.9	54	86.9	88.4	85.2	54	0.0	100.0	46.0
55	72.5	99.8	74.8	55	61.9	98.9	68.0	55	86.9	88.4	84.7	55	0.0	100.0	45.0
56	72.5	99.8	74.1	56	61.9	98.9	67.1	56	86.9	88.4	84.1	56	0.0	100.0	44.0
57	72.5	99.8	73.3	57	61.9	98.9	66.2	57	86.9	88.4	83.6	57	0.0	100.0	43.0
58	72.5	99.8	72.5	58	61.9	98.9	65.3	58	86.9	88.4	83.0	58	0.0	100.0	42.0
59	72.5	99.8	71.6	59	61.9	98.9	64.3	59	86.9	88.4	82.4	59	0.0	100.0	41.0
60	72.5	99.8	70.8	60	61.9	98.9	63.4	60	86.9	88.4	81.8	60	0.0	100.0	40.0
61	72.5	99.8	69.9	61	61.9	98.9	62.4	61	86.9	88.4	81.2	61	0.0	100.0	39.0
62	72.5	99.8	69.0	62	61.9	98.9	61.4	62	86.9	88.4	80.5	62	0.0	100.0	38.0
63	72.5	99.8	68.1	63	61.9	98.9	60.4	63	86.9	88.4	79.9	63	0.0	100.0	37.0
64	72.5	99.8	67.1	64	61.9	98.9	59.3	64	86.9	88.4	79.2	64	0.0	100.0	36.0
65	72.5	99.8	66.2	65	61.9	98.9	58.3	65	86.9	88.4	78.4	65	0.0	100.0	35.0
66	72.5	99.8	65.2	66	61.9	98.9	57.2	66	86.9	88.4	77.7	66	0.0	100.0	34.0
67	72.5	99.8	64.2	67	61.9	98.9	56.1	67	86.9	88.4	76.9	67	0.0	100.0	33.0
68	72.5	99.8	63.1	68	61.9	98.9	55.0	68	86.9	88.4	76.1	68	0.0	100.0	32.0
69	72.5	99.8	62.0	69	61.9	98.9	53.8	69	86.9	88.4	75.2	69	0.0	100.0	31.0
70	72.5	99.8	60.9	70	61.9	98.9	52.7	70	86.9	88.4	74.3	70	0.0	100.0	30.0
71	72.5	99.8	59.7	71	61.9	98.9	51.5	71	86.9	88.4	73.4	71	0.0	100.0	29.0
72	72.5	99.8	58.6	72	61.9	98.9	50.2	72	86.9	88.4	72.4	72	0.0	100.0	28.0
73	72.5	99.8	57.3	73	61.9	98.9	49.0	73	86.9	88.4	71.4	73	0.0	100.0	27.0
74	72.5	99.8	56.1	74	61.9	98.9	47.7	74	86.9	88.4	70.4	74	0.0	100.0	26.0
75	72.5	99.8	54.8	75	61.9	98.9	46.4	75	86.9	88.4	69.2	75	0.0	100.0	25.0
76	72.5	99.8	53.4	76	61.9	98.9	45.0	76	86.9	88.4	68.1	76	0.0	100.0	24.0
77	72.5	99.8	52.0	77	61.9	98.9	43.7	77	86.9	88.4	66.9	77	0.0	100.0	23.0
78	72.5	99.8	50.6	78	61.9	98.9	42.3	78	86.9	88.4	65.6	78	0.0	100.0	22.0
79	72.5	99.8	49.1	79	61.9	98.9	40.8	79	86.9	88.4	64.2	79	0.0	100.0	21.0
80	72.5	99.8	47.6	80	61.9	98.9	39.3	80	86.9	88.4	62.8	80	0.0	100.0	20.0
81	72.5	99.8	46.0	81	61.9	98.9	37.8	81	86.9	88.4	61.3	81	0.0	100.0	19.0
82	72.5	99.8	44.4	82	61.9	98.9	36.3	82	86.9	88.4	59.7	82	0.0	100.0	18.0
83	72.5	99.8	42.7	83	61.9	98.9	34.7	83	86.9	88.4	58.0	83	0.0	100.0	17.0
84	72.5	99.8	40.9	84	61.9	98.9	33.1	84	86.9	88.4	56.3	84	0.0	100.0	16.0
85	72.5	99.8	39.1	85	61.9	98.9	31.4	85	86.9	88.4	54.4	85	0.0	100.0	15.0
86	72.5	99.8	37.2	86	61.9	98.9	29.7	86	86.9	88.4	52.4	86	0.0	100.0	14.0
87	72.5	99.8	35.2	87	61.9	98.9	27.9	87	86.9	88.4	50.2	87	0.0	100.0	13.0
88	72.5	99.8	33.1	88	61.9	98.9	26.1	88	86.9	88.4	47.9	88	0.0	100.0	12.0
89	72.5	99.8	31.0	89	61.9	98.9	24.3	89	86.9	88.4	45.5	89	0.0	100.0	11.0
90	72.5	99.8	28.8	90	61.9	98.9	22.4	90	86.9	88.4	42.9	90	0.0	100.0	10.0
91	72.5	99.8	26.4	91	61.9	98.9	20.4	91	86.9	88.4	40.1	91	0.0	100.0	9.0
92	72.5	99.8	24.0	92	61.9	98.9	18.4	92	86.9	88.4	37.0	92	0.0	100.0	8.0
93	72.5	99.8	21.5	93	61.9	98.9	16.3	93	86.9	88.4	33.7	93	0.0	100.0	7.0
94	72.5	99.8	18.8	94	61.9	98.9	14.2	94	86.9	88.4	30.1	94	0.0	100.0	6.0
95	72.5	99.8	16.1	95	61.9	98.9	12.0	95	86.9	88.4	26.2	95	0.0	100.0	5.0
96	72.5	99.8	13.1	96	61.9	98.9	9.8	96	86.9	88.4	22.0	96	0.0	100.0	4.0
97	72.5	99.8	10.1	97	61.9	98.9	7.4	97	86.9	88.4	17.3	97	0.0	100.0	3.0
98	72.5	99.8	6.9	98	61.9	98.9	5.0	98	86.9	88.4	12.1	98	0.0	100.0	2.0
99	72.5	99.8	3.5	99	61.9	98.9	2.6	99	86.9	88.4	6.4	99	0.0	100.0	1.0
100	72.5	99.8	0.0	100	61.9	98.9	0.0	100	86.9	88.4	0.0	100	0.0	100.0	0.0

RRR-				SRR-				RSR-				SSR-			
Prevalence of pyrazinamide resistance	Sensitivity	Specificity	Negative Predictive Value	Prevalence of pyrazinamide resistance	Sensitivity	Specificity	Negative Predictive Value	Prevalence of pyrazinamide resistance	Sensitivity	Specificity	Negative Predictive Value	Prevalence of pyrazinamide resistance	Sensitivity	Specificity	Negative Predictive Value
1	97.3	66.9	100.0	1	n/a	100.0	n/a	1	83.3	100.0	99.8	1	n/a	100.0	n/a
2	97.3	66.9	99.9	2	n/a	100.0	n/a	2	83.3	100.0	99.7	2	n/a	100.0	n/a
3	97.3	66.9	99.9	3	n/a	100.0	n/a	3	83.3	100.0	99.5	3	n/a	100.0	n/a
4	97.3	66.9	99.8	4	n/a	100.0	n/a	4	83.3	100.0	99.3	4	n/a	100.0	n/a
5	97.3	66.9	99.8	5	n/a	100.0	n/a	5	83.3	100.0	99.1	5	n/a	100.0	n/a
6	97.3	66.9	99.7	6	n/a	100.0	n/a	6	83.3	100.0	98.9	6	n/a	100.0	n/a
7	97.3	66.9	99.7	7	n/a	100.0	n/a	7	83.3	100.0	98.8	7	n/a	100.0	n/a
8	97.3	66.9	99.6	8	n/a	100.0	n/a	8	83.3	100.0	98.6	8	n/a	100.0	n/a
9	97.3	66.9	99.6	9	n/a	100.0	n/a	9	83.3	100.0	98.4	9	n/a	100.0	n/a
10	97.3	66.9	99.5	10	n/a	100.0	n/a	10	83.3	100.0	98.2	10	n/a	100.0	n/a
11	97.3	66.9	99.5	11	n/a	100.0	n/a	11	83.3	100.0	98.0	11	n/a	100.0	n/a
12	97.3	66.9	99.4	12	n/a	100.0	n/a	12	83.3	100.0	97.8	12	n/a	100.0	n/a
13	97.3	66.9	99.4	13	n/a	100.0	n/a	13	83.3	100.0	97.6	13	n/a	100.0	n/a
14	97.3	66.9	99.3	14	n/a	100.0	n/a	14	83.3	100.0	97.4	14	n/a	100.0	n/a
15	97.3	66.9	99.3	15	n/a	100.0	n/a	15	83.3	100.0	97.1	15	n/a	100.0	n/a
16	97.3	66.9	99.2	16	n/a	100.0	n/a	16	83.3	100.0	96.9	16	n/a	100.0	n/a
17	97.3	66.9	99.2	17	n/a	100.0	n/a	17	83.3	100.0	96.7	17	n/a	100.0	n/a
18	97.3	66.9	99.1	18	n/a	100.0	n/a	18	83.3	100.0	96.5	18	n/a	100.0	n/a
19	97.3	66.9	99.0	19	n/a	100.0	n/a	19	83.3	100.0	96.2	19	n/a	100.0	n/a
20	97.3	66.9	99.0	20	n/a	100.0	n/a	20	83.3	100.0	96.0	20	n/a	100.0	n/a
21	97.3	66.9	98.9	21	n/a	100.0	n/a	21	83.3	100.0	95.8	21	n/a	100.0	n/a
22	97.3	66.9	98.9	22	n/a	100.0	n/a	22	83.3	100.0	95.5	22	n/a	100.0	n/a
23	97.3	66.9	98.8	23	n/a	100.0	n/a	23	83.3	100.0	95.3	23	n/a	100.0	n/a
24	97.3	66.9	98.7	24	n/a	100.0	n/a	24	83.3	100.0	95.0	24	n/a	100.0	n/a
25	97.3	66.9	98.7	25	n/a	100.0	n/a	25	83.3	100.0	94.7	25	n/a	100.0	n/a
26	97.3	66.9	98.6	26	n/a	100.0	n/a	26	83.3	100.0	94.5	26	n/a	100.0	n/a

27	97.3	66.9	98.5	27	n/a	100.0	n/a	27	83.3	100.0	94.2	27	n/a	100.0	n/a
28	97.3	66.9	98.4	28	n/a	100.0	n/a	28	83.3	100.0	93.9	28	n/a	100.0	n/a
29	97.3	66.9	98.4	29	n/a	100.0	n/a	29	83.3	100.0	93.6	29	n/a	100.0	n/a
30	97.3	66.9	98.3	30	n/a	100.0	n/a	30	83.3	100.0	93.3	30	n/a	100.0	n/a
31	97.3	66.9	98.2	31	n/a	100.0	n/a	31	83.3	100.0	93.0	31	n/a	100.0	n/a
32	97.3	66.9	98.1	32	n/a	100.0	n/a	32	83.3	100.0	92.7	32	n/a	100.0	n/a
33	97.3	66.9	98.0	33	n/a	100.0	n/a	33	83.3	100.0	92.4	33	n/a	100.0	n/a
34	97.3	66.9	97.9	34	n/a	100.0	n/a	34	83.3	100.0	92.1	34	n/a	100.0	n/a
35	97.3	66.9	97.8	35	n/a	100.0	n/a	35	83.3	100.0	91.8	35	n/a	100.0	n/a
36	97.3	66.9	97.7	36	n/a	100.0	n/a	36	83.3	100.0	91.4	36	n/a	100.0	n/a
37	97.3	66.9	97.6	37	n/a	100.0	n/a	37	83.3	100.0	91.1	37	n/a	100.0	n/a
38	97.3	66.9	97.5	38	n/a	100.0	n/a	38	83.3	100.0	90.7	38	n/a	100.0	n/a
39	97.3	66.9	97.4	39	n/a	100.0	n/a	39	83.3	100.0	90.4	39	n/a	100.0	n/a
40	97.3	66.9	97.3	40	n/a	100.0	n/a	40	83.3	100.0	90.0	40	n/a	100.0	n/a
41	97.3	66.9	97.2	41	n/a	100.0	n/a	41	83.3	100.0	89.6	41	n/a	100.0	n/a
42	97.3	66.9	97.1	42	n/a	100.0	n/a	42	83.3	100.0	89.2	42	n/a	100.0	n/a
43	97.3	66.9	97.0	43	n/a	100.0	n/a	43	83.3	100.0	88.8	43	n/a	100.0	n/a
44	97.3	66.9	96.9	44	n/a	100.0	n/a	44	83.3	100.0	88.4	44	n/a	100.0	n/a
45	97.3	66.9	96.8	45	n/a	100.0	n/a	45	83.3	100.0	88.0	45	n/a	100.0	n/a
46	97.3	66.9	96.6	46	n/a	100.0	n/a	46	83.3	100.0	87.6	46	n/a	100.0	n/a
47	97.3	66.9	96.5	47	n/a	100.0	n/a	47	83.3	100.0	87.1	47	n/a	100.0	n/a
48	97.3	66.9	96.4	48	n/a	100.0	n/a	48	83.3	100.0	86.7	48	n/a	100.0	n/a
49	97.3	66.9	96.2	49	n/a	100.0	n/a	49	83.3	100.0	86.2	49	n/a	100.0	n/a
50	97.3	66.9	96.1	50	n/a	100.0	n/a	50	83.3	100.0	85.7	50	n/a	100.0	n/a
51	97.3	66.9	95.9	51	n/a	100.0	n/a	51	83.3	100.0	85.2	51	n/a	100.0	n/a
52	97.3	66.9	95.7	52	n/a	100.0	n/a	52	83.3	100.0	84.7	52	n/a	100.0	n/a
53	97.3	66.9	95.6	53	n/a	100.0	n/a	53	83.3	100.0	84.2	53	n/a	100.0	n/a
54	97.3	66.9	95.4	54	n/a	100.0	n/a	54	83.3	100.0	83.6	54	n/a	100.0	n/a
55	97.3	66.9	95.2	55	n/a	100.0	n/a	55	83.3	100.0	83.1	55	n/a	100.0	n/a
56	97.3	66.9	95.0	56	n/a	100.0	n/a	56	83.3	100.0	82.5	56	n/a	100.0	n/a
57	97.3	66.9	94.8	57	n/a	100.0	n/a	57	83.3	100.0	81.9	57	n/a	100.0	n/a
58	97.3	66.9	94.6	58	n/a	100.0	n/a	58	83.3	100.0	81.3	58	n/a	100.0	n/a
59	97.3	66.9	94.4	59	n/a	100.0	n/a	59	83.3	100.0	80.7	59	n/a	100.0	n/a
60	97.3	66.9	94.2	60	n/a	100.0	n/a	60	83.3	100.0	80.0	60	n/a	100.0	n/a
61	97.3	66.9	94.0	61	n/a	100.0	n/a	61	83.3	100.0	79.3	61	n/a	100.0	n/a
62	97.3	66.9	93.7	62	n/a	100.0	n/a	62	83.3	100.0	78.6	62	n/a	100.0	n/a
63	97.3	66.9	93.5	63	n/a	100.0	n/a	63	83.3	100.0	77.9	63	n/a	100.0	n/a
64	97.3	66.9	93.2	64	n/a	100.0	n/a	64	83.3	100.0	77.1	64	n/a	100.0	n/a
65	97.3	66.9	92.9	65	n/a	100.0	n/a	65	83.3	100.0	76.4	65	n/a	100.0	n/a
66	97.3	66.9	92.6	66	n/a	100.0	n/a	66	83.3	100.0	75.6	66	n/a	100.0	n/a
67	97.3	66.9	92.3	67	n/a	100.0	n/a	67	83.3	100.0	74.7	67	n/a	100.0	n/a
68	97.3	66.9	92.0	68	n/a	100.0	n/a	68	83.3	100.0	73.8	68	n/a	100.0	n/a
69	97.3	66.9	91.6	69	n/a	100.0	n/a	69	83.3	100.0	72.9	69	n/a	100.0	n/a
70	97.3	66.9	91.3	70	n/a	100.0	n/a	70	83.3	100.0	72.0	70	n/a	100.0	n/a
71	97.3	66.9	90.9	71	n/a	100.0	n/a	71	83.3	100.0	71.0	71	n/a	100.0	n/a
72	97.3	66.9	90.5	72	n/a	100.0	n/a	72	83.3	100.0	70.0	72	n/a	100.0	n/a
73	97.3	66.9	90.0	73	n/a	100.0	n/a	73	83.3	100.0	68.9	73	n/a	100.0	n/a
74	97.3	66.9	89.5	74	n/a	100.0	n/a	74	83.3	100.0	67.8	74	n/a	100.0	n/a
75	97.3	66.9	89.0	75	n/a	100.0	n/a	75	83.3	100.0	66.7	75	n/a	100.0	n/a
76	97.3	66.9	88.5	76	n/a	100.0	n/a	76	83.3	100.0	65.5	76	n/a	100.0	n/a
77	97.3	66.9	87.9	77	n/a	100.0	n/a	77	83.3	100.0	64.2	77	n/a	100.0	n/a
78	97.3	66.9	87.3	78	n/a	100.0	n/a	78	83.3	100.0	62.9	78	n/a	100.0	n/a
79	97.3	66.9	86.6	79	n/a	100.0	n/a	79	83.3	100.0	61.5	79	n/a	100.0	n/a
80	97.3	66.9	85.9	80	n/a	100.0	n/a	80	83.3	100.0	60.0	80	n/a	100.0	n/a
81	97.3	66.9	85.1	81	n/a	100.0	n/a	81	83.3	100.0	58.5	81	n/a	100.0	n/a
82	97.3	66.9	84.3	82	n/a	100.0	n/a	82	83.3	100.0	56.8	82	n/a	100.0	n/a
83	97.3	66.9	83.3	83	n/a	100.0	n/a	83	83.3	100.0	55.1	83	n/a	100.0	n/a
84	97.3	66.9	82.3	84	n/a	100.0	n/a	84	83.3	100.0	53.3	84	n/a	100.0	n/a
85	97.3	66.9	81.1	85	n/a	100.0	n/a	85	83.3	100.0	51.4	85	n/a	100.0	n/a
86	97.3	66.9	79.9	86	n/a	100.0	n/a	86	83.3	100.0	49.4	86	n/a	100.0	n/a
87	97.3	66.9	78.5	87	n/a	100.0	n/a	87	83.3	100.0	47.3	87	n/a	100.0	n/a
88	97.3	66.9	76.9	88	n/a	100.0	n/a	88	83.3	100.0	45.0	88	n/a	100.0	n/a
89	97.3	66.9	75.1	89	n/a	100.0	n/a	89	83.3	100.0	42.6	89	n/a	100.0	n/a
90	97.3	66.9	73.0	90	n/a	100.0	n/a	90	83.3	100.0	40.0	90	n/a	100.0	n/a
91	97.3	66.9	70.7	91	n/a	100.0	n/a	91	83.3	100.0	37.2	91	n/a	100.0	n/a
92	97.3	66.9	68.0	92	n/a	100.0	n/a	92	83.3	100.0	34.3	92	n/a	100.0	n/a
93	97.3	66.9	64.7	93	n/a	100.0	n/a	93	83.3	100.0	31.1	93	n/a	100.0	n/a
94	97.3	66.9	60.9	94	n/a	100.0	n/a	94	83.3	100.0	27.7	94	n/a	100.0	n/a
95	97.3	66.9	56.2	95	n/a	100.0	n/a	95	83.3	100.0	24.0	95	n/a	100.0	n/a
96	97.3	66.9	50.4	96	n/a	100.0	n/a	96	83.3	100.0	20.0	96	n/a	100.0	n/a
97	97.3	66.9	43.0	97	n/a	100.0	n/a	97	83.3	100.0	15.7	97	n/a	100.0	n/a
98	97.3	66.9	33.2	98	n/a	100.0	n/a	98	83.3	100.0	10.9	98	n/a	100.0	n/a
99	97.3	66.9	19.8	99	n/a	100.0	n/a	99	83.3	100.0	5.7	99	n/a	100.0	n/a
100	97.3	66.9	0.0	100	n/a	100.0	n/a	100	83.3	100.0	0.0	100	n/a	100.0	n/a

S4

	Resistant phenotype, n (%)					Susceptible phenotype, n (%)															
	R	S	U	F	Total	R	S	U	F	Total	PPV, (%)	NPV, (%)	Sensitivity (predicitons only) (%)	Sensitivity (all) (%)	Specificity (predicitons only) (%)	Specificity (all), (%)	No genotypic prediction made, (%)	Resistance prevalence (all) (%)	Resistance prevalence (predictions only) (%)	Difference in sensitivity vs. non deduplicated results (p-value)	Difference in specificity vs. non deduplicated results (p-value)
Deduplicated for genomically identical samples																					
Isoniazid	2,672	84	84	32	2,872	61	5,502	203	108	5,874	97.8	98.5	97.0	93.0	98.9	93.7	4.9	32.8	33.1	0.7	0.7
Rifampicin	2,388	65	6	68	2,527	77	5,935	190	137	6,339	96.9	98.9	97.4	94.5	98.7	93.6	4.5	28.5	29.0	0.7	0.9
Ethambutol	1,226	73	86	39	1,424	409	5,979	709	64	7,161	75.0	98.8	94.4	86.1	93.6	83.5	10.5	16.6	16.9	0.9	1.0
Pyrazinamide	740	77	105	59	981	168	5,436	170	86	5,860	81.5	98.6	90.6	75.4	97.0	92.8	6.1	14.3	12.7	0.6	0.5
Deduplicated for isolates within 5 SNPs																					
Isoniazid	2,112	77	75	24	2,288	34	4,741	181	102	5,058	98.4	98.4	96.5	92.3	99.3	93.7	5.2	31.1	31.4	0.2	0.1
Rifampicin	1,868	56	5	54	1,983	61	5,145	144	116	5,466	96.8	98.9	97.1	94.2	98.8	94.1	4.3	26.6	27.0	0.4	0.8
Ethambutol	907	67	69	31	1,074	309	5,156	627	50	6,142	74.6	98.7	93.1	84.5	94.3	83.9	10.8	14.9	15.1	0.1	0.1
Pyrazinamide	575	67	72	48	762	140	4,770	89	66	5,065	80.4	98.6	89.6	75.5	97.1	94.2	4.7	13.1	11.6	0.3	0.3

S5

S5: Drug profile predictions for isolates with complete profiles

	Prediction													
	RRRR	RRRS	RRSR	RRSS	RSRR	RSRS	RSSR	RSSS	SRRR	SRRS	SRSS	SSRS	SSSR	SSSS	Total
Phenotype															
RRRR	488	10	6	0	0	1	0	0	1	0	0	0	0	0	506
RRRS	95	158	3	10	0	4	1	1	0	0	1	0	0	0	273
RRSR	105	12	33	5	0	0	2	1	0	0	0	0	0	0	158
RRSS	35	92	9	176	0	0	0	4	0	1	5	0	0	11	333
RSRR	8	1	0	0	1	0	0	1	0	0	0	0	0	0	11
RSRS	0	2	0	0	0	18	0	2	0	0	0	0	0	3	25
RSSR	1	0	1	0	1	0	8	4	0	0	0	0	0	1	16
RSSS	2	4	0	8	0	22	2	306	0	0	0	0	0	25	369
SRRS	0	2	0	0	0	0	0	0	0	0	2	0	0	0	4
SRSR	0	0	0	0	0	0	0	0	0	0	1	0	0	2	3
SRSS	0	1	0	5	0	0	0	0	0	0	59	0	0	16	81
SSRS	0	0	0	0	0	0	0	0	0	0	1	0	0	7	8
SSSR	0	0	0	0	0	0	0	0	0	0	0	0	51	20	71
SSSS	0	0	0	1	0	0	0	18	1	0	23	2	10	3,952	4,007
Total	734	282	52	205	2	45	13	337	2	1	92	2	61	4,037	5,865

All profiles are presented in the following order: Isoniazid, Rifampicin, Ethabutol, Pyrazinamide, with S indicating susceptibility and R indicating resitance.

S6

Result for isolates with full phenotypic drug profiles:

	Resistant phenotype, n (%)					Susceptible phenotype, n (%)													
	R	S	U	F	Total	R	S	U	F	Total	PPV, (%)	NPV, (%)	Sensitivity (predictions only) (%)	Sensitivity (all) (%)	Specificity (predictions only) (%)	Specificity (all), (%)	No genotypic prediction made, (%)	Resistance prevalence (all) (%)	Resistance prevalence (predictions only) (%)
All isolates																			
Isoniazid	2,214	65	70	34	2,383	30	4,848	147	108	5,133	98.7	98.7	97.1	92.9	99.4	94.4	4.8	31.7	31.8
Rifampicin	1,852	51	3	61	1,967	70	5,213	133	133	5,549	96.4	99.0	97.3	94.2	98.7	93.9	4.4	26.2	26.5
Ethambutol	1,038	47	61	39	1,185	397	5,263	611	60	6,331	72.3	99.1	95.7	87.6	93.0	83.1	10.3	15.8	16.1
Pyrazinamide	795	81	110	74	1,060	173	5,989	188	106	6,456	82.1	98.7	90.8	75.0	97.2	92.8	6.4	14.1	12.4
Result for isolates with full phenotypic profiles for the collections that are not enriched for resistance:																			
Isoniazid	268	7	9	4	288	15	3,639	101	86	3,841	94.7	99.8	97.5	93.1	99.6	94.7	4.8	7.0	7.0
Rifampicin	91	0	0	8	99	28	3,794	100	108	4,030	76.5	100.0	100.0	91.9	99.3	94.1	5.2	2.4	2.3
Ethambutol	59	0	0	0	59	39	3,566	431	34	4,070	60.2	100.0	100.0	100.0	98.9	87.6	11.3	1.4	1.6
Pyrazinamide	99	6	4	5	114	26	3,917	14	58	4,015	79.2	99.8	94.3	86.8	99.3	97.6	2.0	2.8	2.6

S7

Drug profile predictions for collections from Germany, Italy, the Netherlands and the UK that are unenriched for resistance. Includes isolates with uncharacterised variants relevant to rifampicin, ethambutol or pyrazinamide.

Prediction	Genotypic drug profiles				Phenotype						Predictions
	Inh	Rif	Emb	Pza	Pan-susceptible	Some resistance	Sensitivity %	Specificity %	PPV %	NPV %	made %
(a) Predicted pan-susceptible	S	S	S	S	3,008	9					
(b) Predicted pan-susceptible after inferring that 'U' mutations are consistent with susceptibility in this context	S	S	S	U	9	0					
	S	S	U	S	331	2					
	S	S	U	U	2	0					
	S	U	S	S	64	0					
	S	U	U	S	25	0					
	Total				3,439	11					
(c) Predicted to have some phenotypic resistance	R	S	R or S		12	145					
	S	R or S			25	47					
	R	R	R or S		1	77					
	Total				38	269					
Results for (a) and (c)							96.8	98.8	87.6	99.7	80.5
Results for (a), (b) and (c)							96.1	98.9	87.6	99.7	91.0
No prediction made (incomplete prediction)	U	S or U			91	6					
	At least one F, no R				193	6					
	At least one R and U, no F				4	43					
	At least one R and F, no U				3	24					
	At least one R, U, and F				0	2					
	Total				291	81					

PPV = Positive Predictive Value; NPV = Negative Predictive Value; Inh=Isoniazid; Rif=Rifampicin; Emb=Ethambutol; Pza=Pyrazinamide; R=resistant; S=susceptible; U=mutation of unknown association present; F=genotypic prediction failed due to missing data around a genomic resistance locus. Sensitivity, specificity, NPV and PPV are calculated including and excluding predictions of pan-susceptibility for isolates containing a 'U' mutation.

S8

Isolates for which the phenotypes could be re-tested or cross-checked:

Source	Labnumber	Drug	Original reported phenotype	Mutation	Phenotype after re-testing	Results of cross-checking source database
University of Sydney	S2	Ethambutol	S	embB_G406D	Not growing	
University of Sydney	S3	Ethambutol	S	embB_M306V	R	
University of Sydney	S7	Ethambutol	S	embB_M306V	R	
University of Sydney	S8	Ethambutol	S	embB_M306I	R	
University of Sydney	S11	Ethambutol	S	embb_M306I, embB_G406S	R	
University of Sydney	S15	Ethambutol	S	embB_M306I	S	
University of Sydney	S20	Ethambutol	S	embB_M306V	Contaminated with Bacillus	
University of Sydney	S23	Ethambutol	S	embB_G406D	Could not be tested	
University of Sydney	S30	Ethambutol	S	embB_D354A	R	
University of Sydney	S33	Ethambutol	S	embB_M306V	R	
University of Sydney	S41	Ethambutol	S	embB_G406A	R	
Harvard	NLA000016764	Ethambutol	R			Clerical error - no phenotype was done for Ethambutol
Harvard	NLA000801694	Isoniazid	R			Clerical error - was tested as susceptible
Harvard	NLA000801697	Isoniazid	R			Clerical error - was tested as susceptible
Harvard	NLA000801694	Rifampicin	R			Clerical error - was tested as susceptible
Harvard	NLA000801697	Rifampicin	R			Clerical error - was tested as susceptible
Netherlands	NL491	Ethambutol	R			Clerical error - this was reported as susceptible by MGIT, resistant by MIC plate (10 mg/l)
Netherlands	NL294	Ethambutol	S	embB_M306I		Susceptible by MGIT, resistant by MIC plate (10 mg/l)
Netherlands	NL241	Ethambutol	S	embB_M306V		Susceptible by MGIT, resistant by MIC plate (10 mg/l)
Netherlands	NL294	Pyrazinamide	S	pncA_F58L		Susceptible in MGIT at 100mh/l (x3) but resistant at 25mg/l (x1) and 50mg/l (x1), therefore can be considered intermediate
Peru	14722_6_10	Rifampicin	R		S	
Peru	14722_6_13	Isoniazid	S	fabG1_C-15T	S	
Peru	14722_6_21	Isoniazid	R		S	
Peru	14722_6_36	Rifampicin	S	rpoB_D435F	R	
Peru	14722_6_38	Isoniazid	S	fabG1_C-15T	S	
Peru	14722_6_41	Rifampicin	S	rpoB_D435F	R	
Peru	14722_6_59	Isoniazid	S	fabG1_G-17T	S	
Peru	14722_6_86	Isoniazid	S	fabG1_C-15T	S	
Peru	14892_2_37	Rifampicin	S	rpoB_D435V	R	
Peru	14892_2_59	Isoniazid	S	fabG1_G-17T	S	
Peru	14893_2_23	Isoniazid	S	fabG1_C-15T	S	
Peru	14893_2_45	Isoniazid	S	fabG1_C-15T	S	
Peru	14893_2_48	Isoniazid	S	fabG1_C-15T	S	
Peru	14893_2_52	Isoniazid	S	fabG1_G-17T	S	
Peru	14893_2_64	Isoniazid	R		R	
Peru	15277_3_57	Isoniazid	S	fabG1_C-15T	S	

S9

Performance over whole data set of variants consistent with susceptibility that were present in phenotypically resistant isolates. Variants only shown where they feature as the only variant relevant to a drug in an isolate

Drug	Variant	Phenotypically resistant	Phenotypically susceptible
Isoniazid	ahpC_C-52T	1	0
Isoniazid	fabG1_S126N	1	5
Isoniazid	inhA_C-40T	1	208
Isoniazid	katG_C-85T	3	63
Isoniazid	katG_T475I	1	10
Ethambutol	embA_G-43C	3	4
Ethambutol	embB_D1024N	5	5
Ethambutol	embB_D328H	2	3
Ethambutol	embB_E504D	2	0
Ethambutol	embB_G-6A	1	26
Ethambutol	embB_H1002R	7	7
Ethambutol	embB_Q497P	2	3
Ethambutol	embB_T1082A	1	18
Ethambutol	embC_A774S	1	29
Pyrazinamide	pncA_D63A	2	0

Performance over whole data set of resistance variants that were found in susceptible isolates. Variants only counted where they feature as the only resistant-variant relevant to a drug in an isolate

Drug	Variant	Phenotypically reisstant	Phenotypically susceptible
Isoniazid	fabG1_C-15T	228	21
Isoniazid	fabG1_G-17T	7	3
Isoniazid	fabG1_L203L	55	28
Isoniazid	fabG1_T-8A	7	3
Isoniazid	fabG1_T-8C	4	1
Isoniazid	inhA_I21T	1	1
Isoniazid	inhA_S94A	4	3
Isoniazid	katG_1438_indel	0	1
Isoniazid	katG_2005_indel	2	1
Isoniazid	katG_606_indel	0	1
Rifampicin	rpoB_D435A	0	1
Rifampicin	rpoB_D435F	8	2
Rifampicin	rpoB_D435G	2	2
Rifampicin	rpoB_D435V	181	2
Rifampicin	rpoB_D435Y	29	13
Rifampicin	rpoB_D545E	0	3
Rifampicin	rpoB_H445N	9	10
Rifampicin	rpoB_H445R	24	1
Rifampicin	rpoB_H445Y	141	2
Rifampicin	rpoB_I491F	40	7
Rifampicin	rpoB_L430P	12	22
Rifampicin	rpoB_L452M	0	1
Rifampicin	rpoB_L452P	39	9
Rifampicin	rpoB_M434I	1	0
Rifampicin	rpoB_Q432P	6	1
Rifampicin	rpoB_S450W	56	1
Rifampicin	rpoB_V359A	0	2
Ethambutol	embA_C-12T	28	24
Ethambutol	embA_C-16G	22	3
Ethambutol	embA_C-16T	12	7
Ethambutol	embB_D328Y	8	2
Ethambutol	embB_D354A	107	93
Ethambutol	embB_G406A	53	21
Ethambutol	embB_G406D	36	38
Ethambutol	embB_G406S	19	3
Ethambutol	embB_M306I	320	130
Ethambutol	embB_M306L	26	10
Ethambutol	embB_M306V	512	98
Ethambutol	embB_Q497K	15	6
Ethambutol	embB_Q497R	122	28
Pyrazinamide	pncA_-30_indel	0	1
Pyrazinamide	pncA_-3_indel	0	3
Pyrazinamide	pncA_-5_indel	7	4
Pyrazinamide	pncA_-79_indel	1	1
Pyrazinamide	pncA_-868_indel	0	1
Pyrazinamide	pncA_189_indel	0	1
Pyrazinamide	pncA_193_indel	2	1
Pyrazinamide	pncA_220_indel	0	1
Pyrazinamide	pncA_256_indel	1	1
Pyrazinamide	pncA_303_indel	0	1
Pyrazinamide	pncA_382_indel	1	1
Pyrazinamide	pncA_386_indel	3	1
Pyrazinamide	pncA_391_indel	19	1
Pyrazinamide	pncA_407_indel	4	1
Pyrazinamide	pncA_408_indel	1	2
Pyrazinamide	pncA_417_indel	3	1
Pyrazinamide	pncA_478_indel	0	1
Pyrazinamide	pncA_501_indel	0	1
Pyrazinamide	pncA_62_indel	0	1
Pyrazinamide	pncA_80_indel	0	1
Pyrazinamide	pncA_A-11G	38	14
Pyrazinamide	pncA_A102P	3	1
Pyrazinamide	pncA_A134V	4	1
Pyrazinamide	pncA_A143D	1	1
Pyrazinamide	pncA_A146T	1	1
Pyrazinamide	pncA_A171E	0	1
Pyrazinamide	pncA_A171T	1	5
Pyrazinamide	pncA_A46V	2	1
Pyrazinamide	pncA_C14R	6	2
Pyrazinamide	pncA_D12A	4	9
Pyrazinamide	pncA_D12E	2	1
Pyrazinamide	pncA_D12G	3	1
Pyrazinamide	pncA_D136N	0	2
Pyrazinamide	pncA_D49G	3	1
Pyrazinamide	pncA_D63G	0	4
Pyrazinamide	pncA_D63H	1	1

Pyrazinamide	pncA_D8G	6	1
Pyrazinamide	pncA_D8N	2	1
Pyrazinamide	pncA_F13V	0	2
Pyrazinamide	pncA_F58L	3	3
Pyrazinamide	pncA_F81S	0	2
Pyrazinamide	pncA_F81V	0	2
Pyrazinamide	pncA_F94C	2	1
Pyrazinamide	pncA_F94L	2	3
Pyrazinamide	pncA_G108R	2	1
Pyrazinamide	pncA_G124S	0	1
Pyrazinamide	pncA_G132A	4	2
Pyrazinamide	pncA_G17D	1	1
Pyrazinamide	pncA_G24D	4	1
Pyrazinamide	pncA_G78C	0	1
Pyrazinamide	pncA_G97D	24	4
Pyrazinamide	pncA_H51R	12	1
Pyrazinamide	pncA_H57D	70	3
Pyrazinamide	pncA_H71R	2	4
Pyrazinamide	pncA_H71Y	17	3
Pyrazinamide	pncA_I31T	0	1
Pyrazinamide	pncA_I5T	0	1
Pyrazinamide	pncA_K48E	1	1
Pyrazinamide	pncA_K48T	6	8
Pyrazinamide	pncA_K96Q	0	2
Pyrazinamide	pncA_K96R	8	2
Pyrazinamide	pncA_L116P	2	1
Pyrazinamide	pncA_L151S	8	4
Pyrazinamide	pncA_L172P	5	1
Pyrazinamide	pncA_L182S	1	3
Pyrazinamide	pncA_L19P	3	1
Pyrazinamide	pncA_L27P	4	2
Pyrazinamide	pncA_L35P	2	4
Pyrazinamide	pncA_L4S	20	5
Pyrazinamide	pncA_L4W	1	3
Pyrazinamide	pncA_L85P	3	1
Pyrazinamide	pncA_L85R	5	2
Pyrazinamide	pncA_M175I	0	2
Pyrazinamide	pncA_M175V	4	2
Pyrazinamide	pncA_P54L	19	3
Pyrazinamide	pncA_P62S	1	3
Pyrazinamide	pncA_P69S	0	1
Pyrazinamide	pncA_S104R	3	2
Pyrazinamide	pncA_S65P	0	1
Pyrazinamide	pncA_S67P	8	1
Pyrazinamide	pncA_T135I	0	1
Pyrazinamide	pncA_T135P	15	1
Pyrazinamide	pncA_T135S	0	1
Pyrazinamide	pncA_T142M	3	1
Pyrazinamide	pncA_T168S	0	2
Pyrazinamide	pncA_T47A	1	3
Pyrazinamide	pncA_T47I	0	2
Pyrazinamide	pncA_V125F	0	2
Pyrazinamide	pncA_V128G	9	2
Pyrazinamide	pncA_V139A	8	8
Pyrazinamide	pncA_V139G	5	1
Pyrazinamide	pncA_V139L	2	1
Pyrazinamide	pncA_V21A	0	1
Pyrazinamide	pncA_V7A	0	1
Pyrazinamide	pncA_V7F	2	1
Pyrazinamide	pncA_V7G	13	2
Pyrazinamide	pncA_V7L	1	1
Pyrazinamide	pncA_W119R	2	2
Pyrazinamide	pncA_W68G	14	1
Pyrazinamide	pncA_W68R	6	1
Pyrazinamide	pncA_Y103*	12	2
Pyrazinamide	pncA_Y41*	0	1

S10

For each of isoniazid and rifampicin, calculate the expected mislabelling rate based on isolates with mutations (rpoB S450L or katG S315T) but a susceptible phenotype.
For these calculations, the collection from Russia was excluded for isoniazid as the katG S315T mutations with a susceptible phenotype had already been excluded at source (in a previous publication).
The isolates inlcuded below inlcude all those that were excluded from the main analysis after being identified as likely mislabelling errors.
Expected:
(Total number of isolates with a susceptible phenotype x number with the mutation and a susceptible phenotype) / (sum of isolates with the mutation) x ((1-prevalence of resistance)/prevalence of resistance)

The ratio of expected to observed errors was then taken as the proportion of discrepant isolates likely to be due to mislabelling

		Phenotypes			Where a katG_S315T or rpoB_S450L mutation is present		Among the susceptible phenotypes		Errors (predicted resistant but phenotypically susceptible)		
Drug	Source (see S1)	Resistant	Susceptible	Prevalence of resistance	Resistant phenotype	Susceptible phenotype	Predicted susceptible	Predicted resistant	Expected errors	Observed errors	Ratio (expected / observed)
Isoniazid	University of Sydney	42	0	100.00	31	0	0	0	.	0	
Rifampicin	University of Sydney	38	4	90.48	21	0	4	0	0.00	0	
Isoniazid	Genoscreen (isolates from Belgium)	100	135	42.55	86	0	127	0	0.00	0	
Rifampicin	Genoscreen (isolates from Belgium)	98	137	41.70	74	0	128	1	0.00	1	
Isoniazid	Birmingham	259	3,115	7.68	168	9	2,953	19	13.17	19	
Rifampicin	Birmingham	100	3,295	2.95	58	1	3,088	29	1.69	29	
Isoniazid	British Columbia Centre for Disease Control	189	1,152	14.09	70	0	1,080	19	0.00	19	
Rifampicin	British Columbia Centre for Disease Control	38	1,304	2.83	18	0	1,208	4	0.00	4	
Isoniazid	Hamburg	23	253	8.33	17	2	241	6	2.42	6	
Rifampicin	Hamburg	15	261	5.43	12	0	254	3	0.00	3	
Isoniazid	Italy	130	0	100.00	121	0	0	0	.	0	
Rifampicin	Italy	128	2	98.46	91	0	2	0	0.00	0	
Isoniazid	Italy_MGITstudy	11	84	11.58	7	0	74	0	0.00	0	
Rifampicin	Italy_MGITstudy	4	91	4.21	2	0	79	0	0.00	0	
Isoniazid	Leeds	3	28	9.68	3	0	25	0	0.00	0	
Rifampicin	Leeds	3	28	9.68	3	0	25	0	0.00	0	
Isoniazid	London	358	21	94.46	312	0	17	0	0.00	0	
Rifampicin	London	350	23	93.83	248	0	20	0	0.00	0	
Isoniazid	Harvard (isolates from the Netherlands)	302	38	88.82	218	1	34	1	1.38	1	
Rifampicin	Harvard (isolates from the Netherlands)	292	47	86.14	171	1	38	8	1.70	8	
Isoniazid	Netherlands	36	484	6.92	33	1	456	2	1.06	2	
Rifampicin	Netherlands	14	514	2.65	13	0	490	2	0.00	2	
Isoniazid	Oxford	6	29	17.14	2	0	23	0	0.00	0	
Rifampicin	Oxford	2	33	5.71	2	0	30	0	0.00	0	
Isoniazid	Pakistan	357	62	85.20	273	3	54	5	3.88	5	
Rifampicin	Pakistan	352	67	84.01	201	1	57	8	1.74	8	
Isoniazid	Peru	85	32	72.65	78	2	18	12	2.13	12	
Rifampicin	Peru	64	53	54.70	40	0	45	4	0.00	4	
Isoniazid	Serbia	107	0	100.00	85	0	0	0	.	0	
Rifampicin	Serbia	107	0	100.00	59	0	0	0	.	0	
Isoniazid	Yang et. al. Lancet Infect Dis. 2017 Mar;17(3):275-284 (isolates from China)	121	0	100.00	102	0	0	0	.	0	
Rifampicin	Yang et. al. Lancet Infect Dis. 2017 Mar;17(3):275-284 (isolates from China)	121	0	100.00	72	0	0	0	.	0	
Isoniazid	South Africa	197	701	21.94	110	7	655	13	11.79	13	
Rifampicin	South Africa	337	670	33.47	182	2	629	12	3.66	12	
Isoniazid	Spain	13	51	20.31	7	0	50	0	0.00	0	
Rifampicin	Spain	10	54	15.63	7	0	52	0	0.00	0	
Isoniazid	MSF (isolates from Swaziland)	137	136	50.18	115	0	121	8	0.00	8	
Rifampicin	MSF (isolates from Swaziland)	120	146	45.11	57	0	143	2	0.00	2	
Isoniazid	Thailand	199	58	77.43	157	1	53	1	1.26	1	
Rifampicin	Thailand	196	61	76.26	89	0	56	1	0.00	1	
Isoniazid	Zhang et. al. Nat Genet. 2013 Oct;45(10):1255-60 (isolates from China)	117	44	72.67	65	0	40	0	0.00	0	
Rifampicin	Zhang et. al. Nat Genet. 2013 Oct;45(10):1255-60 (isolates from China)	117	44	72.67	56	0	43	1	0.00	1	

Totals	Expected	Observed	Ratio (%)
Isoniazid	37.08	86	43.12
Rifampicin	8.80	75	11.73

Isolates from Russia (from Casali et. al. Nat Genet. 2014 Mar;46(3):279-86) were excluded from the calculation as isolates with a katG_S315T mutation and a susceptible phenotype for isoniazid were excluded from the publication from which these isolates were derived.
